# Global, regional, and national cascades of diabetes care, 2000–2023: diagnosis, treatment, and glycaemic management

**DOI:** 10.1016/S2213-8587(25)00217-7

**Published:** 2025-09-09

**Authors:** Lauryn K Stafford, Anna Gage, Yvonne Yiru Xu, Madeleine Conrad, Ismael Barreras Beltran, Edward J Boyko, Bruce B Duncan, Simon I Hay, Hailey Lenox, Rafael Lozano, Dianna J Magliano, Carlos A Aguilar Salinas, Nikhil Tandon, Pedro Zitko, Christopher JL Murray, Theo Vos, Annie Haakenstad, Kanyin Liane Ong

**Affiliations:** 1. Institute for Health Metrics and Evaluation, University of Washington, Seattle, WA, USA; 2. School of Medicine, University of Washington, Seattle, WA, USA; 3. General Medicine Service, Department of Veterans Affairs, Seattle WA, USA; 4. Postgraduate Program in Epidemiology, Federal University of Rio Grande do Sul, Porto Alegre, Brazil; 5. Department of Health Metrics Sciences, School of Medicine, University of Washington, Seattle, WA, USA; 6. Department of Public Health, School of Medicine, National Autonomous University of Mexico, Mexico City, Mexico; 7. School of Public Health and Preventive Medicine, Monash University, Melbourne, VIC, Australia; 8. Diabetes and Population Health Laboratory, Baker Heart and Diabetes Institute, Melbourne, VIC, Australia; 9. Departamento de Endocrinología y Metabolismo, Instituto Nacional de Ciencias Médicas y Nutrición Salvador Zubirán, Mexico City, Mexico; 10. All India Institute of Medical Sciences, Department of Endocrinology & Metabolism, Biotechnology Block, New Delhi, India; 11. Health Service & Population Research Department, IoPPN, King’s College London, London, UK; 12. Departamento de Salud Global, Escuela de Salud Pública, Universidad de Chile, Santiago, Chile

## Abstract

**Background:**

Diabetes is a serious global health challenge, with a rising prevalence and substantial impact on disability and mortality worldwide. Despite medical advancements, gaps in the cascade of diabetes care—comprising diagnosis, treatment, and maintaining optimal glycaemic levels—persist, hindering effective management. This study comprehensively assesses the state of the diabetes cascade of care globally, identifying areas of strength and needs for improvement in diabetes management.

**Methods:**

Using Global Burden of Diseases, Injuries, and Risk Factors Study (GBD) data and methods, this analysis spanned the years 2000 to 2023 and covered 204 countries and territories. We systematically reviewed cross-sectional surveys that are representative of the general population and the published and grey literature to estimate the proportion of people with diabetes who are undiagnosed, diagnosed but untreated, treated with suboptimal glycaemic levels, and treated with optimal glycaemic levels. Treatment was defined as current use of insulin or other hypoglycaemic medication. We separately modelled these quantities by location, year, age, and sex, then scaled the estimates so that they sum to 100% of people living with diabetes in each stratum. Using GBD 2023 estimates of the number of people with diabetes, we calculated the diabetes cascade of care: proportion diagnosed among those with diabetes, proportion treated among those with diagnosed diabetes, and proportion with optimal glycaemic levels among those with treated diabetes across all strata.

**Findings:**

In 2023, an estimated 55.8% (95% UI 49.3–62.3) of people with diabetes aged 15 years and older were diagnosed with diabetes globally. The proportion of people with diagnosed diabetes who were on treatment was 91.4% (88.0–94.2), and the proportion of people on diabetes treatment with optimal glycaemic levels was 41.6% (35.7–48.5). Among all people with diabetes, the proportion with optimal glycaemic levels on treatment was 21.2% (17.4–25.6) in 2023 globally. Substantial regional differences were observed, with the highest rates of diagnosis in high-income North America, the highest rates of treatment among those with diagnosed diabetes in high-income Asia Pacific, and the highest rates of optimal glycaemic levels among those with treated diabetes in southern Latin America. Between 2000 and 2023 globally, the proportion of people diagnosed with diabetes increased by 8.3 (6.6–10.0) percentage points, while the treated patients among the diagnosed increased by 7.2 (5.7–8.8) percentage points. The proportion of people with treated diabetes who had optimal glycaemic levels increased by 1.3 (0.8–1.8) percentage points.

**Interpretation:**

Despite improvements over the past two decades, underdiagnosis and suboptimal glycaemic management of diabetes remain major challenges globally, particularly in low- and middle-income countries. These findings underscore the urgent need for enhanced strategies and capacity building to improve the detection, treatment, and management of diabetes worldwide. Targeted interventions to bolster health-care systems’ capacity to effectively diagnose and manage diabetes could lead to better health outcomes and reduce the burden of this growing disease.

**Funding:**

Bill & Melinda Gates Foundation.

## Introduction

Diabetes is a metabolic disorder in which the body does not produce enough insulin or respond normally to it, causing chronically high blood glucose levels.^1-4^ According to the Global Burden of Diseases, Injuries, and Risk Factors 2021 Study (GBD 2021), diabetes was the eighth leading cause of disability-adjusted life-years (DALYs) in 2021.^5^ In 2021, there were an estimated 529 million people living with diabetes in the world.^5,6^ Over the next three decades, every country is projected to experience an increase in diabetes prevalence, resulting in over 1.3 billion people living with diabetes worldwide in 2050.^6^

For people living with diabetes, lifelong management is necessary to prevent disabling microvascular complications, such as kidney disease, foot ulcers, lower limb amputations, and vision loss, and reduce the risk of developing cardiovascular diseases.^3^ These micro- and macrovascular complications are preventable through management of glycaemia with initiation of lifestyle changes^7^ and treatment with hypoglycaemic medication,^8-10^ and management of hypertension and hypercholesterolaemia.^11^ Timely diagnosis leading to early initiation of treatment is critical in reducing the risk of developing complications.^12^ Glycaemic management and monitoring for microvascular complications among people with diabetes are high-priority interventions to reduce the probability of premature death in populations by 50%, according to the 2024 *Lancet* Commission on Investing in Health.^13^

The activities of diagnosis, treatment, and management are commonly analysed together as the “cascade of care” to assess health-care system performance for people with a given disease. While historically targeted towards infectious diseases,^14,15^ the cascade of care approach has become more common in assessing coverage for those with non-communicable diseases such as hypertension and diabetes.^16-21^ The cascade of care approach allows identification of where in the continuum of clinical care the greatest strengths or weaknesses occur in a given setting, which can help facilitate the development of targeted interventions to address these gaps in disease management. This makes the care cascade method complementary to the effective coverage approach, which focuses on the success of health interventions in achieving optimal health outcomes at a broader scale and is often used to assess universal health coverage.^22^

The most comprehensive cascade of care studies for diabetes published to date were an analysis of national-level survey data from 28 low- and middle-income countries and a pooled meta-analysis of published literature reporting estimates globally and for select countries.^19,20^ Here, we report on a global systematic analysis of countries’ diabetes care cascade with estimates of the proportion of people with diabetes who are diagnosed with diabetes, proportion of people with diagnosed diabetes who are treated, and proportion of people on diabetes treatment who have optimal glycaemic levels. Estimates are produced globally, regionally, and across 204 countries and territories estimated by the GBD, by age and sex, for the years 2000 through 2023.

## Methods

### Data inputs

The majority of the data for this analysis were identified from the pool of data sources used to estimate diabetes prevalence in the GBD (appendix section 6.2).^5,6^ These data sources were identified in a variety of ways, including performing PubMed systematic reviews, searching the Global Health Data Exchange (GHDx; https://ghdx.healthdata.org/) for updated rounds of known multi-year surveys that meet inclusion criteria, and working with members of the GBD Collaborator Network^23^ with interest in diabetes for access to eligible data sources. These data were supplemented with new data retrieved from a systematic review of PubMed specific to this analysis updated to May 28, 2024 (appendix section 2.1).

Data sources were included in this analysis if they were cross-sectional, representative of the general population, conducted in a location modelled by the GBD, and accessible in microdata form, or were published literature reporting at least one quantity of interest. Included microdata sources had data on age, sex, blood glucose—either fasting plasma glucose (FPG) or glycated hemoglobin (HbA1c)—measurements, and current use of diabetes treatment defined as insulin or other hypoglycaemic medication. Data on self-report of previous diabetes diagnosis were also used in this analysis when available. A total of 266 source-years across 119 countries and territories representing all 21 GBD regions and spanning 1988 through 2023 were included in the analysis. Details on included data sources and the GBD location hierarchy can be found in appendix sections 2 and 7 (tables S19 and S21).

This study complies with the Guidelines for Accurate and Transparent Health Estimates Reporting (GATHER)^24^ and PRISMA guidelines (appendix table S1, appendix figure S1).

### Data processing

Only persons who completed a FPG or HbA1c test and were 15 years or older were included in the analysis. Aggregations of the microdata used sample weighting and were carried out per study, sex, and ten- or 20-year age bin, depending on sample size. According to established diagnostics standards,^2^ the case definition for those with diabetes was FPG greater than or equal to 7 mmol/L, or on treatment; or HbA1c greater than or equal to 6.5% (48 mmol/mol), or on treatment. Five sets of estimates were modelled as intermediate results for the cascade of care quantities of interest. First, the undiagnosed proportion was calculated as those who do not self-report “yes” to having ever been diagnosed with diabetes, among those with diabetes. Second, the diagnosed but untreated proportion was calculated as those who self-report “yes” to having ever been diagnosed with diabetes and do not self-report current diabetes treatment use, among those with diabetes. Third, the treated proportion was calculated as those who self-report “yes” to current diabetes treatment use irrespective of glycaemic levels, among those with diabetes. Fourth, the treated with suboptimal glycaemic levels proportion was calculated as those who self-report “yes” to current diabetes treatment use who have a FPG greater than or equal to 7.2 mmol/L^25^—among those with diabetes. Finally, the treated with optimal glycaemic levels proportion was calculated as those who self-report “yes” to current diabetes treatment use who have a FPG less than 7.2 mmol/L, among those with diabetes. More details on microdata aggregation can be found in appendix section 3.1.

Where tabulated data reported the proportions aggregated for both sexes, these data were disaggregated into sex-specific data by applying a sex ratio generated by the meta-regression—Bayesian, regularised, trimmed (MR-BRT) tool^26^ based on available sex-specific data for a given analysis component. Where tabulated data reported the proportions aggregated for age groups spanning more than 25 years, these data were disaggregated into five-year age groups using the age pattern estimated by DisMod-MR 2.1,^5^ a hierarchical Bayesian meta-regression modelling tool, from a model containing the subset of data for a given analysis component with age range less than 25 years and sample size greater than 30 persons (appendix section 3.2).

Finally, where microdata sources only reported HbA1c rather than FPG, or where tabulated data in published literature reported the proportions using different thresholds for glycaemic management, we adjusted the values to reflect the standards defined above. We used the MR-BRT tool^26^ to produce coefficients to adjust each of these alternative estimates of suboptimal and optimal glycaemic level proportions to reflect values estimated to have been present if the reference definition had been used (appendix section 3.3).

### Modelling and post-processing

Five separate models for the undiagnosed proportion, the untreated diagnosed proportion, the diagnosed and treated proportion, the treated with suboptimal glycaemic levels proportion, and the treated with optimal glycaemic levels proportion were run using DisMod-MR 2.1.^5^ In each model, mean body-mass index (BMI) and the Healthcare Access and Quality Index (HAQ Index), which combines 32 causes of amenable mortality to assess health-care access and quality in a comparable manner across countries,^27^ were used as location-level covariates to inform proportions for locations with missing data along with priors from locations with data higher in the GBD geographical hierarchy (appendix table S21). Further details on the model parameters and coefficients can be found in appendix section 4.1.

After modelling, estimates from the undiagnosed proportion and the untreated diagnosed proportion models were scaled by age group, sex, location, and year to sum to the untreated proportion. Similarly, estimates from the treated with suboptimal glycaemic levels proportion and the treated with optimal glycaemic levels proportion models were scaled to sum to the treated proportion. Thus, the scaled proportions summed to 100% of people with diabetes. After scaling, estimates for the diagnosed proportion among those with diabetes, the treated proportion among those with diagnosed diabetes, and the optimal glycaemic levels proportion among those with treated diabetes were calculated by multiplying each scaled proportion by the corresponding population with diabetes reported by GBD 2023 (unpublished data; GBD 2023 Disease and Injury and Risk Factor Collaborators) to calculate counts of each quantity, and redividing by the desired denominator. Estimates for all age groups over age 15 and age groups 15–39 years, 40–64 years, and 65 years and older were calculated similarly. For more details on post-processing, please see appendix section 4.2. For more details on GBD 2023 estimation of diabetes cases, please see appendix sections 6 and 8.

### Uncertainty and presentation of results

We sampled 250 random draws from each age-, sex-, location-, and year-specific parameter distribution from DisMod-MR. All post-processing steps were conducted at the draw level. 95% uncertainty intervals (UI) for estimates were calculated by taking the 2.5th and 97.5th percentile values across the 250 draws.

All count data reported are presented to three significant figures, while rates and percentages are presented to one decimal place.

### Sensitivity analysis

A sensitivity analysis was conducted using a FPG value of 8.6 mmol/L as the threshold for optimal glycaemic levels. We determined this to be the best estimate in available cross-sectional survey data of a FPG value most similar to a HbA1c value of 8%, the threshold used by the WHO Global Diabetes Compact for their optimal glycaemic levels target,^28^ based on a linear regression of approximately 162,000 individual-level observations with both FPG and HbA1c measurements from 40 studies also included in the main analysis. The data processing and modelling steps were followed as described above (appendix section 5).

### Code availability

All codes used for these analyses are publicly available online. https://github.com/ihmeuw/birds/tree/diabetescascade_lancetdiabetesendocrinology_2025 Analyses were carried out with R (version 4.4.1).

### Role of the funding source

The funder of the study had no role in study design, data collection, data analysis, data interpretation, or the writing of the report.

## Results

### Diagnosis, treatment, and glycaemic management in 2023

In 2023, globally, 55.8% (95% UI 49.3–62.3) of people living with diabetes were diagnosed with diabetes, 91.4% (88.0–94.2) of people diagnosed with diabetes were on treatment, and 41.6% (35.7–48.5) of people on diabetes treatment had optimal glycaemic levels (table 1). These proportions yield 313 million (273–358) people with diagnosed diabetes, 286 million (241–336) people on diabetes treatment, and 119 million (95.2–145) people with diabetes maintaining optimal glycaemic levels worldwide (appendix table S12). An estimated 248 million (207–289) people had undiagnosed diabetes (appendix table S13). Globally, only 21.2% (17.4–25.6) of all people with diabetes had optimal glycaemic levels (appendix table S14).

**Figure 1:**
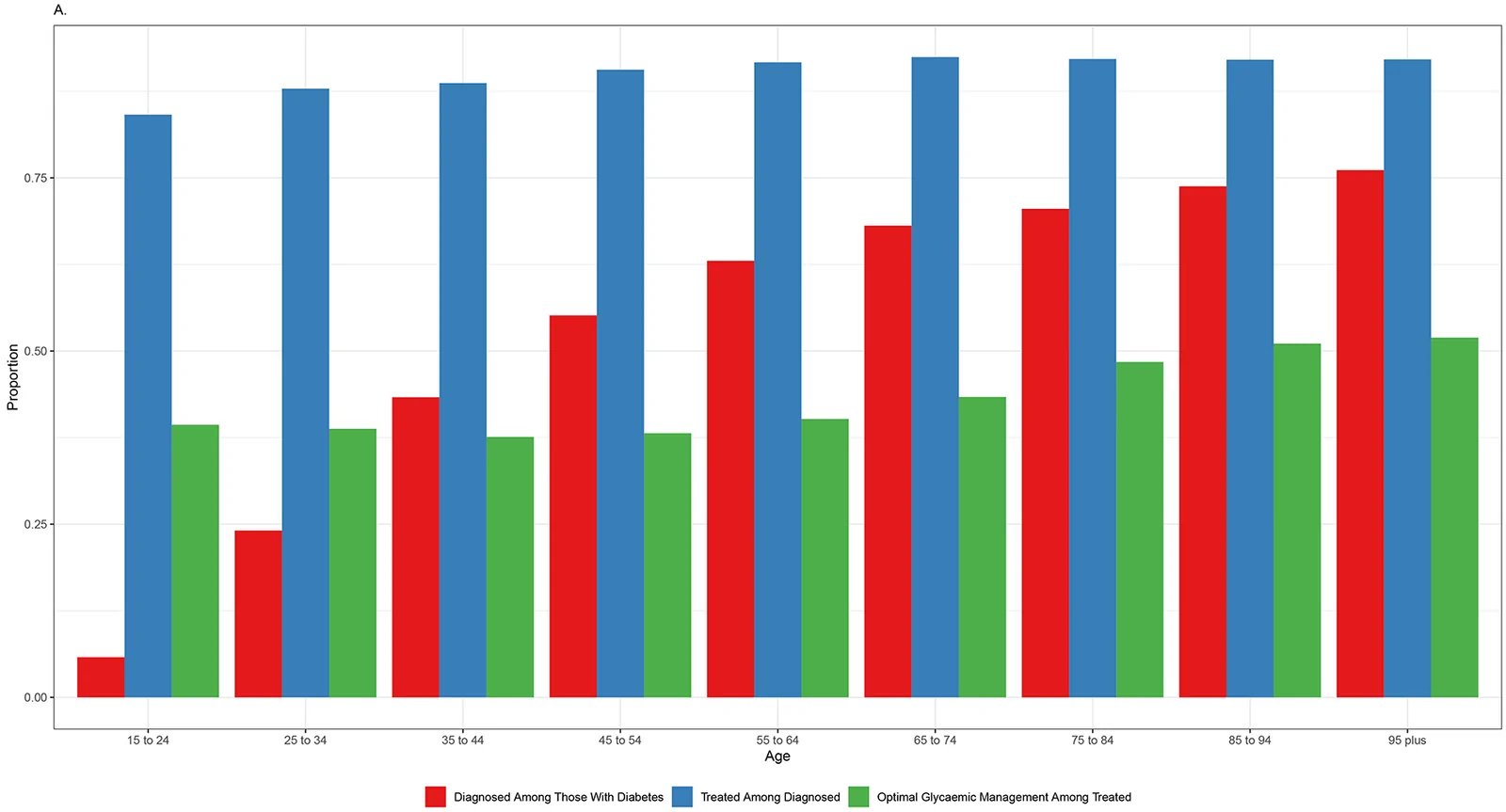

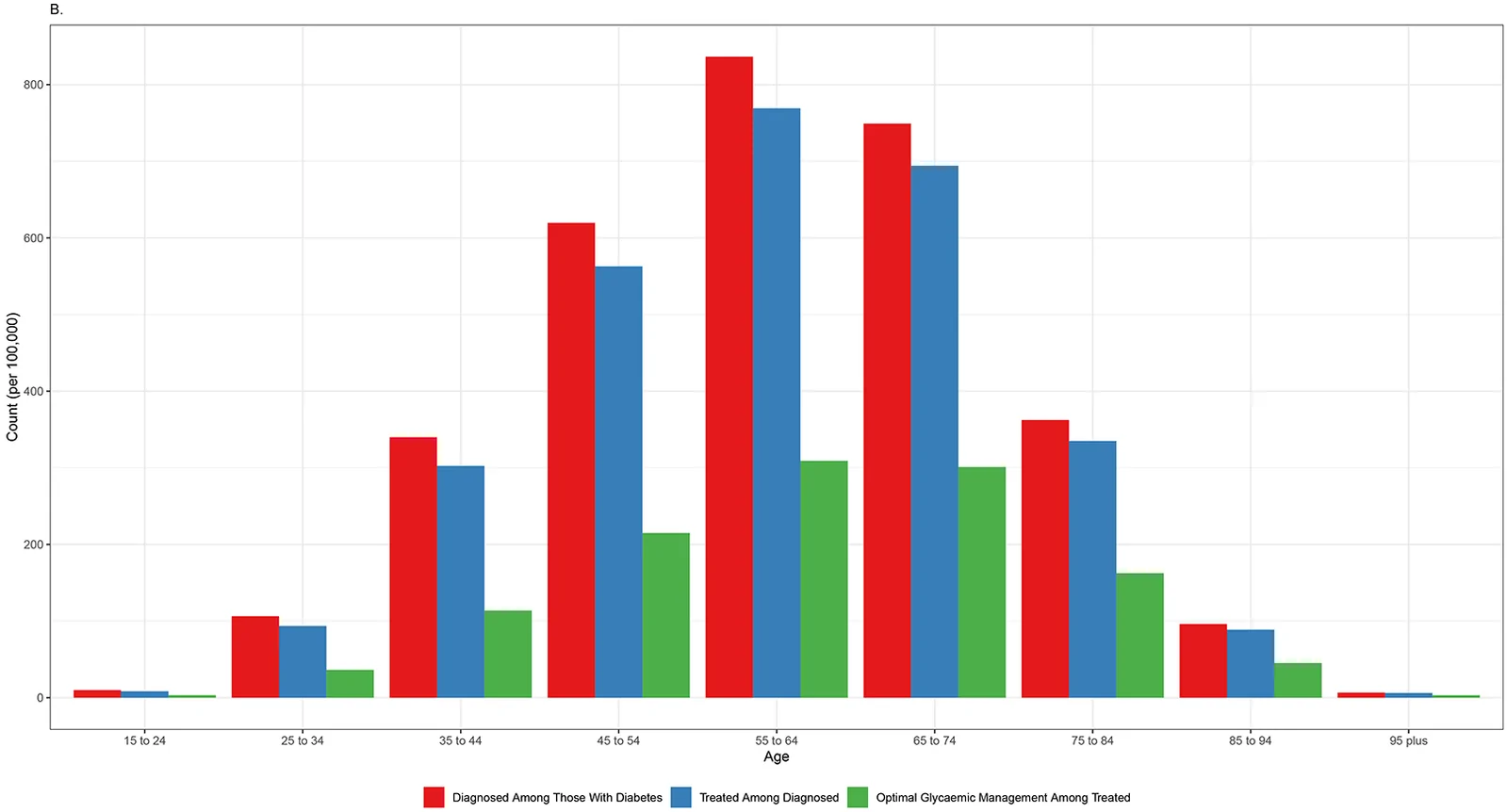
Diabetes cascade of care proportions (A) and counts (B) in 2023 for both sexes over age, globally

While the proportion treated among those with diagnosed diabetes and the proportion with optimal glycaemic levels among those with treated diabetes were similar between males and females globally, males had a greater gap in diagnosis at 51.8% (95% UI 45.6–58.0) compared to 59.8% (53.0–66.6) among females (appendix table S15). While only 26.2% (21.5–32.2) of those aged 15–39 years with diabetes were diagnosed, this proportion increased with older ages to 69.3% (58.6–80.0) among people 65 years and older. Optimal glycaemic levels among those with treated diabetes was also lowest among adults aged 15–39 years at 38.2% (30.5–48.6) and increased with older ages to 45.6% (36.7–54.3) among people 65 years and older. Treatment among those with diagnosed diabetes was high for all age groups (figure 1A). With these gaps in diagnosis among 15–39-year-olds, 71.6 million (62.1–83.5) people in this age group were undiagnosed in 2023 globally, compared to 122 million (89.9–151) adults aged 40–64 years—due to the higher prevalence of diabetes in middle age—and 53.9 million (33.9–72.6) adults aged 65 years and older (figure 1B).

#### Geographical differences in diabetes diagnosis in 2023

**Figure 2:**
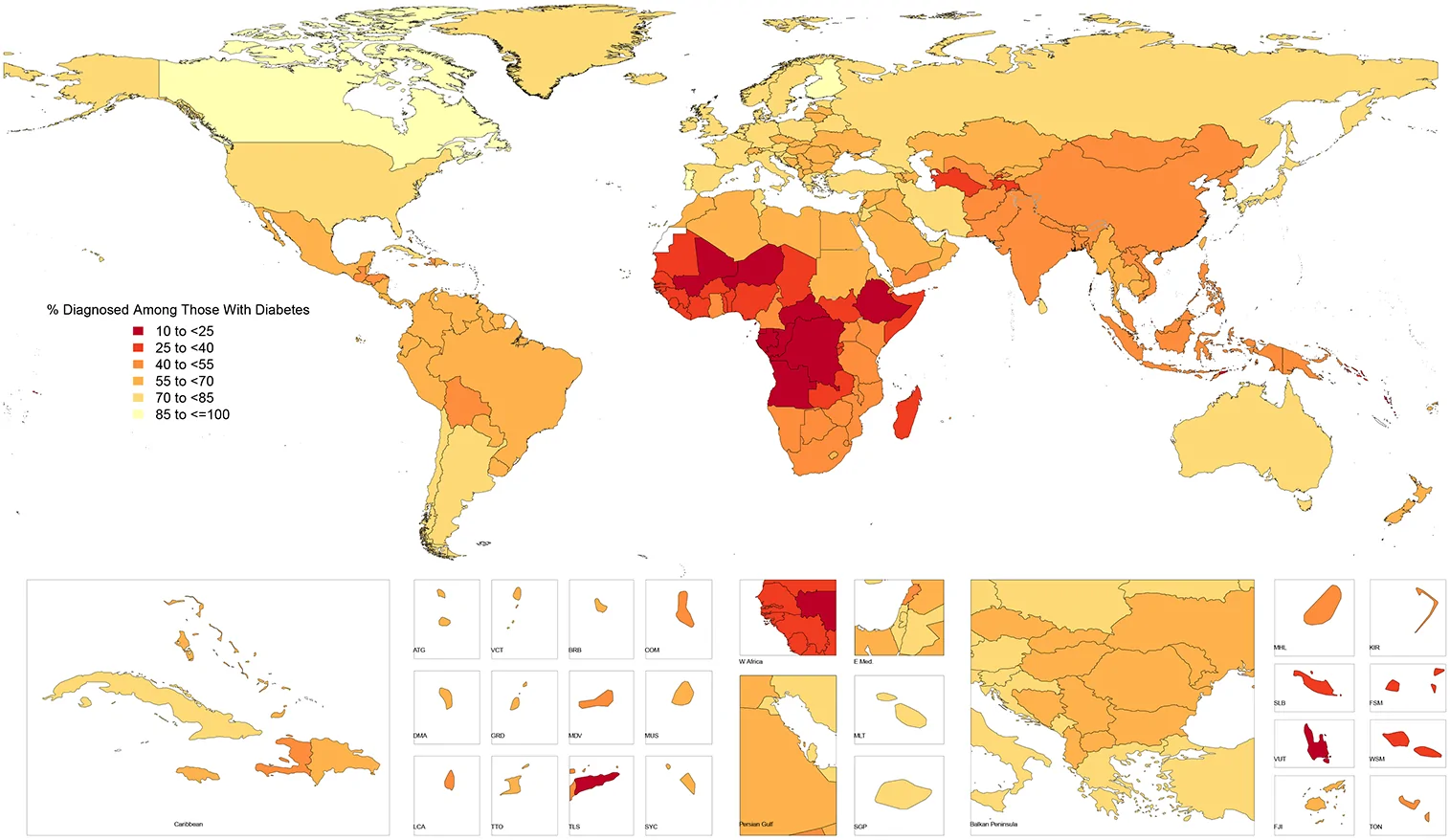

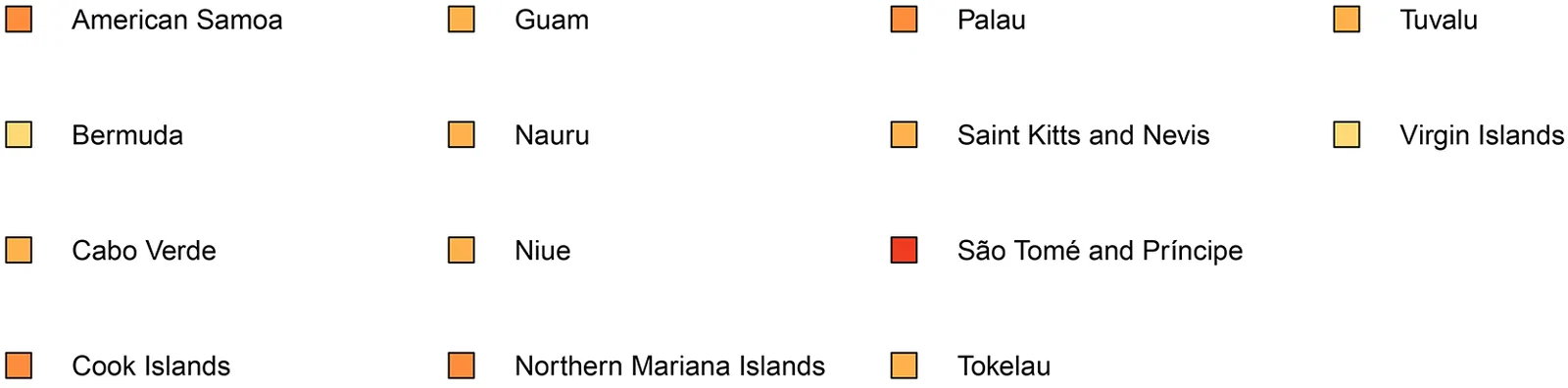

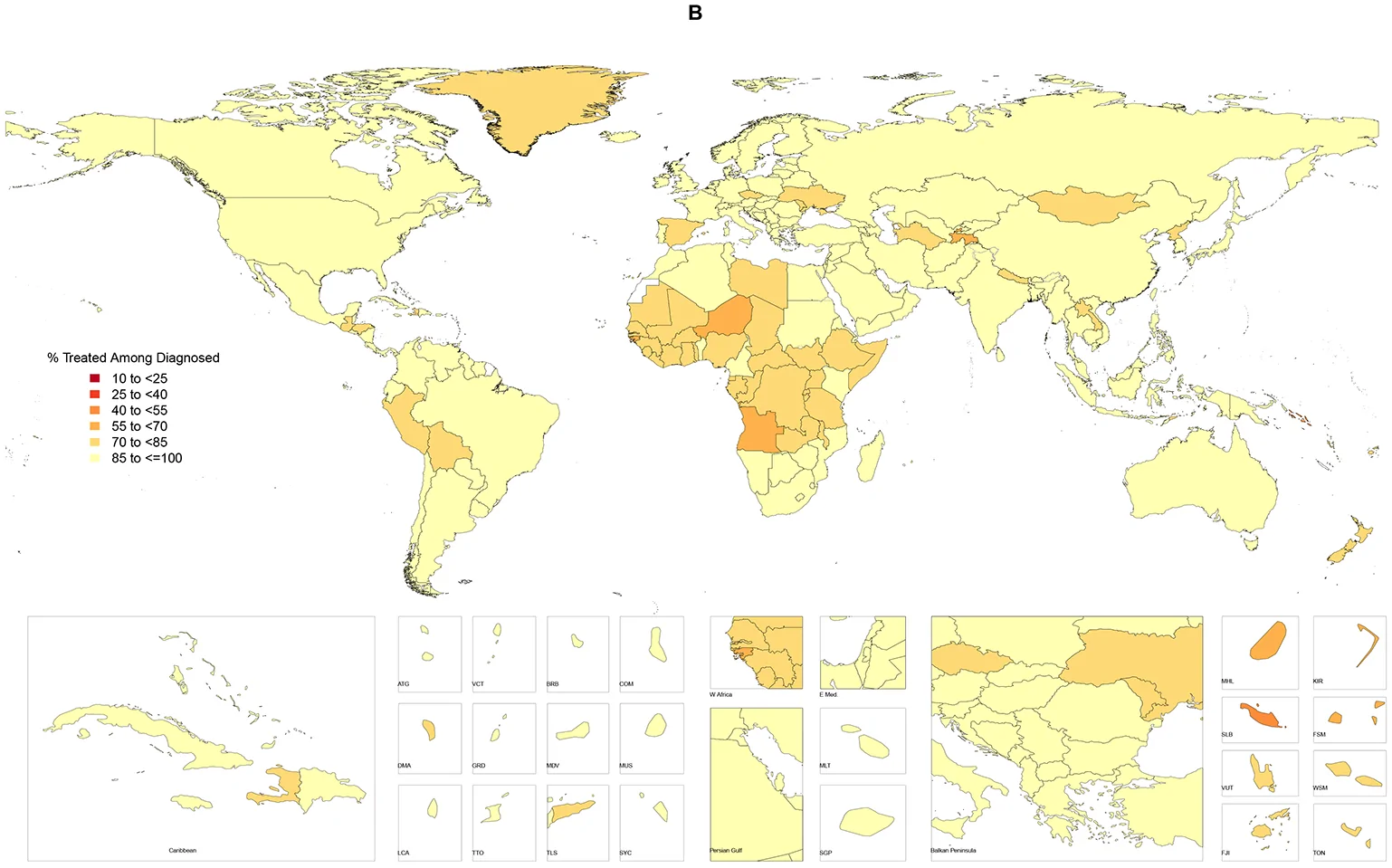

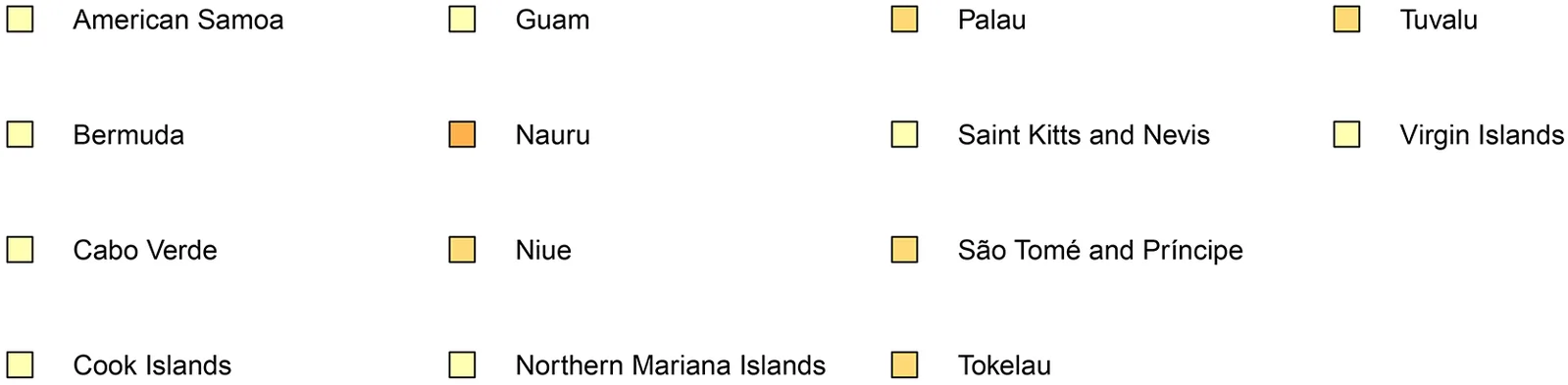

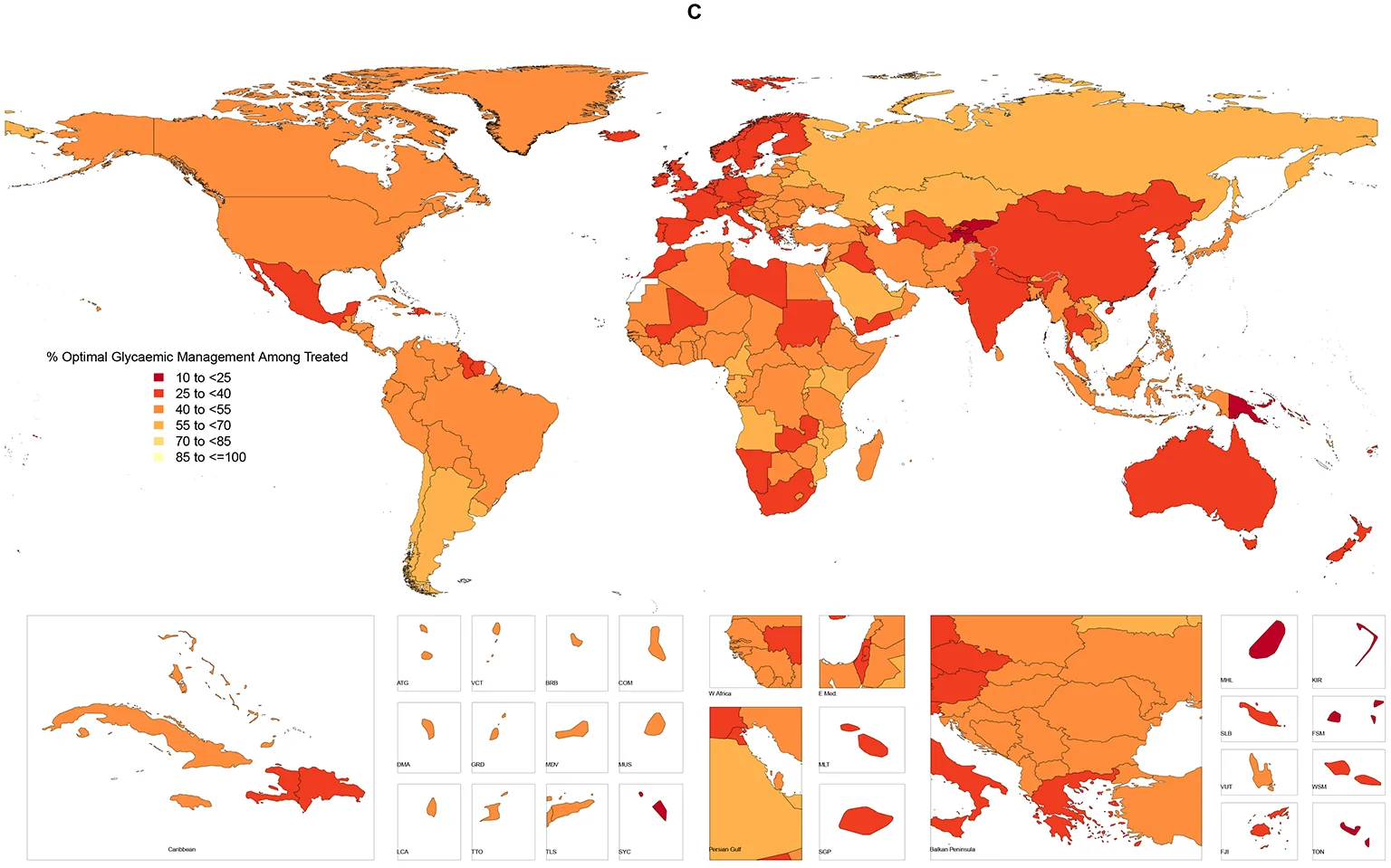

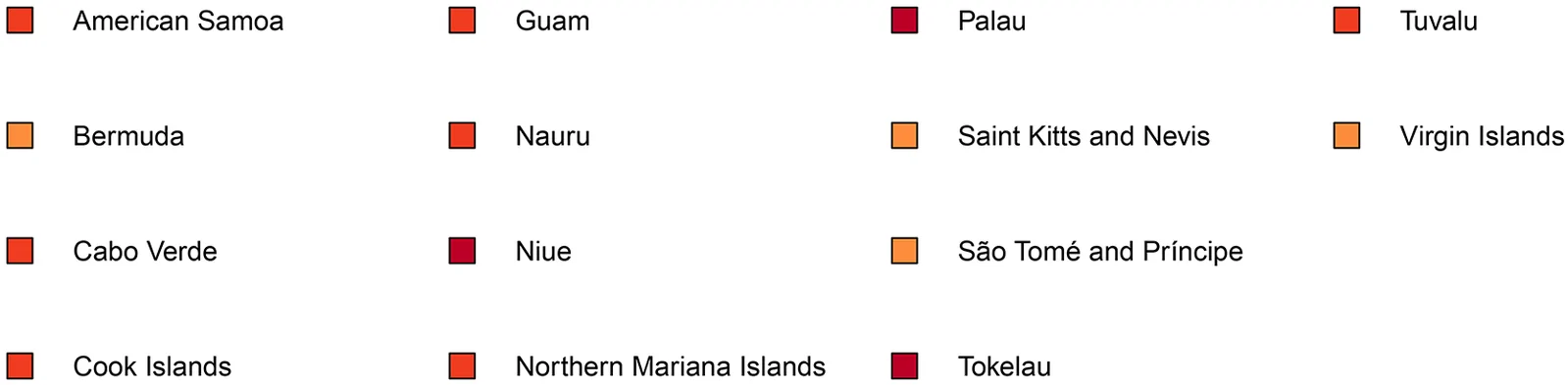
Proportion (A) diagnosed among people living with diabetes, (B) proportion treated among people with diagnosed diabetes, and (C) proportion with optimal glycaemic management among people with treated diabetes, both sexes and ages 15 years and older in 2023

The regions with the highest diagnosed proportions among people with diabetes were high-income North America (82.9% [95% UI 75.5–89.3]), southern Latin America (79.9% [74.9–85.0]), and western Europe (77.5% [70.6–83.9]) (table 1). The central sub-Saharan Africa region had the largest gaps in diagnosis, with only 16.3% (14.0–18.9) diagnosed; all six countries in this region had less than 20% diagnosed. Less than half of the people with diabetes were diagnosed in 64 of 204 (31.4%) countries and territories, with the lowest proportion diagnosed in Niger at 10.7% (8.5–12.9) (table 1, figure 2A).

#### Geographical differences in diabetes treatment in 2023

Among those diagnosed with diabetes, the proportion on treatment varied from 96.5% (95% UI 94.6–98.0) in the high-income Asia Pacific region to 69.1% (62.5–75.3) in the central sub-Saharan Africa region (table 1). Over 90% of people with diagnosed diabetes were treated in 91 out of 204 (44.6%) countries and territories, led by Saudi Arabia at 98.4% (97.3–99.3). Of the nine countries and territories with less than 70% treated, five (55.6%) locations were in Oceania, two (22.2%) locations were in western sub-Saharan Africa, one (11.1%) location was in central sub-Saharan Africa, and one (11.1%) location was in central Asia (table 1, figure 2B).

#### Geographical differences in glycaemic management in 2023

Among people with treated diabetes, the proportion with optimal glycaemic levels varied regionally from 62.6% (95% UI 57.0–67.6) in southern Latin America to 24.4% (19.7–30.1) in Oceania (table 1). 14 out of the 20 (70.0%) countries and territories with less than 30% of treated people with optimal glycaemic levels were in Oceania, with the lowest proportion in Tokelau at 17.0% (13.6–21.5). Among people with treated diabetes, over half had optimal glycaemic levels in 46 out of 204 (22.5%) countries and territories, led by Kazakhstan at 68.3% (62.0–74.1) (table 1, figure 2C). Among all people with diabetes, over 30% had optimal glycaemic levels in 29 out of 204 (14.2%) countries and territories, led by Argentina at 46.9% (41.1–52.3) (appendix figure S16, table S14). A sensitivity analysis applying an optimal glycaemic threshold of FPG 8.6 mmol/L increased the proportion with optimal glycaemic levels among those with treated diabetes to 59.2% (52.3–64.8) globally, spanning 81.2% (75.7–85.3) in central sub-Saharan Africa to 36.7% (29.3–43.3) in Oceania (appendix table S16).

### Change in diagnosis, treatment, and glycaemic management from 2000 to 2023

Globally in 2000, 47.5% (95% UI 42.1–53.1) of people living with diabetes had a diagnosis, 84.2% (79.3–88.4) of people diagnosed with diabetes were on treatment, and 40.3% (34.5–46.8) of people on diabetes treatment had optimal glycaemic levels (appendix table S17). The largest absolute increase across the analysis components between 2000 and 2023 was in diagnosis, with an 8.3 (6.6–10.0) percentage point increase, followed by treatment with a 7.2 (5.7–8.8) percentage point increase, and finally optimal glycaemic management with a 1.3 (0.8–1.8) percentage point increase (table 1). The global number of people with undiagnosed diabetes increased by 73.1% (61.9–83.3), from 143 million (124–163) in 2000 to 248 million (207–289) in 2023 due to higher diabetes prevalence coupled with increased population and ageing (appendix tables S12 and S17). Between 2000 and 2023, among adults aged 15–39 years, diagnosis increased by 4.6 (2.9–6.4) percentage points, and optimal glycaemic levels among those with treated diabetes increased by 4.0 (3.2–4.7) percentage points (appendix figure S17).

Among regions, the percentage point change in diagnosis from 2000 to 2023 ranged from 13.1 (95% UI 10.9–15.3) in central Latin America to 0.5 (–0.6 to 1.8) in southern sub-Saharan Africa; the percentage point change in treatment among those with diagnosed diabetes spanned 17.2 (14.2–19.9) in east Asia to −1.1 (−1.9 to −0.4) in southern sub-Saharan Africa; and the percentage point change in optimal glycaemic levels among those with treated diabetes spanned 7.3 (6.1–8.2) in eastern Europe to −1.9 (−2.6 to −1.1) in southern sub-Saharan Africa (table 1, appendix figure S18). Among 204 countries and territories, 191 (93.6%) had increases in the proportion of people with diabetes who are diagnosed, led by the Republic of Korea with a 20.7 (18.0–23.1) percentage point increase. 185 (90.7%) locations had increases in the proportion of people with diagnosed diabetes who are treated, and 183 (89.7%) countries and territories had increases in the proportion of people on diabetes treatment with optimal glycaemic levels (table 1).

## Discussion

Globally in 2023, health systems performed best in providing treatment for 91.4% of people with diagnosed diabetes, followed by diagnosing 55.8% of people living with diabetes, while the largest gap was in managing optimal glycaemic levels among those with treated diabetes at 41.6%. Globally, diagnosis was lowest between adults aged 15–39 years at 26.2%, although the number of people with undiagnosed diabetes was highest among 40–64-year-olds at 122 million. Between 2000 and 2023, diagnosis increased in all regions, while increases in treatment occurred in 19 out of 21 (90.5%) regions and increases in glycaemic management occurred in 18 out of 21 (85.7%) regions. In 2022, the WHO Global Diabetes Compact set a target to have 80% of people with diabetes clinically diagnosed by 2030.^28^ Given the 8.3 percentage point increase in diagnosis between 2000 and 2023, more rapid progress is needed in screening diabetes to achieve this goal by 2030, in conjunction with efforts to curb the rapid increase in diabetes cases.

Among all regions and nearly all countries and territories, the proportion of people with diabetes who were diagnosed increased between 2000 and 2023, suggesting that detection of diabetes improved over time. Guidelines for screening to detect pre-diabetes and diabetes have been set by organisations such as the American Diabetes Association (ADA)^2^ and the World Health Organization (WHO)^4^ that often influence national-level guidelines for diabetes screening and management.^29^ Greater international recognition of the serious disease burden attributable to diabetes has increased awareness of diabetes risk factors and diagnosis.^30^ In the Republic of Korea, for example, which we estimated to have had the greatest increase in diabetes diagnosis between 2000 and 2023, a comprehensive national health promotion plan that involved mass diabetes education campaigns and early diabetes detection in primary care was found to be associated with reduced underdiagnosis.^31^ However, modest increases in diabetes diagnosis in some locations suggests that challenges remain in translating international screening guidelines and advocacy into sustainable change. For example, in some low- and middle-income countries (LMICs), diabetes health services continue to be inaccessible to certain populations due to geographical and socioeconomic constraints.^32^ In many sub-Saharan African countries, diabetes diagnostic capacity in primary health centres is under-resourced due to a scarcity of testing strips, glucose meters, and laboratories.^33^ Due to these resource constraints, diabetes guidelines for screening and management—which are based on evidence from high-income settings—may require careful adaptation and contextualisation for low-resource health-care settings in LMICs.^34,35^

The largest gaps in diabetes diagnosis were among younger adults ages 15–39 years. Although the greatest number of individuals with undiagnosed diabetes was concentrated between 40 and 64 years, the development of type 2 diabetes in people younger than 40 years is associated with a greater risk of complications compared to diabetes onset later in life, in part due to longer diabetes duration and more rapid β-cell decline.^36,37^ Young adults continue to face challenges in accessing health-care services including cost barriers, health insurance illiteracy and gaps in coverage, a lack of agency in health-care decision-making, and a lack of trust in health systems.^38-40^ In 2022, the ADA reduced their recommended age at which to initiate screening, regardless of weight and risk factors, from 45 years to 35 years, and emphasised the importance of screening those with diabetes risk factors regardless of age.^41^ Further efforts to reduce underdiagnosis of diabetes among younger adults such as contextualising screening standards to capture those at the highest risk of complications is needed, although in limited-resource settings, the cost of more expansive screening programmes will need to be balanced with the capacity of the health system to expand treatment and effective glycaemic management.

The increase in the proportion of people with treated diabetes with optimal glycaemic levels between 2000 and 2023 varied across countries and was substantially smaller than the increases in diagnosis and treatment. This suggests that while treatment coverage has increased over the past two decades, glycaemic management has largely not improved. New glucose-lowering medications such as SGLT2 inhibitors and GLP-1 receptor agonists have become available, with even more treatment options under development.^42,43^ Additionally, there are now more innovative insulin delivery methods, the most recent being continuous glucose monitoring devices.^44^ However, substantial price reductions are necessary to make these advances in treatment affordable and cost-effective for LMICs.^45^ Over the past few decades, many countries, including the USA through the Affordable Care Act^46^ and Brazil through its federal program “Health Has No Price” (*Saúde Não Tem Preço*—SNTP),^47^ have expanded access to less affordable diabetes medications and management tools. Substantial progress has been made in sub-Saharan Africa, where many countries now include diabetes drugs on their lists of essential medicines—making these medicines readily accessible and free—or subsidise insulin at tax-free prices.^33^ However, the lack of sustained improvement in optimal glycaemic management among those with treated diabetes signals that challenges remain in delivering high-quality diabetes care. While approximately 80% of 160 WHO member states report having national guidelines for diabetes management,^48^ many LMIC guidelines are narrow in scope and lack socioeconomic contextualisation, making implementation difficult.^29,35^ Other challenges include shortages of health-care personnel who are adequately trained on diabetes care and high out-of-pocket costs for diabetes medicines such as insulin in many LMICs, which is often cited as a key reason for poor treatment adherence.^32,33,49^ Treatment regimens can be complex, requiring various prescribed medications—often for multiple chronic comorbid conditions—and high dosing frequencies, which are burdensome to an individual and create additional adherence challenges.^50,51^ Furthermore, the prevalence of overweight and obesity has rapidly increased over the past few decades,^52,53^ and if current trends continue, it is estimated that nearly two in three adults will be overweight or obese by 2050, which may further hinder effective glycaemic management.

Our global findings are similar to previous publications on components of the diabetes care cascade. The International Diabetes Federation (IDF) estimated that 44.7% of adults aged 20–79 years with diabetes were undiagnosed in 2021 globally, a decrease of 5.1 percentage points since 2011 estimates,^54^ which is comparable to our undiagnosed estimates of 44.2% among those 15 years and older in 2023, an 8.3 percentage point decrease since 2000. A pooled global analysis of published literature reported that after 2010, 41% of people living with diabetes were undiagnosed, 47% were treated, and 22% had optimal glycaemic levels,^20^ which is similar to our estimates that 44.2% of people with diabetes were undiagnosed, 51.0% were treated, and 21.2% had optimal glycaemic levels in 2023. The NCD Risk Factor Collaboration (NCD-RisC) estimated that approximately 41% of those 30 years and older with diabetes were treated in 2022, which is lower than our estimate that 51.0% of those 15 years and older with diabetes were treated in 2023.^55^ Some of the variation between our findings and previously published estimates can be attributed to differences in diabetes case definitions—in this analysis, we define diabetes using only FPG or only HbA1c, whereas other studies may also capture diabetes cases using oral glucose tolerance test (OGTT), random blood glucose test, or self-report of previous diabetes diagnosis alone. Furthermore, global estimates of diabetes care cascade components may vary due to differences in country-level estimates of diabetes cases, which are used to aggregate country-level cascade components to global-level estimates.

There are limitations in this analysis that should be considered. First, in cases where no country-level data could be included in the models, estimates were informed by data elsewhere in the region and by the mean body-mass index (BMI) and the Healthcare Access and Quality Index (HAQ Index) covariates. These covariates also helped to inform changes in estimate trends over time in the absence of data covering multiple time points for a location. Second, the population-based epidemiological studies used in this analysis only collected single glucose measurements per participant, which were used to determine diabetes status. However, clinical diagnostic criteria for diabetes typically require more than one abnormal glucose test result,^4^ as relying on a single measurement likely overestimates diabetes prevalence.^56-58^ If a single FPG value greater than or equal to 7 mmol/L has 70% repeatability compared to two values,^56^ our estimates of the proportion of people with diabetes with a diagnosis may be underestimated by up to ten percentage points. Third, data on self-report of current use of insulin or other hypoglycaemic medication were used to determine treatment status. While studies have reported high validity of self-report of diabetes medication use compared to pharmacy data,^59^ these data do not reflect true treatment adherence.^60^ We did not take into account lifestyle modifications such as improving diet and increasing exercise that may lower glucose levels in this analysis; therefore, people with optimal glucose levels within the diagnostic threshold for diabetes while not on pharmacological treatment are considered untreated, which may underestimate the proportion achieving optimal glycaemic levels. Antihypertensive and cholesterol-lowering medications were also not considered in this analysis as they do not directly impact glycaemic levels, although they are often recommended to reduce risk of cardiovascular morbidity and mortality in comprehensive clinical diabetes care.^11,28,61^ Estimation of cardiovascular medication coverage and impact among people with diabetes in future studies would allow us to quantitatively assess the effectiveness of comprehensive diabetes care in reducing overall diabetes morbidity and mortality. Fourth, we decided to use a definition based on a FPG value rather than a HbA1c value because the majority of our data inputs only collected FPG among participants, as HbA1c is more costly.^62^ However, the use of FPG as an indicator of glycaemic management may be subject to misclassification, as FPG can vary considerably from day to day.^56^ Applying a threshold of FPG 8.6 mmol/L as a sensitivity analysis increased our estimated proportion with optimal glycaemic levels among those with treated diabetes to 59.2%, compared to 41.6% below FPG 7.2 mmol/L. In clinical practice, individualised approaches to glycaemic targets are typically taken as they often vary depending on life expectancy, complications, comorbidities, and risk of hypoglycaemia.^2,61^ Finally, although we report estimates for 2023, scarce data availability during the COVID-19 pandemic limits our ability to consider the pandemic’s impact on diabetes diagnosis, treatment, and glycaemic management. Therefore, our reporting of estimates in 2023 may not reflect the lasting effect of the pandemic on the diabetes care cascade, including delays in medical care—potentially delaying diagnosis and interrupting treatment—and disruptions to routine lifestyle behaviours.^63,64^

Diabetes is a growing threat to population health that requires timely detection, treatment, and adequate management of glycaemic levels to reduce the risk of complications and premature death. Understanding these dimensions and how the components of the cascade of diabetes care—including diagnosis, treatment, and glycaemic management—vary both across countries and within populations is critical in identifying gaps in delivering diabetes care at the population level. Country health systems must continue to prioritise efforts to build greater diagnostic capacity and improve health-care service delivery to reduce complications among those living with diabetes.

## Supplementary Material

Appendix 1: Supplementary methods and results to “Global, regional, and national cascades of diabetes care, 2000–2023: diagnosis, treatment, and glycaemic management”

## Figures and Tables

**Table 1:** Diagnosed proportion among people living with diabetes, treated proportion among people with diagnosed diabetes, and optimal glycaemic management proportion among people with treated diabetes in 2023, and corresponding absolute change between 2000 and 2023, for both sexes and ages 15 and older globally, and by Global Burden of Disease super-regions, regions, and countries and territories

*95% confidence* *intervals in parentheses*						
Location	Diagnosed, 2023 (%)	Change in Diagnosed, 2000- 2023 (%)	Treated, 2023 (%)	Change in Treated, 2000- 2023 (%)	Optimal Glycaemic Management, 2023 (%)	Change in Optimal Glycaemic Management, 2000-2023 (%)
**Global**	**55·8** **(49·3–62·3)**	**8·3** **(6·6–10·0)**	**91·4** **(88·0–94·2)**	**7·2** **(5·7–8·8)**	**41·6** **(35·7–48·5)**	**1·3** **(0·8–1·8)**
**Central Europe, eastern Europe, and central Asia**	**67·7** **(59·6–75·7)**	**5·3** **(3·7–6·8)**	**92·1** **(87·6–95·6)**	**2·8** **(1·6–4·0)**	**50·4** **(43·6–57·6)**	**3·7** **(3·0–4·4)**
Central Asia	54·1 (48·2–60·6)	7·2 (5·1–9·3)	88·4 (83·0–92·7)	8·0 (5·4–10·8)	42·2 (35·7–49·2)	0·9 (−0·6–2·6)
Armenia	63·7 (56·1–71·5)	8·9 (5·3–12·5)	91·3 (85·8–95·2)	7·7 (4·1–11·5)	40·8 (34·5–47·7)	2·5 (0·4–4·6)
Azerbaijan	65·6 (58·8–72·0)	11·4 (6·8–15·4)	93·9 (90·6–96·5)	5·9 (2·9–9·4)	29·1 (23·6–35·0)	2·0 (−0·2–4·6)
Georgia	73·3 (67·2–79·2)	5·9 (1·6–10·8)	90·9 (86·4–94·4)	3·9 (0·7–7·9)	45·0 (39·0–51·7)	1·3 (−1·0–3·9)
Kazakhstan	59·3 (52·3–67·2)	12·1 (7·7–16·3)	91·6 (86·3–95·4)	13·2 (8·8–17·5)	68·3 (62·0–74·1)	1·8 (−0·1–3·5)
Kyrgyzstan	56·5 (50·0–64·0)	11·3 (7·5–15·7)	93·2 (89·6–96·0)	7·5 (4·8–11·0)	22·0 (17·2–27·4)	1·3 (−0·3–3·0)
Mongolia	44·9 (39·6–50·7)	6·6 (4·0–9·5)	78·2 (72·4–84·3)	13·0 (9·8–16·6)	39·1 (33·4–45·5)	1·1 (−1·0–3·4)
Tajikistan	38·7 (34·1–43·5)	2·4 (0·3–4·6)	64·5 (55·9–72·5)	9·4 (6·0–13·4)	22·9 (16·7–31·3)	0·7 (−1·6–3·2)
Turkmenistan	28·8 (24·9–33·0)	2·6 (1·2–4·5)	81·7 (75·6–86·7)	7·5 (4·6–10·5)	25·5 (20·0–31·6)	1·1 (−0·6–3·0)
Uzbekistan	49·9 (43·2–58·5)	8·1 (5·6–11·9)	87·0 (81·1–92·2)	7·0 (5·1–9·3)	35·7 (29·7–42·9)	1·8 (−0·3–4·0)
Central Europe	71·8 (63·0–80·5)	2·7 (0·9–4·5)	91·9 (86·6–95·6)	1·5 (0·7–2·5)	47·7 (40·6–55·3)	0·8 (−0·3–1·9)
Albania	67·8 (58·2–78·8)	7·1 (3·5–11·0)	90·5 (84·6–95·2)	4·2 (2·2–6·0)	42·5 (35·6–50·8)	2·6 (0·7–4·8)
Bosnia and Herzegovina	66·6 (57·2–77·6)	4·5 (1·3–7·7)	88·7 (81·9–94·2)	3·9 (1·8–6·4)	42·5 (35·4–50·5)	2·9 (0·9–4·8)
Bulgaria	64·1 (55·2–74·3)	1·4 (−0·9–3·6)	85·4 (77·4–91·7)	1·7 (0·1–3·3)	42·9 (35·5–50·9)	2·3 (0·7–3·9)
Croatia	73·9 (63·3–84·2)	4·3 (0·8–7·9)	93·4 (88·1–96·9)	2·3 (0·6–4·3)	44·1 (37·1–52·0)	3·3 (1·5–5·2)
Czechia	62·0 (53·5–72·0)	4·5 (0·6–10·7)	83·6 (75·3–90·2)	5·1 (1·7–8·9)	33·5 (27·4–40·9)	4·5 (1·2–7·4)
Hungary	68·4 (59·4–78·4)	2·1 (−0·8–4·6)	89·3 (83·1–94·1)	0·9 (−0·5–2·5)	44·0 (37·3–51·9)	2·8 (1·3–4·3)
Montenegro	70·2 (60·3–80·1)	3·6 (0·4–6·9)	91·1 (85·1–95·6)	1·7 (−0·2–3·2)	43·8 (37·0–51·4)	3·0 (1·5–4·8)
North Macedonia	65·8 (57·1–76·2)	4·9 (1·8–8·1)	87·5 (80·3–93·2)	4·5 (2·6–6·9)	43·0 (35·7–50·3)	2·7 (0·8–4·7)
Poland	78·6 (68·7–87·0)	3·1 (0·3–6·2)	95·9 (92·5–98·2)	0·9 (0·2–2·0)	53·1 (45·9–60·7)	1·3 (−0·1–2·7)
Romania	65·8 (56·5–75·9)	4·4 (2·2–6·6)	87·9 (81·1–93·3)	3·6 (1·9–5·5)	42·7 (36·0–50·7)	3·0 (1·2–4·6)
Serbia	65·6 (56·6–76·0)	4·1 (1·4–7·0)	86·6 (79·5–92·8)	4·7 (2·4–7·1)	43·3 (36·3–51·1)	3·1 (1·5–4·5)
Slovakia	67·8 (58·2–77·9)	4·0 (1·4–6·8)	89·8 (83·7–94·7)	3·2 (1·3–5·0)	43·0 (36·2–50·4)	2·4 (0·9–3·9)
Slovenia	79·0 (69·5–87·3)	6·9 (1·5–11·4)	96·2 (92·9–98·3)	3·0 (0·8–5·4)	44·2 (37·5–52·0)	3·3 (1·9–5·4)
Eastern Europe	69·5 (60·9–77·5)	9·0 (6·6–11·0)	93·3 (89·3–96·3)	3·0 (1·7–4·6)	55·3 (48·5–62·4)	7·3 (6·1–8·2)
Belarus	77·3 (72·7–81·5)	9·3 (3·2–14·2)	88·3 (84·2–91·9)	13·6 (5·9–20·8)	60·3 (54·1–66·8)	0·9 (−2·4–4·2)
Estonia	72·9 (64·0–82·3)	10·0 (5·1–13·5)	94·1 (89·9–97·2)	6·5 (3·5–9·8)	50·5 (43·7–57·9)	2·5 (0·9–4·1)
Latvia	67·0 (58·7–76·6)	5·0 (1·7–8·3)	89·5 (83·9–94·5)	3·4 (1·4–5·5)	50·3 (42·9–58·2)	2·4 (0·5–4·5)
Lithuania	68·8 (59·7–78·4)	7·9 (2·7–15·3)	93·3 (89·0–96·6)	3·1 (0·7–5·6)	40·4 (33·9–48·1)	2·5 (−0·7–6·0)
Moldova	57·9 (52·1–64·2)	3·7 (−0·3–7·1)	79·1 (73·2–84·8)	5·2 (1·0–9·0)	45·7 (39·9–53·1)	1·8 (−0·9–4·5)
Russia	71·5 (62·1–80·8)	10·9 (8·0–13·3)	96·2 (93·5–98·1)	2·4 (1·4–3·6)	56·7 (49·6–63·9)	8·7 (7·4–9·7)
Ukraine	58·4 (50·8–65·9)	0·0 (−2·1–2·0)	82·2 (74·6–88·4)	−4·7 (−6·7–−2·8)	48·0 (41·4–55·5)	1·7 (0·3–3·3)
**High income**	**79·5** **(72·6–85·8)**	**6·3** **(4·9–7·6)**	**91·5** **(86·5–95·2)**	**2·8** **(2·0–3·8)**	**44·9** **(38·8–51·8)**	**3·1** **(2·3–3·9)**
Australasia	70·6 (63·1–78·6)	6·3 (2·2–11·8)	86·1 (79·4–91·7)	5·6 (2·1–9·0)	33·3 (27·2–40·0)	3·9 (1·6–5·9)
Australia	70·7 (63·0–78·9)	6·7 (2·1–13·2)	86·4 (79·6–91·9)	5·5 (1·6–9·6)	33·6 (27·4–40·1)	4·1 (1·2–6·3)
New Zealand	70·0 (62·9–77·4)	4·9 (2·5–7·3)	85·0 (77·8–91·3)	6·3 (4·0–8·6)	32·3 (26·6–39·2)	3·3 (1·7–4·7)
High-income Asia Pacific	75·4 (68·5–81·9)	11·1 (7·7–14·2)	96·5 (94·6–98·0)	4·2 (2·5–6·3)	50·8 (45·3–56·4)	4·5 (2·6–6·3)
Brunei	73·8 (66·9–79·7)	8·4 (3·9–14·7)	90·8 (86·3–94·2)	4·6 (1·2–8·7)	38·2 (31·9–44·7)	2·0 (−1·1–5·0)
Japan	74·7 (65·9–83·4)	8·5 (4·5–12·3)	96·4 (93·9–98·2)	2·4 (1·1–4·1)	51·3 (44·4–58·5)	5·1 (3·3–7·0)
South Korea	77·1 (74·9–79·1)	20·7 (18·0–23·1)	96·7 (95·8–97·4)	14·0 (11·9–16·2)	50·9 (48·3–53·2)	2·3 (−0·4–5·2)
Singapore	74·5 (67·2–82·1)	17·9 (11·9–24·1)	96·1 (93·7–97·9)	7·2 (5·5–8·9)	36·4 (30·5–42·8)	2·2 (−1·0–5·4)
High-income North America	82·9 (75·5–89·3)	4·4 (2·5–6·0)	92·4 (87·2–96·1)	−0·2 (−1·4–0·9)	46·9 (40·6–54·0)	3·0 (1·7–4·3)
Canada	86·0 (79·4–91·8)	2·6 (−1·3–6·6)	96·4 (93·5–98·3)	0·5 (−0·7–1·7)	44·8 (38·1–51·9)	0·3 (−1·4–2·1)
Greenland	72·5 (64·9–80·7)	6·3 (2·3–11·1)	82·7 (74·3–90·4)	9·9 (6·4–14·0)	42·2 (35·2–49·5)	1·0 (−1·6–4·2)
USA	82·8 (75·4–89·2)	4·5 (2·6–6·2)	92·1 (86·7–95·9)	−0·2 (−1·6–0·9)	47·1 (40·7–54·1)	3·2 (1·8–4·5)
Southern Latin America	79·9 (74·9–85·0)	5·5 (2·0–9·6)	91·7 (87·7–94·8)	4·0 (1·6–8·2)	62·6 (57·0–67·6)	1·2 (−1·4–3·7)
Argentina	79·9 (75·0–85·1)	4·2 (−0·7–9·5)	92·0 (88·4–94·9)	2·9 (0·1–7·6)	63·7 (58·3–69·0)	0·9 (−2·1–3·9)
Chile	82·9 (77·6–88·0)	10·6 (7·0–14·2)	91·5 (87·4–94·9)	10·3 (7·1–13·7)	58·3 (52·1–63·6)	5·6 (2·7–8·5)
Uruguay	68·5 (62·0–74·7)	3·0 (−0·1–5·9)	89·0 (84·4–93·0)	3·0 (0·6–5·7)	65·8 (59·7–71·1)	2·2 (0·2–4·3)
Western Europe	77·5 (70·6–83·9)	3·5 (1·8–5·4)	88·0 (82·4–93·0)	3·9 (3·0–4·9)	37·0 (31·1–44·0)	0·8 (0·1–1·5)
Andorra	79·8 (72·5–86·4)	3·2 (−0·2–6·7)	93·4 (89·0–96·6)	1·3 (−0·3–2·9)	35·8 (29·8–42·5)	1·1 (−0·3–2·8)
Austria	78·9 (70·6–86·3)	4·0 (0·7–7·4)	92·0 (87·2–96·1)	3·1 (1·1–5·3)	36·8 (30·4–43·8)	1·3 (−0·3–2·8)
Belgium	77·5 (69·8–85·2)	3·6 (0·6–6·3)	91·0 (85·9–95·2)	3·1 (1·1–5·2)	36·5 (30·6–43·1)	1·1 (0·0–2·5)
Cyprus	77·1 (68·9–84·7)	9·2 (5·7–12·5)	90·5 (85·6–95·0)	12·1 (8·7–16·2)	35·8 (29·7–42·3)	1·0 (−0·3–2·3)
Denmark	77·7 (69·8–85·3)	6·9 (2·9–10·4)	91·1 (85·3–95·3)	7·5 (4·7–11·4)	36·8 (30·3–43·6)	2·0 (0·1–3·8)
Finland	86·8 (80·4–92·1)	3·9 (−0·4–8·0)	95·4 (91·7–97·8)	2·6 (0·0–5·2)	37·3 (31·1–43·9)	2·2 (0·4–4·0)
France	72·3 (64·8–80·0)	8·3 (2·3–15·3)	89·2 (83·8–93·8)	8·6 (4·6–12·8)	36·6 (30·8–43·4)	1·3 (−0·1–2·5)
Germany	77·3 (69·6–84·7)	2·4 (−0·6–4·8)	89·7 (84·5–94·6)	2·4 (0·3–4·5)	37·3 (31·4–44·6)	1·1 (−0·6–2·6)
Greece	75·7 (68·6–82·9)	0·6 (−1·7–3·4)	86·9 (81·0–92·5)	−1·6 (−3·4–0·3)	37·6 (31·6–44·1)	1·6 (0·0–3·1)
Iceland	78·7 (70·3–85·5)	2·7 (−0·4–5·7)	92·5 (88·3–96·1)	1·5 (−0·4–3·3)	36·8 (30·7–43·8)	1·1 (−0·4–2·5)
Ireland	79·1 (71·2–86·1)	8·8 (4·4–12·6)	92·6 (88·3–96·0)	8·3 (5·2–11·9)	36·7 (30·6–43·4)	1·4 (0·0–2·9)
Israel	76·4 (68·7–84·0)	5·3 (2·1–8·7)	89·8 (84·3–94·7)	7·0 (3·9–10·1)	36·8 (30·9–43·4)	0·5 (−1·0–1·9)
Italy	79·5 (71·3–86·7)	2·3 (0·6–3·7)	92·5 (88·0–96·1)	1·2 (0·4–2·0)	36·8 (30·7–43·9)	1·5 (0·7–2·4)
Luxembourg	75·6 (67·8–83·2)	5·9 (1·0–11·6)	90·7 (85·7–95·0)	5·6 (2·7–9·2)	36·0 (30·0–42·4)	0·9 (−0·4–2·1)
Malta	77·3 (69·1–84·9)	4·6 (0·9–8·2)	90·3 (84·8–94·7)	4·4 (1·7–7·9)	37·1 (30·7–43·8)	2·0 (0·3–3·9)
Monaco	78·0 (70·0–85·4)	2·7 (−0·1–5·4)	89·5 (83·9–94·6)	2·3 (0·4–4·7)	38·2 (32·0–45·0)	1·0 (−0·7–2·5)
Netherlands	79·7 (71·1–86·7)	5·1 (1·4–8·9)	92·7 (87·7–96·3)	4·4 (1·9–7·0)	37·0 (30·8–44·0)	1·6 (0·1–3·1)
Norway	78·6 (70·0–86·2)	1·4 (−0·4–3·1)	97·2 (94·9–98·6)	1·0 (0·5–1·6)	37·9 (32·0–44·6)	0·9 (−0·3–2·0)
Portugal	88·4 (82·6–93·2)	4·3 (−1·0–9·4)	95·4 (91·7–97·9)	3·8 (0·7–6·3)	37·3 (30·8–44·2)	2·0 (0·5–3·8)
San Marino	81·1 (73·0–88·3)	2·2 (−0·9–4·9)	93·1 (88·6–96·5)	0·3 (−1·2–1·6)	37·5 (31·6–44·3)	1·1 (−0·4–2·6)
Spain	82·7 (77·4–87·1)	2·4 (−1·0–5·6)	71·5 (63·1–80·7)	10·0 (4·3–16·0)	37·6 (31·7–44·8)	0·9 (−0·7–2·5)
Sweden	80·2 (72·5–86·9)	3·5 (0·1–6·9)	93·8 (89·4–96·8)	2·6 (0·8–4·6)	36·7 (30·8–43·3)	0·9 (−0·6–2·3)
Switzerland	69·7 (62·4–77·5)	5·7 (0·7–11·3)	88·4 (83·1–92·9)	6·0 (2·4–9·1)	41·6 (35·4–48·5)	0·9 (−3·1–4·5)
UK	74·2 (66·7–81·1)	3·2 (1·9–4·3)	88·5 (83·4–93·2)	1·1 (0·2–2·0)	36·2 (30·3–42·9)	−1·7 (−2·3–−1·2)
**Latin America and Caribbean**	**63·6** **(55·8–71·5)**	**11·6** **(9·5–13·4)**	**91·4** **(87·4–94·7)**	**2·9** **(2·0–4·1)**	**41·9** **(35·7–48·9)**	**3·8** **(2·9–4·5)**
Andean Latin America	57·3 (51·1–63·4)	7·2 (5·6–9·0)	82·4 (76·8–87·6)	8·8 (6·5–11·2)	42·9 (36·9–49·4)	3·9 (2·7–5·1)
Bolivia	50·3 (44·1–56·9)	4·6 (2·6–7·1)	74·3 (66·3–81·4)	11·2 (9·2–13·7)	40·9 (34·7–48·4)	3·3 (2·1–4·6)
Ecuador	61·8 (55·1–69·1)	8·8 (3·5–13·5)	92·4 (89·1–95·0)	3·3 (0·8–6·1)	44·6 (38·1–51·2)	4·1 (1·5–6·7)
Peru	57·0 (50·7–63·6)	6·8 (4·1–10·1)	75·8 (68·5–82·6)	13·4 (10·7–15·9)	42·0 (35·5–48·9)	4·0 (2·0–5·8)
Caribbean	63·4 (57·0–70·1)	4·3 (3·0–5·4)	90·9 (86·8–94·1)	1·0 (0·3–1·6)	41·1 (35·3–48·2)	3·1 (2·3–4·1)
Antigua and Barbuda	67·2 (59·4–74·8)	5·6 (2·7–8·1)	93·2 (89·5–96·1)	0·8 (−0·4–1·9)	40·9 (34·5–47·9)	3·2 (1·5–5·1)
The Bahamas	61·3 (53·5–69·1)	5·8 (3·3–8·3)	89·2 (84·1–93·3)	0·8 (−0·8–2·1)	40·2 (33·3–48·3)	3·3 (1·7–5·1)
Barbados	69·9 (62·5–77·9)	4·6 (1·8–7·6)	92·1 (88·1–95·4)	1·0 (−0·6–2·4)	43·0 (36·7–51·1)	3·6 (1·4–5·8)
Belize	59·6 (53·0–66·8)	7·4 (4·7–9·8)	86·9 (81·2–91·4)	5·0 (3·0–7·6)	41·3 (34·6–48·2)	2·0 (0·4–4·1)
Bermuda	80·3 (73·0–86·3)	7·1 (4·0–10·4)	97·7 (96·1–98·7)	0·8 (0·1–1·6)	45·4 (40·1–52·0)	5·6 (3·4–7·9)
Cuba	73·3 (66·1–80·0)	8·2 (5·2–11·2)	96·2 (93·8–97·8)	0·9 (0·3–1·8)	41·8 (35·6–48·9)	4·1 (2·0–5·7)
Dominica	61·1 (53·8–68·6)	2·1 (0·2–4·4)	84·6 (78·4–90·1)	−1·2 (−2·9–0·3)	42·5 (36·1–49·9)	3·0 (1·2–5·0)
Dominican Republic	57·5 (50·7–65·5)	5·2 (3·2–6·9)	87·5 (82·2–92·0)	0·8 (−0·5–2·1)	39·1 (32·6–45·8)	2·6 (1·2–4·0)
Grenada	63·9 (56·3–71·8)	4·0 (1·7–6·5)	89·4 (83·9–93·7)	−0·2 (−1·6–1·1)	41·5 (34·4–49·1)	3·2 (1·3–5·0)
Guyana	69·2 (62·8–75·5)	7·9 (4·6–11·5)	91·3 (87·7–94·3)	2·9 (0·8–6·1)	38·4 (33·1–45·4)	3·4 (1·0–5·4)
Haiti	46·5 (41·0–53·1)	2·3 (0·7–4·0)	78·4 (71·4–84·9)	3·7 (1·7–5·9)	34·6 (29·0–41·9)	1·5 (0·3–2·9)
Jamaica	63·5 (56·3–70·8)	3·2 (0·5–5·6)	91·2 (86·7–94·5)	0·2 (−1·0–1·4)	41·0 (34·6–47·8)	2·6 (0·9–4·1)
Puerto Rico	78·1 (70·1–85·4)	8·7 (4·9–12·0)	96·4 (94·0–98·1)	2·2 (1·1–3·9)	45·3 (40·0–52·2)	4·8 (2·7–6·7)
Saint Kitts and Nevis	62·9 (55·0–71·2)	6·8 (3·1–10·1)	88·0 (82·1–92·7)	1·4 (−1·1–3·8)	40·6 (34·0–48·5)	2·3 (−0·5–5·2)
Saint Lucia	52·3 (47·3–58·3)	3·4 (−0·6–6·7)	85·4 (80·3–89·4)	0·6 (−2·1–3·2)	41·7 (35·8–48·2)	2·2 (−0·2–4·8)
Saint Vincent and the Grenadines	63·6 (55·9–71·6)	5·4 (3·3–7·7)	90·0 (84·9–93·9)	0·8 (−0·5–2·2)	40·8 (34·2–48·6)	3·3 (1·7–5·3)
Suriname	58·1 (50·9–66·1)	7·1 (4·8–9·5)	86·8 (81·2–91·5)	3·0 (1·4–4·7)	38·3 (31·9–45·2)	2·6 (1·1–4·2)
Trinidad and Tobago	66·0 (57·9–73·7)	7·0 (4·4–9·8)	90·3 (85·4–94·0)	3·5 (1·7–5·4)	41·6 (34·6–49·6)	3·4 (1·7–5·1)
Virgin Islands	72·7 (65·4–80·1)	10·6 (6·6–14·0)	93·8 (90·2–96·6)	5·0 (2·9–7·7)	44·3 (37·8–51·5)	5·2 (2·8–7·7)
Central Latin America	63·4 (55·7–71·5)	13·1 (10·9–15·3)	92·4 (88·7–95·4)	2·4 (1·5–3·5)	41·4 (35·0–48·5)	6·0 (4·7–7·1)
Colombia	67·3 (59·1–76·0)	12·7 (8·5–17·2)	96·1 (93·9–97·7)	3·4 (2·0–4·9)	52·2 (45·5–59·5)	3·9 (1·5–6·1)
Costa Rica	66·8 (58·6–75·0)	9·8 (3·3–15·5)	94·6 (91·7–97·0)	2·4 (0·2–4·4)	43·2 (36·9–50·4)	2·9 (−0·8–7·1)
El Salvador	57·1 (49·6–65·5)	6·9 (4·8–9·4)	90·7 (86·4–94·5)	3·0 (1·6–4·5)	43·7 (37·2–50·7)	1·8 (0·3–3·4)
Guatemala	47·3 (43·6–50·8)	5·9 (2·2–9·4)	83·5 (79·0–87·3)	5·2 (1·4–9·1)	42·5 (36·5–50·0)	1·9 (0·5–3·3)
Honduras	46·6 (40·4–53·9)	4·8 (3·3–6·6)	84·3 (78·7–89·1)	4·0 (2·6–5·4)	40·4 (34·4–47·1)	1·2 (0·1–2·4)
Mexico	65·4 (57·1–74·4)	15·5 (12·9–18·2)	92·5 (88·6–95·7)	2·5 (1·4–3·9)	37·9 (31·6–44·7)	6·8 (5·5–8·0)
Nicaragua	56·8 (49·8–64·7)	8·7 (6·3–11·5)	91·8 (88·1–95·1)	3·8 (2·3–5·4)	42·6 (36·1–49·6)	1·6 (0·1–2·9)
Panama	66·8 (58·5–75·0)	9·3 (6·3–12·5)	96·0 (93·5–97·8)	1·7 (0·8–2·6)	42·8 (36·3–50·2)	2·3 (0·8–3·7)
Venezuela	56·7 (48·9–65·1)	5·9 (3·4–8·5)	89·6 (84·5–93·7)	−0·6 (−2·3–0·9)	43·7 (37·0–51·3)	2·1 (0·3–3·8)
Tropical Latin America	65·3 (57·1–73·8)	12·2 (9·5–14·5)	91·3 (86·9–95·0)	4·0 (2·6–5·5)	42·8 (36·5–50·0)	1·1 (0·2–1·8)
Brazil	65·5 (57·2–74·0)	12·5 (9·7–14·9)	91·4 (87·0–95·0)	4·1 (2·7–5·6)	42·8 (36·5–50·1)	1·1 (0·3–1·9)
Paraguay	59·8 (52·4–68·9)	3·3 (1·1–5·5)	88·1 (81·9–93·0)	0·7 (−0·6–1·9)	41·7 (35·2–49·3)	−0·2 (−1·6–1·4)
**North Africa and Middle East**	**65·4** **(59·8–71·2)**	**9·0** **(7·3–11·0)**	**92·5** **(89·5–94·8)**	**4·1** **(2·7–5·4)**	**45·0** **(39·0–51·4)**	**3·0** **(2·1–4·2)**
Afghanistan	41·5 (36·0–47·9)	3·9 (−0·6–9·1)	92·8 (89·6–95·0)	3·8 (1·2–7·6)	46·2 (39·3–54·1)	3·2 (−0·2–6·7)
Algeria	64·7 (59·8–69·8)	14·6 (11·5–17·9)	93·1 (90·4–95·2)	3·4 (1·4–5·8)	41·6 (36·6–47·5)	−2·5 (−6·1–1·3)
Bahrain	68·9 (59·3–77·9)	14·0 (9·7–18·3)	94·4 (90·9–97·2)	5·8 (3·5–8·5)	44·1 (36·9–52·4)	2·7 (0·4–4·7)
Egypt	61·8 (57·9–66·2)	−2·9 (−5·5–1·3)	89·2 (85·9–92·1)	−0·9 (−2·5–1·5)	43·5 (36·0–50·8)	2·9 (1·2–4·5)
Iran	72·4 (65·3–79·5)	9·7 (8·1–11·5)	86·8 (80·6–92·1)	4·8 (3·4–6·4)	43·4 (36·9–50·7)	−3·7 (−5·0–−2·2)
Iraq	60·3 (54·9–66·9)	14·2 (9·8–19·0)	94·1 (91·9–96·0)	9·7 (5·7–14·1)	36·5 (31·3–42·7)	2·2 (−0·6–4·7)
Jordan	78·8 (73·2–83·5)	16·8 (11·6–21·1)	95·0 (92·6–96·9)	10·3 (6·1–13·8)	43·5 (38·2–48·6)	7·2 (4·5–10·4)
Kuwait	74·7 (67·2–82·4)	6·8 (0·7–11·9)	94·9 (91·7–97·3)	1·5 (−0·6–3·9)	33·7 (28·6–39·6)	7·5 (3·4–13·1)
Lebanon	51·3 (46·0–57·4)	7·9 (3·6–11·3)	94·3 (91·7–96·5)	5·4 (2·7–9·0)	50·8 (44·1–56·3)	2·1 (−0·5–4·3)
Libya	56·6 (49·3–64·8)	4·5 (0·4–9·7)	84·9 (79·2–90·7)	−3·2 (−6·8–0·4)	39·8 (33·0–47·5)	2·7 (0·0–6·0)
Morocco	55·5 (50·6–60·6)	9·3 (5·3–13·5)	89·2 (85·8–92·1)	6·8 (3·2–11·1)	36·3 (30·7–41·9)	2·2 (−0·1–4·8)
Oman	63·3 (55·1–72·1)	6·6 (2·8–10·1)	93·4 (89·3–96·3)	1·4 (−0·2–2·7)	44·3 (37·5–52·0)	3·8 (2·2–5·7)
Palestine	70·6 (62·2–78·5)	10·0 (3·5–17·1)	95·5 (92·6–97·7)	4·7 (2·5–6·9)	31·8 (26·5–38·4)	2·5 (−0·7–6·6)
Qatar	75·5 (67·4–83·5)	10·8 (5·1–16·5)	95·1 (91·5–97·7)	7·3 (3·6–11·6)	52·8 (45·8–61·5)	4·3 (2·4–6·3)
Saudi Arabia	68·3 (59·4–78·0)	10·0 (4·6–16·5)	98·4 (97·3–99·3)	1·2 (0·4–2·1)	60·7 (56·7–64·8)	16·3 (11·3–24·4)
Sudan	58·6 (53·7–63·8)	8·3 (4·6–12·0)	90·5 (87·5–93·3)	5·5 (2·5–9·6)	29·1 (24·3–34·2)	2·7 (0·4–4·6)
Syria	67·3 (58·6–76·6)	11·6 (8·3–15·0)	92·9 (88·8–96·2)	2·1 (0·8–3·5)	43·3 (35·7–50·8)	2·6 (0·8–4·5)
Tunisia	75·6 (67·1–84·1)	11·1 (7·4–15·1)	96·8 (94·5–98·4)	2·2 (1·1–3·8)	41·6 (34·4–49·1)	2·0 (0·1–3·8)
Türkiye	73·6 (65·9–81·2)	16·0 (12·1–20·2)	95·3 (92·2–97·5)	7·5 (4·7–10·4)	48·8 (41·7–56·0)	2·6 (1·2–4·1)
United Arab Emirates	76·0 (66·8–85·3)	15·8 (9·9–21·2)	94·2 (90·5–97·2)	1·2 (−1·0–3·8)	58·5 (52·1–65·8)	2·1 (−2·0–5·6)
Yemen	53·6 (46·3–62·6)	5·5 (3·1–7·7)	90·2 (86·0–94·0)	4·1 (2·4–5·8)	38·4 (31·7–45·7)	1·3 (−0·1–2·6)
**South Asia**	**43·8** **(37·0–51·2)**	**12·0** **(9·9–14·3)**	**95·7** **(93·7–97·1)**	**6·3** **(4·6–8·2)**	**34·3** **(28·3–40·7)**	**−0·2** **(−1·3–0·7)**
Bangladesh	51·0 (47·6–54·9)	5·4 (1·4–9·6)	91·8 (89·7–93·5)	5·2 (2·4–8·9)	40·3 (36·0–45·0)	−5·4 (−11·0–0·7)
Bhutan	55·4 (49·4–62·2)	16·9 (12·2–22·0)	91·4 (87·6–94·4)	11·5 (8·4–15·6)	62·9 (56·5–68·6)	6·6 (2·6–9·3)
India	43·6 (36·1–51·7)	13·9 (11·4–16·8)	97·2 (95·8–98·2)	5·1 (3·7–6·9)	33·1 (27·1–39·7)	1·0 (0·3–2·2)
Nepal	41·4 (36·1–47·1)	5·1 (2·7–7·5)	81·5 (75·4–86·8)	8·9 (5·9–12·6)	32·0 (26·3–38·4)	1·9 (0·2–3·6)
Pakistan	41·2 (35·1–48·4)	4·5 (2·9–6·3)	87·3 (82·6–91·3)	7·5 (5·9–9·2)	40·8 (33·9–48·3)	−1·7 (−3·5–0·2)
**Southeast Asia, east Asia, and Oceania**	**47·2** **(41·0–54·1)**	**10·0** **(7·3–13·1)**	**87·5** **(83·0–91·5)**	**15·2** **(12·5–17·7)**	**38·8** **(32·9–45·6)**	**−0·1** **(−1·3–1·1)**
East Asia	45·4 (39·2–52·3)	9·4 (6·7–12·8)	86·5 (81·6–90·7)	17·2 (14·2–19·9)	35·4 (29·7–42·2)	−1·5 (−2·8–−0·2)
China	45·2 (39·0–52·1)	9·5 (6·7–12·9)	86·4 (81·5–90·7)	17·6 (14·6–20·3)	35·3 (29·6–42·1)	−1·6 (−2·9–−0·3)
North Korea	40·0 (34·8–46·3)	4·5 (2·6–6·4)	78·8 (72·1–84·8)	10·4 (8·0–12·5)	35·9 (29·8–43·1)	0·5 (−1·0–2·1)
Taiwan	57·0 (49·2–65·3)	11·3 (8·7–14·6)	92·7 (89·1–95·6)	4·5 (2·9–6·7)	39·4 (33·7–46·3)	1·7 (−0·3–3·6)
Oceania	45·4 (39·7–52·4)	3·1 (1·3–5·1)	82·1 (75·8–87·8)	1·8 (0·5–3·3)	24·4 (19·7–30·1)	0·6 (−0·3–1·7)
American Samoa	53·3 (50·5–56·6)	9·8 (5·8–13·7)	85·4 (82·7–87·9)	0·5 (−2·8–4·0)	37·2 (34·0–40·2)	5·0 (1·2–9·2)
Cook Islands	46·3 (41·1–52·9)	8·3 (5·3–12·1)	85·3 (80·0–89·9)	7·1 (5·1–9·5)	35·4 (29·6–42·4)	3·8 (1·4–6·3)
Fiji	61·0 (53·5–68·8)	4·4 (0·0–9·6)	81·7 (74·0–88·8)	−0·5 (−5·5–3·6)	28·1 (22·3–34·7)	1·3 (−0·9–3·8)
Guam	60·8 (52·8–69·8)	7·3 (4·3–10·6)	93·4 (89·9–96·4)	0·6 (−0·4–1·7)	26·4 (21·2–32·7)	2·1 (−0·1–4·3)
Kiribati	40·8 (36·0–46·3)	1·9 (0·0–3·9)	69·6 (61·2–76·1)	−1·0 (−3·9–1·8)	24·8 (19·9–30·7)	1·8 (0·3–3·3)
Marshall Islands	40·3 (38·3–42·4)	3·3 (−0·4–6·5)	67·2 (64·1–70·0)	2·1 (−3·1–7·6)	18·6 (16·6–20·9)	2·5 (−0·2–4·9)
Federated States of Micronesia	37·5 (35·5–39·9)	3·9 (1·8–6·5)	68·7 (65·7–72·3)	1·3 (−2·4–5·4)	17·4 (15·1–20·1)	−2·6 (−6·4–0·6)
Nauru	60·7 (54·8–65·8)	4·6 (2·7–6·8)	67·8 (60·5–75·0)	8·0 (5·4–11·2)	37·1 (30·7–44·1)	−1·7 (−5·7–1·0)
Niue	57·4 (50·5–65·4)	0·9 (−1·0–2·8)	81·6 (74·3–88·1)	−2·7 (−4·4–−0·9)	24·3 (19·6–29·9)	1·1 (−0·3–2·4)
Northern Mariana Islands	54·1 (47·3–63·3)	12·3 (8·1–17·4)	86·8 (80·2–92·3)	0·6 (−2·7–4·0)	27·0 (21·1–33·8)	2·9 (−0·4–6·5)
Palau	53·4 (50·6–56·2)	6·2 (0·7–11·1)	79·8 (76·9–83·0)	−0·1 (−5·3–5·5)	22·5 (20·1–25·6)	2·2 (−1·7–5·4)
Papua New Guinea	42·8 (36·6–50·6)	3·9 (2·1–6·1)	86·0 (80·5–90·8)	3·6 (2·1–5·1)	21·9 (17·4–27·7)	0·6 (−0·5–1·9)
Samoa	36·5 (31·9–41·1)	−2·1 (−4·9–0·5)	75·4 (68·4–80·7)	0·6 (−2·8–3·9)	29·7 (24·3–36·4)	2·3 (0·2–4·5)
Solomon Islands	36·7 (32·5–41·5)	0·9 (−0·5–2·4)	46·1 (39·0–53·9)	−0·3 (−2·9–2·1)	26·1 (20·8–32·8)	0·3 (−1·4–1·9)
Tokelau	59·7 (54·2–64·6)	0·7 (−1·6–3·3)	72·4 (65·9–78·6)	−2·6 (−5·6–0·5)	17·0 (13·6–21·5)	0·8 (−0·5–2·2)
Tonga	50·6 (44·1–57·9)	3·2 (1·5–5·4)	78·2 (71·1–85·2)	−0·1 (−2·3–1·7)	21·1 (16·7–26·3)	1·4 (0·2–2·7)
Tuvalu	58·3 (52·3–65·0)	2·5 (−0·1–4·9)	80·7 (72·9–87·0)	1·1 (−1·9–3·8)	25·8 (20·8–31·9)	2·9 (1·1–4·8)
Vanuatu	24·8 (21·2–29·2)	1·8 (0·8–2·7)	77·8 (71·2–83·5)	2·2 (0·8–3·7)	43·0 (35·4–50·7)	1·1 (−0·6–2·7)
Southeast Asia	52·4 (46·1–59·4)	9·8 (7·5–12·5)	90·2 (86·2–93·8)	7·0 (5·7–8·7)	46·4 (40·1–53·3)	1·9 (0·3–3·3)
Cambodia	61·5 (54·3–70·2)	9·7 (6·0–14·2)	88·7 (82·8–93·5)	11·5 (8·8–14·9)	51·5 (44·8–58·6)	1·3 (−0·9–3·2)
Indonesia	48·1 (41·6–56·3)	8·8 (6·8–11·5)	87·5 (82·3–92·1)	7·2 (5·7–8·9)	43·5 (36·8–50·6)	2·0 (0·8–3·2)
Laos	53·2 (47·7–59·2)	6·7 (4·5–9·0)	84·6 (79·6–89·3)	14·8 (11·1–18·3)	40·2 (33·5–47·1)	2·0 (0·1–3·9)
Malaysia	47·4 (40·0–55·5)	11·9 (5·5–19·5)	96·5 (94·7–98·0)	2·8 (1·2–4·1)	53·7 (47·2–60·6)	2·3 (−2·1–6·4)
Maldives	43·3 (38·0–49·7)	6·0 (3·4–8·8)	90·8 (87·3–93·6)	8·4 (5·6–11·8)	45·5 (39·1–53·0)	2·3 (0·2–4·7)
Mauritius	60·2 (52·6–69·4)	8·5 (5·1–12·4)	92·6 (88·5–96·0)	2·2 (0·9–4·1)	47·8 (41·0–55·3)	3·5 (1·3–5·6)
Myanmar	56·3 (51·2–61·7)	10·8 (6·8–14·5)	89·0 (84·9–92·0)	13·7 (9·3–18·8)	43·8 (38·0–50·0)	1·1 (−1·8–3·6)
Philippines	47·8 (41·3–55·5)	5·1 (3·8–6·6)	88·5 (83·6–92·8)	2·7 (1·9–3·5)	43·6 (37·1–50·6)	1·5 (0·5–2·3)
Seychelles	60·6 (52·3–69·7)	7·9 (3·4–13·8)	94·9 (91·8–97·2)	1·3 (0·0–2·7)	20·2 (15·6–26·3)	1·6 (−0·1–3·9)
Sri Lanka	73·2 (68·1–78·2)	14·0 (9·1–19·0)	90·3 (86·6–93·7)	14·0 (8·7–19·9)	53·8 (48·0–58·6)	3·0 (−1·1–6·7)
Thailand	59·6 (50·8–69·1)	16·2 (8·8–22·8)	96·6 (94·8–98·0)	2·0 (0·8–3·1)	37·1 (30·8–44·1)	2·5 (−4·9–7·7)
Timor-Leste	24·7 (20·2–29·7)	2·4 (0·9–4·4)	81·3 (74·2–87·2)	9·9 (6·7–13·7)	45·6 (36·5–53·9)	1·5 (−0·6–3·9)
Viet Nam	51·3 (45·4–57·9)	8·8 (4·1–12·7)	91·2 (87·9–94·3)	7·0 (4·0–10·0)	62·9 (57·1–68·6)	−0·9 (−4·5–1·8)
**Sub-Saharan Africa**	**34·2** **(29·7–38·8)**	**0·8** **(0·2–1·7)**	**81·0** **(75·2–85·9)**	**5·4** **(4·5–6·6)**	**48·4** **(41·9–55·4)**	**1·0** **(0·3–1·6)**
Central sub-Saharan Africa	16·3 (14·0–18·9)	0·6 (−0·2–1·4)	69·1 (62·5–75·3)	11·6 (9·8–13·5)	50·8 (44·1–58·3)	1·2 (0·1–2·4)
Angola	18·2 (15·8–20·6)	−0·3 (−1·6–0·8)	62·8 (56·1–69·4)	13·4 (11·1–15·8)	60·8 (53·3–68·3)	2·8 (1·3–4·3)
Central African Republic	15·9 (13·9–18·2)	1·3 (0·6–2·0)	77·3 (71·6–82·0)	3·9 (2·1–5·8)	53·7 (47·5–60·3)	2·0 (0·0–3·7)
Congo (Brazzaville)	17·2 (14·9–19·6)	0·9 (0·0–1·8)	71·1 (64·3–77·3)	10·4 (8·3–12·6)	54·0 (47·3–60·8)	1·0 (−0·7–2·5)
DR Congo	15·3 (13·0–17·6)	0·6 (−0·2–1·5)	71·8 (64·6–78·2)	12·5 (10·0–15·0)	46·3 (39·5–53·6)	−0·4 (−1·9–1·3)
Equatorial Guinea	16·4 (14·0–18·9)	0·4 (−0·6–1·5)	77·6 (71·6–83·3)	14·0 (11·4–17·4)	54·6 (48·0–60·8)	1·6 (−0·8–3·5)
Gabon	19·9 (17·1–22·7)	1·8 (0·7–2·9)	71·3 (63·8–77·5)	8·4 (6·3–11·0)	57·2 (50·4–64·2)	2·1 (0·4–3·6)
Eastern sub-Saharan Africa	37·8 (33·0–42·6)	2·0 (1·1–3·1)	79·8 (74·1–84·9)	7·7 (6·2–9·2)	54·8 (48·3–61·3)	0·6 (0·0–1·3)
Burundi	36·7 (31·7–42·3)	0·0 (−1·6–1·8)	82·9 (77·2–87·8)	6·8 (5·0–8·9)	53·0 (46·8–59·0)	−0·4 (−2·0–1·3)
Comoros	53·1 (45·9–60·0)	5·4 (3·4–7·5)	90·8 (86·4–94·5)	3·9 (2·3–5·7)	44·7 (38·0–51·2)	1·5 (0·3–3·0)
Djibouti	42·0 (36·2–48·8)	6·7 (4·7–9·2)	86·0 (81·1–90·4)	7·9 (5·9–9·8)	54·2 (47·9–61·1)	1·2 (0·0–2·5)
Eritrea	58·7 (51·7–66·6)	7·6 (3·8–11·7)	92·8 (88·5–96·0)	4·3 (2·3–6·7)	52·3 (46·0–58·7)	0·2 (−1·5–2·3)
Ethiopia	22·5 (19·1–25·3)	−0·8 (−2·3–0·5)	70·1 (62·8–75·8)	19·5 (17·5–21·3)	54·7 (48·0–61·0)	1·2 (0·0–2·3)
Kenya	46·4 (40·7–52·8)	0·5 (−0·3–1·3)	85·1 (79·9–89·8)	−1·7 (−2·3–−1·0)	60·2 (54·2–66·6)	2·9 (2·3–3·4)
Madagascar	37·9 (32·8–43·4)	1·8 (0·1–3·7)	85·2 (80·4–89·5)	5·3 (3·4–7·4)	52·9 (46·4–59·4)	0·2 (−1·2–1·8)
Malawi	46·4 (40·9–51·9)	0·8 (−1·2–2·7)	78·2 (71·5–84·2)	7·3 (4·6–10·2)	61·4 (53·3–68·5)	0·6 (−1·5–3·0)
Mozambique	44·0 (36·9–51·4)	0·5 (−1·4–2·9)	87·3 (81·8–91·8)	2·7 (1·2–4·5)	65·7 (53·6–75·0)	−0·1 (−2·1–2·0)
Rwanda	34·0 (29·3–38·6)	5·5 (3·6–7·7)	84·4 (79·0–88·7)	15·8 (12·7–19·4)	66·6 (56·3–75·5)	2·3 (0·4–4·3)
Somalia	36·4 (31·7–40·8)	0·8 (−0·7–2·2)	71·6 (64·4–78·5)	1·7 (−0·2–3·9)	54·8 (48·0–61·7)	1·3 (−0·8–3·1)
South Sudan	36·9 (32·2–42·1)	1·2 (−0·6–3·1)	79·1 (72·7–84·7)	1·5 (−0·6–3·4)	53·5 (47·3–60·7)	−0·2 (−2·1–1·8)
Uganda	47·1 (41·8–52·5)	4·0 (1·8–6·2)	84·7 (79·5–89·6)	9·5 (6·5–12·2)	67·1 (61·3–72·4)	0·0 (−1·4–1·4)
Tanzania	48·2 (42·4–53·7)	2·5 (0·8–4·4)	77·0 (70·1–83·3)	5·4 (3·4–7·3)	42·6 (36·2–49·0)	0·9 (−0·6–2·4)
Zambia	25·2 (21·9–28·5)	−1·2 (−3·0–0·5)	76·0 (69·9–81·3)	9·3 (6·5–12·2)	32·5 (27·1–38·4)	0·7 (−1·3–2·6)
Southern sub-Saharan Africa	52·4 (45·0–59·8)	0·5 (−0·6–1·8)	88·7 (83·9–92·9)	−1·1 (−1·9–−0·4)	36·7 (30·8–43·9)	−1·9 (−2·6–−1·1)
Botswana	51·8 (46·2–58·2)	7·0 (4·5–9·3)	91·7 (88·1–94·6)	0·6 (−0·8–1·9)	53·7 (47·7–60·4)	3·6 (1·4–5·7)
Eswatini	46·8 (40·6–53·3)	5·7 (3·5–8·0)	94·0 (91·3–96·1)	0·6 (−0·2–1·5)	50·1 (43·9–56·4)	−0·3 (−2·5–1·8)
Lesotho	57·2 (50·2–64·4)	−1·0 (−2·8–1·2)	92·2 (88·5–95·1)	−1·5 (−2·5–−0·7)	45·6 (38·6–52·8)	0·8 (−0·7–2·4)
Namibia	54·2 (46·2–62·4)	4·5 (1·6–7·3)	95·0 (92·7–97·0)	1·2 (0·2–2·4)	37·7 (31·4–44·2)	2·2 (0·2–3·9)
South Africa	52·8 (45·3–60·4)	0·3 (−0·9–1·8)	88·0 (83·0–92·4)	−1·0 (−1·8–−0·2)	35·2 (29·4–42·2)	−2·4 (−3·4–−1·6)
Zimbabwe	48·5 (41·6–55·6)	−1·2 (−3·4–0·8)	92·1 (88·7–94·8)	−2·9 (−4·4–−1·9)	43·3 (36·7–49·9)	1·1 (0·0–2·4)
Western sub-Saharan Africa	32·0 (27·8–36·5)	1·6 (0·7–2·7)	80·1 (74·3–85·3)	8·1 (6·7–9·6)	48·3 (42·1–55·2)	1·3 (0·4–2·2)
Benin	25·9 (22·2–29·3)	0·1 (−1·4–1·6)	85·3 (80·6–88·7)	6·5 (4·6–8·9)	52·3 (46·0–58·3)	0·6 (−1·4–2·7)
Burkina Faso	30·6 (26·5–34·6)	0·6 (−0·5–1·8)	80·1 (73·9–85·2)	5·8 (4·0–7·7)	47·5 (40·8–54·1)	0·3 (−1·6–1·8)
Cabo Verde	57·4 (51·8–63·0)	10·1 (6·1–14·0)	91·5 (87·9–94·1)	5·2 (2·4–8·5)	39·9 (34·4–46·3)	0·3 (−2·5–3·3)
Cameroon	41·3 (35·8–46·9)	1·0 (−2·2–3·9)	87·6 (83·4–91·7)	5·0 (2·7–7·4)	55·1 (48·9–61·6)	0·9 (−1·7–3·5)
Chad	26·0 (22·7–30·0)	−1·1 (−2·1–0·0)	74·9 (68·5–80·9)	5·6 (3·9–7·6)	45·4 (39·0–52·5)	−0·3 (−1·8–1·1)
Côte d'Ivoire	27·5 (24·1–32·0)	1·7 (0·3–3·2)	76·3 (69·7–82·2)	8·3 (6·6–10·3)	47·3 (41·2–54·3)	1·1 (−0·2–2·6)
The Gambia	27·9 (24·2–31·6)	1·0 (0·0–1·8)	76·3 (70·4–82·1)	4·7 (3·1–6·7)	48·2 (41·8–54·6)	1·2 (0·0–2·6)
Ghana	50·5 (44·8–56·7)	1·8 (−0·3–4·1)	79·2 (72·3–85·5)	5·9 (3·7–8·4)	44·2 (38·0–50·7)	1·3 (−0·2–2·9)
Guinea	34·5 (30·0–39·3)	−2·2 (−4·0–−0·2)	75·1 (68·2–81·3)	6·4 (3·9–8·8)	53·0 (45·2–62·2)	−2·5 (−5·9–0·3)
Guinea-Bissau	26·0 (22·6–29·5)	−0·4 (−1·6–0·7)	68·8 (61·7–75·3)	8·5 (6·5–10·2)	46·6 (40·2–53·3)	0·3 (−1·7–2·0)
Liberia	31·9 (27·4–36·9)	1·4 (−0·7–3·3)	83·8 (77·8–88·8)	5·9 (2·9–8·8)	50·2 (41·9–58·4)	0·5 (−2·3–3·1)
Mali	23·3 (20·0–26·7)	1·7 (0·5–3·1)	74·7 (68·1–80·3)	6·5 (4·4–9·2)	39·2 (32·1–46·7)	0·6 (−1·2–2·6)
Mauritania	27·4 (22·5–33·1)	2·3 (0·6–4·6)	74·4 (65·9–82·1)	10·4 (7·9–12·8)	53·3 (44·8–61·0)	2·7 (0·7–4·9)
Niger	10·7 (8·5–12·9)	0·6 (−0·2–1·4)	68·7 (59·3–78·1)	11·2 (7·5–13·9)	44·0 (35·7–52·7)	0·7 (−1·0–2·5)
Nigeria	32·3 (27·8–36·8)	2·0 (0·9–3·4)	81·7 (76·0–86·6)	9·7 (8·1–11·6)	48·9 (42·5–55·7)	1·6 (0·4–2·6)
São Tomé and Príncipe	34·6 (30·3–39·0)	0·5 (−1·9–2·5)	76·3 (70·4–81·9)	7·5 (4·5–10·4)	50·5 (43·3–57·9)	−1·4 (−4·3–1·6)
Senegal	29·6 (25·9–33·8)	1·8 (0·5–3·3)	80·1 (74·5–85·1)	7·6 (5·8–9·3)	48·0 (41·9–54·7)	0·9 (−0·4–2·3)
Sierra Leone	27·8 (24·3–32·0)	−2·7 (−4·3–−1·2)	78·0 (71·3–83·4)	6·4 (4·1–9·0)	46·8 (40·6–53·2)	0·0 (−2·4–2·4)
Togo	30·4 (26·4–34·7)	3·9 (2·6–5·4)	77·9 (71·6–83·4)	7·5 (5·7–9·2)	47·7 (41·1–54·9)	1·5 (0·1–3·3)

## Data Availability

To download the data used in these analyses, please visit the Global Health Data Exchange website https://ghdx.healthdata.org/record/ihme-data/diabetes-cascade-of-care-estimates-2000-2023 Data sources are also listed by location and institution in the appendix (table S19).

## Research in context

### Evidence before this study

The Global Burden of Diseases, Injuries, and Risk Factors 2021 Study (GBD 2021) estimated that in 2021, 529 million people were living with diabetes, which was forecast to reach 1.3 billion people by 2050. Diabetes was the eighth leading cause of age-standardised disability-adjusted life-years (DALYs) globally in 2021, the result of a 38% increase in DALYs since 1990. Macro- and microvascular complications arising from diabetes can be averted with timely and appropriate diagnosis, coupled with effective treatment and blood glucose management. These activities are collectively known as the diabetes care cascade. We searched the PubMed database for cross-sectional or cohort studies using terms related to “diagnosis”, “treatment”, and “glycaemic control” among people with diabetes and meta-analyses or systematic reviews of the care cascade until May 28, 2024. The most comprehensive studies on the diabetes cascade of care were an analysis of national-level survey data from 28 low- and middle-income countries and a pooled meta-analysis of published literature reporting estimates globally and for select countries. The International Diabetes Federation (IDF) produces global estimates of undiagnosed diabetes in those aged 20–79 years, most recently in 2021. The NCD Risk Factor Collaborators (NCD-RisC) reported on worldwide trends in pharmacological treatment coverage among people with diabetes aged 30 years or older from 1990 to 2022. To our knowledge, there have been no published studies of comprehensive, modelled estimates of all components of the diabetes care cascade—including diagnosis, treatment, and glycaemic management—spanning all countries over time.

### Added value of this study

Global estimates of the diabetes care cascade are vital to comparing performance in diabetes management, including by assessing progress, identifying gaps, and targeting interventions for improvements in care. In this study, we comprehensively assess the proportion of people living with diabetes who are diagnosed with diabetes, the proportion of people with diagnosed diabetes who are treated, and the proportion of people with treated diabetes who have optimal glycaemic levels globally from 2000 to 2023, using GBD data and methods. Our estimates span every region of the world, including 204 countries and territories, and are granular by age and sex.

### Implications of all the available evidence

We find major gaps in diagnosing, treating, and managing diabetes globally, with substantial variation between countries. In 2023, 55.8% of people living with diabetes globally were diagnosed with diabetes, 91.4% of people with diagnosed diabetes were on treatment, and 41.6% of people with treated diabetes had optimal glycaemic levels, demonstrating a serious and growing need for increased health-care access, targeted screening programmes, and improved quality of diabetes care. In combination with the prior GBD study on diabetes prevalence, these estimates can be used by policy makers and health-care professionals globally to target interventions more effectively to remedy shortcomings in the diabetes care cascade.
