# Supplementary material for: Global, regional, and national cascades of diabetes care, 2000–2023: diagnosis, treatment, and glycaemic management
Source: Lancet Diabetes Endocrinol. Author manuscript; Available in PMC 2026 Sep 2. (PMC13537427; doi:10.1016/S2213-8587(25)00217-7)
Supplement: Supplementary file 1 [file NIHMS2096779-supplement-Appendix_1__Supplementary_methods_and_results_to__Global__regional__and_national_cascades_of_diabetes_care__2000_2023__diagnosis__treatment__and_glycaemic_management_.docx]

**Appendix 1: Supplementary methods and results to “Global, regional, and national cascades of diabetes care, 2000–2023: diagnosis, treatment, and glycaemic management”**

This appendix provides supplemental figures and more detailed results for “Global, regional, and national cascades of diabetes care, 2000–2023: diagnosis, treatment, and glycaemic management”.

**Table of Contents**

[List of figures and tables	3](#_Toc198885872)

[Figures	3](#_Toc198885873)

[Tables	3](#_Toc198885874)

[Section 1. Statement of GATHER compliance	5](#_Toc198885875)

[Section 2. Data inputs	6](#_Toc198885876)

[Section 2.1. Data seeking	6](#_Toc198885877)

[Section 2.1.1. Systematic review	6](#_Toc198885878)

[Section 2.1.2. Other data seeking	7](#_Toc198885879)

[Section 2.2. Data coverage	8](#_Toc198885880)

[Section 3. Data processing	14](#_Toc198885881)

[Section 3.1. Microdata aggregation	14](#_Toc198885882)

[Section 3.2. Sex and age splitting	15](#_Toc198885883)

[Section 3.3. Glycaemic management threshold adjustment	15](#_Toc198885884)

[Section 3.4. Outliering	16](#_Toc198885885)

[Section 4. Modelling and post-processing	16](#_Toc198885886)

[Section 4.1. DisMod-MR 2.1 modelling	16](#_Toc198885887)

[Section 4.2. Post-processing	17](#_Toc198885888)

[Section 5. Sensitivity analysis	17](#_Toc198885889)

[Section 6. GBD 2023 diabetes prevalence estimation	19](#_Toc198885890)

[Section 6.1. Case definitions	19](#_Toc198885891)

[Section 6.2. Data inputs and seeking	20](#_Toc198885892)

[Section 6.3. Data processing	21](#_Toc198885893)

[Section 6.4. Modelling and post-processing	25](#_Toc198885894)

[Section 7. References	26](#_Toc198885895)

[Section 8. Additional figures and tables	27](#_Toc198885896)

[Figure S15. Diabetes cascade of care proportions (A) and counts (B) in 2023 for both sexes over age, by Global Burden of Disease region	27](#_Toc198885897)

[Figure S16. Optimal glycaemic management proportion among people with diabetes, both sexes and ages 15 years and older in 2023	28](#_Toc198885898)

[Figure S17. Absolute change in percentage points in diabetes cascade of care proportions between 2000 and 2023 globally for both sexes, by age	29](#_Toc198885899)

[Figure S18. Absolute change in percentage points in diabetes cascade of care proportions between 2000 and 2023 for ages 15 and older and both sexes, by Global Burden of Disease region	29](#_Toc198885900)

[Table S12. Counts of diagnosed diabetes, treated diabetes, and diabetes with optimal glycaemic levels in 2023, and corresponding percent change between 2000 and 2023, for both sexes and ages 15 and older globally, and by Global Burden of Disease super-regions, regions, and countries and territories	30](#_Toc198885901)

[Table S13. Counts of undiagnosed diabetes, diagnosed and untreated diabetes, treated diabetes with suboptimal glycaemic levels, and treated diabetes with optimal glycaemic levels in 2023, and corresponding percent change between 2000 and 2023, for both sexes and ages 15 and older globally, and by Global Burden of Disease super-regions, regions, and countries and territories	38](#_Toc198885902)

[Table S14. Undiagnosed proportion, diagnosed and untreated proportion, treated with suboptimal glycaemic levels proportion, and treated with optimal glycaemic levels proportion among people with diabetes in 2023, and corresponding absolute change between 2000 and 2023, for both sexes and ages 15 and older globally, and by Global Burden of Disease super-regions, regions, and countries and territories	48](#_Toc198885903)

[Table S15. Diagnosed proportion among people living with diabetes, treated proportion among people with diagnosed diabetes, and optimal glycaemic management proportion among people with treated diabetes in 2023, males and females for ages 15 and older globally, and by Global Burden of Disease super-regions, regions, and countries and territories	57](#_Toc198885904)

[Table S16. Diagnosed proportion among people living with diabetes, treated proportion among people with diagnosed diabetes, and optimal glycaemic management proportion among people with treated diabetes in 2023, and corresponding absolute change between 2000 and 2023, for both sexes and ages 15 and older globally, and by Global Burden of Disease super-regions, regions, and countries and territories – sensitivity analysis	65](#_Toc198885905)

[Table S17. Diagnosed proportion among people living with diabetes, treated proportion among people with diagnosed diabetes, and optimal glycaemic management proportion among people with treated diabetes in 2000, and corresponding counts, for both sexes and ages 15 and older globally, and by Global Burden of Disease super-regions, regions, and countries and territories	75](#_Toc198885906)

[Table S18. GBD 2023 diabetes cases counts in 2000 and 2023, for both sexes and ages 15 and older globally, and by Global Burden of Disease super-regions, regions, and countries and territories	83](#_Toc198885907)

[Table S19. Cascade of care input data characteristics and citations	92](#_Toc198885908)

[Table S20. GBD 2023 diabetes prevalence input data citations	117](#_Toc198885909)

[Table S21. Global Burden of Disease 2023 location hierarchy	158](#_Toc198885910)

# List of figures and tables

Figures and tables are available throughout the text and in section 8.

## Figures

Figure S1. PRISMA diagram for diabetes cascade of care

Figure S2. Data inputs map – all models combined

Figure S3. Data inputs map –undiagnosed proportion among those with diabetes model

Figure S4. Data inputs map –diagnosed and untreated proportion among those with diabetes model

Figure S5. Data inputs map –diagnosed and treated proportion among those with diabetes model

Figure S6. Data inputs map –treated with suboptimal glycaemic levels proportion among those with diabetes model

Figure S7. Data inputs map – treated with optimal glycaemic levels proportion among those with diabetes model

Figure S8. Data inputs by study start year and Global Burden of Disease super-region – all models combined

Figure S9. Microdata aggregation flowchart

Figure S10. MR-BRT adjustment factors for suboptimal/optimal glycaemic management threshold adjustment

Figure S11. FPG vs. HbA1c linear regression, sensitivity analysis

Figure S12. MR-BRT adjustment factors for suboptimal/optimal glycaemic management threshold adjustment, sensitivity analysis

Figure S13. PRISMA diagram for GBD 2023 diabetes prevalence

Figure S14. Prevalence of GBD 2023 diabetes female to male sex ratio

Figure S15. Diabetes cascade of care proportions (A) and counts (B) in 2023 for both sexes over age, by Global Burden of Disease region

Figure S16. Optimal glycaemic management proportion among people with diabetes, both sexes and ages 15 years plus in 2023

Figure S17. Absolute change in percentage points in diabetes cascade of care proportions between 2000 and 2023 globally for both sexes, by age

Figure S18. Absolute change in percentage points in diabetes cascade of care proportions between 2000 and 2023 for ages 15 and older and both sexes, by Global Burden of Disease region

## Tables

Table S1. GATHER checklist

Table S2. Data inclusion and exclusion criteria

Table S3. Number of processed datapoints by model – sex and age splitting

Table S4. Number of processed datapoints by model – suboptimal/optimal glycaemic management threshold adjustment

Table S5. DisMod-MR 2.1 model covariates

Table S6. Number of processed datapoints by model – suboptimal/optimal glycaemic management threshold adjustment, sensitivity analysis

Table S7. DisMod-MR 2.1 model covariates, sensitivity analysis

Table S8. Case definitions for GBD 2023 diabetes mellitus prevalence

Table S9. MR-BRT adjustment factors for GBD 2023 diabetes prevalence alternative case definitions

Table S10. MR-BRT adjustment factors for GBD 2023 diabetes prevalence clinical data

Table S11. DisMod-MR 2.1 model covariates, GBD 2023 diabetes prevalence

Table S12. Counts of diagnosed diabetes, treated diabetes, and diabetes with optimal glycaemic levels in 2023, and corresponding percent change between 2000 and 2023, for both sexes and ages 15 and older globally, and by Global Burden of Disease super-regions, regions, and countries and territories

Table S13. Counts of undiagnosed diabetes, diagnosed and untreated diabetes, treated diabetes with suboptimal glycaemic levels, and treated diabetes with optimal glycaemic levels in 2023, and corresponding percent change between 2000 and 2023, for both sexes and ages 15 and older globally, and by Global Burden of Disease super-regions, regions, and countries and territories

Table S14. Undiagnosed proportion, diagnosed and untreated proportion, treated with suboptimal glycaemic levels proportion, and treated with optimal glycaemic levels proportion among people with diabetes in 2023, and corresponding absolute change between 2000 and 2023, for both sexes and ages 15 and older globally, and by Global Burden of Disease super-regions, regions, and countries and territories

Table S15. Diagnosed proportion among people living with diabetes, treated proportion among people with diagnosed diabetes, and optimal glycaemic management proportion among people with treated diabetes in 2023, males and females for ages 15 and older globally, and by Global Burden of Disease super-regions, regions, and countries and territories

Table S16. Diagnosed proportion among people living with diabetes, treated proportion among people with diagnosed diabetes, and optimal glycaemic management proportion among people with treated diabetes in 2023, and corresponding absolute change between 2000 and 2023, for both sexes and ages 15 and older globally, and by Global Burden of Disease super-regions, regions, and countries and territories – sensitivity analysis

Table S17. Diagnosed proportion among people living with diabetes, treated proportion among people with diagnosed diabetes, and optimal glycaemic management proportion among people with treated diabetes in 2000, and corresponding counts, for both sexes and ages 15 and older globally, and by Global Burden of Disease super-regions, regions, and countries and territories

Table S18. GBD 2023 diabetes cases counts in 2000 and 2023, for both sexes and ages 15 and older globally, and by Global Burden of Disease super-regions, regions, and countries and territories

Table S19. Cascade of care input data characteristics and citations

Table S20. GBD 2023 diabetes prevalence input data citations

Table S21. Global Burden of Disease 2023 location hierarchy

# Section 1. Statement of GATHER compliance

This study complies with the Guidelines for Accurate and Transparent Health Estimates Reporting (GATHER) recommendations. Below is the GATHER checklist.^1^

***Table S1. GATHER checklist***

| **#** | **GATHER checklist item** | **Description of compliance** | **Reference** |
| --- | --- | --- | --- |
| **Objectives and funding** | | | |
| **1** | Define the indicator(s), populations (including age, sex, and geographic entities), and time period(s) for which estimates were made. | Narrative provided in paper describing indicators, definitions, populations, and time periods | Main text (Methods) |
| **2** | List the funding sources for the work. | Funding sources listed in paper | Summary (Funding) |
| **Data Inputs** | | | |
| *For all data inputs from multiple sources that are synthesized as part of the study:* | | | |
| **3** | Describe how the data were identified and how the data were accessed. | Narrative description of data seeking methods provided | Main text (Methods) and Appendix (Section 2) |
| **4** | Specify the inclusion and exclusion criteria. Identify all ad-hoc exclusions. | Narrative about inclusion and exclusion criteria provided | Main text (Methods) and Appendix (Section 2) |
| **5** | Provide information on all included data sources and their main characteristics. For each data source used, report reference information or contact name/institution, population represented, data collection method, year(s) of data collection, sex and age range, diagnostic criteria or measurement method, and sample size, as relevant. | Details on all included data sources and relevant characteristics provided in appendix; Each data source used in this analysis is further catalogued in an interactive, online data source tool that provides metadata | Appendix (Section 2) and citations in Appendix (Section 8); Global Health Data Exchange (https://ghdx.healthdata.org/) |
| **6** | Identify and describe any categories of input data that have potentially important biases (e.g., based on characteristics listed in item 5). | Summary of known biases included in appendix | Appendix (Section 2-3) |
| *For data inputs that contribute to the analysis but were not synthesized as part of the study:* | | | |
| **7** | Describe and give sources for any other data inputs. | Included in online data source tool | Global Health Data Exchange (https://ghdx.healthdata.org/) |
| *For all data inputs:* | | | |
| **8** | Provide all data inputs in a file format from which data can be efficiently extracted (e.g., a spreadsheet rather than a PDF), including all relevant meta-data listed in item 5. For any data inputs that cannot be shared because of ethical or legal reasons, such as third-party ownership, provide a contact name or the name of the institution that retains the right to the data. | Downloads of input data available through online data tools; input data not available in tools will be made available upon request | Global Health Data Exchange (https://ghdx.healthdata.org/) |
| **Data analysis** | | | |
| **9** | Provide a conceptual overview of the data analysis method. A diagram may be helpful. | Narrative descriptions of data analysis process provided | Main text (Methods) and Appendix (Sections 3-5) |
| **10** | Provide a detailed description of all steps of the analysis, including mathematical formulae. This description should cover, as relevant, data cleaning, data pre-processing, data adjustments and weighting of data sources, and mathematical or statistical model(s). | Narrative description covering all data extraction, processing, and modelling processes provided | Main text (Methods) and Appendix (Sections 3-5) |
| **11** | Describe how candidate models were evaluated and how the final model(s) were selected. | Provided in main text methods narrative description and appendix | Main text (Methods) and Appendix (Section 4) |
| **12** | Provide the results of an evaluation of model performance, if done, as well as the results of any relevant sensitivity analysis. | Provided in main text methods narrative description and appendix | Main text (Methods) and Appendix (Sections 4-5) |
| **13** | Describe methods for calculating uncertainty of the estimates. State which sources of uncertainty were, and were not, accounted for in the uncertainty analysis. | Provided in main text methods narrative description and appendix | Main text (Methods) and Appendix (Sections 3-4) |
| **14** | State how analytic or statistical source code used to generate estimates can be accessed. | Remote code repository for access to analytic code provided | Remote code repository |
| **Results and Discussion** | | | |
| **15** | Provide published estimates in a file format from which data can be efficiently extracted. | Published estimates available in main text or appendix | Main text (Methods, Results and Discussion), Appendix (Section 8) |
| **16** | Report a quantitative measure of the uncertainty of the estimates (e.g. uncertainty intervals). | Uncertainty provided with all results | Main text (Methods, Results and Discussion), Appendix (Section 8) |
| **17** | Interpret results in light of existing evidence. If updating a previous set of estimates, describe the reasons for changes in estimates. | Discussion of results provided in manuscript narrative | Main text (Methods, Results and Discussion) |
| **18** | Discuss limitations of the estimates. Include a discussion of any modelling assumptions or data limitations that affect interpretation of the estimates. | Discussion of limitations, including modelling assumptions and data limitations, included in manuscript narrative and appendix | Main text (Methods and Discussion), Appendix (Sections 2-4) |

# Section 2. Data inputs

## Section 2.1. Data seeking

### Section 2.1.1. Systematic review

A systematic review of the literature was conducted with the following search terms in PubMed:

(‘undiagnosed diabetes’[TIAB] OR ‘diagnosed diabetes’[TIAB] OR ‘aware diabetes’[TIAB] OR ‘unaware diabetes’[TIAB] OR ‘controlled diabetes’[TIAB] OR ‘uncontrolled diabetes’[TIAB] OR ‘treated diabetes’[TIAB] OR ‘untreated diabetes’[TIAB] OR ‘diabetes on treatment’[TIAB]) NOT (‘randomized controlled trial’[TIAB] OR ‘randomised controlled trial’[TIAB] OR ‘randomised control trial’[TIAB] OR ‘randomized control trial’[TIAB] OR ‘literature review’[TIAB] OR ‘meta-analysis’[TIAB] OR ‘systematic review’[TIAB]) AND (‘proportion’[TIAB] OR ‘prevalence’[TIAB]) AND ("Cross-Sectional Studies"[Mesh] OR "Cohort Studies"[Mesh] OR “cross-sectional”[TIAB] OR “cohort”[TIAB]) NOT (‘Review’[Publication Type] OR ‘Systematic Review’[Publication Type] OR ‘Clinical Trial’[Publication Type]) NOT (‘animal’ [All Fields]) NOT (“NHANES”[TIAB] OR “KNHANES”[TIAB] OR “Health Survey for England”[TIAB] OR “STEPS”[TIAB]) AND 1990/01/01:3000/01/01[dp]

Search date: May 28^th^, 2024

Below is a PRISMA^2^ diagram of the systematic review.

***Figure S1. PRISMA diagram for diabetes cascade of care***

*Removed before screening due to already being captured in other data efforts

**DistillerSR (<https://www.distillersr.com/products/distillersr-systematic-review-software>) was used to assist in title/abstract screening. The first 499 (21%%) records were screened by two reviewers each and all conflicts were resolved collaboratively. Based on these manual decisions, DistillerSR’s AI tool calculated a prediction score for all records that reflects the probability of inclusion. All manually excluded records had a score below 0.416, which was used as a threshold to bulk exclude 1,440 sources from the remaining 1,885 records to be reviewed (76%). The remaining 445 records were screened by one reviewer each.

Below is a table detailing the inclusion and exclusion criteria for the systematic review. A full list of data inputs with citations and additional information can be found in section 7 below.

***Table S2. Data inclusion and exclusion criteria***

| **Criteria** | **Details** |
| --- | --- |
| Measure | Proportion or Prevalence |
| Denominator | People with diabetes   - Must be inclusive of both type 1 and type 2 diabetes - Cannot include people with gestational diabetes - Diabetes must be defined as either:   - Fasting plasma glucose (FPG) greater than or equal to 7 mmol/L (126 mg/dL) or on treatment (insulin or other hypoglycemic medication)   - Glycated hemoglobin (HbA1c) greater than or equal to 6.5% or on treatment (insulin or other hypoglycemic medication) - Cannot include diabetes case definitions that consider self-report only as a basis for diagnosis |
| Numerator | One or more of the following:   1. People with undiagnosed diabetes    a. Above the FPG/HbA1c threshold for diabetes but self-report “no” to previous diabetes diagnosis 2. People with diagnosed diabetes but are not treated with insulin or other hypoglycemic medication    a. Above the FPG/HbA1c threshold for diabetes and self-report “yes” to previous diabetes diagnosis, but self-report “no” to being on insulin or other hypoglycemic medication 3. People with diagnosed diabetes and are treated with insulin or other hypoglycemic medication    a. Self-report “yes” to previous diabetes diagnosis and self-report “yes” to being on insulin or other hypoglycemic medication, regardless of FPG/HbA1c value 4. People with diagnosed diabetes and are treated with insulin or other hypoglycemic medication but have suboptimal glycaemic levels    a. Self-report “yes” to previous diabetes diagnosis and self-report “yes” to being on insulin or other hypoglycemic medication and are above a given FPG/HbA1c threshold 5. People with diagnosed diabetes and are treated with insulin or other hypoglycemic medication and have optimal glycaemic levels    a. Self-report “yes” to previous diabetes diagnosis and self-report “yes” to being on insulin or other hypoglycemic medication and are below a given FPG/HbA1c threshold |
| Study population | Representative of general diabetes population   - Cannot exclude those with comorbid conditions, e.g. cardiovascular or chronic kidney disease - Cannot be among a subpopulation, e.g. a specific ethnic group |
| Study duplicity | Not duplicative of a study where individual-level data is available |

### Section 2.1.2. Other data seeking

Outside of the systematic review, data sources were identified through the following approaches:

1. GBD nonfatal diabetes mellitus data sources
   a. For more information on data seeking for GBD diabetes mellitus nonfatal estimation, please see Ong et al^3^ and section 6 below. Briefly, data sources were identified through performing PubMed systematic reviews, searching the Global Health Data Exchange (GHDx; <https://ghdx.healthdata.org/>) for updated rounds of known multi-year surveys that meet inclusion criteria, and working with members of the GBD Collaborator Network^4^ with interest in diabetes for access to eligible data sources.
   b. For this analysis, literature sources in the GBD diabetes mellitus model tagged as having data available on diabetes treatment were reviewed for inclusion. Individual-level data with variables on FPG or HbA1c and diabetes treatment usage (insulin or other hypoglycemic medication) were included in this analysis.
2. GHDx opportunistic search
   a. An additional opportunistic search of the GHDx was undertaken in September 2024 to identify updated rounds of known multi-year surveys since GBD data seeking for which we have access to individual-level data. Reports for STEPS surveys conducted post-2020 for which we do not yet have access to individual-level data were also assessed.

Inclusion and exclusion criteria were the same as detailed in the systematic review section above. A full list of data inputs with citations and additional information can be found in section 8 below.

## Section 2.2. Data coverage

Data coverage is reflected in the figures below. Sources are counted in “source-years” at the country-level, meaning each unique combination of source and year for a given country is counted as one source-year. For example, a source that provides data for Chile for 2010 and for 2013 is counted as two source-years.

***Figure S2. Data inputs map – all models combined***

***Figure S3. Data inputs map – undiagnosed proportion among those with diabetes model***

***Figure S4. Data inputs map –diagnosed and untreated proportion among those with diabetes model***

***Figure S5. Data inputs map –diagnosed and treated proportion among those with diabetes model***

***Figure S6. Data inputs map –treated with suboptimal glycaemic levels proportion among those with diabetes model***

***Figure S7. Data inputs map –treated with optimal glycaemic levels proportion among those with diabetes model***

***Figure S8. Data inputs by study start year and Global Burden of Disease super-region – all models combined***

# Section 3. Data processing

## Section 3.1. Microdata aggregation

Prior to microdata aggregation, the following processing steps were taken:

1. FPG values reported in mg/dL were standardised to mmol/L by dividing by 18
2. Datasets were restricted to only persons who completed a FPG or HbA1c test and were 15 years or older
3. Datasets were restricted to persons without extreme FPG or HbA1c values - defined as a FPG less than 2.5 mmol/L or greater than 25 mmol/L, or a HbA1c less than 2% or greater than 20%. These thresholds are used in GBD 2021 for diabetes microdata processing and were determined based on population-level FPG and HbA1c distributions.^3^
4. If the survey collected data on both self-report diagnosis and treatment and an individual’s value was missing or unknown, we made the following assumptions: a negative self-report of prior diagnosis indicated the individual was not on treatment; a positive self-report of being on treatment indicated the individual had a positive prior diagnosis.

Aggregations of the microdata used available sample weighting and were carried out per study, year, sex, and 10- or 20-year age bin, depending on sample size. Sources where 20% or more datapoints had sample size of 10 persons or less upon aggregation with 10-year age bins where instead aggregated with 20-year age bins to increase sample size. The denominator for all aggregations was the number of people with diabetes, defined as FPG greater than or equal to 7 mmol/L, or on treatment (insulin or other hypoglycemic medication); or HbA1c greater than or equal to 6.5%, or on treatment (insulin or other hypoglycemic medication). The numerator was calculated for each of the modelling components as described in table S2 above and shown in the flowchart below. The threshold for suboptimal/optimal glycaemic levels was FPG 7.2 mmol/L. For microdata sources were HbA1c values were used for aggregation rather than FPG, the threshold for suboptimal/optimal glycaemic levels was HbA1c 7%; these data were then adjusted to reflect values estimated to have been present if a threshold of FPG 7.2 mmol/L had been used, as described in section 3.3 below.

***Figure S9. Microdata aggregation flowchart***

All microdata extraction and processing were conducted in R version 4.4.1.

## Section 3.2. Sex and age splitting

Tabulated data that we do not have microdata access for may be reported for both sexes combined, or for a broad age range. Prior to modelling, we disaggregated these data into sex- and age-specific datapoints:

1. Within-study age-sex splitting: First, if studies reported proportions for broad age groups by sex, and also separately by specific age groups but for both sexes combined, a sex ratio was calculated from the sex-specific data. This within-study sex ratio was used to disaggregate the age-specific both sex data into sex- and age-specific data.
2. Sex splitting: Available sex-specific data for a given analysis component were used as inputs in the meta-regression Bayesian, regularised, trimmed (MR-BRT) tool^5^ to produce a global sex ratio by age. This ratio was used to disaggregate remaining both sex data into sex-specific data, taking into account the sex distribution of the diabetes population in the location, year, and age of the both sex datapoints. Uncertainty from the original both sex data and the MR-BRT sex ratio was propagated forward to the new sex-specific data.
3. Age splitting: Finally, available data for a given analysis component with age range less than 25 years and sample size greater than 30 persons were used as inputs in DisMod-MR 2.1,^6^ a hierarchical Bayesian meta-regression modelling tool to produce an age pattern by sex and super-region. These age patterns were used to disaggregate the remaining data with age range spanning more than 25 years into five-year age groups, taking into account the age distribution of the diabetes population in the location, year, and sex of the broad age range datapoints. Uncertainty from the original broad age range data and the DisMod-MR 2.1 age pattern was propagated forward to the new age-specific data.

The below table details the number of datapoints involved in each sex and age splitting step, for each model.

***Table S3. Number of processed datapoints by model – sex and age splitting***

| **Model** | **Within-study age-sex split: input** | **Within-study age-sex split: output** | **Sex split: MR-BRT sex ratio input** | **Sex split: input** | **Sex split: output** | **Age split: DisMod-MR 2.1 age pattern input** | **Age split: input** | **Age split: output** |
| --- | --- | --- | --- | --- | --- | --- | --- | --- |
| Undiagnosed | 20 | 28 | 616 | 12 | 24 | 863 | 44 | 512 |
| Diagnosed, untreated | 6 | 8 | 435 | 10 | 20 | 827 | 30 | 402 |
| Diagnosed, treated | 17 | 22 | 648 | 16 | 32 | 924 | 59 | 670 |
| Diagnosed, treated, suboptimal glycaemic levels | 12 | 16 | 592 | 8 | 16 | 896 | 30 | 390 |
| Diagnosed, treated, optimal glycaemic levels | 18 | 24 | 580 | 9 | 18 | 910 | 36 | 450 |

## Section 3.3. Glycaemic management threshold adjustment

The reference threshold for suboptimal/optimal glycaemic management in this analysis is FPG 7.2 mmol/L. In cases where we aggregated microdata sources using HbA1c 7% as the threshold, or where tabulated data in published literature reported the proportions using different thresholds, we adjusted these data to reflect values estimated to have been present if a threshold of FPG 7.2 mmol/L had been used. Adjustment coefficients for each adjustment were calculated using the MR-BRT tool^6^, where inputs were ratios constructed between alternative case definitions and the reference case definition using microdata from surveys that measured both FPG and HbA1c among participants. We performed this analysis in logit-space.

There were four alternative definitions for the treated with suboptimal glycaemic levels proportion, i.e. a threshold of FPG greater than or equal to 5.6 or 7 mmol/L, and HbA1c greater than or equal to 6.5% or 7%. There were five alternative definitions for the treated with optimal glycaemic levels proportion, i.e. a threshold of FPG less than 5.6, 7, or 7.22 mmol/L, and HbA1c less than 6.5% or 7%.

The below table details the number of datapoints involved in each suboptimal/optimal glycaemic management threshold adjustment.

***Table S4. Number of processed datapoints by model – suboptimal/optimal glycaemic management threshold adjustment***

| **Model** | **Alternative threshold** | **Adjusted datapoints** |
| --- | --- | --- |
| Diagnosed and treated with suboptimal glycaemic levels proportion | FPG ≥ 5.6 mmol/L | 6 |
|  | FPG ≥ 7 mmol/L | 71 |
|  | HbA1c ≥ 6.5% | 110 |
|  | HbA1c ≥ 7% | 230 |
| Diagnosed and treated with optimal glycaemic levels proportion | FPG < 5.6 mmol/L | 6 |
|  | FPG < 7 mmol/L | 96 |
|  | FPG < 7.22 mmol/L | 8 |
|  | HbA1c < 6.5% | 108 |
|  | HbA1c < 7% | 215 |

The below figure shows the adjustment betas used for each suboptimal/optimal glycaemic management threshold adjustment.

***Figure S10. MR-BRT adjustment factors for suboptimal/optimal glycaemic management threshold adjustment***

## Section 3.4. Outliering

Prior to the above data processing steps and modelling, data with sample size less than or equal to 10 persons were outliered such that they did not influence any processing step or model.

# Section 4. Modelling and post-processing

## Section 4.1. DisMod-MR 2.1 modelling

Five separate models for the undiagnosed proportion, the untreated diagnosed proportion, the diagnosed and treated proportion, the treated with suboptimal glycaemic levels proportion, and the treated with optimal glycaemic levels proportion were run using DisMod-MR 2.1.^6^ In each model, the time window for fit was set to 10 years, meaning that the model took into account data 10 years before and 10 years after the given estimation year.

In each model, mean body-mass index (BMI) and the Healthcare Access and Quality Index (HAQ Index), which uses a select number of causes of amenable mortality to assess healthcare access and quality in a comparable manner across countries,^7^ were used as covariates. Below is a table of the covariate betas for each model.

***Table S5. DisMod-MR 2.1 model covariates***

| **Model** | **Covariate** | **Type** | **Parameter** | **Exponentiated beta (95% Uncertainty Interval)** |
| --- | --- | --- | --- | --- |
| Undiagnosed proportion | Healthcare access and quality index | Country-level | Proportion | 0.99 (0.99 – 0.99) |
|  | Mean body-mass index | Country-level | Proportion | 1.00 (0.98 – 1.01) |
| Diagnosed and untreated proportion | Healthcare access and quality index | Country-level | Proportion | 0.96 (0.96 – 0.97) |
|  | Mean body-mass index | Country-level | Proportion | 1.12 (1.07 – 1.16) |
| Diagnosed and treated proportion | Healthcare access and quality index | Country-level | Proportion | 1.02 (1.02 – 1.02) |
|  | Mean body-mass index | Country-level | Proportion | 0.97 (0.95 – 0.99) |
| Diagnosed and treated with suboptimal glycaemic levels proportion | Healthcare access and quality index | Country-level | Proportion | 1.02 (1.02 – 1.02) |
|  | Mean body-mass index | Country-level | Proportion | 0.99 (0.97 – 1.01) |
| Diagnosed and treated with optimal glycaemic levels proportion | Healthcare access and quality index | Country-level | Proportion | 1.02 (1.02 – 1.03) |
|  | Mean body-mass index | Country-level | Proportion | 1.02 (0.99 – 1.05) |

## Section 4.2. Post-processing

After modelling, we sampled 250 random draws from each age-, sex-, location-, and year-specific parameter distribution from DisMod-MR. All post-processing steps detailed below were conducted at the draw level and by age, sex, location, and year.

First, estimates from the undiagnosed proportion and the untreated diagnosed proportion models were scaled by age group, sex, location and year to sum to the untreated proportion, calculated as the inverse of the treated proportion. Similarly, estimates from the treated with suboptimal glycaemic levels proportion and the treated with optimal glycaemic levels proportion models were scaled to sum to the treated proportion. Thus, the scaled undiagnosed proportions, untreated diagnosed proportions, treated with suboptimal glycaemic levels proportions, and treated with optimal glycaemic levels proportions summed to 100% of people with diabetes by age group, sex, location, and year.

After scaling, proportions for each analysis component were converted to counts by multiplying each scaled proportion by the corresponding population with diabetes reported by GBD 2023. Tables of GBD 2023 diabetes cases counts and citations can be found in section 8 below. Counts of diagnosed diabetes were calculated by subtracting the estimated undiagnosed count from the corresponding total population with diabetes. Cascade estimates for proportion diagnosed among those with diabetes, proportion treated among those with diagnosed diabetes, and proportion with optimal glycaemic levels among those with treated diabetes were then calculated by redividing numerator counts by the desired denominator counts. Finally, estimates for all age groups over age 15 were calculated by summing counts across age groups and redividing by the population with diabetes ages 15 and above per sex, location, and year. Calculations for other custom age groupings were calculated similarly.

After post-processing, 95% uncertainty intervals for estimates were calculated by taking the 2.5th and 97.5th percentile values across the 250 draws.

All post-processing steps were conducted in R version 4.4.1.

# Section 5. Sensitivity analysis

A sensitivity analysis was conducted using a FPG value of 8.6 mmol/L as the threshold for optimal glycaemic levels. We determined this to be the best estimate in available cross-sectional survey data of a FPG value most similar to a HbA1c value of 8%, the threshold used by the WHO Global Diabetes Compact for their optimal glycaemic levels target,^8,9^ based on a linear regression with individual-level data that measured both FPG and HbA1c among participants as inputs. There were approximately 162,000 observations from 40 studies informing the linear regression. Below is the linear regression equation:

$$FPG= -1.557+1.265A1c$$

***Figure S11. FPG vs. HbA1c linear regression, sensitivity analysis***

The R^2^ value was 0.619.

The data processing steps for the sensitivity analysis were followed as described in section 3 above, with the exception of collapsing the microdata and adjusting alternative definitions to reflect FPG 8.6 mmol/L as the threshold. For the sensitivity analysis, there were six alternative definitions for the treated with suboptimal glycaemic levels proportion, i.e. a threshold of FPG greater than or equal to 5.6, 7, or 7.2 mmol/L, and HbA1c greater than or equal to 6.5%, 7%, or 8%. There were seven alternative definitions for the treated with optimal glycaemic levels proportion, i.e. a threshold of FPG less than 5.6, 7, 7.2, or 7.22 mmol/L, and HbA1c less than 6.5%, 7%, or 8%.

The below table details the number of datapoints involved in each suboptimal/optimal glycaemic management threshold adjustment for the sensitivity analysis.

***Table S6. Number of processed datapoints by model – suboptimal/optimal glycaemic management threshold adjustment, sensitivity analysis***

| **Model** | **Alternative threshold** | **Adjusted datapoints** |
| --- | --- | --- |
| Diagnosed and treated with suboptimal glycaemic levels proportion | FPG ≥ 5.6 mmol/L | 6 |
|  | FPG ≥ 7 mmol/L | 71 |
|  | FPG ≥ 7.2 mmol/L | 2 |
|  | HbA1c ≥ 6.5% | 110 |
|  | HbA1c ≥ 7% | 8 |
|  | HbA1c ≥ 8% | 218 |
| Diagnosed and treated with optimal glycaemic levels proportion | FPG < 5.6 mmol/L | 6 |
|  | FPG < 7 mmol/L | 96 |
|  | FPG < 7.2 mmol/L | 2 |
|  | FPG < 7.22 mmol/L | 8 |
|  | HbA1c < 6.5% | 108 |
|  | HbA1c < 7% | 8 |
|  | HbA1c < 8% | 218 |

The below figure shows the adjustment betas used for each suboptimal/optimal glycaemic management threshold adjustment for the sensitivity analysis.

***Figure S12. MR-BRT adjustment factors for suboptimal/optimal glycaemic management threshold adjustment, sensitivity analysis***

Updated DisMod-MR 2.1 models for the treated with suboptimal glycaemic levels proportion and the treated with optimal glycaemic levels proportion were run with data reflecting the new threshold. The model parameters were the same as described in section 4 above. Below is a table of the covariate betas for each model.

***Table S7. DisMod-MR 2.1 model covariates, sensitivity analysis***

| **Model** | **Covariate** | **Type** | **Parameter** | **Exponentiated beta (95% Uncertainty Interval)** |
| --- | --- | --- | --- | --- |
| Diagnosed and treated with suboptimal glycaemic levels proportion | Healthcare access and quality index | Country-level | Proportion | 1.02 (1.02 – 1.02) |
|  | Mean body-mass index | Country-level | Proportion | 0.97 (0.94 – 0.99) |
| Diagnosed and treated with optimal glycaemic levels proportion | Healthcare access and quality index | Country-level | Proportion | 1.02 (1.02 – 1.03) |
|  | Mean body-mass index | Country-level | Proportion | 1.03 (1.00 – 1.06) |

Post-processing for the sensitivity analysis follows the same steps as described in section 4 above.

# Section 6. GBD 2023 diabetes prevalence estimation

## Section 6.1. Case definitions

Reference and alternative case definitions and diagnostic criteria for diabetes mellitus prevalence estimation in GBD 2023 are presented in the table below.

***Table S8. Case definitions for GBD 2023 diabetes mellitus prevalence***

| **Quantity of interest** | **Reference or Alternative** | **Definition** |
| --- | --- | --- |
| Diabetes mellitus | Reference | Fasting plasma glucose (FPG) greater or equal to 126 mg/dl (7 mmol/L) or current treatment (insulin or drugs) |
| Diabetes mellitus | Alternative | FPG greater than a threshold not equal to 126 mg/dl (7mmol/L) or current treatment (insulin or drugs) |
| Diabetes mellitus | Alternative | Blood sugar tests measured from glycated haemoglobin (HbA1c) or current treatment (insulin or drugs) |
| Diabetes mellitus | Alternative | Blood sugar tests measured from oral glucose tolerance test (OGTT) or current treatment (insulin or drugs) |
| Diabetes mellitus | Alternative | Blood sugar tests measured from post-prandial glucose test (PPG) or current treatment (insulin or drugs) |
| Diabetes mellitus | Alternative | Combination of non-FPG blood sugar test(s) and FPG or current treatment (insulin or drugs) |
| Diabetes mellitus | Alternative | Blood sugar tests measured from FPG, HbA1c, OGTT, or PPG (no treatment) |

## Section 6.2. Data inputs and seeking

To incorporate all available population-representative data of diabetes, we accepted other measures of blood sugar (glycated haemoglobin A1c, oral glucose tolerance test, post-prandial glucose test) in addition to fasting plasma glucose to define diabetes. Studies that used random plasma glucose to define diabetes or self-reporting of diabetes status were not accepted.

Data inputs came from four types of sources:

- Estimates of diabetes in a representative population
- Estimates of mean FPG in a representative population
- Individual-level data of blood sugar from surveys
- Clinical data inclusive of inpatient and outpatient data from US, Poland, Republic of Korea and Mongolia

 When a study reported both mean FPG and prevalence of diabetes, we used the prevalence of diabetes. Where possible, individual-level data from a cohort superseded any data described in a published paper. Individual-level data were collapsed and aggregated to produce estimates for each age group, sex, location, and year a survey was conducted.

A total of 96 new sources were included in the overall diabetes mellitus model for GBD 2023. Collaborator-provided sources that were either shared directly with us or were identified through searching the Global Health Data Exchange (GHDx) were reviewed for inclusion. An opportunistic search was conducted to identify scientific literature that reported on mortality-related measures, including relative risk of mortality, standard mortality ratio and with-condition mortality rate for overall diabetes. A total of 39 sources were used in the overall diabetes model. Additionally, clinical data prepared by the clinical informatics team, that are representative of the sum of inpatient and outpatient visits from USA, Poland and Republic of Korea and Mongolia were included. Data from US Medicare, Poland patient claims data, Korea national health insurance services data, and Mongolia H-info health systems data are all new additions to GBD 2023.

An updated systematic review was conducted for GBD 2023. The search took place for the following dates: 10/17/2018-2/11/2022. Below is the PubMed search string:

(((diabetes[TI] AND (prevalence[TIAB] OR incidence[TIAB])) OR ('Diabetes Mellitus'[MeSH Terms] AND 'epidemiology'[MeSH Terms]) OR (diabetes[TI] AND 'epidemiology'[MeSH Terms]) NOT gestational[All Fields] NOT ('neoplasms'[MeSH Terms] OR 'neoplasms'[All Fields] OR 'cancer'[All Fields]) NOT ('mice'[MeSH Terms] OR 'mice'[All Fields]) NOT ('schizophrenia'[MeSH Terms] OR 'schizophrenia'[All Fields]) NOT ('emigrants and immigrants'[MeSH Terms] OR ('emigrants'[All Fields] AND 'immigrants'[All Fields]) OR 'emigrants and immigrants'[All Fields] OR 'immigrants'[All Fields]) NOT ('pregnancy'[MeSH Terms] OR 'pregnancy'[All Fields] OR 'gestation'[All Fields]) NOT ('rats'[MeSH Terms] OR 'rats'[All Fields] OR 'rat'[All Fields]) NOT ('kidney'[MeSH Terms] OR 'kidney'[All Fields]) NOT renal[All Fields] NOT ('vitamins'[Pharmacological Action] OR 'vitamins'[MeSH Terms] OR 'vitamins'[All Fields] OR 'vitamin'[All Fields])) OR (((“glucose”[Mesh] OR “hyperglycemia”[Mesh] OR “prediabetic state”[Mesh]) AND "Geographic Locations"[Mesh] NOT "United States"[Mesh]) AND ("humans"[Mesh] AND "adult"[MeSH]) AND ("Data Collection"[Mesh] OR "Health Services Research"[Mesh] OR "Population Surveillance"[Mesh] OR "Vital statistics"[Mesh] OR "Population"[Mesh] OR "Epidemiology"[Mesh] OR surve*[TiAb]) NOT Comment[ptyp] NOT Case Reports[ptyp] NOT "hospital"[TiAb])) AND (("2018/10/17"[Date - Publication] : "3000"[Date - Publication]))

***Figure S13. PRISMA diagram for GBD 2023 diabetes prevalence***

## Section 6.3. Data processing

We performed several processing steps to the data to address sampling and measurement inconsistencies that will ensure the data are comparable across data sources and between high fasting plasma glucose modelling efforts.

1. *Small sample size and null prevalence*: Data with a sample size of 10 or less or if the source’s mean prevalence was reported to be 0 were outliered prior to modelling.
2. *Mean FPG processing*: We used an ensemble distribution to estimate the prevalence of diabetes based on mean FPG for sources where data on prevalence of diabetes were not available but there was data on mean FPG. Essentially, we constructed a distribution based on unit-level data available in 31 different countries. Then we predicted out the prevalence of diabetes by age and sex. This provides the conversion of mean FPG to prevalence of diabetes defined as FPG greater than or equal to 126 mg/dL (7 mmol/L). Because this definition is not consistent with our reference case definition (which also includes those on treatment), we then adjust these datapoints to the reference case definition. For information on how these adjustments are made, please see the section, “Alternative definition bias adjustments” below.
3. *Sex splitting*: In GBD 2023, reported estimates of prevalence were split by sex and where possible. Studies with broad age groups estimates were no longer age-split given minimal impact on age-specific model estimates after testing models with and without age-splitting. First, if studies reported prevalence for broad age groups by sex and by specific age groups but for both sexes combined, age-specific estimates were split by sex using the sex ratio from within the study. Then, input data reporting prevalence for both sexes that could not be split using a within-study ratio were split using a sex ratio derived from a meta-analysis of existing sex-specific data (excluding studies already split using within-study sex ratios) using MR-BRT.^5^ Age-specific female to male ratio for diabetes prevalence is shown in the figure below. Additionally, incidence data for diabetes were separately split using an all-age female to male ratio of 0.68 (95% CI: 0.60-0.77).

***Figure S14. Prevalence of GBD 2023 diabetes female to male sex ratio***

4. *Alternative definition bias adjustments:* We adjusted estimates from alternative case definitions to the reference case definition. Ratios were constructed between alternative case definitions and the reference case definition using microdata from surveys that measured glucose level based on different glucose tests on a single. We used MR-BRT analysis and performed this analysis in logit space due to the high prevalence of diabetes to ensure post-adjustment prevalence values do not exceed 1. Detailed steps are outlined below and adjustment factors for each alternative definition are outlined in the table below.
5. *Clinical data bias adjustments:* Clinical data often underestimate prevalence due to not being able to capture individuals who do not seek care and/or are undiagnosed. We examined and adjusted for clinical data bias to ensure population representation by comparing these data to population representative population surveys from the same country.  However, we assume that claims data in persons <15 years are type 1 diabetes and that 100% of people with diabetes are captured in this age group. Thus, we only consider adjustments of the claims data for reported estimates in persons >15 years.  When we compared US Medicare data to US NHANES data and Republic of Korea clinical data to Korea NHANES data, we found that estimates were similar, therefore these two sources did not undergo any bias adjustment. We were not able to include Mongolia claims data for ages >15 this round. This is because there was not sufficient reference data to produce a reliable adjustment factor and we believe the claims data is heavily biased if no adjustments were applied. For Poland claims data, a sex covariate was included to account for differences in the adjustment factor by sex upon observing differences by sex. There were a total of 5 years of Poland claims data from the years 2015-2019, all 5 surveys used the Poland lipidogram study from 2015-2016 as reference given the rather stable estimates of claims data over the 5 year period. Detailed steps are outlined below and adjustment factors for each clinical data source are outlined in the table below. Additionally, given small sample sizes and minimal representation in population surveys of people over the age of 85, we were not able to confidently apply bias adjustment factors for clinical data from this age group and therefore did not include clinical data in ages 85 and above in the model.

The process of adjusting for non-reference or clinical data using MR-BRT with the logit-transformation method is described below:

1. Identify datapoints with overlapping year, age, sex, and location between alternative case definition and reference case definition
2. Logit transform overlapping datapoints of alternative and reference case definitions
3. Convert overlapping datapoints into a difference in logit space using the following equation:

logit(alternative) – logit(reference)

4. Use the delta method to compute standard errors of overlapping datapoints in logit space, then calculate standard error of logit difference using the following equation:

√((variance of alternative) + (variance of reference))

5. Using MR-BRT, conduct a random effects meta-regression to obtain the pooled logit difference of alternative to reference
6. Apply the pooled logit difference to all datapoints of alternative case definitions using the following equation:

New estimate = inverse.logit((logit(alternative)) –  (pooled logit difference))

7. Calculate new standard errors using the delta method, accounting for gamma (between-study heterogeneity)

***Table S9. MR-BRT adjustment factors for GBD 2023 diabetes prevalence alternative case definitions***

| **Case definition**  (fpg = fasting plasma glucose mmol/L;  ogtt = oral glucose tolerance test mmol/L;  hbA1c= hemoglobin A1c %) | **Reference or alternative case definition** | **Gamma** | **Beta Coefficient Log/Logit (95% UI) *** | **Adjustment factor **** |
| --- | --- | --- | --- | --- |
| fpg >=7mmol/L or on treatment | Ref | --- | --- | --- |
| fpg >=5.5 or on treatment | Alt | 0.27 | 1.62 (1.6 to 1.65) | 5.05 (4.95 to 5.21) |
| fpg >=5.6 | Alt | 0.25 | 1.34 (1.32 to 1.37) | 3.82 (3.74 to 3.94) |
| fpg >=5.6 ogtt >=11.1 or on treatment | Alt | 0.63 | 1.88 (1.75 to 2.02) | 6.55 (5.75 to 7.54) |
| fpg >=5.6 or on treatment | Alt | 0.2 | 1.4 (1.38 to 1.42) | 4.06 (3.97 to 4.14) |
| fpg >=6.1 | Alt | 0.06 | 0.59 (0.57 to 0.6) | 1.8 (1.77 to 1.82) |
| fpg >=6.1o r ogtt >=11.1 or on treatment | Alt | 0.25 | 0.89 (0.79 to 0.99) | 2.44 (2.2 to 2.69) |
| fpg >=6.1 or ogtt >=7.8 or on treatment | Alt | 0.1 | 1.63 (1.55 to 1.71) | 5.1 (4.71 to 5.53) |
| fpg >=6.1 or on treatment | Alt | 0.03 | 0.69 (0.67 to 0.7) | 1.99 (1.95 to 2.01) |
| Fpg >=6.7 | Alt | 0 | -0.01 (-0.02 to 0) | 0.99 (0.98 to 1.00) |
| fpg >=6.7 or ogtt >=10 | Alt | 0.3 | 0.5 (0.39 to 0.61) | 1.65 (1.48 to 1.84) |
| fpg >=6.7 or ogtt >=11.1 | Alt | 0.36 | 0.27 (0.15 to 0.38) | 1.31 (1.16 to 1.46) |
| fpg >=6.9 or ogtt >=11.1 or on treatment | Alt | 0.08 | 0.09 (0.01 to 0.17) | 1.09 (1.01 to 1.19) |
| fpg >=7 | Alt | 0 | -0.22 (-0.23 to -0.21) | 0.8 (0.79 to 0.81) |
| fpg >=7 or hba1c >=6.4 or on treatment | Alt | 0.03 | 0.27 (0.24 to 0.31) | 1.31 (1.27 to 1.36) |
| fpg >=7 or hba1c >=6.5 or on treatment | Alt | 0.03 | 0.22 (0.18 to 0.25) | 1.25 (1.2 to 1.28) |
| fpg >=7 or ogtt >=11 or hba1c >=6.5 or on treatment | Alt | 0 | -0.19 (-0.29 to -0.09) | 0.83 (0.75 to 0.91) |
| fpg >=7 or ogtt >=11 or on treatment | Alt | 0.06 | 0.06 (-0.02 to 0.14) | 1.06 (0.98 to 1.15) |
| fpg >=7 or ogtt >=11.1 | Alt | 0.08 | 0.02 (-0.06 to 0.1) | 1.02 (0.94 to 1.11) |
| fpg >=7 or ogtt >=11.1 or hba1c >=6.5 | Alt | 0 | -0.21 (-0.3 to -0.11) | 0.81 (0.74 to 0.90) |
| fpg >=7 or ogtt >=11.1 or hba1c >=6.5 or on treatment | Alt | 0 | -0.21 (-0.31 to -0.11) | 0.81 (0.73 to 0.90) |
| fpg >=7 or ogtt >=11.1 or hba1c >=6.9 or on treatment | Alt | 0 | -0.31 (-0.41 to -0.21) | 0.73 (0.66 to 0.81) |
| fpg >=7 or ogtt >=11.1 or on treatment | Alt | 0.07 | 0.04 (-0.04 to 0.12) | 1.04 (0.96 to 1.13) |
| fpg >=7 or ogtt >=12.2 | Alt | 0.11 | -0.18 (-0.27 to -0.09) | 0.84 (0.76 to 0.91) |
| fpg >=7 or ogtt >=12.2 or on treatment | Alt | 0.1 | -0.15 (-0.24 to -0.06) | 0.86 (0.79 to 0.94) |
| fpg >=7.8 | Alt | 0.01 | -0.66 (-0.68 to -0.65) | 0.52 (0.51 to 0.52) |
| fpg >=7.8 or ogtt >=11.1 | Alt | 0.03 | -0.22 (-0.3 to -0.15) | 0.8 (0.74 to 0.86) |
| fpg >=7.8 or ogtt >=11.1 or on treatment | Alt | 0.01 | -0.2 (-0.27 to -0.13) | 0.82 (0.76 to 0.88) |
| fpg >=7.8 or on treatment | Alt | 0 | -0.29 (-0.3 to -0.27) | 0.75 (0.74 to 0.76) |
| fpg >=8 or ogtt >=11.1 | Alt | 0.03 | -0.24 (-0.31 to -0.16) | 0.79 (0.73 to 0.85) |
| fpg >=8 or ogtt >=11.1 or on treatment | Alt | 0.01 | -0.21 (-0.28 to -0.13) | 0.81 (0.76 to 0.88) |
| fpg >=9.2 or on treatment | Alt | 0.01 | -0.48 (-0.5 to -0.47) | 0.62 (0.61 to 0.63) |
| fpg >=10 or ogtt >=11.1 or on treatment | Alt | 0.01 | -0.27 (-0.34 to -0.19) | 0.76 (0.71 to 0.83) |
| fpg >=11.1 or ogtt >=11.1 | Alt | 0.01 | -0.3 (-0.38 to -0.22) | 0.74 (0.68 to 0.8) |
| fpg >=11.1 or ogtt >=11.1 or on treatment | Alt | 0.01 | -0.27 (-0.35 to -0.2) | 0.76 (0.7 to 0.82) |
| hba1c >=6 or on treatment | Alt | 0.11 | 0.74 (0.7 to 0.78) | 2.1 (2.01 to 2.18) |
| hba1c >=6.5 | Alt | 0.11 | -0.36 (-0.4 to -0.32) | 0.7 (0.67 to 0.73) |
| hba1c >=6.5 or on treatment | Alt | 0.09 | -0.1 (-0.13 to -0.07) | 0.9 (0.88 to 0.93) |
| ogtt >=10 | Alt | 0 | 0.51 (0.31 to 0.7) | 1.67 (1.36 to 2.01) |
| ogtt >=10 or on treatment | Alt | 0 | 0.54 (0.35 to 0.74) | 1.72 (1.42 to 2.1) |
| ogtt >=11.1 | Alt | 0 | 0.06 (-0.16 to 0.27) | 1.06 (0.85 to 1.31) |
| ogtt >=11.1 or on treatment | Alt | 0 | 0.11 (-0.1 to 0.32) | 1.12 (0.9 to 1.38) |

***Table S10. MR-BRT adjustment factors for GBD 2023 diabetes prevalence clinical data***

| **Data input** | **Reference or alternative case definition** | **Gamma** | **Beta Coefficient Log/Logit (95% UI) *** | **Adjustment factor **** |
| --- | --- | --- | --- | --- |
| US NHANES 2001-2002 | Ref | 0 | --- | --- |
| US  Marketscan 2000 | Alt |  | -0.71 (-0.9 to -0.51) | 0.49 (0.41 to 0.6) |
| US NHANES 2009-2010 | Ref | 0 | --- | --- |
| US  Marketscan 2010 | Alt |  | -0.48 (-0.65 to -0.31) | 0.62 (0.52 to 0.73) |
| US NHANES 2011-2012 | Ref | 0 |  |  |
| US  Marketscan 2011 | Alt |  | -0.47 (-0.65 to -0.29) | 0.63 (0.52 to 0.75) |
| US NHANES 2011-2012 | Ref | 0 |  |  |
| US Marketscan 2012 | Alt |  | -0.46 (-0.65 to -0.28) | 0.63 (0.52 to 0.76) |
| US NHANES 2013-2014 | Ref | 0 | --- | --- |
| US  Marketscan 2013 | Alt |  | -0.48 (-0.65 to -0.31) | 0.62 (0.52 to 0.73) |
| US NHANES 2013-2014 | Ref | 0 | --- | --- |
| US  Marketscan 2014 | Alt |  | -0.44 (-0.61 to -0.27) | 0.64 (0.54 to 0.76) |
| US NHANES 2015-2016 | Ref | 0 | --- | --- |
| US  Marketscan 2015 | Alt |  | -0.48 (-0.65 to -0.3) | 0.62 (0.52 to 0.74) |
| US NHANES 2015-2016 | Ref | 0 | --- | --- |
| US  Marketscan 2016 | Alt |  | -0.49 (-0.66 to -0.32) | 0.61 (0.52 to 0.73) |
| US NHANES 2017-2020 | Ref | 0 | --- | --- |
| US  Marketscan 2017 | Alt |  | -0.59 (-0.75 to -0.43) | 0.55 (0.47 to 0.65) |
| US NHANES 2017-2020 | Ref | 0 | --- | --- |
| US  Marketscan 2018 | Alt |  | -0.67 (-0.82 to -0.51) | 0.51 (0.44 to 0.6) |
| US NHANES 2017-2020 | Ref | 0 | --- | --- |
| US  Marketscan 2019 | Alt |  | -0.64 (-0.8 to -0.48) | 0.53 (0.45 to 0.62) |
| Poland Lipidogram Study 2015-2016 | Ref | 0.05 |  |  |
| Poland Patient Claims Data 2015-2019 - Male | Alt |  | -1.00  (-1.15 to -0.84) | 0.37 (0.32 to 0.43) |
| Poland Patient Claims Data 2015-2019 - Female | Alt |  | -0.89 (-1.15 to -0.63) | 0.41 (0.32 to 0.53) |

***The adjustment factor column is the exponentiated beta coefficient. For log beta coefficients, this is the relative rate between the two case definitions. For logit beta coefficients, this is the relative odds between the two case definitions.*

## Section 6.4. Modelling and post-processing

No significant changes to the modelling strategy for diabetes prevalence were made in GBD 2023.

We estimated diabetes mellitus using DisMod-MR 2.1,^6^ which produces estimates of the prevalence of diabetes for each age, sex, geographical location, and year. We used data that reported prevalence and incidence for diabetes mellitus.

Model parameters and estimates

- We set a value prior of 0 for remission for ages 0 to 14
- We set a value prior of a maximum value of 0.01 for remission for ages 15 to 100
- We set a value prior of a maximum value of 0.15 for excess mortality for all ages
- We set a value prior of 0 for incidence for ages 0 to 1
- We set a value prior of a maximum value of 0.0008 for incidence for ages 1 to 15
- We set a value prior of a maximum value of 0.1 for incidence for ages 15 to 100

***Table S11. DisMod-MR 2.1 model covariates, GBD 2023 diabetes prevalence***

| Covariate | Type | Parameter | Exponentiated beta (95% uncertainty interval) |
| --- | --- | --- | --- |
| Prevalence of obesity (age-standardised) | Country-level | Prevalence | 10.81 (10.20–11.62) |
| Year | Country-level | Prevalence | 1.01 (1.01–1.01) |

# Section 7. References

1	Stevens GA, Alkema L, Black RE, *et al.* Guidelines for Accurate and Transparent Health Estimates Reporting: the GATHER statement. *The Lancet* 2016; **388**: e19–23.

2	Page MJ, McKenzie JE, Bossuyt PM, *et al.* The PRISMA 2020 statement: an updated guideline for reporting systematic reviews. *BMJ* 2021; **372**: n71.

3	Ong KL, Stafford LK, McLaughlin SA, *et al.* Global, regional, and national burden of diabetes from 1990 to 2021, with projections of prevalence to 2050: a systematic analysis for the Global Burden of Disease Study 2021. *The Lancet* 2023; **0**. DOI:10.1016/S0140-6736(23)01301-6.

4	GBD Collaborator Network | Institute for Health Metrics and Evaluation. https://www.healthdata.org/research-analysis/gbd/collaborator-network (accessed Sept 5, 2024).

5	Zheng P, Barber R, Sorensen RJD, Murray CJL, Aravkin AY. Trimmed Constrained Mixed Effects Models: Formulations and Algorithms. *Journal of Computational and Graphical Statistics* 2021; **30**: 544–56.

6	Ferrari AJ, Santomauro DF, Aali A, *et al.* Global incidence, prevalence, years lived with disability (YLDs), disability-adjusted life-years (DALYs), and healthy life expectancy (HALE) for 371 diseases and injuries in 204 countries and territories and 811 subnational locations, 1990–2021: a systematic analysis for the Global Burden of Disease Study 2021. *The Lancet* 2024; **403**: 2133–61.

7	GBD 2016 Healthcare Access and Quality Collaborators. Measuring performance on the Healthcare Access and Quality Index for 195 countries and territories and selected subnational locations: a systematic analysis from the Global Burden of Disease Study 2016. *Lancet* 2018; **391**: 2236–71.

8	Gregg EW, Buckley J, Ali MK, *et al.* Improving health outcomes of people with diabetes: target setting for the WHO Global Diabetes Compact. *Lancet* 2023; **401**: 1302–12.

9	First-ever global coverage targets for diabetes adopted at the 75th World Health Assembly. https://www.who.int/news-room/feature-stories/detail/first-ever-global-coverage-targets-for-diabetes-adopted-at-the-75-th-world-health-assembly (accessed July 13, 2024).

# Section 8. Additional figures and tables

## Figure S15. Diabetes cascade of care proportions (A) and counts (B) in 2023 for both sexes over age, by Global Burden of Disease region

A. Proportion

B. Count

## Figure S16. Optimal glycaemic management proportion among people with diabetes, both sexes and ages 15 years and older in 2023

## Figure S17. Absolute change in percentage points in diabetes cascade of care proportions between 2000 and 2023 globally for both sexes, by age

## Figure S18. Absolute change in percentage points in diabetes cascade of care proportions between 2000 and 2023 for ages 15 and older and both sexes, by Global Burden of Disease region

## Table S12. Counts of diagnosed diabetes, treated diabetes, and diabetes with optimal glycaemic levels in 2023, and corresponding percent change between 2000 and 2023, for both sexes and ages 15 and older globally, and by Global Burden of Disease super-regions, regions, and countries and territories

|  | **Location** | **Diagnosed, 2023 (per 1,000)** | **Change in Diagnosed, 2000-2023 (%)** | **Treated, 2023 (per 1,000)** | **Change in Treated, 2000-2023 (%)** | **Optimal Glycaemic Management, 2023 (per 1,000)** | **Change in Optimal Glycaemic Management, 2000-2023 (%)** |  |
| --- | --- | --- | --- | --- | --- | --- | --- | --- |
|  | **Global** | **313000 (273000–358000)** | **142·3 (135·9–147·8)** | **286000 (241000–336000)** | **163·1 (157·6–168·3)** | **119000 (95200–145000)** | **171·7 (165·7–178·0)** |  |
|  | **Central Europe, eastern Europe, and central Asia** | **19200 (16400–22000)** | **102·4 (96·3–107·8)** | **17700 (14700–21000)** | **108·8 (100·7–116·0)** | **8930 (7170–10900)** | **125·3 (116·3–135·0)** |  |
|  | Central Asia | 2590 (2260–2950) | 162·2 (146·3–177·7) | 2290 (1980–2700) | 188·6 (166·1–209·0) | 968 (767–1190) | 195·2 (170·4–219·7) |  |
|  | Armenia | 127 (109–145) | 57·7 (39·1–73·3) | 116 (97·8–137) | 72·4 (50·4–93·7) | 47·2 (36·9–57·9) | 84·1 (58·0–106·9) |  |
|  | Azerbaijan | 423 (368–484) | 195·2 (162·5–225·4) | 397 (342–463) | 215·4 (172·5–255·1) | 116 (90·5–146) | 238·9 (186·1–298·1) |  |
|  | Georgia | 172 (152–193) | 50·5 (34·3–66·5) | 156 (136–181) | 57·4 (36·7–79·3) | 70·3 (57·4–83·9) | 62·3 (39·6–86·6) |  |
|  | Kazakhstan | 668 (574–771) | 143·9 (115·9–175·2) | 613 (520–721) | 185·3 (148·3–224·1) | 418 (346–500) | 192·9 (155·3–231·3) |  |
|  | Kyrgyzstan | 144 (123–170) | 194·9 (167·1–227·5) | 134 (113–161) | 221·1 (187·6–261·2) | 29·5 (22·1–38·1) | 242·3 (197·6–297·2) |  |
|  | Mongolia | 60·1 (51·5–70·7) | 219·7 (188·2–254·0) | 47·1 (38·6–57·2) | 283·7 (242·3–335·9) | 18·4 (14·5–23·3) | 295·0 (253·1–350·7) |  |
|  | Tajikistan | 163 (140–191) | 237·3 (203·9–273·2) | 105 (87·4–131) | 295·4 (257·7–339·1) | 24 (16–35·5) | 308·2 (250·4–363·6) |  |
|  | Turkmenistan | 68·8 (57·9–80·1) | 193·3 (163·9–224·6) | 56·2 (46·1–67·6) | 223·2 (187·3–257·9) | 14·3 (10·5–18·6) | 238·9 (194·6–288·7) |  |
|  | Uzbekistan | 768 (653–918) | 226·4 (197·5–259·3) | 670 (547–814) | 255·2 (221·9–293·5) | 239 (182–308) | 274·2 (227·7–326·9) |  |
|  | Central Europe | 7640 (6610–8790) | 64·5 (56·7–72·9) | 7030 (5710–8320) | 67·3 (59·0–76·4) | 3350 (2630–4100) | 70·0 (59·7–80·2) |  |
|  | Albania | 89·4 (73·2–107) | 137·1 (115·6–159·9) | 81·1 (64·2–100) | 148·6 (127·2–172·3) | 34·4 (25·5–43·7) | 164·8 (137·3–195·0) |  |
|  | Bosnia and Herzegovina | 224 (185–266) | 110·9 (87·3–131·2) | 199 (153–249) | 120·8 (94·3–143·9) | 84·6 (61·5–111) | 137·1 (107·0–162·7) |  |
|  | Bulgaria | 382 (318–451) | 33·7 (24·4–41·9) | 327 (259–409) | 36·5 (26·4–45·6) | 140 (104–185) | 44·3 (31·1–55·0) |  |
|  | Croatia | 348 (289–405) | 119·1 (100·1–137·0) | 325 (258–389) | 124·6 (104·0–148·2) | 143 (110–178) | 143·0 (118·4–166·6) |  |
|  | Czechia | 606 (504–720) | 155·3 (125·1–188·7) | 508 (402–645) | 172·1 (134·6–217·2) | 170 (121–229) | 214·9 (167·7–265·7) |  |
|  | Hungary | 468 (380–553) | 90·0 (72·6–105·7) | 419 (326–519) | 92·0 (73·8–110·9) | 184 (136–236) | 105·3 (82·8–128·7) |  |
|  | Montenegro | 48·5 (41·3–57·7) | 141·8 (123·4–164·1) | 44·2 (35·7–54·7) | 146·4 (124·5–171·4) | 19·3 (14·7–24·7) | 164·7 (139·8–192·1) |  |
|  | North Macedonia | 133 (112–157) | 119·6 (99·3–139·9) | 117 (91–146) | 131·6 (108·6–155·2) | 50·2 (36·6–65·6) | 147·2 (121·3–172·3) |  |
|  | Poland | 3630 (3120–4110) | 44·3 (33·6–54·9) | 3490 (2910–4030) | 45·8 (34·8–57·1) | 1850 (1500–2220) | 49·5 (37·0–61·6) |  |
|  | Romania | 752 (631–888) | 75·6 (56·6–95·0) | 662 (516–819) | 83·2 (63·2–103·1) | 283 (210–368) | 97·1 (75·3–121·3) |  |
|  | Serbia | 486 (418–579) | 60·9 (48·1–73·4) | 422 (341–527) | 70·2 (55·6–84·9) | 183 (139–241) | 83·5 (66·2–103·5) |  |
|  | Slovakia | 218 (186–258) | 102·9 (86·1–118·7) | 196 (160–242) | 110·4 (90·9–129·1) | 84·4 (65·8–107) | 123·0 (100·7–144·1) |  |
|  | Slovenia | 139 (117–159) | 121·5 (99·6–145·1) | 134 (109–156) | 128·8 (103·0–154·3) | 59·1 (45·5–72·2) | 147·7 (117·1–177·9) |  |
|  | Eastern Europe | 8990 (7570–10500) | 132·7 (123·0–142·7) | 8400 (6900–10100) | 140·6 (128·0–153·8) | 4640 (3740–5700) | 177·2 (161·9–193·6) |  |
|  | Belarus | 474 (423–527) | 106·5 (80·9–137·9) | 418 (369–480) | 145·4 (101·5–197·0) | 252 (211–296) | 149·3 (99·4–203·0) |  |
|  | Estonia | 74·8 (63·5–86·4) | 120·4 (94·6–145·7) | 70·5 (58·5–83·6) | 136·9 (108·4–168·0) | 35·6 (28·7–43·8) | 149·6 (119·1–186·5) |  |
|  | Latvia | 114 (97·4–133) | 115·1 (94·0–139·8) | 102 (84–124) | 123·7 (100·6–151·5) | 51·2 (39·6–64·3) | 135·0 (107·1–164·7) |  |
|  | Lithuania | 145 (122–171) | 159·0 (125·4–197·3) | 136 (109–163) | 168·0 (132·3–216·9) | 54·7 (41·7–68·9) | 186·0 (144·7–237·0) |  |
|  | Moldova | 170 (149–197) | 111·6 (90·1–131·5) | 135 (113–164) | 126·8 (95·0–155·9) | 61·4 (49–77·1) | 136·3 (100·1–172·3) |  |
|  | Russia | 6800 (5660–8100) | 171·0 (159·4–183·4) | 6550 (5380–7910) | 178·1 (163·9–193·2) | 3710 (2980–4550) | 228·8 (211·2–250·5) |  |
|  | Ukraine | 1200 (1000–1440) | 33·7 (26·1–42·6) | 985 (775–1250) | 26·5 (18·5–36·8) | 472 (360–598) | 31·2 (21·8–43·0) |  |
|  | **High income** | **79200 (70700–87300)** | **85·7 (80·7–91·1)** | **72600 (62100–82500)** | **91·6 (84·8–98·0)** | **32600 (26800–38300)** | **105·8 (97·5–113·5)** |  |
|  | Australasia | 1030 (893–1180) | 118·4 (101·5–139·2) | 887 (733–1070) | 133·8 (110·9–162·0) | 295 (221–376) | 164·6 (130·4–200·3) |  |
|  | Australia | 795 (679–919) | 104·9 (85·4–127·2) | 688 (560–837) | 119·1 (92·9–152·8) | 231 (171–296) | 149·3 (109·5–189·2) |  |
|  | New Zealand | 233 (206–264) | 182·9 (167·8–199·9) | 199 (164–237) | 205·7 (183·2–227·9) | 64·1 (48·2–81·1) | 240·9 (212·5–273·5) |  |
|  | High-income Asia Pacific | 14600 (12800–16500) | 63·0 (53·6–72·1) | 14100 (12300–16000) | 70·5 (58·8–81·5) | 7160 (6100–8320) | 87·1 (71·7–102·2) |  |
|  | Brunei | 29·1 (25·6–32·8) | 226·4 (194·8–261·6) | 26·5 (22·6–30·3) | 244·3 (202·4–299·4) | 10·1 (7·8–12·1) | 263·3 (213·1–321·7) |  |
|  | Japan | 10400 (8700–12200) | 39·4 (30·7–48·2) | 9990 (8250–11800) | 43·0 (32·5–53·4) | 5120 (4130–6230) | 58·9 (45·1–71·2) |  |
|  | South Korea | 3940 (3770–4100) | 175·9 (159·4–195·3) | 3810 (3640–3970) | 222·6 (200·2–248·7) | 1940 (1830–2060) | 238·3 (210·5–269·0) |  |
|  | Singapore | 278 (238–326) | 153·1 (123·9–190·2) | 267 (228–316) | 173·6 (139·0–214·8) | 97·2 (75·9–119) | 191·2 (151·0–237·7) |  |
|  | High-income North America | 35500 (31800–38800) | 133·0 (122·6–142·9) | 32800 (27900–37000) | 132·6 (120·4–143·2) | 15400 (12500–18100) | 148·8 (133·3–161·8) |  |
|  | Canada | 2080 (1860–2330) | 128·5 (108·7–149·9) | 2010 (1750–2270) | 129·7 (107·7–152·6) | 899 (730–1070) | 131·4 (107·7–153·7) |  |
|  | Greenland | 1·76 (1·5–2·03) | 133·9 (109·5–157·2) | 1·46 (1·14–1·8) | 166·1 (137·7–200·0) | 0·615 (0·464–0·775) | 172·9 (136·4–217·1) |  |
|  | USA | 33400 (29900–36500) | 133·3 (122·1–143·4) | 30800 (26100–34600) | 132·8 (120·2–144·2) | 14500 (11700–17100) | 150·0 (134·5–163·6) |  |
|  | Southern Latin America | 2460 (2190–2700) | 110·7 (95·8–124·9) | 2260 (1980–2540) | 120·4 (99·6–145·1) | 1410 (1220–1600) | 124·9 (103·1–146·8) |  |
|  | Argentina | 1790 (1590–1980) | 95·6 (78·3–112·6) | 1650 (1440–1860) | 102·3 (79·2–131·2) | 1050 (901–1190) | 105·5 (81·6–129·9) |  |
|  | Chile | 544 (485–604) | 203·7 (180·4–226·3) | 498 (430–569) | 242·3 (210·8–274·2) | 290 (246–334) | 278·8 (240·3–323·2) |  |
|  | Uruguay | 122 (106–139) | 69·1 (56·5–85·1) | 109 (90·9–128) | 75·1 (59·7–92·0) | 71·6 (60·3–85·5) | 81·4 (63·8–100·5) |  |
|  | Western Europe | 25600 (22600–28400) | 52·8 (48·4–58·1) | 22500 (19100–26100) | 59·8 (54·5–66·1) | 8330 (6570–10200) | 63·5 (57·3–70·7) |  |
|  | Andorra | 3·79 (3·3–4·35) | 114·8 (101·9–128·6) | 3·55 (3–4·18) | 117·9 (102·7–136·0) | 1·27 (0·996–1·58) | 124·8 (105·8–145·1) |  |
|  | Austria | 341 (298–389) | 54·1 (43·0–64·4) | 314 (263–366) | 59·7 (46·6–72·7) | 115 (88·7–142) | 65·4 (51·0–80·5) |  |
|  | Belgium | 520 (450–589) | 53·5 (40·7–65·3) | 474 (398–560) | 59·1 (43·8–72·6) | 173 (133–215) | 64·3 (48·3–79·9) |  |
|  | Cyprus | 75·2 (65·5–86·1) | 124·7 (107·5–144·4) | 68·1 (57·4–80·4) | 159·9 (130·2–188·2) | 24·4 (19·4–30·3) | 167·5 (137·4–198·8) |  |
|  | Denmark | 265 (229–297) | 100·5 (84·0–117·1) | 241 (201–282) | 118·6 (95·5–142·5) | 88·8 (69·2–110) | 131·1 (102·6–159·7) |  |
|  | Finland | 495 (433–550) | 62·7 (49·8–77·5) | 472 (401–529) | 67·4 (51·0–85·4) | 176 (140–210) | 78·0 (58·1–99·6) |  |
|  | France | 3210 (2790–3680) | 73·3 (52·8–97·7) | 2870 (2390–3430) | 92·1 (63·2–123·5) | 1050 (807–1330) | 99·1 (68·7–134·5) |  |
|  | Germany | 5360 (4610–6130) | 32·8 (21·4–43·6) | 4810 (3950–5700) | 36·6 (23·1–48·8) | 1790 (1360–2190) | 40·7 (25·9–55·5) |  |
|  | Greece | 554 (484–634) | 49·8 (37·4–62·6) | 483 (404–574) | 47·1 (34·2–61·2) | 181 (138–226) | 53·6 (39·6–69·2) |  |
|  | Iceland | 15·4 (13·4–17·6) | 127·5 (109·1–147·0) | 14·2 (11·8–16·8) | 131·2 (110·2–151·8) | 5·24 (4·14–6·39) | 138·4 (115·0–161·2) |  |
|  | Ireland | 196 (172–224) | 188·6 (163·3–219·3) | 182 (154–213) | 217·3 (182·5–256·8) | 66·8 (52·6–82·4) | 230·4 (193·4–272·1) |  |
|  | Israel | 334 (293–371) | 103·5 (88·3–119·4) | 300 (253–346) | 120·8 (101·9–143·0) | 111 (87·3–138) | 123·7 (99·6–145·0) |  |
|  | Italy | 3470 (2970–3980) | 31·9 (27·2–37·1) | 3210 (2700–3790) | 33·6 (28·3–39·3) | 1180 (905–1460) | 39·3 (33·4–46·3) |  |
|  | Luxembourg | 24·8 (21·5–28·8) | 114·3 (93·8–136·3) | 22·5 (18·8–26·8) | 128·7 (101·6–156·3) | 8·1 (6·21–10·3) | 134·5 (105·9–163·8) |  |
|  | Malta | 28 (24·4–31·7) | 114·6 (94·3–133·1) | 25·3 (21·2–29·9) | 125·8 (100·3–153·0) | 9·37 (7·33–11·7) | 138·6 (112·8–170·4) |  |
|  | Monaco | 2·19 (1·88–2·55) | 86·1 (74·2–98·3) | 1·97 (1·64–2·37) | 91·2 (76·3–107·0) | 0·75 (0·583–0·936) | 96·5 (79·7–114·6) |  |
|  | Netherlands | 764 (667–861) | 46·6 (36·0–57·3) | 709 (599–821) | 54·0 (40·4–66·9) | 262 (207–321) | 61·1 (46·9–77·3) |  |
|  | Norway | 296 (252–343) | 48·7 (43·8–54·8) | 288 (240–337) | 50·2 (45·0–56·3) | 109 (85·2–134) | 54·0 (45·6–60·6) |  |
|  | Portugal | 835 (748–916) | 54·0 (40·3–67·9) | 796 (696–889) | 60·4 (41·7–78·6) | 297 (237–351) | 69·3 (48·2–88·9) |  |
|  | San Marino | 2·13 (1·84–2·45) | 147·2 (131·6–163·1) | 1·98 (1·67–2·34) | 148·2 (130·7–166·4) | 0·744 (0·587–0·921) | 155·9 (134·4–179·7) |  |
|  | Spain | 3520 (3130–3900) | 51·5 (39·5–64·5) | 2520 (2040–3040) | 76·2 (55·0–102·9) | 947 (725–1190) | 80·4 (58·8–104·6) |  |
|  | Sweden | 669 (587–748) | 73·5 (62·8–86·4) | 628 (533–718) | 78·6 (64·7–94·1) | 230 (183–284) | 83·4 (67·7–99·5) |  |
|  | Switzerland | 359 (308–417) | 58·7 (43·2–74·9) | 318 (261–387) | 70·4 (50·1–91·0) | 132 (99·9–165) | 74·0 (52·2–100·3) |  |
|  | UK | 4240 (3700–4800) | 79·5 (72·5–85·8) | 3750 (3160–4400) | 81·8 (73·6–88·8) | 1360 (1070–1670) | 73·7 (65·4–81·6) |  |
|  | **Latin America and Caribbean** | **29800 (25900–34500)** | **173·1 (163·0–182·0)** | **27300 (22800–32400)** | **182·2 (169·7–193·3)** | **11400 (8980–14100)** | **210·2 (195·1–225·3)** |  |
|  | Andean Latin America | 1710 (1510–1920) | 227·5 (211·1–243·5) | 1410 (1180–1670) | 267·2 (242·1–290·3) | 606 (491–731) | 304·1 (275·7–330·0) |  |
|  | Bolivia | 288 (242–341) | 193·4 (171·6–213·1) | 215 (173–271) | 246·1 (222·8–270·2) | 87·5 (65·9–112) | 276·5 (248·8–309·0) |  |
|  | Ecuador | 682 (592–769) | 224·1 (182·9–265·1) | 630 (538–730) | 236·3 (189·4–283·2) | 281 (228–336) | 271·0 (214·4–329·5) |  |
|  | Peru | 747 (640–853) | 246·4 (220·9–276·6) | 567 (455–689) | 321·0 (285·1–359·1) | 238 (187–292) | 365·8 (323·2–414·7) |  |
|  | Caribbean | 2460 (2180–2770) | 96·8 (88·3–105·6) | 2240 (1890–2580) | 98·9 (89·4–108·1) | 919 (740–1110) | 115·4 (103·4–127·2) |  |
|  | Antigua and Barbuda | 6·92 (6·01–8) | 107·7 (94·8–122·4) | 6·45 (5·42–7·6) | 109·5 (94·6–126·0) | 2·64 (2·11–3·26) | 127·6 (109·4–147·7) |  |
|  | The Bahamas | 25·5 (21·4–30·2) | 158·0 (139·5–184·3) | 22·8 (18·5–27·6) | 160·4 (140·6–188·4) | 9·16 (7·04–11·6) | 183·6 (161·0–216·1) |  |
|  | Barbados | 35·6 (31·3–40·9) | 92·2 (77·1–110·3) | 32·8 (27·8–38·6) | 94·3 (77·8–113·4) | 14·1 (11·6–17) | 112·0 (91·8–137·6) |  |
|  | Belize | 21·8 (18·9–25·2) | 262·4 (239·5–287·7) | 18·9 (15·8–22·6) | 284·8 (256·4–317·5) | 7·81 (6·03–9·62) | 304·3 (268·5–342·8) |  |
|  | Bermuda | 5·01 (4·35–5·64) | 74·4 (59·4–87·1) | 4·89 (4·23–5·56) | 75·8 (60·5–89·0) | 2·22 (1·85–2·61) | 100·8 (80·3–121·8) |  |
|  | Cuba | 585 (500–664) | 89·1 (71·8–107·5) | 563 (475–647) | 91·0 (73·3–109·8) | 235 (188–281) | 111·9 (87·4–137·6) |  |
|  | Dominica | 5·9 (4·98–6·81) | 79·1 (68·4–91·6) | 5 (4·05–6·02) | 76·5 (64·8–91·1) | 2·12 (1·65–2·69) | 90·2 (75·4–107·3) |  |
|  | Dominican Republic | 497 (416–578) | 173·3 (157·7–191·9) | 436 (360–524) | 175·8 (157·8–195·4) | 170 (132–214) | 195·5 (174·3–219·4) |  |
|  | Grenada | 10·4 (9–11·9) | 86·9 (73·4–101·0) | 9·32 (7·73–11) | 86·4 (72·3–102·0) | 3·86 (3·04–4·7) | 102·0 (84·0–120·7) |  |
|  | Guyana | 55·9 (49·5–64·4) | 77·5 (63·6–95·7) | 51·1 (44·1–60·2) | 83·5 (68·1–106·6) | 19·6 (15·7–24·3) | 101·3 (79·7–127·7) |  |
|  | Haiti | 295 (244–346) | 124·4 (109·9–140·0) | 232 (188–287) | 135·7 (117·7–153·5) | 80·2 (59·3–103) | 146·2 (124·9–165·7) |  |
|  | Jamaica | 179 (155–207) | 87·0 (72·5–103·6) | 163 (138–194) | 87·5 (72·6–105·2) | 66·9 (52·6–81·7) | 100·2 (83·0–120·8) |  |
|  | Puerto Rico | 391 (343–438) | 42·7 (30·0–53·9) | 377 (325–428) | 46·1 (31·6–59·2) | 171 (141–203) | 63·7 (46·2–80·5) |  |
|  | Saint Kitts and Nevis | 3·45 (2·92–4·08) | 96·5 (78·2–116·1) | 3·05 (2·45–3·7) | 99·8 (79·0–124·0) | 1·24 (0·951–1·53) | 111·8 (84·7–142·0) |  |
|  | Saint Lucia | 13·4 (11·5–15·4) | 104·8 (85·8–130·5) | 11·4 (9·59–13·5) | 106·3 (82·8–137·1) | 4·75 (3·81–5·62) | 117·8 (89·6–150·5) |  |
|  | Saint Vincent and the Grenadines | 10·3 (8·88–11·8) | 91·9 (79·7–103·9) | 9·31 (7·68–10·9) | 93·7 (79·2–107·3) | 3·79 (2·95–4·64) | 111·0 (94·5–128·2) |  |
|  | Suriname | 44 (37·7–51·6) | 196·1 (172·4–224·4) | 38·3 (31·4–46·5) | 206·7 (180·1–237·1) | 14·6 (11·1–18·5) | 229·5 (198·3–262·4) |  |
|  | Trinidad and Tobago | 190 (164–215) | 87·0 (68·1–115·5) | 172 (143–200) | 94·6 (73·0–124·7) | 71·3 (55·6–86·1) | 112·0 (88·8–146·9) |  |
|  | Virgin Islands | 8·72 (7·48–9·95) | 63·4 (50·1–79·6) | 8·19 (6·85–9·57) | 72·7 (56·1–92·3) | 3·62 (2·88–4·42) | 95·9 (74·5–122·7) |  |
|  | Central Latin America | 16100 (13900–18500) | 181·1 (169·2–192·4) | 14900 (12500–17600) | 188·7 (174·4–203·4) | 6170 (4850–7530) | 237·5 (216·7–256·3) |  |
|  | Colombia | 2290 (1950–2620) | 213·0 (189·7–242·5) | 2200 (1820–2550) | 224·7 (198·4–257·1) | 1150 (925–1360) | 251·2 (216·5–291·2) |  |
|  | Costa Rica | 341 (289–398) | 234·4 (199·7–272·6) | 323 (266–383) | 243·1 (202·2–288·5) | 139 (112–170) | 267·5 (211·3–321·0) |  |
|  | El Salvador | 259 (216–303) | 121·9 (102·5–140·4) | 235 (191–284) | 129·6 (108·6–148·8) | 103 (78·8–126) | 139·3 (117·5–165·3) |  |
|  | Guatemala | 602 (539–674) | 280·5 (240·1–324·6) | 502 (442–574) | 306·5 (250·7–373·0) | 214 (173–256) | 325·6 (268·0–393·7) |  |
|  | Honduras | 218 (183–257) | 220·5 (201·1–241·3) | 184 (151–228) | 236·5 (215·5–259·7) | 74·3 (56·8–92·8) | 246·9 (224·2–271·5) |  |
|  | Mexico | 10800 (9280–12700) | 172·4 (158·2–185·0) | 9990 (8300–12100) | 180·0 (163·1–196·8) | 3790 (2940–4680) | 241·3 (217·2–264·4) |  |
|  | Nicaragua | 185 (156–216) | 224·4 (201·4–246·4) | 170 (141–204) | 238·6 (212·4–261·9) | 72·3 (56·9–90·5) | 251·6 (220·6–279·9) |  |
|  | Panama | 223 (187–254) | 216·8 (196·1–246·5) | 214 (175–247) | 222·6 (199·6–253·8) | 91·6 (70·9–109) | 241·0 (213·6–277·1) |  |
|  | Venezuela | 1240 (1030–1470) | 158·1 (135·7–180·5) | 1110 (898–1350) | 156·5 (133·6–179·4) | 487 (361–606) | 169·5 (142·2–196·5) |  |
|  | Tropical Latin America | 9540 (8120–11300) | 178·9 (166·9–190·5) | 8730 (7200–10500) | 191·7 (176·6–206·6) | 3730 (2920–4650) | 199·1 (181·8–217·2) |  |
|  | Brazil | 9260 (7880–11000) | 179·1 (167·0–190·9) | 8480 (7010–10200) | 192·2 (176·8–207·4) | 3630 (2840–4520) | 199·9 (181·7–218·1) |  |
|  | Paraguay | 279 (240–333) | 173·5 (152·9–193·1) | 247 (203–305) | 175·8 (154·9–197·3) | 103 (79·8–128) | 174·5 (154·4–196·4) |  |
|  | **North Africa and Middle East** | **43500 (38500–48800)** | **307·0 (290·1–321·3)** | **40300 (35200–46100)** | **325·8 (303·7–345·0)** | **18100 (14900–22200)** | **356·8 (331·2–381·6)** |  |
|  | Afghanistan | 840 (712–994) | 244·3 (204·1–296·7) | 780 (645–937) | 259·6 (209·1–325·5) | 360 (286–455) | 286·6 (223·7–350·6) |  |
|  | Algeria | 3150 (2780–3530) | 358·6 (321·0–399·1) | 2930 (2540–3320) | 376·2 (329·0–425·0) | 1220 (1000–1470) | 349·9 (289·3–409·8) |  |
|  | Bahrain | 211 (178–250) | 618·5 (545·2–695·2) | 200 (162–241) | 665·8 (581·0–761·1) | 88 (67·5–114) | 715·2 (632·3–822·1) |  |
|  | Egypt | 7540 (6720–8240) | 239·8 (213·3–271·7) | 6720 (5960–7440) | 236·6 (207·9–272·9) | 2920 (2420–3600) | 260·9 (227·5–301·4) |  |
|  | Iran | 5710 (4910–6570) | 295·2 (283·4–307·9) | 4960 (4190–6000) | 318·7 (301·7–336·1) | 2150 (1700–2760) | 285·6 (265·6–307·2) |  |
|  | Iraq | 2710 (2370–3070) | 413·1 (353·5–489·6) | 2550 (2220–2950) | 473·0 (388·6–569·3) | 931 (752–1130) | 510·4 (411·8–625·7) |  |
|  | Jordan | 1110 (1000–1220) | 619·0 (547·3–683·8) | 1050 (944–1170) | 707·6 (597·2–792·8) | 458 (381–533) | 870·6 (738·1–988·6) |  |
|  | Kuwait | 693 (605–789) | 584·1 (509·1–656·3) | 658 (561–768) | 595·4 (504·8–678·4) | 222 (175–283) | 797·9 (633·9–993·2) |  |
|  | Lebanon | 383 (331–436) | 254·4 (215·4–290·6) | 361 (310–416) | 276·4 (228·2–323·8) | 184 (149–220) | 293·4 (237·6–342·7) |  |
|  | Libya | 581 (494–692) | 338·8 (295·7–389·5) | 494 (402–611) | 323·3 (273·1–388·0) | 197 (147–255) | 354·0 (294·5–428·4) |  |
|  | Morocco | 2180 (1920–2460) | 244·5 (209·2–282·5) | 1940 (1680–2250) | 273·6 (222·2–332·0) | 704 (568–847) | 299·0 (241·1–367·8) |  |
|  | Oman | 303 (254–361) | 504·9 (460·1–561·6) | 283 (232–344) | 514·2 (462·3–578·2) | 126 (93·1–162) | 572·6 (512·7–650·2) |  |
|  | Palestine | 333 (285–387) | 343·7 (303·2–389·8) | 318 (269–374) | 366·8 (316·4–422·3) | 101 (78·2–130) | 407·2 (337·3–494·5) |  |
|  | Qatar | 341 (288–399) | 922·5 (812·5–1033·1) | 324 (268–386) | 1009·0 (859·0–1163·1) | 171 (131–224) | 1108·9 (949·8–1285·2) |  |
|  | Saudi Arabia | 4560 (3930–5390) | 345·6 (302·8–398·7) | 4490 (3830–5340) | 351·1 (305·7–406·0) | 2720 (2260–3310) | 520·3 (428·6–669·5) |  |
|  | Sudan | 1450 (1280–1630) | 245·6 (212·2–281·1) | 1320 (1140–1520) | 268·6 (223·8–314·9) | 383 (302–473) | 306·6 (253·7–368·2) |  |
|  | Syria | 1480 (1250–1740) | 297·9 (265·2–332·6) | 1370 (1140–1650) | 307·4 (271·2–346·0) | 593 (462–751) | 333·6 (296·2–380·4) |  |
|  | Tunisia | 1120 (967–1290) | 208·5 (184·0–235·6) | 1080 (919–1270) | 215·9 (188·4–244·9) | 450 (365–557) | 231·7 (200·0–268·2) |  |
|  | Türkiye | 6430 (5540–7510) | 257·0 (224·0–290·1) | 6130 (5180–7280) | 287·9 (241·1–332·9) | 2990 (2380–3610) | 309·8 (262·4–356·0) |  |
|  | United Arab Emirates | 1580 (1350–1820) | 1038·0 (913·2–1167·6) | 1490 (1250–1760) | 1052·9 (908·7–1207·6) | 872 (699–1100) | 1097·3 (937·5–1283·2) |  |
|  | Yemen | 848 (699–1030) | 581·5 (534·6–629·2) | 765 (613–958) | 614·0 (565·0–675·8) | 294 (219–378) | 638·7 (581·6–702·7) |  |
|  | **South Asia** | **58100 (48600–70600)** | **247·9 (233·7–260·1)** | **55600 (45600–68200)** | **272·4 (258·7–287·1)** | **19100 (14400–24000)** | **269·8 (253·0–287·6)** |  |
|  | Bangladesh | 5290 (4760–5890) | 186·7 (156·4–218·9) | 4850 (4380–5430) | 204·2 (166·7–248·4) | 1950 (1640–2290) | 169·8 (119·8–231·6) |  |
|  | Bhutan | 21 (18–24·7) | 193·5 (158·6–234·5) | 19·2 (16·1–23·1) | 236·1 (190·7–294·8) | 12·1 (9·81–14·6) | 275·6 (224·8–347·0) |  |
|  | India | 46400 (38000–57000) | 275·1 (257·4–289·1) | 45200 (36500–55700) | 295·9 (279·9–311·4) | 15000 (11100–19000) | 308·3 (289·1–329·7) |  |
|  | Nepal | 729 (629–845) | 155·5 (130·5–179·7) | 595 (497–719) | 187·1 (151·4–223·5) | 190 (145–242) | 205·7 (168·6–241·0) |  |
|  | Pakistan | 5710 (4780–6990) | 165·6 (155·4–177·6) | 4990 (4030–6240) | 190·6 (180·1–202·0) | 2040 (1560–2610) | 178·8 (159·7–196·2) |  |
|  | **Southeast Asia, east Asia, and Oceania** | **72800 (62300–86600)** | **111·6 (98·9–124·8)** | **63800 (52900–78900)** | **156·2 (144·7–167·7)** | **24700 (18900–30900)** | **155·5 (143·0–166·7)** |  |
|  | East Asia | 51500 (43700–61400) | 92·0 (78·5–105·8) | 44600 (36600–55400) | 139·9 (128·1–151·5) | 15800 (11900–20000) | 130·1 (117·4–141·5) |  |
|  | China | 49500 (42000–59200) | 91·5 (77·8–105·5) | 42800 (35100–53300) | 140·7 (128·6–152·9) | 15100 (11400–19200) | 130·1 (116·8–142·1) |  |
|  | North Korea | 700 (583–829) | 92·9 (78·1–105·4) | 553 (454–688) | 122·2 (104·1–138·2) | 198 (151–253) | 125·3 (106·9–145·0) |  |
|  | Taiwan | 1300 (1080–1520) | 110·0 (91·2–130·4) | 1210 (982–1440) | 120·7 (99·7–144·3) | 475 (361–589) | 130·8 (107·2–157·9) |  |
|  | Oceania | 465 (402–541) | 158·2 (144·8–171·8) | 382 (317–465) | 164·1 (148·6–180·6) | 93 (68·3–121) | 171·0 (148·9–195·7) |  |
|  | American Samoa | 4·53 (4·1–4·98) | 76·5 (55·7–95·4) | 3·87 (3·49–4·29) | 77·7 (53·4–101·4) | 1·44 (1·25–1·65) | 106·3 (72·6–147·0) |  |
|  | Cook Islands | 1·3 (1·11–1·52) | 34·3 (24·8–46·2) | 1·11 (0·916–1·35) | 46·5 (34·6–61·1) | 0·393 (0·298–0·496) | 64·2 (46·5–86·9) |  |
|  | Fiji | 96·7 (84·4–110) | 128·8 (107·0–153·7) | 79·2 (64·5–96·9) | 127·5 (97·0–162·0) | 22·2 (16·1–28·8) | 138·8 (103·7–178·9) |  |
|  | Guam | 8·57 (7·27–10·1) | 88·1 (71·7–104·5) | 8·02 (6·64–9·65) | 89·4 (71·9–106·9) | 2·11 (1·58–2·68) | 105·5 (83·8–126·0) |  |
|  | Kiribati | 5·97 (5·17–6·9) | 136·9 (122·7–154·3) | 4·16 (3·42–5·1) | 133·7 (114·6–155·4) | 1·03 (0·764–1·32) | 151·6 (129·5–178·7) |  |
|  | Marshall Islands | 2·45 (2·2–2·7) | 55·3 (39·6–72·6) | 1·65 (1·45–1·82) | 60·6 (39·4–83·5) | 0·307 (0·261–0·358) | 87·0 (51·0–136·5) |  |
|  | Federated States of Micronesia | 6·79 (6·28–7·38) | 135·8 (114·8–157·3) | 4·66 (4·16–5·19) | 140·8 (115·0–167·3) | 0·812 (0·681–0·968) | 111·2 (67·9–161·0) |  |
|  | Nauru | 0·812 (0·706–0·901) | 77·0 (67·7–87·4) | 0·552 (0·462–0·658) | 100·9 (85·7–117·0) | 0·205 (0·158–0·258) | 92·1 (69·6–114·3) |  |
|  | Niue | 0·19 (0·163–0·221) | 29·9 (20·8–38·8) | 0·155 (0·123–0·19) | 25·8 (16·0–36·2) | 0·0376 (0·0276–0·0484) | 31·8 (19·3–43·2) |  |
|  | Northern Mariana Islands | 3·4 (2·89–4·05) | 61·1 (41·7–80·3) | 2·96 (2·35–3·7) | 62·4 (40·2–86·7) | 0·798 (0·58–1·07) | 81·9 (46·6–114·8) |  |
|  | Palau | 1·57 (1·43–1·71) | 92·9 (69·3–119·7) | 1·25 (1·13–1·38) | 93·2 (62·0–127·3) | 0·281 (0·243–0·329) | 116·3 (71·3–183·7) |  |
|  | Papua New Guinea | 269 (229–323) | 193·8 (176·2–214·9) | 232 (189–286) | 206·5 (186·9–230·8) | 50·8 (36·7–67·3) | 215·2 (187·8–246·4) |  |
|  | Samoa | 8·97 (7·62–10·3) | 99·7 (82·9–117·8) | 6·77 (5·48–8·22) | 101·4 (80·0–123·6) | 2·01 (1·53–2·57) | 118·1 (90·8–149·3) |  |
|  | Solomon Islands | 20·7 (17·4–23·8) | 233·8 (210·1–253·2) | 9·54 (7·74–11·8) | 231·5 (207·2–259·4) | 2·49 (1·85–3·33) | 235·4 (202·9–273·6) |  |
|  | Tokelau | 0·171 (0·151–0·191) | 55·9 (45·4–67·0) | 0·124 (0·105–0·146) | 50·6 (37·4–67·4) | 0·0211 (0·0161–0·0269) | 58·4 (39·4–79·4) |  |
|  | Tonga | 6·24 (5·42–7·27) | 79·6 (66·6–92·2) | 4·89 (3·98–6·04) | 79·4 (65·5–94·5) | 1·03 (0·736–1·34) | 92·4 (73·4–111·2) |  |
|  | Tuvalu | 0·68 (0·594–0·775) | 88·8 (74·6–100·8) | 0·549 (0·455–0·657) | 91·7 (75·7–107·5) | 0·142 (0·106–0·18) | 116·0 (91·8–145·3) |  |
|  | Vanuatu | 5·73 (4·82–6·87) | 175·6 (158·4–195·2) | 4·46 (3·64–5·54) | 183·6 (165·5–204·1) | 1·92 (1·46–2·43) | 191·3 (168·0–215·5) |  |
|  | Southeast Asia | 20800 (18000–24100) | 181·7 (170·1–193·6) | 18800 (15700–22400) | 205·4 (192·7–217·9) | 8730 (6940–10700) | 218·2 (202·0–235·8) |  |
|  | Cambodia | 343 (290–408) | 222·3 (194·8–254·3) | 305 (250–372) | 270·6 (232·9–310·5) | 157 (125–196) | 280·3 (243·9–326·2) |  |
|  | Indonesia | 7160 (6050–8660) | 198·5 (184·0–212·1) | 6280 (5080–7870) | 225·3 (210·8–238·9) | 2730 (2100–3470) | 240·9 (224·2–256·7) |  |
|  | Laos | 161 (138–188) | 165·7 (147·4–186·5) | 137 (115–164) | 222·5 (192·8–254·0) | 54·8 (42·8–67·6) | 239·9 (205·8–281·0) |  |
|  | Malaysia | 1270 (1050–1540) | 193·3 (150·7–245·2) | 1220 (1010–1490) | 202·1 (155·4–260·5) | 655 (516–813) | 215·5 (160·1–279·7) |  |
|  | Maldives | 10·5 (8·86–12·5) | 334·6 (297·3–380·1) | 9·57 (7·84–11·5) | 379·3 (335·2–441·7) | 4·35 (3·36–5·44) | 405·0 (352·6–477·7) |  |
|  | Mauritius | 143 (124–169) | 123·4 (107·7–142·1) | 133 (111–162) | 129·0 (110·9–150·2) | 63·4 (50·6–78·7) | 147·2 (124·1–178·4) |  |
|  | Myanmar | 1920 (1680–2160) | 138·3 (115·3–161·0) | 1710 (1460–1970) | 182·1 (145·8–218·7) | 750 (601–902) | 189·6 (150·6–233·8) |  |
|  | Philippines | 2690 (2270–3250) | 154·1 (147·5–160·9) | 2380 (1930–2940) | 162·0 (154·4–169·2) | 1040 (793–1320) | 171·1 (161·9–179·6) |  |
|  | Seychelles | 6·7 (5·69–7·85) | 183·6 (159·7–215·4) | 6·36 (5·33–7·55) | 187·7 (160·6–223·3) | 1·28 (0·968–1·67) | 212·9 (172·5–254·5) |  |
|  | Sri Lanka | 1660 (1500–1850) | 169·9 (143·6–196·6) | 1490 (1310–1720) | 220·2 (175·6–270·3) | 803 (697–918) | 239·7 (189·1–297·6) |  |
|  | Thailand | 3180 (2670–3720) | 197·3 (160·8–230·1) | 3080 (2530–3630) | 203·5 (163·5–239·7) | 1140 (876–1420) | 225·8 (165·5–283·9) |  |
|  | Timor-Leste | 9·29 (7·43–11·4) | 173·5 (148·4–197·6) | 7·56 (5·86–9·54) | 212·0 (187·4–241·2) | 3·44 (2·43–4·71) | 223·1 (193·9–258·9) |  |
|  | Viet Nam | 2230 (1910–2620) | 200·2 (167·3–231·1) | 2040 (1710–2460) | 225·5 (184·8–264·8) | 1280 (1050–1570) | 221·0 (177·3–261·2) |  |
|  | **Sub-Saharan Africa** | **10900 (9140–12800)** | **142·1 (136·8–148·0)** | **8850 (7230–10800)** | **159·6 (154·0–164·4)** | **4280 (3380–5360)** | **165·1 (158·1–172·2)** |  |
|  | Central sub-Saharan Africa | 871 (720–1030) | 157·9 (142·8–172·4) | 602 (496–737) | 210·2 (195·8–227·5) | 306 (235–389) | 218·0 (201·2–236·1) |  |
|  | Angola | 266 (224–309) | 217·8 (192·2–246·0) | 167 (136–208) | 304·9 (274·6–334·3) | 102 (78–128) | 324·8 (296·8–355·8) |  |
|  | Central African Republic | 37·7 (31·4–44·7) | 140·1 (121·9–155·1) | 29·2 (24·4–36) | 152·8 (134·5–171·1) | 15·7 (12·5–19·7) | 162·7 (141·0–183·8) |  |
|  | Congo (Brazzaville) | 46·6 (38·6–55) | 154·5 (135·6–171·9) | 33·2 (27–41·9) | 198·3 (177·7–217·6) | 17·9 (14·1–22·7) | 203·7 (179·4–227·1) |  |
|  | DR Congo | 485 (397–581) | 135·5 (117·5–154·3) | 348 (289–429) | 185·3 (166·3–207·2) | 161 (124–207) | 183·1 (161·0–207·3) |  |
|  | Equatorial Guinea | 10·5 (8·58–12·5) | 245·6 (219·6–268·3) | 8·17 (6·48–10·1) | 322·3 (294·3–352·4) | 4·46 (3·44–5·56) | 335·2 (299·8–372·6) |  |
|  | Gabon | 22·3 (18·6–26·4) | 133·2 (117·4–151·3) | 16 (12·9–19·6) | 164·6 (146·6–184·6) | 9·12 (7·23–11·5) | 174·7 (152·7–196·1) |  |
|  | Eastern sub-Saharan Africa | 3060 (2580–3540) | 133·0 (127·0–139·5) | 2450 (2010–2940) | 158·1 (150·3–164·4) | 1340 (1070–1650) | 161·2 (152·6–168·1) |  |
|  | Burundi | 82 (68·5–96·4) | 135·2 (121·5–148·9) | 68·1 (55·2–82·7) | 156·5 (139·1–172·6) | 36 (28·3–45·8) | 154·8 (137·4–173·6) |  |
|  | Comoros | 13·8 (11·7–15·9) | 154·4 (140·5–169·1) | 12·5 (10·5–15) | 165·9 (148·0–184·1) | 5·59 (4·44–6·95) | 175·5 (155·4–196·6) |  |
|  | Djibouti | 13·1 (10·8–15·5) | 291·4 (268·2–318·8) | 11·3 (9·17–13·9) | 331·3 (309·5–360·9) | 6·12 (4·78–7·58) | 341·5 (316·5–373·6) |  |
|  | Eritrea | 86·3 (72·8–102) | 167·4 (143·8–192·6) | 80·2 (66·7–97·3) | 180·4 (150·5–206·6) | 41·9 (34–50·8) | 181·7 (154·3–212·3) |  |
|  | Ethiopia | 465 (391–535) | 81·2 (70·0–92·6) | 326 (267–397) | 151·5 (139·0–165·9) | 178 (140–225) | 157·4 (144·1–172·2) |  |
|  | Kenya | 537 (452–630) | 151·6 (147·8–156·5) | 458 (376–560) | 146·7 (142·4–153·2) | 276 (217–339) | 159·2 (153·5–166·4) |  |
|  | Madagascar | 191 (158–225) | 144·7 (128·2–160·5) | 163 (131–198) | 160·9 (140·1–179·1) | 86 (67–106) | 161·9 (141·7–184·2) |  |
|  | Malawi | 127 (105–145) | 118·7 (105·5–132·6) | 99·5 (82·2–120) | 141·5 (122·2–158·5) | 61·1 (48–75·9) | 144·1 (123·3–164·3) |  |
|  | Mozambique | 198 (166–231) | 116·8 (101·4–132·8) | 174 (140–212) | 123·9 (106·6–142·6) | 114 (87·2–145) | 123·4 (104·1–141·5) |  |
|  | Rwanda | 79·1 (67·7–91·1) | 158·9 (139·0–179·4) | 66·8 (55·4–80·1) | 219·1 (191·1–245·3) | 44·5 (35·5–54·8) | 230·6 (200·6–259·6) |  |
|  | Somalia | 130 (108–151) | 148·6 (134·0–163·6) | 93·5 (75·2–115) | 154·6 (136·9–170·6) | 51·2 (40·2–64·6) | 160·6 (141·8–179·6) |  |
|  | South Sudan | 60·1 (49·8–70·2) | 76·9 (66·8–88·0) | 47·6 (38·8–58·5) | 80·5 (67·4–93·5) | 25·5 (19·5–31·6) | 79·9 (64·4–94·8) |  |
|  | Uganda | 407 (343–472) | 145·7 (128·6–162·6) | 345 (288–418) | 176·9 (148·5–198·6) | 231 (188–277) | 176·8 (150·6–198·9) |  |
|  | Tanzania | 518 (435–596) | 152·3 (136·5–168·7) | 399 (326–484) | 171·6 (152·5–191·6) | 170 (132–215) | 177·8 (155·8–199·7) |  |
|  | Zambia | 131 (110–151) | 172·4 (148·4–200·3) | 100 (79·9–122) | 210·7 (178·1–244·5) | 32·5 (25·2–41·5) | 218·2 (177·5–255·1) |  |
|  | Southern sub-Saharan Africa | 2610 (2210–3130) | 115·9 (110·1–123·5) | 2320 (1900–2870) | 113·1 (106·7–122·1) | 851 (641–1080) | 102·8 (93·7–111·0) |  |
|  | Botswana | 47·4 (40·5–54·5) | 130·8 (114·3–149·6) | 43·5 (36·6–51·2) | 132·3 (113·5–153·1) | 23·3 (19·2–28·6) | 149·0 (126·6–171·6) |  |
|  | Eswatini | 25·2 (21·6–29·5) | 120·3 (105·6–138·9) | 23·7 (20·1–28·2) | 121·7 (105·7–141·2) | 11·9 (9·65–14·6) | 120·6 (102·1–142·7) |  |
|  | Lesotho | 34·8 (29·9–40·2) | 47·4 (37·8–55·3) | 32·2 (27·2–38) | 45·0 (35·1–53·0) | 14·6 (11·9–18·1) | 47·5 (35·6–58·2) |  |
|  | Namibia | 56·5 (47·3–67·4) | 150·9 (131·4–166·8) | 53·7 (44–64·7) | 154·3 (133·6–173·1) | 20·2 (16·1–25·3) | 170·0 (143·6–194·6) |  |
|  | South Africa | 2250 (1900–2710) | 120·8 (114·2–129·3) | 1980 (1620–2460) | 118·4 (110·6–128·3) | 696 (516–894) | 104·3 (93·4–115·3) |  |
|  | Zimbabwe | 202 (169–239) | 76·7 (63·7–89·5) | 186 (152–224) | 71·2 (58·6–84·7) | 80·5 (62·4–101) | 75·8 (61·9–91·1) |  |
|  | Western sub-Saharan Africa | 4340 (3640–5100) | 165·5 (156·8–175·6) | 3480 (2830–4280) | 195·5 (185·5–204·7) | 1680 (1310–2110) | 204·0 (191·2–217·7) |  |
|  | Benin | 97·3 (81·2–113) | 215·2 (190·6–241·9) | 83·1 (67·8–100) | 241·5 (213·8–271·7) | 43·4 (34·5–53·6) | 245·4 (213·1–277·7) |  |
|  | Burkina Faso | 103 (86·4–121) | 117·8 (106·0–129·7) | 82·9 (66·8–101) | 134·8 (121·4–150·6) | 39·4 (30·5–49·5) | 136·3 (121·2–152·4) |  |
|  | Cabo Verde | 15·2 (13·1–17·4) | 131·4 (111·9–156·5) | 13·9 (11·9–16·4) | 145·5 (120·0–176·0) | 5·54 (4·34–6·7) | 147·7 (115·5–184·9) |  |
|  | Cameroon | 395 (332–472) | 243·1 (208·1–270·0) | 346 (280–427) | 263·9 (220·1–298·4) | 191 (151–238) | 270·4 (224·6–312·5) |  |
|  | Chad | 98·5 (81·5–116) | 106·3 (94·4–120·4) | 73·8 (59·8–91·7) | 123·1 (108·3–138·8) | 33·5 (26·3–43·1) | 121·8 (106·4–138·5) |  |
|  | Côte d'Ivoire | 276 (230–321) | 178·8 (161·9–194·9) | 211 (171–257) | 213·1 (193·5–231·8) | 99·9 (77·6–126) | 220·4 (199·6–241·7) |  |
|  | The Gambia | 26·4 (21·9–31) | 159·4 (147·0–172·8) | 20·2 (16·3–25·1) | 176·7 (161·7–190·8) | 9·73 (7·76–12·3) | 184·0 (166·5–203·3) |  |
|  | Ghana | 607 (513–712) | 158·6 (143·5–174·3) | 482 (393–589) | 179·5 (158·9–200·0) | 213 (165–268) | 187·7 (163·1–214·2) |  |
|  | Guinea | 110 (92·7–129) | 77·7 (64·7–89·7) | 82·8 (67·9–101) | 94·5 (75·9–111·6) | 43·9 (34·3–55·6) | 85·9 (65·7–107·0) |  |
|  | Guinea-Bissau | 16·7 (13·9–19·6) | 108·3 (95·0–122·9) | 11·5 (9·37–14·4) | 137·7 (123·6–153·7) | 5·36 (4·23–6·77) | 139·3 (120·5–159·5) |  |
|  | Liberia | 62·4 (51·5–74·8) | 176·0 (154·7–199·4) | 52·3 (42·7–64·9) | 197·1 (169·9–229·9) | 26·3 (20·3–33·5) | 200·1 (167·3–237·3) |  |
|  | Mali | 239 (193–283) | 138·2 (123·2–156·9) | 179 (144–221) | 161·1 (141·7–182·6) | 70 (52·5–92·7) | 165·3 (139·6–189·8) |  |
|  | Mauritania | 47·6 (37·9–58·5) | 141·6 (123·4–163·7) | 35·5 (26·8–47·3) | 180·9 (158·2–206·6) | 18·9 (13·4–25·2) | 196·0 (168·6–225·6) |  |
|  | Niger | 57·8 (45·2–71) | 175·5 (153·5–201·9) | 39·7 (30·5–51) | 229·6 (206·9–253·7) | 17·5 (12·6–23·9) | 235·2 (209·9–263·2) |  |
|  | Nigeria | 1790 (1480–2120) | 175·6 (165·5–187·0) | 1460 (1200–1820) | 212·9 (202·6–223·7) | 715 (545–903) | 223·4 (208·8–238·7) |  |
|  | São Tomé and Príncipe | 3·47 (2·92–4·04) | 169·4 (145·4–193·6) | 2·65 (2·15–3·19) | 199·1 (164·1–229·1) | 1·34 (1·04–1·71) | 191·3 (153·2–229·1) |  |
|  | Senegal | 251 (209–295) | 158·5 (142·2–174·7) | 202 (166–248) | 185·6 (165·8–205·8) | 96·7 (77·7–120) | 191·2 (170·4–213·5) |  |
|  | Sierra Leone | 66·7 (55·2–78) | 86·8 (71·1–104·4) | 52 (42·6–63·8) | 103·8 (83·5–122·9) | 24·4 (19·1–30·9) | 103·8 (80·1–125·9) |  |
|  | Togo | 63 (52·2–75·7) | 206·7 (185·3–225·4) | 49·2 (39·8–62·2) | 239·7 (217·5–261·1) | 23·5 (18·3–29·8) | 251·0 (227·9–278·3) |  |
|  |  |  |  |  |  |  |  |  |

## Table S13. Counts of undiagnosed diabetes, diagnosed and untreated diabetes, treated diabetes with suboptimal glycaemic levels, and treated diabetes with optimal glycaemic levels in 2023, and corresponding percent change between 2000 and 2023, for both sexes and ages 15 and older globally, and by Global Burden of Disease super-regions, regions, and countries and territories

|  | **Location** | **Undiagnosed, 2023 (per 1,000)** | **Change in Undiagnosed, 2000-2023 (%)** | **Diagnosed & Untreated, 2023 (per 1,000)** | **Change in Diagnosed & Untreated, 2000-2023 (%)** | **Treated & Suboptimal Glycaemic Management, 2023 (per 1,000)** | **Change in Treated & Suboptimal Glycaemic Management, 2000-2023 (%)** | **Treated & Optimal Glycaemic Management, 2023 (per 1,000)** | **Change in Treated & Optimal Glycaemic Management, 2000-2023 (%)** |  |
| --- | --- | --- | --- | --- | --- | --- | --- | --- | --- | --- |
|  | **Global** | **248000 (207000–289000)** | **73·1 (61·9–83·3)** | **26600 (19400–36200)** | **30·8 (22·2–40·3)** | **167000 (134000–201000)** | **157·3 (151·4–162·6)** | **119000 (95200–145000)** | **171·7 (165·7–178·0)** |  |
|  | **Central Europe, eastern Europe, and central Asia** | **9160 (6920–11400)** | **59·6 (48·7–69·1)** | **1500 (937–2260)** | **49·3 (39·6–59·0)** | **8790 (6830–10900)** | **94·2 (86·3–101·5)** | **8930 (7170–10900)** | **125·3 (116·3–135·0)** |  |
|  | Central Asia | 2200 (1870–2500) | 96·5 (83·1–109·2) | 298 (210–427) | 54·3 (39·4–68·1) | 1330 (1060–1610) | 184·2 (160·2–204·9) | 968 (767–1190) | 195·2 (170·4–219·7) |  |
|  | Armenia | 72·3 (55·6–89) | 8·6 (-5·0–22·4) | 10·9 (6·39–17·6) | -16·6 (-32·3–-1·4) | 68·6 (56·7–83·3) | 65·4 (42·5–85·4) | 47·2 (36·9–57·9) | 84·1 (58·0–106·9) |  |
|  | Azerbaijan | 222 (176–271) | 82·6 (59·9–105·9) | 25·4 (15·9–39·2) | 49·3 (22·6–79·5) | 282 (234–334) | 207·0 (165·3–244·0) | 116 (90·5–146) | 238·9 (186·1–298·1) |  |
|  | Georgia | 62·6 (46·5–77·2) | 13·4 (-4·7–32·3) | 15·5 (10·1–22) | 7·1 (-17·6–32·4) | 86 (69·5–103) | 53·8 (33·0–76·1) | 70·3 (57·4–83·9) | 62·3 (39·6–86·6) |  |
|  | Kazakhstan | 459 (366–542) | 49·3 (30·1–68·7) | 55·4 (33·5–89·3) | -6·5 (-23·8–12·1) | 194 (145–251) | 170·4 (130·2–208·1) | 418 (346–500) | 192·9 (155·3–231·3) |  |
|  | Kyrgyzstan | 111 (90·3–130) | 86·6 (64·9–106·8) | 9·62 (6·33–14·8) | 38·6 (16·6–61·1) | 105 (84·9–128) | 215·7 (178·9–257·1) | 29·5 (22·1–38·1) | 242·3 (197·6–297·2) |  |
|  | Mongolia | 73·7 (63·7–82·9) | 143·2 (124·8–165·0) | 13 (9·95–17) | 99·8 (75·2–125·9) | 28·7 (22·3–35·7) | 277·1 (233·8–331·2) | 18·4 (14·5–23·3) | 295·0 (253·1–350·7) |  |
|  | Tajikistan | 257 (230–285) | 203·6 (180·3–225·6) | 57·7 (44·8–73·3) | 166·4 (126·7–202·4) | 81·1 (63·1–105) | 291·8 (250·1–337·0) | 24 (16–35·5) | 308·2 (250·4–363·6) |  |
|  | Turkmenistan | 170 (155–184) | 156·6 (132·2–176·0) | 12·6 (9·52–16·6) | 108·2 (80·4–138·0) | 41·9 (33·1–51·2) | 218·3 (182·8–257·8) | 14·3 (10·5–18·6) | 238·9 (194·6–288·7) |  |
|  | Uzbekistan | 770 (622–900) | 134·8 (108·3–158·3) | 98·7 (67·5–139) | 110·4 (79·0–146·7) | 431 (332–539) | 245·4 (214·1–282·7) | 239 (182–308) | 274·2 (227·7–326·9) |  |
|  | Central Europe | 2990 (2070–3920) | 43·9 (30·6–55·2) | 613 (366–947) | 38·3 (25·8–50·2) | 3680 (2880–4640) | 64·9 (56·3–73·4) | 3350 (2630–4100) | 70·0 (59·7–80·2) |  |
|  | Albania | 42·5 (27·5–56·7) | 73·3 (40·8–101·5) | 8·31 (4·79–12·6) | 62·3 (32·4–91·1) | 46·7 (35·3–60·4) | 137·8 (115·7–163·7) | 34·4 (25·5–43·7) | 164·8 (137·3–195·0) |  |
|  | Bosnia and Herzegovina | 112 (75–146) | 72·6 (47·5–95·5) | 25 (15–36·8) | 55·8 (30·6–82·9) | 115 (85·7–148) | 110·2 (85·7–134·2) | 84·6 (61·5–111) | 137·1 (107·0–162·7) |  |
|  | Bulgaria | 214 (152–266) | 25·6 (16·2–35·8) | 55·2 (35·4–80·5) | 19·2 (7·6–30·9) | 187 (142–245) | 31·2 (21·6–40·9) | 140 (104–185) | 44·3 (31·1–55·0) |  |
|  | Croatia | 122 (75·7–170) | 75·8 (44·7–104·4) | 22·4 (11·8–36·3) | 62·1 (30·3–94·4) | 182 (138–231) | 112·1 (89·5–133·3) | 143 (110–178) | 143·0 (118·4–166·6) |  |
|  | Czechia | 371 (277–450) | 111·2 (73·2–144·5) | 98 (64·3–142) | 92·8 (52·8–134·7) | 338 (263–438) | 154·6 (117·9–201·2) | 170 (121–229) | 214·9 (167·7–265·7) |  |
|  | Hungary | 216 (150–280) | 72·2 (50·2–92·5) | 49 (30·1–74·3) | 74·5 (48·3–97·2) | 235 (173–307) | 82·7 (65·6–100·2) | 184 (136–236) | 105·3 (82·8–128·7) |  |
|  | Montenegro | 20·6 (13·5–28·4) | 103·7 (74·8–130·8) | 4·23 (2·42–6·76) | 102·9 (69·6–137·2) | 24·9 (19·3–32·5) | 133·9 (113·6–157·8) | 19·3 (14·7–24·7) | 164·7 (139·8–192·1) |  |
|  | North Macedonia | 69·4 (47·8–89·8) | 77·3 (53·9–98·7) | 16·4 (10·4–24·3) | 59·8 (32·4–81·7) | 66·7 (50·1–87) | 121·2 (98·4–147·5) | 50·2 (36·6–65·6) | 147·2 (121·3–172·3) |  |
|  | Poland | 991 (603–1460) | 20·6 (-0·1–40·1) | 148 (73·7–252) | 16·9 (-5·8–36·1) | 1640 (1270–2060) | 41·8 (29·7–52·4) | 1850 (1500–2220) | 49·5 (37·0–61·6) |  |
|  | Romania | 391 (271–492) | 44·8 (24·3–64·4) | 89·5 (53·7–135) | 34·6 (14·1–53·3) | 379 (296–478) | 74·1 (54·9–94·7) | 283 (210–368) | 97·1 (75·3–121·3) |  |
|  | Serbia | 256 (174–331) | 34·4 (17·7–48·4) | 64·1 (39·4–94·3) | 18·5 (2·8–33·1) | 240 (182–314) | 61·4 (46·7–75·2) | 183 (139–241) | 83·5 (66·2–103·5) |  |
|  | Slovakia | 104 (70·5–135) | 69·4 (44·7–86·8) | 21·9 (13·1–32·5) | 53·6 (30·4–75·1) | 112 (85·6–145) | 101·8 (84·6–119·3) | 84·4 (65·8–107) | 123·0 (100·7–144·1) |  |
|  | Slovenia | 36·8 (22·1–55·8) | 51·4 (13·6–95·5) | 5·14 (2·57–8·94) | 22·8 (-13·8–62·3) | 74·7 (59·1–91·5) | 115·8 (91·9–140·4) | 59·1 (45·5–72·2) | 147·7 (117·1–177·9) |  |
|  | Eastern Europe | 3960 (2900–5050) | 56·1 (43·0–70·7) | 596 (372–891) | 59·5 (43·7–75·5) | 3760 (2830–4760) | 106·8 (94·2–120·8) | 4640 (3740–5700) | 177·2 (161·9–193·6) |  |
|  | Belarus | 139 (110–170) | 29·4 (3·3–64·5) | 55·3 (39–73·7) | -3·5 (-28·0–31·9) | 166 (133–202) | 140·6 (94·4–196·1) | 252 (211–296) | 149·3 (99·4–203·0) |  |
|  | Estonia | 27·8 (18–37·6) | 38·4 (9·4–65·7) | 4·31 (2·33–6·92) | 3·7 (-23·6–30·8) | 34·9 (27·3–43·6) | 125·3 (97·3–155·9) | 35·6 (28·7–43·8) | 149·6 (119·1–186·5) |  |
|  | Latvia | 55·8 (39–70·8) | 72·1 (49·5–97·4) | 11·7 (6·91–17·2) | 61·9 (34·6–87·3) | 50·6 (38·9–64·1) | 113·3 (91·7–139·6) | 51·2 (39·6–64·3) | 135·0 (107·1–164·7) |  |
|  | Lithuania | 65·7 (45·8–85·9) | 82·5 (37·8–119·2) | 9·61 (5·44–14·9) | 75·9 (22·5–123·7) | 80·9 (62·5–102) | 157·0 (117·7–199·4) | 54·7 (41·7–68·9) | 186·0 (144·7–237·0) |  |
|  | Moldova | 123 (103–140) | 81·8 (59·4–104·5) | 35·4 (27·2–46·3) | 70·3 (46·6–94·7) | 73·2 (58·4–91·8) | 119·7 (87·2–151·0) | 61·4 (49–77·1) | 136·3 (100·1–172·3) |  |
|  | Russia | 2710 (1860–3660) | 65·2 (42·5–87·7) | 252 (143–405) | 62·7 (36·9–86·2) | 2840 (2120–3630) | 131·4 (116·5–146·4) | 3710 (2980–4550) | 228·8 (211·2–250·5) |  |
|  | Ukraine | 851 (680–1030) | 33·6 (23·8–44·2) | 211 (148–289) | 82·8 (65·0–104·6) | 513 (387–677) | 22·5 (13·7–33·0) | 472 (360–598) | 31·2 (21·8–43·0) |  |
|  | **High income** | **20400 (14000–27000)** | **30·4 (16·0–43·8)** | **6670 (4060–9770)** | **38·3 (21·6–53·4)** | **40000 (32800–47700)** | **81·4 (74·8–87·9)** | **32600 (26800–38300)** | **105·8 (97·5–113·5)** |  |
|  | Australasia | 428 (309–537) | 63·4 (30·6–87·2) | 141 (93·5–201) | 53·9 (16·0–85·1) | 592 (477–736) | 120·9 (96·2–148·4) | 295 (221–376) | 164·6 (130·4–200·3) |  |
|  | Australia | 329 (232–420) | 50·7 (16·2–76·4) | 106 (69·4–151) | 43·9 (3·7–77·4) | 457 (363–568) | 106·4 (78·9–137·6) | 231 (171–296) | 149·3 (109·5–189·2) |  |
|  | New Zealand | 100 (75·2–123) | 125·8 (103·8–145·3) | 34·7 (22–48·7) | 98·2 (76·6–122·8) | 134 (107–166) | 191·4 (170·2–213·0) | 64·1 (48·2–81·1) | 240·9 (212·5–273·5) |  |
|  | High-income Asia Pacific | 4770 (3510–6170) | -4·5 (-16·3–8·2) | 509 (331–743) | -26·0 (-37·4–-12·8) | 6940 (5770–8250) | 56·4 (45·2–68·7) | 7160 (6100–8320) | 87·1 (71·7–102·2) |  |
|  | Brunei | 10·4 (7·91–13·5) | 118·7 (75·6–155·3) | 2·67 (1·76–3·79) | 119·5 (66·6–175·0) | 16·4 (13·5–19·1) | 233·8 (188·1–292·4) | 10·1 (7·8–12·1) | 263·3 (213·1–321·7) |  |
|  | Japan | 3500 (2260–4840) | -7·9 (-23·8–7·0) | 366 (206–565) | -17·2 (-34·9–0·8) | 4870 (3820–6110) | 29·4 (20·3–39·7) | 5120 (4130–6230) | 58·9 (45·1–71·2) |  |
|  | South Korea | 1170 (1060–1280) | 6·1 (-4·2–17·3) | 129 (105–162) | -47·6 (-55·1–-39·7) | 1870 (1750–2000) | 208·3 (182·7–238·5) | 1940 (1830–2060) | 238·3 (210·5–269·0) |  |
|  | Singapore | 95·1 (67·6–123) | 12·6 (-17·2–39·6) | 10·7 (6·55–16·2) | -12·5 (-39·9–15·7) | 170 (139–205) | 164·3 (130·5–206·6) | 97·2 (75·9–119) | 191·2 (151·0–237·7) |  |
|  | High-income North America | 7300 (4450–10300) | 75·1 (55·6–94·8) | 2670 (1490–4240) | 136·7 (103·2–167·2) | 17400 (14200–21200) | 119·9 (107·0–131·7) | 15400 (12500–18100) | 148·8 (133·3–161·8) |  |
|  | Canada | 339 (196–498) | 87·3 (35·7–142·9) | 73·4 (36·7–130) | 102·2 (34·7–167·6) | 1110 (928–1320) | 128·4 (106·3–154·8) | 899 (730–1070) | 131·4 (107·7–153·7) |  |
|  | Greenland | 0·667 (0·463–0·876) | 72·8 (40·6–103·5) | 0·3 (0·192–0·438) | 46·6 (13·3–78·1) | 0·844 (0·616–1·07) | 161·4 (131·0–196·3) | 0·615 (0·464–0·775) | 172·9 (136·4–217·1) |  |
|  | USA | 6950 (4240–9800) | 74·5 (55·0–94·5) | 2600 (1450–4130) | 138·3 (104·4–168·4) | 16300 (13200–19900) | 119·4 (106·2–132·2) | 14500 (11700–17100) | 150·0 (134·5–163·6) |  |
|  | Southern Latin America | 617 (457–781) | 54·6 (27·8–80·4) | 203 (135–284) | 44·0 (17·8–73·2) | 845 (683–1010) | 114·0 (90·7–148·8) | 1410 (1220–1600) | 124·9 (103·1–146·8) |  |
|  | Argentina | 451 (333–576) | 55·2 (22·3–93·2) | 143 (97–203) | 46·8 (12·1–87·6) | 599 (482–722) | 97·8 (70·3–138·1) | 1050 (901–1190) | 105·5 (81·6–129·9) |  |
|  | Chile | 112 (78·5–148) | 62·8 (27·3–104·6) | 45·7 (28·7–66·6) | 36·0 (-1·1–70·2) | 208 (169–252) | 202·1 (167·6–241·6) | 290 (246–334) | 278·8 (240·3–323·2) |  |
|  | Uruguay | 56·2 (44·5–68·2) | 47·4 (33·0–64·8) | 13·3 (9·36–18·6) | 33·6 (12·7–53·9) | 37·3 (28·7–45·7) | 64·6 (47·6–84·7) | 71·6 (60·3–85·5) | 81·4 (63·8–100·5) |  |
|  | Western Europe | 7410 (5210–9510) | 25·4 (9·4–38·2) | 3030 (1870–4280) | 14·2 (-3·3–28·1) | 14200 (11600–17000) | 57·7 (52·5–63·8) | 8330 (6570–10200) | 63·5 (57·3–70·7) |  |
|  | Andorra | 0·958 (0·615–1·32) | 77·5 (49·0–108·9) | 0·247 (0·136–0·391) | 79·4 (47·5–118·2) | 2·28 (1·86–2·73) | 114·3 (97·6–132·9) | 1·27 (0·996–1·58) | 124·8 (105·8–145·1) |  |
|  | Austria | 90·9 (60–125) | 22·4 (-0·4–40·5) | 26·9 (14·4–41·6) | 9·9 (-12·3–31·4) | 199 (164–241) | 56·5 (43·9–69·2) | 115 (88·7–142) | 65·4 (51·0–80·5) |  |
|  | Belgium | 151 (95·7–204) | 25·6 (8·5–43·6) | 46·4 (27–69·4) | 13·5 (-7·3–36·7) | 301 (242–366) | 56·2 (41·3–70·9) | 173 (133–215) | 64·3 (48·3–79·9) |  |
|  | Cyprus | 22·3 (14·8–29·9) | 40·6 (12·6–67·6) | 7·1 (3·98–10·7) | -2·4 (-25·6–19·7) | 43·8 (35·4–53·4) | 155·9 (126·6–185·9) | 24·4 (19·4–30·3) | 167·5 (137·4–198·8) |  |
|  | Denmark | 75·8 (49·3–104) | 39·0 (12·5–66·8) | 23·3 (13·3–35·5) | 7·9 (-18·9–31·9) | 153 (124–184) | 111·9 (88·7–136·0) | 88·8 (69·2–110) | 131·1 (102·6–159·7) |  |
|  | Finland | 75·1 (46·4–110) | 20·5 (-17·0–61·8) | 22·8 (11·8–39·5) | 4·6 (-32·8–54·1) | 296 (246–346) | 61·8 (44·8–80·0) | 176 (140–210) | 78·0 (58·1–99·6) |  |
|  | France | 1230 (888–1610) | 17·8 (-11·6–49·6) | 342 (219–502) | -5·3 (-33·4–26·5) | 1820 (1490–2230) | 88·2 (60·3–118·1) | 1050 (807–1330) | 99·1 (68·7–134·5) |  |
|  | Germany | 1570 (1030–2080) | 16·2 (0·9–31·2) | 548 (316–810) | 7·2 (-10·2–25·9) | 3020 (2400–3680) | 34·3 (21·7–47·1) | 1790 (1360–2190) | 40·7 (25·9–55·5) |  |
|  | Greece | 178 (119–232) | 44·9 (24·9–64·0) | 71·8 (44·3–102) | 71·5 (43·5–98·4) | 301 (242–367) | 43·5 (30·7–58·1) | 181 (138–226) | 53·6 (39·6–69·2) |  |
|  | Iceland | 4·16 (2·71–5·77) | 95·1 (60·6–127·6) | 1·14 (0·628–1·77) | 90·1 (44·6–132·8) | 9 (7·42–11) | 127·2 (106·8–148·5) | 5·24 (4·14–6·39) | 138·4 (115·0–161·2) |  |
|  | Ireland | 51·8 (34·3–73·3) | 79·3 (41·4–118·0) | 14·5 (8·12–22·7) | 34·8 (-2·8–69·6) | 115 (94·5–141) | 210·3 (175·9–248·4) | 66·8 (52·6–82·4) | 230·4 (193·4–272·1) |  |
|  | Israel | 103 (68·6–137) | 53·8 (28·6–76·3) | 33·5 (19–48·3) | 19·6 (-4·4–42·7) | 190 (153–228) | 119·2 (99·5–145·1) | 111 (87·3–138) | 123·7 (99·6–145·0) |  |
|  | Italy | 892 (561–1260) | 14·9 (3·5–25·8) | 256 (146–403) | 13·6 (3·1–23·6) | 2030 (1660–2490) | 30·5 (24·8–36·6) | 1180 (905–1460) | 39·3 (33·4–46·3) |  |
|  | Luxembourg | 8·02 (5·41–10·5) | 58·6 (16·1–94·9) | 2·27 (1·33–3·43) | 31·4 (-12·1–68·1) | 14·4 (11·5–17·8) | 125·6 (99·4–153·2) | 8·1 (6·21–10·3) | 134·5 (105·9–163·8) |  |
|  | Malta | 8·23 (5·43–11·3) | 67·4 (39·5–97·2) | 2·68 (1·59–3·96) | 46·9 (17·9–77·6) | 15·9 (12·8–19·4) | 119·0 (93·2–145·8) | 9·37 (7·33–11·7) | 138·6 (112·8–170·4) |  |
|  | Monaco | 0·617 (0·403–0·852) | 59·5 (34·7–83·1) | 0·227 (0·129–0·334) | 51·2 (24·9–75·4) | 1·22 (0·966–1·55) | 88·1 (73·0–103·5) | 0·75 (0·583–0·936) | 96·5 (79·7–114·6) |  |
|  | Netherlands | 194 (124–273) | 9·1 (-12·4–31·4) | 54·9 (30·4–88·4) | -9·6 (-30·0–10·9) | 447 (369–537) | 50·1 (37·1–62·8) | 262 (207–321) | 61·1 (46·9–77·3) |  |
|  | Norway | 80·5 (52·5–114) | 36·9 (23·2–50·4) | 8·25 (4·29–13·9) | 9·5 (-3·1–23·0) | 179 (147–215) | 48·0 (42·3–54·3) | 109 (85·2–134) | 54·0 (45·6–60·6) |  |
|  | Portugal | 110 (63·3–163) | 6·5 (-39·8–56·6) | 38·3 (18·4–65) | -16·1 (-57·4–33·8) | 499 (420–574) | 55·5 (38·1–74·4) | 297 (237–351) | 69·3 (48·2–88·9) |  |
|  | San Marino | 0·496 (0·302–0·71) | 115·6 (80·3–155·9) | 0·146 (0·0793–0·231) | 135·9 (93·8–190·8) | 1·24 (1·03–1·51) | 143·7 (127·7–161·1) | 0·744 (0·587–0·921) | 155·9 (134·4–179·7) |  |
|  | Spain | 737 (552–952) | 28·8 (2·2–56·1) | 999 (705–1300) | 11·7 (-12·6–34·3) | 1570 (1260–1940) | 73·8 (50·8–101·7) | 947 (725–1190) | 80·4 (58·8–104·6) |  |
|  | Sweden | 165 (110–229) | 40·9 (17·6–66·2) | 41·2 (22·1–65·5) | 21·4 (-6·2–49·8) | 397 (330–472) | 75·9 (62·1–91·9) | 230 (183–284) | 83·4 (67·7–99·5) |  |
|  | Switzerland | 156 (114–194) | 22·6 (-3·1–44·7) | 41·4 (27·4–58·8) | 3·3 (-18·9–32·1) | 186 (149–230) | 67·8 (43·4–94·1) | 132 (99·9–165) | 74·0 (52·2–100·3) |  |
|  | UK | 1470 (1060–1860) | 52·6 (41·7–61·8) | 481 (303–682) | 63·1 (48·0–76·2) | 2400 (1950–2910) | 86·7 (78·2–94·3) | 1360 (1070–1670) | 73·7 (65·4–81·6) |  |
|  | **Latin America and Caribbean** | **17100 (13400–20900)** | **68·8 (53·2–82·3)** | **2540 (1660–3570)** | **102·3 (79·3–122·2)** | **15900 (12600–19500)** | **165·0 (152·8–177·5)** | **11400 (8980–14100)** | **210·2 (195·1–225·3)** |  |
|  | Andean Latin America | 1270 (1080–1460) | 144·9 (130·5–158·4) | 301 (221–390) | 117·7 (99·8–134·9) | 806 (642–986) | 243·6 (219·9–267·6) | 606 (491–731) | 304·1 (275·7–330·0) |  |
|  | Bolivia | 285 (244–332) | 143·9 (127·5–158·3) | 73·6 (55·1–95·5) | 103·2 (85·9–121·4) | 127 (95·9–166) | 227·9 (202·3–251·4) | 87·5 (65·9–112) | 276·5 (248·8–309·0) |  |
|  | Ecuador | 422 (345–495) | 125·6 (102·6–156·3) | 51·3 (35·4–70·7) | 127·9 (93·9–168·8) | 349 (279–419) | 213·2 (163·9–264·6) | 281 (228–336) | 271·0 (214·4–329·5) |  |
|  | Peru | 563 (473–654) | 163·1 (136·7–188·8) | 180 (134–230) | 121·7 (94·3–152·5) | 329 (252–413) | 293·5 (255·8–329·8) | 238 (187–292) | 365·8 (323·2–414·7) |  |
|  | Caribbean | 1420 (1150–1700) | 64·1 (56·3–72·0) | 223 (159–307) | 77·8 (67·5–90·6) | 1320 (1050–1570) | 88·8 (78·9–97·6) | 919 (740–1110) | 115·4 (103·4–127·2) |  |
|  | Antigua and Barbuda | 3·38 (2·54–4·21) | 62·5 (44·5–81·5) | 0·467 (0·289–0·699) | 85·6 (59·2–117·9) | 3·82 (3·08–4·64) | 98·6 (81·9–116·8) | 2·64 (2·11–3·26) | 127·6 (109·4–147·7) |  |
|  | The Bahamas | 16·1 (12·5–19·2) | 102·5 (85·6–123·6) | 2·74 (1·86–3·83) | 139·1 (108·8–177·3) | 13·6 (10·7–16·8) | 146·8 (124·9–175·3) | 9·16 (7·04–11·6) | 183·6 (161·0–216·1) |  |
|  | Barbados | 15·3 (11·4–19·1) | 55·3 (36·3–74·5) | 2·77 (1·77–4·15) | 70·2 (42·5–98·6) | 18·7 (15–22·8) | 82·9 (64·6–104·3) | 14·1 (11·6–17) | 112·0 (91·8–137·6) |  |
|  | Belize | 14·8 (12–17·4) | 167·8 (141·8–192·8) | 2·84 (1·96–3·87) | 161·2 (128·3–198·0) | 11·1 (8·86–13·7) | 272·1 (240·4–305·1) | 7·81 (6·03–9·62) | 304·3 (268·5–342·8) |  |
|  | Bermuda | 1·23 (0·842–1·68) | 16·7 (-3·4–36·5) | 0·112 (0·0662–0·181) | 28·9 (-1·2–61·4) | 2·67 (2·18–3·17) | 59·4 (43·2–72·4) | 2·22 (1·85–2·61) | 100·8 (80·3–121·8) |  |
|  | Cuba | 213 (153–272) | 28·1 (10·5–45·1) | 22 (13·3–33·4) | 50·5 (20·5–79·8) | 328 (269–390) | 78·5 (59·7–96·5) | 235 (188–281) | 111·9 (87·4–137·6) |  |
|  | Dominica | 3·75 (3–4·55) | 63·7 (50·4–76·4) | 0·901 (0·603–1·22) | 94·8 (74·5–118·8) | 2·88 (2·21–3·58) | 67·5 (55·6–82·7) | 2·12 (1·65–2·69) | 90·2 (75·4–107·3) |  |
|  | Dominican Republic | 368 (303–433) | 121·6 (105·8–138·9) | 61·9 (42·1–85·8) | 156·1 (129·4–184·6) | 266 (207–329) | 164·5 (144·0–184·1) | 170 (132–214) | 195·5 (174·3–219·4) |  |
|  | Grenada | 5·87 (4·6–7·2) | 57·4 (41·6–72·4) | 1·09 (0·733–1·57) | 90·3 (66·8–119·1) | 5·46 (4·34–6·78) | 76·8 (61·8–91·1) | 3·86 (3·04–4·7) | 102·0 (84·0–120·7) |  |
|  | Guyana | 24·8 (19·9–29·8) | 24·8 (11·1–39·9) | 4·86 (3·34–6·65) | 33·5 (12·8–60·7) | 31·5 (25·4–37·4) | 74·0 (57·1–96·0) | 19·6 (15·7–24·3) | 101·3 (79·7–127·7) |  |
|  | Haiti | 339 (285–387) | 104·8 (92·3–116·8) | 63·5 (46·3–84·7) | 91·3 (72·4–109·6) | 152 (119–191) | 130·5 (112·3–149·3) | 80·2 (59·3–103) | 146·2 (124·9–165·7) |  |
|  | Jamaica | 103 (79·9–126) | 63·4 (50·1–76·6) | 15·7 (10·3–22·5) | 81·9 (58·8–107·0) | 96·5 (75·7–118) | 79·7 (64·5–97·4) | 66·9 (52·6–81·7) | 100·2 (83·0–120·8) |  |
|  | Puerto Rico | 109 (73·8–148) | -9·8 (-27·6–6·9) | 13·8 (8·01–21·9) | -12·4 (-32·9–8·9) | 206 (171–244) | 34·4 (21·8–46·7) | 171 (141–203) | 63·7 (46·2–80·5) |  |
|  | Saint Kitts and Nevis | 2·03 (1·58–2·46) | 48·1 (31·4–67·5) | 0·409 (0·274–0·596) | 74·3 (47·2–105·5) | 1·81 (1·4–2·26) | 92·4 (70·9–115·8) | 1·24 (0·951–1·53) | 111·8 (84·7–142·0) |  |
|  | Saint Lucia | 12·2 (10·5–13·7) | 78·5 (63·6–98·1) | 1·94 (1·45–2·59) | 98·6 (72·2–130·8) | 6·67 (5·29–8·03) | 99·1 (72·1–129·8) | 4·75 (3·81–5·62) | 117·8 (89·6–150·5) |  |
|  | Saint Vincent and the Grenadines | 5·92 (4·58–7·26) | 52·5 (37·9–64·5) | 1·02 (0·67–1·43) | 77·1 (52·0–97·8) | 5·52 (4·32–6·77) | 83·3 (68·4–98·6) | 3·79 (2·95–4·64) | 111·0 (94·5–128·2) |  |
|  | Suriname | 31·7 (25·4–37·8) | 122·2 (101·1–143·1) | 5·76 (4·08–7·99) | 140·4 (110·0–170·6) | 23·6 (18·3–29·6) | 194·1 (168·0–224·1) | 14·6 (11·1–18·5) | 229·5 (198·3–262·4) |  |
|  | Trinidad and Tobago | 97·9 (74·7–122) | 38·5 (21·9–60·1) | 18·3 (12·1–26·3) | 36·7 (14·1–64·9) | 100 (78·8–122) | 83·9 (63·2–113·4) | 71·3 (55·6–86·1) | 112·0 (88·8–146·9) |  |
|  | Virgin Islands | 3·28 (2·34–4·23) | 0·4 (-14·1–15·1) | 0·535 (0·318–0·813) | -9·9 (-27·6–9·1) | 4·57 (3·67–5·57) | 58·0 (41·9–75·0) | 3·62 (2·88–4·42) | 95·9 (74·5–122·7) |  |
|  | Central Latin America | 9310 (7210–11400) | 63·4 (47·9–77·2) | 1210 (805–1720) | 111·9 (84·8–137·4) | 8760 (6920–10700) | 161·9 (147·6–175·9) | 6170 (4850–7530) | 237·5 (216·7–256·3) |  |
|  | Colombia | 1110 (782–1400) | 82·3 (47·8–110·4) | 89 (54–135) | 66·1 (25·2–103·6) | 1050 (833–1290) | 199·9 (172·3–232·6) | 1150 (925–1360) | 251·2 (216·5–291·2) |  |
|  | Costa Rica | 169 (123–216) | 120·2 (76·8–166·5) | 18·1 (11·3–27·4) | 130·2 (69·7–199·6) | 183 (145–225) | 226·6 (180·2–275·1) | 139 (112–170) | 267·5 (211·3–321·0) |  |
|  | El Salvador | 195 (154–231) | 67·4 (49·9–84·6) | 23·9 (15·7–32·9) | 67·2 (47·2–89·0) | 133 (103–166) | 122·6 (101·7–142·6) | 103 (78·8–126) | 139·3 (117·5–165·3) |  |
|  | Guatemala | 671 (601–742) | 198·9 (174·2–230·1) | 99·5 (75·2–128) | 190·7 (159·5–232·8) | 289 (239–339) | 293·5 (239·2–354·3) | 214 (173–256) | 325·6 (268·0–393·7) |  |
|  | Honduras | 250 (209–289) | 163·2 (145·1–178·0) | 33·9 (24·2–45·2) | 155·0 (131·2–178·3) | 110 (85·2–140) | 229·8 (208·7–252·8) | 74·3 (56·8–92·8) | 246·9 (224·2–271·5) |  |
|  | Mexico | 5710 (4290–7130) | 43·1 (25·7–57·8) | 796 (502–1160) | 102·4 (69·7–130·6) | 6200 (4900–7660) | 152·3 (135·2–168·6) | 3790 (2940–4680) | 241·3 (217·2–264·4) |  |
|  | Nicaragua | 141 (112–166) | 128·4 (104·3–147·1) | 15 (9·84–20·9) | 120·0 (91·0–149·0) | 97·7 (75·7–122) | 229·5 (200·7–251·8) | 72·3 (56·9–90·5) | 251·6 (220·6–279·9) |  |
|  | Panama | 111 (79·6–140) | 112·5 (84·8–137·2) | 8·73 (5·36–12·9) | 120·4 (85·3–156·8) | 123 (95·9–148) | 210·1 (185·7–242·8) | 91·6 (70·9–109) | 241·0 (213·6–277·1) |  |
|  | Venezuela | 947 (760–1120) | 103·4 (83·0–125·4) | 128 (85·4–179) | 171·9 (132·4–218·2) | 628 (474–787) | 147·2 (124·3–170·3) | 487 (361–606) | 169·5 (142·2–196·5) |  |
|  | Tropical Latin America | 5060 (3780–6380) | 67·4 (46·9–84·8) | 817 (505–1180) | 89·7 (63·8–112·6) | 5000 (3870–6290) | 186·4 (170·8–201·2) | 3730 (2920–4650) | 199·1 (181·8–217·2) |  |
|  | Brazil | 4870 (3620–6150) | 65·5 (44·4–83·2) | 785 (486–1140) | 87·7 (61·7–111·0) | 4850 (3760–6120) | 186·7 (170·5–201·6) | 3630 (2840–4520) | 199·9 (181·7–218·1) |  |
|  | Paraguay | 187 (144–226) | 138·1 (118·5–156·4) | 32·8 (21–45·5) | 157·3 (131·7–188·6) | 144 (110–182) | 176·7 (153·5–199·5) | 103 (79·8–128) | 174·5 (154·4–196·4) |  |
|  | **North Africa and Middle East** | **23000 (18800–27200)** | **177·6 (158·7–195·3)** | **3240 (2330–4430)** | **163·2 (137·8–189·9)** | **22100 (18500–25900)** | **303·6 (281·9–322·5)** | **18100 (14900–22200)** | **356·8 (331·2–381·6)** |  |
|  | Afghanistan | 1180 (1030–1340) | 192·3 (161·7–219·6) | 60·4 (43·8–83·9) | 125·7 (81·3–175·0) | 420 (331–519) | 239·5 (188·2–310·4) | 360 (286–455) | 286·6 (223·7–350·6) |  |
|  | Algeria | 1720 (1450–1980) | 150·4 (124·4–175·9) | 217 (156–297) | 208·5 (157·8–261·5) | 1710 (1430–2000) | 397·8 (341·1–462·9) | 1220 (1000–1470) | 349·9 (289·3–409·8) |  |
|  | Bahrain | 95·4 (65·7–124) | 292·0 (222·1–360·8) | 11·6 (6·84–17·7) | 249·3 (165·4–336·3) | 112 (87·8–142) | 630·9 (540·5–730·2) | 88 (67·5–114) | 715·2 (632·3–822·1) |  |
|  | Egypt | 4660 (3960–5360) | 285·0 (237·0–320·8) | 816 (615–1080) | 269·2 (211·0–324·2) | 3800 (3120–4450) | 220·1 (190·3–260·0) | 2920 (2420–3600) | 260·9 (227·5–301·4) |  |
|  | Iran | 2180 (1590–2800) | 151·9 (118·6–178·0) | 749 (480–1060) | 187·2 (151·7–218·8) | 2810 (2230–3470) | 348·2 (326·4–371·3) | 2150 (1700–2760) | 285·6 (265·6–307·2) |  |
|  | Iraq | 1780 (1440–2100) | 187·8 (151·7–229·0) | 159 (113–219) | 95·4 (55·0–139·9) | 1620 (1360–1880) | 454·3 (368·8–549·9) | 931 (752–1130) | 510·4 (411·8–625·7) |  |
|  | Jordan | 299 (234–377) | 215·5 (160·0–288·9) | 54·9 (34·9–78·3) | 133·7 (72·0–231·4) | 596 (514–681) | 616·2 (519·7–709·5) | 458 (381–533) | 870·6 (738·1–988·6) |  |
|  | Kuwait | 234 (161–313) | 389·8 (282·4–507·8) | 35 (20·4–52·6) | 432·4 (270·1–597·6) | 436 (369–514) | 525·1 (434·1–611·9) | 222 (175–283) | 797·9 (633·9–993·2) |  |
|  | Lebanon | 364 (310–412) | 157·8 (137·3–184·1) | 21·5 (13·5–31·8) | 81·7 (50·0–114·8) | 178 (145–215) | 260·9 (216·2–311·4) | 184 (149–220) | 293·4 (237·6–342·7) |  |
|  | Libya | 446 (347–535) | 265·5 (215·6–308·4) | 86·8 (57·9–121) | 451·7 (342·3–550·0) | 298 (232–375) | 304·9 (251·5–369·7) | 197 (147–255) | 354·0 (294·5–428·4) |  |
|  | Morocco | 1740 (1490–1990) | 136·6 (116·0–160·5) | 234 (177–305) | 112·5 (80·2–146·6) | 1240 (1020–1460) | 261·0 (208·5–316·8) | 704 (568–847) | 299·0 (241·1–367·8) |  |
|  | Oman | 176 (127–223) | 357·1 (296·2–409·5) | 19·8 (12·3–29·6) | 397·0 (306·4–490·3) | 158 (127–205) | 474·4 (418·2–537·1) | 126 (93·1–162) | 572·6 (512·7–650·2) |  |
|  | Palestine | 139 (95·9–179) | 183·8 (114·3–251·7) | 14·9 (8·27–23·3) | 115·3 (33·0–208·9) | 217 (178–263) | 350·0 (293·7–410·5) | 101 (78·2–130) | 407·2 (337·3–494·5) |  |
|  | Qatar | 110 (71·2–151) | 504·5 (351·1–652·3) | 16·5 (8·34–27·1) | 309·1 (181·6–455·3) | 153 (118–191) | 916·1 (768·2–1070·2) | 171 (131–224) | 1108·9 (949·8–1285·2) |  |
|  | Saudi Arabia | 2120 (1420–2820) | 188·0 (114·0–243·6) | 73·2 (38·3–112) | 156·0 (58·3–255·6) | 1760 (1410–2130) | 220·1 (163·2–268·3) | 2720 (2260–3310) | 520·3 (428·6–669·5) |  |
|  | Sudan | 1030 (870–1170) | 146·5 (123·9–170·7) | 138 (98·4–182) | 119·9 (86·2–157·0) | 933 (788–1090) | 255·3 (207·8–305·6) | 383 (302–473) | 306·6 (253·7–368·2) |  |
|  | Syria | 718 (505–915) | 142·0 (105·4–174·7) | 104 (62·8–156) | 204·1 (141·8–260·5) | 779 (619–977) | 289·3 (251·3–330·2) | 593 (462–751) | 333·6 (296·2–380·4) |  |
|  | Tunisia | 361 (233–494) | 79·7 (42·9–112·7) | 35·3 (19–56·9) | 80·0 (35·1–122·6) | 632 (509–770) | 205·4 (177·5–238·3) | 450 (365–557) | 231·7 (200·0–268·2) |  |
|  | Türkiye | 2310 (1560–2980) | 72·9 (42·2–102·4) | 298 (175–469) | 35·6 (-0·6–68·7) | 3140 (2520–3880) | 269·2 (225·9–312·7) | 2990 (2380–3610) | 309·8 (262·4–356·0) |  |
|  | United Arab Emirates | 501 (300–690) | 439·2 (286·3–562·1) | 91·2 (46·6–141) | 857·4 (561·4–1125·6) | 618 (501–751) | 998·8 (823·8–1197·1) | 872 (699–1100) | 1097·3 (937·5–1283·2) |  |
|  | Yemen | 734 (593–885) | 446·9 (406·6–488·9) | 82·4 (55·9–113) | 380·7 (329·0–435·5) | 472 (371–596) | 599·5 (549·8–658·3) | 294 (219–378) | 638·7 (581·6–702·7) |  |
|  | **South Asia** | **74500 (62200–86600)** | **107·8 (95·2–119·1)** | **2480 (1820–3360)** | **41·0 (28·3–51·3)** | **36600 (28800–45500)** | **273·7 (259·4–288·9)** | **19100 (14400–24000)** | **269·8 (253·0–287·6)** |  |
|  | Bangladesh | 5090 (4530–5650) | 130·2 (109·2–152·1) | 435 (339–548) | 77·4 (47·3–108·6) | 2900 (2500–3310) | 235·1 (180·0–295·2) | 1950 (1640–2290) | 169·8 (119·8–231·6) |  |
|  | Bhutan | 16·9 (13·8–19·6) | 47·4 (27·7–67·3) | 1·8 (1·24–2·46) | 25·3 (1·3–51·4) | 7·12 (5·56–8·83) | 185·8 (140·5–249·1) | 12·1 (9·81–14·6) | 275·6 (224·8–347·0) |  |
|  | India | 60100 (49700–70600) | 104·3 (88·8–117·7) | 1270 (916–1780) | 30·7 (16·7–43·9) | 30200 (23600–38000) | 290·1 (273·5–305·3) | 15000 (11100–19000) | 308·3 (289·1–329·7) |  |
|  | Nepal | 1030 (915–1170) | 105·4 (90·9–120·3) | 134 (98·4–177) | 72·4 (53·6–95·4) | 404 (332–491) | 179·3 (145·7–214·1) | 190 (145–242) | 205·7 (168·6–241·0) |  |
|  | Pakistan | 8160 (7000–9530) | 119·8 (109·4–129·4) | 721 (533–974) | 66·5 (53·3–78·9) | 2960 (2270–3800) | 199·1 (185·0–216·4) | 2040 (1560–2610) | 178·8 (159·7–196·2) |  |
|  | **Southeast Asia, east Asia, and Oceania** | **81500 (69700–95000)** | **40·4 (28·2–51·9)** | **9010 (6630–12100)** | **-5·4 (-14·5–5·2)** | **39100 (30900–48900)** | **156·6 (143·0–170·5)** | **24700 (18900–30900)** | **155·5 (143·0–166·7)** |  |
|  | East Asia | 62000 (53300–71900) | 29·6 (18·1–40·1) | 6910 (5070–9290) | -16·2 (-24·5–-6·5) | 28800 (22700–36100) | 145·5 (131·0–159·8) | 15800 (11900–20000) | 130·1 (117·4–141·5) |  |
|  | China | 59900 (51600–69500) | 29·1 (17·6–39·8) | 6670 (4890–8970) | -17·3 (-25·4–-7·7) | 27700 (21800–34800) | 146·8 (131·9–161·5) | 15100 (11400–19200) | 130·1 (116·8–142·1) |  |
|  | North Korea | 1050 (895–1200) | 59·3 (49·4–68·4) | 147 (112–196) | 29·0 (15·9–41·5) | 355 (281–451) | 120·5 (101·8–136·8) | 198 (151–253) | 125·3 (106·9–145·0) |  |
|  | Taiwan | 980 (778–1160) | 32·8 (16·6–48·2) | 93·2 (62·3–131) | 29·3 (11·7–49·5) | 732 (565–899) | 114·6 (93·7–138·2) | 475 (361–589) | 130·8 (107·2–157·9) |  |
|  | Oceania | 560 (474–640) | 128·0 (115·1–139·8) | 82·9 (59·5–109) | 133·4 (116·4–153·4) | 289 (234–356) | 161·9 (146·3–178·3) | 93 (68·3–121) | 171·0 (148·9–195·7) |  |
|  | American Samoa | 3·96 (3·57–4·37) | 18·7 (7·4–29·6) | 0·662 (0·546–0·8) | 73·0 (43·7–106·8) | 2·43 (2·11–2·72) | 64·8 (39·4–90·1) | 1·44 (1·25–1·65) | 106·3 (72·6–147·0) |  |
|  | Cook Islands | 1·51 (1·28–1·73) | -4·7 (-13·3–3·4) | 0·191 (0·139–0·251) | -9·8 (-22·0–2·4) | 0·72 (0·573–0·872) | 38·3 (25·0–52·9) | 0·393 (0·298–0·496) | 64·2 (46·5–86·9) |  |
|  | Fiji | 61·9 (47·9–74·2) | 90·5 (62·3–119·6) | 17·5 (11·7–24·4) | 133·5 (88·4–181·5) | 56·9 (44·7–71·6) | 123·3 (92·3–158·0) | 22·2 (16·1–28·8) | 138·8 (103·7–178·9) |  |
|  | Guam | 5·52 (4·25–6·78) | 39·1 (24·9–54·5) | 0·557 (0·347–0·791) | 71·1 (45·5–95·1) | 5·9 (4·83–7·15) | 84·3 (66·5–102·6) | 2·11 (1·58–2·68) | 105·5 (83·8–126·0) |  |
|  | Kiribati | 8·65 (7·68–9·67) | 119·0 (104·9–132·5) | 1·81 (1·4–2·32) | 144·5 (120·6–168·6) | 3·13 (2·5–3·87) | 128·3 (109·1–151·0) | 1·03 (0·764–1·32) | 151·6 (129·5–178·7) |  |
|  | Marshall Islands | 3·64 (3·29–3·98) | 35·1 (24·7–46·7) | 0·806 (0·704–0·92) | 47·2 (23·7–74·6) | 1·34 (1·19–1·49) | 55·9 (34·5–81·0) | 0·307 (0·261–0·358) | 87·0 (51·0–136·5) |  |
|  | Federated States of Micronesia | 11·3 (10·5–12·1) | 98·6 (86·2–112·1) | 2·12 (1·9–2·35) | 127·1 (98·3–161·1) | 3·85 (3·39–4·26) | 148·8 (121·4–182·8) | 0·812 (0·681–0·968) | 111·2 (67·9–161·0) |  |
|  | Nauru | 0·525 (0·451–0·619) | 46·1 (34·1–55·3) | 0·261 (0·202–0·332) | 41·2 (26·0–55·5) | 0·347 (0·278–0·419) | 106·7 (90·7–128·7) | 0·205 (0·158–0·258) | 92·1 (69·6–114·3) |  |
|  | Niue | 0·141 (0·112–0·168) | 25·1 (16·4–32·8) | 0·0345 (0·0243–0·0466) | 52·9 (37·8–68·3) | 0·117 (0·0919–0·148) | 23·9 (14·7–34·4) | 0·0376 (0·0276–0·0484) | 31·8 (19·3–43·2) |  |
|  | Northern Mariana Islands | 2·89 (2·25–3·48) | -2·1 (-15·5–10·1) | 0·443 (0·289–0·617) | 53·0 (21·4–86·8) | 2·16 (1·68–2·78) | 56·2 (33·6–82·2) | 0·798 (0·58–1·07) | 81·9 (46·6–114·8) |  |
|  | Palau | 1·37 (1·25–1·51) | 50·7 (35·5–73·2) | 0·316 (0·264–0·365) | 96·8 (55·8–153·5) | 0·971 (0·869–1·08) | 87·9 (56·1–125·0) | 0·281 (0·243–0·329) | 116·3 (71·3–183·7) |  |
|  | Papua New Guinea | 361 (302–413) | 150·1 (132·3–166·0) | 37·4 (26·6–50·8) | 133·7 (111·6–159·6) | 181 (148–228) | 204·2 (185·0–228·3) | 50·8 (36·7–67·3) | 215·2 (187·8–246·4) |  |
|  | Samoa | 15·6 (13·8–17·5) | 118·5 (103·8–135·4) | 2·2 (1·76–2·8) | 95·1 (69·6–120·9) | 4·76 (3·76–5·91) | 95·1 (74·4–119·6) | 2·01 (1·53–2·57) | 118·1 (90·8–149·3) |  |
|  | Solomon Islands | 35·7 (31·6–39·8) | 220·7 (201·2–239·7) | 11·2 (8·72–13·9) | 235·9 (205·2–263·6) | 7·05 (5·58–8·92) | 230·1 (204·9–258·0) | 2·49 (1·85–3·33) | 235·4 (202·9–273·6) |  |
|  | Tokelau | 0·116 (0·0988–0·133) | 51·7 (39·2–64·3) | 0·0471 (0·0364–0·0589) | 73·4 (54·1–97·2) | 0·103 (0·0863–0·121) | 49·1 (35·9–65·2) | 0·0211 (0·0161–0·0269) | 58·4 (39·4–79·4) |  |
|  | Tonga | 6·1 (5·18–7·08) | 58·1 (47·4–68·5) | 1·35 (0·968–1·8) | 80·1 (64·1–98·2) | 3·86 (3·09–4·89) | 76·3 (61·9–91·5) | 1·03 (0·736–1·34) | 92·4 (73·4–111·2) |  |
|  | Tuvalu | 0·487 (0·391–0·568) | 70·4 (57·5–82·9) | 0·131 (0·0933–0·176) | 78·5 (57·8–103·2) | 0·408 (0·327–0·491) | 84·5 (67·2–101·4) | 0·142 (0·106–0·18) | 116·0 (91·8–145·3) |  |
|  | Vanuatu | 17·3 (15·6–19·2) | 150·1 (138·3–164·1) | 1·27 (0·941–1·7) | 150·6 (132·3–175·3) | 2·55 (1·96–3·32) | 178·1 (157·7–198·1) | 1·92 (1·46–2·43) | 191·3 (168·0–215·5) |  |
|  | Southeast Asia | 19000 (15800–22000) | 90·1 (75·4–101·7) | 2020 (1420–2720) | 62·7 (45·3–78·8) | 10100 (7910–12500) | 194·9 (177·1–211·7) | 8730 (6940–10700) | 218·2 (202·0–235·8) |  |
|  | Cambodia | 215 (163–258) | 116·3 (85·8–140·5) | 38·5 (25–55·5) | 57·9 (24·7–88·1) | 148 (113–186) | 260·8 (218·4–308·7) | 157 (125–196) | 280·3 (243·9–326·2) |  |
|  | Indonesia | 7740 (6330–9180) | 107·9 (93·3–119·7) | 885 (625–1230) | 87·9 (69·9–106·0) | 3550 (2670–4590) | 214·2 (198·2–228·3) | 2730 (2100–3470) | 240·9 (224·2–256·7) |  |
|  | Laos | 142 (119–164) | 103·3 (88·6–116·2) | 24·7 (17·8–33·1) | 35·0 (19·0–49·9) | 81·8 (66–100) | 211·9 (182·8–242·6) | 54·8 (42·8–67·6) | 239·9 (205·8–281·0) |  |
|  | Malaysia | 1400 (1150–1650) | 79·5 (49·8–104·7) | 44·2 (28·7–63·9) | 61·5 (18·5–109·5) | 567 (437–733) | 187·6 (132·9–255·9) | 655 (516–813) | 215·5 (160·1–279·7) |  |
|  | Maldives | 13·8 (11·6–15·9) | 237·9 (211·0–263·2) | 0·96 (0·689–1·29) | 126·5 (95·0–157·5) | 5·22 (4·08–6·41) | 360·0 (312·7–414·2) | 4·35 (3·36–5·44) | 405·0 (352·6–477·7) |  |
|  | Mauritius | 94·7 (71·7–112) | 57·7 (38·9–74·5) | 10·5 (6·34–15·8) | 70·2 (44·6–101·5) | 69·4 (53·4–87·1) | 114·6 (94·7–137·1) | 63·4 (50·6–78·7) | 147·2 (124·1–178·4) |  |
|  | Myanmar | 1490 (1280–1700) | 54·3 (38·9–68·8) | 210 (151–282) | 6·1 (-9·0–22·5) | 963 (784–1150) | 176·9 (140·0–215·6) | 750 (601–902) | 189·6 (150·6–233·8) |  |
|  | Philippines | 2940 (2450–3440) | 106·8 (98·7–114·0) | 306 (211–428) | 105·4 (94·2–116·6) | 1340 (1030–1710) | 155·3 (147·4–163·7) | 1040 (793–1320) | 171·1 (161·9–179·6) |  |
|  | Seychelles | 4·36 (3·36–5·45) | 105·0 (68·4–135·6) | 0·334 (0·199–0·522) | 121·8 (68·7–171·0) | 5·08 (4·16–6·16) | 181·9 (153·8–217·7) | 1·28 (0·968–1·67) | 212·9 (172·5–254·5) |  |
|  | Sri Lanka | 607 (478–724) | 43·2 (20·7–64·4) | 160 (107–217) | 11·0 (-12·4–33·6) | 691 (583–815) | 201·3 (158·5–255·9) | 803 (697–918) | 239·7 (189·1–297·6) |  |
|  | Thailand | 2160 (1620–2650) | 54·5 (22·2–86·2) | 105 (67·1–152) | 84·8 (36·3–139·0) | 1940 (1540–2390) | 192·0 (146·9–238·8) | 1140 (876–1420) | 225·8 (165·5–283·9) |  |
|  | Timor-Leste | 28·3 (25–31·8) | 138·7 (126·4–150·1) | 1·73 (1·21–2·44) | 78·4 (57·7–101·5) | 4·12 (2·96–5·55) | 203·5 (178·8–228·9) | 3·44 (2·43–4·71) | 223·1 (193·9–258·9) |  |
|  | Viet Nam | 2120 (1780–2490) | 110·3 (88·1–136·0) | 195 (133–259) | 66·3 (35·4–100·3) | 755 (582–953) | 233·7 (190·8–291·3) | 1280 (1050–1570) | 221·0 (177·3–261·2) |  |
|  | **Sub-Saharan Africa** | **21000 (18600–23500)** | **133·3 (128·7–137·5)** | **2070 (1570–2690)** | **87·9 (82·1–93·7)** | **4570 (3530–5820)** | **154·7 (148·9–160·2)** | **4280 (3380–5360)** | **165·1 (158·1–172·2)** |  |
|  | Central sub-Saharan Africa | 4460 (4060–4840) | 146·0 (136·5–155·0) | 268 (204–340) | 87·1 (76·1–100·2) | 296 (228–381) | 202·5 (186·5–221·1) | 306 (235–389) | 218·0 (201·2–236·1) |  |
|  | Angola | 1200 (1070–1320) | 223·2 (206·9–241·5) | 98·9 (77·5–122) | 133·1 (112·3–154·2) | 65·6 (47·2–85·2) | 277·6 (245·3–313·5) | 102 (78–128) | 324·8 (296·8–355·8) |  |
|  | Central African Republic | 199 (181–219) | 117·4 (106·8–128·3) | 8·55 (6·57–10·9) | 105·2 (88·9–124·6) | 13·5 (10·4–16·9) | 142·5 (121·3–165·3) | 15·7 (12·5–19·7) | 162·7 (141·0–183·8) |  |
|  | Congo (Brazzaville) | 225 (200–246) | 138·3 (128·6–149·3) | 13·5 (10·2–17·4) | 87·0 (70·6–102·6) | 15·3 (11·9–19·9) | 192·1 (169·6–214·1) | 17·9 (14·1–22·7) | 203·7 (179·4–227·1) |  |
|  | DR Congo | 2690 (2440–2960) | 125·1 (113·8–138·2) | 137 (98·8–183) | 62·9 (47·5–79·5) | 187 (145–243) | 187·4 (165·3–211·7) | 161 (124–207) | 183·1 (161·0–207·3) |  |
|  | Equatorial Guinea | 53·6 (48·2–60·3) | 234·6 (217·5–253·0) | 2·35 (1·74–3·06) | 112·0 (92·8–135·8) | 3·71 (2·82–4·78) | 308·1 (279·0–343·1) | 4·46 (3·44–5·56) | 335·2 (299·8–372·6) |  |
|  | Gabon | 89·7 (81·8–98·6) | 108·0 (97·7–119·4) | 6·39 (4·89–8·19) | 80·0 (64·1–98·6) | 6·83 (5·07–8·75) | 152·3 (133·8–174·6) | 9·12 (7·23–11·5) | 174·7 (152·7–196·1) |  |
|  | Eastern sub-Saharan Africa | 5040 (4510–5630) | 113·8 (108·8–118·0) | 617 (476–804) | 68·3 (62·6–73·7) | 1110 (847–1400) | 154·4 (146·5–162·0) | 1340 (1070–1650) | 161·2 (152·6–168·1) |  |
|  | Burundi | 141 (122–160) | 135·1 (122·1–148·2) | 13·9 (10·3–18·8) | 67·4 (52·6–85·5) | 32 (24·8–41) | 158·5 (139·6–176·9) | 36 (28·3–45·8) | 154·8 (137·4–173·6) |  |
|  | Comoros | 12·2 (9·99–14·2) | 104·5 (90·9–117·5) | 1·25 (0·837–1·76) | 78·0 (58·4–95·1) | 6·92 (5·47–8·51) | 158·7 (139·6–177·6) | 5·59 (4·44–6·95) | 175·5 (155·4–196·6) |  |
|  | Djibouti | 18·1 (15·7–20·6) | 195·0 (175·6–215·9) | 1·82 (1·32–2·4) | 148·7 (122·9–172·8) | 5·17 (3·96–6·66) | 320·0 (291·2–352·6) | 6·12 (4·78–7·58) | 341·5 (316·5–373·6) |  |
|  | Eritrea | 60·8 (48·3–72·9) | 96·0 (74·8–115·2) | 6·14 (3·86–9·51) | 65·7 (32·8–95·6) | 38·3 (29·8–48·3) | 179·0 (145·3–209·1) | 41·9 (34–50·8) | 181·7 (154·3–212·3) |  |
|  | Ethiopia | 1600 (1440–1770) | 89·3 (83·2–94·5) | 139 (109–178) | 9·3 (3·7–15·1) | 148 (113–188) | 144·9 (131·2–159·5) | 178 (140–225) | 157·4 (144·1–172·2) |  |
|  | Kenya | 620 (533–713) | 147·0 (140·7–152·7) | 79·4 (57·9–106) | 183·6 (175·4–192·2) | 182 (138–233) | 130·1 (125·1–136·5) | 276 (217–339) | 159·2 (153·5–166·4) |  |
|  | Madagascar | 312 (273–353) | 126·6 (113·1–140·6) | 28·1 (20·4–37) | 80·2 (62·9–98·5) | 76·7 (58·3–98·1) | 159·8 (138·5–180·6) | 86 (67–106) | 161·9 (141·7–184·2) |  |
|  | Malawi | 147 (128–166) | 111·7 (99·4–125·9) | 27·5 (19·8–36·2) | 63·5 (48·4–81·5) | 38·4 (28·8–49·2) | 137·9 (117·1–157·7) | 61·1 (48–75·9) | 144·1 (123·3–164·3) |  |
|  | Mozambique | 253 (217–296) | 112·3 (99·7–123·1) | 24·9 (17·2–34·5) | 78·2 (60·6–97·4) | 59·5 (39·4–82·3) | 125·0 (101·3–147·4) | 114 (87·2–145) | 123·4 (104·1–141·5) |  |
|  | Rwanda | 153 (138–173) | 100·4 (88·9–111·3) | 12·3 (9·06–16·6) | 27·8 (15·3–41·2) | 22·3 (15·9–30·2) | 198·5 (166·9–230·9) | 44·5 (35·5–54·8) | 230·6 (200·6–259·6) |  |
|  | Somalia | 228 (203–252) | 140·4 (128·9–150·7) | 36·8 (28·3–47·5) | 134·9 (113·8–154·7) | 42·3 (32·5–54·4) | 147·7 (128·1–168·3) | 51·2 (40·2–64·6) | 160·6 (141·8–179·6) |  |
|  | South Sudan | 103 (90–115) | 68·1 (59·0–77·0) | 12·5 (9·34–16·2) | 64·9 (50·9–81·2) | 22·1 (16·8–28) | 81·2 (66·9–97·3) | 25·5 (19·5–31·6) | 79·9 (64·4–94·8) |  |
|  | Uganda | 457 (394–522) | 109·0 (96·1–123·1) | 62·1 (43·3–80·6) | 51·4 (37·0–67·5) | 114 (90–145) | 177·4 (148·7–206·0) | 231 (188–277) | 176·8 (150·6–198·9) |  |
|  | Tanzania | 557 (479–628) | 128·0 (112·7–141·2) | 118 (88·8–155) | 103·7 (87·8–121·3) | 229 (181–289) | 167·2 (149·1–188·1) | 170 (132–215) | 177·8 (155·8–199·7) |  |
|  | Zambia | 391 (349–431) | 189·2 (173·0–209·4) | 31·4 (24·5–39·6) | 96·0 (78·5–116·2) | 67·6 (53–83·3) | 207·3 (174·3–244·5) | 32·5 (25·2–41·5) | 218·2 (177·5–255·1) |  |
|  | Southern sub-Saharan Africa | 2370 (1970–2800) | 111·7 (104·0–118·5) | 292 (205–397) | 140·1 (130·2–152·6) | 1470 (1160–1850) | 119·6 (112·5–128·2) | 851 (641–1080) | 102·8 (93·7–111·0) |  |
|  | Botswana | 44 (37·5–50·4) | 73·8 (61·4–86·5) | 3·92 (2·63–5·49) | 117·2 (90·4–146·9) | 20·1 (15·8–25) | 115·7 (97·1–136·9) | 23·3 (19·2–28·6) | 149·0 (126·6–171·6) |  |
|  | Eswatini | 28·6 (24·3–32·8) | 74·8 (64·8–85·9) | 1·5 (1·06–2·11) | 101·1 (78·9–122·8) | 11·8 (9·52–14·6) | 122·9 (105·1–145·0) | 11·9 (9·65–14·6) | 120·6 (102·1–142·7) |  |
|  | Lesotho | 26 (21·4–30·6) | 53·7 (45·0–62·9) | 2·69 (1·9–3·7) | 84·0 (66·6–102·7) | 17·5 (14–22) | 43·0 (32·6–52·9) | 14·6 (11·9–18·1) | 47·5 (35·6–58·2) |  |
|  | Namibia | 47·6 (38·8–56) | 109·4 (92·3–125·6) | 2·77 (1·86–3·82) | 101·7 (78·0–135·0) | 33·5 (26·8–42) | 145·7 (125·7–166·6) | 20·2 (16·1–25·3) | 170·0 (143·6–194·6) |  |
|  | South Africa | 2010 (1660–2380) | 117·9 (108·9–125·5) | 267 (187–362) | 140·3 (129·8–153·6) | 1280 (1010–1630) | 126·8 (118·2–136·6) | 696 (516–894) | 104·3 (93·4–115·3) |  |
|  | Zimbabwe | 214 (181–252) | 85·0 (74·0–95·2) | 15·6 (11·1–21·4) | 183·4 (154·9–219·7) | 105 (82·4–130) | 67·9 (53·9–80·8) | 80·5 (62·4–101) | 75·8 (61·9–91·1) |  |
|  | Western sub-Saharan Africa | 9200 (8190–10300) | 146·0 (140·4–151·0) | 858 (647–1110) | 88·2 (79·9–96·5) | 1800 (1390–2310) | 188·0 (178·5–197·8) | 1680 (1310–2110) | 204·0 (191·2–217·7) |  |
|  | Benin | 279 (249–308) | 213·8 (196·6–234·9) | 14·2 (11·2–18·2) | 118·1 (96·9–140·4) | 39·7 (30·6–49·5) | 237·3 (203·9–269·4) | 43·4 (34·5–53·6) | 245·4 (213·1–277·7) |  |
|  | Burkina Faso | 234 (209–260) | 111·7 (102·4–121·0) | 20·5 (15·4–26·4) | 68·5 (55·4–81·4) | 43·5 (34·5–55·4) | 133·6 (119·1–151·1) | 39·4 (30·5–49·5) | 136·3 (121·2–152·4) |  |
|  | Cabo Verde | 11·3 (9·42–13·4) | 54·2 (39·0–72·1) | 1·29 (0·911–1·77) | 44·4 (22·0–72·8) | 8·33 (6·92–10) | 144·2 (116·8–177·6) | 5·54 (4·34–6·7) | 147·7 (115·5–184·9) |  |
|  | Cameroon | 561 (477–643) | 229·7 (201·0–258·4) | 48·7 (34·9–66·2) | 144·2 (114·1–178·9) | 155 (119–197) | 256·4 (213·5–294·9) | 191 (151–238) | 270·4 (224·6–312·5) |  |
|  | Chad | 280 (249–312) | 117·8 (108·4–128·5) | 24·6 (18·8–31·6) | 68·4 (54·5–82·7) | 40·3 (31·6–51·7) | 124·2 (108·7–142·0) | 33·5 (26·3–43·1) | 121·8 (106·4–138·5) |  |
|  | Côte d'Ivoire | 727 (645–815) | 155·9 (144·1–167·1) | 65·2 (48·4–84·2) | 105·9 (91·3–123·0) | 111 (86·2–141) | 206·9 (185·9–228·3) | 99·9 (77·6–126) | 220·4 (199·6–241·7) |  |
|  | The Gambia | 68·4 (60·3–76·1) | 147·1 (135·8–161·0) | 6·24 (4·76–7·99) | 116·1 (99·4–133·8) | 10·5 (8·11–13·2) | 170·3 (153·8–186·0) | 9·73 (7·76–12·3) | 184·0 (166·5–203·3) |  |
|  | Ghana | 596 (507–693) | 140·5 (124·6–155·1) | 126 (91–167) | 101·0 (80·2–123·4) | 269 (210–342) | 173·3 (151·1–194·4) | 213 (165–268) | 187·7 (163·1–214·2) |  |
|  | Guinea | 210 (186–234) | 96·0 (83·0–107·8) | 27·4 (20·7–36·1) | 41·1 (28·1–54·9) | 38·9 (28·9–50·8) | 105·5 (83·8–128·7) | 43·9 (34·3–55·6) | 85·9 (65·7–107·0) |  |
|  | Guinea-Bissau | 47·6 (41·9–52·8) | 112·8 (101·5–123·0) | 5·21 (3·97–6·74) | 63·6 (50·4–77·9) | 6·14 (4·81–7·82) | 136·4 (120·6–153·5) | 5·36 (4·23–6·77) | 139·3 (120·5–159·5) |  |
|  | Liberia | 133 (118–150) | 158·9 (142·6–174·3) | 10·1 (6·95–13·9) | 102·4 (79·4–133·0) | 26 (19·7–33·8) | 194·3 (161·0–227·9) | 26·3 (20·3–33·5) | 200·1 (167·3–237·3) |  |
|  | Mali | 786 (705–879) | 115·9 (103·5–127·1) | 60·1 (45·2–79·7) | 89·0 (71·3–108·7) | 109 (82·3–141) | 158·4 (137·1–181·7) | 70 (52·5–92·7) | 165·3 (139·6–189·8) |  |
|  | Mauritania | 126 (112–145) | 115·0 (102·7–127·1) | 12·1 (8·78–15·9) | 70·9 (53·3–87·6) | 16·6 (11·4–23·1) | 165·5 (141·7–194·6) | 18·9 (13·4–25·2) | 196·0 (168·6–225·6) |  |
|  | Niger | 482 (437–528) | 157·0 (145·6–170·4) | 18·1 (12–26·6) | 102·5 (79·5–133·2) | 22·2 (16–30·5) | 225·3 (203·4–252·1) | 17·5 (12·6–23·9) | 235·2 (209·9–263·2) |  |
|  | Nigeria | 3750 (3360–4210) | 151·2 (144·9–156·8) | 325 (244–429) | 79·6 (70·6–88·9) | 747 (574–949) | 203·6 (192·3–214·6) | 715 (545–903) | 223·4 (208·8–238·7) |  |
|  | São Tomé and Príncipe | 6·54 (5·66–7·36) | 163·5 (145·2–183·6) | 0·82 (0·622–1·05) | 104·5 (82·4–127·6) | 1·31 (1–1·65) | 207·6 (171·9–243·0) | 1·34 (1·04–1·71) | 191·3 (153·2–229·1) |  |
|  | Senegal | 598 (529–667) | 136·2 (123·6–149·4) | 49·8 (37·6–63·5) | 86·9 (72·3–102·7) | 105 (82·8–133) | 180·8 (160·5–202·4) | 96·7 (77·7–120) | 191·2 (170·4–213·5) |  |
|  | Sierra Leone | 173 (152–192) | 112·4 (100·5–127·6) | 14·6 (11·1–18·9) | 44·5 (30·0–60·8) | 27·7 (21·6–35·2) | 103·9 (84·1–126·8) | 24·4 (19·1–30·9) | 103·8 (80·1–125·9) |  |
|  | Togo | 144 (127–161) | 153·6 (139·7–167·7) | 13·9 (10·5–18·4) | 128·0 (109·2–148·9) | 25·7 (19·9–32·5) | 230·0 (205·4–252·4) | 23·5 (18·3–29·8) | 251·0 (227·9–278·3) |  |
|  |  |  |  |  |  |  |  |  |  |  |

## Table S14. Undiagnosed proportion, diagnosed and untreated proportion, treated with suboptimal glycaemic levels proportion, and treated with optimal glycaemic levels proportion among people with diabetes in 2023, and corresponding absolute change between 2000 and 2023, for both sexes and ages 15 and older globally, and by Global Burden of Disease super-regions, regions, and countries and territories

|  | **Location** | **Undiagnosed, 2023 (%)** | **Change in Undiagnosed, 2000-2023 (%)** | **Diagnosed & Untreated, 2023 (%)** | **Change in Diagnosed & Untreated, 2000-2023 (%)** | **Treated & Suboptimal Glycaemic Management, 2023 (%)** | **Change in Treated & Suboptimal Glycaemic Management, 2000-2023 (%)** | **Treated & Optimal Glycaemic Management, 2023 (%)** | **Change in Treated & Optimal Glycaemic Management, 2000-2023 (%)** |  |
| --- | --- | --- | --- | --- | --- | --- | --- | --- | --- | --- |
|  | **Global** | **44·2 (37·7–50·7)** | **-8·3 (-10·0–-6·6)** | **4·7 (3·5–6·4)** | **-2·7 (-3·3–-2·2)** | **29·8 (24·4–35·6)** | **5·9 (4·8–7·2)** | **21·2 (17·4–25·6)** | **5·1 (4·2–6·1)** |  |
|  | **Central Europe, eastern Europe, and central Asia** | **32·3 (24·3–40·4)** | **-5·3 (-6·8–-3·7)** | **5·3 (3·3–7·8)** | **-1·3 (-1·8–-0·9)** | **31·0 (24·7–38·4)** | **1·2 (0·1–2·5)** | **31·5 (25·4–37·6)** | **5·4 (4·3–6·5)** |  |
|  | Central Asia | 45·9 (39·4–51·8) | -7·2 (-9·3–-5·1) | 6·2 (4·5–8·8) | -2·9 (-4·1–-2·1) | 27·7 (22·7–33·5) | 5·5 (3·9–7·0) | 20·2 (16·3–24·3) | 4·6 (3·5–5·9) |  |
|  | Armenia | 36·3 (28·5–43·9) | -8·9 (-12·5–-5·3) | 5·5 (3·3–8·9) | -3·4 (-5·3–-2·0) | 34·5 (28·3–41·8) | 6·1 (3·4–8·7) | 23·7 (18·8–28·9) | 6·2 (4·3–8·2) |  |
|  | Azerbaijan | 34·4 (28·0–41·2) | -11·4 (-15·4–-6·8) | 3·9 (2·5–6·0) | -2·5 (-4·1–-1·3) | 43·7 (37·3–50·7) | 8·9 (4·7–12·5) | 17·9 (14·2–21·7) | 5·0 (3·1–7·3) |  |
|  | Georgia | 26·7 (20·8–32·8) | -5·9 (-10·8–-1·6) | 6·6 (4·3–9·2) | -2·0 (-3·9–-0·3) | 36·7 (30·2–43·1) | 3·6 (-0·2–7·3) | 30·0 (24·9–34·6) | 4·3 (1·2–7·3) |  |
|  | Kazakhstan | 40·7 (32·8–47·7) | -12·1 (-16·3–-7·7) | 4·9 (2·9–7·9) | -5·2 (-7·5–-3·6) | 17·2 (13·3–22·1) | 4·8 (3·0–6·7) | 37·1 (31·3–43·1) | 12·5 (9·7–15·3) |  |
|  | Kyrgyzstan | 43·5 (36·0–50·0) | -11·3 (-15·7–-7·5) | 3·8 (2·5–5·5) | -2·6 (-3·7–-1·7) | 41·2 (34·6–48·9) | 10·4 (6·9–14·4) | 11·6 (8·7–14·8) | 3·6 (2·3–5·1) |  |
|  | Mongolia | 55·1 (49·3–60·4) | -6·6 (-9·5–-4·0) | 9·7 (7·5–12·3) | -3·6 (-5·1–-2·3) | 21·4 (17·0–26·1) | 5·9 (4·3–7·9) | 13·8 (11·1–16·8) | 4·2 (3·1–5·5) |  |
|  | Tajikistan | 61·3 (56·5–65·9) | -2·4 (-4·6–-0·3) | 13·7 (10·8–17·0) | -2·6 (-4·3–-1·0) | 19·3 (15·6–24·2) | 3·7 (2·3–5·4) | 5·7 (4·1–8·2) | 1·3 (0·7–2·3) |  |
|  | Turkmenistan | 71·2 (67·0–75·1) | -2·6 (-4·5–-1·2) | 5·2 (4·1–6·7) | -1·5 (-2·4–-0·8) | 17·5 (14·3–21·2) | 2·8 (1·7–4·2) | 6·0 (4·5–7·8) | 1·3 (0·8–1·9) |  |
|  | Uzbekistan | 50·1 (41·5–56·8) | -8·1 (-11·9–-5·6) | 6·4 (4·5–9·0) | -1·9 (-2·7–-1·1) | 28·0 (21·5–34·6) | 5·8 (3·6–8·8) | 15·5 (11·9–19·9) | 4·2 (2·8–6·2) |  |
|  | Central Europe | 28·2 (19·5–37·0) | -2·7 (-4·5–-0·9) | 5·8 (3·4–8·7) | -0·8 (-1·2–-0·3) | 34·6 (27·5–42·6) | 1·4 (0·2–2·6) | 31·5 (24·9–38·4) | 2·2 (0·9–3·6) |  |
|  | Albania | 32·2 (21·2–41·8) | -7·1 (-11·0–-3·5) | 6·3 (3·7–9·5) | -1·9 (-2·8–-0·9) | 35·4 (27·1–44·9) | 3·8 (1·2–6·8) | 26·1 (19·9–33·4) | 5·2 (3·0–7·7) |  |
|  | Bosnia and Herzegovina | 33·4 (22·4–42·8) | -4·5 (-7·7–-1·3) | 7·4 (4·4–11·5) | -1·9 (-3·1–-0·9) | 34·0 (25·5–43·3) | 2·2 (-0·1–4·8) | 25·2 (18·7–31·9) | 4·3 (2·3–6·4) |  |
|  | Bulgaria | 35·9 (25·7–44·8) | -1·4 (-3·6–0·9) | 9·3 (6·0–13·4) | -0·9 (-1·7–0·0) | 31·3 (24·4–40·7) | 0·1 (-1·6–1·7) | 23·5 (17·7–30·3) | 2·2 (0·7–3·7) |  |
|  | Croatia | 26·1 (15·8–36·7) | -4·3 (-7·9–-0·8) | 4·8 (2·5–7·7) | -1·3 (-2·3–-0·4) | 38·7 (30·3–48·1) | 1·1 (-1·8–4·0) | 30·5 (23·1–37·9) | 4·6 (2·5–7·0) |  |
|  | Czechia | 38·0 (28·0–46·5) | -4·5 (-10·7–-0·6) | 10·0 (6·8–14·5) | -2·2 (-4·0–-0·6) | 34·6 (27·4–44·1) | 2·4 (-0·9–7·8) | 17·4 (12·8–22·9) | 4·3 (1·9–6·9) |  |
|  | Hungary | 31·6 (21·6–40·6) | -2·1 (-4·6–0·8) | 7·2 (4·5–10·7) | -0·4 (-1·4–0·4) | 34·3 (26·5–43·7) | -0·3 (-2·4–1·6) | 26·9 (20·8–33·8) | 2·8 (1·0–4·7) |  |
|  | Montenegro | 29·8 (19·9–39·7) | -3·6 (-6·9–-0·4) | 6·1 (3·4–9·7) | -0·8 (-1·6–0·2) | 36·1 (28·1–44·6) | 0·7 (-1·7–3·0) | 28·1 (21·0–35·2) | 3·7 (1·7–5·7) |  |
|  | North Macedonia | 34·2 (23·8–42·9) | -4·9 (-8·1–-1·8) | 8·1 (5·2–11·9) | -2·2 (-3·4–-1·2) | 32·9 (25·0–42·1) | 2·7 (0·5–5·2) | 24·8 (18·2–31·5) | 4·4 (2·6–6·4) |  |
|  | Poland | 21·4 (13·0–31·3) | -3·1 (-6·2–-0·3) | 3·2 (1·6–5·3) | -0·6 (-1·1–-0·1) | 35·3 (28·3–44·4) | 0·8 (-0·9–2·6) | 40·0 (32·2–47·6) | 2·9 (1·0–5·2) |  |
|  | Romania | 34·2 (24·1–43·5) | -4·4 (-6·6–-2·2) | 7·8 (5·0–11·5) | -1·7 (-2·6–-0·7) | 33·2 (26·0–42·3) | 1·9 (0·0–3·6) | 24·8 (18·7–31·4) | 4·1 (2·7–5·7) |  |
|  | Serbia | 34·4 (24·0–43·4) | -4·1 (-7·0–-1·4) | 8·6 (5·4–12·7) | -2·3 (-3·5–-1·4) | 32·3 (24·6–41·8) | 2·1 (0·0–3·8) | 24·6 (18·5–31·5) | 4·3 (2·6–6·3) |  |
|  | Slovakia | 32·2 (22·1–41·8) | -4·0 (-6·8–-1·4) | 6·8 (4·1–10·2) | -1·6 (-2·7–-0·6) | 34·8 (26·9–44·2) | 1·9 (-0·1–4·3) | 26·2 (20·0–32·8) | 3·7 (1·9–5·6) |  |
|  | Slovenia | 21·0 (12·7–30·5) | -6·9 (-11·4–-1·5) | 2·9 (1·5–5·0) | -1·9 (-3·1–-0·7) | 42·5 (34·3–51·6) | 2·6 (-1·4–6·0) | 33·6 (26·8–40·8) | 6·1 (2·9–9·6) |  |
|  | Eastern Europe | 30·5 (22·5–39·1) | -9·0 (-11·0–-6·6) | 4·6 (2·8–7·0) | -1·2 (-1·8–-0·8) | 29·0 (22·7–36·4) | 0·6 (-1·2–2·2) | 35·8 (28·9–42·3) | 9·6 (7·8–11·2) |  |
|  | Belarus | 22·7 (18·5–27·3) | -9·3 (-14·2–-3·2) | 9·0 (6·4–12·0) | -8·1 (-12·3–-3·9) | 27·1 (22·6–32·0) | 6·5 (2·0–10·0) | 41·2 (35·7–46·6) | 10·9 (4·8–16·7) |  |
|  | Estonia | 27·1 (17·7–36·0) | -10·0 (-13·5–-5·1) | 4·2 (2·3–6·8) | -3·4 (-5·0–-2·2) | 34·1 (27·4–41·5) | 5·2 (2·1–7·7) | 34·7 (27·9–41·8) | 8·2 (5·5–10·8) |  |
|  | Latvia | 33·0 (23·4–41·3) | -5·0 (-8·3–-1·7) | 6·9 (4·1–10·0) | -1·6 (-2·6–-0·6) | 29·9 (23·1–37·9) | 2·0 (0·2–3·9) | 30·2 (23·4–37·1) | 4·6 (2·5–6·7) |  |
|  | Lithuania | 31·2 (21·6–40·3) | -7·9 (-15·3–-2·7) | 4·6 (2·6–7·0) | -1·4 (-2·7–-0·3) | 38·4 (30·3–47·2) | 4·1 (-0·6–9·7) | 25·9 (19·8–31·9) | 5·1 (1·8–8·7) |  |
|  | Moldova | 42·1 (35·8–47·9) | -3·7 (-7·1–0·3) | 12·1 (9·4–15·5) | -2·0 (-3·7–-0·4) | 24·9 (20·1–30·7) | 2·4 (-0·9–4·6) | 20·9 (17·0–25·4) | 3·3 (1·0–5·6) |  |
|  | Russia | 28·5 (19·2–37·9) | -10·9 (-13·3–-8·0) | 2·7 (1·5–4·3) | -1·1 (-1·5–-0·7) | 29·8 (23·2–37·5) | 0·2 (-1·8–2·1) | 39·0 (31·5–46·0) | 11·7 (9·4–13·6) |  |
|  | Ukraine | 41·6 (34·1–49·2) | 0·0 (-2·0–2·1) | 10·3 (7·2–13·9) | 2·7 (1·8–3·8) | 25·1 (19·1–32·2) | -2·3 (-3·8–-0·8) | 23·1 (17·7–28·4) | -0·4 (-1·6–1·0) |  |
|  | **High income** | **20·5 (14·2–27·4)** | **-6·3 (-7·6–-4·9)** | **6·7 (4·1–9·6)** | **-1·5 (-2·1–-1·0)** | **40·1 (33·6–47·8)** | **2·3 (1·1–3·5)** | **32·7 (26·8–38·3)** | **5·5 (4·5–6·6)** |  |
|  | Australasia | 29·4 (21·4–36·9) | -6·3 (-11·8–-2·2) | 9·7 (6·3–13·8) | -2·8 (-4·8–-1·0) | 40·6 (32·8–49·9) | 4·1 (-0·2–9·0) | 20·3 (15·5–25·4) | 5·0 (2·4–7·9) |  |
|  | Australia | 29·3 (21·1–37·0) | -6·7 (-13·2–-2·1) | 9·5 (6·2–13·6) | -2·7 (-5·1–-0·8) | 40·7 (32·9–50·0) | 4·1 (-0·8–9·9) | 20·6 (15·8–25·8) | 5·3 (2·2–8·7) |  |
|  | New Zealand | 30·0 (22·6–37·1) | -4·9 (-7·3–-2·5) | 10·4 (6·6–14·8) | -3·4 (-4·7–-2·2) | 40·3 (32·5–49·2) | 3·9 (1·6–6·3) | 19·2 (14·7–24·4) | 4·4 (2·8–5·9) |  |
|  | High-income Asia Pacific | 24·6 (18·1–31·5) | -11·1 (-14·2–-7·7) | 2·6 (1·7–3·8) | -2·3 (-3·3–-1·5) | 35·8 (30·4–41·6) | 3·9 (1·8–5·9) | 37·0 (31·6–42·3) | 9·5 (7·2–11·3) |  |
|  | Brunei | 26·2 (20·3–33·1) | -8·4 (-14·7–-3·9) | 6·8 (4·5–9·5) | -2·2 (-4·4–-0·4) | 41·4 (34·7–48·1) | 5·5 (0·9–10·5) | 25·6 (20·0–30·7) | 5·2 (2·1–9·0) |  |
|  | Japan | 25·3 (16·6–34·1) | -8·5 (-12·3–-4·5) | 2·6 (1·5–4·0) | -1·3 (-2·0–-0·7) | 35·1 (28·3–42·7) | 1·5 (-0·9–4·0) | 37·0 (30·0–44·3) | 8·2 (5·2–10·7) |  |
|  | South Korea | 22·9 (20·9–25·1) | -20·7 (-23·1–-18·0) | 2·5 (2·1–3·2) | -7·2 (-8·7–-5·9) | 36·7 (34·5–38·9) | 12·6 (10·2–14·5) | 37·9 (35·8–40·2) | 15·3 (13·5–17·1) |  |
|  | Singapore | 25·5 (17·9–32·8) | -17·9 (-24·1–-11·9) | 2·9 (1·8–4·4) | -3·4 (-4·5–-2·4) | 45·6 (38·8–52·8) | 12·4 (7·4–17·7) | 26·1 (20·9–31·1) | 8·9 (5·3–12·5) |  |
|  | High-income North America | 17·1 (10·7–24·5) | -4·4 (-6·0–-2·5) | 6·2 (3·4–9·8) | 0·5 (-0·4–1·5) | 40·7 (33·4–48·8) | -0·1 (-1·9–1·5) | 36·0 (29·4–42·1) | 4·0 (2·6–5·5) |  |
|  | Canada | 14·0 (8·2–20·6) | -2·6 (-6·6–1·3) | 3·0 (1·6–5·3) | -0·3 (-1·2–0·6) | 45·8 (38·4–52·8) | 1·3 (-1·4–4·5) | 37·2 (30·6–43·6) | 1·6 (-1·2–4·0) |  |
|  | Greenland | 27·5 (19·3–35·1) | -6·3 (-11·1–-2·3) | 12·4 (7·8–17·2) | -5·5 (-8·1–-3·4) | 34·8 (26·2–43·9) | 6·4 (3·1–10·8) | 25·4 (19·6–31·4) | 5·5 (2·8–8·7) |  |
|  | USA | 17·2 (10·8–24·6) | -4·5 (-6·2–-2·6) | 6·4 (3·6–10·2) | 0·5 (-0·3–1·6) | 40·4 (33·1–48·5) | -0·2 (-2·0–1·5) | 35·9 (29·4–42·1) | 4·2 (2·7–5·8) |  |
|  | Southern Latin America | 20·1 (15·0–25·1) | -5·5 (-9·6–-2·0) | 6·6 (4·3–9·2) | -2·5 (-4·7–-1·0) | 27·4 (22·7–32·5) | 2·2 (-0·2–5·1) | 45·9 (40·4–51·3) | 5·8 (2·3–9·5) |  |
|  | Argentina | 20·1 (14·9–25·0) | -4·2 (-9·5–0·7) | 6·4 (4·3–8·9) | -1·8 (-4·5–-0·1) | 26·7 (21·8–31·6) | 1·5 (-1·6–5·1) | 46·9 (41·1–52·3) | 4·5 (0·0–9·2) |  |
|  | Chile | 17·1 (12·0–22·4) | -10·6 (-14·2–-7·0) | 7·0 (4·4–9·9) | -6·5 (-8·9–-4·6) | 31·7 (26·3–37·5) | 3·9 (0·7–7·2) | 44·3 (38·5–49·3) | 13·3 (9·5–16·5) |  |
|  | Uruguay | 31·5 (25·3–38·0) | -3·0 (-5·9–0·1) | 7·5 (5·2–10·3) | -1·6 (-3·0–-0·4) | 20·9 (16·5–25·2) | 0·3 (-1·5–2·1) | 40·1 (34·3–46·7) | 4·3 (1·2–6·7) |  |
|  | Western Europe | 22·5 (16·1–29·4) | -3·5 (-5·4–-1·8) | 9·2 (5·7–12·8) | -2·5 (-3·2–-1·8) | 43·1 (35·8–51·0) | 3·3 (1·8–5·0) | 25·3 (20·2–30·9) | 2·7 (1·7–3·8) |  |
|  | Andorra | 20·2 (13·6–27·5) | -3·2 (-6·7–0·2) | 5·2 (2·9–8·2) | -0·8 (-1·7–0·3) | 47·9 (40·6–55·5) | 1·8 (-1·2–4·7) | 26·7 (21·0–32·1) | 2·2 (0·6–4·4) |  |
|  | Austria | 21·1 (13·7–29·4) | -4·0 (-7·4–-0·7) | 6·2 (3·4–9·5) | -2·0 (-3·4–-0·7) | 46·0 (38·3–53·8) | 3·0 (0·5–5·7) | 26·7 (21·0–32·8) | 3·1 (1·3–5·2) |  |
|  | Belgium | 22·5 (14·8–30·2) | -3·6 (-6·3–-0·6) | 6·9 (4·0–10·4) | -2·0 (-3·3–-0·7) | 44·8 (37·0–53·6) | 2·8 (0·2–5·3) | 25·8 (20·4–31·9) | 2·8 (1·0–4·5) |  |
|  | Cyprus | 22·9 (15·3–31·1) | -9·2 (-12·5–-5·7) | 7·3 (4·2–10·7) | -7·4 (-9·6–-5·4) | 44·9 (37·1–52·9) | 10·1 (6·9–13·5) | 25·0 (19·8–31·0) | 6·4 (4·4–8·3) |  |
|  | Denmark | 22·3 (14·7–30·2) | -6·9 (-10·4–-2·9) | 6·8 (4·0–10·4) | -4·7 (-6·7–-3·1) | 44·8 (36·8–53·7) | 6·1 (2·6–9·8) | 26·1 (20·7–32·1) | 5·4 (3·1–8·1) |  |
|  | Finland | 13·2 (7·9–19·6) | -3·9 (-8·0–0·4) | 4·0 (2·0–6·8) | -2·0 (-3·8–0·0) | 51·9 (44·6–59·2) | 2·0 (-2·2–6·0) | 30·9 (24·8–36·8) | 3·9 (1·0–6·8) |  |
|  | France | 27·7 (20·0–35·2) | -8·3 (-15·3–-2·3) | 7·7 (4·9–11·1) | -4·7 (-7·4–-2·4) | 41·0 (33·7–49·1) | 7·6 (2·6–13·4) | 23·6 (18·3–29·3) | 5·4 (2·2–9·3) |  |
|  | Germany | 22·7 (15·3–30·4) | -2·4 (-4·8–0·6) | 7·9 (4·5–11·4) | -1·6 (-2·8–-0·2) | 43·6 (35·2–52·3) | 1·8 (-1·0–4·0) | 25·9 (20·3–31·7) | 2·2 (0·4–3·8) |  |
|  | Greece | 24·3 (17·1–31·4) | -0·6 (-3·4–1·7) | 9·8 (6·1–13·8) | 1·3 (0·2–2·3) | 41·1 (33·8–49·7) | -1·4 (-3·5–0·7) | 24·8 (19·4–30·5) | 0·8 (-0·8–2·6) |  |
|  | Iceland | 21·3 (14·5–29·7) | -2·7 (-5·7–0·4) | 5·8 (3·3–8·7) | -0·9 (-2·2–0·3) | 46·0 (38·8–54·6) | 1·5 (-1·3–4·5) | 26·8 (21·2–32·5) | 2·1 (0·4–4·0) |  |
|  | Ireland | 20·9 (13·9–28·8) | -8·8 (-12·6–-4·4) | 5·8 (3·4–8·8) | -5·2 (-7·1–-3·4) | 46·4 (39·0–54·9) | 8·0 (4·3–11·7) | 26·9 (21·3–32·9) | 6·0 (3·8–8·3) |  |
|  | Israel | 23·6 (16·0–31·3) | -5·3 (-8·7–-2·1) | 7·7 (4·4–11·2) | -4·4 (-6·1–-2·7) | 43·4 (35·5–51·8) | 5·9 (3·0–8·7) | 25·3 (20·3–31·0) | 3·9 (1·9–6·0) |  |
|  | Italy | 20·5 (13·3–28·7) | -2·3 (-3·7–-0·6) | 5·9 (3·4–8·9) | -0·7 (-1·2–-0·3) | 46·6 (38·9–55·1) | 0·9 (-0·7–2·2) | 27·1 (21·5–33·1) | 2·2 (1·3–3·1) |  |
|  | Luxembourg | 24·4 (16·8–32·2) | -5·9 (-11·6–-1·0) | 6·9 (4·1–10·2) | -3·4 (-5·9–-1·7) | 44·0 (36·4–51·8) | 5·5 (1·4–10·5) | 24·7 (19·5–29·8) | 3·9 (1·5–6·8) |  |
|  | Malta | 22·7 (15·1–30·9) | -4·6 (-8·2–-0·9) | 7·4 (4·4–11·0) | -2·7 (-4·6–-1·2) | 44·0 (36·0–52·4) | 3·4 (0·0–6·7) | 25·9 (20·6–32·1) | 4·0 (1·5–6·3) |  |
|  | Monaco | 22·0 (14·6–30·0) | -2·7 (-5·4–0·1) | 8·1 (4·7–11·6) | -1·5 (-3·0–-0·3) | 43·3 (35·7–52·7) | 1·9 (-0·6–4·6) | 26·7 (20·9–32·7) | 2·3 (0·5–4·4) |  |
|  | Netherlands | 20·3 (13·3–28·9) | -5·1 (-8·9–-1·4) | 5·7 (3·2–9·2) | -2·9 (-4·6–-1·5) | 46·6 (38·6–55·7) | 4·0 (0·7–7·3) | 27·4 (21·6–33·8) | 4·0 (2·0–6·4) |  |
|  | Norway | 21·4 (13·8–30·0) | -1·4 (-3·1–0·4) | 2·2 (1·2–3·7) | -0·7 (-1·1–-0·4) | 47·5 (40·3–55·3) | 0·6 (-0·7–2·2) | 28·9 (23·5–34·5) | 1·5 (0·1–2·6) |  |
|  | Portugal | 11·6 (6·8–17·4) | -4·3 (-9·4–1·0) | 4·1 (2·0–6·8) | -3·0 (-5·1–-0·7) | 52·9 (45·6–59·8) | 3·1 (-2·0–8·1) | 31·5 (25·3–37·5) | 4·2 (1·0–7·1) |  |
|  | San Marino | 18·9 (11·7–27·0) | -2·2 (-4·9–0·9) | 5·5 (3·0–8·7) | -0·1 (-1·0–0·9) | 47·2 (39·5–55·5) | 0·6 (-2·1–2·9) | 28·3 (22·6–34·8) | 1·7 (-0·4–3·2) |  |
|  | Spain | 17·3 (12·9–22·6) | -2·4 (-5·6–1·0) | 23·5 (16·5–29·9) | -7·4 (-11·8–-3·1) | 37·0 (30·1–45·0) | 5·7 (1·1–10·8) | 22·3 (17·5–27·8) | 4·1 (1·9–7·0) |  |
|  | Sweden | 19·8 (13·1–27·5) | -3·5 (-6·9–-0·1) | 4·9 (2·7–7·8) | -1·8 (-3·0–-0·6) | 47·6 (40·0–55·8) | 2·7 (-0·2–5·6) | 27·6 (21·8–34·3) | 2·6 (0·9–4·7) |  |
|  | Switzerland | 30·3 (22·5–37·6) | -5·7 (-11·3–-0·7) | 8·0 (5·4–11·0) | -3·3 (-4·9–-1·1) | 36·0 (29·1–43·6) | 4·7 (-0·4–9·3) | 25·6 (20·6–31·7) | 4·2 (1·2–7·5) |  |
|  | UK | 25·8 (18·9–33·3) | -3·2 (-4·3–-1·9) | 8·4 (5·4–11·8) | -0·4 (-1·0–0·2) | 42·0 (34·9–49·7) | 3·3 (2·1–4·5) | 23·8 (18·8–29·2) | 0·3 (-0·5–1·0) |  |
|  | **Latin America and Caribbean** | **36·4 (28·5–44·2)** | **-11·6 (-13·4–-9·5)** | **5·4 (3·7–7·6)** | **-0·5 (-1·0–0·0)** | **33·8 (27·4–40·7)** | **5·3 (3·7–7·1)** | **24·3 (19·6–29·4)** | **6·8 (5·4–8·2)** |  |
|  | Andean Latin America | 42·7 (36·6–48·9) | -7·2 (-9·0–-5·6) | 10·1 (7·6–12·8) | -3·2 (-4·1–-2·3) | 27·0 (21·8–32·6) | 4·5 (3·0–5·9) | 20·3 (16·5–24·3) | 5·9 (4·7–7·1) |  |
|  | Bolivia | 49·7 (43·1–55·9) | -4·6 (-7·1–-2·6) | 12·8 (10·0–16·4) | -4·0 (-5·4–-2·8) | 22·1 (17·3–27·8) | 4·1 (2·9–5·8) | 15·3 (11·8–19·0) | 4·4 (3·4–5·7) |  |
|  | Ecuador | 38·2 (30·9–44·9) | -8·8 (-13·5–-3·5) | 4·7 (3·2–6·3) | -1·0 (-2·0–-0·2) | 31·7 (26·1–37·8) | 3·5 (-0·3–6·7) | 25·4 (21·0–30·1) | 6·3 (3·7–9·1) |  |
|  | Peru | 43·0 (36·4–49·3) | -6·8 (-10·1–-4·1) | 13·7 (10·6–17·5) | -5·1 (-6·6–-3·8) | 25·1 (19·8–31·0) | 5·7 (3·5–8·3) | 18·2 (14·6–22·1) | 6·3 (4·3–8·1) |  |
|  | Caribbean | 36·6 (29·9–43·0) | -4·3 (-5·4–-3·0) | 5·7 (4·1–7·9) | -0·2 (-0·5–0·2) | 34·0 (28·2–40·1) | 1·0 (0·0–2·1) | 23·7 (19·5–28·0) | 3·5 (2·6–4·4) |  |
|  | Antigua and Barbuda | 32·8 (25·2–40·6) | -5·6 (-8·1–-2·7) | 4·5 (2·8–6·5) | -0·1 (-0·7–0·5) | 37·1 (30·4–44·0) | 1·5 (-0·9–3·8) | 25·6 (20·4–31·1) | 4·2 (2·4–5·9) |  |
|  | The Bahamas | 38·7 (30·9–46·5) | -5·8 (-8·3–-3·3) | 6·6 (4·5–9·1) | 0·2 (-0·5–1·0) | 32·7 (25·9–39·3) | 1·8 (-0·3–3·8) | 22·0 (17·6–27·0) | 3·9 (2·5–5·8) |  |
|  | Barbados | 30·1 (22·1–37·5) | -4·6 (-7·6–-1·8) | 5·5 (3·5–8·0) | -0·3 (-1·0–0·6) | 36·8 (30·1–43·9) | 0·7 (-1·8–3·3) | 27·7 (22·6–33·3) | 4·2 (2·2–6·3) |  |
|  | Belize | 40·4 (33·2–47·0) | -7·4 (-9·8–-4·7) | 7·8 (5·6–10·6) | -1·6 (-2·7–-0·9) | 30·4 (24·3–37·1) | 4·5 (2·3–6·9) | 21·4 (16·8–26·0) | 4·6 (3·0–6·3) |  |
|  | Bermuda | 19·7 (13·7–27·0) | -7·1 (-10·4–-4·0) | 1·8 (1·1–2·9) | -0·4 (-0·9–0·0) | 42·8 (36·4–49·2) | 0·1 (-3·0–2·8) | 35·7 (30·9–41·8) | 7·4 (5·0–9·6) |  |
|  | Cuba | 26·7 (20·0–33·9) | -8·2 (-11·2–-5·2) | 2·8 (1·7–4·1) | -0·3 (-0·7–0·2) | 41·1 (34·5–48·1) | 2·4 (0·2–4·9) | 29·5 (24·0–34·4) | 6·1 (4·1–8·0) |  |
|  | Dominica | 38·9 (31·4–46·2) | -2·1 (-4·4–-0·2) | 9·3 (6·5–12·4) | 1·1 (0·3–2·1) | 29·8 (23·0–36·7) | -0·9 (-2·4–0·9) | 22·0 (17·2–26·7) | 2·0 (0·7–3·5) |  |
|  | Dominican Republic | 42·5 (34·5–49·3) | -5·2 (-6·9–-3·2) | 7·1 (5·0–9·6) | 0·2 (-0·5–0·9) | 30·7 (24·6–37·3) | 1·8 (0·2–3·6) | 19·7 (15·6–24·1) | 3·1 (1·9–4·5) |  |
|  | Grenada | 36·1 (28·2–43·7) | -4·0 (-6·5–-1·7) | 6·7 (4·4–9·4) | 0·5 (-0·2–1·4) | 33·5 (27·2–41·2) | 0·3 (-1·4–2·3) | 23·7 (18·5–28·9) | 3·1 (1·7–5·0) |  |
|  | Guyana | 30·8 (24·5–37·2) | -7·9 (-11·5–-4·6) | 6·0 (4·2–8·1) | -1·1 (-2·5–0·0) | 38·9 (32·6–44·6) | 3·7 (0·6–6·9) | 24·3 (19·6–29·4) | 5·3 (3·5–7·4) |  |
|  | Haiti | 53·5 (46·9–59·0) | -2·3 (-4·0–-0·7) | 10·0 (7·5–13·0) | -1·2 (-2·0–-0·4) | 23·9 (18·8–29·7) | 1·7 (0·5–3·1) | 12·6 (9·8–16·3) | 1·7 (0·8–2·6) |  |
|  | Jamaica | 36·5 (29·2–43·7) | -3·2 (-5·6–-0·5) | 5·5 (3·7–7·7) | 0·1 (-0·5–0·8) | 34·2 (27·4–41·6) | 0·4 (-1·6–2·2) | 23·7 (19·1–28·5) | 2·6 (1·2–4·1) |  |
|  | Puerto Rico | 21·9 (14·6–29·9) | -8·7 (-12·0–-4·9) | 2·8 (1·6–4·3) | -1·2 (-2·0–-0·6) | 41·3 (34·5–47·9) | 2·3 (-0·7–4·8) | 34·1 (28·9–39·4) | 7·7 (5·4–9·9) |  |
|  | Saint Kitts and Nevis | 37·1 (28·8–45·0) | -6·8 (-10·1–-3·1) | 7·5 (5·0–10·6) | 0·0 (-1·1–1·2) | 33·0 (26·3–40·7) | 2·9 (0·0–5·7) | 22·5 (17·5–27·6) | 3·8 (1·5–6·6) |  |
|  | Saint Lucia | 47·7 (41·7–52·7) | -3·4 (-6·7–0·6) | 7·6 (5·8–9·9) | 0·3 (-0·7–1·2) | 26·1 (21·7–31·3) | 0·9 (-2·4–3·4) | 18·6 (15·3–21·9) | 2·2 (0·1–4·1) |  |
|  | Saint Vincent and the Grenadines | 36·4 (28·4–44·1) | -5·4 (-7·7–-3·3) | 6·3 (4·3–8·8) | 0·1 (-0·7–0·8) | 34·0 (27·4–41·9) | 1·5 (-0·5–3·7) | 23·3 (18·3–28·0) | 3·9 (2·4–5·2) |  |
|  | Suriname | 41·9 (33·9–49·1) | -7·1 (-9·5–-4·8) | 7·6 (5·4–10·7) | -0·6 (-1·5–0·1) | 31·2 (24·7–38·2) | 3·6 (1·7–5·8) | 19·3 (15·2–23·9) | 4·1 (2·7–5·5) |  |
|  | Trinidad and Tobago | 34·0 (26·3–42·1) | -7·0 (-9·8–-4·4) | 6·4 (4·3–9·0) | -1·4 (-2·3–-0·5) | 34·8 (27·6–42·2) | 3·1 (1·1–5·7) | 24·8 (19·7–30·4) | 5·2 (3·5–7·1) |  |
|  | Virgin Islands | 27·3 (19·9–34·6) | -10·6 (-14·0–-6·6) | 4·5 (2·7–6·8) | -2·4 (-3·6–-1·4) | 38·0 (31·3–44·7) | 4·4 (1·4–7·3) | 30·2 (24·5–35·8) | 8·6 (6·1–11·1) |  |
|  | Central Latin America | 36·6 (28·5–44·3) | -13·1 (-15·3–-10·9) | 4·7 (3·2–6·7) | -0·2 (-0·8–0·4) | 34·4 (27·9–41·2) | 5·1 (3·2–7·2) | 24·2 (19·6–29·1) | 8·2 (6·6–9·9) |  |
|  | Colombia | 32·7 (24·0–40·9) | -12·7 (-17·2–-8·5) | 2·6 (1·7–3·9) | -1·4 (-2·0–-0·8) | 31·0 (24·7–37·7) | 4·8 (1·6–8·1) | 33·7 (28·0–39·4) | 9·3 (6·3–12·4) |  |
|  | Costa Rica | 33·2 (25·0–41·4) | -9·8 (-15·5–-3·3) | 3·5 (2·2–5·3) | -0·8 (-1·7–0·2) | 36·0 (29·2–43·8) | 4·5 (0·1–9·4) | 27·3 (21·8–32·8) | 6·1 (2·1–9·6) |  |
|  | El Salvador | 42·9 (34·5–50·4) | -6·9 (-9·4–-4·8) | 5·3 (3·5–7·2) | -0·9 (-1·4–-0·4) | 29·2 (23·0–36·2) | 3·6 (1·9–5·6) | 22·6 (17·9–27·7) | 4·2 (2·8–5·7) |  |
|  | Guatemala | 52·7 (49·2–56·4) | -5·9 (-9·4–-2·2) | 7·8 (6·0–10·0) | -1·1 (-2·1–-0·2) | 22·7 (18·7–26·3) | 3·4 (0·7–6·0) | 16·8 (13·8–19·8) | 3·6 (1·7–5·6) |  |
|  | Honduras | 53·4 (46·1–59·6) | -4·8 (-6·6–-3·3) | 7·2 (5·3–9·4) | -0·9 (-1·5–-0·4) | 23·5 (18·5–29·2) | 3·0 (2·0–4·3) | 15·9 (12·5–19·6) | 2·7 (1·8–3·8) |  |
|  | Mexico | 34·6 (25·6–42·9) | -15·5 (-18·2–-12·9) | 4·8 (3·1–7·0) | -0·1 (-0·8–0·6) | 37·6 (30·5–44·9) | 6·6 (4·4–9·3) | 23·0 (18·0–27·9) | 9·0 (7·0–10·9) |  |
|  | Nicaragua | 43·2 (35·3–50·2) | -8·7 (-11·5–-6·3) | 4·6 (3·1–6·3) | -1·1 (-1·6–-0·6) | 30·0 (23·7–36·9) | 5·0 (3·1–7·0) | 22·2 (17·9–27·6) | 4·8 (3·2–6·4) |  |
|  | Panama | 33·2 (25·0–41·5) | -9·3 (-12·5–-6·3) | 2·6 (1·6–3·9) | -0·6 (-1·0–-0·2) | 36·7 (29·6–44·4) | 4·4 (1·9–6·9) | 27·4 (22·1–32·9) | 5·5 (3·8–7·5) |  |
|  | Venezuela | 43·3 (34·9–51·1) | -5·9 (-8·5–-3·4) | 5·8 (4·1–8·0) | 0·9 (0·0–2·0) | 28·7 (22·1–35·7) | 1·9 (0·0–3·8) | 22·2 (17·1–27·3) | 3·1 (1·6–4·8) |  |
|  | Tropical Latin America | 34·7 (26·2–42·9) | -12·2 (-14·5–-9·5) | 5·6 (3·5–8·2) | -1·1 (-1·7–-0·4) | 34·2 (27·1–41·6) | 7·1 (5·0–9·1) | 25·5 (20·2–31·5) | 6·1 (4·6–7·7) |  |
|  | Brazil | 34·5 (26·0–42·8) | -12·5 (-14·9–-9·7) | 5·5 (3·5–8·1) | -1·1 (-1·8–-0·5) | 34·3 (27·2–41·8) | 7·2 (5·1–9·3) | 25·7 (20·3–31·6) | 6·3 (4·7–7·9) |  |
|  | Paraguay | 40·2 (31·1–47·6) | -3·3 (-5·5–-1·1) | 7·0 (4·6–9·9) | 0·0 (-0·6–0·7) | 30·8 (23·9–38·3) | 2·1 (0·2–3·8) | 22·0 (17·2–27·4) | 1·3 (0·2–2·7) |  |
|  | **North Africa and Middle East** | **34·6 (28·8–40·2)** | **-9·0 (-11·0–-7·3)** | **4·9 (3·7–6·7)** | **-1·6 (-2·2–-1·0)** | **33·3 (28·2–38·8)** | **4·3 (2·9–5·7)** | **27·3 (22·7–32·6)** | **6·3 (5·2–7·6)** |  |
|  | Afghanistan | 58·5 (52·1–64·0) | -3·9 (-9·1–0·6) | 3·0 (2·2–4·0) | -1·2 (-2·1–-0·4) | 20·7 (16·8–25·5) | 1·6 (-1·3–5·4) | 17·8 (14·1–22·2) | 3·4 (0·8–6·0) |  |
|  | Algeria | 35·3 (30·2–40·2) | -14·6 (-17·9–-11·5) | 4·5 (3·3–5·9) | -0·7 (-1·5–0·2) | 35·2 (30·0–40·1) | 10·1 (7·2–13·0) | 25·1 (21·1–29·4) | 5·3 (2·7–7·8) |  |
|  | Bahrain | 31·1 (22·1–40·7) | -14·0 (-18·3–-9·7) | 3·8 (2·2–5·8) | -2·4 (-3·4–-1·5) | 36·4 (28·9–44·9) | 7·9 (4·3–11·3) | 28·7 (22·0–35·7) | 8·5 (6·0–11·4) |  |
|  | Egypt | 38·2 (33·8–42·1) | 2·9 (-1·3–5·5) | 6·7 (5·2–8·7) | 0·2 (-0·9–1·1) | 31·2 (26·3–36·4) | -3·4 (-5·5–-0·3) | 24·0 (20·0–29·0) | 0·3 (-1·3–2·6) |  |
|  | Iran | 27·6 (20·5–34·7) | -9·7 (-11·5–-8·1) | 9·5 (6·2–13·5) | -1·8 (-2·6–-1·0) | 35·6 (28·6–42·6) | 8·4 (6·6–10·5) | 27·3 (21·8–33·7) | 3·1 (1·8–4·5) |  |
|  | Iraq | 39·7 (33·1–45·1) | -14·2 (-19·0–-9·8) | 3·5 (2·5–4·6) | -3·6 (-5·0–-2·3) | 36·0 (31·5–41·5) | 10·5 (6·5–14·6) | 20·7 (16·6–24·6) | 7·4 (5·2–9·7) |  |
|  | Jordan | 21·2 (16·5–26·8) | -16·8 (-21·1–-11·6) | 3·9 (2·6–5·6) | -5·6 (-7·5–-3·3) | 42·3 (37·2–47·4) | 8·8 (3·9–13·1) | 32·6 (27·8–37·1) | 13·5 (10·0–16·5) |  |
|  | Kuwait | 25·3 (17·6–32·8) | -6·8 (-11·9–-0·7) | 3·8 (2·2–5·7) | -0·7 (-2·0–0·4) | 47·1 (39·5–54·4) | 0·2 (-6·0–5·5) | 23·9 (18·9–30·0) | 7·3 (3·9–11·5) |  |
|  | Lebanon | 48·7 (42·6–54·0) | -7·9 (-11·3–-3·6) | 2·9 (1·9–4·2) | -1·9 (-3·1–-1·0) | 23·8 (19·4–28·4) | 4·0 (1·3–6·0) | 24·6 (20·6–28·7) | 5·8 (3·0–8·0) |  |
|  | Libya | 43·4 (35·2–50·7) | -4·5 (-9·7–-0·4) | 8·4 (5·8–11·2) | 2·3 (0·7–3·9) | 29·0 (22·8–35·8) | 0·1 (-3·4–4·1) | 19·1 (14·8–24·4) | 2·1 (-0·3–5·4) |  |
|  | Morocco | 44·5 (39·4–49·4) | -9·3 (-13·5–-5·3) | 6·0 (4·6–7·6) | -2·1 (-3·6–-0·9) | 31·6 (26·9–36·3) | 6·4 (3·3–9·5) | 18·0 (14·7–20·9) | 5·0 (3·3–7·0) |  |
|  | Oman | 36·7 (27·9–44·9) | -6·6 (-10·1–-2·8) | 4·1 (2·6–6·1) | -0·4 (-1·1–0·4) | 33·0 (26·5–40·6) | 1·9 (-0·7–4·4) | 26·2 (20·2–32·7) | 5·1 (3·2–7·4) |  |
|  | Palestine | 29·4 (21·5–37·8) | -10·0 (-17·1–-3·5) | 3·2 (1·8–4·9) | -2·4 (-3·6–-1·2) | 46·0 (39·0–54·4) | 7·1 (1·6–13·6) | 21·4 (17·0–26·5) | 5·3 (2·3–8·6) |  |
|  | Qatar | 24·5 (16·5–32·6) | -10·8 (-16·5–-5·1) | 3·6 (1·9–6·0) | -4·1 (-6·1–-2·3) | 33·9 (27·7–41·4) | 4·6 (0·7–8·3) | 38·0 (30·8–46·5) | 10·4 (6·8–14·4) |  |
|  | Saudi Arabia | 31·7 (22·0–40·6) | -10·0 (-16·5–-4·6) | 1·1 (0·6–1·6) | -0·5 (-1·1–-0·1) | 26·4 (22·0–31·4) | -5·1 (-10·1–-1·2) | 40·8 (33·9–47·4) | 15·6 (10·7–21·6) |  |
|  | Sudan | 41·4 (36·2–46·3) | -8·3 (-12·0–-4·6) | 5·5 (4·1–7·1) | -1·9 (-3·3–-0·9) | 37·6 (32·5–42·5) | 6·1 (2·4–9·5) | 15·4 (12·4–18·7) | 4·1 (2·6–5·8) |  |
|  | Syria | 32·7 (23·4–41·4) | -11·6 (-15·0–-8·3) | 4·7 (2·9–7·0) | -0·4 (-1·1–0·4) | 35·5 (28·3–43·3) | 5·5 (2·8–8·0) | 27·0 (21·2–33·0) | 6·5 (4·4–8·8) |  |
|  | Tunisia | 24·4 (15·9–32·9) | -11·1 (-15·1–-7·4) | 2·4 (1·3–3·9) | -1·1 (-1·8–-0·5) | 42·7 (35·6–50·6) | 5·9 (2·8–9·3) | 30·5 (24·6–37·1) | 6·3 (4·1–8·7) |  |
|  | Türkiye | 26·4 (18·8–34·1) | -16·0 (-20·2–-12·1) | 3·4 (2·0–5·4) | -3·5 (-4·8–-2·4) | 35·9 (29·5–43·5) | 8·7 (5·7–11·7) | 34·2 (27·8–40·4) | 10·9 (8·3–13·7) |  |
|  | United Arab Emirates | 24·0 (14·7–33·2) | -15·8 (-21·2–-9·9) | 4·4 (2·4–6·8) | 0·2 (-1·1–1·4) | 29·7 (23·5–36·1) | 5·3 (1·3–8·9) | 41·9 (34·4–50·4) | 10·3 (6·3–14·7) |  |
|  | Yemen | 46·4 (37·4–53·7) | -5·5 (-7·7–-3·1) | 5·2 (3·5–7·1) | -1·4 (-2·1–-0·7) | 29·8 (23·9–36·9) | 3·7 (2·1–5·5) | 18·6 (14·4–23·6) | 3·2 (2·0–4·4) |  |
|  | **South Asia** | **56·2 (48·8–63·0)** | **-12·0 (-14·3–-9·9)** | **1·9 (1·4–2·5)** | **-1·5 (-1·9–-1·2)** | **27·6 (22·1–33·3)** | **8·9 (7·2–10·9)** | **14·4 (11·2–18·0)** | **4·5 (3·5–5·8)** |  |
|  | Bangladesh | 49·0 (45·1–52·4) | -5·4 (-9·6–-1·4) | 4·2 (3·4–5·2) | -1·9 (-3·1–-0·9) | 27·9 (24·3–31·0) | 6·5 (3·0–10·0) | 18·8 (16·4–21·6) | 0·8 (-2·5–4·0) |  |
|  | Bhutan | 44·6 (37·8–50·6) | -16·9 (-22·0–-12·2) | 4·7 (3·4–6·3) | -3·0 (-4·2–-2·0) | 18·8 (14·8–23·1) | 5·4 (2·8–8·3) | 31·9 (27·4–37·4) | 14·5 (10·5–18·3) |  |
|  | India | 56·4 (48·3–63·9) | -13·9 (-16·8–-11·4) | 1·2 (0·9–1·6) | -1·1 (-1·5–-0·9) | 28·4 (22·3–34·7) | 9·8 (7·8–12·3) | 14·0 (10·7–17·8) | 5·3 (4·0–6·7) |  |
|  | Nepal | 58·6 (52·9–63·9) | -5·1 (-7·5–-2·7) | 7·6 (5·7–10·0) | -2·3 (-3·5–-1·3) | 23·0 (18·8–27·6) | 4·5 (2·7–6·3) | 10·8 (8·4–13·4) | 2·9 (1·8–3·9) |  |
|  | Pakistan | 58·8 (51·6–64·9) | -4·5 (-6·3–-2·9) | 5·2 (4·0–6·8) | -2·2 (-2·8–-1·7) | 21·3 (16·5–26·7) | 4·5 (3·1–6·0) | 14·7 (11·7–18·7) | 2·2 (1·4–3·3) |  |
|  | **Southeast Asia, east Asia, and Oceania** | **52·8 (45·9–59·0)** | **-10·0 (-13·1–-7·3)** | **5·8 (4·4–7·8)** | **-4·4 (-5·5–-3·5)** | **25·3 (20·2–31·1)** | **8·8 (6·8–11·3)** | **16·0 (12·7–19·8)** | **5·6 (4·1–7·0)** |  |
|  | East Asia | 54·6 (47·7–60·8) | -9·4 (-12·8–-6·7) | 6·1 (4·6–8·2) | -4·9 (-6·1–-3·9) | 25·4 (20·2–31·4) | 9·7 (7·5–12·1) | 13·9 (10·9–17·4) | 4·7 (3·4–6·1) |  |
|  | China | 54·8 (47·9–61·0) | -9·5 (-12·9–-6·7) | 6·1 (4·6–8·2) | -5·0 (-6·3–-4·0) | 25·3 (20·1–31·3) | 9·8 (7·6–12·3) | 13·8 (10·7–17·3) | 4·7 (3·4–6·1) |  |
|  | North Korea | 60·0 (53·7–65·2) | -4·5 (-6·4–-2·6) | 8·4 (6·6–11·3) | -2·8 (-3·5–-2·0) | 20·3 (15·9–25·1) | 4·5 (3·2–6·1) | 11·3 (8·8–14·3) | 2·7 (1·9–3·6) |  |
|  | Taiwan | 43·0 (34·7–50·8) | -11·3 (-14·6–-8·7) | 4·1 (2·7–5·6) | -1·2 (-1·9–-0·7) | 32·1 (25·5–39·4) | 6·9 (4·8–9·3) | 20·8 (16·4–25·9) | 5·6 (4·0–7·5) |  |
|  | Oceania | 54·6 (47·6–60·3) | -3·1 (-5·1–-1·3) | 8·1 (5·9–10·6) | -0·2 (-0·7–0·3) | 28·2 (22·9–34·7) | 2·3 (0·8–4·3) | 9·1 (6·8–11·7) | 1·0 (0·4–1·9) |  |
|  | American Samoa | 46·7 (43·4–49·5) | -9·8 (-13·7–-5·8) | 7·8 (6·6–9·1) | 1·3 (0·1–2·3) | 28·6 (25·9–31·6) | 3·5 (-0·3–6·9) | 17·0 (14·9–19·3) | 5·1 (3·1–7·3) |  |
|  | Cook Islands | 53·7 (47·1–58·9) | -8·3 (-12·1–-5·3) | 6·8 (5·1–8·9) | -1·5 (-2·3–-0·7) | 25·6 (20·9–31·4) | 5·2 (2·7–8·1) | 14·0 (11·1–17·2) | 4·6 (3·0–6·6) |  |
|  | Fiji | 39·0 (31·2–46·5) | -4·4 (-9·6–0·0) | 11·0 (7·5–15·1) | 1·0 (-1·0–3·3) | 35·9 (28·1–44·8) | 1·8 (-2·7–7·0) | 14·0 (10·4–18·1) | 1·6 (-0·6–4·1) |  |
|  | Guam | 39·2 (30·2–47·2) | -7·3 (-10·6–-4·3) | 3·9 (2·5–5·8) | 0·1 (-0·4–0·6) | 41·9 (34·6–50·0) | 4·3 (1·1–7·0) | 15·0 (11·4–19·0) | 2·9 (1·6–4·2) |  |
|  | Kiribati | 59·2 (53·7–64·0) | -1·9 (-3·9–0·0) | 12·4 (9·8–15·4) | 0·9 (-0·1–2·1) | 21·4 (17·2–26·2) | 0·2 (-1·3–2·1) | 7·0 (5·3–9·0) | 0·7 (0·0–1·5) |  |
|  | Marshall Islands | 59·7 (57·6–61·7) | -3·3 (-6·5–0·4) | 13·2 (12·1–14·5) | 0·3 (-2·0–2·2) | 22·0 (20·4–23·9) | 1·8 (-1·1–4·4) | 5·0 (4·3–5·9) | 1·2 (0·4–1·9) |  |
|  | Federated States of Micronesia | 62·5 (60·1–64·5) | -3·9 (-6·5–-1·8) | 11·7 (10·7–12·9) | 0·8 (-0·8–2·1) | 21·3 (19·5–23·8) | 3·2 (1·3–5·0) | 4·5 (3·8–5·4) | 0·0 (-1·1–0·8) |  |
|  | Nauru | 39·3 (34·2–45·2) | -4·6 (-6·8–-2·7) | 19·5 (15·2–24·2) | -3·0 (-5·0–-1·5) | 25·9 (21·1–31·3) | 5·4 (3·5–7·6) | 15·3 (12·1–19·3) | 2·3 (0·5–3·9) |  |
|  | Niue | 42·6 (34·6–49·5) | -0·9 (-2·8–1·0) | 10·5 (7·5–14·1) | 1·7 (0·8–2·5) | 35·6 (28·8–43·8) | -1·1 (-2·9–0·9) | 11·4 (8·5–14·7) | 0·3 (-0·5–1·2) |  |
|  | Northern Mariana Islands | 45·9 (36·7–52·7) | -12·3 (-17·4–-8·1) | 7·1 (4·7–10·1) | 1·3 (-0·1–3·0) | 34·3 (27·0–43·1) | 7·0 (2·8–11·7) | 12·7 (9·3–16·8) | 4·0 (1·7–6·3) |  |
|  | Palau | 46·6 (43·8–49·4) | -6·2 (-11·1–-0·7) | 10·8 (9·3–12·3) | 1·3 (-1·1–3·3) | 33·0 (30·2–36·0) | 2·9 (-2·8–7·7) | 9·6 (8·3–11·1) | 1·9 (0·1–3·6) |  |
|  | Papua New Guinea | 57·2 (49·4–63·4) | -3·9 (-6·1–-2·1) | 5·9 (4·3–8·0) | -0·8 (-1·4–-0·3) | 28·8 (23·3–35·6) | 3·5 (2·1–5·5) | 8·1 (5·9–10·7) | 1·2 (0·6–2·1) |  |
|  | Samoa | 63·5 (58·9–68·1) | 2·1 (-0·5–4·9) | 8·9 (7·1–11·0) | -0·8 (-2·0–0·3) | 19·4 (15·7–23·6) | -1·6 (-3·7–0·5) | 8·2 (6·2–10·3) | 0·2 (-0·7–1·3) |  |
|  | Solomon Islands | 63·3 (58·5–67·5) | -0·9 (-2·4–0·5) | 19·8 (16·3–24·0) | 0·6 (-0·8–2·1) | 12·5 (10·0–15·4) | 0·2 (-0·5–0·9) | 4·4 (3·4–5·8) | 0·1 (-0·2–0·6) |  |
|  | Tokelau | 40·3 (35·4–45·8) | -0·7 (-3·3–1·6) | 16·4 (12·9–20·1) | 1·8 (0·3–3·4) | 35·9 (30·9–41·8) | -1·3 (-4·2–1·7) | 7·4 (5·7–9·3) | 0·2 (-0·7–1·0) |  |
|  | Tonga | 49·4 (42·1–55·9) | -3·2 (-5·4–-1·5) | 10·9 (8·0–14·1) | 0·7 (-0·1–1·8) | 31·3 (24·9–38·9) | 1·4 (-0·3–3·6) | 8·3 (6·1–10·9) | 1·0 (0·4–1·8) |  |
|  | Tuvalu | 41·7 (35·0–47·7) | -2·5 (-4·9–0·1) | 11·2 (7·9–14·9) | -0·2 (-1·4–1·3) | 35·0 (28·4–42·2) | 0·7 (-2·2–3·1) | 12·1 (9·1–15·6) | 2·0 (0·9–3·3) |  |
|  | Vanuatu | 75·2 (70·8–78·8) | -1·8 (-2·7–-0·8) | 5·5 (4·2–7·2) | -0·1 (-0·5–0·3) | 11·0 (8·6–13·9) | 0·9 (0·3–1·5) | 8·3 (6·3–10·6) | 1·0 (0·4–1·6) |  |
|  | Southeast Asia | 47·6 (40·6–53·9) | -9·8 (-12·5–-7·5) | 5·1 (3·6–6·9) | -2·0 (-2·6–-1·6) | 25·4 (20·3–30·8) | 5·7 (4·0–7·8) | 21·9 (18·0–26·5) | 6·1 (4·4–7·7) |  |
|  | Cambodia | 38·5 (29·8–45·7) | -9·7 (-14·2–-6·0) | 6·9 (4·4–9·8) | -4·9 (-6·5–-3·6) | 26·5 (20·9–33·1) | 6·6 (3·9–9·9) | 28·1 (23·3–34·2) | 8·0 (5·5–11·0) |  |
|  | Indonesia | 51·9 (43·7–58·4) | -8·8 (-11·5–-6·8) | 5·9 (4·2–8·0) | -1·7 (-2·3–-1·3) | 23·8 (18·3–29·5) | 5·4 (3·8–7·3) | 18·3 (14·5–22·9) | 5·2 (3·8–6·8) |  |
|  | Laos | 46·8 (40·8–52·3) | -6·7 (-9·0–-4·5) | 8·2 (6·0–10·7) | -5·8 (-7·4–-4·5) | 27·0 (22·2–32·1) | 6·8 (5·0–9·0) | 18·1 (14·6–22·0) | 5·7 (4·3–7·2) |  |
|  | Malaysia | 52·6 (44·5–60·0) | -11·9 (-19·5–-5·5) | 1·7 (1·1–2·3) | -0·6 (-1·0–-0·1) | 21·2 (16·4–27·0) | 5·0 (1·3–9·7) | 24·6 (19·6–29·8) | 7·4 (2·9–12·3) |  |
|  | Maldives | 56·7 (50·3–62·0) | -6·0 (-8·8–-3·4) | 3·9 (2·9–5·3) | -2·6 (-3·6–-1·8) | 21·5 (17·3–25·8) | 4·0 (2·2–6·0) | 17·9 (14·5–21·9) | 4·6 (3·1–6·5) |  |
|  | Mauritius | 39·8 (30·6–47·4) | -8·5 (-12·4–-5·1) | 4·4 (2·7–6·3) | -0·5 (-1·3–0·1) | 29·2 (22·8–36·4) | 3·1 (0·8–5·8) | 26·6 (21·2–32·9) | 6·0 (3·8–8·3) |  |
|  | Myanmar | 43·7 (38·3–48·8) | -10·8 (-14·5–-6·8) | 6·2 (4·6–8·2) | -5·0 (-7·1–-3·6) | 28·2 (23·6–32·9) | 8·5 (5·6–11·1) | 22·0 (17·8–26·2) | 7·3 (5·0–9·9) |  |
|  | Philippines | 52·2 (44·5–58·7) | -5·1 (-6·6–-3·8) | 5·4 (3·8–7·3) | -0·6 (-0·8–-0·3) | 23·9 (18·4–29·6) | 2·6 (1·7–3·7) | 18·5 (14·7–23·0) | 3·0 (2·1–3·9) |  |
|  | Seychelles | 39·4 (30·3–47·7) | -7·9 (-13·8–-3·4) | 3·0 (1·8–4·6) | -0·3 (-1·0–0·3) | 46·0 (38·5–55·4) | 5·8 (1·7–11·3) | 11·6 (8·8–15·1) | 2·5 (1·0–4·2) |  |
|  | Sri Lanka | 26·8 (21·8–31·9) | -14·0 (-19·0–-9·1) | 7·1 (4·7–9·5) | -6·8 (-9·8–-4·5) | 30·6 (26·2–35·8) | 8·3 (4·9–12·0) | 35·5 (30·9–40·1) | 12·6 (8·8–16·7) |  |
|  | Thailand | 40·4 (30·9–49·2) | -16·2 (-22·8–-8·8) | 2·0 (1·3–2·8) | -0·3 (-0·8–0·2) | 36·3 (30·0–43·7) | 9·4 (4·1–15·1) | 21·3 (16·5–26·0) | 7·1 (3·0–10·7) |  |
|  | Timor-Leste | 75·3 (70·3–79·8) | -2·4 (-4·4–-0·9) | 4·6 (3·3–6·6) | -1·8 (-3·0–-1·0) | 11·0 (7·9–14·2) | 2·0 (1·2–3·0) | 9·2 (6·7–12·1) | 2·2 (1·3–3·4) |  |
|  | Viet Nam | 48·7 (42·1–54·6) | -8·8 (-12·7–-4·1) | 4·5 (3·2–5·9) | -2·2 (-3·2–-1·2) | 17·4 (13·4–21·7) | 4·4 (2·3–7·2) | 29·4 (24·9–34·9) | 6·6 (3·5–9·7) |  |
|  | **Sub-Saharan Africa** | **65·8 (61·2–70·3)** | **-0·8 (-1·7–-0·2)** | **6·5 (5·1–8·3)** | **-1·7 (-2·1–-1·3)** | **14·3 (11·3–17·6)** | **1·0 (0·7–1·5)** | **13·4 (10·9–16·3)** | **1·5 (1·1–1·9)** |  |
|  | Central sub-Saharan Africa | 83·7 (81·1–86·0) | -0·6 (-1·4–0·2) | 5·0 (4·0–6·5) | -1·6 (-2·2–-1·2) | 5·6 (4·3–7·0) | 1·0 (0·7–1·4) | 5·7 (4·6–7·2) | 1·3 (0·9–1·7) |  |
|  | Angola | 81·8 (79·4–84·2) | 0·3 (-0·8–1·6) | 6·8 (5·5–8·2) | -2·6 (-3·6–-1·8) | 4·5 (3·3–5·7) | 0·7 (0·3–1·0) | 7·0 (5·6–8·6) | 1·7 (1·2–2·2) |  |
|  | Central African Republic | 84·1 (81·8–86·1) | -1·3 (-2·0–-0·6) | 3·6 (2·9–4·7) | -0·3 (-0·5–0·0) | 5·7 (4·5–6·9) | 0·5 (0·1–0·9) | 6·6 (5·5–8·1) | 1·0 (0·6–1·5) |  |
|  | Congo (Brazzaville) | 82·8 (80·4–85·1) | -0·9 (-1·8–0·0) | 5·0 (3·9–6·3) | -1·4 (-2·0–-1·0) | 5·6 (4·4–7·0) | 1·0 (0·6–1·4) | 6·6 (5·4–8·1) | 1·4 (0·9–1·9) |  |
|  | DR Congo | 84·7 (82·4–87·0) | -0·6 (-1·5–0·2) | 4·3 (3·3–5·7) | -1·7 (-2·4–-1·2) | 5·9 (4·5–7·4) | 1·2 (0·9–1·7) | 5·1 (4·1–6·3) | 1·0 (0·7–1·4) |  |
|  | Equatorial Guinea | 83·6 (81·1–86·0) | -0·4 (-1·5–0·6) | 3·7 (2·8–4·7) | -2·1 (-2·9–-1·6) | 5·8 (4·6–7·2) | 1·0 (0·6–1·5) | 7·0 (5·6–8·4) | 1·6 (1·0–2·0) |  |
|  | Gabon | 80·1 (77·3–82·9) | -1·8 (-2·9–-0·7) | 5·7 (4·5–7·5) | -1·0 (-1·6–-0·6) | 6·1 (4·7–7·6) | 1·0 (0·6–1·4) | 8·1 (6·7–9·9) | 1·8 (1·4–2·5) |  |
|  | Eastern sub-Saharan Africa | 62·2 (57·4–67·0) | -2·0 (-3·1–-1·1) | 7·6 (6·0–9·7) | -2·4 (-3·0–-1·9) | 13·6 (10·8–16·8) | 1·8 (1·4–2·3) | 16·5 (13·6–19·9) | 2·6 (2·1–3·1) |  |
|  | Burundi | 63·3 (57·7–68·3) | 0·0 (-1·8–1·6) | 6·2 (4·7–8·1) | -2·5 (-3·3–-1·9) | 14·3 (11·4–17·9) | 1·3 (0·4–2·2) | 16·1 (13·1–19·8) | 1·2 (0·3–2·3) |  |
|  | Comoros | 46·9 (40·0–54·1) | -5·4 (-7·5–-3·4) | 4·8 (3·2–6·8) | -1·4 (-2·0–-0·8) | 26·7 (21·2–32·3) | 3·1 (1·5–4·6) | 21·6 (17·1–26·5) | 3·7 (2·3–5·0) |  |
|  | Djibouti | 58·0 (51·2–63·8) | -6·7 (-9·2–-4·7) | 5·8 (4·3–7·7) | -1·9 (-2·5–-1·3) | 16·5 (12·8–20·7) | 3·6 (2·4–4·9) | 19·6 (16·0–24·0) | 5·0 (3·8–6·6) |  |
|  | Eritrea | 41·3 (33·4–48·3) | -7·6 (-11·7–-3·8) | 4·2 (2·6–6·5) | -1·7 (-2·7–-0·7) | 26·0 (20·9–32·0) | 4·3 (1·7–7·0) | 28·5 (23·7–34·1) | 5·0 (2·5–7·4) |  |
|  | Ethiopia | 77·5 (74·7–80·9) | 0·8 (-0·5–2·3) | 6·7 (5·4–8·4) | -4·8 (-5·9–-3·9) | 7·1 (5·6–8·8) | 1·7 (1·3–2·1) | 8·6 (7·0–10·4) | 2·3 (1·8–3·0) |  |
|  | Kenya | 53·6 (47·2–59·3) | -0·5 (-1·3–0·3) | 6·9 (5·2–9·0) | 0·8 (0·6–1·1) | 15·8 (12·2–19·6) | -1·3 (-1·7–-0·9) | 23·8 (19·7–28·7) | 0·9 (0·4–1·7) |  |
|  | Madagascar | 62·1 (56·6–67·2) | -1·8 (-3·7–-0·1) | 5·6 (4·2–7·3) | -1·6 (-2·3–-1·1) | 15·3 (11·9–19·0) | 1·6 (0·6–2·6) | 17·1 (13·9–20·6) | 1·9 (0·9–3·1) |  |
|  | Malawi | 53·6 (48·1–59·1) | -0·8 (-2·7–1·2) | 10·1 (7·6–13·2) | -3·2 (-4·6–-2·2) | 14·0 (10·5–18·0) | 1·3 (0·3–2·4) | 22·3 (18·1–27·4) | 2·6 (1·1–4·1) |  |
|  | Mozambique | 56·0 (48·6–63·1) | -0·5 (-2·9–1·4) | 5·5 (4·0–7·5) | -1·1 (-1·8–-0·6) | 13·2 (8·8–18·1) | 0·6 (-0·5–1·8) | 25·3 (19·9–31·5) | 1·0 (-0·6–2·9) |  |
|  | Rwanda | 66·0 (61·4–70·7) | -5·5 (-7·7–-3·6) | 5·3 (4·0–7·2) | -3·7 (-4·8–-2·8) | 9·6 (6·9–13·0) | 2·6 (1·6–3·8) | 19·1 (15·5–23·2) | 6·5 (5·1–8·3) |  |
|  | Somalia | 63·6 (59·2–68·3) | -0·8 (-2·2–0·7) | 10·3 (8·2–13·0) | -0·4 (-1·3–0·3) | 11·8 (9·2–14·7) | 0·2 (-0·5–0·9) | 14·3 (11·5–17·5) | 0·9 (0·0–1·9) |  |
|  | South Sudan | 63·1 (57·9–67·8) | -1·2 (-3·1–0·6) | 7·7 (5·9–9·8) | -0·3 (-0·9–0·3) | 13·6 (10·6–16·9) | 0·7 (-0·2–1·7) | 15·6 (12·5–19·2) | 0·7 (-0·5–2·0) |  |
|  | Uganda | 52·9 (47·5–58·2) | -4·0 (-6·2–-1·8) | 7·2 (5·2–9·4) | -3·5 (-4·6–-2·4) | 13·2 (10·3–16·4) | 2·5 (1·4–3·6) | 26·8 (22·5–31·6) | 5·0 (3·2–6·7) |  |
|  | Tanzania | 51·8 (46·3–57·6) | -2·5 (-4·4–-0·8) | 11·0 (8·4–14·4) | -1·9 (-2·8–-1·0) | 21·3 (17·2–26·3) | 2·2 (1·1–3·6) | 15·8 (12·5–19·5) | 2·2 (1·3–3·4) |  |
|  | Zambia | 74·8 (71·5–78·1) | 1·2 (-0·5–3·0) | 6·0 (4·8–7·4) | -2·7 (-3·7–-2·0) | 12·9 (10·4–15·4) | 0·9 (-0·2–2·2) | 6·2 (4·8–7·7) | 0·6 (0·0–1·3) |  |
|  | Southern sub-Saharan Africa | 47·6 (40·2–55·0) | -0·5 (-1·8–0·6) | 5·9 (4·2–8·1) | 0·6 (0·4–1·0) | 29·5 (23·3–36·2) | 0·8 (-0·1–1·9) | 17·1 (13·3–21·2) | -0·9 (-1·5–-0·3) |  |
|  | Botswana | 48·2 (41·8–53·8) | -7·0 (-9·3–-4·5) | 4·3 (3·0–5·9) | 0·3 (-0·1–0·8) | 22·0 (17·7–26·7) | 1·6 (0·0–3·2) | 25·5 (21·7–30·1) | 5·1 (3·3–6·6) |  |
|  | Eswatini | 53·2 (46·7–59·4) | -5·7 (-8·0–-3·5) | 2·8 (2·0–3·8) | 0·1 (-0·2–0·4) | 22·0 (17·6–26·6) | 2·9 (1·4–4·4) | 22·0 (18·4–26·2) | 2·7 (1·1–4·2) |  |
|  | Lesotho | 42·8 (35·6–49·8) | 1·0 (-1·2–2·8) | 4·4 (3·2–6·1) | 0·8 (0·5–1·3) | 28·8 (23·2–35·4) | -1·4 (-3·0–-0·1) | 24·1 (19·5–28·6) | -0·4 (-1·6–0·9) |  |
|  | Namibia | 45·8 (37·6–53·8) | -4·5 (-7·3–-1·6) | 2·7 (1·8–3·6) | -0·4 (-0·8–0·0) | 32·2 (26·2–38·8) | 2·0 (-0·1–4·1) | 19·4 (15·6–23·9) | 2·8 (1·3–4·4) |  |
|  | South Africa | 47·2 (39·6–54·7) | -0·3 (-1·8–0·9) | 6·3 (4·5–8·6) | 0·5 (0·3–0·9) | 30·2 (23·9–37·1) | 1·0 (0·0–2·3) | 16·4 (12·6–20·6) | -1·2 (-1·9–-0·5) |  |
|  | Zimbabwe | 51·5 (44·4–58·4) | 1·2 (-0·8–3·4) | 3·8 (2·7–5·1) | 1·4 (1·0–1·9) | 25·4 (20·2–31·1) | -2·0 (-3·3–-0·7) | 19·4 (15·1–23·9) | -0·5 (-1·7–0·6) |  |
|  | Western sub-Saharan Africa | 68·0 (63·5–72·2) | -1·6 (-2·7–-0·7) | 6·3 (4·9–8·0) | -2·1 (-2·7–-1·8) | 13·3 (10·5–16·4) | 1·7 (1·1–2·3) | 12·4 (10·1–15·0) | 2·1 (1·6–2·8) |  |
|  | Benin | 74·1 (70·7–77·8) | -0·1 (-1·6–1·4) | 3·8 (3·0–4·8) | -1·7 (-2·3–-1·2) | 10·5 (8·4–12·8) | 0·7 (0·0–1·6) | 11·5 (9·4–13·9) | 1·0 (0·2–1·9) |  |
|  | Burkina Faso | 69·4 (65·4–73·5) | -0·6 (-1·8–0·5) | 6·1 (4·7–7·8) | -1·6 (-2·3–-1·1) | 12·9 (10·2–16·1) | 1·1 (0·5–1·9) | 11·7 (9·3–14·3) | 1·1 (0·5–1·7) |  |
|  | Cabo Verde | 42·6 (37·0–48·2) | -10·1 (-14·0–-6·1) | 4·9 (3·6–6·7) | -1·6 (-2·7–-0·6) | 31·5 (26·6–36·6) | 6·8 (3·9–9·9) | 21·0 (16·8–24·7) | 4·8 (2·4–7·0) |  |
|  | Cameroon | 58·7 (53·1–64·2) | -1·0 (-3·9–2·2) | 5·1 (3·8–6·6) | -1·9 (-2·8–-1·2) | 16·3 (12·8–20·2) | 1·0 (-0·6–2·6) | 20·0 (16·4–23·9) | 1·9 (-0·3–4·0) |  |
|  | Chad | 74·0 (70·0–77·3) | 1·1 (0·0–2·1) | 6·5 (5·1–8·2) | -1·8 (-2·5–-1·2) | 10·6 (8·4–13·3) | 0·5 (0·0–1·0) | 8·9 (7·1–10·9) | 0·3 (-0·2–0·7) |  |
|  | Côte d'Ivoire | 72·5 (68·0–75·9) | -1·7 (-3·2–-0·3) | 6·5 (5·0–8·3) | -1·8 (-2·4–-1·3) | 11·1 (8·7–13·7) | 1·6 (1·0–2·4) | 10·0 (8·1–12·2) | 1·8 (1·3–2·6) |  |
|  | The Gambia | 72·1 (68·4–75·8) | -1·0 (-1·8–0·0) | 6·6 (5·1–8·3) | -1·0 (-1·7–-0·6) | 11·0 (8·8–13·6) | 0·8 (0·3–1·4) | 10·3 (8·3–12·5) | 1·2 (0·6–1·7) |  |
|  | Ghana | 49·5 (43·3–55·2) | -1·8 (-4·1–0·3) | 10·4 (7·8–13·3) | -2·5 (-3·6–-1·6) | 22·3 (17·4–27·1) | 2·0 (0·3–3·4) | 17·7 (13·9–21·7) | 2·3 (1·1–3·8) |  |
|  | Guinea | 65·5 (60·7–70·0) | 2·2 (0·2–4·0) | 8·6 (6·6–11·1) | -2·9 (-3·9–-2·1) | 12·2 (9·2–15·4) | 1·0 (-0·1–2·3) | 13·7 (10·9–16·8) | -0·3 (-1·7–1·0) |  |
|  | Guinea-Bissau | 74·0 (70·5–77·4) | 0·4 (-0·7–1·6) | 8·1 (6·4–10·1) | -2·4 (-3·1–-1·7) | 9·5 (7·6–12·0) | 1·0 (0·5–1·6) | 8·3 (6·8–10·2) | 1·0 (0·5–1·5) |  |
|  | Liberia | 68·1 (63·1–72·6) | -1·4 (-3·3–0·7) | 5·2 (3·7–7·2) | -1·6 (-2·5–-0·7) | 13·3 (10·3–16·5) | 1·3 (0·1–2·7) | 13·4 (10·8–16·9) | 1·6 (0·2–3·2) |  |
|  | Mali | 76·7 (73·3–80·0) | -1·7 (-3·1–-0·5) | 5·9 (4·6–7·6) | -1·0 (-1·6–-0·4) | 10·6 (8·0–13·4) | 1·5 (0·8–2·4) | 6·8 (5·2–8·7) | 1·1 (0·6–1·8) |  |
|  | Mauritania | 72·6 (66·9–77·5) | -2·3 (-4·6–-0·6) | 6·9 (5·3–9·1) | -2·0 (-2·8–-1·4) | 9·6 (6·8–13·1) | 1·6 (0·8–2·8) | 10·9 (7·9–14·5) | 2·7 (1·7–4·3) |  |
|  | Niger | 89·3 (87·1–91·5) | -0·6 (-1·4–0·2) | 3·4 (2·3–4·9) | -0·9 (-1·6–-0·3) | 4·1 (3·0–5·5) | 0·8 (0·6–1·2) | 3·2 (2·4–4·4) | 0·7 (0·5–1·1) |  |
|  | Nigeria | 67·7 (63·2–72·2) | -2·0 (-3·4–-0·9) | 5·9 (4·6–7·6) | -2·6 (-3·2–-2·1) | 13·5 (10·6–16·7) | 2·0 (1·4–2·7) | 12·9 (10·4–15·6) | 2·6 (2·0–3·3) |  |
|  | São Tomé and Príncipe | 65·4 (61·0–69·7) | -0·5 (-2·5–1·9) | 8·2 (6·4–10·3) | -2·4 (-3·4–-1·6) | 13·1 (10·2–16·1) | 1·8 (0·4–3·3) | 13·4 (10·7–16·5) | 1·2 (-0·3–2·4) |  |
|  | Senegal | 70·4 (66·2–74·1) | -1·8 (-3·3–-0·5) | 5·9 (4·5–7·5) | -1·7 (-2·3–-1·3) | 12·4 (9·8–15·2) | 1·7 (1·0–2·5) | 11·4 (9·2–13·9) | 1·9 (1·2–2·7) |  |
|  | Sierra Leone | 72·2 (68·0–75·7) | 2·7 (1·2–4·3) | 6·1 (4·7–7·8) | -2·5 (-3·6–-1·9) | 11·6 (9·2–14·2) | -0·1 (-0·9–0·9) | 10·2 (8·2–12·6) | -0·1 (-0·9–0·7) |  |
|  | Togo | 69·6 (65·3–73·6) | -3·9 (-5·4–-2·6) | 6·7 (5·2–8·6) | -1·2 (-1·7–-0·6) | 12·4 (9·8–15·4) | 2·3 (1·6–3·2) | 11·3 (9·2–13·9) | 2·7 (1·9–3·5) |  |
|  |  |  |  |  |  |  |  |  |  |  |

## Table S15. Diagnosed proportion among people living with diabetes, treated proportion among people with diagnosed diabetes, and optimal glycaemic management proportion among people with treated diabetes in 2023, males and females for ages 15 and older globally, and by Global Burden of Disease super-regions, regions, and countries and territories

|  | **Location** | **Diagnosed, 2023 Males (%)** | **Diagnosed, 2023 Females (%)** | **Treated, 2023 Males (%)** | **Treated, 2023 Females (%)** | **Optimal Glycaemic Management, 2023 Males (%)** | **Optimal Glycaemic Management, 2023 Females (%)** |  |
| --- | --- | --- | --- | --- | --- | --- | --- | --- |
|  | **Global** | **51·8 (45·6–58·0)** | **59·8 (53·0–66·6)** | **90·0 (86·3–93·0)** | **92·7 (89·4–95·3)** | **41·1 (35·1–47·9)** | **42·1 (36·2–49·1)** |  |
|  | **Central Europe, eastern Europe, and central Asia** | **64·8 (56·6–73·3)** | **70·3 (62·1–78·0)** | **90·7 (85·6–94·7)** | **93·1 (88·7–96·2)** | **49·4 (42·6–56·7)** | **51·3 (44·5–58·8)** |  |
|  | Central Asia | 53·0 (46·9–59·8) | 55·4 (49·5–61·7) | 87·1 (81·0–91·8) | 89·6 (84·7–93·4) | 39·1 (32·5–46·1) | 44·5 (38·2–50·7) |  |
|  | Armenia | 60·1 (51·7–69·1) | 66·7 (58·9–74·2) | 90·7 (84·1–95·1) | 91·7 (86·3–95·2) | 37·8 (30·4–46·2) | 42·7 (36·2–48·8) |  |
|  | Azerbaijan | 59·3 (52·6–67·4) | 71·0 (64·2–78·2) | 91·8 (87·7–95·3) | 95·5 (92·5–97·6) | 32·9 (26·2–40·6) | 26·5 (21·5–32·1) |  |
|  | Georgia | 73·8 (67·9–79·8) | 73·1 (66·5–80·2) | 86·8 (81·2–91·8) | 94·0 (91·0–96·6) | 44·0 (37·1–51·5) | 45·6 (39·6–51·6) |  |
|  | Kazakhstan | 65·4 (57·6–74·8) | 54·5 (47·5–62·0) | 93·7 (87·9–97·3) | 89·9 (84·7–93·7) | 55·2 (47·6–62·4) | 81·1 (76·8–84·9) |  |
|  | Kyrgyzstan | 53·3 (46·8–61·5) | 59·9 (52·7–67·9) | 87·2 (81·5–92·0) | 97·1 (95·2–98·5) | 19·5 (14·7–24·6) | 23·7 (19·0–29·2) |  |
|  | Mongolia | 42·8 (37·8–49·0) | 47·0 (41·3–52·3) | 78·9 (72·6–84·6) | 77·7 (71·9–84·0) | 39·7 (33·8–47·3) | 38·5 (33·0–45·0) |  |
|  | Tajikistan | 36·2 (31·7–41·0) | 41·3 (36·4–46·3) | 62·6 (54·3–70·6) | 66·1 (57·4–74·0) | 26·7 (19·6–36·0) | 19·7 (13·6–27·8) |  |
|  | Turkmenistan | 27·1 (23·1–31·3) | 30·4 (26·6–34·7) | 83·0 (76·9–87·6) | 80·5 (74·0–86·1) | 22·0 (16·6–28·9) | 28·6 (23·2–35·0) |  |
|  | Uzbekistan | 48·7 (42·1–57·1) | 51·1 (44·2–59·4) | 85·1 (78·4–90·8) | 88·6 (82·9–93·3) | 33·7 (28·1–40·9) | 37·5 (31·5–45·0) |  |
|  | Central Europe | 72·2 (63·1–81·0) | 71·8 (62·9–80·8) | 91·7 (86·4–95·6) | 92·1 (86·7–95·7) | 46·6 (39·4–54·2) | 48·9 (41·9–56·4) |  |
|  | Albania | 68·7 (59·0–79·2) | 67·1 (57·0–78·4) | 90·7 (84·5–95·3) | 90·4 (83·9–95·2) | 43·9 (36·4–53·0) | 41·2 (34·3–49·1) |  |
|  | Bosnia and Herzegovina | 67·3 (57·7–78·5) | 66·2 (56·5–77·4) | 88·8 (81·3–94·4) | 88·5 (81·4–94·1) | 44·0 (37·0–51·7) | 41·4 (34·5–49·6) |  |
|  | Bulgaria | 64·7 (56·0–74·6) | 63·9 (55·0–75·0) | 85·5 (78·4–92·0) | 85·2 (77·1–91·8) | 44·2 (36·8–52·1) | 42·0 (34·8–49·8) |  |
|  | Croatia | 74·7 (63·8–85·3) | 73·5 (62·7–84·0) | 93·6 (88·3–97·5) | 93·3 (87·6–96·8) | 45·5 (38·4–53·1) | 43·1 (35·7–51·3) |  |
|  | Czechia | 62·0 (53·3–72·6) | 62·3 (54·1–72·5) | 85·9 (77·8–91·9) | 81·6 (73·4–89·2) | 39·1 (32·3–46·7) | 28·8 (22·9–35·7) |  |
|  | Hungary | 69·2 (59·7–79·1) | 68·0 (58·9–77·8) | 89·5 (83·1–94·3) | 89·2 (82·9–94·2) | 45·4 (38·7–53·9) | 43·2 (36·1–50·3) |  |
|  | Montenegro | 71·0 (61·0–81·7) | 69·7 (59·9–80·0) | 91·2 (85·2–96·1) | 91·0 (84·7–95·4) | 45·2 (38·6–52·8) | 42·5 (35·5–49·7) |  |
|  | North Macedonia | 66·6 (57·9–77·1) | 65·0 (55·6–75·9) | 87·7 (80·3–93·7) | 87·3 (80·5–93·2) | 44·5 (36·6–51·6) | 41·5 (34·4–49·0) |  |
|  | Poland | 79·2 (69·2–87·8) | 78·3 (68·4–87·0) | 95·2 (91·0–97·9) | 96·3 (93·2–98·4) | 49·5 (42·1–57·0) | 56·1 (49·2–63·4) |  |
|  | Romania | 66·5 (57·5–76·9) | 65·3 (56·1–75·5) | 88·1 (81·3–93·5) | 87·7 (80·5–93·5) | 44·1 (37·1–52·1) | 41·8 (34·8–49·6) |  |
|  | Serbia | 66·4 (57·1–76·4) | 65·0 (56·1–75·6) | 86·8 (79·8–92·9) | 86·4 (78·9–92·9) | 44·8 (38·0–52·4) | 42·0 (34·3–49·8) |  |
|  | Slovakia | 68·5 (59·3–79·2) | 67·4 (58·0–77·4) | 90·0 (83·7–95·0) | 89·7 (83·6–94·6) | 44·2 (37·1–51·4) | 42·0 (35·0–49·4) |  |
|  | Slovenia | 79·5 (69·7–89·1) | 78·7 (68·8–87·7) | 96·3 (93·0–98·5) | 96·2 (93·0–98·4) | 45·5 (38·6–53·1) | 43·2 (36·4–51·5) |  |
|  | Eastern Europe | 62·8 (54·2–71·6) | 74·2 (65·4–81·9) | 90·8 (85·9–94·7) | 94·6 (90·6–97·2) | 56·2 (49·5–63·6) | 54·7 (47·9–62·1) |  |
|  | Belarus | 77·0 (71·9–81·3) | 77·7 (72·2–82·5) | 85·7 (81·4–89·9) | 90·0 (85·6–93·7) | 60·5 (54·1–67·0) | 60·2 (54·0–67·0) |  |
|  | Estonia | 67·6 (58·3–78·4) | 76·8 (67·1–85·8) | 92·9 (88·4–96·5) | 94·9 (90·8–97·7) | 48·5 (41·2–56·2) | 51·9 (45·1–59·2) |  |
|  | Latvia | 61·3 (53·0–71·3) | 71·0 (62·0–80·8) | 87·6 (81·3–93·0) | 90·5 (84·8–95·4) | 48·4 (40·9–56·3) | 51·4 (44·2–59·2) |  |
|  | Lithuania | 65·8 (56·0–76·5) | 71·2 (62·1–80·0) | 94·4 (90·5–97·3) | 92·4 (87·4–96·1) | 41·4 (35·0–49·4) | 40·0 (32·8–47·9) |  |
|  | Moldova | 49·7 (44·1–56·3) | 64·1 (58·1–70·3) | 77·5 (70·6–83·6) | 79·9 (73·4–86·0) | 30·5 (24·7–37·5) | 53·4 (46·8–60·6) |  |
|  | Russia | 63·3 (53·2–73·4) | 77·2 (67·1–85·7) | 95·1 (92·0–97·5) | 96·8 (94·0–98·6) | 57·5 (50·5–64·8) | 56·2 (49·2–63·7) |  |
|  | Ukraine | 55·5 (48·6–62·9) | 60·8 (53·1–69·0) | 74·9 (66·6–82·7) | 86·5 (79·9–92·0) | 53·3 (46·1–60·8) | 44·7 (38·4–52·7) |  |
|  | **High income** | **78·6 (71·7–85·3)** | **80·4 (73·8–86·3)** | **91·7 (86·7–95·5)** | **91·4 (86·5–94·9)** | **43·2 (37·3–50·1)** | **46·7 (40·5–53·2)** |  |
|  | Australasia | 72·2 (64·8–80·5) | 69·1 (61·4–76·9) | 86·8 (79·8–92·7) | 85·5 (78·7–91·1) | 34·3 (28·1–40·8) | 32·6 (26·7–39·3) |  |
|  | Australia | 72·3 (64·5–80·7) | 69·3 (61·0–77·5) | 87·1 (80·2–92·9) | 85·9 (79·0–91·7) | 34·5 (28·2–40·9) | 32·9 (26·8–39·5) |  |
|  | New Zealand | 71·7 (64·5–79·2) | 68·5 (60·9–76·2) | 85·6 (78·0–92·1) | 84·5 (77·4–90·5) | 33·3 (27·3–40·5) | 31·6 (26·0–38·0) |  |
|  | High-income Asia Pacific | 75·2 (67·9–81·6) | 75·8 (69·0–82·4) | 95·8 (93·6–97·5) | 97·1 (95·5–98·3) | 48·7 (43·2–54·4) | 52·8 (47·4–58·3) |  |
|  | Brunei | 73·7 (67·3–79·5) | 74·0 (65·7–81·1) | 85·3 (79·4–90·5) | 96·2 (93·9–97·8) | 37·7 (31·1–44·8) | 38·8 (32·8–45·2) |  |
|  | Japan | 76·2 (67·0–84·6) | 73·5 (64·4–82·6) | 95·9 (93·3–98·0) | 97·0 (94·8–98·5) | 49·8 (42·9–56·9) | 52·7 (45·7–59·8) |  |
|  | South Korea | 72·7 (69·8–75·1) | 81·9 (79·8–84·3) | 95·6 (94·5–96·5) | 97·7 (97·0–98·3) | 46·9 (44·3–49·6) | 54·4 (51·5–57·0) |  |
|  | Singapore | 74·8 (67·4–83·0) | 74·5 (66·1–82·9) | 96·1 (93·7–98·0) | 96·1 (93·7–97·9) | 38·6 (32·5–45·7) | 33·8 (28·5–40·1) |  |
|  | High-income North America | 82·3 (74·8–89·0) | 83·5 (76·4–89·6) | 92·7 (87·7–96·3) | 92·1 (86·8–95·7) | 43·6 (37·4–50·7) | 50·0 (43·4–57·1) |  |
|  | Canada | 85·0 (78·2–91·5) | 86·9 (80·0–92·8) | 96·4 (93·4–98·3) | 96·4 (93·3–98·5) | 43·3 (36·6–50·3) | 46·2 (39·4–52·8) |  |
|  | Greenland | 71·1 (63·7–80·0) | 74·1 (66·8–81·8) | 82·6 (74·0–90·1) | 82·9 (74·6–90·1) | 41·0 (33·9–48·8) | 43·7 (36·8–50·9) |  |
|  | USA | 82·1 (74·6–88·9) | 83·4 (76·3–89·5) | 92·5 (87·3–96·2) | 91·8 (86·3–95·6) | 43·6 (37·5–50·7) | 50·3 (43·6–57·3) |  |
|  | Southern Latin America | 78·2 (72·3–83·6) | 81·3 (75·9–86·5) | 87·6 (82·1–92·6) | 94·9 (92·5–96·9) | 68·0 (62·1–73·2) | 58·7 (53·4–63·7) |  |
|  | Argentina | 80·0 (73·7–85·8) | 79·6 (73·3–85·3) | 86·9 (80·9–92·4) | 96·6 (94·8–97·9) | 72·0 (66·6–77·1) | 57·2 (52·0–62·5) |  |
|  | Chile | 77·5 (72·1–83·0) | 87·3 (81·6–92·9) | 92·0 (87·9–95·0) | 91·2 (85·6–95·8) | 54·7 (48·3–60·9) | 60·6 (54·3–65·3) |  |
|  | Uruguay | 56·9 (49·6–64·5) | 77·8 (71·3–84·5) | 83·3 (77·1–88·9) | 92·3 (88·4–95·7) | 62·5 (55·7–68·8) | 67·7 (62·1–72·9) |  |
|  | Western Europe | 76·2 (68·8–83·2) | 78·6 (72·2–84·6) | 88·8 (83·3–93·8) | 87·3 (81·4–92·3) | 37·9 (31·9–45·0) | 36·4 (30·4–43·2) |  |
|  | Andorra | 78·3 (70·4–86·0) | 81·4 (74·0–87·6) | 94·0 (89·7–96·9) | 92·9 (88·3–96·2) | 36·6 (30·4–43·7) | 35·2 (29·8–41·8) |  |
|  | Austria | 77·5 (68·9–84·6) | 80·1 (71·8–88·0) | 92·7 (87·1–96·4) | 91·4 (85·8–95·8) | 37·3 (31·1–44·3) | 36·5 (30·2–43·5) |  |
|  | Belgium | 76·0 (68·5–84·3) | 78·9 (71·1–86·6) | 91·8 (86·7–95·6) | 90·3 (85·2–95·2) | 37·3 (30·9–44·0) | 36·1 (30·0–42·8) |  |
|  | Cyprus | 75·1 (66·3–83·2) | 78·8 (71·7–86·3) | 91·2 (86·2–95·3) | 89·9 (84·3–94·8) | 36·7 (30·4–43·5) | 35·2 (29·0–41·8) |  |
|  | Denmark | 76·6 (68·9–84·4) | 78·8 (70·3–86·3) | 92·0 (86·8–95·8) | 90·3 (84·2–94·9) | 37·6 (31·0–44·6) | 36·4 (30·1–43·3) |  |
|  | Finland | 85·0 (77·9–91·1) | 88·4 (81·8–93·3) | 95·5 (91·9–98·0) | 95·3 (91·6–97·9) | 37·9 (31·6–44·9) | 37·1 (30·8–43·8) |  |
|  | France | 71·0 (63·2–79·8) | 73·4 (65·7–80·9) | 90·5 (85·4–94·9) | 88·2 (82·0–93·2) | 37·3 (31·0–44·1) | 36·2 (30·2–43·1) |  |
|  | Germany | 75·8 (67·8–84·4) | 78·6 (71·0–85·7) | 90·6 (85·1–95·2) | 88·9 (82·3–93·7) | 38·0 (31·8–45·4) | 36·9 (30·8–44·0) |  |
|  | Greece | 74·0 (66·4–82·2) | 77·2 (69·8–84·2) | 88·0 (81·7–93·4) | 86·1 (79·4–91·7) | 38·5 (32·0–45·4) | 37·0 (31·1–44·0) |  |
|  | Iceland | 77·2 (69·4–85·0) | 80·2 (72·0–87·1) | 93·2 (89·0–96·5) | 91·9 (87·0–95·8) | 37·7 (31·4–44·5) | 36·2 (30·3–43·1) |  |
|  | Ireland | 77·8 (70·2–85·8) | 80·3 (72·6–87·0) | 93·2 (89·2–96·6) | 92·0 (87·3–95·9) | 37·7 (31·7–44·9) | 36·0 (30·0–42·1) |  |
|  | Israel | 74·5 (66·6–83·1) | 77·8 (70·3–85·0) | 90·8 (85·5–95·3) | 89·0 (82·9–94·0) | 37·2 (31·1–43·8) | 36·9 (30·7–44·5) |  |
|  | Italy | 78·5 (69·8–86·2) | 80·5 (73·1–87·6) | 93·6 (89·4–96·8) | 91·7 (86·7–95·5) | 37·4 (31·4–44·5) | 36·6 (30·6–43·3) |  |
|  | Luxembourg | 73·6 (65·7–82·3) | 77·4 (69·6–84·3) | 91·4 (86·1–95·5) | 90·2 (85·3–94·6) | 36·8 (30·2–43·7) | 35·4 (29·7–42·0) |  |
|  | Malta | 75·5 (66·3–83·9) | 78·9 (70·6–86·2) | 91·1 (86·1–95·2) | 89·5 (83·1–94·6) | 37·6 (31·0–44·4) | 37·0 (30·7–43·9) |  |
|  | Monaco | 76·8 (68·4–85·1) | 79·1 (71·4–85·8) | 90·5 (84·5–95·3) | 88·7 (82·4–93·7) | 39·1 (32·7–46·2) | 37·5 (31·3–43·8) |  |
|  | Netherlands | 78·5 (69·8–86·2) | 80·8 (72·7–88·4) | 93·4 (88·7–96·8) | 92·1 (86·4–96·2) | 37·7 (31·5–44·8) | 36·6 (30·3–43·6) |  |
|  | Norway | 76·7 (67·6–84·5) | 80·5 (72·3–87·7) | 97·5 (95·6–98·8) | 96·8 (94·2–98·4) | 38·1 (32·3–45·1) | 37·9 (32·0–44·6) |  |
|  | Portugal | 87·1 (80·9–92·5) | 89·4 (83·9–94·3) | 95·7 (92·4–98·2) | 95·1 (91·1–97·8) | 38·0 (31·7–44·7) | 37·1 (30·8–43·9) |  |
|  | San Marino | 79·8 (71·7–87·9) | 82·2 (74·8–89·3) | 93·7 (89·4–97·0) | 92·6 (87·7–96·3) | 38·4 (32·4–45·5) | 36·9 (31·0–43·2) |  |
|  | Spain | 81·4 (76·5–86·1) | 83·7 (78·6–88·0) | 70·9 (62·0–80·7) | 72·1 (63·5–80·6) | 38·3 (32·0–45·0) | 37·2 (31·4–44·3) |  |
|  | Sweden | 78·8 (70·6–86·0) | 81·5 (74·2–88·0) | 94·4 (90·1–97·2) | 93·2 (88·9–96·8) | 37·5 (31·5–44·2) | 36·3 (30·4–43·0) |  |
|  | Switzerland | 69·8 (61·4–78·5) | 69·8 (62·5–76·8) | 94·4 (90·8–97·1) | 82·6 (75·6–88·6) | 36·6 (30·5–43·6) | 47·1 (40·5–53·8) |  |
|  | UK | 73·3 (65·6–80·6) | 75·0 (67·7–81·3) | 88·8 (83·4–93·6) | 88·3 (82·9–93·0) | 38·8 (32·8–45·8) | 33·9 (28·2–40·2) |  |
|  | **Latin America and Caribbean** | **61·3 (54·2–68·9)** | **65·5 (57·4–73·8)** | **89·2 (84·5–93·4)** | **93·3 (89·8–95·9)** | **39·8 (33·5–47·0)** | **43·5 (37·3–50·4)** |  |
|  | Andean Latin America | 61·5 (55·3–68·0) | 53·4 (47·6–59·3) | 82·9 (77·1–88·8) | 81·8 (76·3–86·9) | 42·5 (36·4–49·3) | 43·4 (37·4–50·0) |  |
|  | Bolivia | 54·3 (47·9–61·7) | 46·5 (40·5–52·5) | 74·5 (66·8–82·2) | 74·2 (65·9–81·0) | 40·7 (34·0–47·9) | 41·0 (34·7–48·6) |  |
|  | Ecuador | 61·3 (54·4–68·1) | 62·1 (55·4–69·9) | 91·0 (87·5–94·1) | 93·6 (90·6–95·9) | 43·1 (37·3–49·6) | 45·9 (39·5–52·2) |  |
|  | Peru | 64·6 (57·7–71·4) | 49·5 (43·4–55·4) | 79·6 (72·6–86·6) | 71·3 (63·6–78·5) | 42·6 (36·1–49·9) | 41·4 (35·1–48·3) |  |
|  | Caribbean | 56·1 (49·7–63·3) | 69·9 (62·6–76·8) | 85·7 (79·9–90·8) | 94·7 (91·9–96·8) | 50·9 (44·1–58·5) | 35·2 (29·8–42·1) |  |
|  | Antigua and Barbuda | 58·4 (50·7–67·8) | 75·3 (66·7–84·3) | 88·2 (81·8–93·5) | 96·4 (93·8–98·2) | 50·9 (43·5–58·8) | 35·0 (29·1–41·4) |  |
|  | The Bahamas | 52·4 (45·8–61·1) | 69·2 (59·7–77·5) | 81·6 (74·8–88·2) | 94·0 (90·1–96·7) | 50·5 (43·2–59·0) | 34·5 (28·0–41·6) |  |
|  | Barbados | 58·0 (50·1–66·0) | 79·9 (71·3–88·7) | 84·3 (76·6–90·2) | 96·8 (94·3–98·6) | 56·3 (48·9–64·9) | 37·5 (31·7–44·5) |  |
|  | Belize | 51·7 (45·7–59·4) | 67·2 (58·9–75·6) | 78·8 (71·2–85·5) | 92·7 (88·5–96·1) | 51·4 (44·3–58·5) | 34·9 (28·3–41·9) |  |
|  | Bermuda | 74·5 (65·5–84·1) | 85·6 (79·1–90·4) | 96·2 (93·2–98·2) | 98·8 (97·9–99·4) | 53·3 (46·6–61·3) | 40·5 (35·3–46·5) |  |
|  | Cuba | 65·0 (57·5–74·5) | 81·1 (73·5–87·8) | 93·4 (89·6–96·6) | 98·1 (96·4–99·1) | 51·2 (44·2–59·1) | 36·1 (30·6–42·5) |  |
|  | Dominica | 53·9 (47·2–61·2) | 68·6 (60·1–77·6) | 76·0 (68·7–83·9) | 91·4 (86·2–95·2) | 52·0 (45·3–59·6) | 36·2 (30·1–43·4) |  |
|  | Dominican Republic | 49·2 (43·7–56·4) | 65·3 (57·4–74·4) | 79·5 (72·6–85·8) | 93·0 (89·1–96·0) | 49·0 (41·5–56·4) | 33·2 (27·2–39·5) |  |
|  | Grenada | 55·7 (48·5–64·2) | 72·3 (63·9–80·9) | 82·7 (75·5–89·0) | 94·4 (90·3–97·1) | 51·1 (43·6–58·6) | 35·2 (28·7–42·3) |  |
|  | Guyana | 61·3 (54·9–69·0) | 76·6 (70·1–82·7) | 85·4 (79·1–91·2) | 95·4 (92·5–97·5) | 45·5 (39·2–52·9) | 33·8 (28·7–40·0) |  |
|  | Haiti | 40·6 (36·0–45·7) | 51·6 (44·8–59·1) | 67·4 (59·0–75·2) | 86·1 (80·4–91·2) | 45·0 (37·8–52·5) | 29·0 (24·2–35·4) |  |
|  | Jamaica | 55·1 (47·6–63·0) | 71·4 (63·4–79·7) | 85·2 (78·5–90·8) | 95·3 (92·2–97·7) | 50·9 (44·0–58·7) | 35·0 (28·9–41·1) |  |
|  | Puerto Rico | 70·8 (61·9–79·7) | 84·5 (77·0–90·6) | 93·9 (89·7–96·8) | 98·1 (96·6–99·2) | 54·2 (47·9–61·6) | 40·0 (34·8–46·2) |  |
|  | Saint Kitts and Nevis | 54·7 (47·6–63·1) | 70·5 (61·4–79·3) | 80·1 (72·1–87·4) | 93·4 (89·1–96·5) | 50·7 (43·1–58·7) | 34·7 (28·6–41·6) |  |
|  | Saint Lucia | 45·9 (40·9–51·3) | 58·4 (52·9–64·9) | 78·7 (72·2–84·4) | 90·2 (86·2–93·3) | 50·4 (43·2–57·3) | 35·3 (30·1–41·4) |  |
|  | Saint Vincent and the Grenadines | 55·7 (49·0–64·0) | 71·9 (62·8–80·6) | 84·0 (77·0–90·0) | 94·8 (90·9–97·3) | 50·3 (42·9–57·8) | 34·2 (28·2–41·3) |  |
|  | Suriname | 49·5 (43·5–56·4) | 65·9 (57·2–75·3) | 78·2 (69·5–85·1) | 92·5 (88·0–95·9) | 48·3 (40·9–55·5) | 32·6 (26·7–39·3) |  |
|  | Trinidad and Tobago | 57·2 (49·6–66·3) | 74·4 (65·0–82·5) | 83·7 (76·5–90·1) | 94·9 (91·3–97·3) | 51·5 (43·7–59·8) | 35·7 (29·2–43·4) |  |
|  | Virgin Islands | 64·0 (55·3–72·7) | 80·6 (73·0–87·4) | 89·3 (83·2–94·0) | 96·8 (94·2–98·5) | 53·9 (47·3–61·6) | 38·6 (32·6–45·3) |  |
|  | Central Latin America | 60·4 (53·2–68·6) | 66·0 (57·8–74·5) | 91·2 (86·9–94·6) | 93·4 (89·9–96·1) | 38·6 (32·2–45·8) | 43·5 (37·3–50·7) |  |
|  | Colombia | 62·4 (54·5–71·1) | 71·6 (63·0–81·1) | 94·9 (92·0–97·0) | 96·9 (94·7–98·4) | 47·2 (40·6–55·0) | 56·0 (49·7–62·8) |  |
|  | Costa Rica | 64·9 (56·9–72·8) | 68·6 (59·9–77·1) | 93·4 (89·6–96·4) | 95·7 (93·4–97·6) | 44·3 (37·9–52·2) | 42·2 (35·6–49·0) |  |
|  | El Salvador | 52·7 (45·2–60·5) | 60·6 (53·1–70·7) | 88·6 (83·7–92·6) | 92·0 (88·0–95·4) | 41·7 (35·6–49·2) | 45·0 (38·3–52·2) |  |
|  | Guatemala | 44·0 (40·1–47·7) | 49·9 (46·0–54·0) | 80·5 (75·3–84·7) | 85·7 (81·7–89·3) | 40·9 (34·6–47·8) | 43·9 (37·9–51·6) |  |
|  | Honduras | 44·1 (38·3–50·8) | 48·9 (42·4–56·4) | 81·9 (75·6–86·8) | 86·3 (81·0–91·0) | 39·1 (33·3–46·3) | 41·5 (35·1–48·1) |  |
|  | Mexico | 62·7 (55·4–71·7) | 67·7 (59·0–76·9) | 91·5 (87·1–95·1) | 93·4 (89·6–96·2) | 35·2 (29·0–42·0) | 40·2 (34·1–47·0) |  |
|  | Nicaragua | 53·2 (46·6–61·3) | 60·0 (52·4–68·6) | 90·1 (85·3–93·9) | 93·1 (89·6–96·1) | 40·9 (34·4–48·6) | 43·9 (37·4–50·6) |  |
|  | Panama | 63·3 (55·1–72·3) | 70·0 (60·7–78·6) | 95·2 (92·3–97·3) | 96·7 (94·6–98·3) | 41·0 (34·5–48·7) | 44·3 (37·9–51·3) |  |
|  | Venezuela | 54·5 (46·1–64·3) | 58·7 (50·4–68·0) | 87·8 (82·6–92·9) | 91·0 (86·2–94·7) | 41·9 (35·1–49·7) | 45·3 (38·7–52·6) |  |
|  | Tropical Latin America | 64·6 (57·3–72·5) | 65·9 (56·5–75·0) | 87·6 (82·0–92·8) | 94·6 (91·5–97·0) | 38·7 (32·6–45·9) | 46·0 (39·6–53·2) |  |
|  | Brazil | 64·8 (57·5–72·7) | 66·1 (56·7–75·2) | 87·7 (82·2–92·9) | 94·7 (91·5–97·1) | 38·8 (32·6–45·9) | 46·0 (39·6–53·2) |  |
|  | Paraguay | 59·8 (52·6–68·2) | 59·8 (51·8–70·6) | 83·7 (76·6–90·0) | 92·5 (88·4–95·9) | 37·6 (30·9–45·2) | 45·3 (39·0–52·5) |  |
|  | **North Africa and Middle East** | **63·9 (57·8–70·0)** | **67·0 (61·7–72·6)** | **91·7 (88·5–94·2)** | **93·3 (90·3–95·4)** | **46·2 (40·2–52·5)** | **43·5 (37·3–49·9)** |  |
|  | Afghanistan | 39·2 (33·5–45·2) | 43·8 (37·5–50·8) | 88·7 (83·5–92·1) | 96·6 (95·0–97·8) | 58·8 (52·1–66·8) | 34·1 (28·1–41·9) |  |
|  | Algeria | 61·3 (56·2–66·1) | 68·2 (62·2–74·1) | 93·1 (90·6–95·1) | 93·1 (90·1–95·6) | 43·1 (37·6–49·2) | 40·3 (35·4–45·8) |  |
|  | Bahrain | 67·2 (57·3–76·4) | 72·5 (63·6–81·9) | 93·5 (89·4–96·8) | 95·9 (92·8–98·1) | 45·2 (37·8–53·6) | 42·0 (35·0–50·2) |  |
|  | Egypt | 62·3 (58·0–67·1) | 61·1 (56·3–67·1) | 88·6 (85·2–91·5) | 90·0 (85·9–93·2) | 45·1 (37·6–52·5) | 41·6 (34·4–49·2) |  |
|  | Iran | 67·5 (60·4–75·0) | 77·4 (70·3–84·0) | 84·0 (77·6–90·4) | 89·2 (83·1–93·8) | 43·4 (36·7–50·8) | 43·3 (36·7–50·6) |  |
|  | Iraq | 59·7 (53·8–66·6) | 61·1 (56·1–67·3) | 93·8 (91·4–96·0) | 94·5 (92·5–96·3) | 33·2 (28·0–39·3) | 40·0 (34·6–45·7) |  |
|  | Jordan | 75·7 (69·6–80·2) | 82·7 (76·9–88·4) | 95·0 (92·3–96·7) | 95·0 (91·8–97·4) | 43·8 (38·5–49·7) | 42·7 (37·4–47·7) |  |
|  | Kuwait | 74·2 (65·9–82·1) | 76·1 (69·3–83·8) | 95·0 (91·7–97·5) | 94·7 (91·7–97·2) | 33·6 (28·3–39·6) | 33·6 (28·2–39·1) |  |
|  | Lebanon | 47·4 (41·1–53·0) | 55·3 (49·2–62·6) | 92·1 (88·5–94·8) | 96·3 (94·4–97·8) | 56·3 (50·2–62·2) | 46·0 (39·3–51·6) |  |
|  | Libya | 52·1 (45·2–61·1) | 61·4 (53·8–69·2) | 85·2 (79·4–90·8) | 84·9 (79·1–91·0) | 35·3 (28·3–42·8) | 44·1 (37·3–51·8) |  |
|  | Morocco | 56·4 (51·0–62·2) | 54·4 (49·8–59·7) | 88·5 (84·9–91·6) | 89·9 (86·5–92·7) | 41·0 (35·0–47·1) | 31·1 (26·0–35·7) |  |
|  | Oman | 61·2 (53·3–70·9) | 67·5 (58·4–76·9) | 92·2 (87·5–95·9) | 95·3 (92·1–97·4) | 45·2 (38·1–53·5) | 42·5 (35·8–50·3) |  |
|  | Palestine | 68·5 (60·2–76·5) | 72·9 (64·6–82·0) | 94·1 (90·3–96·9) | 96·8 (94·5–98·5) | 32·8 (27·2–39·9) | 30·8 (25·5–36·8) |  |
|  | Qatar | 73·5 (64·6–82·4) | 81·0 (73·4–88·3) | 94·4 (89·9–97·4) | 96·8 (94·3–98·4) | 52·8 (45·1–61·5) | 52·6 (46·0–60·1) |  |
|  | Saudi Arabia | 65·8 (56·5–76·0) | 73·7 (65·1–82·5) | 97·5 (96·0–98·8) | 99·5 (99·1–99·8) | 58·9 (55·2–63·5) | 64·2 (59·6–68·6) |  |
|  | Sudan | 61·0 (56·1–66·4) | 55·7 (50·2–62·3) | 89·0 (85·7–92·5) | 92·6 (89·9–94·9) | 33·6 (28·7–38·8) | 23·9 (19·4–29·0) |  |
|  | Syria | 65·6 (57·5–74·8) | 68·9 (60·7–77·5) | 91·3 (86·8–95·4) | 94·3 (90·6–97·1) | 45·0 (37·9–52·7) | 41·5 (33·9–49·1) |  |
|  | Tunisia | 73·4 (64·2–81·6) | 77·8 (68·8–86·6) | 96·0 (93·4–98·1) | 97·5 (95·6–98·9) | 43·5 (36·7–50·9) | 39·9 (32·4–47·2) |  |
|  | Türkiye | 69·4 (60·4–78·3) | 77·7 (70·0–85·2) | 94·0 (90·3–96·7) | 96·4 (93·8–98·3) | 47·3 (40·2–54·9) | 50·0 (43·0–56·8) |  |
|  | United Arab Emirates | 75·6 (66·6–84·7) | 77·3 (68·5–87·5) | 93·6 (90·0–97·0) | 95·9 (92·7–98·2) | 58·4 (52·2–65·9) | 58·6 (51·8–65·4) |  |
|  | Yemen | 50·8 (44·4–58·0) | 56·4 (48·8–65·4) | 88·0 (83·2–92·6) | 92·1 (88·2–95·6) | 39·9 (33·2–47·0) | 36·8 (30·4–43·9) |  |
|  | **South Asia** | **38·4 (32·4–45·4)** | **49·2 (41·7–57·0)** | **94·3 (91·7–96·1)** | **96·7 (95·1–97·9)** | **33·4 (27·5–40·0)** | **34·9 (29·0–41·3)** |  |
|  | Bangladesh | 49·8 (46·1–54·2) | 52·1 (48·3–56·7) | 91·6 (89·3–93·9) | 91·9 (89·7–93·5) | 40·7 (36·4–45·6) | 39·8 (35·4–45·0) |  |
|  | Bhutan | 56·6 (50·1–64·4) | 54·0 (47·9–60·0) | 90·0 (85·5–93·8) | 93·1 (90·2–95·4) | 60·9 (54·4–67·1) | 65·3 (59·1–70·8) |  |
|  | India | 36·9 (30·5–44·7) | 50·2 (41·7–59·1) | 96·3 (94·4–97·6) | 97·9 (96·8–98·7) | 31·8 (25·9–38·4) | 34·1 (28·1–40·6) |  |
|  | Nepal | 44·7 (39·1–50·5) | 38·3 (32·9–43·6) | 79·0 (72·9–85·0) | 83·9 (78·3–88·6) | 31·8 (26·3–38·6) | 32·3 (26·5–38·5) |  |
|  | Pakistan | 40·6 (34·7–47·6) | 41·7 (35·5–49·1) | 85·0 (80·0–89·7) | 89·5 (85·5–93·0) | 40·7 (33·5–48·1) | 41·0 (34·4–48·6) |  |
|  | **Southeast Asia, east Asia, and Oceania** | **39·6 (34·2–45·8)** | **54·9 (47·7–62·4)** | **84·6 (79·3–89·2)** | **89·8 (85·5–93·6)** | **38·2 (32·2–45·0)** | **39·3 (33·5–46·5)** |  |
|  | East Asia | 37·5 (32·2–43·5) | 53·6 (46·4–61·2) | 83·7 (78·2–88·4) | 88·6 (84·0–92·8) | 34·2 (28·4–41·1) | 36·5 (30·8–43·7) |  |
|  | China | 37·3 (32·1–43·4) | 53·4 (46·2–61·1) | 83·7 (78·1–88·4) | 88·5 (83·9–92·7) | 34·1 (28·2–40·9) | 36·4 (30·7–43·6) |  |
|  | North Korea | 32·8 (28·2–38·3) | 46·6 (40·0–53·4) | 75·1 (67·3–81·7) | 81·3 (74·6–87·2) | 34·9 (28·6–42·5) | 36·6 (30·5–43·4) |  |
|  | Taiwan | 48·0 (41·2–56·2) | 65·4 (56·6–74·4) | 90·9 (86·6–94·4) | 94·1 (90·5–96·8) | 38·7 (32·8–45·7) | 40·1 (34·3–46·7) |  |
|  | Oceania | 43·6 (38·2–50·0) | 47·2 (40·9–54·7) | 79·8 (73·3–86·1) | 84·2 (78·5–89·4) | 25·1 (20·0–30·9) | 23·7 (19·1–29·5) |  |
|  | American Samoa | 51·9 (48·4–55·4) | 54·9 (51·5–58·4) | 84·6 (81·9–87·4) | 86·1 (83·4–88·8) | 37·2 (33·6–40·7) | 37·3 (33·7–40·9) |  |
|  | Cook Islands | 41·8 (36·5–47·7) | 50·7 (44·3–58·5) | 79·8 (73·1–85·4) | 89·4 (85·3–93·2) | 29·2 (24·2–36·1) | 39·6 (33·4–46·7) |  |
|  | Fiji | 60·9 (53·8–68·9) | 61·0 (52·9–69·5) | 79·5 (70·1–87·5) | 84·0 (77·2–90·3) | 30·2 (23·9–37·2) | 26·1 (20·9–32·3) |  |
|  | Guam | 57·1 (49·5–66·7) | 64·5 (56·2–73·6) | 92·1 (88·0–95·5) | 94·5 (91·1–97·1) | 26·6 (21·2–33·0) | 26·3 (21·1–32·5) |  |
|  | Kiribati | 38·7 (34·0–44·4) | 42·4 (37·4–48·0) | 65·4 (57·0–73·2) | 72·9 (65·3–79·1) | 30·9 (25·0–37·5) | 20·2 (15·8–25·4) |  |
|  | Marshall Islands | 40·5 (38·3–42·8) | 40·1 (38·0–42·5) | 65·0 (61·8–68·2) | 69·4 (66·1–72·6) | 19·7 (17·6–22·3) | 17·6 (15·6–19·9) |  |
|  | Federated States of Micronesia | 36·6 (34·5–39·2) | 38·7 (36·3–41·2) | 68·1 (64·8–72·0) | 69·3 (66·0–72·8) | 16·7 (14·5–19·4) | 18·1 (15·5–21·1) |  |
|  | Nauru | 56·6 (50·6–62·6) | 64·7 (58·7–69·7) | 72·2 (64·7–79·1) | 64·4 (56·5–72·1) | 39·2 (32·8–45·9) | 34·8 (29·0–41·7) |  |
|  | Niue | 48·5 (42·0–56·5) | 65·8 (59·0–73·6) | 81·7 (73·9–87·7) | 81·8 (74·2–88·9) | 20·8 (16·3–26·5) | 26·9 (21·7–32·8) |  |
|  | Northern Mariana Islands | 51·7 (45·0–60·8) | 56·7 (48·5–66·3) | 84·7 (77·5–90·6) | 88·9 (82·6–93·7) | 27·4 (21·5–34·3) | 26·7 (21·1–33·4) |  |
|  | Palau | 53·0 (50·1–56·2) | 53·8 (50·9–57·0) | 79·1 (75·9–82·4) | 80·8 (77·8–83·8) | 21·9 (19·4–24·8) | 23·1 (20·2–26·7) |  |
|  | Papua New Guinea | 40·8 (35·1–47·5) | 44·9 (38·5–53·5) | 84·0 (78·2–89·5) | 88·0 (82·9–92·4) | 22·4 (17·6–28·2) | 21·5 (17·0–27·4) |  |
|  | Samoa | 33·2 (28·8–37·9) | 39·8 (34·6–45·2) | 69·2 (61·7–76·0) | 80·7 (74·6–86·0) | 32·9 (26·8–40·3) | 27·3 (22·3–33·3) |  |
|  | Solomon Islands | 34·1 (30·0–39·1) | 39·6 (35·1–44·8) | 42·3 (35·1–50·2) | 49·6 (42·2–57·4) | 22·0 (17·2–28·2) | 29·7 (23·6–36·7) |  |
|  | Tokelau | 55·6 (50·2–60·6) | 63·7 (58·3–69·3) | 66·3 (59·3–73·5) | 77·5 (71·3–83·7) | 20·3 (16·4–25·3) | 14·6 (11·5–18·4) |  |
|  | Tonga | 48·0 (42·2–55·1) | 52·9 (46·0–60·4) | 74·6 (66·8–82·3) | 81·1 (74·3–87·8) | 24·5 (19·4–30·5) | 18·3 (14·3–23·4) |  |
|  | Tuvalu | 57·6 (51·2–65·0) | 58·9 (53·0–65·9) | 81·7 (74·2–88·3) | 79·5 (71·6–86·4) | 18·6 (14·3–23·9) | 33·0 (27·2–39·9) |  |
|  | Vanuatu | 22·7 (19·2–26·2) | 27·0 (22·6–32·3) | 65·0 (56·9–71·9) | 88·7 (84·2–92·5) | 41·2 (34·2–48·5) | 43·9 (36·4–52·2) |  |
|  | Southeast Asia | 45·9 (40·2–52·8) | 58·3 (51·2–66·3) | 86·8 (81·9–91·0) | 92·7 (89·4–95·6) | 47·6 (41·1–54·7) | 45·5 (39·3–52·4) |  |
|  | Cambodia | 57·8 (50·8–66·5) | 64·4 (56·9–72·9) | 88·4 (82·3–93·1) | 88·7 (83·0–94·0) | 52·4 (45·6–59·5) | 50·6 (43·3–57·9) |  |
|  | Indonesia | 42·0 (36·1–49·1) | 54·2 (46·8–63·5) | 82·3 (75·9–87·8) | 91·5 (87·0–95·2) | 45·4 (38·3–52·8) | 42·0 (35·3–49·0) |  |
|  | Laos | 42·0 (37·1–48·3) | 64·0 (58·0–70·9) | 75·4 (68·1–82·0) | 90·6 (86·4–94·3) | 51·7 (44·3–59·1) | 33·1 (27·1–39·3) |  |
|  | Malaysia | 43·4 (36·1–51·5) | 51·4 (43·3–61·1) | 96·2 (94·3–97·8) | 96·6 (94·5–98·2) | 54·1 (47·2–61·2) | 53·2 (47·0–60·1) |  |
|  | Maldives | 29·3 (25·1–34·1) | 65·0 (56·6–74·0) | 82·4 (77·1–87·2) | 96·6 (94·2–98·2) | 46·4 (39·4–54·1) | 44·3 (37·5–51·4) |  |
|  | Mauritius | 53·8 (45·9–63·4) | 66·2 (58·0–76·0) | 89·9 (84·4–94·3) | 94·5 (90·9–97·3) | 49·0 (42·1–56·6) | 46·9 (40·0–54·3) |  |
|  | Myanmar | 55·4 (49·8–62·1) | 56·9 (51·5–62·3) | 89·1 (84·5–92·8) | 89·0 (85·2–92·0) | 51·4 (45·2–58·1) | 35·9 (30·5–41·9) |  |
|  | Philippines | 40·8 (35·1–48·0) | 54·5 (47·0–63·0) | 83·8 (77·8–89·1) | 91·9 (87·5–95·4) | 44·9 (37·7–52·5) | 42·7 (36·3–49·7) |  |
|  | Seychelles | 52·4 (44·6–62·5) | 70·2 (61·4–79·6) | 93·0 (88·7–96·4) | 96·6 (94·0–98·3) | 15·4 (11·4–20·5) | 24·6 (19·5–31·3) |  |
|  | Sri Lanka | 71·7 (66·8–77·0) | 74·5 (69·1–80·9) | 86·8 (82·4–91·5) | 93·0 (89·8–95·7) | 58·1 (52·4–63·4) | 50·4 (43·9–55·7) |  |
|  | Thailand | 49·4 (41·7–58·3) | 68·5 (58·6–78·5) | 94·2 (91·5–96·5) | 98·1 (96·6–99·1) | 32·8 (26·8–39·5) | 39·8 (33·2–47·0) |  |
|  | Timor-Leste | 22·6 (18·9–26·6) | 26·8 (21·2–33·4) | 74·1 (66·1–80·6) | 87·9 (81·6–92·5) | 43·6 (34·1–52·6) | 47·4 (38·5–55·7) |  |
|  | Viet Nam | 42·5 (36·5–48·9) | 58·9 (52·9–66·1) | 91·1 (87·4–94·1) | 91·2 (87·7–95·0) | 61·0 (54·1–67·6) | 64·0 (58·1–70·1) |  |
|  | **Sub-Saharan Africa** | **32·7 (28·5–37·3)** | **35·5 (30·7–40·3)** | **76·9 (70·5–82·5)** | **84·2 (79·1–88·6)** | **48·7 (42·4–55·6)** | **48·1 (41·7–55·1)** |  |
|  | Central sub-Saharan Africa | 18·0 (15·5–20·8) | 14·8 (12·6–17·1) | 65·3 (58·5–71·6) | 73·4 (66·8–79·4) | 50·4 (43·7–57·7) | 51·0 (43·8–58·6) |  |
|  | Angola | 19·4 (16·9–21·8) | 17·3 (14·9–19·7) | 52·1 (45·7–59·3) | 73·0 (66·3–78·7) | 42·4 (35·0–50·5) | 70·8 (64·2–76·8) |  |
|  | Central African Republic | 15·3 (13·3–17·7) | 16·5 (14·3–18·6) | 74·4 (67·8–79·9) | 79·9 (74·6–84·1) | 60·0 (53·0–66·9) | 46·9 (40·3–53·5) |  |
|  | Congo (Brazzaville) | 18·2 (15·7–20·6) | 16·2 (13·9–18·6) | 66·8 (59·7–73·9) | 75·9 (69·6–81·1) | 53·1 (46·4–59·9) | 54·8 (48·2–61·6) |  |
|  | DR Congo | 17·5 (14·9–20·2) | 13·2 (11·1–15·6) | 71·3 (64·1–77·7) | 72·6 (64·1–79·7) | 51·3 (44·6–58·6) | 42·2 (35·0–49·8) |  |
|  | Equatorial Guinea | 16·0 (13·6–18·3) | 17·0 (14·6–19·8) | 73·3 (67·2–79·8) | 81·5 (75·9–86·2) | 53·5 (47·0–59·7) | 55·5 (48·8–62·0) |  |
|  | Gabon | 21·2 (18·2–24·4) | 18·6 (15·9–21·5) | 67·2 (59·6–74·0) | 75·9 (69·1–81·2) | 56·7 (49·6–63·8) | 57·6 (51·2–64·1) |  |
|  | Eastern sub-Saharan Africa | 34·7 (30·1–39·0) | 40·5 (35·5–45·6) | 76·2 (69·7–81·7) | 82·6 (77·3–87·3) | 55·5 (48·9–62·1) | 54·2 (47·5–60·5) |  |
|  | Burundi | 33·8 (29·5–38·8) | 39·6 (33·8–46·4) | 79·0 (72·1–84·6) | 86·3 (81·6–90·7) | 53·3 (47·4–60·0) | 52·7 (46·2–58·9) |  |
|  | Comoros | 52·8 (45·9–59·5) | 53·6 (46·2–61·1) | 86·3 (80·2–90·9) | 94·2 (91·0–96·6) | 45·7 (38·8–52·6) | 43·8 (37·5–50·2) |  |
|  | Djibouti | 38·3 (33·6–44·5) | 46·1 (39·2–54·0) | 82·6 (76·8–87·7) | 89·1 (84·9–92·8) | 54·6 (47·9–61·6) | 53·9 (47·2–60·4) |  |
|  | Eritrea | 58·3 (51·4–66·1) | 59·5 (51·6–67·9) | 91·2 (85·9–95·1) | 94·3 (90·4–96·9) | 60·5 (54·4–66·4) | 45·5 (39·2–51·9) |  |
|  | Ethiopia | 16·8 (14·2–19·0) | 28·0 (24·0–31·5) | 55·5 (47·5–62·0) | 78·6 (72·5–83·5) | 55·4 (48·6–61·4) | 54·0 (47·3–60·2) |  |
|  | Kenya | 47·8 (41·9–53·9) | 44·9 (39·1–51·4) | 82·8 (76·8–87·9) | 87·9 (83·3–91·9) | 79·7 (75·3–84·0) | 39·9 (34·0–46·5) |  |
|  | Madagascar | 34·6 (29·7–40·2) | 41·1 (35·6–46·7) | 81·3 (75·7–86·4) | 88·1 (83·9–91·7) | 53·0 (46·3–60·3) | 52·7 (46·0–59·2) |  |
|  | Malawi | 47·0 (41·1–52·5) | 46·3 (40·8–52·3) | 73·0 (65·3–80·1) | 82·1 (76·3–87·5) | 63·6 (54·1–71·1) | 59·7 (51·8–66·7) |  |
|  | Mozambique | 32·1 (25·9–39·0) | 53·9 (46·4–62·6) | 85·9 (78·9–91·2) | 88·4 (82·4–93·0) | 52·9 (42·0–62·4) | 72·6 (59·8–82·0) |  |
|  | Rwanda | 30·0 (25·4–34·2) | 37·4 (32·6–42·4) | 75·7 (68·2–82·0) | 89·5 (85·3–92·7) | 65·3 (54·1–74·6) | 67·4 (57·4–76·0) |  |
|  | Somalia | 32·8 (28·2–36·8) | 39·8 (34·9–44·7) | 65·7 (58·1–72·9) | 76·0 (69·1–82·4) | 54·9 (48·2–61·7) | 54·7 (48·2–61·7) |  |
|  | South Sudan | 34·5 (30·0–39·4) | 39·2 (33·9–45·6) | 74·7 (67·1–80·9) | 82·7 (76·8–88·1) | 53·9 (47·3–61·2) | 53·1 (46·3–59·7) |  |
|  | Uganda | 40·5 (35·0–47·1) | 51·8 (45·7–56·9) | 92·7 (89·5–95·3) | 80·6 (74·7–86·4) | 70·4 (65·0–75·5) | 64·5 (58·7–69·9) |  |
|  | Tanzania | 51·3 (45·0–57·5) | 45·4 (39·6–51·1) | 75·5 (68·3–82·4) | 78·3 (71·4–84·3) | 30·3 (24·9–36·2) | 57·0 (50·3–63·8) |  |
|  | Zambia | 19·3 (16·7–22·0) | 30·5 (26·2–35·1) | 58·7 (51·0–65·6) | 85·2 (80·6–88·9) | 32·5 (26·7–39·3) | 32·4 (27·2–38·0) |  |
|  | Southern sub-Saharan Africa | 50·0 (43·1–57·1) | 54·7 (47·0–62·7) | 83·3 (77·4–88·7) | 92·7 (89·0–95·8) | 37·7 (31·5–44·5) | 36·0 (29·9–43·2) |  |
|  | Botswana | 43·5 (37·6–49·8) | 59·7 (53·1–67·7) | 81·3 (74·5–87·0) | 97·3 (95·9–98·5) | 51·9 (45·5–58·6) | 55·0 (48·3–61·2) |  |
|  | Eswatini | 52·1 (45·1–59·9) | 42·6 (36·5–49·1) | 94·3 (91·5–96·6) | 94·0 (91·2–95·9) | 44·9 (38·7–51·3) | 55·0 (48·4–61·1) |  |
|  | Lesotho | 41·8 (36·0–48·3) | 69·5 (61·6–77·1) | 82·7 (76·2–88·0) | 96·6 (94·3–98·3) | 42·0 (35·9–49·1) | 47·2 (39·8–54·7) |  |
|  | Namibia | 47·4 (40·5–54·7) | 60·3 (52·2–69·6) | 91·4 (87·5–94·6) | 97·3 (95·6–98·5) | 37·3 (31·2–44·0) | 38·0 (31·5–44·5) |  |
|  | South Africa | 51·0 (44·0–58·3) | 54·6 (46·7–63·0) | 82·7 (76·4–88·4) | 92·0 (88·0–95·5) | 36·7 (30·8–43·7) | 34·0 (28·1–41·0) |  |
|  | Zimbabwe | 43·4 (37·1–49·8) | 52·8 (45·2–60·8) | 87·0 (82·2–91·0) | 95·4 (92·6–97·3) | 41·4 (35·3–48·0) | 44·8 (37·8–52·1) |  |
|  | Western sub-Saharan Africa | 31·3 (27·3–35·6) | 32·7 (28·2–37·3) | 77·0 (70·6–82·6) | 82·8 (77·3–87·4) | 48·4 (42·0–55·1) | 48·6 (42·2–55·6) |  |
|  | Benin | 31·3 (27·2–35·4) | 20·9 (17·9–24·0) | 85·0 (79·9–89·1) | 86·0 (82·1–89·1) | 58·6 (52·3–64·4) | 44·3 (38·6–50·9) |  |
|  | Burkina Faso | 30·0 (26·2–33·9) | 31·1 (26·7–35·4) | 77·0 (70·1–82·7) | 82·6 (76·9–87·4) | 45·8 (39·2–52·5) | 49·0 (42·0–55·7) |  |
|  | Cabo Verde | 63·0 (55·6–70·8) | 53·1 (48·1–58·4) | 96·0 (94·1–97·7) | 85·3 (80·9–89·6) | 39·1 (33·4–45·4) | 41·0 (35·2–47·6) |  |
|  | Cameroon | 47·3 (40·7–54·4) | 35·5 (30·7–40·8) | 91·6 (88·2–95·0) | 82·2 (77·0–87·1) | 57·0 (50·8–63·5) | 52·6 (46·0–59·5) |  |
|  | Chad | 26·3 (22·7–30·3) | 25·6 (22·1–29·7) | 72·1 (65·0–78·6) | 77·7 (71·7–83·1) | 43·8 (37·1–50·6) | 47·2 (41·0–54·3) |  |
|  | Côte d'Ivoire | 27·2 (23·6–31·4) | 27·9 (23·9–32·1) | 73·5 (66·5–79·8) | 79·3 (73·0–84·8) | 45·0 (38·7–52·1) | 49·9 (43·9–56·4) |  |
|  | The Gambia | 28·0 (24·2–31·9) | 27·9 (24·1–31·9) | 73·2 (67·4–79·2) | 79·0 (73·0–84·5) | 46·2 (39·4–52·6) | 50·1 (43·5–56·5) |  |
|  | Ghana | 43·4 (37·7–49·3) | 57·2 (50·8–63·7) | 72·3 (64·1–79·9) | 83·9 (77·5–89·6) | 51·5 (44·8–58·4) | 39·8 (33·6–46·1) |  |
|  | Guinea | 28·7 (24·7–32·4) | 40·1 (34·8–46·2) | 59·2 (50·8–66·6) | 84·7 (79·2–89·8) | 26·1 (19·1–35·5) | 68·5 (60·3–75·6) |  |
|  | Guinea-Bissau | 25·4 (22·2–29·2) | 26·6 (23·1–29·9) | 65·2 (58·1–72·2) | 71·8 (65·1–78·0) | 44·2 (37·5–50·9) | 48·6 (41·9–55·2) |  |
|  | Liberia | 33·3 (28·5–38·5) | 30·3 (26·0–35·3) | 82·0 (75·6–87·4) | 85·8 (80·0–90·3) | 44·0 (36·2–52·3) | 57·6 (48·5–65·5) |  |
|  | Mali | 23·3 (19·8–27·0) | 23·2 (19·8–26·8) | 66·7 (59·0–73·7) | 82·9 (77·7–87·4) | 39·2 (30·2–47·9) | 39·3 (32·8–46·0) |  |
|  | Mauritania | 28·1 (23·2–33·9) | 26·6 (21·9–32·1) | 75·0 (67·0–82·3) | 73·7 (64·7–82·1) | 48·1 (39·3–56·0) | 57·0 (48·7–64·9) |  |
|  | Niger | 10·4 (8·5–12·5) | 11·0 (8·7–13·7) | 60·6 (49·7–70·7) | 75·7 (67·4–83·9) | 45·5 (37·2–54·1) | 42·6 (34·2–51·1) |  |
|  | Nigeria | 31·0 (27·0–35·1) | 33·3 (28·7–38·5) | 78·3 (72·1–83·8) | 84·2 (79·1–88·8) | 46·9 (40·3–53·5) | 50·5 (44·0–57·4) |  |
|  | São Tomé and Príncipe | 28·8 (25·2–32·9) | 40·6 (35·2–46·5) | 59·6 (52·0–67·3) | 87·1 (82·8–91·0) | 48·7 (38·9–59·3) | 51·3 (44·8–57·9) |  |
|  | Senegal | 28·7 (24·9–32·6) | 30·4 (26·4–35·0) | 77·2 (70·9–82·9) | 82·5 (76·6–87·3) | 46·1 (39·8–52·6) | 49·7 (43·2–56·3) |  |
|  | Sierra Leone | 27·7 (24·4–31·4) | 28·0 (24·3–32·2) | 75·2 (68·2–80·9) | 80·6 (74·4–85·3) | 44·7 (38·2–51·4) | 48·8 (42·5–55·0) |  |
|  | Togo | 28·9 (25·3–33·1) | 31·7 (27·4–36·4) | 74·4 (68·1–80·5) | 80·6 (74·3–85·6) | 45·0 (38·6–52·2) | 49·8 (43·1–56·5) |  |
|  |  |  |  |  |  |  |  |  |

## Table S16. Diagnosed proportion among people living with diabetes, treated proportion among people with diagnosed diabetes, and optimal glycaemic management proportion among people with treated diabetes in 2023, and corresponding absolute change between 2000 and 2023, for both sexes and ages 15 and older globally, and by Global Burden of Disease super-regions, regions, and countries and territories – sensitivity analysis

|  | **Location** | **Diagnosed, 2023 (%)** | **Change in Diagnosed, 2000-2023 (%)** | **Treated, 2023 (%)** | **Change in Treated, 2000-2023 (%)** | **Optimal Glycaemic Management, 2023 (%)** | **Change in Optimal Glycaemic Management, 2000-2023 (%)** |  |
| --- | --- | --- | --- | --- | --- | --- | --- | --- |
|  | **Global** | **55·8 (49·3–62·3)** | **8·3 (6·6–10·0)** | **91·4 (88·0–94·2)** | **7·2 (5·7–8·8)** | **59·2 (52·3–64·8)** | **1·4 (0·8–2·0)** |  |
|  | **Central Europe, eastern Europe, and central Asia** | **67·7 (59·6–75·7)** | **5·3 (3·7–6·8)** | **92·1 (87·6–95·6)** | **2·8 (1·6–4·0)** | **66·0 (58·9–71·5)** | **2·3 (1·7–2·9)** |  |
|  | Central Asia | 54·1 (48·2–60·6) | 7·2 (5·1–9·3) | 88·4 (83·0–92·7) | 8·0 (5·4–10·8) | 55·0 (47·7–61·2) | 1·7 (0·2–3·2) |  |
|  | Armenia | 63·7 (56·1–71·5) | 8·9 (5·3–12·5) | 91·3 (85·8–95·2) | 7·7 (4·1–11·5) | 63·9 (56·9–69·4) | 2·6 (0·3–4·8) |  |
|  | Azerbaijan | 65·6 (58·8–72·0) | 11·4 (6·8–15·4) | 93·9 (90·6–96·5) | 5·9 (2·9–9·4) | 48·6 (41·5–54·8) | 3·4 (0·9–6·5) |  |
|  | Georgia | 73·3 (67·2–79·2) | 5·9 (1·6–10·8) | 90·9 (86·4–94·4) | 3·9 (0·7–7·9) | 57·4 (51·1–63·0) | 1·5 (-0·8–4·3) |  |
|  | Kazakhstan | 59·3 (52·3–67·2) | 12·1 (7·7–16·3) | 91·6 (86·3–95·4) | 13·2 (8·8–17·5) | 69·2 (61·4–75·2) | 3·0 (1·0–5·0) |  |
|  | Kyrgyzstan | 56·5 (50·0–64·0) | 11·3 (7·5–15·7) | 93·2 (89·6–96·0) | 7·5 (4·8–11·0) | 32·4 (25·9–37·8) | 2·2 (0·1–4·4) |  |
|  | Mongolia | 44·9 (39·6–50·7) | 6·6 (4·0–9·5) | 78·2 (72·4–84·3) | 13·0 (9·8–16·6) | 57·5 (51·0–63·4) | 1·4 (-1·2–3·9) |  |
|  | Tajikistan | 38·7 (34·1–43·5) | 2·4 (0·3–4·6) | 64·5 (55·9–72·5) | 9·4 (6·0–13·4) | 41·0 (34·1–47·8) | 2·1 (-0·5–4·5) |  |
|  | Turkmenistan | 28·8 (24·9–33·0) | 2·6 (1·2–4·5) | 81·7 (75·6–86·7) | 7·5 (4·6–10·5) | 39·8 (33·2–46·5) | 1·9 (-0·1–4·0) |  |
|  | Uzbekistan | 49·9 (43·2–58·5) | 8·1 (5·6–11·9) | 87·0 (81·1–92·2) | 7·0 (5·1–9·3) | 50·6 (42·8–57·9) | 2·5 (0·7–4·3) |  |
|  | Central Europe | 71·8 (63·0–80·5) | 2·7 (0·9–4·5) | 91·9 (86·6–95·6) | 1·5 (0·7–2·5) | 67·2 (59·8–72·9) | 0·9 (-0·1–2·0) |  |
|  | Albania | 67·8 (58·2–78·8) | 7·1 (3·5–11·0) | 90·5 (84·6–95·2) | 4·2 (2·2–6·0) | 61·1 (52·8–67·9) | 1·8 (-0·2–3·7) |  |
|  | Bosnia and Herzegovina | 66·6 (57·2–77·6) | 4·5 (1·3–7·7) | 88·7 (81·9–94·2) | 3·9 (1·8–6·4) | 61·2 (53·0–67·8) | 1·9 (0·1–3·7) |  |
|  | Bulgaria | 64·1 (55·2–74·3) | 1·4 (-0·9–3·6) | 85·4 (77·4–91·7) | 1·7 (0·1–3·3) | 61·6 (53·7–68·5) | 1·2 (-0·1–2·7) |  |
|  | Croatia | 73·9 (63·3–84·2) | 4·3 (0·8–7·9) | 93·4 (88·1–96·9) | 2·3 (0·6–4·3) | 63·3 (55·3–69·6) | 2·2 (0·4–4·1) |  |
|  | Czechia | 62·0 (53·5–72·0) | 4·5 (0·6–10·7) | 83·6 (75·3–90·2) | 5·1 (1·7–8·9) | 49·2 (41·3–56·5) | 3·1 (0·3–5·2) |  |
|  | Hungary | 68·4 (59·4–78·4) | 2·1 (-0·8–4·6) | 89·3 (83·1–94·1) | 0·9 (-0·5–2·5) | 63·4 (56·1–70·3) | 1·9 (0·3–3·6) |  |
|  | Montenegro | 70·2 (60·3–80·1) | 3·6 (0·4–6·9) | 91·1 (85·1–95·6) | 1·7 (-0·2–3·2) | 63·6 (55·6–70·0) | 2·3 (0·5–4·1) |  |
|  | North Macedonia | 65·8 (57·1–76·2) | 4·9 (1·8–8·1) | 87·5 (80·3–93·2) | 4·5 (2·6–6·9) | 62·0 (54·3–68·7) | 2·0 (0·3–3·6) |  |
|  | Poland | 78·6 (68·7–87·0) | 3·1 (0·3–6·2) | 95·9 (92·5–98·2) | 0·9 (0·2–2·0) | 73·3 (67·2–78·2) | 2·5 (1·3–3·8) |  |
|  | Romania | 65·8 (56·5–75·9) | 4·4 (2·2–6·6) | 87·9 (81·1–93·3) | 3·6 (1·9–5·5) | 61·4 (53·8–68·1) | 2·3 (0·5–4·5) |  |
|  | Serbia | 65·6 (56·6–76·0) | 4·1 (1·4–7·0) | 86·6 (79·5–92·8) | 4·7 (2·4–7·1) | 62·6 (54·5–69·5) | 2·5 (0·9–4·1) |  |
|  | Slovakia | 67·8 (58·2–77·9) | 4·0 (1·4–6·8) | 89·8 (83·7–94·7) | 3·2 (1·3–5·0) | 62·1 (54·4–68·4) | 1·7 (0·4–3·1) |  |
|  | Slovenia | 79·0 (69·5–87·3) | 6·9 (1·5–11·4) | 96·2 (92·9–98·3) | 3·0 (0·8–5·4) | 63·5 (56·3–69·6) | 2·3 (0·4–4·1) |  |
|  | Eastern Europe | 69·5 (60·9–77·5) | 9·0 (6·6–11·0) | 93·3 (89·3–96·3) | 3·0 (1·7–4·6) | 68·2 (61·2–73·5) | 5·5 (4·7–6·4) |  |
|  | Belarus | 77·3 (72·7–81·5) | 9·3 (3·2–14·2) | 88·3 (84·2–91·9) | 13·6 (5·9–20·8) | 71·0 (65·5–75·7) | 3·4 (0·4–6·7) |  |
|  | Estonia | 72·9 (64·0–82·3) | 10·0 (5·1–13·5) | 94·1 (89·9–97·2) | 6·5 (3·5–9·8) | 65·2 (58·5–70·8) | 3·3 (1·6–5·1) |  |
|  | Latvia | 67·0 (58·7–76·6) | 5·0 (1·7–8·3) | 89·5 (83·9–94·5) | 3·4 (1·4–5·5) | 64·7 (57·2–70·5) | 2·9 (1·3–4·8) |  |
|  | Lithuania | 68·8 (59·7–78·4) | 7·9 (2·7–15·3) | 93·3 (89·0–96·6) | 3·1 (0·7–5·6) | 56·8 (48·9–63·0) | 3·3 (0·4–6·0) |  |
|  | Moldova | 57·9 (52·1–64·2) | 3·7 (-0·3–7·1) | 79·1 (73·2–84·8) | 5·2 (1·0–9·0) | 58·9 (52·1–64·7) | 3·5 (1·4–5·8) |  |
|  | Russia | 71·5 (62·1–80·8) | 10·9 (8·0–13·3) | 96·2 (93·5–98·1) | 2·4 (1·4–3·6) | 69·2 (62·3–74·5) | 7·5 (6·5–8·5) |  |
|  | Ukraine | 58·4 (50·8–65·9) | 0·0 (-2·1–2·0) | 82·2 (74·6–88·4) | -4·7 (-6·7–-2·8) | 64·3 (56·8–70·4) | -0·3 (-1·7–1·0) |  |
|  | **High income** | **79·5 (72·6–85·8)** | **6·3 (4·9–7·6)** | **91·5 (86·5–95·2)** | **2·8 (2·0–3·8)** | **67·0 (61·1–71·8)** | **3·4 (2·4–4·1)** |  |
|  | Australasia | 70·6 (63·1–78·6) | 6·3 (2·2–11·8) | 86·1 (79·4–91·7) | 5·6 (2·1–9·0) | 56·1 (49·0–62·4) | 5·1 (2·9–6·8) |  |
|  | Australia | 70·7 (63·0–78·9) | 6·7 (2·1–13·2) | 86·4 (79·6–91·9) | 5·5 (1·6–9·6) | 56·6 (49·5–62·8) | 5·5 (2·9–7·6) |  |
|  | New Zealand | 70·0 (62·9–77·4) | 4·9 (2·5–7·3) | 85·0 (77·8–91·3) | 6·3 (4·0–8·6) | 54·5 (47·1–61·3) | 4·1 (2·8–5·5) |  |
|  | High-income Asia Pacific | 75·4 (68·5–81·9) | 11·1 (7·7–14·2) | 96·5 (94·6–98·0) | 4·2 (2·5–6·3) | 73·7 (69·8–77·4) | 3·6 (1·9–5·4) |  |
|  | Brunei | 73·8 (66·9–79·7) | 8·4 (3·9–14·7) | 90·8 (86·3–94·2) | 4·6 (1·2–8·7) | 57·5 (51·1–63·3) | 2·4 (-0·5–5·3) |  |
|  | Japan | 74·7 (65·9–83·4) | 8·5 (4·5–12·3) | 96·4 (93·9–98·2) | 2·4 (1·1–4·1) | 73·3 (67·9–78·0) | 3·1 (1·6–4·7) |  |
|  | South Korea | 77·1 (74·9–79·1) | 20·7 (18·0–23·1) | 96·7 (95·8–97·4) | 14·0 (11·9–16·2) | 76·5 (74·4–78·6) | 4·8 (3·0–6·9) |  |
|  | Singapore | 74·5 (67·2–82·1) | 17·9 (11·9–24·1) | 96·1 (93·7–97·9) | 7·2 (5·5–8·9) | 53·9 (47·3–59·5) | 3·8 (0·4–6·8) |  |
|  | High-income North America | 82·9 (75·5–89·3) | 4·4 (2·5–6·0) | 92·4 (87·2–96·1) | -0·2 (-1·4–0·9) | 68·8 (62·5–73·8) | 4·0 (2·7–5·3) |  |
|  | Canada | 86·0 (79·4–91·8) | 2·6 (-1·3–6·6) | 96·4 (93·5–98·3) | 0·5 (-0·7–1·7) | 65·5 (59·4–70·8) | 1·1 (-0·4–2·6) |  |
|  | Greenland | 72·5 (64·9–80·7) | 6·3 (2·3–11·1) | 82·7 (74·3–90·4) | 9·9 (6·4–14·0) | 62·9 (54·2–69·4) | 2·6 (0·4–5·1) |  |
|  | USA | 82·8 (75·4–89·2) | 4·5 (2·6–6·2) | 92·1 (86·7–95·9) | -0·2 (-1·6–0·9) | 69·0 (62·6–74·0) | 4·2 (2·9–5·5) |  |
|  | Southern Latin America | 79·9 (74·9–85·0) | 5·5 (2·0–9·6) | 91·7 (87·7–94·8) | 4·0 (1·6–8·2) | 74·4 (69·9–78·5) | 2·1 (0·1–4·4) |  |
|  | Argentina | 79·9 (75·0–85·1) | 4·2 (-0·7–9·5) | 92·0 (88·4–94·9) | 2·9 (0·1–7·6) | 75·8 (71·5–79·8) | 1·7 (-0·5–4·3) |  |
|  | Chile | 82·9 (77·6–88·0) | 10·6 (7·0–14·2) | 91·5 (87·4–94·9) | 10·3 (7·1–13·7) | 71·0 (65·9–75·8) | 6·5 (4·0–9·2) |  |
|  | Uruguay | 68·5 (62·0–74·7) | 3·0 (-0·1–5·9) | 89·0 (84·4–93·0) | 3·0 (0·6–5·7) | 70·7 (65·4–75·6) | 2·3 (0·6–4·3) |  |
|  | Western Europe | 77·5 (70·6–83·9) | 3·5 (1·8–5·4) | 88·0 (82·4–93·0) | 3·9 (3·0–4·9) | 60·1 (53·3–65·8) | 1·4 (0·8–2·0) |  |
|  | Andorra | 79·8 (72·5–86·4) | 3·2 (-0·2–6·7) | 93·4 (89·0–96·6) | 1·3 (-0·3–2·9) | 58·2 (51·0–64·2) | 1·6 (-0·2–3·4) |  |
|  | Austria | 78·9 (70·6–86·3) | 4·0 (0·7–7·4) | 92·0 (87·2–96·1) | 3·1 (1·1–5·3) | 59·1 (52·0–64·9) | 1·5 (0·1–2·9) |  |
|  | Belgium | 77·5 (69·8–85·2) | 3·6 (0·6–6·3) | 91·0 (85·9–95·2) | 3·1 (1·1–5·2) | 58·8 (51·9–64·9) | 1·5 (0·1–3·0) |  |
|  | Cyprus | 77·1 (68·9–84·7) | 9·2 (5·7–12·5) | 90·5 (85·6–95·0) | 12·1 (8·7–16·2) | 58·2 (51·3–64·0) | 2·1 (0·8–3·7) |  |
|  | Denmark | 77·7 (69·8–85·3) | 6·9 (2·9–10·4) | 91·1 (85·3–95·3) | 7·5 (4·7–11·4) | 59·0 (52·0–65·1) | 2·6 (0·6–4·5) |  |
|  | Finland | 86·8 (80·4–92·1) | 3·9 (-0·4–8·0) | 95·4 (91·7–97·8) | 2·6 (0·0–5·2) | 60·1 (53·5–66·2) | 2·7 (0·9–4·8) |  |
|  | France | 72·3 (64·8–80·0) | 8·3 (2·3–15·3) | 89·2 (83·8–93·8) | 8·6 (4·6–12·8) | 59·0 (52·0–64·8) | 1·9 (0·5–3·3) |  |
|  | Germany | 77·3 (69·6–84·7) | 2·4 (-0·6–4·8) | 89·7 (84·5–94·6) | 2·4 (0·3–4·5) | 59·9 (53·1–66·0) | 1·0 (-0·3–2·6) |  |
|  | Greece | 75·7 (68·6–82·9) | 0·6 (-1·7–3·4) | 86·9 (81·0–92·5) | -1·6 (-3·4–0·3) | 60·5 (53·8–66·6) | 1·8 (0·3–3·4) |  |
|  | Iceland | 78·7 (70·3–85·5) | 2·7 (-0·4–5·7) | 92·5 (88·3–96·1) | 1·5 (-0·4–3·3) | 59·9 (53·2–65·5) | 1·3 (-0·1–2·9) |  |
|  | Ireland | 79·1 (71·2–86·1) | 8·8 (4·4–12·6) | 92·6 (88·3–96·0) | 8·3 (5·2–11·9) | 59·6 (52·7–65·4) | 2·2 (0·7–3·7) |  |
|  | Israel | 76·4 (68·7–84·0) | 5·3 (2·1–8·7) | 89·8 (84·3–94·7) | 7·0 (3·9–10·1) | 59·3 (52·4–65·4) | 1·0 (-0·4–2·5) |  |
|  | Italy | 79·5 (71·3–86·7) | 2·3 (0·6–3·7) | 92·5 (88·0–96·1) | 1·2 (0·4–2·0) | 59·4 (52·8–65·0) | 1·9 (1·0–2·9) |  |
|  | Luxembourg | 75·6 (67·8–83·2) | 5·9 (1·0–11·6) | 90·7 (85·7–95·0) | 5·6 (2·7–9·2) | 58·4 (51·6–64·2) | 1·3 (-0·4–2·8) |  |
|  | Malta | 77·3 (69·1–84·9) | 4·6 (0·9–8·2) | 90·3 (84·8–94·7) | 4·4 (1·7–7·9) | 59·7 (52·6–65·5) | 2·6 (0·8–4·5) |  |
|  | Monaco | 78·0 (70·0–85·4) | 2·7 (-0·1–5·4) | 89·5 (83·9–94·6) | 2·3 (0·4–4·7) | 61·4 (54·6–67·3) | 1·3 (-0·1–2·7) |  |
|  | Netherlands | 79·7 (71·1–86·7) | 5·1 (1·4–8·9) | 92·7 (87·7–96·3) | 4·4 (1·9–7·0) | 59·6 (52·8–65·8) | 2·1 (0·6–3·7) |  |
|  | Norway | 78·6 (70·0–86·2) | 1·4 (-0·4–3·1) | 97·2 (94·9–98·6) | 1·0 (0·5–1·6) | 58·0 (51·3–63·3) | 0·4 (-0·8–1·8) |  |
|  | Portugal | 88·4 (82·6–93·2) | 4·3 (-1·0–9·4) | 95·4 (91·7–97·9) | 3·8 (0·7–6·3) | 60·1 (53·2–65·8) | 2·6 (0·9–4·3) |  |
|  | San Marino | 81·1 (73·0–88·3) | 2·2 (-0·9–4·9) | 93·1 (88·6–96·5) | 0·3 (-1·2–1·6) | 60·6 (54·1–66·6) | 1·4 (0·1–3·0) |  |
|  | Spain | 82·7 (77·4–87·1) | 2·4 (-1·0–5·6) | 71·5 (63·1–80·7) | 10·0 (4·3–16·0) | 60·8 (54·2–66·7) | 1·3 (-0·7–2·7) |  |
|  | Sweden | 80·2 (72·5–86·9) | 3·5 (0·1–6·9) | 93·8 (89·4–96·8) | 2·6 (0·8–4·6) | 59·1 (52·4–64·8) | 1·1 (-0·3–2·8) |  |
|  | Switzerland | 69·7 (62·4–77·5) | 5·7 (0·7–11·3) | 88·4 (83·1–92·9) | 6·0 (2·4–9·1) | 59·7 (52·9–65·9) | 2·0 (-2·1–5·9) |  |
|  | UK | 74·2 (66·7–81·1) | 3·2 (1·9–4·3) | 88·5 (83·4–93·2) | 1·1 (0·2–2·0) | 62·0 (55·3–67·6) | 0·2 (-0·6–1·0) |  |
|  | **Latin America and Caribbean** | **63·6 (55·8–71·5)** | **11·6 (9·5–13·4)** | **91·4 (87·4–94·7)** | **2·9 (2·0–4·1)** | **59·2 (51·7–65·4)** | **4·0 (3·3–4·6)** |  |
|  | Andean Latin America | 57·3 (51·1–63·4) | 7·2 (5·6–9·0) | 82·4 (76·8–87·6) | 8·8 (6·5–11·2) | 60·6 (54·4–66·5) | 6·0 (4·9–7·1) |  |
|  | Bolivia | 50·3 (44·1–56·9) | 4·6 (2·6–7·1) | 74·3 (66·3–81·4) | 11·2 (9·2–13·7) | 57·5 (49·9–64·0) | 4·6 (3·2–6·0) |  |
|  | Ecuador | 61·8 (55·1–69·1) | 8·8 (3·5–13·5) | 92·4 (89·1–95·0) | 3·3 (0·8–6·1) | 56·8 (50·9–62·6) | 6·2 (3·9–8·9) |  |
|  | Peru | 57·0 (50·7–63·6) | 6·8 (4·1–10·1) | 75·8 (68·5–82·6) | 13·4 (10·7–15·9) | 65·5 (58·3–71·3) | 5·0 (2·9–7·1) |  |
|  | Caribbean | 63·4 (57·0–70·1) | 4·3 (3·0–5·4) | 90·9 (86·8–94·1) | 1·0 (0·3–1·6) | 55·7 (49·0–61·8) | 3·7 (2·7–4·7) |  |
|  | Antigua and Barbuda | 67·2 (59·4–74·8) | 5·6 (2·7–8·1) | 93·2 (89·5–96·1) | 0·8 (-0·4–1·9) | 56·3 (49·5–62·4) | 4·5 (2·7–6·3) |  |
|  | The Bahamas | 61·3 (53·5–69·1) | 5·8 (3·3–8·3) | 89·2 (84·1–93·3) | 0·8 (-0·8–2·1) | 56·3 (48·7–63·1) | 4·9 (3·1–6·7) |  |
|  | Barbados | 69·9 (62·5–77·9) | 4·6 (1·8–7·6) | 92·1 (88·1–95·4) | 1·0 (-0·6–2·4) | 60·6 (53·1–67·0) | 4·3 (2·2–5·9) |  |
|  | Belize | 59·6 (53·0–66·8) | 7·4 (4·7–9·8) | 86·9 (81·2–91·4) | 5·0 (3·0–7·6) | 57·5 (50·0–63·9) | 3·3 (1·3–5·1) |  |
|  | Bermuda | 80·3 (73·0–86·3) | 7·1 (4·0–10·4) | 97·7 (96·1–98·7) | 0·8 (0·1–1·6) | 59·1 (53·2–64·6) | 4·8 (2·6–7·1) |  |
|  | Cuba | 73·3 (66·1–80·0) | 8·2 (5·2–11·2) | 96·2 (93·8–97·8) | 0·9 (0·3–1·8) | 56·4 (49·9–62·8) | 5·0 (2·9–6·8) |  |
|  | Dominica | 61·1 (53·8–68·6) | 2·1 (0·2–4·4) | 84·6 (78·4–90·1) | -1·2 (-2·9–0·3) | 58·5 (51·2–64·9) | 4·3 (2·5–6·0) |  |
|  | Dominican Republic | 57·5 (50·7–65·5) | 5·2 (3·2–6·9) | 87·5 (82·2–92·0) | 0·8 (-0·5–2·1) | 53·7 (47·0–60·1) | 4·0 (2·2–5·7) |  |
|  | Grenada | 63·9 (56·3–71·8) | 4·0 (1·7–6·5) | 89·4 (83·9–93·7) | -0·2 (-1·6–1·1) | 56·7 (49·4–62·9) | 4·6 (2·9–6·1) |  |
|  | Guyana | 69·2 (62·8–75·5) | 7·9 (4·6–11·5) | 91·3 (87·7–94·3) | 2·9 (0·8–6·1) | 53·5 (46·3–60·2) | 4·6 (2·4–7·4) |  |
|  | Haiti | 46·5 (41·0–53·1) | 2·3 (0·7–4·0) | 78·4 (71·4–84·9) | 3·7 (1·7–5·9) | 46·0 (38·4–53·4) | 2·2 (0·7–3·9) |  |
|  | Jamaica | 63·5 (56·3–70·8) | 3·2 (0·5–5·6) | 91·2 (86·7–94·5) | 0·2 (-1·0–1·4) | 56·5 (49·4–62·7) | 3·9 (2·3–5·4) |  |
|  | Puerto Rico | 78·1 (70·1–85·4) | 8·7 (4·9–12·0) | 96·4 (94·0–98·1) | 2·2 (1·1–3·9) | 60·2 (53·3–65·8) | 4·1 (2·0–6·5) |  |
|  | Saint Kitts and Nevis | 62·9 (55·0–71·2) | 6·8 (3·1–10·1) | 88·0 (82·1–92·7) | 1·4 (-1·1–3·8) | 56·4 (49·0–63·5) | 4·0 (1·2–6·7) |  |
|  | Saint Lucia | 52·3 (47·3–58·3) | 3·4 (-0·6–6·7) | 85·4 (80·3–89·4) | 0·6 (-2·1–3·2) | 52·6 (46·7–58·4) | 3·6 (1·1–6·9) |  |
|  | Saint Vincent and the Grenadines | 63·6 (55·9–71·6) | 5·4 (3·3–7·7) | 90·0 (84·9–93·9) | 0·8 (-0·5–2·2) | 55·0 (47·8–61·5) | 4·3 (2·4–6·2) |  |
|  | Suriname | 58·1 (50·9–66·1) | 7·1 (4·8–9·5) | 86·8 (81·2–91·5) | 3·0 (1·4–4·7) | 52·3 (44·9–59·2) | 3·9 (2·0–5·6) |  |
|  | Trinidad and Tobago | 66·0 (57·9–73·7) | 7·0 (4·4–9·8) | 90·3 (85·4–94·0) | 3·5 (1·7–5·4) | 57·4 (50·2–63·4) | 4·9 (3·5–7·0) |  |
|  | Virgin Islands | 72·7 (65·4–80·1) | 10·6 (6·6–14·0) | 93·8 (90·2–96·6) | 5·0 (2·9–7·7) | 60·3 (53·4–66·3) | 5·4 (2·9–7·8) |  |
|  | Central Latin America | 63·4 (55·7–71·5) | 13·1 (10·9–15·3) | 92·4 (88·7–95·4) | 2·4 (1·5–3·5) | 55·3 (47·6–61·9) | 6·8 (5·8–7·7) |  |
|  | Colombia | 67·3 (59·1–76·0) | 12·7 (8·5–17·2) | 96·1 (93·9–97·7) | 3·4 (2·0–4·9) | 69·4 (62·6–74·2) | 4·4 (2·3–6·7) |  |
|  | Costa Rica | 66·8 (58·6–75·0) | 9·8 (3·3–15·5) | 94·6 (91·7–97·0) | 2·4 (0·2–4·4) | 63·8 (56·6–69·8) | 4·8 (0·8–8·2) |  |
|  | El Salvador | 57·1 (49·6–65·5) | 6·9 (4·8–9·4) | 90·7 (86·4–94·5) | 3·0 (1·6–4·5) | 60·8 (53·5–67·4) | 2·8 (1·5–4·1) |  |
|  | Guatemala | 47·3 (43·6–50·8) | 5·9 (2·2–9·4) | 83·5 (79·0–87·3) | 5·2 (1·4–9·1) | 59·3 (52·0–65·3) | 3·1 (1·4–4·9) |  |
|  | Honduras | 46·6 (40·4–53·9) | 4·8 (3·3–6·6) | 84·3 (78·7–89·1) | 4·0 (2·6–5·4) | 55·6 (48·4–61·9) | 1·9 (0·6–3·1) |  |
|  | Mexico | 65·4 (57·1–74·4) | 15·5 (12·9–18·2) | 92·5 (88·6–95·7) | 2·5 (1·4–3·9) | 50·0 (42·6–56·8) | 7·2 (6·0–8·1) |  |
|  | Nicaragua | 56·8 (49·8–64·7) | 8·7 (6·3–11·5) | 91·8 (88·1–95·1) | 3·8 (2·3–5·4) | 59·6 (51·8–65·8) | 2·8 (1·2–4·3) |  |
|  | Panama | 66·8 (58·5–75·0) | 9·3 (6·3–12·5) | 96·0 (93·5–97·8) | 1·7 (0·8–2·6) | 59·3 (51·7–65·7) | 3·5 (2·0–5·0) |  |
|  | Venezuela | 56·7 (48·9–65·1) | 5·9 (3·4–8·5) | 89·6 (84·5–93·7) | -0·6 (-2·3–0·9) | 61·0 (53·3–67·4) | 3·2 (1·7–4·9) |  |
|  | Tropical Latin America | 65·3 (57·1–73·8) | 12·2 (9·5–14·5) | 91·3 (86·9–95·0) | 4·0 (2·6–5·5) | 66·9 (59·7–72·5) | 0·7 (0·0–1·4) |  |
|  | Brazil | 65·5 (57·2–74·0) | 12·5 (9·7–14·9) | 91·4 (87·0–95·0) | 4·1 (2·7–5·6) | 66·9 (59·8–72·5) | 0·8 (0·1–1·4) |  |
|  | Paraguay | 59·8 (52·4–68·9) | 3·3 (1·1–5·5) | 88·1 (81·9–93·0) | 0·7 (-0·6–1·9) | 65·7 (58·0–71·7) | -0·4 (-2·1–0·9) |  |
|  | **North Africa and Middle East** | **65·4 (59·8–71·2)** | **9·0 (7·3–11·0)** | **92·5 (89·5–94·8)** | **4·1 (2·7–5·4)** | **62·4 (56·3–67·5)** | **5·3 (4·2–6·6)** |  |
|  | Afghanistan | 41·5 (36·0–47·9) | 3·9 (-0·6–9·1) | 92·8 (89·6–95·0) | 3·8 (1·2–7·6) | 70·2 (64·1–75·6) | 2·4 (-1·3–6·4) |  |
|  | Algeria | 64·7 (59·8–69·8) | 14·6 (11·5–17·9) | 93·1 (90·4–95·2) | 3·4 (1·4–5·8) | 59·6 (54·6–64·5) | 1·9 (-1·2–5·0) |  |
|  | Bahrain | 68·9 (59·3–77·9) | 14·0 (9·7–18·3) | 94·4 (90·9–97·2) | 5·8 (3·5–8·5) | 62·5 (54·8–69·2) | 5·1 (3·3–6·9) |  |
|  | Egypt | 61·8 (57·9–66·2) | -2·9 (-5·5–1·3) | 89·2 (85·9–92·1) | -0·9 (-2·5–1·5) | 61·5 (54·0–67·8) | 5·4 (3·9–7·0) |  |
|  | Iran | 72·4 (65·3–79·5) | 9·7 (8·1–11·5) | 86·8 (80·6–92·1) | 4·8 (3·4–6·4) | 58·0 (50·7–64·2) | -2·3 (-3·5–-1·2) |  |
|  | Iraq | 60·3 (54·9–66·9) | 14·2 (9·8–19·0) | 94·1 (91·9–96·0) | 9·7 (5·7–14·1) | 54·6 (49·3–59·8) | 2·9 (-0·4–6·5) |  |
|  | Jordan | 78·8 (73·2–83·5) | 16·8 (11·6–21·1) | 95·0 (92·6–96·9) | 10·3 (6·1–13·8) | 60·7 (55·9–65·1) | 11·3 (7·6–14·9) |  |
|  | Kuwait | 74·7 (67·2–82·4) | 6·8 (0·7–11·9) | 94·9 (91·7–97·3) | 1·5 (-0·6–3·9) | 53·0 (45·7–60·1) | 10·3 (6·0–15·5) |  |
|  | Lebanon | 51·3 (46·0–57·4) | 7·9 (3·6–11·3) | 94·3 (91·7–96·5) | 5·4 (2·7–9·0) | 65·7 (59·7–70·9) | 4·6 (2·4–7·6) |  |
|  | Libya | 56·6 (49·3–64·8) | 4·5 (0·4–9·7) | 84·9 (79·2–90·7) | -3·2 (-6·8–0·4) | 64·1 (56·5–70·4) | 5·5 (2·5–8·6) |  |
|  | Morocco | 55·5 (50·6–60·6) | 9·3 (5·3–13·5) | 89·2 (85·8–92·1) | 6·8 (3·2–11·1) | 53·0 (46·6–58·8) | 5·5 (2·6–8·7) |  |
|  | Oman | 63·3 (55·1–72·1) | 6·6 (2·8–10·1) | 93·4 (89·3–96·3) | 1·4 (-0·2–2·7) | 62·6 (55·9–69·2) | 7·0 (5·2–8·5) |  |
|  | Palestine | 70·6 (62·2–78·5) | 10·0 (3·5–17·1) | 95·5 (92·6–97·7) | 4·7 (2·5–6·9) | 45·2 (37·8–51·8) | 3·9 (0·1–7·6) |  |
|  | Qatar | 75·5 (67·4–83·5) | 10·8 (5·1–16·5) | 95·1 (91·5–97·7) | 7·3 (3·6–11·6) | 60·3 (53·2–66·9) | 6·3 (4·0–8·8) |  |
|  | Saudi Arabia | 68·3 (59·4–78·0) | 10·0 (4·6–16·5) | 98·4 (97·3–99·3) | 1·2 (0·4–2·1) | 80·5 (77·1–83·5) | 16·4 (11·9–21·8) |  |
|  | Sudan | 58·6 (53·7–63·8) | 8·3 (4·6–12·0) | 90·5 (87·5–93·3) | 5·5 (2·5–9·6) | 41·4 (35·9–46·9) | 4·7 (2·3–7·1) |  |
|  | Syria | 67·3 (58·6–76·6) | 11·6 (8·3–15·0) | 92·9 (88·8–96·2) | 2·1 (0·8–3·5) | 61·3 (53·3–67·6) | 5·1 (3·4–7·3) |  |
|  | Tunisia | 75·6 (67·1–84·1) | 11·1 (7·4–15·1) | 96·8 (94·5–98·4) | 2·2 (1·1–3·8) | 58·5 (50·4–65·0) | 4·0 (2·3–5·9) |  |
|  | Türkiye | 73·6 (65·9–81·2) | 16·0 (12·1–20·2) | 95·3 (92·2–97·5) | 7·5 (4·7–10·4) | 65·9 (58·7–71·4) | 4·7 (3·1–6·4) |  |
|  | United Arab Emirates | 76·0 (66·8–85·3) | 15·8 (9·9–21·2) | 94·2 (90·5–97·2) | 1·2 (-1·0–3·8) | 73·7 (68·5–78·4) | 6·5 (3·2–10·3) |  |
|  | Yemen | 53·6 (46·3–62·6) | 5·5 (3·1–7·7) | 90·2 (86·0–94·0) | 4·1 (2·4–5·8) | 52·2 (45·3–58·7) | 2·6 (1·3–4·0) |  |
|  | **South Asia** | **43·8 (37·0–51·2)** | **12·0 (9·9–14·3)** | **95·7 (93·7–97·1)** | **6·3 (4·6–8·2)** | **45·5 (38·5–52·0)** | **1·4 (0·4–2·4)** |  |
|  | Bangladesh | 51·0 (47·6–54·9) | 5·4 (1·4–9·6) | 91·8 (89·7–93·5) | 5·2 (2·4–8·9) | 56·9 (51·7–61·8) | -3·6 (-7·8–1·2) |  |
|  | Bhutan | 55·4 (49·4–62·2) | 16·9 (12·2–22·0) | 91·4 (87·6–94·4) | 11·5 (8·4–15·6) | 76·6 (71·4–80·6) | 5·3 (2·5–7·8) |  |
|  | India | 43·6 (36·1–51·7) | 13·9 (11·4–16·8) | 97·2 (95·8–98·2) | 5·1 (3·7–6·9) | 42·9 (35·7–49·7) | 3·1 (2·3–4·0) |  |
|  | Nepal | 41·4 (36·1–47·1) | 5·1 (2·7–7·5) | 81·5 (75·4–86·8) | 8·9 (5·9–12·6) | 49·1 (42·4–55·3) | 2·9 (0·8–5·2) |  |
|  | Pakistan | 41·2 (35·1–48·4) | 4·5 (2·9–6·3) | 87·3 (82·6–91·3) | 7·5 (5·9–9·2) | 60·8 (53·3–67·2) | -0·1 (-2·1–1·6) |  |
|  | **Southeast Asia, east Asia, and Oceania** | **47·2 (41·0–54·1)** | **10·0 (7·3–13·1)** | **87·5 (83·0–91·5)** | **15·2 (12·5–17·7)** | **58·8 (51·6–64·9)** | **2·4 (1·3–3·4)** |  |
|  | East Asia | 45·4 (39·2–52·3) | 9·4 (6·7–12·8) | 86·5 (81·6–90·7) | 17·2 (14·2–19·9) | 58·0 (50·8–64·4) | 2·1 (0·8–3·3) |  |
|  | China | 45·2 (39·0–52·1) | 9·5 (6·7–12·9) | 86·4 (81·5–90·7) | 17·6 (14·6–20·3) | 58·0 (50·7–64·3) | 2·0 (0·8–3·3) |  |
|  | North Korea | 40·0 (34·8–46·3) | 4·5 (2·6–6·4) | 78·8 (72·1–84·8) | 10·4 (8·0–12·5) | 55·2 (47·6–61·6) | 1·3 (0·0–3·1) |  |
|  | Taiwan | 57·0 (49·2–65·3) | 11·3 (8·7–14·6) | 92·7 (89·1–95·6) | 4·5 (2·9–6·7) | 61·1 (53·6–67·3) | 3·1 (1·2–5·0) |  |
|  | Oceania | 45·4 (39·7–52·4) | 3·1 (1·3–5·1) | 82·1 (75·8–87·8) | 1·8 (0·5–3·3) | 36·7 (29·3–43·3) | 1·5 (-0·1–2·8) |  |
|  | American Samoa | 53·3 (50·5–56·6) | 9·8 (5·8–13·7) | 85·4 (82·7–87·9) | 0·5 (-2·8–4·0) | 55·6 (51·4–59·6) | 8·3 (4·1–12·8) |  |
|  | Cook Islands | 46·3 (41·1–52·9) | 8·3 (5·3–12·1) | 85·3 (80·0–89·9) | 7·1 (5·1–9·5) | 51·0 (44·0–58·0) | 5·2 (2·6–7·7) |  |
|  | Fiji | 61·0 (53·5–68·8) | 4·4 (0·0–9·6) | 81·7 (74·0–88·8) | -0·5 (-5·5–3·6) | 45·4 (36·7–52·4) | 3·7 (0·3–6·5) |  |
|  | Guam | 60·8 (52·8–69·8) | 7·3 (4·3–10·6) | 93·4 (89·9–96·4) | 0·6 (-0·4–1·7) | 40·0 (33·2–46·6) | 2·9 (1·1–5·0) |  |
|  | Kiribati | 40·8 (36·0–46·3) | 1·9 (0·0–3·9) | 69·6 (61·2–76·1) | -1·0 (-3·9–1·8) | 32·0 (25·7–38·3) | 3·2 (1·4–5·1) |  |
|  | Marshall Islands | 40·3 (38·3–42·4) | 3·3 (-0·4–6·5) | 67·2 (64·1–70·0) | 2·1 (-3·1–7·6) | 33·1 (29·4–36·5) | 6·2 (2·8–9·4) |  |
|  | Federated States of Micronesia | 37·5 (35·5–39·9) | 3·9 (1·8–6·5) | 68·7 (65·7–72·3) | 1·3 (-2·4–5·4) | 33·0 (28·7–36·9) | 0·3 (-2·9–3·0) |  |
|  | Nauru | 60·7 (54·8–65·8) | 4·6 (2·7–6·8) | 67·8 (60·5–75·0) | 8·0 (5·4–11·2) | 47·8 (40·6–53·8) | -0·9 (-4·1–2·1) |  |
|  | Niue | 57·4 (50·5–65·4) | 0·9 (-1·0–2·8) | 81·6 (74·3–88·1) | -2·7 (-4·4–-0·9) | 40·3 (33·6–46·9) | 3·1 (1·5–4·8) |  |
|  | Northern Mariana Islands | 54·1 (47·3–63·3) | 12·3 (8·1–17·4) | 86·8 (80·2–92·3) | 0·6 (-2·7–4·0) | 42·2 (33·5–49·5) | 4·1 (0·0–8·3) |  |
|  | Palau | 53·4 (50·6–56·2) | 6·2 (0·7–11·1) | 79·8 (76·9–83·0) | -0·1 (-5·3–5·5) | 39·8 (35·1–43·5) | 4·9 (0·3–9·3) |  |
|  | Papua New Guinea | 42·8 (36·6–50·6) | 3·9 (2·1–6·1) | 86·0 (80·5–90·8) | 3·6 (2·1–5·1) | 31·4 (25·0–38·0) | 1·2 (-0·2–2·5) |  |
|  | Samoa | 36·5 (31·9–41·1) | -2·1 (-4·9–0·5) | 75·4 (68·4–80·7) | 0·6 (-2·8–3·9) | 43·3 (36·4–49·9) | 3·0 (0·7–5·6) |  |
|  | Solomon Islands | 36·7 (32·5–41·5) | 0·9 (-0·5–2·4) | 46·1 (39·0–53·9) | -0·3 (-2·9–2·1) | 46·3 (38·0–54·1) | 1·1 (-0·9–3·3) |  |
|  | Tokelau | 59·7 (54·2–64·6) | 0·7 (-1·6–3·3) | 72·4 (65·9–78·6) | -2·6 (-5·6–0·5) | 25·9 (21·5–30·9) | 2·5 (0·8–4·3) |  |
|  | Tonga | 50·6 (44·1–57·9) | 3·2 (1·5–5·4) | 78·2 (71·1–85·2) | -0·1 (-2·3–1·7) | 38·8 (31·6–45·3) | 3·3 (1·8–5·0) |  |
|  | Tuvalu | 58·3 (52·3–65·0) | 2·5 (-0·1–4·9) | 80·7 (72·9–87·0) | 1·1 (-1·9–3·8) | 32·0 (25·6–38·2) | 2·8 (0·4–5·0) |  |
|  | Vanuatu | 24·8 (21·2–29·2) | 1·8 (0·8–2·7) | 77·8 (71·2–83·5) | 2·2 (0·8–3·7) | 52·8 (45·2–59·9) | 2·1 (0·8–3·5) |  |
|  | Southeast Asia | 52·4 (46·1–59·4) | 9·8 (7·5–12·5) | 90·2 (86·2–93·8) | 7·0 (5·7–8·7) | 60·8 (53·7–66·5) | 2·8 (1·4–4·0) |  |
|  | Cambodia | 61·5 (54·3–70·2) | 9·7 (6·0–14·2) | 88·7 (82·8–93·5) | 11·5 (8·8–14·9) | 64·4 (57·3–70·0) | 1·8 (0·0–3·4) |  |
|  | Indonesia | 48·1 (41·6–56·3) | 8·8 (6·8–11·5) | 87·5 (82·3–92·1) | 7·2 (5·7–8·9) | 58·3 (50·3–65·0) | 2·7 (1·4–3·8) |  |
|  | Laos | 53·2 (47·7–59·2) | 6·7 (4·5–9·0) | 84·6 (79·6–89·3) | 14·8 (11·1–18·3) | 42·9 (35·7–49·7) | 2·2 (0·5–3·9) |  |
|  | Malaysia | 47·4 (40·0–55·5) | 11·9 (5·5–19·5) | 96·5 (94·7–98·0) | 2·8 (1·2–4·1) | 68·9 (62·2–74·1) | 2·6 (-1·1–6·6) |  |
|  | Maldives | 43·3 (38·0–49·7) | 6·0 (3·4–8·8) | 90·8 (87·3–93·6) | 8·4 (5·6–11·8) | 62·2 (55·3–68·5) | 4·1 (1·9–6·1) |  |
|  | Mauritius | 60·2 (52·6–69·4) | 8·5 (5·1–12·4) | 92·6 (88·5–96·0) | 2·2 (0·9–4·1) | 64·1 (56·2–70·2) | 4·0 (1·7–6·1) |  |
|  | Myanmar | 56·3 (51·2–61·7) | 10·8 (6·8–14·5) | 89·0 (84·9–92·0) | 13·7 (9·3–18·8) | 50·5 (43·8–56·0) | 2·6 (-0·1–5·2) |  |
|  | Philippines | 47·8 (41·3–55·5) | 5·1 (3·8–6·6) | 88·5 (83·6–92·8) | 2·7 (1·9–3·5) | 58·5 (50·8–65·1) | 1·8 (1·0–2·6) |  |
|  | Seychelles | 60·6 (52·3–69·7) | 7·9 (3·4–13·8) | 94·9 (91·8–97·2) | 1·3 (0·0–2·7) | 36·1 (29·5–42·9) | 3·9 (1·4–6·4) |  |
|  | Sri Lanka | 73·2 (68·1–78·2) | 14·0 (9·1–19·0) | 90·3 (86·6–93·7) | 14·0 (8·7–19·9) | 66·4 (61·6–70·4) | 3·6 (0·4–7·6) |  |
|  | Thailand | 59·6 (50·8–69·1) | 16·2 (8·8–22·8) | 96·6 (94·8–98·0) | 2·0 (0·8–3·1) | 55·9 (48·3–62·8) | 4·7 (-0·2–10·2) |  |
|  | Timor-Leste | 24·7 (20·2–29·7) | 2·4 (0·9–4·4) | 81·3 (74·2–87·2) | 9·9 (6·7–13·7) | 67·8 (59·4–75·1) | 1·1 (-1·0–3·5) |  |
|  | Viet Nam | 51·3 (45·4–57·9) | 8·8 (4·1–12·7) | 91·2 (87·9–94·3) | 7·0 (4·0–10·0) | 76·7 (71·7–80·8) | -0·3 (-2·7–1·9) |  |
|  | **Sub-Saharan Africa** | **34·2 (29·7–38·8)** | **0·8 (0·2–1·7)** | **81·0 (75·2–85·9)** | **5·4 (4·5–6·6)** | **63·3 (56·7–68·9)** | **1·0 (0·4–1·5)** |  |
|  | Central sub-Saharan Africa | 16·3 (14·0–18·9) | 0·6 (-0·2–1·4) | 69·1 (62·5–75·3) | 11·6 (9·8–13·5) | 81·2 (75·7–85·3) | 2·5 (1·6–3·6) |  |
|  | Angola | 18·2 (15·8–20·6) | -0·3 (-1·6–0·8) | 62·8 (56·1–69·4) | 13·4 (11·1–15·8) | 86·4 (81·4–90·4) | 2·2 (1·1–3·6) |  |
|  | Central African Republic | 15·9 (13·9–18·2) | 1·3 (0·6–2·0) | 77·3 (71·6–82·0) | 3·9 (2·1–5·8) | 65·1 (58·4–70·9) | 1·8 (0·1–3·3) |  |
|  | Congo (Brazzaville) | 17·2 (14·9–19·6) | 0·9 (0·0–1·8) | 71·1 (64·3–77·3) | 10·4 (8·3–12·6) | 79·8 (74·8–83·8) | 2·1 (1·1–3·2) |  |
|  | DR Congo | 15·3 (13·0–17·6) | 0·6 (-0·2–1·5) | 71·8 (64·6–78·2) | 12·5 (10·0–15·0) | 78·9 (72·6–83·5) | 1·5 (0·3–2·9) |  |
|  | Equatorial Guinea | 16·4 (14·0–18·9) | 0·4 (-0·6–1·5) | 77·6 (71·6–83·3) | 14·0 (11·4–17·4) | 80·9 (76·1–84·5) | 3·1 (1·8–4·8) |  |
|  | Gabon | 19·9 (17·1–22·7) | 1·8 (0·7–2·9) | 71·3 (63·8–77·5) | 8·4 (6·3–11·0) | 82·7 (77·8–86·1) | 2·8 (1·9–3·8) |  |
|  | Eastern sub-Saharan Africa | 37·8 (33·0–42·6) | 2·0 (1·1–3·1) | 79·8 (74·1–84·9) | 7·7 (6·2–9·2) | 63·0 (56·8–68·5) | 1·0 (0·3–1·7) |  |
|  | Burundi | 36·7 (31·7–42·3) | 0·0 (-1·6–1·8) | 82·9 (77·2–87·8) | 6·8 (5·0–8·9) | 60·7 (53·9–66·3) | 0·3 (-1·3–1·8) |  |
|  | Comoros | 53·1 (45·9–60·0) | 5·4 (3·4–7·5) | 90·8 (86·4–94·5) | 3·9 (2·3–5·7) | 66·9 (60·9–71·9) | 2·0 (0·6–3·4) |  |
|  | Djibouti | 42·0 (36·2–48·8) | 6·7 (4·7–9·2) | 86·0 (81·1–90·4) | 7·9 (5·9–9·8) | 62·8 (56·0–68·5) | 1·6 (0·3–2·7) |  |
|  | Eritrea | 58·7 (51·7–66·6) | 7·6 (3·8–11·7) | 92·8 (88·5–96·0) | 4·3 (2·3–6·7) | 61·8 (55·1–67·2) | 0·9 (-1·1–2·7) |  |
|  | Ethiopia | 22·5 (19·1–25·3) | -0·8 (-2·3–0·5) | 70·1 (62·8–75·8) | 19·5 (17·5–21·3) | 62·9 (57·0–68·3) | 1·5 (0·3–2·9) |  |
|  | Kenya | 46·4 (40·7–52·8) | 0·5 (-0·3–1·3) | 85·1 (79·9–89·8) | -1·7 (-2·3–-1·0) | 55·0 (48·3–61·1) | 1·5 (0·7–2·3) |  |
|  | Madagascar | 37·9 (32·8–43·4) | 1·8 (0·1–3·7) | 85·2 (80·4–89·5) | 5·3 (3·4–7·4) | 60·4 (53·5–65·8) | 0·4 (-1·2–2·1) |  |
|  | Malawi | 46·4 (40·9–51·9) | 0·8 (-1·2–2·7) | 78·2 (71·5–84·2) | 7·3 (4·6–10·2) | 70·6 (63·9–75·9) | 0·5 (-1·5–2·4) |  |
|  | Mozambique | 44·0 (36·9–51·4) | 0·5 (-1·4–2·9) | 87·3 (81·8–91·8) | 2·7 (1·2–4·5) | 67·6 (60·7–73·5) | 1·4 (-0·1–2·9) |  |
|  | Rwanda | 34·0 (29·3–38·6) | 5·5 (3·6–7·7) | 84·4 (79·0–88·7) | 15·8 (12·7–19·4) | 60·8 (52·6–68·2) | 1·7 (-0·3–3·7) |  |
|  | Somalia | 36·4 (31·7–40·8) | 0·8 (-0·7–2·2) | 71·6 (64·4–78·5) | 1·7 (-0·2–3·9) | 62·7 (56·4–68·3) | 1·4 (-0·1–3·0) |  |
|  | South Sudan | 36·9 (32·2–42·1) | 1·2 (-0·6–3·1) | 79·1 (72·7–84·7) | 1·5 (-0·6–3·4) | 61·3 (54·2–67·2) | -0·6 (-2·4–0·9) |  |
|  | Uganda | 47·1 (41·8–52·5) | 4·0 (1·8–6·2) | 84·7 (79·5–89·6) | 9·5 (6·5–12·2) | 81·1 (77·0–84·5) | 0·3 (-1·0–1·5) |  |
|  | Tanzania | 48·2 (42·4–53·7) | 2·5 (0·8–4·4) | 77·0 (70·1–83·3) | 5·4 (3·4–7·3) | 56·7 (50·5–62·6) | 1·6 (0·2–3·2) |  |
|  | Zambia | 25·2 (21·9–28·5) | -1·2 (-3·0–0·5) | 76·0 (69·9–81·3) | 9·3 (6·5–12·2) | 44·8 (37·6–51·2) | 2·0 (-0·1–4·6) |  |
|  | Southern sub-Saharan Africa | 52·4 (45·0–59·8) | 0·5 (-0·6–1·8) | 88·7 (83·9–92·9) | -1·1 (-1·9–-0·4) | 65·5 (58·5–71·0) | -2·0 (-2·8–-1·3) |  |
|  | Botswana | 51·8 (46·2–58·2) | 7·0 (4·5–9·3) | 91·7 (88·1–94·6) | 0·6 (-0·8–1·9) | 65·6 (58·7–70·6) | 5·8 (3·9–7·6) |  |
|  | Eswatini | 46·8 (40·6–53·3) | 5·7 (3·5–8·0) | 94·0 (91·3–96·1) | 0·6 (-0·2–1·5) | 77·9 (72·7–81·9) | 2·1 (0·7–3·4) |  |
|  | Lesotho | 57·2 (50·2–64·4) | -1·0 (-2·8–1·2) | 92·2 (88·5–95·1) | -1·5 (-2·5–-0·7) | 62·6 (55·8–68·4) | 1·7 (0·2–3·0) |  |
|  | Namibia | 54·2 (46·2–62·4) | 4·5 (1·6–7·3) | 95·0 (92·7–97·0) | 1·2 (0·2–2·4) | 61·5 (55·0–67·0) | 3·1 (1·1–5·0) |  |
|  | South Africa | 52·8 (45·3–60·4) | 0·3 (-0·9–1·8) | 88·0 (83·0–92·4) | -1·0 (-1·8–-0·2) | 65·5 (58·4–71·0) | -2·8 (-3·8–-2·0) |  |
|  | Zimbabwe | 48·5 (41·6–55·6) | -1·2 (-3·4–0·8) | 92·1 (88·7–94·8) | -2·9 (-4·4–-1·9) | 64·8 (58·7–70·4) | 1·2 (-0·3–2·5) |  |
|  | Western sub-Saharan Africa | 32·0 (27·8–36·5) | 1·6 (0·7–2·7) | 80·1 (74·3–85·3) | 8·1 (6·7–9·6) | 56·5 (49·7–62·4) | 2·7 (1·9–3·6) |  |
|  | Benin | 25·9 (22·2–29·3) | 0·1 (-1·4–1·6) | 85·3 (80·6–88·7) | 6·5 (4·6–8·9) | 59·1 (52·4–65·0) | 1·3 (-0·7–3·2) |  |
|  | Burkina Faso | 30·6 (26·5–34·6) | 0·6 (-0·5–1·8) | 80·1 (73·9–85·2) | 5·8 (4·0–7·7) | 53·2 (46·5–59·3) | 1·1 (-0·3–2·4) |  |
|  | Cabo Verde | 57·4 (51·8–63·0) | 10·1 (6·1–14·0) | 91·5 (87·9–94·1) | 5·2 (2·4–8·5) | 63·1 (57·3–68·1) | 2·8 (0·3–5·8) |  |
|  | Cameroon | 41·3 (35·8–46·9) | 1·0 (-2·2–3·9) | 87·6 (83·4–91·7) | 5·0 (2·7–7·4) | 64·5 (57·8–69·9) | 1·8 (-0·2–3·9) |  |
|  | Chad | 26·0 (22·7–30·0) | -1·1 (-2·1–0·0) | 74·9 (68·5–80·9) | 5·6 (3·9–7·6) | 51·0 (43·9–57·0) | 0·5 (-0·9–1·9) |  |
|  | Côte d'Ivoire | 27·5 (24·1–32·0) | 1·7 (0·3–3·2) | 76·3 (69·7–82·2) | 8·3 (6·6–10·3) | 55·3 (48·3–61·3) | 2·6 (0·9–4·2) |  |
|  | The Gambia | 27·9 (24·2–31·6) | 1·0 (0·0–1·8) | 76·3 (70·4–82·1) | 4·7 (3·1–6·7) | 55·7 (48·9–61·6) | 2·6 (1·1–3·9) |  |
|  | Ghana | 50·5 (44·8–56·7) | 1·8 (-0·3–4·1) | 79·2 (72·3–85·5) | 5·9 (3·7–8·4) | 54·8 (48·1–60·8) | 2·9 (1·4–4·5) |  |
|  | Guinea | 34·5 (30·0–39·3) | -2·2 (-4·0–-0·2) | 75·1 (68·2–81·3) | 6·4 (3·9–8·8) | 64·6 (56·2–71·0) | 0·7 (-1·5–2·9) |  |
|  | Guinea-Bissau | 26·0 (22·6–29·5) | -0·4 (-1·6–0·7) | 68·8 (61·7–75·3) | 8·5 (6·5–10·2) | 53·4 (46·6–59·1) | 1·6 (-0·3–3·1) |  |
|  | Liberia | 31·9 (27·4–36·9) | 1·4 (-0·7–3·3) | 83·8 (77·8–88·8) | 5·9 (2·9–8·8) | 54·0 (46·4–60·5) | 1·8 (-0·9–4·6) |  |
|  | Mali | 23·3 (20·0–26·7) | 1·7 (0·5–3·1) | 74·7 (68·1–80·3) | 6·5 (4·4–9·2) | 52·9 (45·4–58·7) | 2·3 (0·3–4·0) |  |
|  | Mauritania | 27·4 (22·5–33·1) | 2·3 (0·6–4·6) | 74·4 (65·9–82·1) | 10·4 (7·9–12·8) | 56·7 (47·7–65·2) | 4·4 (2·5–6·4) |  |
|  | Niger | 10·7 (8·5–12·9) | 0·6 (-0·2–1·4) | 68·7 (59·3–78·1) | 11·2 (7·5–13·9) | 31·3 (24·0–37·9) | 1·0 (-0·5–2·7) |  |
|  | Nigeria | 32·3 (27·8–36·8) | 2·0 (0·9–3·4) | 81·7 (76·0–86·6) | 9·7 (8·1–11·6) | 56·6 (49·6–62·4) | 3·2 (2·2–4·2) |  |
|  | São Tomé and Príncipe | 34·6 (30·3–39·0) | 0·5 (-1·9–2·5) | 76·3 (70·4–81·9) | 7·5 (4·5–10·4) | 57·1 (49·9–63·0) | 0·6 (-2·1–3·3) |  |
|  | Senegal | 29·6 (25·9–33·8) | 1·8 (0·5–3·3) | 80·1 (74·5–85·1) | 7·6 (5·8–9·3) | 55·0 (48·1–60·9) | 1·9 (0·6–3·2) |  |
|  | Sierra Leone | 27·8 (24·3–32·0) | -2·7 (-4·3–-1·2) | 78·0 (71·3–83·4) | 6·4 (4·1–9·0) | 53·8 (47·0–59·6) | 1·4 (-0·8–3·5) |  |
|  | Togo | 30·4 (26·4–34·7) | 3·9 (2·6–5·4) | 77·9 (71·6–83·4) | 7·5 (5·7–9·2) | 54·8 (48·0–61·0) | 2·9 (1·4–4·2) |  |
|  |  |  |  |  |  |  |  |  |

## Table S17. Diagnosed proportion among people living with diabetes, treated proportion among people with diagnosed diabetes, and optimal glycaemic management proportion among people with treated diabetes in 2000, and corresponding counts, for both sexes and ages 15 and older globally, and by Global Burden of Disease super-regions, regions, and countries and territories

|  | **Location** | **Diagnosed, 2000 (%)** | **Diagnosed, 2000 (per 1,000)** | **Treated, 2000 (%)** | **Treated, 2000 (per 1,000)** | **Optimal Glycaemic Management, 2000 (%)** | **Optimal Glycaemic Management, 2000 (per 1,000)** |  |
| --- | --- | --- | --- | --- | --- | --- | --- | --- |
|  | **Global** | **47·5 (42·1–53·1)** | **129000 (112000–148000)** | **84·2 (79·3–88·4)** | **109000 (91600–129000)** | **40·3 (34·5–46·8)** | **43800 (34800–54100)** |  |
|  | **Central Europe, eastern Europe, and central Asia** | **62·4 (54·8–70·4)** | **9500 (8020–11000)** | **89·3 (83·9–93·7)** | **8500 (6920–10200)** | **46·8 (40·1–53·7)** | **3970 (3160–4930)** |  |
|  | Central Asia | 46·9 (40·4–53·3) | 990 (843–1140) | 80·4 (72·9–87·0) | 797 (649–973) | 41·3 (35·3–48·3) | 329 (251–415) |  |
|  | Armenia | 54·7 (46·5–63·3) | 80·6 (66·6–93) | 83·7 (74·7–90·7) | 67·5 (54·2–82·9) | 38·3 (31·6–46·0) | 25·8 (19–34) |  |
|  | Azerbaijan | 54·1 (47·4–62·5) | 144 (122–173) | 88·0 (81·9–92·8) | 127 (102–159) | 27·1 (21·7–33·3) | 34·3 (26–44·2) |  |
|  | Georgia | 67·4 (59·5–75·0) | 114 (96·7–133) | 87·0 (80·1–93·0) | 99·8 (81·5–121) | 43·7 (36·8–51·3) | 43·6 (34–55·5) |  |
|  | Kazakhstan | 47·2 (40·4–54·8) | 275 (222–322) | 78·5 (69·0–86·2) | 216 (174–265) | 66·6 (60·4–73·0) | 144 (113–177) |  |
|  | Kyrgyzstan | 45·2 (38·6–52·1) | 48·9 (40·8–57·5) | 85·7 (78·9–90·6) | 42 (33·4–51·5) | 20·6 (16·1–25·7) | 8·64 (6·28–11·3) |  |
|  | Mongolia | 38·3 (33·9–42·6) | 18·8 (16·2–21·6) | 65·3 (57·7–72·4) | 12·3 (10·1–15·2) | 38·0 (32·2–44·7) | 4·68 (3·64–5·95) |  |
|  | Tajikistan | 36·3 (31·7–40·8) | 48·3 (40·3–56·8) | 55·1 (47·1–63·5) | 26·6 (21·9–32·8) | 22·2 (16·1–30·6) | 5·91 (3·95–8·71) |  |
|  | Turkmenistan | 26·1 (22·4–30·1) | 23·5 (19·7–27·5) | 74·2 (66·9–80·7) | 17·4 (14–21·3) | 24·4 (18·7–30·8) | 4·25 (3·02–5·72) |  |
|  | Uzbekistan | 41·8 (36·3–47·6) | 235 (201–270) | 80·0 (73·2–86·4) | 189 (154–226) | 33·9 (28·7–40·3) | 64 (47·1–82·6) |  |
|  | Central Europe | 69·1 (61·4–77·2) | 4650 (3940–5370) | 90·4 (85·1–94·7) | 4200 (3460–5030) | 46·9 (40·1–53·9) | 1970 (1580–2440) |  |
|  | Albania | 60·7 (52·8–69·0) | 37·8 (31·5–45·3) | 86·3 (79·8–92·0) | 32·7 (25·9–40·3) | 39·9 (33·6–46·9) | 13 (9·7–16·8) |  |
|  | Bosnia and Herzegovina | 62·1 (53·4–72·2) | 106 (87·9–132) | 84·7 (76·8–91·4) | 90·4 (70·1–121) | 39·7 (33·1–46·8) | 35·8 (26·3–48·7) |  |
|  | Bulgaria | 62·7 (54·2–72·0) | 286 (239–336) | 83·6 (75·6–90·4) | 240 (189–302) | 40·7 (34·0–48·1) | 97·4 (70·4–126) |  |
|  | Croatia | 69·6 (60·3–80·1) | 159 (133–183) | 91·1 (84·9–95·9) | 145 (114–175) | 40·8 (33·7–48·1) | 59·1 (43·5–75·8) |  |
|  | Czechia | 57·5 (50·9–64·5) | 237 (203–273) | 78·5 (70·9–85·4) | 187 (154–226) | 28·9 (23·6–34·8) | 54 (40·2–69·1) |  |
|  | Hungary | 66·3 (57·7–75·5) | 246 (205–289) | 88·4 (82·1–93·7) | 218 (174–266) | 41·2 (34·5–48·4) | 89·7 (66·1–114) |  |
|  | Montenegro | 66·6 (57·8–76·3) | 20·1 (17–24·3) | 89·5 (82·9–94·4) | 18 (14·2–22·6) | 40·8 (34·6–48·1) | 7·33 (5·43–9·35) |  |
|  | North Macedonia | 60·9 (53·1–70·1) | 60·7 (51·3–72·3) | 83·0 (75·6–89·8) | 50·5 (40·7–63) | 40·3 (33·4–47·9) | 20·3 (15–26·7) |  |
|  | Poland | 75·5 (66·4–84·3) | 2520 (2080–2950) | 94·9 (91·3–97·6) | 2400 (1960–2880) | 51·8 (44·9–59·2) | 1240 (987–1520) |  |
|  | Romania | 61·4 (53·2–71·0) | 429 (362–506) | 84·3 (76·9–90·5) | 362 (288–455) | 39·7 (33·1–47·1) | 144 (107–190) |  |
|  | Serbia | 61·5 (53·0–71·4) | 303 (256–363) | 82·0 (73·5–89·4) | 249 (194–316) | 40·2 (33·5–47·2) | 99·8 (72·5–132) |  |
|  | Slovakia | 63·8 (55·7–72·3) | 108 (90·6–127) | 86·7 (79·9–92·3) | 93·5 (75·4–114) | 40·6 (34·0–47·7) | 37·9 (28·5–50·3) |  |
|  | Slovenia | 72·1 (62·5–82·5) | 62·8 (52·6–72·5) | 93·2 (88·1–96·8) | 58·6 (47·9–70·2) | 40·9 (34·2–48·3) | 23·9 (18–29·9) |  |
|  | Eastern Europe | 60·5 (52·5–68·9) | 3870 (3220–4560) | 90·2 (85·3–94·3) | 3490 (2850–4240) | 48·0 (41·2–55·5) | 1680 (1320–2100) |  |
|  | Belarus | 68·0 (60·6–75·5) | 230 (196–261) | 74·7 (65·6–83·9) | 172 (135–214) | 59·4 (52·5–66·9) | 102 (78·6–129) |  |
|  | Estonia | 62·9 (54·6–71·5) | 34 (28·2–39·9) | 87·7 (81·4–92·5) | 29·9 (23·7–37·1) | 47·9 (41·3–55·7) | 14·3 (10·6–18·3) |  |
|  | Latvia | 62·0 (53·7–70·8) | 52·9 (43·7–63·1) | 86·1 (78·8–91·8) | 45·7 (35·9–57·4) | 47·9 (41·1–55·3) | 21·8 (16·2–27·6) |  |
|  | Lithuania | 61·0 (53·9–68·7) | 56·2 (46·6–64·6) | 90·2 (85·1–93·8) | 50·7 (40·5–59·8) | 37·9 (32·0–43·7) | 19·2 (14·3–24) |  |
|  | Moldova | 54·2 (47·0–61·1) | 80·5 (68·2–92) | 74·0 (65·3–81·9) | 59·6 (47·5–72·7) | 43·9 (37·0–51·6) | 26·1 (19·3–32·9) |  |
|  | Russia | 60·6 (52·4–69·1) | 2510 (2070–3030) | 93·8 (90·2–96·6) | 2360 (1920–2890) | 48·0 (41·3–55·6) | 1130 (887–1420) |  |
|  | Ukraine | 58·4 (50·5–66·5) | 895 (740–1040) | 86·9 (80·8–92·1) | 779 (627–946) | 46·3 (39·7–53·8) | 360 (273–457) |  |
|  | **High income** | **73·3 (66·4–79·4)** | **42700 (38000–47400)** | **88·7 (83·8–92·9)** | **37900 (32400–43900)** | **41·8 (35·7–48·4)** | **15800 (12800–18900)** |  |
|  | Australasia | 64·3 (59·0–69·8) | 471 (424–522) | 80·5 (74·8–86·0) | 379 (326–445) | 29·4 (24·0–34·7) | 112 (86·7–136) |  |
|  | Australia | 64·1 (59·1–69·4) | 388 (348–433) | 80·9 (75·4–85·9) | 314 (271–366) | 29·5 (24·2–34·8) | 92·7 (72·8–114) |  |
|  | New Zealand | 65·1 (58·6–71·8) | 82·5 (72·3–94·1) | 78·7 (71·0–86·2) | 65·1 (52·9–79·7) | 29·0 (23·5–35·3) | 18·9 (14·1–24·1) |  |
|  | High-income Asia Pacific | 64·3 (57·1–71·8) | 8970 (7770–10300) | 92·3 (88·7–95·3) | 8290 (6940–9770) | 46·4 (40·4–53·0) | 3840 (3130–4690) |  |
|  | Brunei | 65·3 (58·2–73·9) | 8·94 (7·77–10·5) | 86·2 (80·7–91·7) | 7·72 (6·45–9·4) | 36·2 (30·0–43·1) | 2·79 (2·15–3·57) |  |
|  | Japan | 66·3 (57·6–75·4) | 7440 (6220–8750) | 94·0 (90·6–96·9) | 7000 (5710–8460) | 46·2 (40·0–53·6) | 3230 (2570–4030) |  |
|  | South Korea | 56·4 (53·6–59·5) | 1430 (1310–1540) | 82·7 (80·1–85·3) | 1180 (1080–1280) | 48·5 (44·9–52·4) | 574 (516–631) |  |
|  | Singapore | 56·6 (53·3–60·1) | 110 (101–120) | 89·0 (86·4–91·3) | 97·7 (88·7–108) | 34·2 (29·9–38·9) | 33·4 (28·2–39·3) |  |
|  | High-income North America | 78·6 (70·9–86·1) | 15200 (13400–17100) | 92·6 (88·3–95·9) | 14100 (12100–16200) | 43·9 (37·7–50·7) | 6190 (4980–7380) |  |
|  | Canada | 83·4 (76·4–90·1) | 911 (817–1010) | 95·9 (93·1–98·0) | 875 (773–991) | 44·5 (38·3–50·7) | 389 (321–456) |  |
|  | Greenland | 66·2 (59·0–73·9) | 0·753 (0·655–0·878) | 72·8 (64·5–81·0) | 0·549 (0·446–0·678) | 41·2 (35·1–48·3) | 0·226 (0·178–0·288) |  |
|  | USA | 78·3 (70·5–85·9) | 14300 (12500–16100) | 92·3 (88·0–95·8) | 13200 (11200–15300) | 43·8 (37·7–50·7) | 5800 (4650–6950) |  |
|  | Southern Latin America | 74·4 (67·4–81·6) | 1170 (1040–1310) | 87·7 (81·4–92·8) | 1030 (867–1190) | 61·4 (55·2–67·3) | 630 (530–739) |  |
|  | Argentina | 75·7 (67·9–83·8) | 918 (813–1030) | 89·1 (82·6–94·2) | 819 (686–954) | 62·8 (56·4–68·9) | 514 (425–612) |  |
|  | Chile | 72·3 (67·7–76·9) | 179 (163–196) | 81·3 (75·5–86·1) | 146 (126–165) | 52·7 (46·5–58·1) | 76·7 (64·9–88·6) |  |
|  | Uruguay | 65·5 (58·5–73·1) | 72·5 (62·3–82·8) | 86·0 (80·5–91·2) | 62·4 (51·2–74·8) | 63·5 (57·1–69·5) | 39·6 (31·2–49) |  |
|  | Western Europe | 74·0 (68·6–79·3) | 16700 (15100–18500) | 84·2 (78·3–89·1) | 14100 (12200–16200) | 36·2 (30·3–43·0) | 5090 (4070–6120) |  |
|  | Andorra | 76·6 (69·0–83·8) | 1·77 (1·53–2·05) | 92·1 (87·5–95·4) | 1·63 (1·38–1·94) | 34·7 (28·8–40·7) | 0·565 (0·439–0·715) |  |
|  | Austria | 74·9 (67·7–82·2) | 221 (196–248) | 88·9 (82·6–93·8) | 197 (167–231) | 35·5 (29·6–42·0) | 69·7 (53·8–87·1) |  |
|  | Belgium | 73·9 (66·7–81·4) | 339 (295–384) | 87·8 (81·7–92·8) | 298 (248–355) | 35·4 (29·3–41·9) | 105 (81·5–132) |  |
|  | Cyprus | 68·0 (61·1–74·6) | 33·5 (28·8–37·9) | 78·3 (70·8–85·3) | 26·3 (21·4–31·8) | 34·8 (28·7–41·2) | 9·14 (6·94–11·8) |  |
|  | Denmark | 70·9 (63·9–78·3) | 132 (116–150) | 83·6 (77·1–89·7) | 111 (90·2–135) | 34·8 (28·9–41·1) | 38·5 (29·5–47·9) |  |
|  | Finland | 83·0 (76·5–88·5) | 305 (271–340) | 92·7 (88·7–96·0) | 283 (243–325) | 35·1 (29·2–41·7) | 99·3 (79–119) |  |
|  | France | 64·0 (60·5–67·2) | 1860 (1670–2050) | 80·6 (76·3–84·5) | 1500 (1340–1680) | 35·3 (29·3–41·8) | 528 (429–637) |  |
|  | Germany | 74·9 (66·8–82·5) | 4040 (3520–4530) | 87·2 (80·8–92·6) | 3530 (2880–4170) | 36·2 (29·7–43·0) | 1280 (962–1600) |  |
|  | Greece | 75·1 (67·7–82·6) | 370 (323–425) | 88·5 (82·3–93·7) | 328 (269–395) | 36·0 (30·2–42·6) | 118 (90·5–150) |  |
|  | Iceland | 76·0 (68·9–83·5) | 6·77 (5·8–7·82) | 91·0 (86·0–95·0) | 6·17 (5·15–7·3) | 35·7 (29·6–41·9) | 2·2 (1·75–2·77) |  |
|  | Ireland | 70·3 (63·2–77·1) | 68·2 (59·4–78·3) | 84·3 (77·6–90·2) | 57·5 (47·6–69·8) | 35·3 (29·5–41·4) | 20·3 (15·6–25·6) |  |
|  | Israel | 71·1 (63·5–78·2) | 164 (144–184) | 82·9 (75·0–89·6) | 136 (111–164) | 36·4 (29·8–43·5) | 49·6 (37·9–62·7) |  |
|  | Italy | 77·3 (69·4–84·6) | 2630 (2250–3040) | 91·3 (86·6–95·3) | 2410 (1980–2870) | 35·3 (29·3–42·2) | 849 (661–1050) |  |
|  | Luxembourg | 69·6 (65·0–74·3) | 11·6 (10·4–12·9) | 85·1 (80·6–88·8) | 9·86 (8·68–11·2) | 35·1 (28·8–41·4) | 3·46 (2·76–4·28) |  |
|  | Malta | 72·7 (65·1–80·5) | 13·1 (11·3–15) | 85·9 (79·3–91·7) | 11·2 (9·28–13·5) | 35·1 (29·3–41·9) | 3·94 (3·01–4·94) |  |
|  | Monaco | 75·3 (68·4–81·9) | 1·18 (1·02–1·37) | 87·2 (80·9–92·2) | 1·03 (0·853–1·25) | 37·1 (31·1–44·1) | 0·382 (0·29–0·48) |  |
|  | Netherlands | 74·6 (67·1–82·1) | 521 (455–596) | 88·3 (82·4–93·2) | 461 (380–547) | 35·4 (29·2–42·1) | 163 (127–201) |  |
|  | Norway | 77·3 (69·2–84·1) | 199 (170–228) | 96·2 (93·3–98·0) | 192 (162–223) | 37·0 (31·0–43·1) | 70·8 (56–87) |  |
|  | Portugal | 84·1 (82·5–86·0) | 542 (504–587) | 91·6 (90·0–93·2) | 497 (464–537) | 35·4 (29·1–41·7) | 176 (141–210) |  |
|  | San Marino | 78·9 (71·0–86·6) | 0·862 (0·73–0·982) | 92·7 (88·3–96·1) | 0·8 (0·672–0·936) | 36·4 (30·2–42·7) | 0·291 (0·228–0·36) |  |
|  | Spain | 80·3 (77·4–83·3) | 2320 (2120–2540) | 61·6 (55·3–67·8) | 1430 (1230–1670) | 36·8 (30·4–43·3) | 526 (416–651) |  |
|  | Sweden | 76·7 (69·2–84·4) | 386 (341–432) | 91·1 (86·7–95·2) | 352 (298–408) | 35·8 (29·6–41·9) | 126 (99·5–155) |  |
|  | Switzerland | 64·0 (59·6–67·9) | 226 (203–251) | 82·3 (78·2–86·3) | 186 (162–213) | 40·7 (36·4–45·7) | 75·8 (63·4–88·7) |  |
|  | UK | 71·0 (63·6–77·7) | 2360 (2050–2660) | 87·4 (82·1–91·9) | 2070 (1710–2420) | 37·8 (31·9–44·5) | 781 (617–970) |  |
|  | **Latin America and Caribbean** | **52·0 (45·5–59·1)** | **10900 (9540–12800)** | **88·5 (84·1–92·4)** | **9680 (8030–11700)** | **38·1 (32·5–44·7)** | **3690 (2870–4540)** |  |
|  | Andean Latin America | 50·2 (44·5–56·1) | 523 (459–591) | 73·5 (66·3–80·5) | 385 (317–461) | 39·0 (33·3–45·7) | 150 (114–189) |  |
|  | Bolivia | 45·7 (40·0–51·1) | 98·2 (83·7–114) | 63·1 (54·8–71·8) | 62 (49·5–76·3) | 37·6 (31·4–44·7) | 23·3 (17·2–30) |  |
|  | Ecuador | 53·0 (45·2–61·4) | 211 (174–248) | 89·1 (84·0–93·5) | 189 (150–229) | 40·5 (34·3–47·3) | 76·2 (55·6–96·9) |  |
|  | Peru | 50·2 (46·0–55·0) | 216 (186–244) | 62·4 (55·9–69·0) | 135 (114–161) | 38·0 (32·4–44·2) | 51·2 (39·8–63·5) |  |
|  | Caribbean | 59·1 (53·0–65·0) | 1250 (1110–1400) | 89·9 (85·9–93·2) | 1130 (960–1300) | 38·0 (32·2–44·6) | 427 (340–512) |  |
|  | Antigua and Barbuda | 61·6 (54·8–69·4) | 3·33 (2·85–3·84) | 92·4 (88·9–95·2) | 3·08 (2·59–3·65) | 37·6 (31·5–44·3) | 1·16 (0·922–1·39) |  |
|  | The Bahamas | 55·5 (49·0–63·1) | 9·9 (8·54–11·5) | 88·3 (83·4–92·3) | 8·76 (7·31–10·5) | 36·9 (30·8–43·6) | 3·23 (2·51–4·06) |  |
|  | Barbados | 65·3 (58·3–72·0) | 18·5 (16·1–20·7) | 91·1 (87·4–94·1) | 16·9 (14·4–19·4) | 39·5 (32·7–46·1) | 6·66 (5·3–8) |  |
|  | Belize | 52·1 (46·8–58·0) | 6·01 (5·21–6·8) | 81·8 (75·8–87·5) | 4·92 (4·13–5·8) | 39·3 (33·1–46·1) | 1·93 (1·52–2·39) |  |
|  | Bermuda | 73·2 (66·6–80·1) | 2·87 (2·55–3·22) | 96·9 (95·3–98·3) | 2·79 (2·45–3·15) | 39·8 (34·1–46·5) | 1·11 (0·886–1·31) |  |
|  | Cuba | 65·1 (58·6–71·7) | 309 (269–350) | 95·3 (92·8–97·0) | 295 (253–335) | 37·7 (32·0–44·2) | 111 (88·7–135) |  |
|  | Dominica | 59·0 (51·8–65·9) | 3·3 (2·88–3·75) | 85·9 (80·2–90·8) | 2·83 (2·38–3·38) | 39·5 (33·3–46·7) | 1·12 (0·869–1·38) |  |
|  | Dominican Republic | 52·3 (46·2–59·1) | 182 (155–209) | 86·7 (81·9–90·9) | 158 (130–190) | 36·5 (31·0–43·0) | 57·6 (45·4–71·7) |  |
|  | Grenada | 59·9 (52·9–67·0) | 5·58 (4·8–6·43) | 89·6 (84·8–93·4) | 5 (4·18–5·9) | 38·3 (31·9–45·5) | 1·91 (1·47–2·37) |  |
|  | Guyana | 61·3 (54·4–69·8) | 31·6 (27·3–36) | 88·3 (83·0–92·7) | 27·9 (23·6–33·3) | 35·1 (29·2–41·7) | 9·79 (7·68–12·2) |  |
|  | Haiti | 44·3 (39·0–50·4) | 132 (110–155) | 74·7 (66·8–81·5) | 98·4 (78·3–124) | 33·2 (27·1–39·6) | 32·6 (24·5–41·9) |  |
|  | Jamaica | 60·4 (53·4–67·7) | 95·8 (84·1–109) | 90·9 (86·6–94·2) | 87·2 (73–101) | 38·4 (32·5–44·9) | 33·4 (26·5–40·9) |  |
|  | Puerto Rico | 69·4 (62·6–76·7) | 274 (239–305) | 94·2 (91·1–96·5) | 258 (221–295) | 40·5 (34·5–47·1) | 104 (83·5–124) |  |
|  | Saint Kitts and Nevis | 56·2 (49·5–62·1) | 1·76 (1·52–2·02) | 86·6 (82·0–90·7) | 1·52 (1·29–1·81) | 38·3 (31·9–44·7) | 0·584 (0·46–0·708) |  |
|  | Saint Lucia | 48·9 (42·2–55·8) | 6·55 (5·31–7·7) | 84·8 (78·6–89·7) | 5·56 (4·33–6·85) | 39·5 (33·0–47·0) | 2·2 (1·67–2·82) |  |
|  | Saint Vincent and the Grenadines | 58·1 (51·4–65·7) | 5·38 (4·59–6·15) | 89·2 (84·3–93·2) | 4·81 (3·98–5·72) | 37·4 (31·6–44·0) | 1·8 (1·38–2·24) |  |
|  | Suriname | 51·0 (44·8–58·0) | 14·9 (12·7–17·6) | 83·8 (77·8–89·3) | 12·5 (10·3–15·4) | 35·7 (29·8–42·2) | 4·45 (3·47–5·72) |  |
|  | Trinidad and Tobago | 59·0 (52·3–66·7) | 102 (85·6–118) | 86·7 (81·2–91·4) | 88·5 (71·2–107) | 38·2 (32·1–45·6) | 33·8 (25·8–42·1) |  |
|  | Virgin Islands | 62·1 (54·3–70·6) | 5·35 (4·55–6·26) | 88·8 (83·7–93·1) | 4·75 (3·93–5·69) | 39·1 (32·2–46·4) | 1·86 (1·45–2·36) |  |
|  | Central Latin America | 50·3 (43·8–57·6) | 5750 (5020–6750) | 90·0 (86·1–93·7) | 5180 (4300–6220) | 35·4 (29·8–42·0) | 1830 (1420–2300) |  |
|  | Colombia | 54·6 (48·1–61·6) | 731 (627–834) | 92·6 (89·6–95·3) | 678 (567–784) | 48·2 (42·1–54·3) | 327 (259–393) |  |
|  | Costa Rica | 57·1 (52·0–63·3) | 102 (89–118) | 92·3 (89·3–94·7) | 94·1 (80·8–110) | 40·3 (34·3–45·6) | 37·9 (31·5–46·2) |  |
|  | El Salvador | 50·2 (43·3–58·0) | 117 (99–137) | 87·7 (83·0–92·1) | 103 (84·7–124) | 41·9 (35·4–48·7) | 42·9 (33·9–53·2) |  |
|  | Guatemala | 41·4 (36·1–47·4) | 159 (135–184) | 78·3 (71·7–84·3) | 124 (101–151) | 40·6 (34·6–47·7) | 50·5 (38·4–64·5) |  |
|  | Honduras | 41·8 (36·2–47·6) | 68 (56·1–80·5) | 80·4 (74·4–86·4) | 54·7 (44·9–67·3) | 39·2 (33·3–46·1) | 21·4 (16·6–27) |  |
|  | Mexico | 49·9 (43·0–57·9) | 3960 (3400–4710) | 90·0 (85·9–93·8) | 3570 (2930–4340) | 31·2 (25·8–37·4) | 1110 (846–1420) |  |
|  | Nicaragua | 48·1 (42·2–55·9) | 57 (48·4–67·5) | 88·0 (83·2–92·1) | 50·3 (41·3–61) | 41·0 (35·0–48·1) | 20·6 (15·7–25·9) |  |
|  | Panama | 57·5 (50·4–65·8) | 70·5 (59·6–82·1) | 94·3 (91·6–96·7) | 66·5 (55–78·7) | 40·5 (34·1–47·1) | 26·9 (20·7–33·4) |  |
|  | Venezuela | 50·8 (43·8–58·7) | 482 (402–566) | 90·1 (86·2–93·7) | 435 (352–525) | 41·6 (35·0–48·4) | 181 (139–222) |  |
|  | Tropical Latin America | 53·2 (46·4–60·7) | 3420 (2920–4070) | 87·4 (82·3–92·0) | 2990 (2480–3690) | 41·7 (35·7–48·7) | 1250 (960–1560) |  |
|  | Brazil | 53·1 (46·4–60·6) | 3320 (2830–3950) | 87·4 (82·3–92·0) | 2900 (2400–3590) | 41·7 (35·7–48·7) | 1210 (932–1520) |  |
|  | Paraguay | 56·5 (49·2–64·0) | 102 (87·4–121) | 87·4 (82·1–92·5) | 89·5 (73–110) | 41·9 (35·0–48·9) | 37·5 (28·9–47·8) |  |
|  | **North Africa and Middle East** | **56·4 (50·7–62·2)** | **10700 (9370–12300)** | **88·4 (84·7–91·8)** | **9460 (8190–11200)** | **42·0 (35·6–48·8)** | **3980 (3180–4900)** |  |
|  | Afghanistan | 37·6 (32·2–43·9) | 245 (204–298) | 88·9 (84·3–92·7) | 218 (177–271) | 43·0 (36·6–50·4) | 93·6 (71·9–119) |  |
|  | Algeria | 50·1 (44·3–56·3) | 689 (580–806) | 89·7 (86·1–93·0) | 618 (514–744) | 44·1 (38·4–50·6) | 273 (217–338) |  |
|  | Bahrain | 54·9 (47·0–64·2) | 29·5 (24·6–34·9) | 88·6 (83·7–92·9) | 26·2 (20·8–32·2) | 41·4 (34·7–49·2) | 10·8 (8·21–14·3) |  |
|  | Egypt | 64·7 (60·5–69·0) | 2220 (2000–2450) | 90·0 (86·8–92·4) | 2000 (1750–2240) | 40·6 (33·5–48·4) | 811 (655–991) |  |
|  | Iran | 62·7 (56·2–68·8) | 1440 (1250–1670) | 81·9 (75·4–88·3) | 1190 (990–1460) | 47·1 (40·3–54·1) | 558 (433–712) |  |
|  | Iraq | 46·1 (40·0–52·9) | 529 (448–625) | 84·4 (78·5–89·6) | 448 (358–550) | 34·4 (28·4–40·7) | 154 (114–199) |  |
|  | Jordan | 62·0 (57·4–67·1) | 154 (138–168) | 84·7 (80·2–88·8) | 131 (115–147) | 36·2 (31·4–41·8) | 47·3 (39·3–56·4) |  |
|  | Kuwait | 67·9 (59·3–76·5) | 102 (86·6–119) | 93·4 (89·5–96·4) | 95 (78·4–114) | 26·2 (21·8–32·1) | 24·9 (19·1–33·5) |  |
|  | Lebanon | 43·4 (37·3–50·6) | 108 (88·7–132) | 88·9 (83·0–93·2) | 96·5 (78·3–120) | 48·7 (41·5–55·9) | 46·9 (36·4–59·3) |  |
|  | Libya | 52·1 (46·7–57·3) | 132 (115–150) | 88·1 (84·3–91·5) | 117 (100–134) | 37·1 (32·1–42·7) | 43·3 (34·5–54·1) |  |
|  | Morocco | 46·2 (40·5–53·1) | 634 (526–763) | 82·4 (76·2–87·8) | 523 (420–656) | 34·0 (28·1–40·6) | 178 (133–234) |  |
|  | Oman | 56·7 (49·1–65·5) | 50·2 (43·2–60·1) | 92·0 (88·3–95·4) | 46·2 (38·4–56·3) | 40·5 (34·1–47·9) | 18·7 (14–24·1) |  |
|  | Palestine | 60·6 (56·1–65·3) | 75 (66·8–82·7) | 90·8 (87·9–93·5) | 68·1 (59·8–76) | 29·3 (25·1–33·8) | 19·9 (16·3–24·3) |  |
|  | Qatar | 64·7 (56·0–74·0) | 33·4 (27·8–40) | 87·8 (81·1–93·6) | 29·4 (23–36·3) | 48·5 (41·4–56·8) | 14·3 (10·6–19) |  |
|  | Saudi Arabia | 58·3 (52·4–64·6) | 1020 (900–1170) | 97·2 (95·8–98·1) | 995 (870–1140) | 44·4 (36·3–51·8) | 442 (339–552) |  |
|  | Sudan | 50·3 (43·5–57·4) | 422 (346–510) | 85·0 (79·1–90·1) | 359 (290–451) | 26·4 (21·5–32·0) | 94·8 (71–122) |  |
|  | Syria | 55·7 (49·2–63·9) | 371 (314–447) | 90·8 (86·6–94·6) | 337 (282–418) | 40·7 (33·9–47·8) | 137 (106–178) |  |
|  | Tunisia | 64·5 (56·4–73·1) | 363 (307–424) | 94·6 (91·5–97·0) | 343 (282–410) | 39·6 (33·3–46·4) | 136 (105–170) |  |
|  | Türkiye | 57·5 (50·8–65·9) | 1800 (1550–2120) | 87·8 (82·6–92·5) | 1590 (1320–1930) | 46·2 (39·1–53·3) | 732 (583–916) |  |
|  | United Arab Emirates | 60·1 (50·3–69·8) | 140 (114–172) | 93·0 (88·3–96·5) | 130 (101–166) | 56·4 (49·0–64·5) | 73·3 (54·8–96·7) |  |
|  | Yemen | 48·1 (41·6–55·4) | 124 (105–151) | 86·1 (80·7–91·0) | 107 (86·7–134) | 37·1 (30·7–44·4) | 39·8 (29·8–51·3) |  |
|  | **South Asia** | **31·8 (27·0–37·5)** | **16700 (14200–20400)** | **89·4 (85·5–92·5)** | **15000 (12200–18700)** | **34·5 (28·9–41·0)** | **5160 (3900–6550)** |  |
|  | Bangladesh | 45·5 (40·5–50·5) | 1850 (1570–2180) | 86·6 (81·9–90·6) | 1600 (1320–1910) | 45·6 (38·5–53·5) | 731 (564–921) |  |
|  | Bhutan | 38·5 (33·7–43·3) | 7·16 (6·14–8·24) | 79·9 (74·1–84·9) | 5·73 (4·74–6·86) | 56·3 (50·0–62·6) | 3·22 (2·55–3·94) |  |
|  | India | 29·6 (24·9–35·4) | 12400 (10300–15400) | 92·1 (88·9–94·5) | 11400 (9230–14500) | 32·1 (26·4–38·8) | 3670 (2720–4700) |  |
|  | Nepal | 36·2 (31·2–41·8) | 286 (237–341) | 72·6 (64·5–79·7) | 208 (167–261) | 30·1 (24·3–36·7) | 62·5 (45·9–80·6) |  |
|  | Pakistan | 36·7 (31·7–42·2) | 2150 (1800–2550) | 79·8 (73·8–85·2) | 1720 (1400–2110) | 42·5 (36·2–49·4) | 730 (559–929) |  |
|  | **Southeast Asia, east Asia, and Oceania** | **37·2 (32·7–42·2)** | **34400 (29300–40000)** | **72·3 (65·6–78·6)** | **24900 (20300–30700)** | **38·9 (33·4–45·4)** | **9670 (7470–12200)** |  |
|  | East Asia | 35·9 (31·5–40·9) | 26800 (22700–31300) | 69·3 (62·2–76·0) | 18600 (15000–23000) | 36·9 (31·2–43·8) | 6860 (5230–8710) |  |
|  | China | 35·8 (31·3–40·6) | 25800 (21800–30100) | 68·8 (61·7–75·6) | 17800 (14300–22100) | 36·9 (31·2–43·8) | 6570 (5000–8350) |  |
|  | North Korea | 35·6 (30·8–40·5) | 363 (302–435) | 68·5 (60·8–76·0) | 249 (194–318) | 35·4 (29·8–42·4) | 88·2 (66·2–115) |  |
|  | Taiwan | 45·7 (39·3–53·2) | 620 (526–723) | 88·3 (83·1–92·2) | 548 (447–662) | 37·7 (31·6–44·9) | 206 (160–260) |  |
|  | Oceania | 42·3 (37·4–47·8) | 180 (155–210) | 80·2 (74·8–85·7) | 145 (122–174) | 23·8 (19·3–29·4) | 34·3 (26·2–44·2) |  |
|  | American Samoa | 43·5 (38·4–48·9) | 2·57 (2·23–2·93) | 84·9 (80·5–88·9) | 2·19 (1·86–2·6) | 32·2 (27·2–38·3) | 0·704 (0·558–0·895) |  |
|  | Cook Islands | 38·0 (34·1–42·3) | 0·972 (0·837–1·09) | 78·2 (72·5–83·3) | 0·76 (0·645–0·902) | 31·6 (26·4–37·3) | 0·24 (0·19–0·301) |  |
|  | Fiji | 56·6 (51·9–61·8) | 42·3 (37·3–47·4) | 82·2 (77·3–87·4) | 34·8 (30·2–40·4) | 26·8 (21·9–32·5) | 9·32 (7·04–11·8) |  |
|  | Guam | 53·5 (46·1–61·7) | 4·56 (3·9–5·37) | 92·8 (89·4–95·6) | 4·24 (3·52–5·1) | 24·4 (19·5–29·7) | 1·03 (0·777–1·33) |  |
|  | Kiribati | 38·9 (35·0–43·3) | 2·52 (2·17–2·87) | 70·5 (63·8–76·4) | 1·78 (1·47–2·11) | 23·0 (18·7–28·3) | 0·409 (0·31–0·518) |  |
|  | Marshall Islands | 37·0 (32·6–41·3) | 1·58 (1·35–1·82) | 65·1 (58·5–72·0) | 1·03 (0·844–1·24) | 16·1 (12·8–20·6) | 0·166 (0·125–0·219) |  |
|  | Federated States of Micronesia | 33·6 (30·5–36·6) | 2·88 (2·59–3·22) | 67·4 (61·8–72·8) | 1·94 (1·67–2·23) | 20·0 (15·9–25·0) | 0·389 (0·288–0·495) |  |
|  | Nauru | 56·1 (50·7–61·4) | 0·459 (0·4–0·522) | 59·8 (52·8–67·1) | 0·275 (0·233–0·331) | 38·9 (31·9–45·5) | 0·107 (0·0799–0·133) |  |
|  | Niue | 56·5 (49·6–63·8) | 0·146 (0·124–0·167) | 84·3 (78·0–90·0) | 0·123 (0·0992–0·148) | 23·2 (18·7–28·6) | 0·0285 (0·0204–0·0369) |  |
|  | Northern Mariana Islands | 41·8 (36·0–49·5) | 2·12 (1·75–2·54) | 86·2 (80·7–91·4) | 1·83 (1·46–2·29) | 24·2 (19·6–30·4) | 0·441 (0·319–0·591) |  |
|  | Palau | 47·2 (40·9–53·9) | 0·817 (0·684–0·972) | 79·9 (73·8–86·4) | 0·654 (0·524–0·808) | 20·2 (15·9–25·1) | 0·132 (0·0973–0·171) |  |
|  | Papua New Guinea | 38·9 (33·4–44·8) | 91·7 (76·5–111) | 82·4 (76·9–87·9) | 75·7 (61·4–95·3) | 21·3 (17·0–26·6) | 16·1 (11·5–21·1) |  |
|  | Samoa | 38·6 (34·6–42·8) | 4·49 (3·86–5·1) | 74·8 (69·2–80·4) | 3·36 (2·83–3·93) | 27·5 (22·6–33·1) | 0·923 (0·706–1·18) |  |
|  | Solomon Islands | 35·8 (31·7–40·7) | 6·21 (5·33–7·21) | 46·4 (39·7–53·9) | 2·88 (2·36–3·5) | 25·8 (20·8–32·2) | 0·742 (0·557–0·948) |  |
|  | Tokelau | 59·0 (52·7–64·6) | 0·11 (0·0955–0·124) | 75·1 (67·9–82·2) | 0·0825 (0·066–0·1) | 16·2 (12·9–20·3) | 0·0134 (0·00953–0·0174) |  |
|  | Tonga | 47·4 (41·3–53·9) | 3·48 (2·98–4·03) | 78·3 (71·2–84·8) | 2·73 (2·18–3·35) | 19·6 (15·8–24·6) | 0·536 (0·387–0·716) |  |
|  | Tuvalu | 55·8 (48·7–63·5) | 0·361 (0·302–0·416) | 79·5 (72·6–86·7) | 0·287 (0·233–0·346) | 22·9 (18·3–28·1) | 0·0657 (0·0477–0·0862) |  |
|  | Vanuatu | 23·1 (19·9–26·7) | 2·08 (1·72–2·45) | 75·6 (69·0–81·7) | 1·58 (1·27–1·96) | 41·8 (35·3–49·2) | 0·659 (0·499–0·845) |  |
|  | Southeast Asia | 42·6 (38·0–48·0) | 7400 (6380–8620) | 83·3 (78·2–87·8) | 6160 (5230–7470) | 44·5 (39·3–50·7) | 2740 (2200–3350) |  |
|  | Cambodia | 51·8 (46·7–57·9) | 107 (90·4–122) | 77·2 (71·1–83·4) | 82·4 (68·1–98·7) | 50·2 (43·7–57·1) | 41·4 (33·2–51·4) |  |
|  | Indonesia | 39·2 (34·0–45·2) | 2400 (1990–2880) | 80·3 (73·9–86·0) | 1930 (1580–2420) | 41·5 (35·2–48·4) | 801 (611–1020) |  |
|  | Laos | 46·6 (41·1–52·1) | 60·8 (50·3–70·7) | 69·8 (61·9–77·4) | 42·5 (34·4–53·1) | 38·1 (31·9–44·7) | 16·2 (12·3–20·6) |  |
|  | Malaysia | 35·6 (33·2–38·6) | 432 (379–477) | 93·7 (92·5–94·8) | 405 (354–450) | 51·3 (47·6–55·7) | 208 (181–239) |  |
|  | Maldives | 37·3 (32·4–42·9) | 2·43 (2·03–2·89) | 82·4 (76·4–87·4) | 2 (1·64–2·47) | 43·2 (36·8–49·8) | 0·864 (0·67–1·08) |  |
|  | Mauritius | 51·7 (44·4–59·7) | 64·2 (54·9–75·5) | 90·3 (86·0–93·9) | 58·1 (47·7–70·6) | 44·3 (37·7–51·7) | 25·7 (20·3–32·4) |  |
|  | Myanmar | 45·5 (39·7–51·5) | 809 (690–941) | 75·4 (67·8–82·1) | 610 (495–757) | 42·7 (36·9–49·5) | 261 (200–330) |  |
|  | Philippines | 42·7 (36·9–49·5) | 1060 (890–1280) | 85·8 (80·7–90·5) | 910 (740–1130) | 42·2 (35·9–49·1) | 384 (294–489) |  |
|  | Seychelles | 52·7 (46·9–58·6) | 2·36 (2·02–2·71) | 93·6 (90·7–95·7) | 2·21 (1·87–2·57) | 18·5 (15·0–23·2) | 0·41 (0·304–0·539) |  |
|  | Sri Lanka | 59·1 (53·2–65·4) | 615 (535–702) | 76·3 (68·8–83·7) | 470 (378–580) | 50·8 (44·2–57·9) | 238 (188–299) |  |
|  | Thailand | 43·4 (39·7–47·7) | 1070 (932–1210) | 94·7 (93·4–95·9) | 1010 (877–1150) | 34·6 (30·6–39·2) | 351 (292–414) |  |
|  | Timor-Leste | 22·3 (18·4–26·7) | 3·4 (2·73–4·2) | 71·3 (61·9–79·6) | 2·43 (1·88–3·13) | 44·0 (34·8–53·1) | 1·07 (0·762–1·45) |  |
|  | Viet Nam | 42·5 (38·0–47·1) | 744 (634–865) | 84·2 (79·3–88·9) | 626 (526–760) | 63·8 (57·9–69·5) | 400 (321–496) |  |
|  | **Sub-Saharan Africa** | **33·3 (28·9–37·3)** | **4510 (3770–5220)** | **75·5 (68·8–81·1)** | **3410 (2800–4150)** | **47·4 (40·9–54·3)** | **1610 (1280–2030)** |  |
|  | Central sub-Saharan Africa | 15·7 (13·2–18·1) | 338 (279–395) | 57·5 (49·5–64·6) | 194 (157–237) | 49·6 (42·4–56·7) | 96·2 (74–121) |  |
|  | Angola | 18·5 (15·9–21·2) | 83·8 (70·2–98·6) | 49·4 (42·3–56·4) | 41·3 (33·4–51·2) | 58·0 (50·5–64·9) | 24 (19–30·4) |  |
|  | Central African Republic | 14·6 (12·5–16·8) | 15·7 (13–18·3) | 73·4 (66·6–78·8) | 11·5 (9·46–14) | 51·8 (44·9–58·4) | 5·97 (4·8–7·5) |  |
|  | Congo (Brazzaville) | 16·3 (13·9–18·6) | 18·3 (15·1–21·2) | 60·7 (52·6–67·6) | 11·1 (8·9–13·8) | 53·0 (46·5–59·8) | 5·9 (4·63–7·35) |  |
|  | DR Congo | 14·7 (12·2–17·1) | 206 (169–245) | 59·3 (51·4–66·7) | 122 (97·5–148) | 46·6 (39·6–53·8) | 57 (42·6–71·7) |  |
|  | Equatorial Guinea | 16·0 (13·6–18·2) | 3·04 (2·5–3·59) | 63·6 (55·3–70·4) | 1·94 (1·58–2·41) | 53·0 (46·2–60·0) | 1·03 (0·806–1·3) |  |
|  | Gabon | 18·2 (15·6–20·5) | 9·58 (8·14–11·1) | 62·9 (55·2–69·8) | 6·03 (4·87–7·38) | 55·1 (48·5–61·8) | 3·32 (2·6–4·18) |  |
|  | Eastern sub-Saharan Africa | 35·8 (31·3–40·1) | 1310 (1100–1500) | 72·1 (64·9–78·1) | 948 (774–1160) | 54·2 (47·5–60·7) | 514 (409–638) |  |
|  | Burundi | 36·7 (31·7–41·1) | 34·9 (28·8–40·3) | 76·1 (69·4–81·9) | 26·6 (21·4–32·6) | 53·3 (46·7–59·8) | 14·2 (11–18) |  |
|  | Comoros | 47·6 (41·3–54·3) | 5·41 (4·57–6·35) | 86·9 (81·2–91·2) | 4·71 (3·85–5·73) | 43·1 (36·6–49·8) | 2·03 (1·56–2·53) |  |
|  | Djibouti | 35·3 (30·8–40·6) | 3·35 (2·78–3·97) | 78·1 (71·8–83·7) | 2·62 (2·13–3·24) | 53·0 (45·8–59·4) | 1·39 (1·07–1·74) |  |
|  | Eritrea | 51·0 (45·2–56·2) | 32·3 (28·2–36·8) | 88·5 (83·8–92·6) | 28·6 (24·4–33·5) | 52·0 (45·9–57·7) | 14·9 (12·1–17·8) |  |
|  | Ethiopia | 23·3 (19·9–26·4) | 257 (212–298) | 50·6 (42·7–57·6) | 130 (103–162) | 53·5 (47·0–59·9) | 69·4 (53·8–88) |  |
|  | Kenya | 46·0 (40·2–51·7) | 214 (180–250) | 86·8 (81·9–91·0) | 186 (152–225) | 57·3 (51·0–63·8) | 106 (83·9–131) |  |
|  | Madagascar | 36·2 (31·2–40·8) | 78 (65·3–91·7) | 79·9 (73·6–85·4) | 62·4 (50·2–76·8) | 52·7 (46·4–59·3) | 32·9 (25·2–41) |  |
|  | Malawi | 45·6 (39·5–51·2) | 58·1 (48·3–66·8) | 70·9 (62·0–78·4) | 41·3 (33·1–50·6) | 60·8 (52·7–68·1) | 25·1 (19·4–31·7) |  |
|  | Mozambique | 43·4 (36·2–50·4) | 91·6 (76–108) | 84·6 (78·5–89·6) | 77·6 (61·5–95·6) | 65·8 (54·5–75·1) | 51·1 (38·2–66·5) |  |
|  | Rwanda | 28·5 (24·3–32·6) | 30·6 (25·5–35·9) | 68·6 (60·7–75·5) | 21 (16·8–25·9) | 64·3 (53·5–73·0) | 13·5 (10·3–17·1) |  |
|  | Somalia | 35·6 (30·8–40·4) | 52·4 (43·4–60·9) | 70·0 (63·0–77·1) | 36·7 (29·8–45·5) | 53·5 (46·9–60·7) | 19·7 (15·6–24·8) |  |
|  | South Sudan | 35·8 (31·2–40·2) | 34 (28·4–38·9) | 77·6 (70·8–83·2) | 26·4 (21·3–31·9) | 53·7 (47·2–60·1) | 14·2 (11·2–17·6) |  |
|  | Uganda | 43·1 (37·8–48·2) | 166 (139–192) | 75·2 (68·0–81·6) | 125 (101–153) | 67·1 (61·3–72·3) | 83·8 (67·1–104) |  |
|  | Tanzania | 45·6 (40·2–50·8) | 205 (172–233) | 71·6 (64·3–78·5) | 147 (119–179) | 41·6 (35·6–47·9) | 61·2 (48·3–76·2) |  |
|  | Zambia | 26·3 (22·8–29·8) | 48·3 (40·7–56·3) | 66·8 (60·1–73·2) | 32·3 (26·5–39·1) | 31·7 (26·4–37·9) | 10·2 (7·91–13·1) |  |
|  | Southern sub-Saharan Africa | 52·0 (44·8–58·9) | 1210 (1020–1420) | 89·8 (85·6–93·7) | 1090 (894–1310) | 38·6 (32·7–45·5) | 419 (325–531) |  |
|  | Botswana | 44·8 (38·4–51·1) | 20·6 (17·1–23·9) | 91·1 (86·9–94·3) | 18·7 (15·3–22·6) | 50·2 (42·9–57·3) | 9·4 (7·55–11·7) |  |
|  | Eswatini | 41·1 (35·4–47·1) | 11·4 (9·57–13·4) | 93·4 (90·3–95·6) | 10·7 (8·88–12·7) | 50·4 (44·4–56·8) | 5·39 (4·23–6·69) |  |
|  | Lesotho | 58·3 (51·1–65·0) | 23·7 (20·3–27·6) | 93·7 (90·5–96·0) | 22·2 (18·8–26·4) | 44·8 (38·1–51·9) | 9·94 (7·83–12·3) |  |
|  | Namibia | 49·8 (42·5–57·1) | 22·5 (18·7–26·6) | 93·8 (90·7–96·2) | 21·2 (17·2–25·5) | 35·6 (29·5–42·0) | 7·52 (5·69–9·46) |  |
|  | South Africa | 52·5 (45·2–59·5) | 1020 (860–1190) | 89·0 (84·3–93·2) | 906 (745–1100) | 37·6 (31·7–44·6) | 340 (261–433) |  |
|  | Zimbabwe | 49·6 (43·2–57·1) | 114 (96–135) | 95·1 (92·7–97·0) | 109 (90·3–131) | 42·2 (35·9–48·7) | 45·8 (35·9–56·4) |  |
|  | Western sub-Saharan Africa | 30·4 (26·2–34·0) | 1630 (1390–1890) | 72·0 (64·9–78·0) | 1180 (968–1430) | 47·0 (40·6–53·7) | 553 (436–700) |  |
|  | Benin | 25·8 (22·4–29·0) | 30·9 (26·4–35·6) | 78·8 (73·0–83·4) | 24·4 (20·4–29·1) | 51·7 (45·4–57·4) | 12·6 (10·1–15·5) |  |
|  | Burkina Faso | 30·0 (25·6–33·9) | 47·5 (40·2–55·6) | 74·3 (66·5–80·5) | 35·4 (28·3–43·1) | 47·2 (40·6–54·2) | 16·7 (12·9–21·5) |  |
|  | Cabo Verde | 47·3 (41·7–52·5) | 6·56 (5·53–7·5) | 86·3 (81·0–90·6) | 5·67 (4·68–6·78) | 39·6 (33·6–46·1) | 2·24 (1·77–2·78) |  |
|  | Cameroon | 40·3 (36·0–45·1) | 115 (99·5–132) | 82·6 (77·2–87·3) | 95·2 (79·9–113) | 54·2 (48·5–60·0) | 51·6 (42·2–63·2) |  |
|  | Chad | 27·1 (23·3–30·7) | 47·8 (40–56·6) | 69·3 (61·4–75·9) | 33·1 (26·9–41·8) | 45·7 (39·4–52·5) | 15·1 (11·8–19·3) |  |
|  | Côte d'Ivoire | 25·9 (22·6–29·1) | 99·1 (82·6–116) | 68·0 (60·6–74·6) | 67·4 (55·1–83) | 46·3 (40·0–52·8) | 31·2 (24·7–39·1) |  |
|  | The Gambia | 26·9 (23·3–30·2) | 10·2 (8·47–11·7) | 71·6 (64·4–77·8) | 7·3 (6·01–9·04) | 47·0 (40·5–53·6) | 3·43 (2·67–4·38) |  |
|  | Ghana | 48·7 (43·3–53·6) | 235 (203–266) | 73·3 (65·8–80·1) | 172 (143–208) | 43·0 (37·0–49·6) | 74·1 (57·8–92·7) |  |
|  | Guinea | 36·7 (31·8–41·2) | 62 (53·2–71) | 68·6 (61·0–75·2) | 42·6 (34·9–52·4) | 55·5 (47·3–63·4) | 23·7 (18·5–30·4) |  |
|  | Guinea-Bissau | 26·4 (22·8–29·6) | 8·02 (6·82–9·14) | 60·3 (52·9–67·4) | 4·84 (3·92–5·85) | 46·3 (39·9–52·8) | 2·24 (1·74–2·84) |  |
|  | Liberia | 30·5 (26·0–35·1) | 22·6 (18·7–26·5) | 77·9 (70·5–83·8) | 17·6 (14·2–21·9) | 49·7 (42·1–57·5) | 8·76 (6·89–11·3) |  |
|  | Mali | 21·6 (18·6–24·2) | 100 (83–118) | 68·2 (60·4–74·7) | 68·4 (56·1–86) | 38·6 (31·7–45·5) | 26·4 (20–34·5) |  |
|  | Mauritania | 25·1 (21·1–29·1) | 19·7 (16·1–23) | 64·0 (55·3–72·4) | 12·6 (9·82–16) | 50·6 (42·6–58·4) | 6·39 (4·61–8·47) |  |
|  | Niger | 10·1 (7·9–12·3) | 21 (16–25·9) | 57·5 (46·7–68·3) | 12·1 (9·37–15·5) | 43·3 (34·9–51·5) | 5·21 (3·8–6·97) |  |
|  | Nigeria | 30·3 (26·0–34·0) | 648 (539–753) | 72·0 (64·5–78·2) | 467 (378–573) | 47·3 (40·8–54·2) | 221 (170–283) |  |
|  | São Tomé and Príncipe | 34·2 (30·0–38·2) | 1·29 (1·09–1·45) | 68·8 (61·1–75·5) | 0·887 (0·722–1·07) | 51·9 (44·4–59·5) | 0·461 (0·359–0·585) |  |
|  | Senegal | 27·8 (24·1–31·3) | 97·2 (82·9–113) | 72·5 (65·6–79·1) | 70·6 (57–86·2) | 47·1 (40·7–53·7) | 33·2 (26–41·5) |  |
|  | Sierra Leone | 30·5 (26·6–34·5) | 35·7 (29·5–42·2) | 71·5 (64·1–78·0) | 25·6 (20·5–31·7) | 46·8 (40·5–54·2) | 12 (9·3–15·4) |  |
|  | Togo | 26·5 (22·9–30·2) | 20·5 (17·1–24) | 70·4 (63·6–76·6) | 14·5 (11·7–18) | 46·2 (39·8–52·9) | 6·69 (5·28–8·53) |  |
|  |  |  |  |  |  |  |  |  |

## Table S18. GBD 2023 diabetes cases counts in 2000 and 2023, for both sexes and ages 15 and older globally, and by Global Burden of Disease super-regions, regions, and countries and territories

|  | **Location** | **Counts, 2000 (per 1,000)** | **Counts, 2023 (per 1,000)** |  |
| --- | --- | --- | --- | --- |
|  | **Global** | **272000 (251000–294000)** | **560000 (517000–604000)** |  |
|  | **Central Europe, eastern Europe, and central Asia** | **15200 (14100–16300)** | **28400 (26400–30500)** |  |
|  | Central Asia | 2110 (1970–2270) | 4790 (4470–5120) |  |
|  | Armenia | 147 (138–158) | 199 (186–221) |  |
|  | Azerbaijan | 265 (243–290) | 645 (589–711) |  |
|  | Georgia | 170 (154–189) | 234 (217–256) |  |
|  | Kazakhstan | 582 (536–634) | 1130 (1030–1220) |  |
|  | Kyrgyzstan | 108 (99·7–119) | 255 (232–280) |  |
|  | Mongolia | 49·1 (45·2–53·8) | 134 (121–147) |  |
|  | Tajikistan | 133 (121–147) | 420 (388–458) |  |
|  | Turkmenistan | 89·9 (83·1–98) | 239 (221–257) |  |
|  | Uzbekistan | 563 (524–607) | 1540 (1430–1700) |  |
|  | Central Europe | 6720 (6260–7260) | 10600 (10100–11200) |  |
|  | Albania | 62·1 (55·9–69·4) | 132 (120–148) |  |
|  | Bosnia and Herzegovina | 171 (155–192) | 337 (298–370) |  |
|  | Bulgaria | 457 (425–488) | 597 (557–642) |  |
|  | Croatia | 228 (208–244) | 470 (435–503) |  |
|  | Czechia | 413 (385–443) | 977 (894–1070) |  |
|  | Hungary | 371 (343–400) | 683 (599–748) |  |
|  | Montenegro | 30·1 (27·6–33·9) | 69 (62·6–77·4) |  |
|  | North Macedonia | 99·8 (90·7–112) | 203 (183–227) |  |
|  | Poland | 3340 (3030–3770) | 4630 (4320–4940) |  |
|  | Romania | 698 (654–749) | 1140 (1030–1240) |  |
|  | Serbia | 492 (443–546) | 742 (682–823) |  |
|  | Slovakia | 169 (153–186) | 322 (294–353) |  |
|  | Slovenia | 87·1 (79·9–94·6) | 176 (159–191) |  |
|  | Eastern Europe | 6390 (5840–7030) | 12900 (11800–14300) |  |
|  | Belarus | 338 (307–364) | 613 (560–673) |  |
|  | Estonia | 54 (48·5–59·5) | 103 (93–112) |  |
|  | Latvia | 85·3 (77·2–94·5) | 169 (156–182) |  |
|  | Lithuania | 92·1 (80·2–101) | 211 (189–228) |  |
|  | Moldova | 148 (133–161) | 293 (265–318) |  |
|  | Russia | 4140 (3750–4600) | 9510 (8580–10600) |  |
|  | Ukraine | 1530 (1360–1690) | 2050 (1850–2290) |  |
|  | **High income** | **58300 (54300–62000)** | **99600 (93500–106000)** |  |
|  | Australasia | 732 (695–773) | 1460 (1360–1550) |  |
|  | Australia | 605 (572–643) | 1120 (1030–1210) |  |
|  | New Zealand | 127 (119–134) | 333 (313–352) |  |
|  | High-income Asia Pacific | 14000 (12800–15300) | 19400 (17900–21000) |  |
|  | Brunei | 13·7 (12·6–14·8) | 39·5 (36·2–42·7) |  |
|  | Japan | 11200 (10200–12400) | 13900 (12500–15400) |  |
|  | South Korea | 2530 (2390–2660) | 5110 (4940–5270) |  |
|  | Singapore | 194 (183–208) | 373 (340–417) |  |
|  | High-income North America | 19400 (18000–20900) | 42800 (40200–45400) |  |
|  | Canada | 1090 (1040–1150) | 2420 (2240–2610) |  |
|  | Greenland | 1·14 (1·04–1·26) | 2·43 (2·2–2·73) |  |
|  | USA | 18300 (16900–19700) | 40300 (37800–42900) |  |
|  | Southern Latin America | 1570 (1490–1660) | 3080 (2820–3290) |  |
|  | Argentina | 1210 (1150–1290) | 2250 (2040–2430) |  |
|  | Chile | 248 (234–265) | 655 (602–710) |  |
|  | Uruguay | 111 (101–121) | 179 (164–191) |  |
|  | Western Europe | 22600 (21200–23900) | 33000 (30600–35100) |  |
|  | Andorra | 2·31 (2·1–2·56) | 4·75 (4·3–5·28) |  |
|  | Austria | 295 (279–313) | 432 (402–467) |  |
|  | Belgium | 459 (424–494) | 671 (619–735) |  |
|  | Cyprus | 49·3 (45·4–54·2) | 97·5 (88·4–107) |  |
|  | Denmark | 187 (174–200) | 341 (306–367) |  |
|  | Finland | 367 (338–397) | 570 (522–626) |  |
|  | France | 2900 (2680–3150) | 4440 (4090–4830) |  |
|  | Germany | 5390 (5000–5730) | 6930 (6390–7500) |  |
|  | Greece | 493 (456–531) | 732 (672–799) |  |
|  | Iceland | 8·9 (8·18–9·82) | 19·5 (17·8–21·5) |  |
|  | Ireland | 97 (88·8–105) | 248 (226–275) |  |
|  | Israel | 231 (218–243) | 437 (408–466) |  |
|  | Italy | 3400 (3080–3760) | 4360 (3890–4840) |  |
|  | Luxembourg | 16·6 (15·2–18·4) | 32·8 (30·1–36·2) |  |
|  | Malta | 18 (16·6–19·8) | 36·2 (33·7–38·9) |  |
|  | Monaco | 1·56 (1·42–1·75) | 2·81 (2·54–3·13) |  |
|  | Netherlands | 699 (653–752) | 958 (893–1030) |  |
|  | Norway | 258 (232–283) | 377 (336–420) |  |
|  | Portugal | 645 (605–692) | 944 (881–1010) |  |
|  | San Marino | 1·09 (0·997–1·19) | 2·63 (2·38–2·92) |  |
|  | Spain | 2890 (2670–3120) | 4260 (3890–4670) |  |
|  | Sweden | 503 (472–544) | 834 (784–894) |  |
|  | Switzerland | 354 (326–381) | 515 (469–566) |  |
|  | UK | 3320 (3050–3610) | 5710 (5180–6170) |  |
|  | **Latin America and Caribbean** | **21000 (19400–22500)** | **46900 (43100–50500)** |  |
|  | Andean Latin America | 1040 (974–1120) | 2990 (2800–3190) |  |
|  | Bolivia | 215 (197–236) | 573 (524–632) |  |
|  | Ecuador | 399 (361–428) | 1100 (1030–1170) |  |
|  | Peru | 430 (394–472) | 1310 (1210–1430) |  |
|  | Caribbean | 2120 (2000–2240) | 3880 (3610–4130) |  |
|  | Antigua and Barbuda | 5·41 (4·99–5·83) | 10·3 (9·62–11·3) |  |
|  | The Bahamas | 17·8 (16·6–19·3) | 41·6 (38–46·1) |  |
|  | Barbados | 28·3 (26·8–30·1) | 50·8 (47·1–55·4) |  |
|  | Belize | 11·5 (10·7–12·5) | 36·6 (34·1–39·2) |  |
|  | Bermuda | 3·92 (3·63–4·29) | 6·23 (5·64–6·85) |  |
|  | Cuba | 475 (436–515) | 797 (725–881) |  |
|  | Dominica | 5·58 (5·15–6·02) | 9·65 (8·84–10·5) |  |
|  | Dominican Republic | 348 (322–375) | 865 (794–950) |  |
|  | Grenada | 9·3 (8·68–9·95) | 16·3 (15·2–17·3) |  |
|  | Guyana | 51·5 (47·5–55·2) | 80·8 (75–89·3) |  |
|  | Haiti | 297 (268–325) | 634 (571–693) |  |
|  | Jamaica | 159 (149–169) | 282 (261–304) |  |
|  | Puerto Rico | 395 (366–421) | 500 (460–538) |  |
|  | Saint Kitts and Nevis | 3·13 (2·88–3·37) | 5·49 (5·07–6) |  |
|  | Saint Lucia | 13·4 (11·8–14·3) | 25·5 (23·1–27·3) |  |
|  | Saint Vincent and the Grenadines | 9·26 (8·45–9·88) | 16·2 (15·1–17·3) |  |
|  | Suriname | 29·2 (26·5–32·1) | 75·8 (69·6–82·5) |  |
|  | Trinidad and Tobago | 173 (150–185) | 288 (269–305) |  |
|  | Virgin Islands | 8·61 (7·86–9·39) | 12 (10·9–13·2) |  |
|  | Central Latin America | 11400 (10600–12300) | 25400 (23400–27300) |  |
|  | Colombia | 1340 (1240–1460) | 3400 (3090–3700) |  |
|  | Costa Rica | 179 (164–197) | 510 (467–569) |  |
|  | El Salvador | 233 (215–250) | 454 (418–495) |  |
|  | Guatemala | 383 (356–409) | 1270 (1190–1380) |  |
|  | Honduras | 163 (147–178) | 468 (425–516) |  |
|  | Mexico | 7940 (7170–8670) | 16500 (14900–18000) |  |
|  | Nicaragua | 118 (109–129) | 325 (295–349) |  |
|  | Panama | 123 (112–134) | 334 (310–359) |  |
|  | Venezuela | 947 (877–1020) | 2190 (2010–2380) |  |
|  | Tropical Latin America | 6430 (5790–7020) | 14600 (13100–16000) |  |
|  | Brazil | 6250 (5620–6830) | 14100 (12700–15500) |  |
|  | Paraguay | 181 (166–195) | 467 (431–502) |  |
|  | **North Africa and Middle East** | **19000 (17500–20500)** | **66500 (61200–72000)** |  |
|  | Afghanistan | 651 (587–714) | 2030 (1850–2220) |  |
|  | Algeria | 1370 (1250–1510) | 4870 (4490–5290) |  |
|  | Bahrain | 53·7 (48·4–59) | 307 (281–333) |  |
|  | Egypt | 3430 (3140–3740) | 12200 (11200–13200) |  |
|  | Iran | 2310 (2080–2550) | 7880 (7060–8740) |  |
|  | Iraq | 1150 (1060–1250) | 4490 (4070–4870) |  |
|  | Jordan | 249 (234–265) | 1410 (1300–1510) |  |
|  | Kuwait | 150 (135–164) | 927 (851–1020) |  |
|  | Lebanon | 250 (226–273) | 747 (686–808) |  |
|  | Libya | 254 (232–277) | 1030 (933–1130) |  |
|  | Morocco | 1370 (1220–1500) | 3920 (3600–4230) |  |
|  | Oman | 88·5 (82–95·3) | 479 (431–523) |  |
|  | Palestine | 124 (115–133) | 471 (428–518) |  |
|  | Qatar | 51·6 (46·4–57·7) | 451 (405–499) |  |
|  | Saudi Arabia | 1760 (1620–1930) | 6680 (6100–7300) |  |
|  | Sudan | 839 (758–919) | 2480 (2280–2730) |  |
|  | Syria | 667 (607–735) | 2190 (2000–2400) |  |
|  | Tunisia | 563 (515–616) | 1480 (1360–1610) |  |
|  | Türkiye | 3140 (2890–3400) | 8740 (7950–9580) |  |
|  | United Arab Emirates | 232 (208–261) | 2080 (1880–2280) |  |
|  | Yemen | 258 (234–285) | 1580 (1410–1730) |  |
|  | **South Asia** | **52500 (47900–57500)** | **133000 (120000–145000)** |  |
|  | Bangladesh | 4060 (3750–4440) | 10400 (9610–11300) |  |
|  | Bhutan | 18·6 (17·2–20·1) | 37·8 (34·6–41·7) |  |
|  | India | 41800 (38000–45800) | 107000 (96500–116000) |  |
|  | Nepal | 789 (715–863) | 1760 (1630–1920) |  |
|  | Pakistan | 5860 (5280–6430) | 13900 (12500–15300) |  |
|  | **Southeast Asia, east Asia, and Oceania** | **92400 (84000–102000)** | **154000 (141000–168000)** |  |
|  | East Asia | 74600 (67600–82700) | 113000 (104000–124000) |  |
|  | China | 72200 (65300–80100) | 109000 (1e+05–120000) |  |
|  | North Korea | 1020 (927–1130) | 1750 (1560–1940) |  |
|  | Taiwan | 1360 (1260–1460) | 2280 (2110–2460) |  |
|  | Oceania | 425 (393–459) | 1020 (953–1110) |  |
|  | American Samoa | 5·92 (5·54–6·32) | 8·49 (7·84–9·16) |  |
|  | Cook Islands | 2·56 (2·39–2·73) | 2·82 (2·61–3·04) |  |
|  | Fiji | 74·8 (69·8–80·3) | 159 (147–169) |  |
|  | Guam | 8·53 (7·77–9·32) | 14·1 (12·9–15·4) |  |
|  | Kiribati | 6·47 (6·03–7) | 14·6 (13·4–15·8) |  |
|  | Marshall Islands | 4·28 (3·91–4·7) | 6·09 (5·55–6·63) |  |
|  | Federated States of Micronesia | 8·57 (8·05–9·07) | 18·1 (17–19·2) |  |
|  | Nauru | 0·819 (0·753–0·883) | 1·34 (1·23–1·44) |  |
|  | Niue | 0·259 (0·237–0·28) | 0·33 (0·304–0·359) |  |
|  | Northern Mariana Islands | 5·06 (4·53–5·64) | 6·28 (5·77–6·82) |  |
|  | Palau | 1·73 (1·56–1·89) | 2·94 (2·7–3·14) |  |
|  | Papua New Guinea | 236 (214–257) | 630 (579–691) |  |
|  | Samoa | 11·6 (10·8–12·6) | 24·6 (22·7–26·7) |  |
|  | Solomon Islands | 17·3 (15·9–18·7) | 56·4 (51·3–61·9) |  |
|  | Tokelau | 0·186 (0·171–0·202) | 0·287 (0·261–0·309) |  |
|  | Tonga | 7·34 (6·79–7·98) | 12·3 (11·4–13·3) |  |
|  | Tuvalu | 0·647 (0·585–0·708) | 1·17 (1·07–1·26) |  |
|  | Vanuatu | 9·02 (8·24–9·85) | 23·1 (21·1–25·3) |  |
|  | Southeast Asia | 17400 (15800–18900) | 39800 (36300–43400) |  |
|  | Cambodia | 206 (187–226) | 558 (506–607) |  |
|  | Indonesia | 6120 (5480–6800) | 14900 (13300–16600) |  |
|  | Laos | 131 (117–144) | 303 (275–332) |  |
|  | Malaysia | 1210 (1100–1320) | 2670 (2440–2930) |  |
|  | Maldives | 6·51 (5·96–7·18) | 24·3 (21·9–27) |  |
|  | Mauritius | 124 (118–131) | 238 (225–254) |  |
|  | Myanmar | 1780 (1620–1950) | 3410 (3150–3690) |  |
|  | Philippines | 2480 (2240–2720) | 5630 (5100–6150) |  |
|  | Seychelles | 4·49 (4·14–4·88) | 11·1 (10·1–12·2) |  |
|  | Sri Lanka | 1040 (968–1110) | 2260 (2100–2430) |  |
|  | Thailand | 2470 (2270–2700) | 5340 (4880–5860) |  |
|  | Timor-Leste | 15·2 (13·9–16·9) | 37·6 (34–41·5) |  |
|  | Viet Nam | 1750 (1590–1920) | 4350 (3990–4750) |  |
|  | **Sub-Saharan Africa** | **13500 (12300–14700)** | **31900 (29000–34700)** |  |
|  | Central sub-Saharan Africa | 2150 (1960–2350) | 5330 (4860–5820) |  |
|  | Angola | 454 (410–499) | 1460 (1320–1610) |  |
|  | Central African Republic | 107 (97·5–117) | 237 (216–259) |  |
|  | Congo (Brazzaville) | 113 (103–123) | 271 (244–297) |  |
|  | DR Congo | 1400 (1270–1540) | 3180 (2880–3480) |  |
|  | Equatorial Guinea | 19·1 (17·3–20·8) | 64·1 (57·7–71·6) |  |
|  | Gabon | 52·7 (47·8–57·1) | 112 (102–122) |  |
|  | Eastern sub-Saharan Africa | 3670 (3360–3980) | 8100 (7380–8790) |  |
|  | Burundi | 95 (85·9–104) | 223 (200–245) |  |
|  | Comoros | 11·4 (10·4–12·4) | 25·9 (23·7–28·2) |  |
|  | Djibouti | 9·5 (8·59–10·4) | 31·3 (28·6–34) |  |
|  | Eritrea | 63·3 (58–68·7) | 147 (133–161) |  |
|  | Ethiopia | 1100 (993–1200) | 2070 (1860–2250) |  |
|  | Kenya | 465 (418–510) | 1160 (1040–1270) |  |
|  | Madagascar | 216 (198–236) | 503 (452–550) |  |
|  | Malawi | 127 (117–138) | 274 (251–298) |  |
|  | Mozambique | 211 (192–228) | 451 (413–490) |  |
|  | Rwanda | 107 (98·2–118) | 233 (215–253) |  |
|  | Somalia | 147 (135–161) | 358 (327–391) |  |
|  | South Sudan | 95 (86·7–103) | 163 (148–177) |  |
|  | Uganda | 385 (349–421) | 864 (783–943) |  |
|  | Tanzania | 450 (414–490) | 1080 (985–1170) |  |
|  | Zambia | 184 (167–200) | 522 (476–570) |  |
|  | Southern sub-Saharan Africa | 2330 (2090–2540) | 4980 (4490–5430) |  |
|  | Botswana | 45·9 (41·9–50) | 91·4 (83·9–100) |  |
|  | Eswatini | 27·8 (25·3–30·2) | 53·8 (49–58·2) |  |
|  | Lesotho | 40·6 (36·9–44·4) | 60·9 (55·7–66·1) |  |
|  | Namibia | 45·3 (41·3–50) | 104 (95·8–113) |  |
|  | South Africa | 1940 (1740–2130) | 4250 (3830–4650) |  |
|  | Zimbabwe | 230 (208–252) | 416 (379–455) |  |
|  | Western sub-Saharan Africa | 5370 (4880–5850) | 13500 (12300–14800) |  |
|  | Benin | 120 (109–131) | 376 (344–410) |  |
|  | Burkina Faso | 158 (146–171) | 338 (310–369) |  |
|  | Cabo Verde | 13·9 (12·5–15) | 26·4 (23·7–28·9) |  |
|  | Cameroon | 285 (263–309) | 956 (860–1050) |  |
|  | Chad | 176 (159–195) | 379 (341–417) |  |
|  | Côte d'Ivoire | 383 (350–421) | 1000 (903–1110) |  |
|  | The Gambia | 37·9 (34·2–41·5) | 94·8 (85–104) |  |
|  | Ghana | 483 (444–523) | 1200 (1090–1320) |  |
|  | Guinea | 169 (156–184) | 320 (288–352) |  |
|  | Guinea-Bissau | 30·4 (27·6–33·4) | 64·3 (57·7–71·2) |  |
|  | Liberia | 74·1 (67·3–81·5) | 196 (178–216) |  |
|  | Mali | 465 (413–516) | 1020 (923–1130) |  |
|  | Mauritania | 78·5 (71·3–86·4) | 174 (157–191) |  |
|  | Niger | 209 (189–232) | 540 (490–591) |  |
|  | Nigeria | 2140 (1930–2350) | 5540 (4980–6100) |  |
|  | São Tomé and Príncipe | 3·77 (3·43–4·14) | 10 (8·93–11·2) |  |
|  | Senegal | 350 (316–381) | 849 (761–934) |  |
|  | Sierra Leone | 117 (106–129) | 240 (217–263) |  |
|  | Togo | 77·5 (71·6–84·6) | 208 (188–227) |  |
|  |  |  |  |  |

## Table S19. Cascade of care input data characteristics and citations

| **Country** | **Title** | **Years** | **Data type** | **Diabetes case definition** | **Model(s)** | **Citation** |
| --- | --- | --- | --- | --- | --- | --- |
| Afghanistan | Afghanistan STEPS Noncommunicable Disease Risk Factors Survey 2018 | 2018 - 2018 | Microdata | FPG ≥ 7 mmol/L or on treatment | Undiagnosed, Diagnosed & Untreated, Treated Envelope, Treated & Suboptimal glycaemic levels, Treated & Optimal glycaemic levels | Ministry of Public Health (Afghanistan), World Health Organization (WHO). Afghanistan STEPS Noncommunicable Disease Risk Factors Survey 2018. Geneva, Switzerland: World Health Organization (WHO). |
| Algeria | Algeria - Sétif and Mostaganem STEPS Noncommunicable Disease Risk Factors Survey 2003 | 2003 - 2003 | Microdata | FPG ≥ 7 mmol/L or on treatment | Undiagnosed, Diagnosed & Untreated, Treated Envelope, Treated & Suboptimal glycaemic levels, Treated & Optimal glycaemic levels | Ministry of Health, Population and Hospital Reform (Algeria), World Health Organization (WHO). Algeria - Sétif and Mostaganem STEPS Noncommunicable Disease Risk Factors Survey 2003. |
| Algeria | Algeria STEPS Noncommunicable Disease Risk Factors Survey 2016-2017 | 2016 - 2017 | Microdata | FPG ≥ 7 mmol/L or on treatment | Undiagnosed, Diagnosed & Untreated, Treated Envelope, Treated & Suboptimal glycaemic levels, Treated & Optimal glycaemic levels | Ministry of Health, Population and Hospital Reform (Algeria), World Health Organization (WHO). Algeria STEPS Noncommunicable Disease Risk Factors Survey 2016-2017. |
| American Samoa | American Samoa STEPS Noncommunicable Disease Risk Factors Survey 2004 | 2004 - 2004 | Microdata | FPG ≥ 7 mmol/L or on treatment | Undiagnosed, Diagnosed & Untreated, Treated Envelope, Treated & Suboptimal glycaemic levels, Treated & Optimal glycaemic levels | Department of Health (American Samoa), Monash University (Australia), World Health Organization (WHO). American Samoa STEPS Noncommunicable Disease Risk Factors Survey 2004. |
| American Samoa | The Protection Gap - Diagnosis, Treatment Status, and Disease Control for People with Diabetes and Hypertension in the US-Affiliated Pacific Islands | 2018 - 2018 | Scientific literature | FPG ≥ 7 mmol/L or on treatment | Undiagnosed, Diagnosed & Untreated, Treated Envelope, Treated & Suboptimal glycaemic levels, Treated & Optimal glycaemic levels | Durand AM, Cash HL, Reichhardt MLE, Taulung L, Tolenoa NM. The Protection Gap - Diagnosis, Treatment Status, and Disease Control for People with Diabetes and Hypertension in the US-Affiliated Pacific Islands. Hawaii J Health Soc Welf. 2023; 82(3): 66-71. |
| Angola | Prevalence, awareness, treatment and control of hypertension, diabetes and hypercholesterolaemia among adults in Dande municipality, Angola | 2013 - 2014 | Scientific literature | FPG ≥ 7 mmol/L or on treatment | Undiagnosed, Diagnosed & Untreated, Treated Envelope, Treated & Suboptimal glycaemic levels, Treated & Optimal glycaemic levels | Pedro JM, Brito M, Barros H. Prevalence, awareness, treatment and control of hypertension, diabetes and hypercholesterolaemia among adults in Dande municipality, Angola. *Cardiovasc J Afr*. 2017; 28: 1–10. |
| Argentina | Argentina National Survey of Risk Factors 2018 | 2018 - 2018 | Microdata | FPG ≥ 7 mmol/L or on treatment | Undiagnosed, Diagnosed & Untreated, Treated Envelope, Treated & Suboptimal glycaemic levels, Treated & Optimal glycaemic levels | Ministry of Health and Social Development (Argentina), National Institute of Statistics and Censuses (Argentina). Argentina National Survey of Risk Factors 2018. Buenos Aires, Argentina: National Institute of Statistics and Censuses (Argentina). |
| Armenia | Armenia STEPS Noncommunicable Disease Risk Factors Survey 2016 | 2016 - 2016 | Microdata | FPG ≥ 7 mmol/L or on treatment | Undiagnosed, Diagnosed & Untreated, Treated Envelope, Treated & Suboptimal glycaemic levels, Treated & Optimal glycaemic levels | World Health Organization (WHO). Armenia STEPS Noncommunicable Disease Risk Factors Survey 2016. |
| Australia | Australia Diabetes, Obesity and Lifestyle Study 1999-2000 | 1999 - 2000 | Microdata | FPG ≥ 7 mmol/L or on treatment | Undiagnosed, Diagnosed & Untreated, Treated Envelope, Treated & Suboptimal glycaemic levels, Treated & Optimal glycaemic levels | International Diabetes Institute (IDI). Australia Diabetes, Obesity and Lifestyle Study 1999-2000. Melbourne, Australia: International Diabetes Institute (IDI). |
| Azerbaijan | Azerbaijan STEPS Noncommunicable Disease Risk Factors Survey 2017 | 2017 - 2017 | Microdata | FPG ≥ 7 mmol/L or on treatment | Undiagnosed, Diagnosed & Untreated, Treated Envelope, Treated & Suboptimal glycaemic levels, Treated & Optimal glycaemic levels | Azerbaijan Ministry of Health, World Health Organization (WHO). Azerbaijan STEPS Noncommunicable Disease Risk Factors Survey 2017. |
| Bangladesh | Bangladesh Urban Health Survey 2006 | 2006 - 2006 | Scientific literature | FPG ≥ 7 mmol/L or on treatment | Treated Envelope, Treated & Suboptimal glycaemic levels, Treated & Optimal glycaemic levels | Associates for Community and Population Research (ACPR), International Centre for Diarrhoeal Disease Research, Bangladesh (ICDDR,B), MEASURE Evaluation Project, Carolina Population Center, University of North Carolina, National Institute of Population Research and Training (NIPORT). Bangladesh Urban Health Survey 2006. |
| Bangladesh | Bangladesh Demographic and Health Survey 2011-2012 | 2011 - 2012 | Microdata | FPG ≥ 7 mmol/L or on treatment | Undiagnosed, Diagnosed & Untreated, Treated Envelope, Treated & Suboptimal glycaemic levels, Treated & Optimal glycaemic levels | ICF Macro, Mitra and Associates, National Institute of Population Research and Training (NIPORT). Bangladesh Demographic and Health Survey 2011-2012. Calverton, United States of America: ICF Macro. |
| Bangladesh | Bangladesh Demographic and Health Survey 2017-2018 | 2017 - 2018 | Microdata | FPG ≥ 7 mmol/L or on treatment | Undiagnosed, Diagnosed & Untreated, Treated Envelope, Treated & Suboptimal glycaemic levels, Treated & Optimal glycaemic levels | ICF International, Mitra and Associates, National Institute of Population Research and Training (NIPORT). Bangladesh Demographic and Health Survey 2017-2018. Fairfax, United States of America: ICF International, 2020. |
| Bangladesh | Bangladesh STEPS Noncommunicable Disease Risk Factors Survey 2018 | 2018 - 2018 | Microdata | FPG ≥ 7 mmol/L or on treatment | Undiagnosed, Diagnosed & Untreated, Treated Envelope, Treated & Suboptimal glycaemic levels, Treated & Optimal glycaemic levels | Ministry of Health and Family Welfare (Bangladesh), National Institute of Preventive and Social Medicine, University of Dhaka (Bangladesh), World Health Organization (WHO). Bangladesh STEPS Noncommunicable Disease Risk Factors Survey 2018. Geneva, Switzerland: World Health Organization (WHO). |
| Barbados | Barbados STEPS Noncommunicable Disease Risk Factors Survey 2007 | 2007 - 2007 | Microdata | FPG ≥ 7 mmol/L or on treatment | Undiagnosed, Diagnosed & Untreated, Treated Envelope, Treated & Suboptimal glycaemic levels, Treated & Optimal glycaemic levels | Ministry of Health (Barbados). Barbados STEPS Noncommunicable Disease Risk Factors Survey 2007. |
| Belarus | Belarus STEPS Noncommunicable Disease Risk Factors Survey 2016-2017 | 2016 - 2017 | Microdata | FPG ≥ 7 mmol/L or on treatment | Undiagnosed, Diagnosed & Untreated, Treated Envelope, Treated & Suboptimal glycaemic levels, Treated & Optimal glycaemic levels | Republican Scientific and Practical Center of Medical Technologies, Informatization, Management and Economics of Public Health (Belarus), World Health Organization (WHO). Belarus STEPS Noncommunicable Disease Risk Factors Survey 2016-2017. |
| Belarus | Belarus STEPS Noncommunicable Disease Risk Factors Survey 2020 | 2020 - 2020 | Scientific literature | FPG ≥ 7 mmol/L or on treatment | Treated Envelope | Republican Scientific and Practical Center of Medical Technologies, Informatization, Management and Economics of Public Health (Belarus), World Health Organization (WHO). Belarus STEPS Noncommunicable Disease Risk Factors Survey 2020. |
| Benin | Benin - Littoral STEPS Noncommunicable Disease Risk Factors Survey 2007 | 2007 - 2007 | Microdata | FPG ≥ 7 mmol/L or on treatment | Undiagnosed, Diagnosed & Untreated, Treated Envelope, Treated & Suboptimal glycaemic levels, Treated & Optimal glycaemic levels | Ministry of Health (Benin), World Health Organization (WHO). Benin - Littoral STEPS Noncommunicable Disease Risk Factors Survey 2007. |
| Benin | Benin STEPS Noncommunicable Disease Risk Factors Survey 2008 | 2008 - 2008 | Microdata | FPG ≥ 7 mmol/L or on treatment | Undiagnosed, Diagnosed & Untreated, Treated Envelope, Treated & Suboptimal glycaemic levels, Treated & Optimal glycaemic levels | Ministry of Health (Benin), World Health Organization (WHO). Benin STEPS Noncommunicable Disease Risk Factors Survey 2008. |
| Benin | Benin STEPS Noncommunicable Disease Risk Factors Survey 2015 | 2015 - 2015 | Microdata | FPG ≥ 7 mmol/L or on treatment | Undiagnosed, Diagnosed & Untreated, Treated Envelope, Treated & Suboptimal glycaemic levels, Treated & Optimal glycaemic levels | Ministry of Health (Benin), World Health Organization (WHO). Benin STEPS Noncommunicable Disease Risk Factors Survey 2015. |
| Bhutan | Bhutan - Thimphu STEPS Noncommunicable Disease Risk Factors Survey 2007 | 2007 - 2007 | Microdata | FPG ≥ 7 mmol/L or on treatment | Undiagnosed, Diagnosed & Untreated, Treated Envelope, Treated & Suboptimal glycaemic levels, Treated & Optimal glycaemic levels | Ministry of Health (Bhutan), World Health Organization (WHO). Bhutan - Thimphu STEPS Noncommunicable Disease Risk Factors Survey 2007. |
| Bhutan | Bhutan STEPS Noncommunicable Disease Risk Factors Survey 2014 | 2014 - 2014 | Microdata | FPG ≥ 7 mmol/L or on treatment | Undiagnosed, Diagnosed & Untreated, Treated Envelope, Treated & Suboptimal glycaemic levels, Treated & Optimal glycaemic levels | Ministry of Health (Bhutan), World Health Organization (WHO). Bhutan STEPS Noncommunicable Disease Risk Factors Survey 2014. |
| Bhutan | Bhutan STEPS Noncommunicable Disease Risk Factors Survey 2019 | 2019 - 2019 | Microdata | FPG ≥ 7 mmol/L or on treatment | Undiagnosed, Diagnosed & Untreated, Treated Envelope, Treated & Suboptimal glycaemic levels, Treated & Optimal glycaemic levels | Ministry of Health (Bhutan), World Health Organization (WHO). Bhutan STEPS Noncommunicable Disease Risk Factors Survey 2019. WHO NCD Microdata Repository, 2021. |
| Botswana | Botswana STEPS Noncommunicable Disease Risk Factors Survey 2014 | 2014 - 2014 | Microdata | FPG ≥ 7 mmol/L or on treatment | Undiagnosed, Diagnosed & Untreated, Treated Envelope, Treated & Suboptimal glycaemic levels, Treated & Optimal glycaemic levels | Ministry of Health (Botswana), World Health Organization (WHO). Botswana STEPS Noncommunicable Disease Risk Factors Survey 2014. |
| Brazil | Type 2 diabetes: prevalence and associated factors in a Brazilian community--the Bambuí health and aging study | 1997 - 1997 | Scientific literature | FPG ≥ 7 mmol/L or on treatment | Treated Envelope | Passos VM de A, Barreto SM, Diniz LM, Lima-Costa MF. Type 2 diabetes: prevalence and associated factors in a Brazilian community--the Bambuí health and aging study. Sao Paulo Med J. 2005; 123(2): 66-71. |
| Brazil | Birth cohort differences in cardiovascular risk factors in a Brazilian population of older elderly: the Bambuí Cohort Study of Aging (1997 and 2008) | 2008 - 2008 | Scientific literature | FPG ≥ 7 mmol/L or on treatment | Treated Envelope | Freitas MPD, Loyola Filho AI de, Lima-Costa MF. Birth cohort differences in cardiovascular risk factors in a Brazilian population of older elderly: the Bambuí Cohort Study of Aging (1997 and 2008). Cad Saude Publica. 2011; S409-417. |
| Brazil | Awareness of the diagnosis, treatment, and control of diabetes mellitus in Brazil | 2013 - 2015 | Scientific literature | HbA1c ≥ 6.5% or on treatment | Undiagnosed, Diagnosed & Untreated, Treated Envelope | Tonaco LAB, Velasquez-Melendez G, Moreira AD, Andrade FCD, Malta DC, Felisbino-Mendes MS. Awareness of the diagnosis, treatment, and control of diabetes mellitus in Brazil. Rev Saude Publica. 2023; 57: 75. |
| Brunei Darussalam | Brunei STEPS Noncommunicable Disease Risk Factors Survey 2015-2016 | 2015 - 2016 | Microdata | FPG ≥ 7 mmol/L or on treatment | Undiagnosed, Diagnosed & Untreated, Treated Envelope, Treated & Suboptimal glycaemic levels, Treated & Optimal glycaemic levels | World Health Organization (WHO). Brunei STEPS Noncommunicable Disease Risk Factors Survey 2015-2016. |
| Cabo Verde | Cape Verde STEPS Noncommunicable Disease Risk Factors Survey 2007 | 2007 - 2007 | Microdata | FPG ≥ 7 mmol/L or on treatment | Undiagnosed, Diagnosed & Untreated, Treated Envelope, Treated & Suboptimal glycaemic levels, Treated & Optimal glycaemic levels | World Health Organization (WHO). Cape Verde STEPS Noncommunicable Disease Risk Factors Survey 2007. |
| Cabo Verde | Cape Verde STEPS Noncommunicable Disease Risk Factors Survey 2020 | 2020 - 2020 | Microdata | FPG ≥ 7 mmol/L or on treatment | Undiagnosed, Diagnosed & Untreated, Treated Envelope, Treated & Suboptimal glycaemic levels, Treated & Optimal glycaemic levels | Cape Verde National Statistics Institute (INE), Ministry of Health (Cape Verde), World Health Organization (WHO). Cape Verde STEPS Noncommunicable Disease Risk Factors Survey 2020. WHO NCD Microdata Repository, 2023. |
| Cambodia | Cambodia STEPS Noncommunicable Disease Risk Factors Survey 2010 | 2010 - 2010 | Microdata | FPG ≥ 7 mmol/L or on treatment | Undiagnosed, Diagnosed & Untreated, Treated Envelope, Treated & Suboptimal glycaemic levels, Treated & Optimal glycaemic levels | Ministry of Health (Cambodia), University of Health Sciences (Cambodia), World Health Organization (WHO). Cambodia STEPS Noncommunicable Disease Risk Factors Survey 2010. |
| Cameroon | Cameroon STEPS Noncommunicable Disease Risk Factors Survey 2003 | 2003 - 2003 | Microdata | FPG ≥ 7 mmol/L or on treatment | Undiagnosed, Diagnosed & Untreated, Treated Envelope, Treated & Suboptimal glycaemic levels, Treated & Optimal glycaemic levels | Health of Populations in Transition (HoPiT) Research Group (Cameroon), Ministry of Public Health (Cameroon), World Diabetes Foundation (WDF), World Health Organization (WHO). Cameroon STEPS Noncommunicable Disease Risk Factors Survey 2003. |
| Cameroon | Coincidence of diabetes mellitus and hypertension in a semi-urban Cameroonian population: a cross-sectional study | 2012 - 2012 | Scientific literature | FPG ≥ 7 mmol/L or on treatment | Undiagnosed, Diagnosed & Untreated, Treated Envelope, Treated & Suboptimal glycaemic levels, Treated & Optimal glycaemic levels | Katte J-C, Dzudie A, Sobngwi E, Mbong EN, Fetse GT, Kouam CK, Kengne A-P. Coincidence of diabetes mellitus and hypertension in a semi-urban Cameroonian population: a cross-sectional study. BMC Public Health. 2014; 14: 696. |
| Cameroon | Prevalence, treatment, and control of diabetes in a community setting in Ngaoundéré, Cameroon | 2015 - 2016 | Scientific literature | FPG ≥ 7 mmol/L or on treatment | Treated Envelope | Pancha Mbouemboue O, Ngoufack Tsougmo JO, Sakinatou I, Velinga Ndolok AC, Tangyi Tamanji M. Prevalence, treatment, and control of diabetes in a community setting in Ngaoundéré, Cameroon. Med Sante Trop. 2018; 28(4): 379-384. |
| Central African Republic | Central African Republic - Bangui STEPS Noncommunicable Disease Risk Factors Survey 2010 | 2010 - 2010 | Microdata | FPG ≥ 7 mmol/L or on treatment | Undiagnosed, Diagnosed & Untreated, Treated Envelope, Treated & Suboptimal glycaemic levels, Treated & Optimal glycaemic levels | World Health Organization (WHO). Central African Republic - Bangui STEPS Noncommunicable Disease Risk Factors Survey 2010. |
| Central African Republic | Central African Republic - Bangui and Ombella M'Poko STEPS Noncommunicable Disease Risk Factors Survey 2017 | 2017 - 2017 | Microdata | FPG ≥ 7 mmol/L or on treatment | Undiagnosed, Diagnosed & Untreated, Treated Envelope, Treated & Suboptimal glycaemic levels, Treated & Optimal glycaemic levels | World Health Organization (WHO). Central African Republic - Bangui and Ombella M'Poko STEPS Noncommunicable Disease Risk Factors Survey 2017. WHO NCD Microdata Repository, 2020. |
| Chile | Chile National Health Survey 2003 | 2003 - 2003 | Microdata | FPG ≥ 7 mmol/L or on treatment | Undiagnosed, Diagnosed & Untreated, Treated Envelope, Treated & Suboptimal glycaemic levels, Treated & Optimal glycaemic levels | Department of Public Health, Pontifical Catholic University of Chile, Institute of Nutrition and Food Technology (INTA), University of Chile, Ministry of Health (Chile), Pan American Health Organization (PAHO). Chile National Health Survey 2003. |
| Chile | Chile National Health Survey 2009-2010 | 2009 - 2010 | Microdata | FPG ≥ 7 mmol/L or on treatment | Undiagnosed, Diagnosed & Untreated, Treated Envelope, Treated & Suboptimal glycaemic levels, Treated & Optimal glycaemic levels | Ministry of Health (Chile). Chile National Health Survey 2009-2010. |
| Chile | Chile National Health Survey 2016-2017 | 2016 - 2017 | Microdata | FPG ≥ 7 mmol/L or on treatment | Undiagnosed, Diagnosed & Untreated, Treated Envelope, Treated & Suboptimal glycaemic levels, Treated & Optimal glycaemic levels | Department of Epidemiology, Ministry of Health (Chile). Chile National Health Survey 2016-2017. Chile: Department of Epidemiology, Ministry of Health (Chile). |
| China | Increasing prevalence and low awareness, treatment and control of diabetes mellitus among Chinese adults: the InterASIA study | 2000 - 2001 | Scientific literature | FPG ≥ 7 mmol/L or on treatment | Undiagnosed, Treated & Optimal glycaemic levels | Hu D, Fu P, Xie J, Chen CS, Yu D, Whelton PK, He J, Gu D, MS for the InterASIA Collaborative Group. Increasing prevalence and low awareness, treatment and control of diabetes mellitus among Chinese adults: the InterASIA study. Diabetes Res Clin Pract. 2008; 81(2): 250-7. |
| China | Awareness, treatment and control of type 2 diabetes among Chinese elderly and its changing trend for past decade | 2001 - 2001 | Scientific literature | FPG ≥ 7 mmol/L or on treatment | Undiagnosed, Diagnosed & Untreated, Treated Envelope, Treated & Suboptimal glycaemic levels, Treated & Optimal glycaemic levels | Liu M, Wang J, He Y, Jiang B, Wu L, Wang Y, Di Z, Zeng J. Awareness, treatment and control of type 2 diabetes among Chinese elderly and its changing trend for past decade. BMC Public Health. 2016; 16(1): 278. |
| China | China - Beijing Eye Study 2006 | 2006 - 2006 | Microdata | FPG ≥ 7 mmol/L or on treatment | Undiagnosed, Diagnosed & Untreated, Treated Envelope, Treated & Suboptimal glycaemic levels, Treated & Optimal glycaemic levels | Xu J, Xu L, Du KF, Shao L, Chen CX, Zhou JQ, Wang YX, You QS, Jonas JB, Wei WB. Subfoveal choroidal thickness in diabetes and diabetic retinopathy [Unpublished data]. *Ophthalmology*. 2013 Oct;120(10):2023-8. |
| China | China - KaiLuan Study Baseline 2006-2007 | 2006 - 2007 | Microdata | FPG ≥ 7 mmol/L or on treatment | Undiagnosed, Diagnosed & Untreated, Treated Envelope, Treated & Suboptimal glycaemic levels, Treated & Optimal glycaemic levels | Kailuan General Hospital, Hebei United University. China - KaiLuan Study Baseline 2006-2007. |
| China | Type 2 Diabetes in Xinjiang Uygur Autonomous Region, China | 2007 - 2010 | Scientific literature | FPG ≥ 7 mmol/L or on treatment | Undiagnosed, Diagnosed & Untreated, Treated Envelope, Treated & Suboptimal glycaemic levels, Treated & Optimal glycaemic levels | Yang Y-N, Xie X, Ma Y-T, Li X-M, Fu Z-Y, Ma X, Huang D, Chen B-D, Liu F, Huang Y, Liu C, Zheng Y-Y, Baituola G, YU Z-X, Chen Y. Type 2 Diabetes in Xinjiang Uygur Autonomous Region, China. PLoS One. 2012; 7(4). |
| China | China - KaiLuan Study 2008-2009 | 2008 - 2009 | Microdata | FPG ≥ 7 mmol/L or on treatment | Undiagnosed, Diagnosed & Untreated, Treated Envelope, Treated & Suboptimal glycaemic levels, Treated & Optimal glycaemic levels | Kailuan General Hospital, Hebei United University. China - KaiLuan Study 2008-2009. |
| China | China Health and Nutrition Survey | 2009 - 2009 | Microdata | FPG ≥ 7 mmol/L or on treatment | Undiagnosed, Diagnosed & Untreated, Treated Envelope, Treated & Suboptimal glycaemic levels, Treated & Optimal glycaemic levels | Carolina Population Center, University of North Carolina at Chapel Hill, Chinese Center for Disease Control and Prevention (CCDC). China Health and Nutrition Survey. Chapel Hill, United States of America: Carolina Population Center, University of North Carolina at Chapel Hill. |
| China | Awareness, treatment and control of type 2 diabetes among Chinese elderly and its changing trend for past decade | 2010 - 2010 | Scientific literature | FPG ≥ 7 mmol/L or on treatment | Undiagnosed, Diagnosed & Untreated, Treated Envelope, Treated & Suboptimal glycaemic levels, Treated & Optimal glycaemic levels | Liu M, Wang J, He Y, Jiang B, Wu L, Wang Y, Di Z, Zeng J. Awareness, treatment and control of type 2 diabetes among Chinese elderly and its changing trend for past decade. BMC Public Health. 2016; 16(1): 278. |
| China | China - KaiLuan Study 2010-2011 | 2010 - 2011 | Microdata | FPG ≥ 7 mmol/L or on treatment | Undiagnosed, Diagnosed & Untreated, Treated Envelope, Treated & Suboptimal glycaemic levels, Treated & Optimal glycaemic levels | Kailuan General Hospital, Hebei United University. China - KaiLuan Study 2010-2011. |
| China | China - Beijing Eye Study 2011 | 2011 - 2011 | Microdata | FPG ≥ 7 mmol/L or on treatment | Treated Envelope, Treated & Suboptimal glycaemic levels, Treated & Optimal glycaemic levels | Xu J, Xu L, Du KF, Shao L, Chen CX, Zhou JQ, Wang YX, You QS, Jonas JB, Wei WB. Subfoveal choroidal thickness in diabetes and diabetic retinopathy [Unpublished data]. *Ophthalmology*. 2013 Oct;120(10):2023-8. |
| China | Prevalence and awareness of diabetes mellitus among a rural population in China: results from Liaoning Province | 2012 - 2013 | Scientific literature | FPG ≥ 7 mmol/L or on treatment | Undiagnosed, Treated Envelope | Zhou X, Guan H, Zheng L, Li Z, Guo X, Yang H, Yu S, Sun G, Li W, Hu W, Guo L, Pan G, Xing L, Zhang Y, Sun Y. Prevalence and awareness of diabetes mellitus among a rural population in China: results from Liaoning Province. Diabet Med. 2015; 32(3): 332-42. |
| China | China - KaiLuan Study 2012-2014 | 2012 - 2014 | Microdata | FPG ≥ 7 mmol/L or on treatment | Undiagnosed, Diagnosed & Untreated, Treated Envelope, Treated & Suboptimal glycaemic levels, Treated & Optimal glycaemic levels | Kailuan General Hospital, Hebei United University. China - KaiLuan Study 2012-2014. |
| Colombia | Colombia - Santander STEPS Noncommunicable Disease Risk Factors Survey 2010 | 2010 - 2010 | Scientific literature | FPG ≥ 7 mmol/L or on treatment | Treated Envelope, Treated & Suboptimal glycaemic levels, Treated & Optimal glycaemic levels | Santander Center for Public Health (Colombia), Santander Ministry of Health (Colombia), World Health Organization (WHO). Colombia - Santander STEPS Noncommunicable Disease Risk Factors Survey 2010. |
| Comoros | Comoros STEPS Noncommunicable Disease Risk Factors Survey 2011 | 2011 - 2011 | Microdata | FPG ≥ 7 mmol/L or on treatment | Undiagnosed, Diagnosed & Untreated, Treated Envelope, Treated & Suboptimal glycaemic levels, Treated & Optimal glycaemic levels | World Health Organization (WHO). Comoros STEPS Noncommunicable Disease Risk Factors Survey 2011. |
| Congo | Congo - Brazzaville STEPS Noncommunicable Disease Risk Factors Survey 2004 | 2004 - 2004 | Microdata | FPG ≥ 7 mmol/L or on treatment | Undiagnosed, Diagnosed & Untreated, Treated Envelope, Treated & Suboptimal glycaemic levels, Treated & Optimal glycaemic levels | Ministry of Health (Congo, Rep.), World Health Organization (WHO). Congo - Brazzaville STEPS Noncommunicable Disease Risk Factors Survey 2004. |
| Cook Islands | Cook Islands STEPS Noncommunicable Disease Risk Factors Survey 2003-2004 | 2003 - 2004 | Microdata | FPG ≥ 7 mmol/L or on treatment | Undiagnosed, Diagnosed & Untreated, Treated Envelope, Treated & Suboptimal glycaemic levels, Treated & Optimal glycaemic levels | College of Medicine, Nursing and Health Sciences, Fiji National University, Ministry of Health (Cook Islands), World Health Organization (WHO). Cook Islands STEPS Noncommunicable Disease Risk Factors Survey 2003-2004. |
| Cook Islands | Cook Islands STEPS Noncommunicable Disease Risk Factors Survey 2013-2015 | 2013 - 2015 | Microdata | FPG ≥ 7 mmol/L or on treatment | Undiagnosed, Diagnosed & Untreated, Treated Envelope, Treated & Suboptimal glycaemic levels, Treated & Optimal glycaemic levels | Ministry of Health (Cook Islands), World Health Organization (WHO). Cook Islands STEPS Noncommunicable Disease Risk Factors Survey 2013-2015. |
| Costa Rica | Costa Rica Longevity and Healthy Aging Study 2004-2006 - ICPSR | 2004 - 2006 | Microdata | FPG ≥ 7 mmol/L or on treatment | Undiagnosed, Diagnosed & Untreated, Treated Envelope, Treated & Suboptimal glycaemic levels, Treated & Optimal glycaemic levels | Rosero-Bixby, Luis , Xinia FernÃ¡ndez, and William H. Dow. CRELES: Costa Rican Longevity and Healthy Aging Study, 2005 (Costa Rica Estudio de Longevidad y Envejecimiento Saludable) [Computer file]. ICPSR26681-v1. Ann Arbor, MI: Inter-university Consortium for Political and Social Research [distributor], 2010-07-21. doi:10.3886/ICPSR26681 |
| Czechia | Prevalence, awareness, treatment and control of hypertension, diabetes and hypercholesterolemia, and associated risk factors in the Czech Republic, Russia, Poland and Lithuania: a cross-sectional study | 2002 - 2005 | Scientific literature | FPG ≥ 7 mmol/L or on treatment | Undiagnosed, Diagnosed & Untreated, Treated Envelope, Treated & Suboptimal glycaemic levels, Treated & Optimal glycaemic levels | Lu W, Pikhart H, Tamosiunas A, Kubinova R, Capkova N, Malyutina S, PajÄ…k A, Bobak M. Prevalence, awareness, treatment and control of hypertension, diabetes and hypercholesterolemia, and associated risk factors in the Czech Republic, Russia, Poland and Lithuania: a cross-sectional study. BMC Public Health. 2022; 22(1): 883. |
| Democratic Republic of the Congo | Democratic Republic of the Congo - Kinshasa STEPS Noncommunicable Disease Risk Factors Survey 2005 | 2005 - 2005 | Microdata | FPG ≥ 7 mmol/L or on treatment | Undiagnosed, Diagnosed & Untreated, Treated Envelope, Treated & Suboptimal glycaemic levels, Treated & Optimal glycaemic levels | Ministry of Public Health (Congo, DR), World Health Organization (WHO). Democratic Republic of the Congo - Kinshasa STEPS Noncommunicable Disease Risk Factors Survey 2005. 2021. |
| Ecuador | Ecuador STEPS Noncommunicable Disease Risk Factors Survey 2018 | 2018 - 2018 | Microdata | FPG ≥ 7 mmol/L or on treatment | Undiagnosed, Diagnosed & Untreated, Treated Envelope, Treated & Suboptimal glycaemic levels, Treated & Optimal glycaemic levels | Ministry of Public Health (Ecuador), World Health Organization (WHO). Ecuador STEPS Noncommunicable Disease Risk Factors Survey 2018. Geneva, Switzerland: World Health Organization (WHO). |
| Egypt | Metabolic and cardiovascular risk profiles and hepatitis C virus infection in rural Egypt | 2002 - 2002 | Scientific literature | FPG ≥ 7 mmol/L or on treatment | Treated Envelope | Marzouk D, Sass J, Bakr I, El Hosseiny M, Abdel-Hamid M, Rekacewicz C, Chaturvedi N, Mohamed MK, Fontanet A. Metabolic and cardiovascular risk profiles and hepatitis C virus infection in rural Egypt. Gut. 2007; 56(8): 1105-10. |
| Egypt | Egypt STEPS Noncommunicable Disease Risk Factors Survey 2017 | 2017 - 2017 | Scientific literature | FPG ≥ 7 mmol/L or on treatment | Treated Envelope | Central Agency for Public Mobilization and Statistics (CAPMAS) (Egypt), Ministry of Health and Population (Egypt), World Health Organization (WHO). Egypt STEPS Noncommunicable Disease Risk Factors Survey 2017. |
| Eritrea | Eritrea STEPS Noncommunicable Disease Risk Factors Survey 2010 | 2010 - 2010 | Microdata | FPG ≥ 7 mmol/L or on treatment | Undiagnosed, Diagnosed & Untreated, Treated Envelope, Treated & Suboptimal glycaemic levels, Treated & Optimal glycaemic levels | World Health Organization (WHO). Eritrea STEPS Noncommunicable Disease Risk Factors Survey 2010. |
| Eswatini | Swaziland STEPS Noncommunicable Disease Risk Factors Survey 2007 | 2007 - 2007 | Microdata | FPG ≥ 7 mmol/L or on treatment | Undiagnosed, Diagnosed & Untreated, Treated Envelope, Treated & Suboptimal glycaemic levels, Treated & Optimal glycaemic levels | Ministry of Health (Swaziland), World Health Organization (WHO). Swaziland STEPS Noncommunicable Disease Risk Factors Survey 2007. |
| Eswatini | Swaziland STEPS Noncommunicable Disease Risk Factors Survey 2014 | 2014 - 2014 | Microdata | FPG ≥ 7 mmol/L or on treatment | Undiagnosed, Diagnosed & Untreated, Treated Envelope, Treated & Suboptimal glycaemic levels, Treated & Optimal glycaemic levels | Ministry of Health (Swaziland), World Health Organization (WHO). Swaziland STEPS Noncommunicable Disease Risk Factors Survey 2014. |
| Ethiopia | Ethiopia STEPS Noncommunicable Disease Risk Factors Survey 2015 | 2015 - 2015 | Scientific literature | FPG ≥ 7 mmol/L or on treatment | Treated Envelope | Ethiopian Public Health Institute (EPHI), World Health Organization (WHO). Ethiopia STEPS Noncommunicable Disease Risk Factors Survey 2015. |
| Ethiopia | Abnormal Glucose Metabolism and Associated Risk Factors Among Adults in Mekelle City, Ethiopia | 2019 - 2019 | Scientific literature | FPG ≥ 7 mmol/L or on treatment | Treated Envelope | Gebreegziabiher G, Belachew T, Tamiru D. Abnormal Glucose Metabolism and Associated Risk Factors Among Adults in Mekelle City, Ethiopia. Diabetes Metab Syndr Obes. 2020; 13: 4017-4032. |
| Ethiopia | Prevalence of Diabetes Mellitus, Prediabetes and Its Associated Factors in Dessie Town, Northeast Ethiopia: A Community-Based Study | 2019 - 2019 | Scientific literature | FPG ≥ 7 mmol/L or on treatment | Undiagnosed | Endris T, Worede A, Asmelash D. Prevalence of Diabetes Mellitus, Prediabetes and Its Associated Factors in Dessie Town, Northeast Ethiopia: A Community-Based Study. Diabetes Metab Syndr Obes. 2019; 12: 2799-2809. |
| Fiji | Fiji STEPS Noncommunicable Disease Risk Factors Survey 2002 | 2002 - 2002 | Microdata | FPG ≥ 7 mmol/L or on treatment | Undiagnosed, Diagnosed & Untreated, Treated Envelope, Treated & Suboptimal glycaemic levels, Treated & Optimal glycaemic levels | Australian Agency for International Development (AusAID), Fiji School of Medicine, Menzies Center for Population Health Research, University of Tasmania (Australia), Ministry of Health (Fiji), World Health Organization (WHO). Fiji STEPS Noncommunicable Disease Risk Factors Survey 2002. |
| Fiji | Fiji STEPS Noncommunicable Disease Risk Factors Survey 2011 | 2011 - 2011 | Microdata | FPG ≥ 7 mmol/L or on treatment | Undiagnosed, Diagnosed & Untreated, Treated Envelope, Treated & Suboptimal glycaemic levels, Treated & Optimal glycaemic levels | World Health Organization (WHO). Fiji STEPS Noncommunicable Disease Risk Factors Survey 2011. |
| Finland | Cardiovascular risk profile and use of statins at the age of 70 years: a comparison of two Finnish birth cohorts born 20 years apart | 1991 - 1992 | Scientific literature | FPG ≥ 7 mmol/L or on treatment | Treated Envelope | Upmeier E, Vire J, Korhonen MJ, Isoaho H, Lehtonen A, Arve S, Wuorela M, Viitanen M. Cardiovascular risk profile and use of statins at the age of 70 years: a comparison of two Finnish birth cohorts born 20 years apart. Age Ageing. 2016; 45(1): 84-90. |
| Finland | Cardiovascular risk profile and use of statins at the age of 70 years: a comparison of two Finnish birth cohorts born 20 years apart | 2011 - 2012 | Scientific literature | FPG ≥ 7 mmol/L or on treatment | Treated Envelope | Upmeier E, Vire J, Korhonen MJ, Isoaho H, Lehtonen A, Arve S, Wuorela M, Viitanen M. Cardiovascular risk profile and use of statins at the age of 70 years: a comparison of two Finnish birth cohorts born 20 years apart. Age Ageing. 2016; 45(1): 84-90. |
| France | France Three-City Cohort Study 1999-2001 | 1999 - 2001 | Scientific literature | FPG ≥ 7 mmol/L or on treatment | Treated Envelope | French Institute of Health and Medical Research (INSERM), University of Victor Segalen Bordeaux (France). France Three-City Cohort Study 1999-2001. |
| France | Low high density lipoprotein cholesterol: prevalence and associated risk-factors in a large French population | 2005 - 2006 | Scientific literature | FPG ≥ 7 mmol/L or on treatment | Treated Envelope | Rouvre M, Vol S, Gusto G, Born C, Lantieri O, Tichet J, Lecomte P. Low high density lipoprotein cholesterol: prevalence and associated risk-factors in a large French population. Ann Epidemiol. 2011; 21(2): 118–27. |
| Georgia | Georgia STEPS Noncommunicable Disease Risk Factors Survey 2016 | 2016 - 2016 | Microdata | FPG ≥ 7 mmol/L or on treatment | Undiagnosed, Diagnosed & Untreated, Treated Envelope, Treated & Suboptimal glycaemic levels, Treated & Optimal glycaemic levels | National Center for Disease Control and Public Health (Georgia). Georgia STEPS Noncommunicable Disease Risk Factors Survey 2016. |
| Ghana | Ghana - Greater Accra Region STEPS Noncommunicable Disease Risk Factors Survey 2006 | 2006 - 2006 | Microdata | FPG ≥ 7 mmol/L or on treatment | Undiagnosed, Diagnosed & Untreated, Treated Envelope, Treated & Suboptimal glycaemic levels, Treated & Optimal glycaemic levels | Ghana Health Service, World Health Organization (WHO). Ghana - Greater Accra Region STEPS Noncommunicable Disease Risk Factors Survey 2006. |
| Guatemala | Guatemala STEPS Noncommunicable Disease Risk Factors Survey 2015 | 2015 - 2015 | Scientific literature | FPG ≥ 7 mmol/L or on treatment | Treated Envelope | World Health Organization (WHO). Guatemala STEPS Noncommunicable Disease Risk Factors Survey 2015. |
| Guinea | Guinea STEPS Noncommunicable Disease Risk Factors Survey 2009 | 2009 - 2009 | Microdata | FPG ≥ 7 mmol/L or on treatment | Undiagnosed, Diagnosed & Untreated, Treated Envelope, Treated & Suboptimal glycaemic levels, Treated & Optimal glycaemic levels | World Health Organization (WHO). Guinea STEPS Noncommunicable Disease Risk Factors Survey 2009. |
| Guyana | Guyana STEPS Noncommunicable Disease Risk Factors Survey 2016 | 2016 - 2016 | Microdata | FPG ≥ 7 mmol/L or on treatment | Undiagnosed, Diagnosed & Untreated, Treated Envelope, Treated & Suboptimal glycaemic levels, Treated & Optimal glycaemic levels | Ministry of Health (Guyana), World Health Organization (WHO). Guyana STEPS Noncommunicable Disease Risk Factors Survey 2016. |
| India | Burden of Hypertension and Diabetes among Urban Population Aged ≥ 60 years in South Delhi: A Community Based Study | 2002 - 2002 | Scientific literature | FPG ≥ 7 mmol/L or on treatment | Treated Envelope, Treated & Suboptimal glycaemic levels, Treated & Optimal glycaemic levels | Goswami AK, Gupta SK, Kalaivani M, Nongkynrih B, Pandav CS. Burden of Hypertension and Diabetes among Urban Population Aged ≥ 60 years in South Delhi: A Community Based Study. J Clin Diagn Res. 2016; 10(3): LC01-05. |
| India | India - Mahārāshtra Central India Eye and Medical Study 2006-2008 | 2006 - 2008 | Microdata | HbA1c ≥ 6.5% or on treatment | Undiagnosed, Diagnosed & Untreated, Treated Envelope | Nangia V, Jonas JB, Sinha A, Matin A, Kulkarni M, Panda-Jonas S. Ocular axial length and its associations in an adult population of Central Rural India. The Central India Eye and Medical Study [Unpublished data]. *Ophthalmology* 2010;117(7):1360-6. |
| Iran (Islamic Republic of) | Iran - Pars Cohort Study | 2012 - 2012 | Microdata | FPG ≥ 7 mmol/L or on treatment | Undiagnosed, Diagnosed & Untreated, Treated Envelope, Treated & Suboptimal glycaemic levels, Treated & Optimal glycaemic levels | Digestive Diseases Research Institute (DDRI) (Iran), Shiraz University of Medical Sciences. Iran - Pars Cohort Study. |
| Iran (Islamic Republic of) | Nationwide Prevalence of Diabetes and Prediabetes and Associated Risk Factors Among Iranian Adults: Analysis of Data from PERSIAN Cohort Study | 2014 - 2020 | Scientific literature | FPG ≥ 7 mmol/L or on treatment | Undiagnosed, Treated & Optimal glycaemic levels | Khamseh ME, Sepanlou SG, Hashemi-Madani N, Joukar F, Mehrparvar AH, Faramarzi E, Okati-Aliabad H, Rahimi Z, Rezaianzadeh A, Homayounfar R, Moradpour F, Valizadeh N, Kheirandish M, Zaboli E, Moslem A, Ahmadi A, Hamzeh B, Harooni J, Pourfarzi F, Abolghasemi MR, Safarpour AR, Aminisani N, Mohammadi Z, Eghtesad S, Poustchi H, Malekzadeh R. Nationwide Prevalence of Diabetes and Prediabetes and Associated Risk Factors Among Iranian Adults: Analysis of Data from PERSIAN Cohort Study. Diabetes Ther. 2021; 12(11): 2921-2938. |
| Iraq | Iraq STEPS Noncommunicable Disease Risk Factors Survey 2015 | 2015 - 2015 | Microdata | FPG ≥ 7 mmol/L or on treatment | Undiagnosed, Diagnosed & Untreated, Treated Envelope, Treated & Suboptimal glycaemic levels, Treated & Optimal glycaemic levels | Ministry of Health (Iraq), Ministry of Planning (Iraq), World Health Organization (WHO). Iraq STEPS Noncommunicable Disease Risk Factors Survey 2015. |
| Japan | Japan National Health and Nutrition Survey Diabetes Status by Age, Sex, and Prefecture 1990-2017 | 2005 - 2005 | Collaborator-provided tabulations | HbA1c ≥ 6.5% or on treatment | Treated Envelope | Ministry of Health, Labour and Welfare (Japan), University of Tokyo. Japan National Health and Nutrition Survey Diabetes Status by Age, Sex, and Prefecture 1990-2017. |
| Japan | Japan National Health and Nutrition Survey Diabetes Status by Age, Sex, and Prefecture 1990-2017 | 2006 - 2006 | Collaborator-provided tabulations | HbA1c ≥ 6.5% or on treatment | Treated Envelope | Ministry of Health, Labour and Welfare (Japan), University of Tokyo. Japan National Health and Nutrition Survey Diabetes Status by Age, Sex, and Prefecture 1990-2017. |
| Japan | Japan National Health and Nutrition Survey Diabetes Status by Age, Sex, and Prefecture 1990-2017 | 2007 - 2007 | Collaborator-provided tabulations | HbA1c ≥ 6.5% or on treatment | Treated Envelope | Ministry of Health, Labour and Welfare (Japan), University of Tokyo. Japan National Health and Nutrition Survey Diabetes Status by Age, Sex, and Prefecture 1990-2017. |
| Japan | Japan National Health and Nutrition Survey Diabetes Status by Age, Sex, and Prefecture 1990-2017 | 2008 - 2008 | Collaborator-provided tabulations | HbA1c ≥ 6.5% or on treatment | Treated Envelope | Ministry of Health, Labour and Welfare (Japan), University of Tokyo. Japan National Health and Nutrition Survey Diabetes Status by Age, Sex, and Prefecture 1990-2017. |
| Japan | Japan National Health and Nutrition Survey Diabetes Status by Age, Sex, and Prefecture 1990-2017 | 2009 - 2009 | Collaborator-provided tabulations | HbA1c ≥ 6.5% or on treatment | Treated Envelope | Ministry of Health, Labour and Welfare (Japan), University of Tokyo. Japan National Health and Nutrition Survey Diabetes Status by Age, Sex, and Prefecture 1990-2017. |
| Japan | Japan National Health and Nutrition Survey Diabetes Status by Age, Sex, and Prefecture 1990-2017 | 2010 - 2010 | Collaborator-provided tabulations | HbA1c ≥ 6.5% or on treatment | Treated Envelope | Ministry of Health, Labour and Welfare (Japan), University of Tokyo. Japan National Health and Nutrition Survey Diabetes Status by Age, Sex, and Prefecture 1990-2017. |
| Japan | Japan National Health and Nutrition Survey Diabetes Status by Age, Sex, and Prefecture 1990-2017 | 2011 - 2011 | Collaborator-provided tabulations | HbA1c ≥ 6.5% or on treatment | Treated Envelope | Ministry of Health, Labour and Welfare (Japan), University of Tokyo. Japan National Health and Nutrition Survey Diabetes Status by Age, Sex, and Prefecture 1990-2017. |
| Japan | Japan National Health and Nutrition Survey Diabetes Status by Age, Sex, and Prefecture 1990-2017 | 2012 - 2012 | Collaborator-provided tabulations | HbA1c ≥ 6.5% or on treatment | Treated Envelope | Ministry of Health, Labour and Welfare (Japan), University of Tokyo. Japan National Health and Nutrition Survey Diabetes Status by Age, Sex, and Prefecture 1990-2017. |
| Japan | Japan National Health and Nutrition Survey Diabetes Status by Age, Sex, and Prefecture 1990-2017 | 2014 - 2014 | Collaborator-provided tabulations | HbA1c ≥ 6.5% or on treatment | Treated Envelope | Ministry of Health, Labour and Welfare (Japan), University of Tokyo. Japan National Health and Nutrition Survey Diabetes Status by Age, Sex, and Prefecture 1990-2017. |
| Japan | Japan National Health and Nutrition Survey Diabetes Status by Age, Sex, and Prefecture 1990-2017 | 2015 - 2015 | Collaborator-provided tabulations | HbA1c ≥ 6.5% or on treatment | Treated Envelope | Ministry of Health, Labour and Welfare (Japan), University of Tokyo. Japan National Health and Nutrition Survey Diabetes Status by Age, Sex, and Prefecture 1990-2017. |
| Japan | Japan National Health and Nutrition Survey Diabetes Status by Age, Sex, and Prefecture 1990-2017 | 2016 - 2016 | Collaborator-provided tabulations | HbA1c ≥ 6.5% or on treatment | Treated Envelope | Ministry of Health, Labour and Welfare (Japan), University of Tokyo. Japan National Health and Nutrition Survey Diabetes Status by Age, Sex, and Prefecture 1990-2017. |
| Japan | Japan National Health and Nutrition Survey Diabetes Status by Age, Sex, and Prefecture 1990-2017 | 2017 - 2017 | Collaborator-provided tabulations | HbA1c ≥ 6.5% or on treatment | Treated Envelope | Ministry of Health, Labour and Welfare (Japan), University of Tokyo. Japan National Health and Nutrition Survey Diabetes Status by Age, Sex, and Prefecture 1990-2017. |
| Japan | Japan National Health and Nutrition Survey 2018 | 2018 - 2018 | Collaborator-provided tabulations | HbA1c ≥ 6.5% or on treatment | Treated Envelope | Ministry of Health, Labour and Welfare (Japan), National Institute of Health and Nutrition (Japan). Japan National Health and Nutrition Survey 2018. |
| Japan | Japan National Health and Nutrition Survey 2019 | 2019 - 2019 | Collaborator-provided tabulations | HbA1c ≥ 6.5% or on treatment | Treated Envelope | Ministry of Health, Labour and Welfare (Japan), National Institute of Health and Nutrition (Japan). Japan National Health and Nutrition Survey 2019. |
| Jordan | Jordan STEPS Noncommunicable Disease Risk Factors Survey 2007 | 2007 - 2007 | Microdata | FPG ≥ 7 mmol/L or on treatment | Undiagnosed, Diagnosed & Untreated, Treated Envelope, Treated & Suboptimal glycaemic levels, Treated & Optimal glycaemic levels | Centers for Disease Control and Prevention (CDC), Ministry of Health (Jordan), World Health Organization (WHO). Jordan STEPS Noncommunicable Disease Risk Factors Survey 2007. |
| Jordan | Jordan Cardiovascular Disease Risk Factor Survey 2009 | 2009 - 2009 | Microdata | FPG ≥ 7 mmol/L or on treatment | Undiagnosed, Diagnosed & Untreated, Treated Envelope, Treated & Suboptimal glycaemic levels, Treated & Optimal glycaemic levels | Jordan University of Science and Technology, National Center for Diabetes, Endocrinology, and Genetics (NCDEG) (Jordan). Jordan Cardiovascular Disease Risk Factor Survey 2009. |
| Jordan | Jordan Cardiovascular Disease Risk Factor Survey 2017 | 2017 - 2017 | Microdata | FPG ≥ 7 mmol/L or on treatment | Undiagnosed, Diagnosed & Untreated, Treated Envelope, Treated & Suboptimal glycaemic levels, Treated & Optimal glycaemic levels | Jordan University of Science and Technology, National Center for Diabetes, Endocrinology, and Genetics (NCDEG) (Jordan). Jordan Cardiovascular Disease Risk Factor Survey 2017. |
| Jordan | Jordan STEPS Noncommunicable Disease Risk Factors Survey 2019 | 2019 - 2019 | Microdata | FPG ≥ 7 mmol/L or on treatment | Undiagnosed, Diagnosed & Untreated, Treated Envelope, Treated & Suboptimal glycaemic levels, Treated & Optimal glycaemic levels | Center for Strategic Studies, University of Jordan, Ministry of Health (Jordan), World Health Organization (WHO). Jordan STEPS Noncommunicable Disease Risk Factors Survey 2019. WHO NCD Microdata Repository, 2021. |
| Kazakhstan | Kazakhstan - Shymkent STEPS Noncommunicable Disease Risk Factors Survey 2015 | 2015 - 2015 | Microdata | FPG ≥ 7 mmol/L or on treatment | Undiagnosed, Diagnosed & Untreated, Treated Envelope, Treated & Suboptimal glycaemic levels, Treated & Optimal glycaemic levels | World Health Organization (WHO). Kazakhstan - Shymkent STEPS Noncommunicable Disease Risk Factors Survey 2015. |
| Kenya | Kenya STEPS Noncommunicable Disease Risk Factors Survey 2015 | 2015 - 2015 | Microdata | FPG ≥ 7 mmol/L or on treatment | Undiagnosed, Diagnosed & Untreated, Treated Envelope, Treated & Suboptimal glycaemic levels, Treated & Optimal glycaemic levels | Kenya National Bureau of Statistics, Ministry of Health (Kenya), World Health Organization (WHO). Kenya STEPS Noncommunicable Disease Risk Factors Survey 2015. |
| Kiribati | Kiribati STEPS Noncommunicable Disease Risk Factors Survey 2004-2006 | 2004 - 2006 | Microdata | FPG ≥ 7 mmol/L or on treatment | Undiagnosed, Diagnosed & Untreated, Treated Envelope, Treated & Suboptimal glycaemic levels, Treated & Optimal glycaemic levels | Ministry of Health and Medical Services (Kiribati), World Health Organization (WHO). Kiribati STEPS Noncommunicable Disease Risk Factors Survey 2004-2006. |
| Kiribati | Kiribati STEPS Noncommunicable Disease Risk Factors Survey 2016 | 2015 - 2016 | Microdata | FPG ≥ 7 mmol/L or on treatment | Undiagnosed, Diagnosed & Untreated, Treated Envelope, Treated & Suboptimal glycaemic levels, Treated & Optimal glycaemic levels | Ministry of Health and Medical Services (Kiribati), World Health Organization (WHO). Kiribati STEPS Noncommunicable Disease Risk Factors Survey 2016. |
| Kuwait | Kuwait STEPS Noncommunicable Disease Risk Factors Survey 2006 | 2006 - 2006 | Microdata | FPG ≥ 7 mmol/L or on treatment | Undiagnosed, Diagnosed & Untreated, Treated Envelope, Treated & Suboptimal glycaemic levels, Treated & Optimal glycaemic levels | Ministry of Health (Kuwait), World Health Organization (WHO). Kuwait STEPS Noncommunicable Disease Risk Factors Survey 2006. |
| Kuwait | Kuwait STEPS Noncommunicable Disease Risk Factors Survey 2014 | 2014 - 2014 | Microdata | FPG ≥ 7 mmol/L or on treatment | Undiagnosed, Diagnosed & Untreated, Treated Envelope, Treated & Suboptimal glycaemic levels, Treated & Optimal glycaemic levels | Ministry of Health (Kuwait), World Health Organization (WHO). Kuwait STEPS Noncommunicable Disease Risk Factors Survey 2014. |
| Kyrgyzstan | Kyrgyzstan STEPS Noncommunicable Disease Risk Factors Survey 2013 | 2013 - 2013 | Microdata | FPG ≥ 7 mmol/L or on treatment | Undiagnosed, Diagnosed & Untreated, Treated Envelope, Treated & Suboptimal glycaemic levels, Treated & Optimal glycaemic levels | Ministry of Health (Kyrgyzstan), World Health Organization (WHO). Kyrgyzstan STEPS Noncommunicable Disease Risk Factors Survey 2013. |
| Lao People's Democratic Republic | Laos STEPS Noncommunicable Disease Risk Factors Survey 2013 | 2013 - 2013 | Microdata | FPG ≥ 7 mmol/L or on treatment | Undiagnosed, Diagnosed & Untreated, Treated Envelope, Treated & Suboptimal glycaemic levels, Treated & Optimal glycaemic levels | World Health Organization (WHO). Laos STEPS Noncommunicable Disease Risk Factors Survey 2013. WHO NCD Microdata Repository, 2019. |
| Lebanon | Lebanon STEPS Noncommunicable Disease Risk Factors Survey 2008-2009 | 2008 - 2009 | Microdata | FPG ≥ 7 mmol/L or on treatment | Undiagnosed, Diagnosed & Untreated, Treated Envelope, Treated & Suboptimal glycaemic levels, Treated & Optimal glycaemic levels | American University of Beirut, World Health Organization (WHO). Lebanon STEPS Noncommunicable Disease Risk Factors Survey 2008-2009. |
| Lebanon | Lebanon STEPS Noncommunicable Disease Risk Factors Survey 2016-2017 | 2016 - 2017 | Microdata | FPG ≥ 7 mmol/L or on treatment | Undiagnosed, Diagnosed & Untreated, Treated Envelope, Treated & Suboptimal glycaemic levels, Treated & Optimal glycaemic levels | Ministry of Public Health (Lebanon), World Health Organization (WHO). Lebanon STEPS Noncommunicable Disease Risk Factors Survey 2016-2017. |
| Lesotho | Lesotho STEPS Noncommunicable Disease Risk Factors Survey 2012 | 2012 - 2012 | Microdata | FPG ≥ 7 mmol/L or on treatment | Undiagnosed, Diagnosed & Untreated, Treated Envelope, Treated & Suboptimal glycaemic levels, Treated & Optimal glycaemic levels | Ministry of Health and Social Welfare (Lesotho), World Health Organization (WHO). Lesotho STEPS Noncommunicable Disease Risk Factors Survey 2012 . |
| Liberia | Liberia STEPS Noncommunicable Disease Risk Factors Survey 2011 | 2011 - 2011 | Microdata | FPG ≥ 7 mmol/L or on treatment | Undiagnosed, Diagnosed & Untreated, Treated Envelope, Treated & Suboptimal glycaemic levels, Treated & Optimal glycaemic levels | World Health Organization (WHO). Liberia STEPS Noncommunicable Disease Risk Factors Survey 2011. |
| Liberia | Liberia STEPS Noncommunicable Disease Risk Factors Survey 2022 | 2022 - 2022 | Scientific literature | FPG ≥ 7 mmol/L or on treatment | Treated Envelope | Ministry of Health (Liberia), World Health Organization (WHO). Liberia STEPS Noncommunicable Disease Risk Factors Survey 2022. |
| Libya | Libya STEPS Noncommunicable Disease Risk Factors Survey 2009 | 2009 - 2009 | Microdata | FPG ≥ 7 mmol/L or on treatment | Undiagnosed, Diagnosed & Untreated, Treated Envelope, Treated & Suboptimal glycaemic levels, Treated & Optimal glycaemic levels | Secretariat of Health and Environment (Libya), World Health Organization (WHO). Libya STEPS Noncommunicable Disease Risk Factors Survey 2009. |
| Lithuania | Prevalence, awareness, treatment and control of hypertension, diabetes and hypercholesterolemia, and associated risk factors in the Czech Republic, Russia, Poland and Lithuania: a cross-sectional study | 2005 - 2008 | Scientific literature | FPG ≥ 7 mmol/L or on treatment | Undiagnosed, Diagnosed & Untreated, Treated Envelope, Treated & Suboptimal glycaemic levels, Treated & Optimal glycaemic levels | Lu W, Pikhart H, Tamosiunas A, Kubinova R, Capkova N, Malyutina S, PajÄ…k A, Bobak M. Prevalence, awareness, treatment and control of hypertension, diabetes and hypercholesterolemia, and associated risk factors in the Czech Republic, Russia, Poland and Lithuania: a cross-sectional study. BMC Public Health. 2022; 22(1): 883. |
| Luxembourg | First nationwide survey on cardiovascular risk factors in Grand-Duchy of Luxembourg (ORISCAV-LUX) | 2007 - 2009 | Scientific literature | FPG ≥ 7 mmol/L or on treatment | Treated Envelope | Alkerwi A, Sauvageot N, Donneau A-F, Lair M-L, Couffignal S, Beissel J, Delagardelle C, Wagener Y, Albert A, Guillaume M. First nationwide survey on cardiovascular risk factors in Grand-Duchy of Luxembourg (ORISCAV-LUX). BMC Public Health. 2010; 10(1): 468. |
| Malawi | Malawi STEPS Noncommunicable Disease Risk Factors Survey 2009 | 2009 - 2009 | Microdata | FPG ≥ 7 mmol/L or on treatment | Undiagnosed, Diagnosed & Untreated, Treated Envelope, Treated & Suboptimal glycaemic levels, Treated & Optimal glycaemic levels | Ministry of Health (Malawi), World Health Organization (WHO). Malawi STEPS Noncommunicable Disease Risk Factors Survey 2009. |
| Malawi | Malawi STEPS Noncommunicable Disease Risk Factors Survey 2017 | 2017 - 2017 | Microdata | FPG ≥ 7 mmol/L or on treatment | Undiagnosed, Diagnosed & Untreated, Treated Envelope, Treated & Suboptimal glycaemic levels, Treated & Optimal glycaemic levels | World Health Organization (WHO). Malawi STEPS Noncommunicable Disease Risk Factors Survey 2017. |
| Malaysia | Variation in the prevalence, awareness, and control of diabetes in a multiethnic population: a nationwide population study in Malaysia | 2004 - 2004 | Scientific literature | FPG ≥ 7 mmol/L or on treatment | Undiagnosed, Diagnosed & Untreated, Treated Envelope, Treated & Suboptimal glycaemic levels, Treated & Optimal glycaemic levels | Rampal S, Rampal L, Rahmat R, Zain AM, Yap YG, Mohamed M, Taha M. Variation in the prevalence, awareness, and control of diabetes in a multiethnic population: a nationwide population study in Malaysia. Asia Pac J Public Health. 2010; 22(2): 194-202. |
| Maldives | Maldives STEPS Noncommunicable Disease Risk Factors Survey 2020-2021 | 2020 - 2021 | Scientific literature | FPG ≥ 7 mmol/L or on treatment | Treated Envelope | Health Protection Agency (Maldives), The Maldives National University, World Health Organization (WHO). Maldives STEPS Noncommunicable Disease Risk Factors Survey 2020-2021. 2022. |
| Mali | Mali STEPS Noncommunicable Disease Risk Factors Survey 2007 | 2007 - 2007 | Microdata | FPG ≥ 7 mmol/L or on treatment | Undiagnosed, Diagnosed & Untreated, Treated Envelope, Treated & Suboptimal glycaemic levels, Treated & Optimal glycaemic levels | World Health Organization (WHO). Mali STEPS Noncommunicable Disease Risk Factors Survey 2007. |
| Mali | Mali - Kati, Ouéléssébougou, Koulikoro, Ségou and Bamako District STEPS Noncommunicable Disease Risk Factors Survey 2013 | 2013 - 2013 | Microdata | FPG ≥ 7 mmol/L or on treatment | Undiagnosed, Diagnosed & Untreated, Treated Envelope, Treated & Suboptimal glycaemic levels, Treated & Optimal glycaemic levels | Ministry of Health (Mali), World Health Organization (WHO). Mali - Kati, Ouéléssébougou, Koulikoro, Ségou and Bamako District STEPS Noncommunicable Disease Risk Factors Survey 2013. WHO NCD Microdata Repository, 2019. |
| Marshall Islands | Marshall Islands STEPS Noncommunicable Disease Risk Factors Survey 2002 | 2002 - 2002 | Microdata | FPG ≥ 7 mmol/L or on treatment | Undiagnosed, Diagnosed & Untreated, Treated Envelope, Treated & Suboptimal glycaemic levels, Treated & Optimal glycaemic levels | Fiji School of Medicine, Menzies Center for Population Health Research, University of Tasmania (Australia), Ministry of Health (Marshall Islands), World Health Organization (WHO). Marshall Islands STEPS Noncommunicable Disease Risk Factors Survey 2002. |
| Marshall Islands | Marshall Islands STEPS Noncommunicable Disease Risk Factors Survey 2017-2018 | 2017 - 2018 | Microdata | FPG ≥ 7 mmol/L or on treatment | Undiagnosed, Diagnosed & Untreated, Treated Envelope, Treated & Suboptimal glycaemic levels, Treated & Optimal glycaemic levels | Ministry of Health and Human Services (Marshall Islands), World Health Organization (WHO). Marshall Islands STEPS Noncommunicable Disease Risk Factors Survey 2017-2018 . Geneva, Switzerland: World Health Organization (WHO), 2020. |
| Marshall Islands | The Protection Gap - Diagnosis, Treatment Status, and Disease Control for People with Diabetes and Hypertension in the US-Affiliated Pacific Islands | 2018 - 2018 | Scientific literature | FPG ≥ 7 mmol/L or on treatment | Undiagnosed, Diagnosed & Untreated, Treated Envelope, Treated & Suboptimal glycaemic levels, Treated & Optimal glycaemic levels | Durand AM, Cash HL, Reichhardt MLE, Taulung L, Tolenoa NM. The Protection Gap - Diagnosis, Treatment Status, and Disease Control for People with Diabetes and Hypertension in the US-Affiliated Pacific Islands. Hawaii J Health Soc Welf. 2023; 82(3): 66-71. |
| Mauritania | Mauritania - Nouakchott STEPS Noncommunicable Disease Risk Factors Survey 2006 | 2006 - 2006 | Microdata | FPG ≥ 7 mmol/L or on treatment | Undiagnosed, Diagnosed & Untreated, Treated Envelope, Treated & Suboptimal glycaemic levels, Treated & Optimal glycaemic levels | Ministry of Health (Mauritania), World Health Organization (WHO). Mauritania - Nouakchott STEPS Noncommunicable Disease Risk Factors Survey 2006. |
| Mexico | Mexico National Health Survey 1999-2000 | 1999 - 2000 | Microdata | FPG ≥ 7 mmol/L or on treatment | Undiagnosed, Diagnosed & Untreated, Treated Envelope, Treated & Suboptimal glycaemic levels, Treated & Optimal glycaemic levels | National Institute of Public Health (Mexico). Mexico National Health Survey 1999-2000. |
| Mexico | Mexico National Survey of Health and Nutrition 2005-2006 | 2005 - 2006 | Microdata | FPG ≥ 7 mmol/L or on treatment | Undiagnosed, Diagnosed & Untreated, Treated Envelope, Treated & Suboptimal glycaemic levels, Treated & Optimal glycaemic levels | National Institute of Public Health (Mexico). Mexico National Survey of Health and Nutrition 2005-2006. Cuernavaca, Mexico: National Institute of Public Health (Mexico). |
| Mexico | Mexico National Survey of Health and Nutrition 2011-2012 | 2011 - 2012 | Microdata | FPG ≥ 7 mmol/L or on treatment | Undiagnosed, Diagnosed & Untreated, Treated Envelope, Treated & Suboptimal glycaemic levels, Treated & Optimal glycaemic levels | National Institute of Public Health (Mexico). Mexico National Survey of Health and Nutrition 2011-2012. Cuernavaca, Mexico: National Institute of Public Health (Mexico). |
| Mexico | Mexico National Survey of Health and Nutrition Mid-way 2016 | 2016 - 2016 | Microdata | FPG ≥ 7 mmol/L or on treatment | Undiagnosed, Diagnosed & Untreated, Treated Envelope, Treated & Suboptimal glycaemic levels, Treated & Optimal glycaemic levels | Ministry of Health (Mexico), National Institute of Public Health (Mexico). Mexico National Survey of Health and Nutrition Mid-way 2016. |
| Mexico | Mexico National Survey of Health and Nutrition (ENSANUT) 2018-2019 | 2018 - 2019 | Microdata | FPG ≥ 7 mmol/L or on treatment | Undiagnosed, Diagnosed & Untreated, Treated Envelope, Treated & Suboptimal glycaemic levels, Treated & Optimal glycaemic levels | National Institute of Public Health (Mexico), National Institute of Statistics and Geography (INEGI) (Mexico). Mexico National Survey of Health and Nutrition (ENSANUT) 2018-2019. Cuernavaca, Mexico: National Institute of Public Health (Mexico). |
| Mexico | Mexico Continuous National Health and Nutrition Survey 2022 | 2022 - 2022 | Microdata | FPG ≥ 7 mmol/L or on treatment | Undiagnosed, Diagnosed & Untreated, Treated Envelope, Treated & Suboptimal glycaemic levels, Treated & Optimal glycaemic levels | Ministry of Health (Mexico), National Institute of Public Health (Mexico). Mexico Continuous National Health and Nutrition Survey 2022. Cuernavaca, Mexico: National Institute of Public Health (Mexico), 2023. |
| Micronesia (Federated States of) | Micronesia - Pohnpei STEPS Noncommunicable Disease Risk Factors Survey 2002 | 2002 - 2002 | Microdata | FPG ≥ 7 mmol/L or on treatment | Undiagnosed, Diagnosed & Untreated, Treated Envelope, Treated & Suboptimal glycaemic levels, Treated & Optimal glycaemic levels | Centre for Physical Activity and Health, University of Sydney (Australia), Department of Health and Social Affairs (Micronesia), Fiji School of Medicine, Micronesia Human Resources Development Center, Pohnpei State Department of Health Services, World Health Organization (WHO). Micronesia - Pohnpei STEPS Noncommunicable Disease Risk Factors Survey 2002. |
| Micronesia (Federated States of) | Micronesia - Chuuk STEPS Noncommunicable Disease Risk Factors Survey 2006 | 2006 - 2006 | Microdata | FPG ≥ 7 mmol/L or on treatment | Undiagnosed, Diagnosed & Untreated, Treated Envelope, Treated & Suboptimal glycaemic levels, Treated & Optimal glycaemic levels | Chuuk Department of Health Services (Micronesia), Department of Health and Social Affairs (Micronesia), World Health Organization (WHO). Micronesia - Chuuk STEPS Noncommunicable Disease Risk Factors Survey 2006. |
| Micronesia (Federated States of) | Micronesia - Pohnpei STEPS Noncommunicable Disease Risk Factors Survey 2008 | 2008 - 2008 | Microdata | FPG ≥ 7 mmol/L or on treatment | Undiagnosed, Diagnosed & Untreated, Treated Envelope, Treated & Suboptimal glycaemic levels, Treated & Optimal glycaemic levels | World Health Organization (WHO). Micronesia - Pohnpei STEPS Noncommunicable Disease Risk Factors Survey 2008 . |
| Micronesia (Federated States of) | Micronesia - Kosrae STEPS Noncommunicable Disease Risk Factors Survey 2009 | 2009 - 2009 | Microdata | FPG ≥ 7 mmol/L or on treatment | Undiagnosed, Diagnosed & Untreated, Treated Envelope, Treated & Suboptimal glycaemic levels, Treated & Optimal glycaemic levels | World Health Organization (WHO). Micronesia - Kosrae STEPS Noncommunicable Disease Risk Factors Survey 2009. |
| Micronesia (Federated States of) | Micronesia - Yap STEPS Noncommunicable Disease Risk Factors Survey 2009 | 2009 - 2009 | Microdata | FPG ≥ 7 mmol/L or on treatment | Undiagnosed, Diagnosed & Untreated, Treated Envelope, Treated & Suboptimal glycaemic levels, Treated & Optimal glycaemic levels | World Health Organization (WHO). Micronesia - Yap STEPS Noncommunicable Disease Risk Factors Survey 2009. |
| Micronesia (Federated States of) | Micronesia - Chuuk STEPS Noncommunicable Disease Risk Factors Survey 2016 | 2016 - 2016 | Microdata | FPG ≥ 7 mmol/L or on treatment | Undiagnosed, Diagnosed & Untreated, Treated Envelope, Treated & Suboptimal glycaemic levels, Treated & Optimal glycaemic levels | Chuuk Department of Health Services (Micronesia), Department of Health and Social Affairs (Micronesia), World Health Organization (WHO). Micronesia - Chuuk STEPS Noncommunicable Disease Risk Factors Survey 2016. WHO NCD Microdata Repository, 2019. |
| Micronesia (Federated States of) | The Protection Gap - Diagnosis, Treatment Status, and Disease Control for People with Diabetes and Hypertension in the US-Affiliated Pacific Islands | 2019 - 2019 | Scientific literature | FPG ≥ 7 mmol/L or on treatment | Undiagnosed, Diagnosed & Untreated, Treated Envelope, Treated & Suboptimal glycaemic levels, Treated & Optimal glycaemic levels | Durand AM, Cash HL, Reichhardt MLE, Taulung L, Tolenoa NM. The Protection Gap - Diagnosis, Treatment Status, and Disease Control for People with Diabetes and Hypertension in the US-Affiliated Pacific Islands. Hawaii J Health Soc Welf. 2023; 82(3): 66-71. |
| Mongolia | Mongolia STEPS Noncommunicable Disease Risk Factors Survey 2005 | 2005 - 2005 | Microdata | FPG ≥ 7 mmol/L or on treatment | Undiagnosed, Diagnosed & Untreated, Treated Envelope, Treated & Suboptimal glycaemic levels, Treated & Optimal glycaemic levels | World Health Organization (WHO), Ministry of Health (Mongolia), National Medical Research Institute (Mongolia), Health Sciences University (Mongolia), National Oncology Center of Mongolia (Mongolia). Mongolia STEPS Noncommunicable Disease Risk Factors Survey 2005. |
| Mongolia | Mongolia STEPS Noncommunicable Disease Risk Factors Survey 2009 | 2009 - 2009 | Microdata | FPG ≥ 7 mmol/L or on treatment | Undiagnosed, Diagnosed & Untreated, Treated Envelope, Treated & Suboptimal glycaemic levels, Treated & Optimal glycaemic levels | World Health Organization (WHO), Ministry of Health (Mongolia), National Medical Research Institute (Mongolia), Health Sciences University (Mongolia), National Oncology Center of Mongolia (Mongolia). Mongolia STEPS Noncommunicable Disease Risk Factors Survey 2009. |
| Mongolia | Mongolia STEPS Noncommunicable Disease Risk Factors Survey 2013 | 2013 - 2013 | Microdata | FPG ≥ 7 mmol/L or on treatment | Undiagnosed, Diagnosed & Untreated, Treated Envelope, Treated & Suboptimal glycaemic levels, Treated & Optimal glycaemic levels | Public Health Institute, Ministry of Health (Mongolia), World Health Organization (WHO). Mongolia STEPS Noncommunicable Disease Risk Factors Survey 2013. |
| Mongolia | Mongolia STEPS Noncommunicable Disease Risk Factors Survey 2019 | 2019 - 2019 | Microdata | FPG ≥ 7 mmol/L or on treatment | Undiagnosed, Diagnosed & Untreated, Treated Envelope, Treated & Suboptimal glycaemic levels, Treated & Optimal glycaemic levels | Public Health Institute, Ministry of Health (Mongolia), World Health Organization (WHO). Mongolia STEPS Noncommunicable Disease Risk Factors Survey 2019. Geneva, Switzerland: World Health Organization (WHO). |
| Morocco | Morocco STEPS Noncommunicable Disease Risk Factors Survey 2017 | 2017 - 2017 | Microdata | FPG ≥ 7 mmol/L or on treatment | Undiagnosed, Diagnosed & Untreated, Treated Envelope, Treated & Suboptimal glycaemic levels, Treated & Optimal glycaemic levels | Ministry of Health (Morocco), World Health Organization (WHO). Morocco STEPS Noncommunicable Disease Risk Factors Survey 2017. |
| Mozambique | Mozambique STEPS Noncommunicable Disease Risk Factors Survey 2005 | 2005 - 2005 | Microdata | FPG ≥ 7 mmol/L or on treatment | Undiagnosed, Diagnosed & Untreated, Treated Envelope, Treated & Suboptimal glycaemic levels, Treated & Optimal glycaemic levels | Ministry of Health (Mozambique), World Health Organization (WHO). Mozambique STEPS Noncommunicable Disease Risk Factors Survey 2005. |
| Myanmar | Myanmar STEPS Noncommunicable Disease Risk Factors Survey 2014 | 2014 - 2014 | Microdata | FPG ≥ 7 mmol/L or on treatment | Undiagnosed, Diagnosed & Untreated, Treated Envelope, Treated & Suboptimal glycaemic levels, Treated & Optimal glycaemic levels | Ministry of Health (Myanmar), Myanmar Medical Association, World Health Organization (WHO). Myanmar STEPS Noncommunicable Disease Risk Factors Survey 2014. Geneva, Switzerland: World Health Organization (WHO). |
| Namibia | Namibia Demographic and Health Survey 2013 | 2013 - 2013 | Scientific literature | FPG ≥ 7 mmol/L or on treatment | Treated & Optimal glycaemic levels | ICF International, Ministry of Health and Social Services (Namibia), Namibia Institute of Pathology, Namibia Statistics Agency. Namibia Demographic and Health Survey 2013. Fairfax, United States of America: ICF International. |
| Nauru | Nauru STEPS Noncommunicable Disease Risk Factors Survey 2004 | 2004 - 2004 | Microdata | FPG ≥ 7 mmol/L or on treatment | Undiagnosed, Diagnosed & Untreated, Treated Envelope, Treated & Suboptimal glycaemic levels, Treated & Optimal glycaemic levels | Centre for Physical Activity and Health, University of Sydney (Australia), Ministry of Health (Nauru), World Health Organization (WHO). Nauru STEPS Noncommunicable Disease Risk Factors Survey 2004. |
| Nauru | Nauru STEPS Noncommunicable Disease Risk Factors Survey 2015-2016 | 2015 - 2016 | Microdata | FPG ≥ 7 mmol/L or on treatment | Undiagnosed, Diagnosed & Untreated, Treated Envelope, Treated & Suboptimal glycaemic levels, Treated & Optimal glycaemic levels | Ministry of Health (Nauru), World Health Organization (WHO). Nauru STEPS Noncommunicable Disease Risk Factors Survey 2015-2016. Geneva, Switzerland: World Health Organization (WHO). |
| Nepal | Nepal STEPS Noncommunicable Disease Risk Factors Survey 2019 | 2019 - 2019 | Microdata | FPG ≥ 7 mmol/L or on treatment | Undiagnosed, Diagnosed & Untreated, Treated Envelope, Treated & Suboptimal glycaemic levels, Treated & Optimal glycaemic levels | Ministry of Health and Population (Nepal), Nepal Health Research Council (NHRC), World Health Organization (WHO). Nepal STEPS Noncommunicable Disease Risk Factors Survey 2019. Geneva, Switzerland: World Health Organization (WHO). |
| Niger | Niger STEPS Noncommunicable Disease Risk Factors Survey 2007 | 2007 - 2007 | Microdata | FPG ≥ 7 mmol/L or on treatment | Undiagnosed, Diagnosed & Untreated, Treated Envelope, Treated & Suboptimal glycaemic levels, Treated & Optimal glycaemic levels | Ministry of Health (Niger), World Health Organization (WHO). Niger STEPS Noncommunicable Disease Risk Factors Survey 2007. |
| Niue | Niue STEPS Noncommunicable Disease Risk Factors Survey 2011-2012 | 2011 - 2012 | Microdata | FPG ≥ 7 mmol/L or on treatment | Undiagnosed, Diagnosed & Untreated, Treated Envelope, Treated & Suboptimal glycaemic levels, Treated & Optimal glycaemic levels | World Health Organization (WHO). Niue STEPS Noncommunicable Disease Risk Factors Survey 2011-2012. |
| Norway | Diabetes prevalence among older people receiving care at home: associations with symptoms, health status and psychological well-being | 2014 - 2015 | Scientific literature | HbA1c ≥ 6.5% or on treatment | Undiagnosed, Diagnosed & Untreated, Treated Envelope | Davies TT, Graue M, Igland J, Tell GS, Birkeland KI, Peyrot M, Haltbakk J. Diabetes prevalence among older people receiving care at home: associations with symptoms, health status and psychological well-being. Diabet Med. 2019; 36(1): 96-104. |
| Pakistan | Prevalence and risk factors for diabetes mellitus in a selected urban population of a city in Punjab | 2008 - 2008 | Scientific literature | FPG ≥ 7 mmol/L or on treatment | Treated & Optimal glycaemic levels | Zafar J, Bhatti F, Akhtar N, Rasheed U, Bashir R, Humayun S, Waheed A, Younus F, Nazar M, Umaimato. Prevalence and risk factors for diabetes mellitus in a selected urban population of a city in Punjab. J Pak Med Assoc. 2011; 61(1): 40-7. |
| Palau | Palau STEPS Noncommunicable Disease Risk Factors Survey 2011-2013 | 2011 - 2011 | Microdata | FPG ≥ 7 mmol/L or on treatment | Undiagnosed, Diagnosed & Untreated, Treated Envelope, Treated & Suboptimal glycaemic levels, Treated & Optimal glycaemic levels | Ministry of Health (Palau), World Health Organization (WHO). Palau STEPS Noncommunicable Disease Risk Factors Survey 2011-2013. Geneva, Switzerland: World Health Organization (WHO). |
| Palau | Palau STEPS Noncommunicable Disease Risk Factors Survey 2016 | 2016 - 2016 | Microdata | FPG ≥ 7 mmol/L or on treatment | Undiagnosed, Diagnosed & Untreated, Treated Envelope, Treated & Suboptimal glycaemic levels, Treated & Optimal glycaemic levels | Ministry of Health (Palau), World Health Organization (WHO). Palau STEPS Noncommunicable Disease Risk Factors Survey 2016. WHO NCD Microdata Repository, 2020. |
| Palau | The Protection Gap - Diagnosis, Treatment Status, and Disease Control for People with Diabetes and Hypertension in the US-Affiliated Pacific Islands | 2017 - 2017 | Scientific literature | FPG ≥ 7 mmol/L or on treatment | Undiagnosed, Diagnosed & Untreated, Treated Envelope, Treated & Suboptimal glycaemic levels, Treated & Optimal glycaemic levels | Durand AM, Cash HL, Reichhardt MLE, Taulung L, Tolenoa NM. The Protection Gap - Diagnosis, Treatment Status, and Disease Control for People with Diabetes and Hypertension in the US-Affiliated Pacific Islands. Hawaii J Health Soc Welf. 2023; 82(3): 66-71. |
| Palestine | Palestine STEPS Noncommunicable Disease Risk Factors Survey 2010-2011 | 2010 - 2011 | Microdata | FPG ≥ 7 mmol/L or on treatment | Undiagnosed, Diagnosed & Untreated, Treated Envelope, Treated & Suboptimal glycaemic levels, Treated & Optimal glycaemic levels | Ministry of Health (Palestine), World Health Organization (WHO). Palestine STEPS Noncommunicable Disease Risk Factors Survey 2010-2011. Geneva, Switzerland: World Health Organization (WHO). |
| Peru | Peru National Survey on Nutritional, Biochemical, Socioeconomic Indicators Related to Chronic Degenerative Diseases 2004-2005 | 2004 - 2005 | Microdata | FPG ≥ 7 mmol/L or on treatment | Undiagnosed, Diagnosed & Untreated, Treated Envelope, Treated & Suboptimal glycaemic levels, Treated & Optimal glycaemic levels | Ministry of Health (Peru), National Center for Food and Nutrition, National Institute of Health (Peru), National Institute of Health (Peru). Peru National Survey on Nutritional, Biochemical, Socioeconomic Indicators Related to Chronic Degenerative Diseases 2004-2005. |
| Poland | Prevalence, awareness, treatment and control of hypertension, diabetes and hypercholesterolemia, and associated risk factors in the Czech Republic, Russia, Poland and Lithuania: a cross-sectional study | 2002 - 2005 | Scientific literature | FPG ≥ 7 mmol/L or on treatment | Undiagnosed, Diagnosed & Untreated, Treated Envelope, Treated & Suboptimal glycaemic levels, Treated & Optimal glycaemic levels | Lu W, Pikhart H, Tamosiunas A, Kubinova R, Capkova N, Malyutina S, PajÄ…k A, Bobak M. Prevalence, awareness, treatment and control of hypertension, diabetes and hypercholesterolemia, and associated risk factors in the Czech Republic, Russia, Poland and Lithuania: a cross-sectional study. BMC Public Health. 2022; 22(1): 883. |
| Poland | Poland LIPIDOGRAM 2015-2016 Study | 2015 - 2016 | Microdata | FPG ≥ 7 mmol/L or on treatment | Undiagnosed, Diagnosed & Untreated, Treated Envelope, Treated & Suboptimal glycaemic levels, Treated & Optimal glycaemic levels | College of Family Physicians in Poland (CFPiP), Częstochowa University of Technology, Polish Lipid Association, Silesian Analytical Laboratories. Poland LIPIDOGRAM 2015-2016 Study. |
| Portugal | Prevalence, management and control of diabetes mellitus and associated risk factors in primary health care in Portugal | 2006 - 2007 | Scientific literature | FPG ≥ 7 mmol/L or on treatment | Treated Envelope | Cortez-Dias N, Martins S, Belo A, Fiuza M. Prevalence, management and control of diabetes mellitus and associated risk factors in primary health care in Portugal. Rev Port Cardiol. 2010; 29(4): 509-37. |
| Qatar | Qatar STEPS Noncommunicable Disease Risk Factors Survey 2012 | 2012 - 2012 | Microdata | FPG ≥ 7 mmol/L or on treatment | Undiagnosed, Diagnosed & Untreated, Treated Envelope, Treated & Suboptimal glycaemic levels, Treated & Optimal glycaemic levels | Qatar Statistics Authority, Supreme Council of Health (Qatar), World Health Organization (WHO). Qatar STEPS Noncommunicable Disease Risk Factors Survey 2012. Geneva, Switzerland: World Health Organization (WHO). |
| Republic of Korea | South Korea National Health and Nutrition Examination Survey 2005 | 2005 - 2005 | Microdata | FPG ≥ 7 mmol/L or on treatment | Undiagnosed, Diagnosed & Untreated, Treated Envelope, Treated & Suboptimal glycaemic levels, Treated & Optimal glycaemic levels | Korea Centers for Disease Control and Prevention (KCDC). South Korea National Health and Nutrition Examination Survey 2005. Osong-eup, Republic of Korea: Korea Centers for Disease Control and Prevention (KCDC). |
| Republic of Korea | South Korea National Health and Nutrition Examination Survey 2007 | 2007 - 2007 | Microdata | FPG ≥ 7 mmol/L or on treatment | Undiagnosed, Diagnosed & Untreated, Treated Envelope, Treated & Suboptimal glycaemic levels, Treated & Optimal glycaemic levels | Korea Centers for Disease Control and Prevention (KCDC). South Korea National Health and Nutrition Examination Survey 2007. Osong-eup, Republic of Korea: Korea Centers for Disease Control and Prevention (KCDC). |
| Republic of Korea | South Korea National Health and Nutrition Examination Survey 2008 | 2008 - 2008 | Microdata | FPG ≥ 7 mmol/L or on treatment | Undiagnosed, Diagnosed & Untreated, Treated Envelope, Treated & Suboptimal glycaemic levels, Treated & Optimal glycaemic levels | Korea Centers for Disease Control and Prevention (KCDC). South Korea National Health and Nutrition Examination Survey 2008. Osong-eup, Republic of Korea: Korea Centers for Disease Control and Prevention (KCDC). |
| Republic of Korea | South Korea National Health and Nutrition Examination Survey 2009 | 2009 - 2009 | Microdata | FPG ≥ 7 mmol/L or on treatment | Undiagnosed, Diagnosed & Untreated, Treated Envelope, Treated & Suboptimal glycaemic levels, Treated & Optimal glycaemic levels | Korea Centers for Disease Control and Prevention (KCDC). South Korea National Health and Nutrition Examination Survey 2009. Osong-eup, Republic of Korea: Korea Centers for Disease Control and Prevention (KCDC). |
| Republic of Korea | South Korea National Health and Nutrition Examination Survey 2010 | 2010 - 2010 | Microdata | FPG ≥ 7 mmol/L or on treatment | Undiagnosed, Diagnosed & Untreated, Treated Envelope, Treated & Suboptimal glycaemic levels, Treated & Optimal glycaemic levels | Korea Centers for Disease Control and Prevention (KCDC). South Korea National Health and Nutrition Examination Survey 2010. Osong-eup, Republic of Korea: Korea Centers for Disease Control and Prevention (KCDC). |
| Republic of Korea | South Korea National Health and Nutrition Examination Survey 2011 | 2011 - 2011 | Microdata | FPG ≥ 7 mmol/L or on treatment | Undiagnosed, Diagnosed & Untreated, Treated Envelope, Treated & Suboptimal glycaemic levels, Treated & Optimal glycaemic levels | Korea Centers for Disease Control and Prevention (KCDC). South Korea National Health and Nutrition Examination Survey 2011. Osong-eup, Republic of Korea: Korea Centers for Disease Control and Prevention (KCDC). |
| Republic of Korea | South Korea National Health and Nutrition Examination Survey 2012 | 2012 - 2012 | Microdata | FPG ≥ 7 mmol/L or on treatment | Undiagnosed, Diagnosed & Untreated, Treated Envelope, Treated & Suboptimal glycaemic levels, Treated & Optimal glycaemic levels | Korea Centers for Disease Control and Prevention (KCDC). South Korea National Health and Nutrition Examination Survey 2012. Osong-eup, Republic of Korea: Korea Centers for Disease Control and Prevention (KCDC). |
| Republic of Korea | South Korea National Health and Nutrition Examination Survey 2013 | 2013 - 2013 | Microdata | FPG ≥ 7 mmol/L or on treatment | Undiagnosed, Diagnosed & Untreated, Treated Envelope, Treated & Suboptimal glycaemic levels, Treated & Optimal glycaemic levels | Korea Centers for Disease Control and Prevention (KCDC). South Korea National Health and Nutrition Examination Survey 2013. Osong-eup, Republic of Korea: Korea Centers for Disease Control and Prevention (KCDC). |
| Republic of Korea | South Korea National Health and Nutrition Examination Survey 2014 | 2014 - 2014 | Microdata | FPG ≥ 7 mmol/L or on treatment | Undiagnosed, Diagnosed & Untreated, Treated Envelope, Treated & Suboptimal glycaemic levels, Treated & Optimal glycaemic levels | Korea Centers for Disease Control and Prevention (KCDC). South Korea National Health and Nutrition Examination Survey 2014. Osong-eup, Republic of Korea: Korea Centers for Disease Control and Prevention (KCDC). |
| Republic of Korea | South Korea National Health and Nutrition Examination Survey 2015 | 2015 - 2015 | Microdata | FPG ≥ 7 mmol/L or on treatment | Undiagnosed, Diagnosed & Untreated, Treated Envelope, Treated & Suboptimal glycaemic levels, Treated & Optimal glycaemic levels | Korea Centers for Disease Control and Prevention (KCDC). South Korea National Health and Nutrition Examination Survey 2015. Osong-eup, Republic of Korea: Korea Centers for Disease Control and Prevention (KCDC). |
| Republic of Korea | South Korea National Health and Nutrition Examination Survey 2016 | 2016 - 2016 | Microdata | FPG ≥ 7 mmol/L or on treatment | Undiagnosed, Diagnosed & Untreated, Treated Envelope, Treated & Suboptimal glycaemic levels, Treated & Optimal glycaemic levels | Korea Centers for Disease Control and Prevention (KCDC). South Korea National Health and Nutrition Examination Survey 2016. Osong-eup, Republic of Korea: Korea Centers for Disease Control and Prevention (KCDC). |
| Republic of Korea | South Korea National Health and Nutrition Examination Survey 2017 | 2017 - 2017 | Microdata | FPG ≥ 7 mmol/L or on treatment | Undiagnosed, Diagnosed & Untreated, Treated Envelope, Treated & Suboptimal glycaemic levels, Treated & Optimal glycaemic levels | Korea Centers for Disease Control and Prevention (KCDC). South Korea National Health and Nutrition Examination Survey 2017. Osong-eup, Republic of Korea: Korea Centers for Disease Control and Prevention (KCDC). |
| Republic of Korea | South Korea National Health and Nutrition Examination Survey 2018 | 2018 - 2018 | Microdata | FPG ≥ 7 mmol/L or on treatment | Undiagnosed, Diagnosed & Untreated, Treated Envelope, Treated & Suboptimal glycaemic levels, Treated & Optimal glycaemic levels | Korea Centers for Disease Control and Prevention (KCDC). South Korea National Health and Nutrition Examination Survey 2018. Osong-eup, Republic of Korea: Korea Centers for Disease Control and Prevention (KCDC). |
| Republic of Korea | South Korea National Health and Nutrition Examination Survey 2019 | 2019 - 2019 | Microdata | FPG ≥ 7 mmol/L or on treatment | Undiagnosed, Diagnosed & Untreated, Treated Envelope, Treated & Suboptimal glycaemic levels, Treated & Optimal glycaemic levels | Korea Centers for Disease Control and Prevention (KCDC). South Korea National Health and Nutrition Examination Survey 2019. Osong-eup, Republic of Korea: Korea Centers for Disease Control and Prevention (KCDC). |
| Republic of Korea | South Korea National Health and Nutrition Examination Survey 2020 | 2020 - 2020 | Microdata | FPG ≥ 7 mmol/L or on treatment | Undiagnosed, Diagnosed & Untreated, Treated Envelope, Treated & Suboptimal glycaemic levels, Treated & Optimal glycaemic levels | Korea Centers for Disease Control and Prevention (KCDC). South Korea National Health and Nutrition Examination Survey 2020. Osong-eup, Republic of Korea: Korea Centers for Disease Control and Prevention (KCDC). |
| Republic of Korea | South Korea National Health and Nutrition Examination Survey 2021 | 2021 - 2021 | Microdata | FPG ≥ 7 mmol/L or on treatment | Undiagnosed, Diagnosed & Untreated, Treated Envelope, Treated & Suboptimal glycaemic levels, Treated & Optimal glycaemic levels | Korea Centers for Disease Control and Prevention (KCDC). South Korea National Health and Nutrition Examination Survey 2021. Osong-eup, Republic of Korea: Korea Centers for Disease Control and Prevention (KCDC), 2023. |
| Republic of Korea | South Korea National Health and Nutrition Examination Survey 2022 | 2022 - 2022 | Microdata | FPG ≥ 7 mmol/L or on treatment | Undiagnosed, Diagnosed & Untreated, Treated Envelope, Treated & Suboptimal glycaemic levels, Treated & Optimal glycaemic levels | Korea Centers for Disease Control and Prevention (KCDC). South Korea National Health and Nutrition Examination Survey 2022. Osong-eup, Republic of Korea: Korea Centers for Disease Control and Prevention (KCDC), 2023. |
| Republic of Moldova | Moldova STEPS Noncommunicable Disease Risk Factors Survey 2013 | 2013 - 2013 | Microdata | FPG ≥ 7 mmol/L or on treatment | Undiagnosed, Diagnosed & Untreated, Treated Envelope, Treated & Suboptimal glycaemic levels, Treated & Optimal glycaemic levels | Ministry of Health (Moldova), National Center of Public Health (Moldova), National Office of Statistics (Moldova), World Health Organization (WHO). Moldova STEPS Noncommunicable Disease Risk Factors Survey 2013. |
| Russian Federation | Prevalence, awareness, treatment and control of hypertension, diabetes and hypercholesterolemia, and associated risk factors in the Czech Republic, Russia, Poland and Lithuania: a cross-sectional study | 2002 - 2005 | Scientific literature | FPG ≥ 7 mmol/L or on treatment | Undiagnosed, Diagnosed & Untreated, Treated Envelope, Treated & Suboptimal glycaemic levels, Treated & Optimal glycaemic levels | Lu W, Pikhart H, Tamosiunas A, Kubinova R, Capkova N, Malyutina S, PajÄ…k A, Bobak M. Prevalence, awareness, treatment and control of hypertension, diabetes and hypercholesterolemia, and associated risk factors in the Czech Republic, Russia, Poland and Lithuania: a cross-sectional study. BMC Public Health. 2022; 22(1): 883. |
| Russian Federation | Russia - Ural Eye and Medical Study 2015-2017 | 2015 - 2017 | Microdata | FPG ≥ 7 mmol/L or on treatment | Undiagnosed, Diagnosed & Untreated, Treated Envelope, Treated & Suboptimal glycaemic levels, Treated & Optimal glycaemic levels | Ufa Eye Research Institute. Russia - Ural Eye and Medical Study 2015-2017. |
| Russian Federation | Awareness of Hypertension, Hypercholesterolemia, and Diabetes Mellitus and Associated Characteristics in Russian Adults | 2015 - 2018 | Scientific literature | HbA1c ≥ 6.5% or on treatment | Undiagnosed, Diagnosed & Untreated, Treated Envelope | Sahatqija F, Hunsberger M, Cook S, Kholmatova K, Shapkina M, Malyutina S, Kudryavtsev AV. Awareness of Hypertension, Hypercholesterolemia, and Diabetes Mellitus and Associated Characteristics in Russian Adults. Int J Hypertens. 2024; 2024: 8542671. |
| Russian Federation | Russia Ural Very Old Study 2017-2020 | 2017 - 2020 | Microdata | FPG ≥ 7 mmol/L or on treatment | Undiagnosed, Diagnosed & Untreated, Treated Envelope, Treated & Suboptimal glycaemic levels, Treated & Optimal glycaemic levels | Ufa Eye Research Institute. Russia Ural Very Old Study 2017-2020. |
| Rwanda | Rwanda STEPS Noncommunicable Disease Risk Factor Survey 2012-2013 | 2012 - 2013 | Microdata | FPG ≥ 7 mmol/L or on treatment | Undiagnosed, Diagnosed & Untreated, Treated Envelope, Treated & Suboptimal glycaemic levels, Treated & Optimal glycaemic levels | Centers for Disease Control and Prevention (CDC), Ministry of Health (Rwanda), National Institute of Statistics of Rwanda, Rwanda Biomedical Center (RBC), World Health Organization (WHO). Rwanda STEPS Noncommunicable Disease Risk Factor Survey 2012-2013. |
| Rwanda | Rwanda STEPS Noncommunicable Disease Risk Factor Survey 2022 | 2021 - 2021 | Scientific literature | FPG ≥ 7 mmol/L or on treatment | Treated Envelope | Centers for Disease Control and Prevention (CDC), Ministry of Health (Rwanda), Rwanda Biomedical Center (RBC), World Health Organization (WHO). Rwanda STEPS Noncommunicable Disease Risk Factor Survey 2022. Kigali, Rwanda: Ministry of Health (Rwanda). |
| Saint Lucia | Saint Lucia STEPS Noncommunicable Disease Risk Factors Survey 2019-2020 | 2019 - 2020 | Microdata | FPG ≥ 7 mmol/L or on treatment | Undiagnosed, Diagnosed & Untreated, Treated Envelope, Treated & Suboptimal glycaemic levels, Treated & Optimal glycaemic levels | Access Accelerated, Central Statistical Office of Saint Lucia, Ministry of Health (Saint Lucia), Pan American Health Organization (PAHO), World Bank, World Health Organization (WHO). Saint Lucia STEPS Noncommunicable Disease Risk Factors Survey 2019-2020. WHO NCD Microdata Repository, 2024. |
| Samoa | Samoa STEPS Noncommunicable Disease Risk Factors Survey 2002 | 2002 - 2002 | Microdata | FPG ≥ 7 mmol/L or on treatment | Undiagnosed, Diagnosed & Untreated, Treated Envelope, Treated & Suboptimal glycaemic levels, Treated & Optimal glycaemic levels | Ministry of Health (Samoa). Samoa STEPS Noncommunicable Disease Risk Factors Survey 2002. |
| Samoa | Samoa STEPS Noncommunicable Disease Risk Factors Survey 2013 | 2013 - 2013 | Microdata | FPG ≥ 7 mmol/L or on treatment | Undiagnosed, Diagnosed & Untreated, Treated Envelope, Treated & Suboptimal glycaemic levels, Treated & Optimal glycaemic levels | George Institute for Global Health (Australia), International Council for the Control of Iodine Deficiency Disorders (ICCIDD), Ministry of Health (Samoa), Samoa Bureau of Statistics, World Health Organization (WHO). Samoa STEPS Noncommunicable Disease Risk Factors Survey 2013. |
| Sao Tome and Principe | Sao Tome and Principe STEPS Noncommunicable Disease Risk Factors Survey 2008 | 2008 - 2008 | Microdata | FPG ≥ 7 mmol/L or on treatment | Undiagnosed, Diagnosed & Untreated, Treated Envelope, Treated & Suboptimal glycaemic levels, Treated & Optimal glycaemic levels | World Health Organization (WHO). Sao Tome and Principe STEPS Noncommunicable Disease Risk Factors Survey 2008. |
| Sao Tome and Principe | São Tomé and Príncipe STEPS Noncommunicable Disease Risk Factors Survey 2019 | 2019 - 2019 | Microdata | FPG ≥ 7 mmol/L or on treatment | Undiagnosed, Diagnosed & Untreated, Treated Envelope, Treated & Suboptimal glycaemic levels, Treated & Optimal glycaemic levels | Ministry of Health (Sao Tome and Principe), World Health Organization (WHO). São Tomé and Príncipe STEPS Noncommunicable Disease Risk Factors Survey 2019. WHO NCD Microdata Repository, 2022. |
| Saudi Arabia | Saudi Arabia STEPS Noncommunicable Disease Risk Factors Survey 2004-2005 | 2004 - 2005 | Microdata | FPG ≥ 7 mmol/L or on treatment | Treated Envelope, Treated & Suboptimal glycaemic levels, Treated & Optimal glycaemic levels | Ministry of Health (Saudi Arabia), World Health Organization (WHO). Saudi Arabia STEPS Noncommunicable Disease Risk Factors Survey 2004-2005. |
| Saudi Arabia | Status of the diabetes epidemic in the Kingdom of Saudi Arabia, 2013 | 2013 - 2013 | Scientific literature | HbA1c ≥ 6.5% or on treatment | Undiagnosed, Diagnosed & Untreated | El Bcheraoui C, Basulaiman M, Tuffaha M, Daoud F, Robinson M, Jaber S, Mikhitarian S, Memish ZA, Al Saeedi M, AlMazroa MA, Mokdad AH. Status of the diabetes epidemic in the Kingdom of Saudi Arabia, 2013. Int J Public Health. 2014; 59(6): 1011-21. |
| Seychelles | Seychelles STEPS Noncommunicable Disease Risk Factors Survey 2004 | 2004 - 2004 | Microdata | FPG ≥ 7 mmol/L or on treatment | Undiagnosed, Diagnosed & Untreated, Treated Envelope, Treated & Suboptimal glycaemic levels, Treated & Optimal glycaemic levels | World Health Organization (WHO), Ministry of Health (Seychelles), Institute of Social and Preventive Medicine, University of Lausanne (Switzerland), University Hospital Center (Switzerland). Seychelles STEPS Noncommunicable Disease Risk Factors Survey 2004. |
| Singapore | Singapore National Health Survey 1992 | 1992 - 1992 | Microdata | FPG ≥ 7 mmol/L or on treatment | Undiagnosed, Diagnosed & Untreated, Treated Envelope, Treated & Suboptimal glycaemic levels, Treated & Optimal glycaemic levels | Ministry of Health (Singapore). Singapore National Health Survey 1992. |
| Singapore | Singapore National Health Survey 1998 | 1998 - 1998 | Microdata | FPG ≥ 7 mmol/L or on treatment | Undiagnosed, Diagnosed & Untreated, Treated Envelope, Treated & Suboptimal glycaemic levels, Treated & Optimal glycaemic levels | Ministry of Health (Singapore). Singapore National Health Survey 1998. |
| Singapore | Singapore National Health Survey 2004 | 2004 - 2004 | Microdata | FPG ≥ 7 mmol/L or on treatment | Undiagnosed, Diagnosed & Untreated, Treated Envelope, Treated & Suboptimal glycaemic levels, Treated & Optimal glycaemic levels | Ministry of Health (Singapore). Singapore National Health Survey 2004. |
| Singapore | Singapore National Health Survey 2010 | 2010 - 2010 | Microdata | FPG ≥ 7 mmol/L or on treatment | Undiagnosed, Diagnosed & Untreated, Treated Envelope, Treated & Suboptimal glycaemic levels, Treated & Optimal glycaemic levels | Ministry of Health (Singapore). Singapore National Health Survey 2010. |
| Singapore | Singapore National Population Health Survey 2016-2017 | 2016 - 2017 | Microdata | HbA1c ≥ 6.5% or on treatment | Undiagnosed, Diagnosed & Untreated, Treated Envelope | Ministry of Health (Singapore). Singapore National Population Health Survey 2016-2017. Singapore, Singapore: Ministry of Health (Singapore). |
| Solomon Islands | Solomon Islands STEPS Noncommunicable Disease Risk Factors Survey 2005-2006 | 2005 - 2006 | Microdata | FPG ≥ 7 mmol/L or on treatment | Undiagnosed, Diagnosed & Untreated, Treated Envelope, Treated & Suboptimal glycaemic levels, Treated & Optimal glycaemic levels | Fiji School of Medicine, Ministry of Health and Medical Services (Solomon Islands), World Health Organization (WHO). Solomon Islands STEPS Noncommunicable Disease Risk Factors Survey 2005-2006. |
| Solomon Islands | Solomon Islands STEPS Noncommunicable Disease Risk Factors Survey 2015 | 2015 - 2015 | Microdata | FPG ≥ 7 mmol/L or on treatment | Undiagnosed, Diagnosed & Untreated, Treated Envelope, Treated & Suboptimal glycaemic levels, Treated & Optimal glycaemic levels | Ministry of Health (Solomon Islands), World Health Organization (WHO). Solomon Islands STEPS Noncommunicable Disease Risk Factors Survey 2015. WHO NCD Microdata Repository, 2020. |
| South Africa | Prevalence and unmet need for diabetes care across the care continuum in a national sample of South African adults: Evidence from the SANHANES-1, 2011-2012 | 2011 - 2012 | Scientific literature | HbA1c ≥ 6.5% or on treatment | Undiagnosed, Diagnosed & Untreated, Treated Envelope | Stokes A, Berry KM, Mchiza Z, Parker WA, Labadarios D, Chola L, Hongoro C, Zuma K, Brennan AT, Rockers PC, Rosen S. Prevalence and unmet need for diabetes care across the care continuum in a national sample of South African adults: Evidence from the SANHANES-1, 2011-2012. PLoS One. 2017; 12(10): e0184264. |
| South Africa | Underdiagnosis of hypertension and diabetes mellitus in South Africa | 2016 - 2016 | Scientific literature | HbA1c ≥ 6.5% or on treatment | Undiagnosed, Diagnosed & Untreated, Treated Envelope | Kamerman P. Underdiagnosis of hypertension and diabetes mellitus in South Africa. S Afr Med J. 2022; 112(1): 13519. |
| Spain | [Prevalence of diabetes in an adult population in the region of Madrid (Spain). The Madrid Cardiovascular Risk study] | 2006 - 2006 | Scientific literature | FPG ≥ 7 mmol/L or on treatment | Undiagnosed, Diagnosed & Untreated, Treated Envelope | Rosado Martín J, Martínez López MÁ, Mantilla Morató T, Dujovne Kohan I, Palau Cuevas FJ, Torres Jiménez R, García Puig J. [Prevalence of diabetes in an adult population in the region of Madrid (Spain). The Madrid Cardiovascular Risk study]. *Gac Sanit*. 2012; 26(3): 243-50. |
| Sri Lanka | Sri Lanka STEPS Noncommunicable Disease Risk Factors Survey 2014-2015 | 2014 - 2015 | Microdata | FPG ≥ 7 mmol/L or on treatment | Undiagnosed, Diagnosed & Untreated, Treated Envelope, Treated & Suboptimal glycaemic levels, Treated & Optimal glycaemic levels | Department of Census and Statistics (Sri Lanka), Ministry of Health (Sri Lanka), World Health Organization (WHO), World Health Organization Regional Office for South-East Asia (SEARO). Sri Lanka STEPS Noncommunicable Disease Risk Factors Survey 2014-2015. |
| Sri Lanka | Sri Lanka STEPS Noncommunicable Disease Risk Factors Survey 2021 | 2021 - 2021 | Microdata | FPG ≥ 7 mmol/L or on treatment | Undiagnosed, Diagnosed & Untreated, Treated Envelope, Treated & Suboptimal glycaemic levels, Treated & Optimal glycaemic levels | Department of Census and Statistics (Sri Lanka), Ministry of Health (Sri Lanka), World Health Organization (WHO), World Health Organization Regional Office for South-East Asia (SEARO). Sri Lanka STEPS Noncommunicable Disease Risk Factors Survey 2021. WHO NCD Microdata Repository, 2021. |
| Sudan | Sudan STEPS Noncommunicable Disease Risk Factors Survey 2016 | 2016 - 2016 | Microdata | FPG ≥ 7 mmol/L or on treatment | Undiagnosed, Diagnosed & Untreated, Treated Envelope, Treated & Suboptimal glycaemic levels, Treated & Optimal glycaemic levels | Federal Ministry of Health (Sudan), World Health Organization (WHO). Sudan STEPS Noncommunicable Disease Risk Factors Survey 2016. Geneva, Switzerland: World Health Organization (WHO), 2019. |
| Switzerland | Prevalence, awareness and treatment of type 2 diabetes mellitus in Switzerland: the CoLaus study | 2003 - 2003 | Scientific literature | FPG ≥ 7 mmol/L or on treatment | Undiagnosed, Diagnosed & Untreated, Treated Envelope, Treated & Suboptimal glycaemic levels, Treated & Optimal glycaemic levels | Kaiser A, Vollenweider P, Waeber G, Marques-Vidal P. Prevalence, awareness and treatment of type 2 diabetes mellitus in Switzerland: the CoLaus study. Diabet Med. 2012; 29(2): 190-7. |
| Türkiye | Turkey Chronic Diseases and Risk Factors Study 2011 | 2011 - 2011 | Microdata | FPG ≥ 7 mmol/L or on treatment | Undiagnosed, Diagnosed & Untreated, Treated Envelope, Treated & Suboptimal glycaemic levels, Treated & Optimal glycaemic levels | Ministry of Health (Turkey). Turkey Chronic Diseases and Risk Factors Study 2011. |
| Tajikistan | Tajikistan STEPS Noncommunicable Disease Risk Factors Survey 2016 | 2016 - 2016 | Microdata | FPG ≥ 7 mmol/L or on treatment | Undiagnosed, Diagnosed & Untreated, Treated Envelope, Treated & Suboptimal glycaemic levels, Treated & Optimal glycaemic levels | Ministry of Health (Tajikistan), World Health Organization (WHO). Tajikistan STEPS Noncommunicable Disease Risk Factors Survey 2016. |
| Thailand | Prevalence and management of diabetes and metabolic risk factors in Thai adults: the Thai National Health Examination Survey IV, 2009 | 2004 - 2004 | Scientific literature | FPG ≥ 7 mmol/L or on treatment | Undiagnosed, Diagnosed & Untreated, Treated Envelope, Treated & Suboptimal glycaemic levels, Treated & Optimal glycaemic levels | Aekplakorn W, Chariyalertsak S, Kessomboon P, Sangthong R, Inthawong R, Putwatana P, Taneepanichskul S. Prevalence and management of diabetes and metabolic risk factors in Thai adults: the Thai National Health Examination Survey IV, 2009. Diabetes Care. 2011; 34(9): 1980-5. |
| Thailand | Thailand National Health and Examination Survey 2008-2009 | 2008 - 2009 | Scientific literature | FPG ≥ 7 mmol/L or on treatment | Undiagnosed, Diagnosed & Untreated, Treated Envelope, Treated & Suboptimal glycaemic levels, Treated & Optimal glycaemic levels | National Health Examination Survey Office (Thailand). Thailand National Health and Examination Survey 2008-2009. |
| Thailand | Prevalence of Diabetes and Relationship with Socioeconomic Status in the Thai Population: National Health Examination Survey, 2004-2014 | 2014 - 2014 | Scientific literature | FPG ≥ 7 mmol/L or on treatment | Undiagnosed | Aekplakorn W, Chariyalertsak S, Kessomboon P, Assanangkornchai S, Taneepanichskul S, Putwatana P. Prevalence of Diabetes and Relationship with Socioeconomic Status in the Thai Population: National Health Examination Survey, 2004-2014. J Diabetes Res. 2018; 2018: 1654530. |
| Timor-Leste | Timor-Leste STEPS Noncommunicable Disease Risk Factors Survey 2014 | 2014 - 2014 | Microdata | FPG ≥ 7 mmol/L or on treatment | Undiagnosed, Diagnosed & Untreated, Treated Envelope, Treated & Suboptimal glycaemic levels, Treated & Optimal glycaemic levels | Ministry of Health (Timor-Leste), National University of East Timor, World Health Organization (WHO). Timor-Leste STEPS Noncommunicable Disease Risk Factors Survey 2014. |
| Tokelau | Tokelau STEPS Noncommunicable Disease Risk Factors Survey 2005 | 2005 - 2005 | Microdata | FPG ≥ 7 mmol/L or on treatment | Undiagnosed, Diagnosed & Untreated, Treated Envelope, Treated & Suboptimal glycaemic levels, Treated & Optimal glycaemic levels | Australian Agency for International Development (AusAID), Department of Health (Tokelau), Fiji School of Medicine, World Health Organization (WHO). Tokelau STEPS Noncommunicable Disease Risk Factors Survey 2005. |
| Tokelau | Tokelau STEPS Noncommunicable Disease Risk Factors Survey 2014 | 2014 - 2014 | Microdata | FPG ≥ 7 mmol/L or on treatment | Undiagnosed, Diagnosed & Untreated, Treated Envelope, Treated & Suboptimal glycaemic levels, Treated & Optimal glycaemic levels | World Health Organization (WHO). Tokelau STEPS Noncommunicable Disease Risk Factors Survey 2014. |
| Tonga | Tonga STEPS Noncommunicable Disease Risk Factors Survey 2011-2012 | 2011 - 2012 | Microdata | FPG ≥ 7 mmol/L or on treatment | Undiagnosed, Diagnosed & Untreated, Treated Envelope, Treated & Suboptimal glycaemic levels, Treated & Optimal glycaemic levels | Ministry of Health (Tonga), World Health Organization (WHO). Tonga STEPS Noncommunicable Disease Risk Factors Survey 2011-2012. 2014. |
| Turkmenistan | Turkmenistan STEPS Noncommunicable Disease Risk Factors Survey 2018 | 2018 - 2018 | Microdata | FPG ≥ 7 mmol/L or on treatment | Undiagnosed, Diagnosed & Untreated, Treated Envelope, Treated & Suboptimal glycaemic levels, Treated & Optimal glycaemic levels | Ministry of Health and Medical Industry (Turkmenistan), State Committee on Statistics of Turkmenistan, World Health Organization (WHO). Turkmenistan STEPS Noncommunicable Disease Risk Factors Survey 2018. WHO NCD Microdata Repository, 2021. |
| Tuvalu | Tuvalu STEPS Noncommunicable Disease Risk Factors Survey 2015 | 2015 - 2015 | Microdata | FPG ≥ 7 mmol/L or on treatment | Undiagnosed, Diagnosed & Untreated, Treated Envelope, Treated & Suboptimal glycaemic levels, Treated & Optimal glycaemic levels | Ministry of Health (Tuvalu), World Health Organization (WHO). Tuvalu STEPS Noncommunicable Disease Risk Factors Survey 2015. Geneva, Switzerland: World Health Organization (WHO), 2020. |
| Uganda | Diabetes and pre-diabetes among persons aged 35 to 60 years in eastern Uganda: prevalence and associated factors | 2012 - 2012 | Scientific literature | FPG ≥ 7 mmol/L or on treatment | Undiagnosed, Diagnosed & Untreated, Treated Envelope, Treated & Suboptimal glycaemic levels, Treated & Optimal glycaemic levels | Mayega RW, Guwatudde D, Makumbi F, Nakwagala FN, Peterson S, Tomson G, Ostenson C-G. Diabetes and pre-diabetes among persons aged 35 to 60 years in eastern Uganda: prevalence and associated factors. PLoS One. 2013; 8(8): e72554. |
| Uganda | Uganda STEPS Noncommunicable Disease Risk Factors Survey 2014 | 2014 - 2014 | Microdata | FPG ≥ 7 mmol/L or on treatment | Undiagnosed, Diagnosed & Untreated, Treated Envelope, Treated & Suboptimal glycaemic levels, Treated & Optimal glycaemic levels | Ministry of Health (Uganda), World Health Organization (WHO). Uganda STEPS Noncommunicable Disease Risk Factors Survey 2014. Geneva, Switzerland: World Health Organization (WHO). |
| Uganda | Uganda STEPS Noncommunicable Disease Risk Factors Survey 2023 | 2023 - 2023 | Scientific literature | FPG ≥ 7 mmol/L or on treatment | Undiagnosed, Diagnosed & Untreated, Treated Envelope | Ministry of Health (Uganda), World Health Organization (WHO). Uganda STEPS Noncommunicable Disease Risk Factors Survey 2023. |
| Ukraine | Ukraine STEPS Noncommunicable Disease Risk Factors Survey 2019 | 2019 - 2019 | Microdata | FPG ≥ 7 mmol/L or on treatment | Undiagnosed, Diagnosed & Untreated, Treated Envelope, Treated & Suboptimal glycaemic levels, Treated & Optimal glycaemic levels | Ministry of Health (Ukraine), Ukrainian Institute for Social Research after Oleksandr Yaremenko, World Bank, World Health Organization (WHO). Ukraine STEPS Noncommunicable Disease Risk Factors Survey 2019. |
| United Arab Emirates | United Arab Emirates World Health Survey 2017-2018 | 2017 - 2018 | Microdata | FPG ≥ 7 mmol/L or on treatment | Undiagnosed, Diagnosed & Untreated, Treated Envelope, Treated & Suboptimal glycaemic levels, Treated & Optimal glycaemic levels | Abu Dhabi Department of Health (United Arab Emirates), Dubai Health Authority, Dubai Statistics Center (United Arab Emirates), Federal Competitiveness and Statistics Authority (United Arab Emirates), IQVIA, Ministry of Health and Prevention (United Arab Emirates), Statistics Centre Abu Dhabi. United Arab Emirates World Health Survey 2017-2018. |
| United Kingdom | United Kingdom Health Survey for England 1993-1994 - UK Data Service | 1993 - 1994 | Microdata | HbA1c ≥ 6.5% or on treatment | Undiagnosed, Diagnosed & Untreated, Treated Envelope | Office of Population Censuses and Surveys. Social Survey Division, Health Survey for England, 1993 [Computer file]. Colchester, Essex: UK Data Archive [distributor], April 1995. SN: 3316, http://dx.doi.org/10.5255/UKDA-SN-3316-1 |
| United Kingdom | United Kingdom - Scottish Health Survey 2003-2004 - UK Data Service | 2003 - 2004 | Microdata | HbA1c ≥ 6.5% or on treatment | Undiagnosed, Diagnosed & Untreated, Treated Envelope | Joint Health Surveys Unit, University College London and Medical Research Council. Social and Public Health Sciences Unit, Scottish Health Survey, 2003 [computer file]. Colchester, Essex: UK Data Archive [distributor], February 2006. SN: 5318. |
| United Kingdom | United Kingdom Health Survey for England 2003-2004 - UK Data Service | 2003 - 2004 | Microdata | HbA1c ≥ 6.5% or on treatment | Undiagnosed, Diagnosed & Untreated, Treated Envelope | National Centre for Social Research and University College London. Department of Epidemiology and Public Health, Health Survey for England, 2003 [computer file]. Colchester, Essex: UK Data Archive [distributor], March 2005. SN: 5098. |
| United Kingdom | United Kingdom Health Survey for England 2005-2006 - UK Data Service | 2005 - 2006 | Microdata | HbA1c ≥ 6.5% or on treatment | Undiagnosed, Diagnosed & Untreated, Treated Envelope | National Centre for Social Research and University College London. Department of Epidemiology and Public Health, Health Survey for England, 2005 [computer file]. Colchester, Essex: UK Data Archive [distributor], July 2007. SN: 5675. |
| United Kingdom | United Kingdom Health Survey for England 2006-2007 - UK Data Service | 2006 - 2007 | Microdata | HbA1c ≥ 6.5% or on treatment | Undiagnosed, Diagnosed & Untreated, Treated Envelope | National Centre for Social Research and University College London. Department of Epidemiology and Public Health, Health Survey for England, 2006 [computer file]. 4th Edition. Colchester, Essex: UK Data Archive [distributor], July 2011. SN: 5809, http://dx.doi.org/10.5255/UKDA-SN-5809-1 |
| United Kingdom | United Kingdom - Scottish Health Survey 2008 - UK Data Service | 2008 - 2008 | Microdata | HbA1c ≥ 6.5% or on treatment | Undiagnosed, Diagnosed & Untreated, Treated Envelope | Scottish Centre for Social Research and University College London. Department of Epidemiology and Public Health, Scottish Health Survey, 2008 [computer file]. 2nd Edition. Colchester, Essex: UK Data Archive [distributor], April 2013. SN: 6383, http://dx.doi.org/10.5255/UKDA-SN-6383-2 |
| United Kingdom | United Kingdom - Scottish Health Survey 2009 - UK Data Service | 2009 - 2009 | Microdata | HbA1c ≥ 6.5% or on treatment | Undiagnosed, Diagnosed & Untreated, Treated Envelope | Scottish Centre for Social Research and University College London. Department of Epidemiology and Public Health, Scottish Health Survey, 2009 [computer file]. 4th Edition. Colchester, Essex: UK Data Archive [distributor], November 2011. SN: 6713, http://dx.doi.org/10.5255/UKDA-SN-6713-2 |
| United Kingdom | United Kingdom Health Survey for England 2009-2010 - UK Data Service | 2009 - 2010 | Microdata | HbA1c ≥ 6.5% or on treatment | Undiagnosed, Diagnosed & Untreated, Treated Envelope | National Centre for Social Research and University College London. Department of Epidemiology and Public Health, Health Survey for England, 2009 [computer file]. 2nd Edition. Colchester, Essex: UK Data Archive [distributor], July 2011. SN: 6732, http://dx.doi.org/10.5255/UKDA-SN-6732-1 |
| United Kingdom | United Kingdom - Scottish Health Survey 2010 - UK Data Service | 2010 - 2010 | Microdata | HbA1c ≥ 6.5% or on treatment | Undiagnosed, Diagnosed & Untreated, Treated Envelope | ScotCen Social Research and University College London. Department of Epidemiology and Public Health, Scottish Health Survey, 2010 [computer file]. Colchester, Essex: UK Data Archive [distributor], April 2012. SN: 6987, http://dx.doi.org/10.5255/UKDA-SN-6987-1 |
| United Kingdom | United Kingdom Health Survey for England 2010 - UK Data Service | 2010 - 2010 | Microdata | HbA1c ≥ 6.5% or on treatment | Undiagnosed, Diagnosed & Untreated, Treated Envelope | NatCen Social Research and Royal Free and University College Medical School. Department of Epidemiology and Public Health, Health Survey for England, 2010 [computer file]. 2nd Edition. Colchester, Essex: UK Data Archive [distributor], July 2012. SN: 6986, http://dx.doi.org/10.5255/UKDA-SN-6986-2 |
| United Kingdom | United Kingdom - Scottish Health Survey 2011 - UK Data Service | 2011 - 2011 | Microdata | HbA1c ≥ 6.5% or on treatment | Undiagnosed, Diagnosed & Untreated, Treated Envelope | ScotCen Social Research, University College London. Department of Epidemiology and Public Health and University of Glasgow. MRC/CSO Social and Public Health Sciences Unit, Scottish Health Survey, 2011 [computer file]. 2nd Edition. Colchester, Essex: UK Data Archive [distributor], August 2013. SN: 7247, http://dx.doi.org/10.5255/UKDA-SN-7247-2 |
| United Kingdom | United Kingdom Health Survey for England 2011-2012 - UK Data Service | 2011 - 2012 | Microdata | HbA1c ≥ 6.5% or on treatment | Undiagnosed, Diagnosed & Untreated, Treated Envelope | NatCen Social Research and University College London. Department of Epidemiology and Public Health, Health Survey for England, 2011 [computer file]. Colchester, Essex: UK Data Archive [distributor], April 2013. SN: 7260, http://dx.doi.org/10.5255/UKDA-SN-7260-1 |
| United Kingdom | United Kingdom Health Survey for England 2012-2013 - UK Data Service | 2012 - 2013 | Microdata | HbA1c ≥ 6.5% or on treatment | Undiagnosed, Diagnosed & Untreated, Treated Envelope | NatCen Social Research and University College London. Department of Epidemiology and Public Health, Health Survey for England, 2012 [computer file]. Colchester, Essex: UK Data Archive [distributor], April 2014. SN: 7480, http://dx.doi.org/10.5255/UKDA-SN-7480-1 |
| United Kingdom | United Kingdom Health Survey for England 2013-2014 - UK Data Service | 2013 - 2014 | Microdata | HbA1c ≥ 6.5% or on treatment | Undiagnosed, Diagnosed & Untreated, Treated Envelope | NatCen Social Research and University College London. Department of Epidemiology and Public Health, Health Survey for England, 2013 [computer file]. Colchester, Essex: UK Data Archive [distributor], January 2015. SN: 7649, http://dx.doi.org/10.5255/UKDA-SN-7649-1 |
| United Kingdom | United Kingdom Health Survey for England 2014-2015 - UK Data Service | 2014 - 2015 | Microdata | HbA1c ≥ 6.5% or on treatment | Undiagnosed, Diagnosed & Untreated, Treated Envelope | NatCen Social Research, University College London. Department of Epidemiology and Public Health. (2016). Health Survey for England, 2014. [data collection]. UK Data Service. SN: 7919, http://dx.doi.org/10.5255/UKDA-SN-7919-1 |
| United Kingdom | United Kingdom Health Survey for England 2015-2016 - UK Data Service | 2015 - 2016 | Microdata | HbA1c ≥ 6.5% or on treatment | Undiagnosed, Diagnosed & Untreated, Treated Envelope | NatCen Social Research, University College London. Department of Epidemiology and Public Health. (2017). Health Survey for England, 2015. [data collection]. UK Data Service. SN: 8280, http://doi.org/10.5255/UKDA-SN-8280-1 |
| United Kingdom | United Kingdom Health Survey for England 2016-2017 - UK Data Service | 2016 - 2017 | Microdata | HbA1c ≥ 6.5% or on treatment | Undiagnosed, Diagnosed & Untreated, Treated Envelope | NatCen Social Research, University College London. Department of Epidemiology and Public Health. (2018). Health Survey for England, 2016. [data collection]. UK Data Service. SN: 8334, http://doi.org/10.5255/UKDA-SN-8334-1 |
| United Republic of Tanzania | Tanzania - Zanzibar STEPS Noncommunicable Disease Risk Factors Survey 2011 | 2011 - 2011 | Microdata | FPG ≥ 7 mmol/L or on treatment | Undiagnosed, Diagnosed & Untreated, Treated Envelope, Treated & Suboptimal glycaemic levels, Treated & Optimal glycaemic levels | Ministry of Health (Zanzibar), World Health Organization (WHO). Tanzania - Zanzibar STEPS Noncommunicable Disease Risk Factors Survey 2011. |
| United Republic of Tanzania | Tanzania STEPS Noncommunicable Disease Risk Factors Survey 2012 | 2012 - 2012 | Microdata | FPG ≥ 7 mmol/L or on treatment | Undiagnosed, Diagnosed & Untreated, Treated Envelope, Treated & Suboptimal glycaemic levels, Treated & Optimal glycaemic levels | National Institute for Medical Research (Tanzania), World Health Organization (WHO). Tanzania STEPS Noncommunicable Disease Risk Factors Survey 2012. |
| United States of America | United States National Health and Nutrition Examination Survey 1988-1994 | 1988 - 1994 | Microdata | HbA1c ≥ 6.5% or on treatment | Undiagnosed, Diagnosed & Untreated, Treated Envelope | National Center for Health Statistics (NCHS), Centers for Disease Control and Prevention (CDC). United States National Health and Nutrition Examination Survey 1988-1994. Hyattsville, United States: National Center for Health Statistics (NCHS), Centers for Disease Control and Prevention (CDC). |
| United States of America | United States National Health and Nutrition Examination Survey 1999-2000 | 1999 - 2000 | Microdata | HbA1c ≥ 6.5% or on treatment | Undiagnosed, Diagnosed & Untreated, Treated Envelope | National Center for Health Statistics (NCHS), Centers for Disease Control and Prevention (CDC). United States National Health and Nutrition Examination Survey 1999-2000. Hyattsville, United States: National Center for Health Statistics (NCHS), Centers for Disease Control and Prevention (CDC). |
| United States of America | United States National Health and Nutrition Examination Survey 2001-2002 | 2001 - 2002 | Microdata | HbA1c ≥ 6.5% or on treatment | Undiagnosed, Diagnosed & Untreated, Treated Envelope | National Center for Health Statistics (NCHS), Centers for Disease Control and Prevention (CDC). United States National Health and Nutrition Examination Survey 2001-2002. Hyattsville, United States: National Center for Health Statistics (NCHS), Centers for Disease Control and Prevention (CDC). |
| United States of America | United States National Health and Nutrition Examination Survey 2003-2004 | 2003 - 2004 | Microdata | HbA1c ≥ 6.5% or on treatment | Undiagnosed, Diagnosed & Untreated, Treated Envelope | National Center for Health Statistics (NCHS), Centers for Disease Control and Prevention (CDC). United States National Health and Nutrition Examination Survey 2003-2004. Hyattsville, United States: National Center for Health Statistics (NCHS), Centers for Disease Control and Prevention (CDC). |
| United States of America | United States National Health and Nutrition Examination Survey 2005-2006 | 2005 - 2006 | Microdata | HbA1c ≥ 6.5% or on treatment | Undiagnosed, Diagnosed & Untreated, Treated Envelope | National Center for Health Statistics (NCHS), Centers for Disease Control and Prevention (CDC). United States National Health and Nutrition Examination Survey 2005-2006. Hyattsville, United States: National Center for Health Statistics (NCHS), Centers for Disease Control and Prevention (CDC), 2007. |
| United States of America | United States National Health and Nutrition Examination Survey 2007-2008 | 2007 - 2008 | Microdata | HbA1c ≥ 6.5% or on treatment | Undiagnosed, Diagnosed & Untreated, Treated Envelope | National Center for Health Statistics (NCHS), Centers for Disease Control and Prevention (CDC). United States National Health and Nutrition Examination Survey 2007-2008. Hyattsville, United States: National Center for Health Statistics (NCHS), Centers for Disease Control and Prevention (CDC), 2009. |
| United States of America | United States National Health and Nutrition Examination Survey 2009-2010 | 2009 - 2010 | Microdata | HbA1c ≥ 6.5% or on treatment | Undiagnosed, Diagnosed & Untreated, Treated Envelope | National Center for Health Statistics (NCHS), Centers for Disease Control and Prevention (CDC). United States National Health and Nutrition Examination Survey 2009-2010. Hyattsville, United States: National Center for Health Statistics (NCHS), Centers for Disease Control and Prevention (CDC), 2011. |
| United States of America | United States National Health and Nutrition Examination Survey 2011-2012 | 2011 - 2012 | Microdata | HbA1c ≥ 6.5% or on treatment | Undiagnosed, Diagnosed & Untreated, Treated Envelope | National Center for Health Statistics, Centers for Disease Control and Prevention. United States National Health and Nutrition Examination Survey 2011-2012. Hyattsville, United States of America: National Center for Health Statistics, Centers for Disease Control and Prevention, 2013. |
| United States of America | United States National Health and Nutrition Examination Survey 2013-2014 | 2013 - 2014 | Microdata | HbA1c ≥ 6.5% or on treatment | Undiagnosed, Diagnosed & Untreated, Treated Envelope | National Center for Health Statistics, Centers for Disease Control and Prevention. United States National Health and Nutrition Examination Survey 2013-2014. Hyattsville, United States of America: National Center for Health Statistics, Centers for Disease Control and Prevention. |
| United States of America | United States National Health and Nutrition Examination Survey 2015-2016 | 2015 - 2016 | Microdata | HbA1c ≥ 6.5% or on treatment | Undiagnosed, Diagnosed & Untreated, Treated Envelope | National Center for Health Statistics, Centers for Disease Control and Prevention. United States National Health and Nutrition Examination Survey 2015-2016. Hyattsville, United States of America: National Center for Health Statistics, Centers for Disease Control and Prevention, 2017. |
| United States of America | United States National Health and Nutrition Examination Survey Pre-Pandemic 2017-2020 | 2017 - 2020 | Microdata | HbA1c ≥ 6.5% or on treatment | Undiagnosed, Diagnosed & Untreated, Treated Envelope | National Center for Health Statistics, Centers for Disease Control and Prevention. United States National Health and Nutrition Examination Survey Pre-Pandemic 2017-2020. Hyattsville, United States of America: National Center for Health Statistics, Centers for Disease Control and Prevention, 2022. |
| Uruguay | Uruguay STEPS Noncommunicable Disease Risk Factors Survey 2006 | 2006 - 2006 | Microdata | FPG ≥ 7 mmol/L or on treatment | Undiagnosed, Diagnosed & Untreated, Treated Envelope, Treated & Suboptimal glycaemic levels, Treated & Optimal glycaemic levels | Ministry of Public Health (Uruguay), World Health Organization (WHO). Uruguay STEPS Noncommunicable Disease Risk Factors Survey 2006. |
| Uruguay | Uruguay STEPS Noncommunicable Disease Risk Factors Survey 2013-2014 | 2013 - 2014 | Microdata | FPG ≥ 7 mmol/L or on treatment | Undiagnosed, Diagnosed & Untreated, Treated Envelope, Treated & Suboptimal glycaemic levels, Treated & Optimal glycaemic levels | Government of Uruguay, Ministry of Public Health (Uruguay). Uruguay STEPS Noncommunicable Disease Risk Factors Survey 2013-2014. Geneva, Switzerland: World Health Organization (WHO). |
| Vanuatu | Vanuatu STEPS Noncommunicable Disease Risk Factors Survey 2011 | 2011 - 2011 | Microdata | FPG ≥ 7 mmol/L or on treatment | Undiagnosed, Diagnosed & Untreated, Treated Envelope, Treated & Suboptimal glycaemic levels, Treated & Optimal glycaemic levels | Ministry of Health (Vanuatu), World Health Organization (WHO). Vanuatu STEPS Noncommunicable Disease Risk Factors Survey 2011. |
| Venezuela (Bolivarian Republic of) | Prevalence of diabetes in three regions of Venezuela. The VEMSOLS study results | 2006 - 2010 | Scientific literature | FPG ≥ 7 mmol/L or on treatment | Undiagnosed | Nieto-Martínez R, Mechanick JI, Brajkovich I, Ugel E, Risques A, Florez H, González-Rivas ­JP. Prevalence of diabetes in three regions of Venezuela. The VEMSOLS study results. Prim Care Diabetes. 2018; 12(2): 126-132. |
| Viet Nam | Vietnam STEPS Noncommunicable Disease Risk Factors Survey 2009 | 2009 - 2009 | Microdata | FPG ≥ 7 mmol/L or on treatment | Undiagnosed, Diagnosed & Untreated, Treated Envelope, Treated & Suboptimal glycaemic levels, Treated & Optimal glycaemic levels | Menzies Research Institute Tasmania, Ministry of Health (Vietnam). Vietnam STEPS Noncommunicable Disease Risk Factors Survey 2009. |
| Viet Nam | Vietnam STEPS Noncommunicable Disease Risk Factors Survey 2015 | 2015 - 2015 | Microdata | FPG ≥ 7 mmol/L or on treatment | Undiagnosed, Diagnosed & Untreated, Treated Envelope, Treated & Suboptimal glycaemic levels, Treated & Optimal glycaemic levels | Ministry of Health (Vietnam). Vietnam STEPS Noncommunicable Disease Risk Factors Survey 2015. Geneva, Switzerland: World Health Organization (WHO), 2016. |
| Viet Nam | Viet Nam STEPS Noncommunicable Disease Risk Factors Survey 2021 | 2021 - 2021 | Microdata | FPG ≥ 7 mmol/L or on treatment | Undiagnosed, Diagnosed & Untreated, Treated Envelope, Treated & Suboptimal glycaemic levels, Treated & Optimal glycaemic levels | General Department of Preventive Medicine, Ministry of Health (Viet Nam), General Statistics Office (Vietnam), World Health Organization (WHO). Viet Nam STEPS Noncommunicable Disease Risk Factors Survey 2021. WHO NCD Microdata Repository, 2024. |
| Zambia | Zambia - Lusaka STEPS Noncommunicable Disease Risk Factors Survey 2008 | 2008 - 2008 | Microdata | FPG ≥ 7 mmol/L or on treatment | Undiagnosed, Diagnosed & Untreated, Treated Envelope, Treated & Suboptimal glycaemic levels, Treated & Optimal glycaemic levels | Ministry of Health (Zambia), World Health Organization (WHO). Zambia - Lusaka STEPS Noncommunicable Disease Risk Factors Survey 2008. |
| Zambia | Zambia STEPS Noncommunicable Disease Risk Factors Survey 2017 | 2017 - 2017 | Microdata | FPG ≥ 7 mmol/L or on treatment | Undiagnosed, Diagnosed & Untreated, Treated Envelope, Treated & Suboptimal glycaemic levels, Treated & Optimal glycaemic levels | World Health Organization (WHO). Zambia STEPS Noncommunicable Disease Risk Factors Survey 2017. Geneva, Switzerland: World Health Organization (WHO). |

## Table S20. GBD 2023 diabetes prevalence input data citations

Citation

Abbasi A, Juszczyk D, van Jaarsveld CHM, Gulliford MC. Body Mass Index and Incident Type 1 and Type 2 Diabetes in Children and Young Adults: A Retrospective Cohort Study. J Endocr Soc. 2017; 1(5): 524-537.

Abdella N, Al Arouj M, Al Nakhi A, Al Assoussi A, Moussa M. Non-insulin-dependent diabetes in Kuwait: prevalence rates and associated risk factors. Diabetes Res Clin Pract. 1998; 42(3): 187-96.

Abebe SM, Berhane Y, Worku A, Assefa A. Diabetes mellitus in North West Ethiopia: a community based study. BMC Public Health. 2014; 97.

Abu Dhabi Department of Health (United Arab Emirates), Dubai Health Authority, Dubai Statistics Center (United Arab Emirates), Federal Competitiveness and Statistics Authority (United Arab Emirates), IQVIA, Ministry of Health and Prevention (United Arab Emirates), Statistics Centre Abu Dhabi. United Arab Emirates World Health Survey 2017-2018.

Abu Sham’a R a. H, Darwazah AK, Kufri FH, Yassin IH, Torok NI. MetS and cardiovascular risk factors among Palestinians of East Jerusalem. East Mediterr Health J. 2009; 15(6): 1464–73.

Achutha Menon Center for Health Sciences Studies, Sree Chitra Tirunal Institute for Medical Sciences and Technology (India), Kerala Directorate of Health Services (India), World Health Organization (WHO). India - Kerala Noncommunicable Disease Risk Factors Survey 2016-2017.

Adegoke OA, Adedoyin RA, Balogun MO, Adebayo RA, Bisiriyu LA, Salawu AA. Prevalence of metabolic syndrome in a rural community in Nigeria. Metab Syndr Relat Disord. 2010; 8(1): 59–62.

Administrative Department of Science, Technology, and Innovation (Colombia), Center for Development Projects, Pontifical Xavierian University, Ministry of Social Protection (Colombia), Specialized Information Systems. Colombia National Health Survey 2007-2008.

Aga Khan University, All India Institute of Medical Sciences, New Delhi (AIIMS), Centre for Chronic Disease Control (India), Emory University (United States), Madras Diabetes Research Foundation, National Institute of Mental Health and Neurosciences (India), Public Health Foundation of India. Center for Cardio-Metabolic Risk Reduction in South Asia Surveillance Study 2010-2011.

Agency of Health Research and Development (Indonesia). Indonesia Basic Health Research 2007-2008.

Ajlouni K, Jaddou H, Batieha A. Diabetes and impaired glucose tolerance in Jordan: prevalence and associated risk factors. J Intern Med. 1998; 244(4): 317-23.

Ajlouni K, Qusous Y, Khawaldeh AK, Jaddou H, Batiehah A, Ammari F, Zaheri M, Mashal A. Incidence of insulin-dependent diabetes mellitus in Jordanian children aged 0-14 y during 1992-1996. Acta Paediatr Suppl. 1999; 88(427): 11-3.

Al Zenki S, Al Omirah H, Al Hooti S, Al Hamad N, Jackson RT, Rao A, Al Jahmah N, Al Obaid I, Al Ghanim J, Al Somaie M, Zaghloul S, Al Othman A. High prevalence of metabolic syndrome among Kuwaiti adults--a wake-up call for public health intervention. Int J Environ Res Public Health. 2012; 9(5): 1984-96.

Alarouj M, Bennakhi A, Alnesef Y, Sharifi M, Elkum N. Diabetes and associated cardiovascular risk factors in the State of Kuwait: the first national survey. Int J Clin Pract. 2013; 67(1): 89-96.

Alberts M, Urdal P, Steyn K, Stensvold I, Tverdal A, Nel JH, Steyn NP. Prevalence of cardiovascular diseases and associated risk factors in a rural black population of South Africa. Eur J Prev Cardiol. 2005; 12(4): 347-54.

Al-Daghri NM, Al-Attas OS, Alokail MS, Alkharfy KM, Yousef M, Sabico SL, Chrousos GP. Diabetes mellitus type 2 and other chronic non-communicable diseases in the central region, Saudi Arabia (Riyadh cohort 2): a decade of an epidemic. BMC Med. 2011; 76.

Aldossari KK, Shubair MM, Al-Zahrani J, Alduraywish AA, AlAhmary K, Bahkali S, Aloudah SM, Almustanyir S, Al-Rizqi L, El-Zahaby SA, Toivola P, El-Metwally A. Association between Chronic Pain and Diabetes/Prediabetes: A Population-Based Cross-Sectional Survey in Saudi Arabia. Pain Res Manag. 2020; 2020: 8239474.

Al-Habori M, Al-Mamari M, Al-Meeri A. Type II diabetes mellitus and impaired glucose tolerance in Yemen: prevalence, associated metabolic changes and risk factors. Diabetes Res Clin Pract. 2004; 65(3): 275-81.

Ali RAB, Hannoun Z, Harraqui K, Zeghari L, Aboussaleh Y, Mohamed S, Anssoufouddine M, Bour A. Profile of diabetes and cardiovascular risk factors in adults Anjouan Island (Comoros). Pan Afr Med J. 2019; 33: 140.

Alikor CA, Emem-Chioma PC. EPIDEMIOLOGY OF DIABETES AND IMPAIRED FASTING GLUCOSE IN A RURAL COMMUNITY OF NIGERIAN NIGER DELTA REGION. Niger J Med. 2015; 24(2): 114–24.

Alkandari A, Alarouj M, Elkum N, Sharma P, Devarajan S, Abu-Farha M, Al-Mulla F, Tuomilehto J, Bennakhi A. Adult Diabetes and Prediabetes Prevalence in Kuwait: Data from the Cross-Sectional Kuwait Diabetes Epidemiology Program. J Clin Med. 2020; 9(11).

Alkerwi A, Sauvageot N, Donneau A-F, Lair M-L, Couffignal S, Beissel J, Delagardelle C, Wagener Y, Albert A, Guillaume M. First nationwide survey on cardiovascular risk factors in Grand-Duchy of Luxembourg (ORISCAV-LUX). BMC Public Health. 2010; 10(1): 468.

Allal-Elasmi M, Feki M, Zayani Y, Hsairi M, Haj Taieb S, Jemaa R, Sanhaji H, Omar S, Mebazaa A, Kaabachi N. Prehypertension among adults in Great Tunis region (Tunisia): A population-based study. Pathol Biol. 2012; 60(3): 174–9.

Al-Lawati JA, Mohammed AJ, Al-Hinai HQ, Jousilahti P. Prevalence of the metabolic syndrome among Omani adults. Diabetes Care. 2003; 26(6): 1781-5.

Allemann S, Saner C, Zwahlen M, Christ ER, Diem P, Stettler C. Long-term cardiovascular and non-cardiovascular mortality in women and men with type 1 and type 2 diabetes mellitus: a 30-year follow-up in Switzerland. Swiss Med Wkly. 2009; 139(39-40): 576-83.

Al-Nozha MM, Al-Maatouq MA, Al-Mazrou YY, Al-Harthi SS, Arafah MR, Khalil MZ, Khan NB, Al-Khadra A, Al-Marzouki K, Nouh MS, Abdullah M, Attas O, Al-Shahid MS, Al-Mobeireek A. Diabetes mellitus in Saudi Arabia. Saudi Med J. 2004; 25(11): 1603-10.

Al-Rubeaan K, Youssef AM, Ibrahim HM, Al-Sharqawi AH, AlQumaidi H, AlNaqeb D, Aburisheh KH. All-cause mortality and its risk factors among type 1 and type 2 diabetes mellitus in a country facing diabetes epidemic. Diabetes Res Clin Pract. 2016; 118: 130-9.

Altobelli E, Chiarelli F, Valenti M, Verrotti A, Tumini S, Di Orio F. Incidence of insulin-dependent diabetes mellitus (0-14 years) in the Abruzzo Region, Italy, 1990-1995: results from a population-based register. J Pediatr Endocrinol Metab. 1998; 11(4): 555-62.

American University of Beirut, World Health Organization (WHO). Lebanon STEPS Noncommunicable Disease Risk Factors Survey 2008-2009.

Amini M, Afshin-Nia F, Bashardoost N, Aminorroaya A, Shahparian M, Kazemi M. Prevalence and risk factors of diabetes mellitus in the Isfahan city population (aged 40 or over) in 1993. Diabetes Res Clin Pract. 1997; 38(3): 185-90.

Aminzadeh M, Navidi N, Valavi E, Aletayeb SMH. Childhood onset type 1 diabetes at a tertiary hospital in south-western Iran during 2000-2015: Rapid increase in admissions and high prevalence of DKA at diagnosis. Prim Care Diabetes. 2019; 13(1): 43-48.

Amoah AGB, Owusu SK, Adjei S. Diabetes in Ghana: a community based prevalence study in Greater Accra. Diabetes Res Clin Pract. 2002; 56(3): 197-205.

Analytical and Information Center of the Ministry of Health of Uzbekistan, Macro International, Inc, Ministry of Macroeconomics and Statistics (Uzbekistan). Uzbekistan Special Demographic and Health Survey 2002. Fairfax, United States of America: ICF International.

Anderson P, Grills N, Singh R, Singh R, Evans RG, Sengupta P, Thrift AG. Prevalence of diabetes and pre-diabetes in rural Tehri Garhwal, India: influence of diagnostic method. BMC Public Health. 2019; 19(1): 817.

Anokute CC. Epidemiologic studies of diabetes mellitus in Saudi Arabia--Part I--Screening of 3158 males in King Saud University. J R Soc Health. 1990; 110(6): 201-3.

Aregbesola A, Voutilainen S, Virtanen JK, Mursu J, Tuomainen TP. Gender difference in type 2 diabetes and the role of body iron stores. Ann Clin Biochem. 2017; 54(1): 113-120.

Arpi ML, Fichera G, Mancuso M, Lucenti C, Italia S, Tomaselli L, Motta RM, Mazza A, Vigneri R, Purrello F, Squatrito S. A ten-year (1989-1998) perspective study of the incidence of Type 1 diabetes in the district of Catania (Sicily) in a 0-14 year age group. J Endocrinol Invest. 2002; 25(5): 414-9.

Arpi ML, Italia S, Motta RM, Raimondo M, Mancuso M, Tomaselli L, Squatrito S, Vigneri R, Purrello F. Incidence of type I diabetes in the district of Catania, Sicily. Acta Diabetol. 1994; 31(1): 37-9.

Aschner P, King H, Triana de Torrado M, Rodriguez BM. Glucose intolerance in Colombia. A population-based survey in an urban community. Diabetes Care. 1993; 16(1): 90-3.

Asfour MG, Lambourne A, Soliman A, Al-Behlani S, Al-Asfoor D, Bold A, Mahtab H, King H. High prevalence of diabetes mellitus and impaired glucose tolerance in the Sultanate of Oman: results of the 1991 national survey. Diabet Med. 1995; 12(12): 1122-5.

Asmar R, Vol S, Pannier B, Brisac AM, Tichet J, El Hasnaoui A. High blood pressure and associated cardiovascular risk factors in France. J Hypertens. 2001; 19(10): 1727-32.

Aspray TJ, Mugusi F, Rashid S, Whiting D, Edwards R, Alberti KG, Unwin NC, Essential Non-Communicable Disease Health Intervention Project. Rural and urban differences in diabetes prevalence in Tanzania: the role of obesity, physical inactivity and urban living. Trans R Soc Trop Med Hyg. 2000; 94(6): 637-44.

Associates for Community and Population Research (ACPR), International Centre for Diarrhoeal Disease Research, Bangladesh (ICDDR,B), MEASURE Evaluation Project, Carolina Population Center, University of North Carolina, National Institute of Population Research and Training (NIPORT). Bangladesh Urban Health Survey 2006.

Australian Agency for International Development (AusAID), Department of Health (Tokelau), Fiji School of Medicine, World Health Organization (WHO). Tokelau STEPS Noncommunicable Disease Risk Factors Survey 2005.

Australian Agency for International Development (AusAID), Fiji School of Medicine, Menzies Center for Population Health Research, University of Tasmania (Australia), Ministry of Health (Fiji), World Health Organization (WHO). Fiji STEPS Noncommunicable Disease Risk Factors Survey 2002.

Australian Institute of Health and Welfare, National Diabetes Register (NDR) (Australia). Incidence of insulin-treated diabetes in Australia, 2020. Canberra, Australia: Australian Institute of Health and Welfare, 2022.

Azerbaijan Ministry of Health, World Health Organization (WHO). Azerbaijan STEPS Noncommunicable Disease Risk Factors Survey 2017.

Azimi-Nezhad M, Ghayour-Mobarhan M, Parizadeh MR, Safarian M, Esmaeili H, Parizadeh SM, Khodaee G, Hosseini J, Abasalti Z, Hassankhani B, Ferns G. Prevalence of type 2 diabetes mellitus in Iran and its relationship with gender, urbanisation, education, marital status and occupation. Singapore Med J. 2008; 49(7): 571-6.

Azimi-Nezhad M, Herbeth B, Siest G, Dadé S, Ndiaye NC, Esmaily H, Hosseini SJ, Ghayour-Mobarhan M, Visvikis-Siest S. High prevalence of metabolic syndrome in Iran in comparison with France: what are the components that explain this?. Metab Syndr Relat Disord. 2012; 10(3): 181–8.

Azizi F, Rahmani M, Emami H, Mirmiran P, Hajipour R, Madjid M, Ghanbili J, Ghanbarian A, Mehrabi Y, Saadat N, Salehi P, Mortazavi N, Heydarian P, Sarbazi N, Allahverdian S, Saadati N, Ainy E, Moeini S. Cardiovascular risk factors in an Iranian urban population: Tehran lipid and glucose study (phase 1). Soz Praventivmed. 2002; 47(6): 408-26.

Bahíllo MP, Hermoso F, Ochoa C, García-Fernández JA, Rodrigo J, Marugán JM, de la Torre S, Manzano F, Lema T, García-Velázquez J, Castilla-León Childhood Type 1 Diabetes Epidemiology Study Group. Incidence and prevalence of type 1 diabetes in children aged Pediatr Diabetes. 2007; 8(6): 369-73.

Bai A, Tao J, Tao L, Liu J. Prevalence and risk factors of diabetes among adults aged 45years or older in China: A national cross-sectional study. Endocrinol Diabetes Metab. 2021; 4(3): e00265.

Baker IDI Heart and Diabetes Institute. Australia Diabetes, Obesity and Lifestyle Study 2011-2012, Follow-up 2. Melbourne, Australia: Baker IDI Heart and Diabetes Institute, 2024.

Bald‚ NM, Diallo I, Bald‚ MD, Barry IS, Kaba L, Diallo MM, Kak‚ A, Camara A, Bah D, Barry MM, Sangar‚-Bah M, Maugendre D. Diabetes and impaired fasting glucose in rural and urban populations in Futa Jallon (Guinea): prevalence and associated risk factors. Diabetes Metab. 2007; 33(2): 114-20.

Baldeón ME, Felix C, Fornasini M, Zertuche F, Largo C, Paucar MJ, Ponce L, Rangarajan S, Yusuf S, López-Jaramillo P. Prevalence of metabolic syndrome and diabetes mellitus type-2 and their association with intake of dairy and legume in Andean communities of Ecuador. PLoS One. 2021; 16(7): e0254812.

Balijepalli C, Losch C, Bramlage P, Erbel R, Humphries KH, Jockel K-H, Moebus S. Percentile distribution of blood pressure readings in 35683 men and women aged 18 to 99 years. J Hum Hypertens. 2014; 28(3): 193–200.

Baragou S, Djibril M, Atta B, Damorou F, Pio M, Balogou A. Prevalence of cardiovascular risk factors in an urban area of Togo: a WHO STEPS-wise approach in Lome, Togo. Cardiovasc J Afr. 2012; 23(6): 309-12.

Barat P, Valade A, Brosselin P, Alberti C, Maurice-Tison S, L‚vy-Marchal C. The growing incidence of type 1 diabetes in children: the 17-year French experience in Aquitaine. Diabetes Metab. 2008; 34(6 Pt 1): 601-5.

Barbagallo CM, Cavera G, Sapienza M, Noto D, Cefalù AB, Pagano M, Montalto G, Notarbartolo A, Averna MR. Prevalence of overweight and obesity in a rural southern Italy population and relationships with total and cardiovascular mortality: the Ventimiglia di Sicilia project. Int J Obes (Lond). 2001; 25(2): 185-90.

Barcelo A, Daroca MC, Ribera R, Duarte E, Zapata A, Vohra M. Diabetes in Bolivia. Rev Panam Salud Publica. 2001; 10(5): 318-23.

Barnett KN, Ogston SA, McMurdo ME, Morris AD, Evans JM. A 12-year follow-up study of all-cause and cardiovascular mortality among 10,532 people newly diagnosed with Type 2 diabetes in Tayside, Scotland. Diabet Med. 2010; 27(10): 1124-9.

Barua S, Saikia N, Sk R. Spatial pattern and determinants of diagnosed diabetes in southern India: evidence from a 2012-13 population-based survey. J Biosoc Sci. 2021; 53(4): 623-638.

Basit A, Askari S, Zafar J, Riaz M, Fawwad A, NDSP Members. NDSP 06: Prevalence and risk factors for obesity in urban and rural areas of Pakistan: A study from second National Diabetes Survey of Pakistan (NDSP), 2016-2017. Obes Res Clin Pract. 2021; 15(1): 19-25.

Basit A, Hydrie MZ, Ahmed K, Hakeem R. Prevalence of diabetes, impaired fasting glucose and associated risk factors in a rural area of Baluchistan province according to new ADA criteria. J Pak Med Assoc. 2002; 52(8): 357-60.

Basit A, Mustafa N, Waris N, Askari S, Fawwad A, NDSP members. Predicting the risk of type 2 diabetes through anthropometric indices in Pakistani adults- A sub-analysis of second National diabetes survey of Pakistan 2016-2017 (NDSP-07). Diabetes Metab Syndr. 2021; 15(2): 543-547.

Battelino T, Krzisnik C. Incidence of type 1 diabetes mellitus in children in Slovenia during the years 1988-1995. Acta Diabetol. 1998; 35(2): 112-4.

Bautista LE, Oróstegui M, Vera LM, Prada GE, Orozco LC, Herrán OF. Prevalence and impact of cardiovascular risk factors in Bucaramanga, Colombia: results from the Countrywide Integrated Noncommunicable Disease Intervention Programme (CINDI/CARMEN) baseline survey. Eur J Cardiovasc Prev Rehabil. 2006; 13(5): 769-75.

Bayram F, Kocer D, Gundogan K, Kaya A, Demir O, Coskun R, Sabuncu T, Karaman A, Cesur M, Rizzo M, Toth PP, Gedik V. Prevalence of dyslipidemia and associated risk factors in Turkish adults. J Clin Lipidol. 2014; 8(2): 206–16.

Belfki H, Ben Ali S, Aounallah-Skhiri H, Traissac P, Bougatef S, Maire B, Delpeuch F, Achour N, Ben Romdhane H. Prevalence and determinants of the metabolic syndrome among Tunisian adults: results of the Transition and Health Impact in North Africa (TAHINA) project. Public Health Nutr. 2013; 16(4): 582–90.

Ben Khalifa F, Mekaouar A, Taktak S, Hamhoum M, Jebara H, Kodia A, Zouari B, Chakroun M. A five-year study of the incidence of insulin-dependent diabetes mellitus in young Tunisians (preliminary results). Diabetes Metab. 1997; 23(5): 395-401.

Bengtsson H, Bergqvist D, Ekberg O, Janzon L. A population based screening of abdominal aortic aneurysms (AAA). Eur J Vasc Surg. 1991; 5(1): 53-7.

Bentil HJ, Abreu AM, Adu-Afarwuah S, Rossi JS, Tovar A, Oaks BM. Association between Dietary Magnesium Intake and Glycemic Markers in Ghanaian Women of Reproductive Age: A Pilot Cross-Sectional Study. Nutrients. 2021; 13(11).

Berger B, Stenström G, Sundkvist G. Incidence, prevalence, and mortality of diabetes in a large population. A report from the Skaraborg Diabetes Registry. Diabetes Care. 1999; 22(5): 773-8.

Bermuda Diabetes Association, Bermuda Hospitals Board (BHB), Caribbean Public Health Agency (CARPHA), Ministry of Health, Seniors and Environment (Bermuda), World Health Organization (WHO). Bermuda STEPS Noncommunicable Disease Risk Factors Survey 2013-2014.

Bernabe-Ortiz A, Carrillo-Larco RM, Gilman RH, Checkley W, Smeeth L, Miranda JJ. Contribution of modifiable risk factors for hypertension and type-2 diabetes in Peruvian resource-limited settings. J Epidemiol Community Health. 2016; 70(1): 49-55.

Bidulescu A, Ferguson TS, Hambleton I, Younger-Coleman N, Francis D, Bennett N, Griswold M, Fox E, MacLeish M, Wilks R, Harris EN, Sullivan LW. Educational health disparities in hypertension and diabetes mellitus among African descent populations in the Caribbean and the USA: a comparative analysis from the Spanish town cohort (Jamaica) and the Jackson heart study (USA). Int J Equity Health. 2017; 16(1): 33.

Binh TQ, Phuong PT, Nhung BT, Tung DD. Metabolic syndrome among a middle-aged population in the Red River Delta region of Vietnam. BMC Endocr Disord. 2014; 14: 77.

Bizzarri C, Patera PI, Arnaldi C, Petrucci S, Bitti MLM, Scrocca R, Manfrini S, Portuesi R, Buzzetti R, Cappa M, Pozzilli P, Immunotherapy Diabetes (IMDIAB) Group. Incidence of type 1 diabetes has doubled in Rome and the Lazio region in the 0- to 14-year age-group: a 6-year prospective study (2004-2009). Diabetes Care. 2010; 33(11): e140.

Blanchard JF, Dean H, Anderson K, Wajda A, Ludwig S, Depew N. Incidence and prevalence of diabetes in children aged 0-14 years in Manitoba, Canada, 1985-1993. Diabetes Care. 1997; 20(4): 512-5.

Bo S, Ciccone G, Rosato R, Gancia R, Grassi G, Merletti F, Pagano GF. Renal damage in patients with Type 2 diabetes: a strong predictor of mortality. Diabet Med. 2005; 22(3): 258-65.

Bo S, Durazzo M, Guidi S, Carello M, Sacerdote C, Silli B, Rosato R, Cassader M, Gentile L, Pagano G. Dietary magnesium and fiber intakes and inflammatory and metabolic indicators in middle-aged subjects from a population-based cohort. Am J Clin Nutr. 2006; 84(5): 1062-9.

Bombelli M, Facchetti R, Fodri D, Brambilla G, Sega R, Grassi G, Mancia G. Impact of body mass index and waist circumference on the cardiovascular risk and all-cause death in a general population: data from the PAMELA study. Nutr Metab Cardiovasc Dis. 2013; 23(7): 650–6.

Borissova AM, Shinkov A, Kovatcheva R, Vlahov J, Dakovska L, Todorov T. Changes in the prevalence of diabetes mellitus in bulgaria (2006-2012). Clin Med Insights Endocrinol Diabetes. 2015; 8: 41-5.

Boronat M, Saavedra P, Perez-Martin N, Lopez-Madrazo MJ, Rodriguez-Perez C, Novoa FJ. High levels of lipoprotein(a) are associated with a lower prevalence of diabetes with advancing age: results of a cross-sectional epidemiological survey in Gran Canaria, Spain. Cardiovasc Diabetol. 2012; 11: 81.

Bratina NU, Tahirovic H, Battelino T, Krzisnik C. Incidence of childhood-onset Type I diabetes in Slovenia and the Tuzia region (Bosnia and Herzegovina) in the period 1990-1998. Diabetologia. 2001; B27-31.

Bream KDW, Breyre A, Garcia K, Calgua E, Chuc JM, Taylor L. Diabetes prevalence in rural Indigenous Guatemala: A geographic-randomized cross-sectional analysis of risk. PLoS One. 2018; 13(8): e0200434.

Breyer M-K, Ofenheimer A, Altziebler J, Hartl S, Burghuber OC, Studnicka M, Purin D, Heinzle C, Drexel H, Franssen FME, Wouters EFM, Harreiter J, KautzkyWiller A, BreyerKohansal R. Marked differences in prediabetes- and diabetes-associated comorbidities between men and women-Epidemiological results from a general population-based cohort aged 6-80 years-The LEAD (Lung, hEart, sociAl, boDy) study. Eur J Clin Invest. 2020; 50(3): e13207.

Brigham and Women's Hospital (USA). Guatemala Building Capacity for Chronic Kidney Disease Survey 2020.

Bruno G, Maule M, Biggeri A, Ledda A, Mannu C, Merletti F, Songini M, Sardinian Group for Diabetes Epidemiology. More than 20 years of registration of type 1 diabetes in Sardinian children: temporal variations of incidence with age, period of diagnosis, and year of birth. Diabetes. 2013; 62(10): 3542-6.

Bruno G, Maule M, Merletti F, Novelli G, Falorni A, Iannilli A, Iughetti L, Altobelli E, d'Annunzio G, Piffer S, Pozzilli P, Iafusco D, Songini M, Roncarolo F, Toni S, Carle F, Cherubini V. Age-period-cohort analysis of 1990-2003 incidence time trends of childhood diabetes in Italy: the RIDI study. Diabetes. 2010; 59(9): 2281-7.

Bruno G, Merletti F, De Salvia A, Lezo A, Arcari R, Pagano G. Comparison of incidence of insulin-dependent diabetes mellitus in children and young adults in the Province of Turin, Italy, 1984-91. Piedmont Study Group for Diabetes Epidemiology. Diabet Med. 1997; 14(11): 964-9.

Bruno G, Novelli G, Panero F, Perotto M, Monasterolo F, Bona G, Perino A, Rabbone I, Cavallo-Perin P, Cerutti F, Piedmont Study Group for Diabetes Epidemiology. The incidence of type 1 diabetes is increasing in both children and young adults in Northern Italy: 1984-2004 temporal trends. Diabetologia. 2009; 52(12): 2531-5.

Bustos P, da Silva AAM, Amigo H, Bettiol H, Barbieri MA. Metabolic syndrome in young adults from two socioeconomic Latin American settings. Nutr Metab Cardiovasc Dis. 2007; 17(8): 581-9.

Cabrera de León A, Rodríguez Pérez Mdel C, Almeida González D, Domínguez Coello S, Aguirre Jaime A, Brito Díaz B, González Hernández A, Pérez Méndez LI, grupo CDC. Presentación de la cohorte "CDC de Canarias": objetivos, diseño y resultados preliminares. Rev Esp Salud Publica. 2008; 82(5): 519-534.

Cai L, Liu A, Zhang L, Li S, Wang P. Prevalence, awareness, treatment, and control of hypertension among adults in Beijing, China. Clin Exp Hypertens. 2012; 34(1): 45–52.

Cai X, Xia L, Pan Y, He D, Zhu H, Wei T, He Y. Differential role of insulin resistance and -cell function in the development of prediabetes and diabetes in middle-aged and elderly Chinese population. Diabetol Metab Syndr. 2019; 11: 24.

Calori G, Gallus G, Garancini P, Repetto F, Micossi P. Identification of the cohort of type 1 diabetes presenting in Lombardy in 1983-84: a validated assessment. Diabet Med. 1990; 7(7): 595-9.

Campos H, Mata L, Siles X, Vives M, Ordovas JM, Schaefer EJ. Prevalence of cardiovascular risk factors in rural and urban Costa Rica. Circulation. 1992; 85(2): 648-58.

Cape Verde National Statistics Institute (INE), Ministry of Health (Cape Verde), World Health Organization (WHO). Cape Verde STEPS Noncommunicable Disease Risk Factors Survey 2020. WHO NCD Microdata Repository, 2023.

Cardwell CR, Carson DJ, Patterson CC. Secular trends, disease maps and ecological analyses of the incidence of childhood onset Type 1 diabetes in Northern Ireland, 1989-2003. Diabet Med. 2007; 24(3): 289-95.

Caribbean Epidemiology Centre (CAREC), Central Statistical Office (Trinidad and Tobago), Ministry of Health (Trinidad and Tobago), Pan American Health Organization (PAHO), University of the West Indies. Trinidad and Tobago STEPS Noncommunicable Disease Risk Factors Survey 2011.

Caribbean Epidemiology Centre (CAREC), Ministry of Health and Social Development (Virgin Islands, British), Pan American Health Organization (PAHO), World Health Organization (WHO). Virgin Islands, British STEPS Noncommunicable Disease Risk Factors Survey 2009.

Carolina Population Center, University of North Carolina at Chapel Hill, Chinese Center for Disease Control and Prevention (CCDC). China Health and Nutrition Survey. Chapel Hill, United States of America: Carolina Population Center, University of North Carolina at Chapel Hill.

Carrasco E, P‚rez-Bravo F, Dorman J, Mondrag¢n A, Santos JL. Increasing incidence of type 1 diabetes in population from Santiago of Chile: trends in a period of 18 years (1986-2003). Diabetes Metab Res Rev. 2006; 22(1): 34-7.

Casiglia E, Tikhonoff V, Mazza A, Piccoli A, Pessina AC. Pulse pressure and coronary mortality in elderly men and women from general population. J Hum Hypertens. 2002; 16(9): 611-20.

Castillo-Reinado K, Maier W, Holle R, Stahl-Pehe A, Baechle C, Kuss O, Hermann J, Holl RW, Rosenbauer J. Associations of area deprivation and urban/rural traits with the incidence of type 1 diabetes: analysis at the municipality level in North Rhine-Westphalia, Germany. Diabet Med. 2020; 37(12): 2089-2097.

Center for Health Development (Mongolia), EBM Research Institute Mongolia, Ministry of Health (Mongolia), National University of Medical Sciences (Mongolia). Mongolia H-Info Health System Data 2019.

Center for Research and Teaching in Economics (CIDE) (Mexico), Duke University, Ibero-American University, National Institute of Public Health (Mexico), University of California, Los Angeles (UCLA). Mexico Family Life Survey 2008-2013.

Center for Strategic Studies, University of Jordan, Ministry of Health (Jordan), World Health Organization (WHO). Jordan STEPS Noncommunicable Disease Risk Factors Survey 2019. WHO NCD Microdata Repository, 2021.

Centers for Disease Control and Prevention (CDC), Institute of Nutrition of Central America and Panama, Pan American Health Organization (PAHO). El Salvador - Santa Tecla Diabetes, Hypertension, and Chronic Disease Risk Factors Survey 2004.

Centers for Disease Control and Prevention (CDC), Institute of Nutrition of Central America and Panama, Pan American Health Organization (PAHO). Guatemala - Villa Nueva Diabetes, Hypertension, and Chronic Disease Risk Factors Survey 2002-2003.

Centers for Disease Control and Prevention (CDC), Ministry of Health (Jordan), World Health Organization (WHO). Jordan STEPS Noncommunicable Disease Risk Factors Survey 2007.

Centers for Disease Control and Prevention (CDC), Ministry of Health (Nicaragua), Pan American Health Organization (PAHO). Nicaragua - Managua Diabetes, Hypertension, and Non-Communicable Disease Risk Factors Survey 2003.

Centers for Disease Control and Prevention (CDC), Ministry of Health (Rwanda), National Institute of Statistics of Rwanda, Rwanda Biomedical Center (RBC), World Health Organization (WHO). Rwanda STEPS Noncommunicable Disease Risk Factor Survey 2012-2013.

Centers for Medicare & Medicaid Services (CMS) (United States). United States Medicare Claims 2000. Baltimore County, MD, United States of America: Centers for Medicare & Medicaid Services (CMS) (United States), 2018.

Centers for Medicare & Medicaid Services (CMS) (United States). United States Medicare Claims 2010. Baltimore County, MD, United States of America: Centers for Medicare & Medicaid Services (CMS) (United States), 2018.

Centers for Medicare & Medicaid Services (CMS) (United States). United States Medicare Claims 2014. Baltimore County, MD, United States of America: Centers for Medicare & Medicaid Services (CMS) (United States), 2018.

Centers for Medicare & Medicaid Services (CMS) (United States). United States Medicare Claims 2015. Baltimore County, MD, United States of America: Centers for Medicare & Medicaid Services (CMS) (United States), 2018.

Centers for Medicare & Medicaid Services (CMS) (United States). United States Medicare Claims 2016. Baltimore County, MD, United States of America: Centers for Medicare & Medicaid Services (CMS) (United States), 2018.

Central Agency for Public Mobilization and Statistics (CAPMAS) (Egypt), Ministry of Health and Population (Egypt), World Health Organization (WHO). Egypt STEPS Noncommunicable Disease Risk Factors Survey 2017.

Central Organization for Statistics and Information Technology (Iraq), Ministry of Health (Iraq), World Health Organization (WHO). Iraq STEPS Noncommunicable Disease Risk Factors Survey 2006.

Centre for Healthy Brain Ageing (CHeBA), University of New South Wales (UNSW), Gerontology Research Programme (GRP), National University Singapore, Department of Psychological Medicine. Singapore Longitudinal Ageing Study COSMIC Harmonized Dataset 2008-2013. Australia: Centre for Healthy Brain Ageing (CHeBA), University of New South Wales (UNSW), 2022.

Centre for Physical Activity and Health, University of Sydney (Australia), Department of Health and Social Affairs (Micronesia), Fiji School of Medicine, Micronesia Human Resources Development Center, Pohnpei State Department of Health Services, World Health Organization (WHO). Micronesia - Pohnpei STEPS Noncommunicable Disease Risk Factors Survey 2002.

Centre for Physical Activity and Health, University of Sydney (Australia), Ministry of Health (Nauru), World Health Organization (WHO). Nauru STEPS Noncommunicable Disease Risk Factors Survey 2004.

Chae HW, Seo GH, Song K, Choi HS, Suh J, Kwon A, Ha S, Kim HS. Incidence and Prevalence of Type 1 Diabetes Mellitus among Korean Children and Adolescents between 2007 and 2017: An Epidemiologic Study Based on a National Database. Diabetes Metab J. 2020; 44(6): 866-874.

Chalmers University of Technology (Sweden), Karolinska Institute (Sweden), Uppsala University, Örebro University (Sweden). Swedish Infrastructure for Medical Population-based Life-course and Environmental Research.

Chandra A, Lyngbakken MN, Eide IA, Røsjø H, Vigen T, Ihle-Hansen H, Orstad EB, Rønning OM, Berge T, Schmidt EB, Tveit A, Omland T, Svensson M. Plasma Trans Fatty Acid Levels, Cardiovascular Risk Factors and Lifestyle: Results from the Akershus Cardiac Examination 1950 Study. Nutrients. 2020; 12(5).

Chen D, Rivera-Andrade Á, González J, Burt D, Mendoza-Montano C, Patrie J, Luna M. Prevalence of risk factors for noncommunicable diseases in an indigenous community in Santiago Atitlán, Guatemala. Rev Panam Salud Publica. 2017; 41: e7.

Chen HD, Shaw CK, Tseng WP, Chen HI, Lee ML. Prevalence of diabetes mellitus and impaired glucose tolerance in Aborigines and Chinese in eastern Taiwan. Diabetes Res Clin Pract. 1997; 38(3): 199-205.

Chhetri JK, Zheng Z, Xu X, Ma C, Chan P. The prevalence and incidence of frailty in Pre-diabetic and diabetic community-dwelling older population: results from Beijing longitudinal study of aging II (BLSA-II). BMC Geriatr. 2017; 17(1): 47.

Chhetri MR, Chapman RS. Prevalence and determinants of diabetes among the elderly population in the Kathmandu Valley of Nepal. Nepal Med Coll J. 2009; 11(1): 34-8.

Chin CY, Pengal S. Cardiovascular disease risk in a semirural community in Malaysia. Asia Pac J Public Health. 2009; 21(4): 410-20.

Chinese Center for Disease Control and Prevention (CCDC). China Chronic Disease and Risk Factor Surveillance 2010.

Chinese Center for Disease Control and Prevention (CCDC). China Chronic Disease and Risk Factor Surveillance 2013.

Chou P, Liao MJ, Kuo HS, Hsiao KJ, Tsai ST. A population survey on the prevalence of diabetes in Kin-Hu, Kinmen. Diabetes Care. 1994; 17(9): 1055-8.

Chronic Disease Research Centre, University of the West Indies, Ministry of Health (Barbados). Barbados Health of the Nation Survey 2011.

Chueca M, Oyarzabal M, Reparaz F, Garagorri JM, Sola A. Incidence of type I diabetes mellitus in Navarre, Spain (1975-91). Acta Paediatr. 1997; 86(6): 632-7.

Chuuk Department of Health Services (Micronesia), Department of Health and Social Affairs (Micronesia), World Health Organization (WHO). Micronesia - Chuuk STEPS Noncommunicable Disease Risk Factors Survey 2006.

Chuuk Department of Health Services (Micronesia), Department of Health and Social Affairs (Micronesia), World Health Organization (WHO). Micronesia - Chuuk STEPS Noncommunicable Disease Risk Factors Survey 2016. WHO NCD Microdata Repository, 2019.

Cinek O, Sumnik Z, Vavrinec J. Continuing increase in incidence of childhood-onset type 1 diabetes in the Czech Republic 1990-2001. Eur J Pediatr. 2003; 162(6): 428-9.

Cockram CS, Woo J, Lau E, Chan JC, Chan AY, Lau J, Swaminathan R, Donnan SP. The prevalence of diabetes mellitus and impaired glucose tolerance among Hong Kong Chinese adults of working age. Diabetes Res Clin Pract. 1993; 21(1): 67-73.

College of Family Physicians in Poland (CFPiP), Czestochowa University of Technology, Polish Lipid Association, Silesian Analytical Laboratories. Poland LIPIDOGRAM 2015-2016 Study.

College of Medicine, Nursing and Health Sciences, Fiji National University, Ministry of Health (Cook Islands), World Health Organization (WHO). Cook Islands STEPS Noncommunicable Disease Risk Factors Survey 2003-2004.

Collier A, Meney C, Hair M, Cameron L, Boyle JG. Cancer has overtaken cardiovascular disease as the commonest cause of death in Scottish type2 diabetes patients: A population-based study (The Ayrshire Diabetes Follow-up Cohort study). J Diabetes Investig. 2020; 11(1): 55-61.

Collins VR, Dowse GK, Ram P, Cabealawa S, Zimmet PZ. Non-insulin-dependent diabetes and 11-year mortality in Asian Indian and Melanesian Fijians. Diabet Med. 1996; 13(2): 125-32.

Collins VR, Dowse GK, Toelupe PM, Imo TT, Aloaina FL, Spark RA, Zimmet PZ. Increasing prevalence of NIDDM in the Pacific island population of Western Samoa over a 13-year period. Diabetes Care. 1994; 17(4): 288-96.

Cook-Huynh M, Ansong D, Steckelberg RC, Boakye I, Seligman K, Appiah L, Kumar N, Amuasi JH. Prevalence of hypertension and diabetes mellitus in adults from a rural community in Ghana. Ethn Dis. 2012; 22(3): 347-52.

Correya TA, Ashraf AP, Griffin R, Aslibekyan S, Kim HD, Middleton S, McCormick K. Temporal trends in incidence of pediatric type 1 diabetes in Alabama: 2000-2017. Pediatr Diabetes. 2020; 21(1): 40-47.

Cortez-Dias N, Martins S, Belo A, Fiuza M. Prevalence, management and control of diabetes mellitus and associated risk factors in primary health care in Portugal. Rev Port Cardiol. 2010; 29(4): 509-37.

Cotellessa M, Barbieri P, Mazzella M, Bonassi S, Minicucci L, Lorini R. High incidence of childhood type 1 diabetes in Liguria, Italy, from 1989 to 1998. Diabetes Care. 2003; 26(6): 1786-9.

Craig ME, Howard NJ, Silink M, Chan A. The rising incidence of childhood type 1 diabetes in New South Wales, Australia. J Pediatr Endocrinol Metab. 2000; 13(4): 363-72.

Croxson SC, Burden AC, Bodington M, Botha JL. The prevalence of diabetes in elderly people. Diabet Med. 1991; 8(1): 28-31.

Cuong TQ, Dibley MJ, Bowe S, Hanh TT, Loan TT. Obesity in adults: an emerging problem in urban areas of Ho Chi Minh City, Vietnam. Eur J Clin Nutr. 2007; 61(5): 673-81.

CuoreData, Progetto Cuore, Italian National Institute of Health, http://www.cuore.iss.it/eng/survey/cuoredata, June 3, 2020.

Cuspidi C, Facchetti R, Dell'Oro R, Quarti-Trevano F, Tadic M, Mancia G, Grassi G. Office and Out-of-Office Blood Pressure Changes Over a Quarter of Century: Findings From the PAMELA Study. Hypertension. 2020; 76(3): 759-765.

Dahiru T, Jibo A, Hassan AA, Mande AT. Prevalence of diabetes in a semi-urban community in Northern Nigeria. Niger J Med. 2008; 17(4): 414-6.

Dahlquist GG, Nystrom L, Patterson CC. Incidence of type 1 diabetes in Sweden among individuals aged 0-34 years, 1983-2007: an analysis of time trends. Diabetes Care. 2011; 34(8): 1754-9.

Damsgaard EM, Frøland A, Mogensen CE. Over-mortality as related to age and gender in patients with established non-insulin-dependent diabetes mellitus. J Diabetes Complications. 1997; 11(2): 77-82.

Davies TT, Graue M, Igland J, Tell GS, Birkeland KI, Peyrot M, Haltbakk J. Diabetes prevalence among older people receiving care at home: associations with symptoms, health status and psychological well-being. Diabet Med. 2019; 36(1): 96-104.

de Marco R, Locatelli F, Zoppini G, Verlato G, Bonora E, Muggeo M. Cause-specific mortality in type 2 diabetes. The Verona Diabetes Study. Diabetes Care. 1999; 22(5): 756-61.

de Oliveira CM, Viater Tureck L, Alvares D, Liu C, Horimoto ARVR, Balcells M, de Oliveira Alvim R, Krieger JE, Pereira AC. Relationship between marital status and incidence of type 2 diabetes mellitus in a Brazilian rural population: The Baependi Heart Study. PLoS One. 2020; 15(8): e0236869.

De Pablos-Velasco PL, Martínez-Martín FJ, Rodríguez-Pérez F, Anía BJ, Losada A, Betancor P. Prevalence and determinants of diabetes mellitus and glucose intolerance in a Canarian Caucasian population - comparison of the 1997 ADA and the 1985 WHO criteria. The Guía Study. Diabet Med. 2001; 18(3): 235-41.

De Sereday MS, Gonzalez C, Giorgini D, De Loredo L, Braguinsky J, Cobeñas C, Libman C, Tesone C. Prevalence of diabetes, obesity, hypertension and hyperlipidemia in the central area of Argentina. Diabetes Metab. 2004; 30(4): 335-9.

de Souza LJ, Chalita FE, Reis AF, Teixeira CL, Gicovate Neto C, Bastos DA, De Souza TF, Côrtes VA, D. S. Filho JT. Prevalência de diabetes mellitus e fatores de risco em Campos dos Goytacazes, RJ. Arq Bras Endocrinol Metabol. 2003; 47(1): 69-74 .

Dedov II, Shestakova MV, Vikulova OK. Epidemiology of Diabetes Mellitus in Russian Federation: Clinical and Statistical Report According to the Federal Diabetes Registry. Diabetes Mellit. 2017; 20(1): 13–41.

Deepa M, Grace M, Binukumar B, Pradeepa R, Roopa S, Khan HM, Fatmi Z, Kadir MM, Naeem I, Ajay VS, Anjana RM, Ali MK, Prabhakaran D, Tandon N, Mohan V, Narayan KM; CARRS Surveillance Research Group. High burden of prediabetes and diabetes in three large cities in South Asia: The Center for Cardio-metabolic Risk Reduction in South Asia (CARRS) Study. Diabetes Res Clin Pract. 2015; 110(2): 172-82.

Defay R, Delcourt C, Ranvier M, Lacroux A, Papoz L. Relationships between physical activity, obesity and diabetes mellitus in a French elderly population: the POLA study. Pathologies Oculaires lieés á l' Age. Int J Obes Relat Metab Disord. 2001; 25(4): 512-8.

Department of Census and Statistics (Sri Lanka), Ministry of Health (Sri Lanka), World Health Organization (WHO), World Health Organization Regional Office for South-East Asia (SEARO). Sri Lanka STEPS Noncommunicable Disease Risk Factors Survey 2014-2015.

Department of Health (American Samoa), Monash University (Australia), World Health Organization (WHO). American Samoa STEPS Noncommunicable Disease Risk Factors Survey 2004.

Digestive Diseases Research Institute (DDRI) (Iran), Shiraz University of Medical Sciences. Iran - Pars Cohort Study.

Dionadji M, Boy B, Mouanodji M, Batakao G. Prevalence of diabetes mellitus in rural areas in Chad. Med Trop (Mars). 2010; 70(4): 414-5.

Directorate General of Health-Duhok (Iraq), Kurdistan Regional Government (Iraq), Ministry of Health (Iraq), World Health Organization (WHO). Iraq - Dahuk STEPS Noncommunicable Disease Risk Factors Survey 2003-2004.

Divison Garrote JA, Masso Orozco J, Carrion Valero L, Lopez Abril J, Carbayo Herencia JA, Artigao Rodenas LM, Gil Guillen V, Grupo de Enfermedades Vasculares de Albacete (GEVA). [Trends in prevalence of risk factors and global cardiovascular risk in general population of albacete, Spain (1992-94 a 2004-06)]. Rev Esp Salud Publica. 2011; 85(3): 275–84.

Dogan N, Toprak D, Demir S. Hypertension prevalence and risk factors among adult population in Afyonkarahisar region: a cross-sectional research. Anadolu Kardiyol Derg. 2012; 12(1): 47–52.

Dong Y, Gao W, Nan H, Yu H, Li F, Duan W, Wang Y, Sun B, Qian R, Tuomilehto J, Qiao Q. Prevalence of Type 2 diabetes in urban and rural Chinese populations in Qingdao, China. Diabet Med. 2005; 22(10): 1427-33.

Dorynska A, Polak M, Kozela M, Szafraniec K, Piotrowski W, Bielecki W, Drygas W, Kozakiewicz K, Piwonski J, Tykarski A, Zdrojewski T, Pajak A. Cardiovascular disease (CVD) risk factors in Krakow and in the whole Poland adult population. Results from the WOBASZ study and Polish arm of the HAPIEE project. Przegl Epidemiol. 2015; 69(1): 79–86.

Dowse GK, Gareeboo H, Zimmet PZ, Alberti KG, Tuomilehto J, Fareed D, Brissonnette LG, Finch CF. High prevalence of NIDDM and impaired glucose tolerance in Indian, Creole, and Chinese Mauritians. Mauritius Noncommunicable Disease Study Group. Diabetes. 1990; 39(3): 390-6.

Drivsholm T, Ibsen H, Schroll M, Davidsen M, Borch-Johnsen K. Increasing prevalence of diabetes mellitus and impaired glucose tolerance among 60-year-old Danes. Diabet Med. 2001; 18(2): 126-32.

Droumaguet C, Balkau B, Simon D, Caces E, Tichet J, Charles MA, Eschwege E, DESIR Study Group. Use of HbA1c in predicting progression to diabetes in French men and women: data from an Epidemiological Study on the Insulin Resistance Syndrome (DESIR). Diabetes Care. 2006; 29(7): 1619–25.

Duboz P, Chapuis-Lucciani N, Boëtsch G, Gueye L. Prevalence of diabetes and associated risk factors in a Senegalese urban (Dakar) population. Diabetes Metab. 2012; 38(4): 332-6.

Ebrahimi H, Emamian MH, Hashemi H, Fotouhi A. High Incidence of Diabetes Mellitus Among a Middle-Aged Population in Iran: A Longitudinal Study. Can J Diabetes. 2016; 40(6): 570–5.

Ebrahimpur M, Sharifi F, Nezhad FA, Bagherzadeh M, Ostovar A, Shafiee G, Heshmat R, Mehrdad N, Razi F, Khashayar P, Nabipour I, Larijani B. Effect of diabetes on BMD and TBS values as determinants of bone health in the elderly: Bushehr Elderly Health program. J Diabetes Metab Disord. 2019; 18(1): 99-106.

Echouffo-Tcheugui JB, Dzudie A, Epacka ME, Choukem SP, Doualla MS, Luma H, Kengne AP. Prevalence and determinants of undiagnosed diabetes in an urban sub-Saharan African population. Prim Care Diabetes. 2012; 6(3): 229–34.

Eggertsen R, Lapidus L, Lindstedt G, Nilsson T, Nyström E. [A study of 56-65 years old persons in Mölnlycke. No association between Helicobacter and heart disease or thyroid disorder]. Lakartidningen. 2002; 99(6): 508-9.

Eglit T, Rajasalu T, Lember M. Prevalence of diabetes and impaired glucose regulation in Estonia. Diabet Med. 2011; 28(4): 504-5.

Ehehalt S, Blumenstock G, Willasch AM, Hub R, Ranke MB, Neu A. Continuous rise in incidence of childhood Type 1 diabetes in Germany. Diabet Med. 2008; 25(6): 755-7.

Ejim EC, Onwubere BJ, Okafor CI, Ulasi II, Emehel A, Onyia U, Akabueze J, Mendis S. Cardiovascular risk factors in middle-aged and elderly residents in South-East Nigeria: the influence of urbanization. Niger J Med. 2013; 22(4): 286–91.

Elbagir MN, Eltom MA, Elmahadi EM, Kadam IM, Berne C. A high prevalence of diabetes mellitus and impaired glucose tolerance in the Danagla community in northern Sudan. Diabet Med. 1998; 15(2): 164-9.

Elbagir MN, Eltom MA, Elmahadi EM, Kadam IM, Berne C. A population-based study of the prevalence of diabetes and impaired glucose tolerance in adults in northern Sudan. Diabetes Care. 1996; 19(10): 1126-8.

El-Hazmi MA, Warsy AS, Al-Swailem AR, Al-Swailem AM, Sulaimani R, Al-Meshari AA. Diabetes mellitus and impaired glucose tolerance in Saudi Arabia. Ann Saudi Med. 1996; 16(4): 381-5.

Endris T, Worede A, Asmelash D. Prevalence of Diabetes Mellitus, Prediabetes and Its Associated Factors in Dessie Town, Northeast Ethiopia: A Community-Based Study. Diabetes Metab Syndr Obes. 2019; 12: 2799-2809.

Erem C, Yildiz R, Kavgaci H, Karahan C, Deger O, Can G, Telatar M. Prevalence of diabetes, obesity and hypertension in a Turkish population (Trabzon city). Diabetes Res Clin Pract. 2001; 54(3): 203-8.

Esen I, Okdemir D. Trend of type 1 diabetes incidence in children between 2009 and 2019 in Elazig, Turkey. Pediatr Diabetes. 2020; 21(3): 460-465.

Espelt A, Goday A, Franch J, Borrell C. Validity of self-reported diabetes in health interview surveys for measuring social inequalities in the prevalence of diabetes. J Epidemiol Community Health. 2012; 66(7): e15.

Ethiopian Public Health Institute (EPHI), World Health Organization (WHO). Ethiopia STEPS Noncommunicable Disease Risk Factors Survey 2015.

Evaristo-Neto AD, Foss-Freitas MC, Foss MC. Prevalence of diabetes mellitus and impaired glucose tolerance in a rural community of Angola. Diabetol Metab Syndr. 2010; 63.

Faculty of Public Health, Medical University of Varna (Bulgaria). Bulgaria - Dolni Chiflik Blood Pressure Study 2007.

Fallahzadeh H, Ostovarfar M, Lotfi MH. Population attributable risk of risk factors for type 2 diabetes; Bayesian methods. Diabetes Metab Syndr. 2019; 13(2): 1365-1368.

Fava C, Sjogren M, Montagnana M, Danese E, Almgren P, Engstrom G, Nilsson P, Hedblad B, Guidi GC, Minuz P, Melander O. Prediction of blood pressure changes over time and incidence of hypertension by a genetic risk score in Swedes. Hypertension. 2013; 61(2): 319–26.

Fawwad A, Alvi SFD, Basit A, Ahmed K, Ahmedani MY, Hakeem R. Changing pattern in the risk factors for diabetes in young adults from the rural area of Baluchistan. J Pak Med Assoc. 2013; 63(9): 1089–93.

Federal Ministry of Health (Sudan), World Health Organization (WHO). Sudan - Khartoum STEPS Noncommunicable Disease Risk Factors Survey 2005-2006.

Federal Ministry of Health (Sudan), World Health Organization (WHO). Sudan STEPS Noncommunicable Disease Risk Factors Survey 2016. Geneva, Switzerland: World Health Organization (WHO), 2019.

Federal University of Minas Gerais, State University of Montes Claros (Brazil). Brazil - Minas Gerais Noncommunicable Disease Risk Factors Survey 2012-2013.

Félix-Redondo FJ, Baena-Díez JM, Grau M, Tormo MÁ, Fernández-Bergés D. Prevalence of obesity and cardiovascular risk in the general population of a health area in Extremadura (Spain): the Hermex study. Endocrinol Nutr. 2012; 59(3): 160–8.

Feltbower RG, McKinney PA, Parslow RC, Stephenson CR, Bodansky HJ. Type 1 diabetes in Yorkshire, UK: time trends in 0-14 and 15-29-year-olds, age at onset and age-period-cohort modelling. Diabet Med. 2003; 20(6): 437-41.

Ferguson TS, Younger NOM, Tulloch-Reid MK, Wright MBL, Ward EM, Ashley DE, Wilks RJ. Prevalence of prehypertension and its relationship to risk factors for cardiovascular disease in Jamaica: analysis from a cross-sectional survey. BMC Cardiovasc Disord. 2008; 8: 20.

Fernandez-Ramos C, Arana-Arri E, Jimenez-Huertas P, Vela A, Rica I. Incidence of childhood-onset type 1 diabetes in Biscay, Spain, 1990-2013. Pediatr Diabetes. 2017; 18(1): 71–6.

Fernando DJ, Siribaddana S, de Silva D. Impaired glucose tolerance and diabetes mellitus in a suburban Sri Lankan community. Postgrad Med J. 1994; 70(823): 347-9.

Fiji School of Medicine, Menzies Center for Population Health Research, University of Tasmania (Australia), Ministry of Health (Marshall Islands), World Health Organization (WHO). Marshall Islands STEPS Noncommunicable Disease Risk Factors Survey 2002.

Fiji School of Medicine, Ministry of Health and Medical Services (Solomon Islands), World Health Organization (WHO). Solomon Islands STEPS Noncommunicable Disease Risk Factors Survey 2005-2006.

Flint SA, Gunn AJ, Hofman PL, Cutfield WS, Han DY, Mouat F, Willis J, Jefferies CA. Evidence of a plateau in the incidence of type 1 diabetes in children 0-4years of age from a regional pediatric diabetes center; Auckland, New Zealand: 1977-2019. Pediatr Diabetes. 2021; 22(6): 854-860.

Florez H, Silva E, Fernández V, Ryder E, Sulbarán T, Campos G, Calmón G, Clavel E, Castillo-Florez S, Goldberg R. Prevalence and risk factors associated with the metabolic syndrome and dyslipidemia in White, Black, Amerindian and Mixed Hispanics in Zulia State, Venezuela. Diabetes Res Clin Pract. 2005; 69(1): 63-77.

Florkowski CM, Scott RS, Coope PA, Moir CL. Predictors of mortality from type 2 diabetes mellitus in Canterbury, New Zealand; a ten-year cohort study. Diabetes Res Clin Pract. 2001; 53(2): 113-20.

Food and Nutrition Research Institute, Department of Science and Technology (Philippines). Philippines National Nutrition Survey 2013-2014.

Forga L, Chueca MJ, Tamayo I, Oyarzabal M, Toni M, Goñi MJ. Cyclical variation in the incidence of childhood-onset type 1 diabetes during 40 years in Navarra (Spain). Pediatr Diabetes. 2018; 19(8): 1416-1421.

Forga L, Tamayo I, Chueca M, Ibáñez B, Sainz de Los Terreros A, Goñi MJ, en representación del Grupo de Estudio de Diabetes tipo 1 de Navarra. Incidence of type 1 diabetes mellitus in Navarre stabilized in the last eight years. Endocrinol Diabetes Nutr (Engl Ed). 2018; 65(5): 274-279.

Formosa N, Calleja N, Torpiano J. Incidence and modes of presentation of childhood type 1 diabetes mellitus in Malta between 2006 and 2010. Pediatr Diabetes. 2012; 13(6): 484-8.

Fortunato F, Cappelli MG, Vece MM, Caputi G, Delvecchio M, Prato R, Martinelli D, Apulian Childhood-Onset Diabetes Registry Workgroup. Incidence of Type 1 Diabetes among Children and Adolescents in Italy between 2009 and 2013: The Role of a Regional Childhood Diabetes Registry. J Diabetes Res. 2016; 2016: 7239692.

Foster C, Rotimi C, Fraser H, Sundarum C, Liao Y, Gibson E, Holder Y, Hoyos M, Mellanson-King R. Hypertension, diabetes, and obesity in Barbados: findings from a recent population-based survey. Ethn Dis. 1993; 3(4): 404-12.

Freitas MPD, Loyola Filho AI de, Lima-Costa MF. Birth cohort differences in cardiovascular risk factors in a Brazilian population of older elderly: the Bambuí Cohort Study of Aging (1997 and 2008). Cad Saude Publica. 2011; S409-417.

French Institute for Public Health Surveillance (INVS), French National Health Insurance Fund for Salaried Workers (CNAMTS), National Conservatory of Arts and Trades (CNAM) (France), University of Paris 13. France National Nutrition and Health Survey 2006-2007 - Public Health France.

French Institute of Health and Medical Research (INSERM), University of Victor Segalen Bordeaux (France). France Three-City Cohort Study 1999-2001.

Gamlath L, Nandasena S, Hennadige Padmal de Silva S, Linhart C, Ngo A, Morrell S, Nathan S, Sharpe A, Taylor R. Differentials in Cardiovascular Risk Factors and Diabetes by Socioeconomic Status and Sex in Kalutara, Sri Lanka. Asia Pac J Public Health. 2017; 29(5): 401-410.

Gao WG, Dong YH, Pang ZC, Nan HR, Zhang L, Wang SJ, Ren J, Ning F, Qiao Q. Increasing trend in the prevalence of Type 2 diabetes and pre-diabetes in the Chinese rural and urban population in Qingdao, China. Diabet Med. 2009; 26(12): 1220-7.

Garancini MP, Calori G, Manara E, Izzo A, Ebbli E, Galli L, Boari L, Gallus G. An Italian population-based study of the prevalence of diabetes: some methodological aspects. Diabetes Metab. 1993; 19(1 Pt 2): 116-20.

Gardete-Correia L, Boavida JM, Raposo JF, Mesquita AC, Fona C, Carvalho R, Massano-Cardoso S. First diabetes prevalence study in Portugal: PREVADIAB study. Diabet Med. 2010; 27(8): 879-81.

Garfias CP, Borzutzky A, Ugarte MF, García HJ, Phingsthorn M, García HG. Mandatory notifications of type 1 diabetes incident cases in Chilean children, 2006 to 2014: A population-based study. Pediatr Diabetes. 2020; 21(1): 48-52.

Gatling W, Tufail S, Mullee MA, Westacott TA, Hill RD. Mortality rates in diabetic patients from a community-based population compared to local age/sex matched controls. Diabet Med. 1997; 14(4): 316-20.

Gause-Nilsson I, Gherman S, Kumar Dey D, Kennerfalk A, Steen B. Prevalence of metabolic syndrome in an elderly Swedish population. Acta Diabetol. 2006; 43(4): 120-6.

Gelaye B, Revilla L, Lopez T, Sanchez S, Williams MA. Prevalence of metabolic syndrome and its relationship with leisure time physical activity among Peruvian adults. Eur J Clin Invest. 2009; 39(10): 891–8.

George Institute for Global Health (Australia), International Council for the Control of Iodine Deficiency Disorders (ICCIDD), Ministry of Health (Samoa), Samoa Bureau of Statistics, World Health Organization (WHO). Samoa STEPS Noncommunicable Disease Risk Factors Survey 2013.

Ghafuri S, Ghaderi E, Fahami Y, Rajabnia M, Naleini SN. Epidemiologic study of type 2 diabetes mellitus and metabolic syndrome in rural population of kurdistan province, Iran, in 2011-2017. Diabetes Metab Syndr. 2019; 13(3): 1689-1697.

Ghana Health Service, World Health Organization (WHO). Ghana - Greater Accra Region STEPS Noncommunicable Disease Risk Factors Survey 2006. WHO NCD Microdata Repository, 2020.

Giri BR, Sharma KP, Chapagai RN, Palzom D. Diabetes and hypertension in urban bhutanese men and women. Indian J Community Med. 2013; 38(3): 138-43.

Glowinska-Olszewska B, Szablowski M, Panas P, Zola Dek K, Jamiolkowska-Sztabkowska M, Milewska AJ, Kadlubiska A, Polkowska A, Luczynski W, Bossowski A. Increasing Co-occurrence of Additional Autoimmune Disorders at Diabetes Type 1 Onset Among Children and Adolescents Diagnosed in Years 2010-2018-Single-Center Study. Front Endocrinol (Lausanne). 2020; 11: 476.

Gnavi R, Petrelli A, Demaria M, Spadea T, Carta Q, Costa G. Mortality and educational level among diabetic and non-diabetic population in the Turin Longitudinal Study: a 9-year follow-up. Int J Epidemiol. 2004; 33(4): 864-71.

Goday A, Castell C, Tresserras R, Canela J, Taberner JL, Lloveras G. Incidence of type 1 (insulin-dependent) diabetes mellitus in Catalonia, Spain. The Catalan Epidemiology Diabetes Study Group. Diabetologia. 1992; 35(3): 267-71.

Gokcel A, Ozsahin AK, Sezgin N, Karakose H, Ertorer ME, Akbaba M, Baklaci N, Sengul A, Guvener N. High prevalence of diabetes in Adana, a southern province of Turkey. Diabetes Care. 2003; 26(11): 3031-4.

Gomez-Huelgas R, Mancera-Romero J, Bernal-Lopez MR, Jansen-Chaparro S, Baca-Osorio AJ, Toledo E, Perez-Gonzalez R, Guijarro-Merino R, Tinahones FJ, Martinez-Gonzalez MA. Prevalence of cardiovascular risk factors in an urban adult population from southern Spain. IMAP Study. Int J Clin Pract. 2011; 65(1): 35–40.

Gong C, Meng X, Jiang Y, Wang X, Cui H, Chen X. Trends in childhood type 1 diabetes mellitus incidence in Beijing from 1995 to 2010: a retrospective multicenter study based on hospitalization data. Diabetes Technol Ther. 2015; 17(3): 159-65.

Goorakani Y, Sedigh Rahimabadi M, Dehghan A, Kazemi M, Chijan MR, Bijani M, Shahraki HR, Davoodi A, Farjam M, Homayounfar R. Correlation of resting heart rate with anthropometric factors and serum biomarkers in a population-based study: Fasa PERSIAN cohort study. BMC Cardiovasc Disord. 2020; 20(1): 319.

Gourdy P, Ruidavets JB, Ferrieres J, Ducimetiere P, Amouyel P, Arveiler D, Cottel D, Lamamy N, Bingham A, Hanaire-Broutin H; MONICA Study. Prevalence of type 2 diabetes and impaired fasting glucose in the middle-aged population of three French regions - the MONICA study 1995-97. Diabetes Metab. 2001; 27(3): 347-58.

Gouveri ET, Tzavara C, Drakopanagiotakis F, Tsaoussoglou M, Marakomichelakis GE, Tountas Y, Diamantopoulos EJ. Mediterranean diet and metabolic syndrome in an urban population: the Athens Study. Nutr Clin Pract. 2011; 26(5): 598–606.

Government of Canada, Public Health Agency of Canada. Canadian Chronic Disease Surveillance System Database 2018 and 2019: Aggregate Canadian Level Incidence and Prevalence Data by Sex and 5-year Age Groups 2000-2017. Ottawa, Canada: Canadian Public Health Association (CPHA), 2021.

Government of Uruguay, Ministry of Public Health (Uruguay). Uruguay STEPS Noncommunicable Disease Risk Factors Survey 2013-2014. Geneva, Switzerland: World Health Organization (WHO).

Graciani A, Leon-Munoz LM, Guallar-Castillon P, Rodriguez-Artalejo F, Banegas JR. Cardiovascular health in a southern Mediterranean European country: a nationwide population-based study. Circ Cardiovasc Qual Outcomes. 2013; 6(1): 90–8.

Grandinetti A, Kaholokula JK, Theriault AG, Mor JM, Chang HK, Waslien C. Prevalence of diabetes and glucose intolerance in an ethnically diverse rural community of Hawaii. Ethn Dis. 2007; 17(2): 250-5.

Gregory CO, Dai J, Ramirez-Zea M, Stein AD. Occupation is more important than rural or urban residence in explaining the prevalence of metabolic and cardiovascular disease risk in Guatemalan adults. J Nutr. 2007; 137(5): 1314-9.

Gronner MF, Bosi PL, Carvalho AM, Casale G, Contrera D, Pereira MA, Diogo TM, Torquato MTCG, Souza GMD, Oishi J, Leal AMO. Prevalence of metabolic syndrome and its association with educational inequalities among Brazilian adults: a population-based study. Braz J Med Biol Res. 2011; 44(7): 713–9.

Guerrero-Romero F, Violante R, Rodriguez-Moran M. Distribution of fasting plasma glucose and prevalence of impaired fasting glucose, impaired glucose tolerance and type 2 diabetes in the Mexican paediatric population. Paediatr Perinat Epidemiol. 2009; 23(4): 363-9.

Gumprecht J, Lip GYH, Sokal A, Sredniawa B, Mitrega K, Stokwiszewski J, Wierucki L, Rajca A, Rutkowski M, Zdrojewski T, Grodzicki T, Kazmierczak J, Opolski G, Kalarus Z. Relationship between diabetes mellitus and atrial fibrillation prevalence in the Polish population: a report from the Non-invasive Monitoring for Early Detection of Atrial Fibrillation (NOMED-AF) prospective cross-sectional observational study. Cardiovasc Diabetol. 2021; 20(1): 128.

Gunaid AA, Al-Radaei AN, LaPorte RE, Al-Qadasi FA, Ishak AA, Al-Serouri AA, AlKebsi TY, Bourji AA, Elshoubaki HR. Incidence of type 1 diabetes among children and adolescents during peace and war times in Yemen. Pediatr Diabetes. 2022; 23(3): 310-319.

Gunaid AA, Assabri AM. Prevalence of type 2 diabetes and other cardiovascular risk factors in a semirural area in Yemen. East Mediterr Health J. 2008; 14(1): 42-56.

Gundogan K, Bayram F, Capak M, Tanriverdi F, Karaman A, Ozturk A, Altunbas H, Gokce C, Kalkan A, Yazici C. Prevalence of metabolic syndrome in the Mediterranean region of Turkey: evaluation of hypertension, diabetes mellitus, obesity, and dyslipidemia. Metab Syndr Relat Disord. 2009; 7(5): 427–34.

Gupta S, Kumar R, Kalaivani M, Nongkynrih B, Kant S, Gupta SK. Prevalence, awareness, treatment, and control of diabetes and hypertension among elderly persons in a rural area of Ballabgarh, Haryana. J Fam Med Prim Care. 2020; 9(2): 777-782.

Guzder RN, Gatling W, Mullee MA, Byrne CD. Early mortality from the time of diagnosis of Type 2 diabetes: a 5-year prospective cohort study with a local age- and sex-matched comparison cohort. Diabet Med. 2007; 24(10): 1164-7.

Gyaneshwar R, Naidu S, Raban MZ, Naidu S, Linhart C, Morrell S, Tukana I, Taylor R. Absolute cardiovascular risk in a Fiji medical zone. BMC Public Health. 2016; 16: 128.

Gyurus EK, Patterson C, Soltesz G. Twenty-one years of prospective incidence of childhood type 1 diabetes in Hungary--the rising trend continues (or peaks and highlands?). Pediatr Diabetes. 2012; 13(1): 21-5.

Haba-Rubio J, Marques-Vidal P, Andries D, Tobback N, Preisig M, Vollenweider P, Waeber G, Luca G, Tafti M, Heinzer R. Objective sleep structure and cardiovascular risk factors in the general population: the HypnoLaus Study. Sleep. 2015; 38(3): 391–400.

Habeb AM, Al-Magamsi MS, Halabi S, Eid IM, Shalaby S, Bakoush O. High incidence of childhood type 1 diabetes in Al-Madinah, North West Saudi Arabia (2004-2009). Pediatr Diabetes. 2011; 12(8): 676-81.

Hamoudi R, Saheb Sharif-Askari N, Saheb Sharif-Askari F, Abusnana S, Aljaibeji H, Taneera J, Sulaiman N. Prediabetes and diabetes prevalence and risk factors comparison between ethnic groups in the United Arab Emirates. Sci Rep. 2019; 9(1): 17437.

Hanninen M-RA, Niiranen TJ, Puukka PJ, Johansson J, Jula AM. Prognostic significance of masked and white-coat hypertension in the general population: the Finn-Home Study. J Hypertens. 2012; 30(4): 705–12.

Hansen TW, Staessen JA, Torp-Pedersen C, Rasmussen S, Li Y, Dolan E, Thijs L, Wang J-G, O Brien E, Ibsen H. Ambulatory arterial stiffness index predicts stroke in a general population. J Hypertens. 2006; 24(11): 2247-54.

Harding JL, Shaw JE, Peeters A, Guiver T, Davidson S, Magliano DJ. Mortality trends among people with type 1 and type 2 diabetes in Australia: 1997-2010. Diabetes Care. 2014; 37(9): 2579-86.

Harjutsalo V, Sjberg L, Tuomilehto J. Time trends in the incidence of type 1 diabetes in Finnish children: a cohort study. Lancet. 2008; 371(9626): 1777-82.

Harjutsalo V, Sund R, Knip M, Groop PH. Incidence of type 1 diabetes in Finland. JAMA. 2013; 310(4): 427-8.

Haynes A, Bower C, Bulsara MK, Jones TW, Davis EA. Continued increase in the incidence of childhood Type 1 diabetes in a population-based Australian sample (1985-2002). Diabetologia. 2004; 47(5): 866-70.

He Y-H, Jiang G-X, Yang Y, Huang H-E, Li R, Li X-Y, Ning G, Cheng Q. Obesity and its associations with hypertension and type 2 diabetes among Chinese adults age 40 years and over. Nutrition. 2009; 25(11-12): 1143–9.

Health Canada, Public Health Agency of Canada, Statistics Canada (StatCan). Canada Health Measures Survey 2007-2009. Ottawa, Canada: Statistics Canada (StatCan).

Health Canada, Public Health Agency of Canada, Statistics Canada (StatCan). Canada Health Measures Survey 2009-2011. Ottawa, Canada: Statistics Canada (StatCan).

Health Insurance Review and Assessment Service (HIRA) (South Korea), Ministry of Health and Welfare (South Korea), National Health Insurance Service (South Korea). South Korea Medical Services Claims Data 2018.

Health Insurance Review and Assessment Service (HIRA) (South Korea), Ministry of Health and Welfare (South Korea), National Health Insurance Service (South Korea). South Korea Medical Services Claims Data 2019.

Health of Populations in Transition (HoPiT) Research Group (Cameroon), Ministry of Public Health (Cameroon), World Diabetes Foundation (WDF), World Health Organization (WHO). Cameroon STEPS Noncommunicable Disease Risk Factors Survey 2003.

Health Promotion Administration, Ministry of Health and Welfare (Taiwan). Taiwan National Nutrition and Health Survey 2013-2016.

Helgason T, Danielsen R, Thorsson AV. Incidence and prevalence of type 1 (insulin-dependent) diabetes mellitus in Icelandic children 1970-1989. Diabetologia. 1992; 35(9): 880-3.

Hightower JD, Hightower CM, Vázquez BYS, Intaglietta M. Incident prediabetes/diabetes and blood pressure in urban and rural communities in the Democratic Republic of Congo. Vasc Health Risk Manag. 2011; 7: 483–9.

Hiltunen L, Luukinen H, Koski K, Kivel„ SL. Prevalence of diabetes mellitus in an elderly Finnish population. Diabet Med. 1994; 11(3): 241-9.

Ho SC, Chen YM, Woo JL, Leung SS, Lam TH, Janus ED. Association between simple anthropometric indices and cardiovascular risk factors. Int J Obes Relat Metab Disord. 2001; 25(11): 1689-97.

Hodge AM, Dowse GK, Zimmet PZ, Gareeboo H, Westerman RA, Tuomilehto J, Alberti KG. Factors associated with impaired vibration perception in Mauritians with normal and abnormal glucose tolerance. Mauritius NCD Study Group. J Diabetes Complications. 1995; 9(3): 149-57.

Hollman G, Kristenson M. The prevalence of the metabolic syndrome and its risk factors in a middle-aged Swedish population--mainly a function of overweight?. Eur J Cardiovasc Nurs. 2008; 7(1): 21-6.

Ho-Pham LT, Nguyen UDT, Tran TX, Nguyen TV. Discordance in the diagnosis of diabetes: Comparison between HbA1c and fasting plasma glucose. PLoS One. 2017; 12(8): e0182192.

Hu G, Jousilahti P, Barengo NC, Qiao Q, Lakka TA, Tuomilehto J. Physical activity, cardiovascular risk factors, and mortality among Finnish adults with diabetes. Diabetes Care. 2005; 28(4): 799-805.

Hu Y, Teng W, Liu L, Chen K, Liu L, Hua R, Chen J, Zhou Y, Chen L. Prevalence and risk factors of diabetes and diabetic retinopathy in Liaoning province, China: a population-based cross-sectional study. PLoS One. 2015; 10(3): e0121477.

Huang G, Xu J, Zhang T, Cai L, Liu H, Yu X, Wu J. Hyperuricemia is associated with metabolic syndrome in the community very elderly in Chengdu. Sci Rep. 2020; 10(1): 8678.

Hussain A, Rahim MA, Azad Khan AK, Ali SM, Vaaler S. Type 2 diabetes in rural and urban population: diverse prevalence and associated risk factors in Bangladesh. Diabet Med. 2005; 22(7): 931-6.

Husseini A, Abdul-Rahim H, Awartani F, Jervell J, Bjertness E. Prevalence of diabetes mellitus and impaired glucose tolerance in a rural Palestinian population. East Mediterr Health J. 2000; 6(5-6): 1039-45.

ICF International, Ministry of Health and Social Services (Namibia), Namibia Institute of Pathology, Namibia Statistics Agency. Namibia Demographic and Health Survey 2013. Fairfax, United States of America: ICF International.

ICF International, Mitra and Associates, National Institute of Population Research and Training (NIPORT). Bangladesh Demographic and Health Survey 2017-2018. Fairfax, United States of America: ICF International, 2020.

ICF Macro, Mitra and Associates, National Institute of Population Research and Training (NIPORT). Bangladesh Demographic and Health Survey 2011-2012. Calverton, United States of America: ICF Macro.

Illangasekera U, Nugegoda DB, Perera LS. Prevalence of diabetes mellitus and impaired glucose tolerance in a rural Sri Lankan community. Ceylon Med J. 1993; 38(3): 123-6.

Ilow R, Regulska-Ilow B, Rozanska D, Kowalisko A, Biernat J. Prevalence of metabolic syndrome among 40- and 50-year-old inhabitants of Wroclaw, Poland. Ann Agric Environ Med. 2012; 19(3): 551–6.

Imamura Y, Uto H, Hiramine Y, Hosoyamada K, Ijuin S, Yoshifuku S, Miyahara H, Maenohara S, Oketani M, Ido A, Tsubouchi H. Increasing prevalence of diabetes mellitus in association with fatty liver in a Japanese population. J Gastroenterol. 2013.

Incidence and Mortality of Diabetes in Serbia 2021. Belgrade, Serbia [historical]: Institute of Public Health of Serbia.

Indian Council of Medical Research (ICMR), Madras Diabetes Research Foundation. Indian Council of Medical Research India Diabetes Study (ICMR-INDIAB) 2008-2010.

Indian Council of Medical Research (ICMR). Indian Council of Medical Research India Diabetes Study (ICMR-INDIAB) - North East 2011-2017.

Indian Council of Medical Research (ICMR). Indian Council of Medical Research India Diabetes Study (ICMR-INDIAB) 2012-2013.

Institute of Public Health (Republic of North Macedonia). North Macedonia Diabetes Mellitus Register 2020. 2021.

International Diabetes Institute (IDI). Australia Diabetes, Obesity and Lifestyle Study 1999-2000. Melbourne, Australia: International Diabetes Institute (IDI).

Ioacara S, Guja C, Ionescu-Tirgoviste C, Martin S, Tiu C, Fica S. Rates and Causes of Death among Adult Diabetes Patients in Romania. Endocr Res. 2019; 44(3): 81-86.

Ionescu-Tirgoviste C, Paterache E, Cheta D, Farcasiu E, Serafinceanu C, Mincu I. Epidemiology of diabetes in Bucharest. Diabet Med. 1994; 11(4): 413-7.

Isezuo SA, Sabir AA, Ohwovorilole AE, Fasanmade OA. Prevalence, associated factors and relationship between prehypertension and hypertension: a study of two ethnic African populations in Northern Nigeria. J Hum Hypertens. 2011; 25(4): 224–30.

Islam FM, Chakrabarti R, Islam MT, Wahab M, Lamoureux E, Finger RP, Shaw JE. Prediabetes, diagnosed and undiagnosed diabetes, their risk factors and association with knowledge of diabetes in rural Bangladesh: The Bangladesh Population-based Diabetes and Eye Study. J Diabetes. 2015; 8(2): nan.

Israel Center for Disease Control (ICDC). Israel Juvenile Diabetes Registry Report 2005-2007. Jerusalem, Israel: Ministry of Health (Israel), 2010.

Israel Center for Disease Control (ICDC). Israel Juvenile Diabetes Registry Report 2008-2009. Jerusalem, Israel: Ministry of Health (Israel), 2011.

Israel Center for Disease Control (ICDC). Israel Juvenile Diabetes Registry Report 2010. Jerusalem, Israel: Ministry of Health (Israel), 2012.

Israel Center for Disease Control (ICDC). Israel Juvenile Diabetes Registry Report 2011. Jerusalem, Israel: Ministry of Health (Israel), 2013.

Israel Center for Disease Control (ICDC). Israel Juvenile Diabetes Registry Report 2012. Jerusalem, Israel: Ministry of Health (Israel), 2014.

Israel Center for Disease Control (ICDC). Israel Juvenile Diabetes Registry Report 2013. Jerusalem, Israel: Ministry of Health (Israel), 2015.

Israel Center for Disease Control (ICDC). Israel Juvenile Diabetes Registry Report 2014. Jerusalem, Israel: Ministry of Health (Israel), 2016.

Israel Center for Disease Control (ICDC). Israel Juvenile Diabetes Registry Report 2015. Jerusalem, Israel: Ministry of Health (Israel), 2017.

Jacobs JM, Stessman J, Ein-Mor E, Bursztyn M. Hypertension and 5-year mortality among 85-year-olds: the Jerusalem Longitudinal Study. J Am Med Dir Assoc. 2012; 13(8): 759e1–6.

Janus ED, Laatikainen T, Dunbar JA, Kilkkinen A, Bunker SJ, Philpot B, Tideman PA, Tirimacco R, Heistaro S. Overweight, obesity and metabolic syndrome in rural southeastern Australia. Med J Aust. 2007; 187(3): 147-52.

Jarosz-Chobot P, Otto-Buczkowska E, Koehler B, Matlakiewicz E, Green A. Increased trend of type 1 diabetes mellitus in children's population (0-14 years) in Upper Silesia region (Poland). Med Sci Monit. 2000; 6(3): 573-80.

Jarosz-Chobot P, Polanska J, Szadkowska A, Kretowski A, Bandurska-Stankiewicz E, Ciechanowska M, Deja G, Mysliwiec M, Peczynska J, Rutkowska J, Sobel-Maruniak A, Fichna P, Chobot A, Rewers M. Rapid increase in the incidence of type 1 diabetes in Polish children from 1989 to 2004, and predictions for 2010 to 2025. Diabetologia. 2011; 54(3): 508-15.

Jasem D, Majaliwa ES, Ramaiya K, Najem S, Swai ABM, Ludvigsson J. Incidence, prevalence and clinical manifestations at onset of juvenile diabetes in Tanzania. Diabetes Res Clin Pract. 2019; 156: 107817.

Jeenduang N, Whanmasae S, Seepawin P, Kullabootr S. The prevalence of dyslipidemia among a rural Thai population in the Nakhon Si Thammarat province. J Med Assoc Thai. 2013; 96(8): 992–1000.

Jensen ET, Dabelea DA, Praveen PA, Amutha A, Hockett CW, Isom SP, Ong TC, Mohan V, D'Agostino R Jr, Kahn MG, Hamman RF, Wadwa P, Dolan L, Lawrence JM, Madhu SV, Chhokar R, Goel K, Tandon N, Mayer-Davis E. Comparison of the incidence of diabetes in United States and Indian youth: An international harmonization of youth diabetes registries. Pediatr Diabetes. 2021; 22(1): 8-14.

Jermendy G, Nadas J, Szigethy E, Szeles G, Nagy A, Hidvegi T, Paragh G, Adany R. Prevalence rate of diabetes mellitus and impaired fasting glycemia in Hungary: cross-sectional study on nationally representative sample of people aged 20-69 years. Croat Med J. 2010; 51(2): 151-6.

Joint Health Surveys Unit of Social and Community Planning Research and University College London, Health Survey for England, 1994 [computer file]. 4th ed. Colchester, Essex: UK Data Archive [distributor], 26 March 2001. SN: 3640.

Joint Health Surveys Unit, University College London and Medical Research Council. Social and Public Health Sciences Unit, Scottish Health Survey, 2003 [computer file]. Colchester, Essex: UK Data Archive [distributor], February 2006. SN: 5318.

Joner G, Stene LC, S?vik O. Nationwide, prospective registration of type 1 diabetes in children aged Diabetes Care. 2004; 27(7): 1618-22.

Jordan University of Science and Technology, National Center for Diabetes, Endocrinology, and Genetics (NCDEG) (Jordan). Jordan Cardiovascular Disease Risk Factor Survey 1994.

Jordan University of Science and Technology, National Center for Diabetes, Endocrinology, and Genetics (NCDEG) (Jordan). Jordan Cardiovascular Disease Risk Factor Survey 2009.

Jordan University of Science and Technology, National Center for Diabetes, Endocrinology, and Genetics (NCDEG) (Jordan). Jordan Cardiovascular Disease Risk Factor Survey 2017.

Jorgensen JMA, Hedt KH, Omar OM, Davies JI. Hypertension and diabetes in Zanzibar - prevalence and access to care. BMC Public Health. 2020; 20(1): 1352.

Jørgensen ME, Borch-Johnsen K, Witte DR, Bjerregaard P. Diabetes in Greenland and its relationship with urbanization. Diabet Med. 2012; 29(6): 755-60.

Jousilahti P, Vartiainen E, Tuomilehto J, Puska P. Sex, age, cardiovascular risk factors, and coronary heart disease: a prospective follow-up study of 14 786 middle-aged men and women in Finland. Circulation. 1999; 99(9): 1165-72.

Kadiki OA, Reddy MR, Marzouk AA. Incidence of insulin-dependent diabetes (IDDM) and non-insulin-dependent diabetes (NIDDM) (0-34 years at onset) in Benghazi, Libya. Diabetes Res Clin Pract. 1996; 32(3): 165-73.

Kadiki OA, Roaeid RB, Bhairi AM, Elamari IM. Incidence of insulin-dependent diabetes mellitus in Benghazi, Libya (1991-1995). Diabetes Metab. 1998; 24(5): 424-7.

Kadiki OA, Roaeid RB. Prevalence of diabetes mellitus and impaired glucose tolerance in Benghazi Libya. Diabetes Metab. 2001; 27(6): 647-54.

Kailuan General Hospital, Hebei United University. China - KaiLuan Study 2008-2009.

Kailuan General Hospital, Hebei United University. China - KaiLuan Study 2010-2011.

Kailuan General Hospital, Hebei United University. China - KaiLuan Study 2012-2014.

Kailuan General Hospital, Hebei United University. China - KaiLuan Study 2014-2016.

Kailuan General Hospital, Hebei United University. China - KaiLuan Study Baseline 2006-2007.

Kaiser A, Vollenweider P, Waeber G, Marques-Vidal P. Prevalence, awareness and treatment of type 2 diabetes mellitus in Switzerland: the CoLaus study. Diabet Med. 2012; 29(2): 190-7.

Kalits I, Podar T. Incidence and prevalence of type 1 (insulin-dependent) diabetes in Estonia in 1988. Diabetologia. 1990; 33(6): 346-9.

Kamdem F, Lemogoum D, Jingi AM, Guetchuin SC, Kenmegne C, Solange Doualla M, Luma H. Prevalence and determinants of abnormal glucose metabolism in urban and rural secondary schools in Cameroon: A cross-sectional study in a sub-Saharan Africa setting. Prim Care Diabetes. 2019; 13(4): 370-375.

Kamerman P. Underdiagnosis of hypertension and diabetes mellitus in South Africa. S Afr Med J. 2022; 112(1): 13519.

Kang S, Oh TJ, Cho BL, Park YS, Roh E, Kim HJ, Lee SG, Kim BJ, Kim M, Won CW, Jang HC. Sex differences in sarcopenia and frailty among community-dwelling Korean older adults with diabetes: The Korean Frailty and Aging Cohort Study. J Diabetes Investig. 2021; 12(2): 155-164.

Kang YM, Kim YJ, Park JY, Lee WJ, Jung CH. Mortality and causes of death in a national sample of type 2 diabetic patients in Korea from 2002 to 2013. Cardiovasc Diabetol. 2016; 15(1): 131.

Karalis IK, Alegakis AK, Kafatos AG, Koutis AD, Vardas PE, Lionis CD. Risk factors for ischaemic heart disease in a Cretan rural population: a twelve year follow-up study. BMC Public Health. 2007; 351.

Karolinska Institute (Sweden), Statistics Sweden, Stockholm County Council. Sweden - Stockholm Public Health Survey 2006-2007.

Katte J-C, Dzudie A, Sobngwi E, Mbong EN, Fetse GT, Kouam CK, Kengne A-P. Coincidence of diabetes mellitus and hypertension in a semi-urban Cameroonian population: a cross-sectional study. BMC Public Health. 2014; 14: 696.

Katulanda P, Constantine GR, Mahesh JG, Sheriff R, Seneviratne RD, Wijeratne S, Wijesuriya M, McCarthy MI, Adler AI, Matthews DR. Prevalence and projections of diabetes and pre-diabetes in adults in Sri Lanka--Sri Lanka Diabetes, Cardiovascular Study (SLDCS). Diabet Med. 2008; 25(9): 1062-9.

Kazakh Academy of Nutrition, Ministry of Education and Science (Kazakhstan), Republican State Enterprise (Kazakhstan). Kazakhstan Complex Prevention of the Epidemic of Excessive Body Mass and Obesity 2012.

Kelestimur F, Cetin M, Pasaoglu H, Coksevim B, Cetinkaya F, Unlühizarci K, Unal S, Köker AH. The prevalence and identification of risk factors for type 2 diabetes mellitus and impaired glucose tolerance in Kayseri, central Anatolia, Turkey. Acta Diabetol. 1999; 36(1-2): 85-91.

Kelly HA, Russell MT, Jones TW, Byrne GC. Dramatic increase in incidence of insulin dependent diabetes mellitus in Western Australia. Med J Aust. 1994; 161(7): 426-9.

Kenya National Bureau of Statistics, Ministry of Health (Kenya), World Health Organization (WHO). Kenya STEPS Noncommunicable Disease Risk Factors Survey 2015.

Khader Y, Batieha A, Ajlouni H, El-Khateeb M, Ajlouni K. Obesity in Jordan: prevalence, associated factors, comorbidities, and change in prevalence over ten years. Metab Syndr Relat Disord. 2008; 6(2): 113-20.

Khan FS, Lotia-Farrukh I, Khan AJ, Siddiqui ST, Sajun SZ, Malik AA, Burfat A, Arshad MH, Codlin AJ, Reininger BM, McCormick JB, Afridi N, Fisher-Hoch SP. The burden of non-communicable disease in transition communities in an Asian megacity: baseline findings from a cohort study in Karachi, Pakistan. PLoS One. 2013; 8(2): e56008.

Khater S, Aouar A, Bensmain N, Bendedouche S, Chabni N, Hamdaoui H, Moussouni A, Moqaddem Z. Very High Incidence of Type 1 Diabetes Among Children Aged Under 15 Years in Tlemcen, Northwest Algeria (2015-2018). J Clin Res Pediatr Endocrinol. 2021; 13(1): 44-51.

Khebir BV, Osman A, Khalid BA. Changing prevalence of diabetes mellitus amongst rural Malays in Kuala Selangor over a 10-year period. Med J Malaysia. 1996; 51(1): 41-7.

Khosravi A, Emamian MH, Hashemi H, Fotouhi A. Obesity is the Most Important Factor for Gender Inequality in Type 2 Diabetes Incidence in an Iranian Population. Int J Prev Med. 2019; 10: 215.

Khosravi-Boroujeni H, Sarrafzadegan N, Sadeghi M, Roohafza H, Ng SK, Ahmed F. Determinants of Changes in Metabolic Syndrome Components in a 12-Year Cohort of Iranian Adults. Metab Syndr Relat Disord. 2019; 17(2): 120-127.

Kida K, Mimura G, Ito T, Murakami K, Ashkenazi I, Laron Z. Incidence of Type 1 diabetes mellitus in children aged 0-14 in Japan, 1986-1990, including an analysis for seasonality of onset and month of birth: JDS study. The Data Committee for Childhood Diabetes of the Japan Diabetes Society (JDS). Diabet Med. 2000; 17(1): 59-63.

Kim JH, Lee CG, Lee YA, Yang SW, Shin CH. Increasing incidence of type 1 diabetes among Korean children and adolescents: analysis of data from a nationwide registry in Korea. Pediatr Diabetes. 2016; 17(7): 519–24.

Kim JM. Vascular disease/risk and late-life depression in a Korean community population. Br J Psychiatry. 2004; 185(2): 102-7.

Kim SG, Yang SW, Jang AS, Seo JP, Han SW, Yeom CH, Kim YC, Oh SH, Kim JS, Nam HS, Chung DJ, Chung MY. Prevalence of diabetes mellitus in the elderly of Namwon County, South Korea. Korean J Intern Med. 2002; 17(3): 180-90.

King H, Abdullaev B, Djumaeva S, Nikitin V, Ashworth L, Dobo MG. Glucose intolerance and associated factors in the Fergana Valley, Uzbekistan. Diabet Med. 1998; 15(12): 1052–62.

King H, Taylor R, Koteka G, Nemaia H, Zimmet P, Bennett PH, Raper LR. Glucose tolerance in Polynesia. Population-based surveys in Rarotonga and Niue. Med J Aust. 1986; 145(10): 505-10.

Knip M, Parviainen A, Turtinen M, But A, Härkönen T, Hepojoki J, Sironen T, Iheozor-Ejiofor R, Ugurlu H, Saksela K, Lempainen J, Ilonen J, Vapalahti O, Finnish Pediatric Diabetes Register. SARS-CoV-2 and type 1 diabetes in children in Finland: an observational study. Lancet Diabetes Endocrinol. 2023; 11(4): 251-260.

Kobayashi J, Nishimura K, Matoba M, Maekawa N, Mabuchi H. Generation and Gender Differences in the Components Contributing to the Diagnosis of the Metabolic Syndrome According to the Japanese Criteria. Circ J. 2007; 71(11): 1734-7.

Korea Centers for Disease Control and Prevention (KCDC). South Korea National Health and Nutrition Examination Survey 1998. Osong-eup, Republic of Korea: Korea Centers for Disease Control and Prevention (KCDC).

Korea Centers for Disease Control and Prevention (KCDC). South Korea National Health and Nutrition Examination Survey 2001. Osong-eup, Republic of Korea: Korea Centers for Disease Control and Prevention (KCDC).

Korea Centers for Disease Control and Prevention (KCDC). South Korea National Health and Nutrition Examination Survey 2005. Osong-eup, Republic of Korea: Korea Centers for Disease Control and Prevention (KCDC).

Korea Centers for Disease Control and Prevention (KCDC). South Korea National Health and Nutrition Examination Survey 2007. Osong-eup, Republic of Korea: Korea Centers for Disease Control and Prevention (KCDC).

Korea Centers for Disease Control and Prevention (KCDC). South Korea National Health and Nutrition Examination Survey 2008. Osong-eup, Republic of Korea: Korea Centers for Disease Control and Prevention (KCDC).

Korea Centers for Disease Control and Prevention (KCDC). South Korea National Health and Nutrition Examination Survey 2009. Osong-eup, Republic of Korea: Korea Centers for Disease Control and Prevention (KCDC).

Korea Centers for Disease Control and Prevention (KCDC). South Korea National Health and Nutrition Examination Survey 2010. Osong-eup, Republic of Korea: Korea Centers for Disease Control and Prevention (KCDC).

Korea Centers for Disease Control and Prevention (KCDC). South Korea National Health and Nutrition Examination Survey 2011. Osong-eup, Republic of Korea: Korea Centers for Disease Control and Prevention (KCDC).

Korea Centers for Disease Control and Prevention (KCDC). South Korea National Health and Nutrition Examination Survey 2012. Osong-eup, Republic of Korea: Korea Centers for Disease Control and Prevention (KCDC).

Korea Centers for Disease Control and Prevention (KCDC). South Korea National Health and Nutrition Examination Survey 2013. Osong-eup, Republic of Korea: Korea Centers for Disease Control and Prevention (KCDC).

Korea Centers for Disease Control and Prevention (KCDC). South Korea National Health and Nutrition Examination Survey 2014. Osong-eup, Republic of Korea: Korea Centers for Disease Control and Prevention (KCDC).

Korea Centers for Disease Control and Prevention (KCDC). South Korea National Health and Nutrition Examination Survey 2015. Osong-eup, Republic of Korea: Korea Centers for Disease Control and Prevention (KCDC).

Korea Centers for Disease Control and Prevention (KCDC). South Korea National Health and Nutrition Examination Survey 2016. Osong-eup, Republic of Korea: Korea Centers for Disease Control and Prevention (KCDC).

Korea Centers for Disease Control and Prevention (KCDC). South Korea National Health and Nutrition Examination Survey 2017. Osong-eup, Republic of Korea: Korea Centers for Disease Control and Prevention (KCDC).

Korea Centers for Disease Control and Prevention (KCDC). South Korea National Health and Nutrition Examination Survey 2018. Osong-eup, Republic of Korea: Korea Centers for Disease Control and Prevention (KCDC).

Korea Centers for Disease Control and Prevention (KCDC). South Korea National Health and Nutrition Examination Survey 2019. Osong-eup, Republic of Korea: Korea Centers for Disease Control and Prevention (KCDC).

Korea Centers for Disease Control and Prevention (KCDC). South Korea National Health and Nutrition Examination Survey 2020. Osong-eup, Republic of Korea: Korea Centers for Disease Control and Prevention (KCDC).

Kretowski A, Kowalska I, Peczyñska J, Urban M, Green A, Kinalska I. The large increase in incidence of Type I diabetes mellitus in Poland. Diabetologia. 2001; B48-50.

Krishnadath ISK, Nahar-van Venrooij LM, Jaddoe VWV, Toelsie JR. Ethnic differences in prediabetes and diabetes in the Suriname Health Study. BMJ Open Diabetes Res Care. 2016; 4(1): e000186.

Kumma WP, Lindtjørn B, Loha E. Prevalence of hypertension, and related factors among adults in Wolaita, southern Ethiopia: A community-based cross-sectional study. PLoS One. 2021; 16(12): e0260403.

Kuo R-J, Wu Y-H, Chen L-K. Inability of waist-to-height ratio to predict new onset diabetes mellitus among older adults in Taiwan: a five-year observational cohort study. Arch Gerontol Geriatr. 2011; 53(1): e1–4.

Kweon S-S, Shin M-H, Park K-S, Nam H-S, Jeong S-K, Ryu S-Y, Chung E-K, Choi J-S. Distribution of the ankle-brachial index and associated cardiovascular risk factors in a population of middle-aged and elderly koreans. J Korean Med Sci. 2005; 20(3): 373-8.

Lai S-W, Tan C-K. Epidemiology of Hyperglycemia in Elderly Persons. J Gerontol A Biol Sci Med Sci. 2000; 55A(5): M257-9.

Laing SP, Swerdlow AJ, Slater SD, Botha JL, Burden AC, Waugh NR, Smith AW, Hill RD, Bingley PJ, Patterson CC, Qiao Z, Keen H. The British Diabetic Association Cohort Study, I: all-cause mortality in patients with insulin-treated diabetes mellitus. Diabet Med. 1999; 16(6): 459-65.

Lapertosa S GC, Benítez J, Céspedes MS, Bordón C, de Loredo L, Santoro S, de Sereday M. Prevalencia de Diabetes Mellitus tipo 2 en población adulta de Gobernador Virasoro, Provincia de Corrientes. Rev Asoc Latinoam Diabetes. 2009; 89-96.

Larenas G, Montecinos A, Manosalva M, Barthou M, Vidal T. Incidence of insulin-dependent diabetes mellitus in the IX region of Chile: ethnic differences. Diabetes Res Clin Pract. 1996; S147-51.

Le C, Jun D, Zhankun S, Yichun L, Jie T. Socioeconomic differences in diabetes prevalence, awareness, and treatment in rural southwest China. Trop Med Int Health. 2011; 16(9): 1070-6.

Lee H-S, Park Y-M, Kwon H-S, Lee J-H, Park YJ, Lim SY, Lee S-H, Yoon K-H, Son H-Y, Kim DS, Yim HW, Lee W-C. Prevalence, awareness, treatment, and control of hypertension among people over 40 years old in a rural area of South Korea: The Chungju Metabolic Disease Cohort (CMC) Study. Clin Exp Hypertens. 2010; 32(3): 166–78.

Lee HY, Won JC, Kang YJ, Yoon SH, Choi EO, Bae JY, Sung MH, Kim H-R, Yang JH, Oh J, Lee YM, Park NH, Ko KS, Rhee BD. Type 2 diabetes in urban and rural districts in Korea: factors associated with prevalence difference. J Korean Med Sci. 2010; 25(12): 1777-83.

Lessa I, Magalhães L, Araújo MJ, de Almeida Filho N, Aquino E, Oliveira MM. Arterial hypertension in the adult population of Salvador (BA)--Brazil. Arq Bras Cardiol. 2006; 87(6): 747-56.

Levitt NS, Katzenellenbogen JM, Bradshaw D, Hoffman MN, Bonnici F. The prevalence and identification of risk factors for NIDDM in urban Africans in Cape Town, South Africa. Diabetes Care. 1993; 16(4): 601-7.

Levy-Marchal C, Czernichow P. Heterogeneity of type 1 diabetes at onset in children: results from the French Incidence Study. Diabetes Metab. 1993; 19(3): 296-303.

Li H, Guo M, An Z, Meng J, Jiang J, Song J, Wu W. Prevalence and Risk Factors of Metabolic Associated Fatty Liver Disease in Xinxiang, China. Int J Environ Res Public Health. 2020; 17(6).

Li HL, Fang J, Zhao LG, Liu DK, Wang J, Han LH, Xiang YB. Personal Characteristics Effects on Validation of Self-reported Type 2 Diabetes From a Cross-sectional Survey Among Chinese Adults. J Epidemiol. 2020; 30(11): 516-521.

Li N, Huang C, Lan B, Lin D, Wang C, You L, Li L, Li F, Xiao H, Yan L, Lao G, Sun K. Association of gonadal hormones and sex hormone binding globulin with risk of diabetes: A cohort study in middle-aged and elderly Chinese males. Int J Clin Pract. 2021; 75(5): e14008.

Li R, Chen G, Jiao A, Lu Y, Guo Y, Li S, Wang C, Xiang H. Residential Green and Blue Spaces and Type 2 Diabetes Mellitus: A Population-Based Health Study in China. Toxics. 2021; 9(1).

Li W, Wang D, Wang X, Gong Y, Cao S, Yin X, Zhuang X, Shi W, Wang Z, Lu Z. The association of metabolic syndrome components and diabetes mellitus: evidence from China National Stroke Screening and Prevention Project. BMC Public Health. 2019; 19(1): 192.

Li Y, Teng D, Shi X, Qin G, Qin Y, Quan H, Shi B, Sun H, Ba J, Chen B, Du J, He L, Lai X, Li Y, Chi H, Liao E, Liu C, Liu L, Tang X, Tong N, Wang G, Zhang JA, Wang Y, Xue Y, Yan L, Yang J, Yang L, Yao Y, Ye Z, Zhang Q, Zhang L, Zhu J, Zhu M, Ning G, Mu Y, Zhao J, Teng W, Shan Z. Prevalence of diabetes recorded in mainland China using 2018 diagnostic criteria from the American Diabetes Association: national cross sectional study. BMJ. 2020; 369: m997.

Li Y, Zhao Z, Wang L, Fu Z, Ji L, Wu X. The Prevalence of Osteoporosis Tested by Quantitative Computed Tomography in Patients With Different Glucose Tolerances. J Clin Endocrinol Metab. 2020; 105(1).

Liang J, Zhang Y, Xue A, Sun J, Song X, Xue B, Ji F, Gao W, He L, Pang Z, Qiao Q, Ning F. Association between fruit, vegetable, seafood, and dairy intake and a reduction in the prevalence of type 2 diabetes in Qingdao, China. Asia Pac J Clin Nutr. 2017; 26(2): 255-261.

Lim N-K, Park S-H, Choi S-J, Lee K-S, Park H-Y. A risk score for predicting the incidence of type 2 diabetes in a middle-aged Korean cohort: the Korean genome and epidemiology study. Circ J. 2012; 76(8): 1904–10.

Lin C-C, Liu C-S, Li T-C, Chen C-C, Li C-I, Lin W-Y. Microalbuminuria and the metabolic syndrome and its components in the Chinese population. Eur J Clin Invest. 2007; 37(10): 783-90.

Lin L, Chen G, Zou X, Zhao J, Zhu F, Tu M, Xu S, Lin W, Yang S, Zhang Y, Lin M, Chen N, Huang H, Liang J, Li L, Yao J. Diabetes, pre-diabetes and associated risks on Minnesota code-indicated major electrocardiogram abnormality among Chinese: a cross-sectional diabetic study in Fujian province, southeast China. Obes Rev. 2009; 10(4): 420-30.

Lin S, Cheng TO, Liu X, Mai J, Rao X, Gao X, Deng H, Shi M. Impact of dysglycemia, body mass index, and waist-to-hip ratio on the prevalence of systemic hypertension in a lean Chinese population. Am J Cardiol. 2006; 97(6): 839-42.

Lind H, Nilsson P, Holthuis N, Lindholm L. Non-obese men with high lipoprotein(a) values-- a cardiovascular risk group different from those with the metabolic syndrome?. Scand J Clin Lab Invest. 1994; 54(2): 177-83.

Lindblad U, Ek J, Eckner J, Larsson CA, Shan G, Rastam L. Prevalence, awareness, treatment, and control of hypertension: rule of thirds in the Skaraborg project. Scand J Prim Health Care. 2012; 30(2): 88–94.

Ling B, Zhao L, Yi J. Cross-sectional study of the prevalence and risk factors of metabolic syndrome in a rural population of the Qianjiang area. Medicine (Baltimore). 2020; 99(35): e21574.

Linkeviciute-Ulinskiene D, Kaceniene A, Dulskas A, Patasius A, Zabuliene L, Smailyte G. Increased Mortality Risk in People with Type 2 Diabetes Mellitus in Lithuania. Int J Environ Res Public Health. 2020; 17(18): 6870.

Lipman TH, Chang Y, Murphy KM. The epidemiology of type 1 diabetes in children in Philadelphia 1990-1994: evidence of an epidemic. Diabetes Care. 2002; 25(11): 1969-75.

Lipman TH, Jawad AF, Murphy KM, Tuttle A, Thompson RL, Ratcliffe SJ, Levitt Katz LE. Incidence of type 1 diabetes in Philadelphia is higher in black than white children from 1995 to 1999: epidemic or misclassification?. Diabetes Care. 2006; 29(11): 2391-5.

Little M, Humphries S, Patel K, Dodd W, Dewey C. Factors associated with glucose tolerance, pre-diabetes, and type 2 diabetes in a rural community of south India: a cross-sectional study. Diabetol Metab Syndr. 2016; 8: 21.

Liu C, Yuan YC, Guo MN, Xin Z, Chen GJ, Bentley AR, Hua L, Zheng JP, Ekoru K, Yang JK. Incidence of Type 1 Diabetes May Be Underestimated in the Chinese Population: Evidence From 21.7 Million People Between 2007 and 2017. Diabetes Care. 2021; 44(11): 2503-2509.

Liu F, Ma Y-T, Yang Y-N, Zhen Y-J, Xie X, Li X-M, Ma X, Chen B-D, Huang Y, Shan C-F, Gao X-M. The prevalence of isolated systolic hypertension in adult populations from the Han, Uygur and Kazakh ethnic groups in Xinjiang, China. Blood Press. 2014; 23(3): 154–9.

Liu L, Zhou C, Du H, Zhang K, Huang D, Wu J. The prevalences of impaired fasting glucose and diabetes mellitus in working age men of North China: Anshan Worker Health Survey. Sci Rep. 2014; 4835.

Liu M, Yang S, Wang S, Zhao Y, Zhu Q, Ning C, He Y. Distribution of blood glucose and prevalence of diabetes among centenarians and oldest-old in China: based on the China Hainan Centenarian Cohort Study and China Hainan Oldest-old Cohort Study. Endocrine. 2020; 70(2): 314-322.

Liu S, Wang W, Zhang J, He Y, Yao C, Zeng Z, Piao J, Howard BV, Fabsitz RR, Best L, Yang X, Lee ET. Prevalence of diabetes and impaired fasting glucose in Chinese adults, China National Nutrition and Health Survey, 2002. Prev Chronic Dis. 2011; 8(1): A13.

López-Siguero JP, Del Pino-De la Fuente A, Martínez-Aedo MJ, Moreno-Molina JA. Increased incidence of type 1 diabetes in the south of Spain. Diabetes Care. 2002; 25(6): 1099.

Lora-Gómez RE, Morales-Pérez FM, Arroyo-Díez FJ, Barquero-Romero J. Incidence of Type 1 diabetes in children in Cáceres, Spain, during 1988-1999. Diabetes Res Clin Pract. 2005; 69(2): 169-74.

Lu K, Chen J, Wu S, Chen J, Hu D. Interaction of Sleep Duration and Sleep Quality on Hypertension Prevalence in Adult Chinese Males. J Epidemiol. 2015; 25(6): 415–22.

Lu S, Bao MY, Miao SM, Zhang X, Jia QQ, Jing SQ, Shan T, Wu XH, Liu Y. Prevalence of hypertension, diabetes, and dyslipidemia, and their additive effects on myocardial infarction and stroke: a cross-sectional study in Nanjing, China. Ann Transl Med. 2019; 7(18): 436.

Lv Y-B, Yin Z-X, Chei C-L, Qian H-Z, Kraus VB, Zhang J, Brasher MS, Shi X-M, Matchar DB, Zeng Y. Low-density lipoprotein cholesterol was inversely associated with 3-year all-cause mortality among Chinese oldest old: data from the Chinese Longitudinal Healthy Longevity Survey. Atherosclerosis. 2015; 239(1): 137–42.

Maffeis C, Mancioppi V, Piona C, Avossa F, Fedeli U, Marigliano M. Type 1 diabetes prevalence and incidence rates in the pediatric population of Veneto Region (Italy) in 2015-2020. Diabetes Res Clin Pract. 2021; 179: 109020.

Malavige GN, de Alwis NM, Weerasooriya N, Fernando DJ, Siribaddana SH. Increasing diabetes and vascular risk factors in a sub-urban Sri Lankan population. Diabetes Res Clin Pract. 2002; 57(2): 143-5.

Malerbi DA, Franco LJ. Multicenter study of the prevalence of diabetes mellitus and impaired glucose tolerance in the urban Brazilian population aged 30-69 yr. The Brazilian Cooperative Group on the Study of Diabetes Prevalence. Diabetes Care. 1992; 15(11): 1509-16.

Malik M, Bakir A, Saab BA, Roglic G, King H. Glucose intolerance and associated factors in the multi-ethnic population of the United Arab Emirates results of a national survey. Diabetes Res Clin Pract. 2005; 69(2): 188-95.

Malta DC, Duncan BB, Schmidt MI, Machado ÍE, Silva AGD, Bernal RTI, Pereira CA, Damacena GN, Stopa SR, Rosenfeld LG, Szwarcwald CL. Prevalence of diabetes mellitus as determined by glycated hemoglobin in the Brazilian adult population, National Health Survey. Rev Bras Epidemiol. 2019; 22Suppl 02(Suppl 02): E190006SUPL2.

Mamoulakis D, Vrouvaki F, Louvari V, Galanakis E. Incidence of childhood Type 1 diabetes mellitus in Crete. Diabet Med. 2018.

Mannami T, Baba S, Ogata J. Potential of Carotid Enlargement as a Useful Indicator Affected by High Blood Pressure in a Large General Population of a Japanese City The Suita Study. Stroke. 2000; 31(12): 2958-65.

Manuwald U, Schoffer O, Hegewald J, Große J, Kugler J, Kapellen TM, Kiess W, Rothe U. Ketoacidosis at onset of type 1 diabetes in children up to 14 years of age and the changes over a period of 18 years in Saxony, Eastern-Germany: A population based register study. PLoS One. 2019; 14(6): e0218807.

Marcopito LF, Rodrigues SSF, Pacheco MA, Shirassu MM, Goldfeder AJ, de Moraes MAOL. Prevalence of a set of risk factors for chronic diseases in the city of Sao Paulo, Brazil. Rev Saude Publica. 2005; 39(5): 738-45.

Marques-Vidal P, Vollenweider P, Waeber G. Alcohol consumption and incidence of type 2 diabetes. Results from the CoLaus study. Nutr Metab Cardiovasc Dis. 2015; 25(1): 75–84.

Martinucci ME, Curradi G, Fasulo A, Medici A, Toni S, Osovik G, Lapistkaya E, Sherbitskaya E. Incidence of childhood type 1 diabetes mellitus in Gomel, Belarus. J Pediatr Endocrinol Metab. 2002; 15(1): 53-7.

Marzouk D, Sass J, Bakr I, El Hosseiny M, Abdel-Hamid M, Rekacewicz C, Chaturvedi N, Mohamed MK, Fontanet A. Metabolic and cardiovascular risk profiles and hepatitis C virus infection in rural Egypt. Gut. 2007; 56(8): 1105-10.

Mathur P, Kulothungan V, Leburu S, Krishnan A, Chaturvedi HK, Salve HR, Amarchand R, Nongkynrih B, Kumar PG, K S VU, Ramakrishnan L, Laxmaiah A, Boruah M, Kumar S, Patro BK, Raghav PR, Rajkumar P, Sarma PS, Sharma R, Tambe M, Thankappan KR, Arlappa N, Mahanta TG, Joshi RP, Rustagi N, Gupta S, Behera BK, Shelke SC, Galhotra A, Bhuyan PJ, Pakhare AP, Kumar D, Topno RK, Gupta MK, Trivedi AV, Garg S. National noncommunicable disease monitoring survey (NNMS) in India: Estimating risk factor prevalence in adult population. PLoS One. 2021; 16(3): e0246712.

Matshipi M, Monyeki KD, Kemper H. The Relationship between Physical Activity and Plasma Glucose Level amongst Ellisras Rural Young Adult Males and Females: Ellisras Longitudinal Study. Int J Environ Res Public Health. 2017; 14(2).

Mauny F, Grandmottet M, Lestradet C, Guitard J, Crenn D, Floret N, Olivier-Koehret M, Viel JF. Increasing trend of childhood type 1 diabetes in Franche-Comté‚ (France): analysis of age and period effects from 1980 to 1998. Eur J Epidemiol. 2005; 20(4): 325-9.

Mayega RW, Guwatudde D, Makumbi F, Nakwagala FN, Peterson S, Tomson G, Ostenson C-G. Diabetes and pre-diabetes among persons aged 35 to 60 years in eastern Uganda: prevalence and associated factors. PLoS One. 2013; 8(8): e72554.

Mazzella M, Cotellessa M, Bonassi S, Mulas R, Caratozzolo A, Gaber S, Romano C. Incidence of type I diabetes in the Liguria Region, Italy. Results of a prospective study in a 0- to 14-year age-group. Diabetes Care. 1994; 17(10): 1193-6.

Mbanya JC, Ngogang J, Salah JN, Minkoulou E, Balkau B. Prevalence of NIDDM and impaired glucose tolerance in a rural and an urban population in Cameroon. Diabetologia. 1997; 40(7): 824-9.

McCullough ML, Wan N, Pezzolesi MG, Collins TW, Grineski SE, Wei YD, Lazaro-Guevara J, Frodsham SG, Vanderslice JA, Holmen JR, Srinivas TR, Clements SA. Type 1 Diabetes incidence among youth in Utah: A geographical analysis. Soc Sci Med. 2021; 278: 113952.

McKenna A, O'Regan M, Ryder K, Fitzgerald H, Hoey H, Roche E. Incidence of childhood type 1 diabetes mellitus in Ireland remains high but no longer rising. Acta Paediatr. 2021; 110(7): 2142-2148.

Mebrahtu G, Maniam J, James S, Ogle GD. High incidence of type 1 diabetes in adolescents and young adults in Eritrea. Diabet Med. 2021; 38(7): e14544.

Medina-Lezama J, Zea-Diaz H, Morey-Vargas OL, Bolaños-Salazar JF, Postigo-Macdowall M, Paredes-Díaz S, Corrales-Medina F, Valdivia-Ascuña Z, Cuba-Bustinza C, Villalobos-Tapia P, Muñoz-Atahualpa E, Chirinos-Pacheco J, Raij L, Chirinos JA. Prevalence and patterns of hypertension in Peruvian Andean Hispanics: the PREVENCION study. J Am Soc Hypertens. 2007; 1(3): 216-25.

Meiloud G, Arfa I, Kefi R, Abdelhamid I, Veten F, Lasram K, Ben Halim N, Sidi Mhamed A, Samb A, Abdelhak S, Houmeida AO. Type 2 diabetes in Mauritania: prevalence of the undiagnosed diabetes, influence of family history and maternal effect. Prim Care Diabetes. 2013; 7(1): 19-24.

Meisinger C, Strassburger K, Heier M, Thorand B, Baumeister SE, Giani G, Rathmann W. Prevalence of undiagnosed diabetes and impaired glucose regulation in 35-59-year-old individuals in Southern Germany: the KORA F4 Study. Diabet Med. 2010; 27(3): 360–2.

Melander O, Maisel AS, Almgren P, Manjer J, Belting M, Hedblad B, Engstrom G, Kilger U, Nilsson P, Bergmann A, Orho-Melander M. Plasma proneurotensin and incidence of diabetes, cardiovascular disease, breast cancer, and mortality. JAMA. 2012; 308(14): 1469–75.

Menotti A, Lanti M, Angeletti M, Panarelli W, Scavizzi P, Botta G, Cirillo M, Laurenzi M, Mancini M, Terradura-Vagnarelli O, Zanchetti A. Twenty-year cardiovascular and all-cause mortality trends and changes in cardiovascular risk factors in Gubbio, Italy: The role of blood pressure changes. J Hypertens. 2009; 27(2): 266-74.

Menzies Research Institute Tasmania, Ministry of Health (Vietnam). Vietnam STEPS Noncommunicable Disease Risk Factors Survey 2009.

Metcalfe MA, Baum JD. Incidence of insulin dependent diabetes in children aged under 15 years in the British Isles during 1988. BMJ. 1991; 302(6774): 443-7.

Metelskaya VA, Shkolnikova MA, Shalnova SA, Andreev EM, Deev AD, Jdanov DA, Shkolnikov VM, Vaupel JW. Prevalence, components, and correlates of metabolic syndrome (MetS) among elderly Muscovites. Arch Gerontol Geriatr. 2012; 55(2): 231–7.

Michalkov  DM, Cernay J, Dankov  A, Rusn k M, Fand kov  K. Incidence and prevalence of childhood diabetes in Slovakia (1985-1992). Slovak Childhood Diabetes Epidemiology Study Group. Diabetes Care. 1995; 18(3): 315-20.

Miettola J, Nykanen I, Kumpusalo E. Health views and metabolic syndrome in a Finnish rural community: a cross-sectional population study. Can J Rural Med. 2012; 17(1): 10–6.

Mihardja L, Delima, Manz HS, Ghani L, Soegondo S. Prevalence and determinants of diabetes mellitus and impaired glucose tolerance in Indonesia (a part of basic health research/Riskesdas). Acta Med Indones. 2009; 41(4): 169–74.

Ministry of Health (Barbados). Barbados STEPS Noncommunicable Disease Risk Factors Survey 2007.

Ministry of Health (Benin), World Health Organization (WHO). Benin - Littoral STEPS Noncommunicable Disease Risk Factors Survey 2007.

Ministry of Health (Benin), World Health Organization (WHO). Benin STEPS Noncommunicable Disease Risk Factors Survey 2008.

Ministry of Health (Benin), World Health Organization (WHO). Benin STEPS Noncommunicable Disease Risk Factors Survey 2015.

Ministry of Health (Bhutan), World Health Organization (WHO). Bhutan - Thimphu STEPS Noncommunicable Disease Risk Factors Survey 2007.

Ministry of Health (Bhutan), World Health Organization (WHO). Bhutan STEPS Noncommunicable Disease Risk Factors Survey 2014.

Ministry of Health (Bhutan), World Health Organization (WHO). Bhutan STEPS Noncommunicable Disease Risk Factors Survey 2019. WHO NCD Microdata Repository, 2021.

Ministry of Health (Botswana), World Health Organization (WHO). Botswana STEPS Noncommunicable Disease Risk Factors Survey 2014.

Ministry of Health (Burkina Faso), West African Health Organization, World Health Organization (WHO). Burkina Faso STEPS Noncommunicable Disease Risk Factors Survey 2013.

Ministry of Health (Cambodia), University of Health Sciences (Cambodia), World Health Organization (WHO). Cambodia STEPS Noncommunicable Disease Risk Factors Survey 2010.

Ministry of Health (Congo, Rep.), World Health Organization (WHO). Congo - Brazzaville STEPS Noncommunicable Disease Risk Factors Survey 2004.

Ministry of Health (Cook Islands), World Health Organization (WHO). Cook Islands STEPS Noncommunicable Disease Risk Factors Survey 2013-2015.

Ministry of Health (Guyana), World Health Organization (WHO). Guyana STEPS Noncommunicable Disease Risk Factors Survey 2016.

Ministry of Health (Indonesia), World Health Organization (WHO). Indonesia - Jawa Barat STEPS Noncommunicable Disease Risk Factors Survey 2003.

Ministry of Health (Indonesia), World Health Organization (WHO). Indonesia - Jawa Barat STEPS Noncommunicable Disease Risk Factors Survey 2006.

Ministry of Health (Iraq), Ministry of Planning (Iraq), World Health Organization (WHO). Iraq STEPS Noncommunicable Disease Risk Factors Survey 2015.

Ministry of Health (Italy), National Association of Hospital Cardiologists (Italy), National Center for Epidemiology, Surveillance, and Health Promotion (Italy), National Institute of Health (Italy). Italy Cardiovascular Epidemiological Observatory/ Health Examination Survey 2008-2012.

Ministry of Health (Kuwait), World Health Organization (WHO). Kuwait STEPS Noncommunicable Disease Risk Factors Survey 2006.

Ministry of Health (Kuwait), World Health Organization (WHO). Kuwait STEPS Noncommunicable Disease Risk Factors Survey 2014.

Ministry of Health (Kyrgyzstan), World Health Organization (WHO). Kyrgyzstan STEPS Noncommunicable Disease Risk Factors Survey 2013.

Ministry of Health (Malawi), World Health Organization (WHO). Malawi STEPS Noncommunicable Disease Risk Factors Survey 2009.

Ministry of Health (Malaysia), World Health Organization (WHO). Malaysia STEPS Noncommunicable Disease Risk Factors Survey 2005-2006.

Ministry of Health (Mali), World Health Organization (WHO). Mali - Kati, Ouéléssébougou, Koulikoro, Ségou and Bamako District STEPS Noncommunicable Disease Risk Factors Survey 2013. WHO NCD Microdata Repository, 2019.

Ministry of Health (Mauritania), World Health Organization (WHO). Mauritania - Nouakchott STEPS Noncommunicable Disease Risk Factors Survey 2006.

Ministry of Health (Mexico), National Institute of Public Health (Mexico). Mexico National Survey of Health and Nutrition Mid-way 2016.

Ministry of Health (Moldova), National Center of Public Health (Moldova), National Office of Statistics (Moldova), World Health Organization (WHO). Moldova STEPS Noncommunicable Disease Risk Factors Survey 2013.

Ministry of Health (Morocco), World Health Organization (WHO). Morocco STEPS Noncommunicable Disease Risk Factors Survey 2017.

Ministry of Health (Mozambique), World Health Organization (WHO). Mozambique STEPS Noncommunicable Disease Risk Factors Survey 2005.

Ministry of Health (Myanmar), Myanmar Medical Association, World Health Organization (WHO). Myanmar STEPS Noncommunicable Disease Risk Factors Survey 2014. Geneva, Switzerland: World Health Organization (WHO).

Ministry of Health (Myanmar), World Health Organization (WHO). Myanmar - Yangon STEPS Noncommunicable Disease Risk Factors Survey 2003.

Ministry of Health (Nauru), World Health Organization (WHO). Nauru STEPS Noncommunicable Disease Risk Factors Survey 2015-2016. Geneva, Switzerland: World Health Organization (WHO).

Ministry of Health (New Zealand). New Zealand Virtual Diabetes Register 2005.

Ministry of Health (New Zealand). New Zealand Virtual Diabetes Register 2006.

Ministry of Health (New Zealand). New Zealand Virtual Diabetes Register 2007.

Ministry of Health (New Zealand). New Zealand Virtual Diabetes Register 2008.

Ministry of Health (New Zealand). New Zealand Virtual Diabetes Register 2009.

Ministry of Health (New Zealand). New Zealand Virtual Diabetes Register 2010.

Ministry of Health (New Zealand). New Zealand Virtual Diabetes Register 2011.

Ministry of Health (New Zealand). New Zealand Virtual Diabetes Register 2012.

Ministry of Health (New Zealand). New Zealand Virtual Diabetes Register 2013.

Ministry of Health (New Zealand). New Zealand Virtual Diabetes Register 2014.

Ministry of Health (New Zealand). New Zealand Virtual Diabetes Register 2015.

Ministry of Health (New Zealand). New Zealand Virtual Diabetes Register 2016.

Ministry of Health (New Zealand). New Zealand Virtual Diabetes Register 2017. Wellington, New Zealand: Ministry of Health (New Zealand), 2018.

Ministry of Health (New Zealand). New Zealand Virtual Diabetes Register 2018. Wellington, New Zealand: Ministry of Health (New Zealand).

Ministry of Health (New Zealand). New Zealand Virtual Diabetes Register 2019. Wellington, New Zealand: Ministry of Health (New Zealand).

Ministry of Health (New Zealand). New Zealand Virtual Diabetes Register 2020. Wellington, New Zealand: Ministry of Health (New Zealand).

Ministry of Health (Niger), World Health Organization (WHO). Niger STEPS Noncommunicable Disease Risk Factors Survey 2007.

Ministry of Health (Oman), World Health Organization (WHO). Oman STEPS Noncommunicable Disease Risk Factors Survey 2017.

Ministry of Health (Oman), World Health Organization (WHO). Oman World Health Survey 2007-2008 - Gulf Cooperation Countries.

Ministry of Health (Palau), World Health Organization (WHO). Palau STEPS Noncommunicable Disease Risk Factors Survey 2011-2013. Geneva, Switzerland: World Health Organization (WHO).

Ministry of Health (Palau), World Health Organization (WHO). Palau STEPS Noncommunicable Disease Risk Factors Survey 2016. WHO NCD Microdata Repository, 2020.

Ministry of Health (Palestine), World Health Organization (WHO). Palestine STEPS Noncommunicable Disease Risk Factors Survey 2010-2011. Geneva, Switzerland: World Health Organization (WHO).

Ministry of Health (Peru), National Center for Food and Nutrition, National Institute of Health (Peru), National Institute of Health (Peru). Peru National Survey on Nutritional, Biochemical, Socioeconomic Indicators Related to Chronic Degenerative Diseases 2004-2005.

Ministry of Health (Saint Lucia), World Health Organization (WHO). Saint Lucia STEPS Noncommunicable Disease Risk Factors Survey 2012.

Ministry of Health (Samoa). Samoa STEPS Noncommunicable Disease Risk Factors Survey 2002.

Ministry of Health (Sao Tome and Principe), World Health Organization (WHO). São Tomé and Príncipe STEPS Noncommunicable Disease Risk Factors Survey 2019. WHO NCD Microdata Repository, 2022.

Ministry of Health (Saudi Arabia), World Health Organization (WHO). Saudi Arabia STEPS Noncommunicable Disease Risk Factors Survey 2004-2005.

Ministry of Health (Singapore). Singapore National Health Survey 1992.

Ministry of Health (Singapore). Singapore National Health Survey 1998.

Ministry of Health (Singapore). Singapore National Health Survey 2004.

Ministry of Health (Singapore). Singapore National Health Survey 2010.

Ministry of Health (Singapore). Singapore National Population Health Survey 2016-2017. Singapore, Singapore: Ministry of Health (Singapore).

Ministry of Health (Solomon Islands), World Health Organization (WHO). Solomon Islands STEPS Noncommunicable Disease Risk Factors Survey 2015. WHO NCD Microdata Repository, 2020.

Ministry of Health (Swaziland), World Health Organization (WHO). Swaziland STEPS Noncommunicable Disease Risk Factors Survey 2007.

Ministry of Health (Swaziland), World Health Organization (WHO). Swaziland STEPS Noncommunicable Disease Risk Factors Survey 2014.

Ministry of Health (Tajikistan), World Health Organization (WHO). Tajikistan STEPS Noncommunicable Disease Risk Factors Survey 2016.

Ministry of Health (Timor-Leste), National University of East Timor, World Health Organization (WHO). Timor-Leste STEPS Noncommunicable Disease Risk Factors Survey 2014.

Ministry of Health (Togo), West African Health Organization, World Health Organization (WHO). Togo STEPS Noncommunicable Disease Risk Factors Survey 2010-2011.

Ministry of Health (Tonga), World Health Organization (WHO). Tonga STEPS Noncommunicable Disease Risk Factors Survey 2004.

Ministry of Health (Tonga), World Health Organization (WHO). Tonga STEPS Noncommunicable Disease Risk Factors Survey 2011-2012. 2014.

Ministry of Health (Turkey). Turkey Chronic Diseases and Risk Factors Study 2011.

Ministry of Health (Tuvalu), World Health Organization (WHO). Tuvalu STEPS Noncommunicable Disease Risk Factors Survey 2015. Geneva, Switzerland: World Health Organization (WHO), 2020.

Ministry of Health (Uganda), World Health Organization (WHO). Uganda STEPS Noncommunicable Disease Risk Factors Survey 2014. Geneva, Switzerland: World Health Organization (WHO).

Ministry of Health (Uzbekistan), World Bank, World Health Organization (WHO). Uzbekistan STEPS Noncommunicable Disease Risk Factors Survey 2014.

Ministry of Health (Vanuatu), World Health Organization (WHO). Vanuatu STEPS Noncommunicable Disease Risk Factors Survey 2011.

Ministry of Health (Vietnam). Vietnam STEPS Noncommunicable Disease Risk Factors Survey 2015. Geneva, Switzerland: World Health Organization (WHO), 2016.

Ministry of Health (Zambia), World Health Organization (WHO). Zambia - Lusaka STEPS Noncommunicable Disease Risk Factors Survey 2008.

Ministry of Health (Zanzibar), World Health Organization (WHO). Tanzania - Zanzibar STEPS Noncommunicable Disease Risk Factors Survey 2011.

Ministry of Health and Family Welfare (Bangladesh), National Institute of Preventive and Social Medicine, University of Dhaka (Bangladesh), World Health Organization (WHO). Bangladesh STEPS Noncommunicable Disease Risk Factors Survey 2018. Geneva, Switzerland: World Health Organization (WHO).

Ministry of Health and Human Services (Marshall Islands), World Health Organization (WHO). Marshall Islands STEPS Noncommunicable Disease Risk Factors Survey 2017-2018 . Geneva, Switzerland: World Health Organization (WHO), 2020.

Ministry of Health and Medical Education (Iran). Iran STEPS Noncommunicable Disease Risk Factors Survey 2011.

Ministry of Health and Medical Industry (Turkmenistan), State Committee on Statistics of Turkmenistan, World Health Organization (WHO). Turkmenistan STEPS Noncommunicable Disease Risk Factors Survey 2018. WHO NCD Microdata Repository, 2021.

Ministry of Health and Medical Services (Kiribati), World Health Organization (WHO). Kiribati STEPS Noncommunicable Disease Risk Factors Survey 2004-2006.

Ministry of Health and Medical Services (Kiribati), World Health Organization (WHO). Kiribati STEPS Noncommunicable Disease Risk Factors Survey 2016.

Ministry of Health and Population (Egypt), United States Agency for International Development (USAID), World Health Organization (WHO). Egypt STEPS Noncommunicable Disease Risk Factors Survey 2005.

Ministry of Health and Population (Egypt), United States Agency for International Development (USAID), World Health Organization (WHO). Egypt STEPS Noncommunicable Disease Risk Factors Survey 2011-2012.

Ministry of Health and Population (Nepal), Nepal Health Research Council (NHRC), World Health Organization (WHO). Nepal STEPS Noncommunicable Disease Risk Factors Survey 2012-2013.

Ministry of Health and Population (Nepal), Nepal Health Research Council (NHRC), World Health Organization (WHO). Nepal STEPS Noncommunicable Disease Risk Factors Survey 2019. Geneva, Switzerland: World Health Organization (WHO).

Ministry of Health and Quality of Life (Mauritius). Mauritius National Health Survey 2009 (Diabetes Tabulations).

Ministry of Health and Quality of Life (Mauritius). Mauritius National Health Survey 2015 (Diabetes Tabulations).

Ministry of Health and Social Development (Argentina), National Institute of Statistics and Censuses (Argentina). Argentina National Survey of Risk Factors 2018. Buenos Aires, Argentina: National Institute of Statistics and Censuses (Argentina).

Ministry of Health and Social Protection (Colombia), National Consulting Center (Colombia), University of Caldas (Colombia), University of Valle (Colombia). Colombia Health, Well-Being and Aging Survey 2015.

Ministry of Health and Social Welfare (Lesotho), World Health Organization (WHO). Lesotho STEPS Noncommunicable Disease Risk Factors Survey 2012 .

Ministry of Health, Labour and Welfare (Japan), National Institute of Health and Nutrition (Japan). Japan National Health and Nutrition Survey 2018.

Ministry of Health, Labour and Welfare (Japan), National Institute of Health and Nutrition (Japan). Japan National Health and Nutrition Survey 2019.

Ministry of Health, Labour and Welfare (Japan), University of Tokyo. Japan National Health and Nutrition Survey Diabetes Status by Age, Sex, and Prefecture 1990-2017.

Ministry of Health, Population and Hospital Reform (Algeria), World Health Organization (WHO). Algeria - Sétif and Mostaganem STEPS Noncommunicable Disease Risk Factors Survey 2003.

Ministry of Health, Population and Hospital Reform (Algeria), World Health Organization (WHO). Algeria STEPS Noncommunicable Disease Risk Factors Survey 2016-2017.

Ministry of Public Health (Afghanistan), World Health Organization (WHO). Afghanistan STEPS Noncommunicable Disease Risk Factors Survey 2018. Geneva, Switzerland: World Health Organization (WHO).

Ministry of Public Health (Congo, DR), World Health Organization (WHO). Democratic Republic of the Congo - Kinshasa STEPS Noncommunicable Disease Risk Factors Survey 2005. 2021.

Ministry of Public Health (Ecuador), National Institute of Statistics and Censuses (Ecuador). Ecuador National Health and Nutrition Survey 2012.

Ministry of Public Health (Ecuador), World Health Organization (WHO). Ecuador STEPS Noncommunicable Disease Risk Factors Survey 2018. Geneva, Switzerland: World Health Organization (WHO).

Ministry of Public Health (Lebanon), World Health Organization (WHO). Lebanon STEPS Noncommunicable Disease Risk Factors Survey 2016-2017.

Ministry of Public Health (Uruguay), World Health Organization (WHO). Uruguay STEPS Noncommunicable Disease Risk Factors Survey 2006.

Miranda AM, Steluti J, Fisberg RM, Marchioni DM. Association between Coffee Consumption and Its Polyphenols with Cardiovascular Risk Factors: A Population-Based Study. Nutrients. 2017; 9(3).

Modesti PA, Bamoshmoosh M, Rapi S, Massetti L, Bianchi S, Al-Hidabi D, Al Goshae H. Relationship between hypertension, diabetes and proteinuria in rural and urban households in Yemen. J Hum Hypertens. 2013; 27(9): 572-9.

Möller CS, Zethelius B, Sundström J, Lind L. Impact of follow-up time and re-measurement of the electrocardiogram and conventional cardiovascular risk factors on their predictive value for myocardial infarction. J Intern Med. 2006; 260(1): 22-30.

Moon H-W, Park CM, Hong SN, Park S, Hur M, Yun Y-M. Assessment of apoB dyslipoproteinemia in Korean population. Clin Biochem. 2013; 46(12): 1041–6.

Morales DD, Punzalan FER, Paz-Pacheco E, Sy RG, Duante CA. Metabolic syndrome in the Philippine general population: prevalence and risk for atherosclerotic cardiovascular disease and diabetes mellitus. Diab Vasc Dis Res. 2008; 5(1): 36-43.

Morales-Pérez FM, Barquero-Romero J, Pérez-Miranda M. Incidence of type I diabetes among children and young adults (0-29 years) in the province of Badajoz, Spain during 1992 to 1996. Acta Paediatr. 2000; 89(1): 101-4.

Morgan CL, Currie CJ, Peters JR. Relationship between diabetes and mortality: a population study using record linkage. Diabetes Care. 2000; 23(8): 1103-7.

Morrish NJ, Wang SL, Stevens LK, Fuller JH, Keen H. Mortality and causes of death in the WHO Multinational Study of Vascular Disease in Diabetes. Diabetologia. 2001; 44(Suppl 2): S14-21.

Moss SE, Klein R, Klein BE. Cause-specific mortality in a population-based study of diabetes. Am J Public Health. 1991; 81(9): 1158-62.

Motala AA, Esterhuizen T, Gouws E, Pirie FJ, Omar MA. Diabetes and other disorders of glycemia in a rural South African community: prevalence and associated risk factors. Diabetes Care. 2008; 31(9): 1783-8.

Motta M, Bennati E, Capri M, Ferlito L, Malaguarnera M. Diabetes mellitus in the extreme longevity. Exp Gerontol. 2008; 43(2): 102-5.

Mousa U, Sav H, Köseoglulari O, Sahin A, Akcan N, Soytaç Inançli S, Bundak R. The Incidence and Demographic Distribution of Type 1 Diabetes Mellitus in Children Aged 16 or Younger Between 2000 and 2016 in Cyprus. J Clin Res Pediatr Endocrinol. 2020; 12(2): 175-179.

Muiesan ML, Padovani A, Salvetti M, Monteduro C, Poisa P, Bonzi B, Paini A, Cottini E, Agosti C, Castellano M, Rizzoni D, Vignolo A, Agabiti-Rosei E. Headache: Prevalence and relationship with office or ambulatory blood pressure in a general population sample (the Vobarno Study). Blood Press. 2006; 15(1): 14-9.

Muiña PG, Herrera MJ, Atance EP, Donado JJ, Sánchez G, Ferrer LS. [Epidemiological study of type 1 diabetes in children under 15 years-old in Castilla-La Mancha (Spain)]. An Pediatr (Barc). 2012; 76(2): 83-91.

Mulatinho LM, Ferraz APCR, Francisqueti FV, Garcia JL, de Souza CFQ, Cardoso MD, Lemes KCT, Mesquita MVCO, Corrente JE, Dos Santos KC, Corrêa CR. Prevalence of metabolic syndrome and associated factors in adults living in Fernando de Noronha, Brazil. Diabetes Metab Syndr. 2019; 13(1): 554-558.

Mulnier HE, Seaman HE, Raleigh VS, Soedamah-Muthu SS, Colhoun HM, Lawrenson RA. Mortality in people with type 2 diabetes in the UK. Diabet Med. 2006; 23(5): 516-21.

Muniz J, Hervada J, Juane R, Lopez-Rodriguez I, Castro-Beiras A. Prevalence of diabetes mellitus in the population aged 40-69 years in Galicia, northwest Spain. Diabetes Res Clin Pract. 1995; 30(2): 137-42.

Murdock D, Salit J, Stoffel M, Friedman JM, Pe’er I, Breslow JL, Bonnen PE. Longitudinal study shows increasing obesity and hyperglycemia in micronesia. Obesity (Silver Spring). 2013; 21(9): E421–7.

Muyer MT, Muls E, Mapatano MA, Makulo JR, Mvitu M, Kimenyembo W, Mandja BA, Kimbondo P, Bieleli CB, Kaimbo Wa Kaimbo D, Buntinx F. Diabetes and intermediate hyperglycaemia in Kisantu, DR Congo: a cross-sectional prevalence study. BMJ Open. 2012; 2(6): nan.

Mykkänen L, Laakso M, Uusitupa M, Pyörälä K. Prevalence of diabetes and impaired glucose tolerance in elderly subjects and their association with obesity and family history of diabetes. Diabetes Care. 1990; 13(11): 1099-105.

Nabipour I, Amiri M, Imami SR, Jahfari SM, Nosrati A, Iranpour D, Soltanian AR. Unhealthy lifestyles and ischaemic electrocardiographic abnormalities: the Persian Gulf Healthy Heart Study. East Mediterr Health J. 2008; 14(4): 858-68.

Nakagami T, Tominaga M, Nishimura R, Yoshiike N, Daimon M, Oizumi T, Tajima N. Is the measurement of glycated hemoglobin A1c alone an efficient screening test for undiagnosed diabetes? Japan National Diabetes Survey. Diabetes Res Clin Pract. 2007; 76(2): 251-6.

Nangia V, Jonas JB, Sinha A, Matin A, Kulkarni M, Panda-Jonas S. Ocular axial length and its associations in an adult population of Central Rural India. The Central India Eye and Medical Study [Unpublished data]. Ophthalmology 2010;117(7):1360-6.

Napoli N, Mottini G, Arigliani M, Creta A, Giua R, Incammisa A, Carotti S, Sihom F, Yimagou I, Alombah R, Mbanya JC, Pozzilli P. Unexpectedly high rates of obesity and dysglycemia among villagers in Cameroon. Diabetes Metab Res Rev. 2010; 26(1): 10–12.

Natarajan H, Shanthi Rani CS, Krishna Kumar D, Anjana RM, Ranjit U, Venkatesan U, Uma Sankari G, Pradeepa R, Mohan V, Deepa M. Future risk of diabetes among Indians with metabolic and phenotypic obesity: Results from the 10-year follow-up of the Chennai Urban Rural Epidemiology Study (CURES-158). Acta Diabetol. 2021; 58(8): 1051-1058.

NatCen Social Research and Royal Free and University College Medical School. Department of Epidemiology and Public Health, Health Survey for England, 2010 [computer file]. 2nd Edition. Colchester, Essex: UK Data Archive [distributor], July 2012. SN: 6986, http://dx.doi.org/10.5255/UKDA-SN-6986-2

NatCen Social Research and University College London. Department of Epidemiology and Public Health, Health Survey for England, 2011 [computer file]. Colchester, Essex: UK Data Archive [distributor], April 2013. SN: 7260, http://dx.doi.org/10.5255/UKDA-SN-7260-1

NatCen Social Research and University College London. Department of Epidemiology and Public Health, Health Survey for England, 2012 [computer file]. Colchester, Essex: UK Data Archive [distributor], April 2014. SN: 7480, http://dx.doi.org/10.5255/UKDA-SN-7480-1

NatCen Social Research and University College London. Department of Epidemiology and Public Health, Health Survey for England, 2013 [computer file]. Colchester, Essex: UK Data Archive [distributor], January 2015. SN: 7649, http://dx.doi.org/10.5255/UKDA-SN-7649-1

NatCen Social Research, University College London. Department of Epidemiology and Public Health. (2016). Health Survey for England, 2014. [data collection]. UK Data Service. SN: 7919, http://dx.doi.org/10.5255/UKDA-SN-7919-1

NatCen Social Research, University College London. Department of Epidemiology and Public Health. (2017). Health Survey for England, 2015. [data collection]. UK Data Service. SN: 8280, http://doi.org/10.5255/UKDA-SN-8280-1

NatCen Social Research, University College London. Department of Epidemiology and Public Health. (2018). Health Survey for England, 2016. [data collection]. UK Data Service. SN: 8334, http://doi.org/10.5255/UKDA-SN-8334-1

National Center for Disease Control and Public Health (Georgia). Georgia STEPS Noncommunicable Disease Risk Factors Survey 2016.

National Center for Health Statistics (NCHS), Centers for Disease Control and Prevention (CDC). United States National Health and Nutrition Examination Survey 1976-1980. Hyattsville, United States: National Center for Health Statistics (NCHS), Centers for Disease Control and Prevention (CDC).

National Center for Health Statistics (NCHS), Centers for Disease Control and Prevention (CDC). United States National Health and Nutrition Examination Survey 1988-1994. Hyattsville, United States: National Center for Health Statistics (NCHS), Centers for Disease Control and Prevention (CDC).

National Center for Health Statistics (NCHS), Centers for Disease Control and Prevention (CDC). United States National Health and Nutrition Examination Survey 1999-2000. Hyattsville, United States: National Center for Health Statistics (NCHS), Centers for Disease Control and Prevention (CDC).

National Center for Health Statistics (NCHS), Centers for Disease Control and Prevention (CDC). United States National Health and Nutrition Examination Survey 2001-2002. Hyattsville, United States: National Center for Health Statistics (NCHS), Centers for Disease Control and Prevention (CDC).

National Center for Health Statistics (NCHS), Centers for Disease Control and Prevention (CDC). United States National Health and Nutrition Examination Survey 2003-2004. Hyattsville, United States: National Center for Health Statistics (NCHS), Centers for Disease Control and Prevention (CDC).

National Center for Health Statistics (NCHS), Centers for Disease Control and Prevention (CDC). United States National Health and Nutrition Examination Survey 2005-2006. Hyattsville, United States: National Center for Health Statistics (NCHS), Centers for Disease Control and Prevention (CDC), 2007.

National Center for Health Statistics (NCHS), Centers for Disease Control and Prevention (CDC). United States National Health and Nutrition Examination Survey 2007-2008. Hyattsville, United States: National Center for Health Statistics (NCHS), Centers for Disease Control and Prevention (CDC), 2009.

National Center for Health Statistics (NCHS), Centers for Disease Control and Prevention (CDC). United States National Health and Nutrition Examination Survey 2009-2010. Hyattsville, United States: National Center for Health Statistics (NCHS), Centers for Disease Control and Prevention (CDC), 2011.

National Center for Health Statistics, Centers for Disease Control and Prevention. United States National Health and Nutrition Examination Survey 2011-2012. Hyattsville, United States of America: National Center for Health Statistics, Centers for Disease Control and Prevention, 2013.

National Center for Health Statistics, Centers for Disease Control and Prevention. United States National Health and Nutrition Examination Survey 2013-2014. Hyattsville, United States of America: National Center for Health Statistics, Centers for Disease Control and Prevention.

National Center for Health Statistics, Centers for Disease Control and Prevention. United States National Health and Nutrition Examination Survey 2015-2016. Hyattsville, United States of America: National Center for Health Statistics, Centers for Disease Control and Prevention, 2017.

National Center for Health Statistics, Centers for Disease Control and Prevention. United States National Health and Nutrition Examination Survey Pre-Pandemic 2017-2020. Hyattsville, United States of America: National Center for Health Statistics, Centers for Disease Control and Prevention, 2022.

National Centre for Social Research and University College London. Department of Epidemiology and Public Health, Health Survey for England, 2003 [computer file]. Colchester, Essex: UK Data Archive [distributor], March 2005. SN: 5098.

National Centre for Social Research and University College London. Department of Epidemiology and Public Health, Health Survey for England, 2006 [computer file]. 4th Edition. Colchester, Essex: UK Data Archive [distributor], July 2011. SN: 5809, http://dx.doi.org/10.5255/UKDA-SN-5809-1

National Centre for Social Research and University College London. Department of Epidemiology and Public Health, Health Survey for England, 2008 [computer file]. 3rd Edition. Colchester, Essex: UK Data Archive [distributor], July 2011. SN: 6397, http://dx.doi.org/10.5255/UKDA-SN-6397-1

National Centre for Social Research and University College London. Department of Epidemiology and Public Health, Health Survey for England, 2009 [computer file]. 2nd Edition. Colchester, Essex: UK Data Archive [distributor], July 2011. SN: 6732, http://dx.doi.org/10.5255/UKDA-SN-6732-1

National Centre for Social Research and University College London. Department of Epidemiology and Public Health, Health Survey for England, 2005 [computer file]. Colchester, Essex: UK Data Archive [distributor], July 2007. SN: 5675.

National Health Examination Survey Office (Thailand). Thailand National Health and Examination Survey 2008-2009.

National Health Fund (Poland). Poland National Health Fund Patient Claims 2015.

National Health Fund (Poland). Poland National Health Fund Patient Claims 2016.

National Health Fund (Poland). Poland National Health Fund Patient Claims 2017.

National Health Fund (Poland). Poland National Health Fund Patient Claims 2018.

National Health Fund (Poland). Poland National Health Fund Patient Claims 2019.

National Institute for Medical Research (Tanzania), World Health Organization (WHO). Tanzania STEPS Noncommunicable Disease Risk Factors Survey 2012.

National Institute of Hygiene, Epidemiology and Microbiology (Cuba), National Office of Statistics and Information (Cuba). Cuba National Survey of Risk Factors and Preventive Activities for Non-transmissible Diseases 2010-2011.

National Institute of Public Health (Mexico), National Institute of Statistics and Geography (INEGI) (Mexico). Mexico National Survey of Health and Nutrition (ENSANUT) 2018-2019. Cuernavaca, Mexico: National Institute of Public Health (Mexico).

National Institute of Public Health (Mexico). Mexico National Health Survey 1999-2000.

National Institute of Public Health (Mexico). Mexico National Survey of Health and Nutrition 2005-2006. Cuernavaca, Mexico: National Institute of Public Health (Mexico).

National Institute of Public Health (Mexico). Mexico National Survey of Health and Nutrition 2011-2012. Cuernavaca, Mexico: National Institute of Public Health (Mexico).

National Research Council (Italy). Italian Longitudinal Study on Ageing (ILSA) 1992-1993.

Negrato CA, Dias JPL, Teixeira MF, Dias A, Salgado MH, Lauris JR, Montenegro RM Jr, Gomes MB, Jovanovic L. Temporal trends in incidence of Type 1 diabetes between 1986 and 2006 in Brazil. J Endocrinol Invest. 2010; 33(6): 373-7.

Newhook LA, Penney S, Fiander J, Dowden J. Recent incidence of type 1 diabetes mellitus in children 0-14 years in Newfoundland and Labrador, Canada climbs to over 45/100,000: a retrospective time trend study. BMC Res Notes. 2012; 628.

NHS England, Scottish Diabetes Data Group. United Kingdom - Scottish Diabetes Survey 2010. Scotland: Diabetes in Scotland.

NHS England, Scottish Diabetes Data Group. United Kingdom - Scottish Diabetes Survey 2011. Scotland: Diabetes in Scotland.

NHS England, Scottish Diabetes Data Group. United Kingdom - Scottish Diabetes Survey 2012. Scotland: Diabetes in Scotland.

NHS England, Scottish Diabetes Data Group. United Kingdom - Scottish Diabetes Survey 2013. Scotland: Diabetes in Scotland.

NHS England, Scottish Diabetes Data Group. United Kingdom - Scottish Diabetes Survey 2014. Scotland: Diabetes in Scotland.

NHS England, Scottish Diabetes Data Group. United Kingdom - Scottish Diabetes Survey 2015. Scotland: Diabetes in Scotland, 2016.

NHS England, Scottish Diabetes Data Group. United Kingdom - Scottish Diabetes Survey 2016. Scotland: Diabetes in Scotland.

NHS England, Scottish Diabetes Data Group. United Kingdom - Scottish Diabetes Survey 2017. Scotland: Diabetes in Scotland, 2018.

NHS England, Scottish Diabetes Data Group. United Kingdom - Scottish Diabetes Survey 2018. Scotland: Diabetes in Scotland, 2019.

NHS England, Scottish Diabetes Data Group. United Kingdom - Scottish Diabetes Survey 2019. Scotland: Diabetes in Scotland, 2020.

NHS England, Scottish Diabetes Data Group. United Kingdom - Scottish Diabetes Survey 2020. Scotland: Diabetes in Scotland, 2021.

Nilsson PM, Johansson SE, Sundquist J. Low educational status is a risk factor for mortality among diabetic people. Diabet Med. 1998; 15(3): 213-9.

Nilsson PM, Møller L, Solstad K. Adverse effects of psychosocial stress on gonadal function and insulin levels in middle-aged males. J Intern Med. 1995; 237(5): 479-86.

Niskanen L, Turpeinen A, Penttilä I, Uusitupa MI. Hyperglycemia and compositional lipoprotein abnormalities as predictors of cardiovascular mortality in type 2 diabetes: a 15-year follow-up from the time of diagnosis. Diabetes Care. 1998; 21(11): 1861-9.

Norwegian Institute of Public Health, University of Bergen, University of Oslo, University of Tromsø - The Arctic University of Norway, University of Trondheim (Norway). Cohort of Norway 1994-2003.

Norwegian University of Science and Technology (NTNU). Norway - Nord-Trøndelag Health Study Wave 3 2006-2008. Trondheim, Norway: Norwegian University of Science and Technology (NTNU).

Nóvoa Y, de La Cuesta A, Caballero E, de Gauna MR, Quinteiro S, Domínguez A, Santana A, Wägner AM. Epidemiology of childhood-onset type 1 diabetes in Gran Canaria (2006-2018). Endocrinol Diabetes Nutr (Engl Ed). 2020; 67(10): 658-664.

Nyenwe EA, Odia OJ, Ihekwaba AE, Ojule A, Babatunde S. Type 2 diabetes in adult Nigerians: a study of its prevalence and risk factors in Port Harcourt, Nigeria. Diabetes Res Clin Pract. 2003; 62(3): 177-85.

O Connor JM, Millar SR, Buckley CM, Kearney PM, Perry IJ. The prevalence and determinants of undiagnosed and diagnosed type 2 diabetes in middle-aged irish adults. PLoS One. 2013; 8(11): e80504.

Oba S, Nagata C, Nakamura K, Takatsuka N, Shimizu H. Self-reported Diabetes Mellitus and Risk of Mortality from All Causes, Cardiovascular Disease, and Cancer in Takayama: A Population-based Prospective Cohort Study in Japan. J Epidemiol. 2008; 18(5): 197–203.

Office of Population Censuses and Surveys. Social Survey Division, Health Survey for England, 1993 [Computer file]. Colchester, Essex: UK Data Archive [distributor], April 1995. SN: 3316, http://dx.doi.org/10.5255/UKDA-SN-3316-1

Ohmura T, Ueda K, Kiyohara Y, Kato I, Iwamoto H, Nakayama K, Nomiyama K, Ohmori S, Yoshitake T, Shinkawu A. Prevalence of type 2 (non-insulin-dependent) diabetes mellitus and impaired glucose tolerance in the Japanese general population: the Hisayama Study. Diabetologia. 1993; 36(11): 1198-203.

Okada K, Furusyo N, Sawayama Y, Kanamoto Y, Murata M, Hayashi J. Prevalence and risk factors for diabetes: a ten year follow-up study of the Yaeyama district of Okinawa. Hukuoka Acta Med. 2010; 101(10): 215-24.

Okesina AB, Oparinde DP, Akindoyin KA, Erasmus RT. Prevalence of some risk factors of coronary heart disease in a rural Nigerian population. East Afr Med J. 1999; 76(4): 212-6.

Oladapo OO, Salako L, Sodiq O, Shoyinka K, Adedapo K, Falase AO. A prevalence of cardiometabolic risk factors among a rural Yoruba south-western Nigerian population: a population-based survey. Cardiovasc J Afr. 2010; 21(1): 26–31.

Omar MA, Seedat MA, Motala AA, Dyer RB, Becker P. The prevalence of diabetes mellitus and impaired glucose tolerance in a group of urban South African blacks. S Afr Med J. 1993; 83(9): 641-3.

Omboni S, Carabelli G, Ghirardi E, Carugo S. Awareness, treatment, and control of major cardiovascular risk factors in a small-scale Italian community: results of a screening campaign. Vasc Health Risk Manag. 2013; 9: 177–85.

Onat A, Hergenc G, Bulur S, Ugur M, Kucukdurmaz Z, Can G. The paradox of high apolipoprotein A-I levels independently predicting incident type-2 diabetes among Turks. Int J Cardiol. 2010; 142(1): 72–9.

Ongosi AN, Wilunda C, Musumari PM, Techasrivichien T, Wang CW, Ono-Kihara M, Serrem C, Kihara M, Nakayama T. Prevalence and Risk Factors of Elevated Blood Pressure and Elevated Blood Glucose among Residents of Kajiado County, Kenya: A Population-Based Cross-Sectional Survey. Int J Environ Res Public Health. 2020; 17(19).

Ono K, Limbu YR, Rai SK, Kurokawa M, Yanagida J, Rai G, Gurung N, Sharma M, Rai CK. The prevalence of type 2 diabetes mellitus and impaired fasting glucose in semi-urban population of Nepal. Nepal Med Coll J. 2007; 9(3): 154-6.

ONROCAD Study Group. Romanian Childhood Diabetes Registry 2016.

ONROCAD Study Group. Romanian Childhood Diabetes Registry 2017.

ONROCAD Study Group. Romanian Childhood Diabetes Registry 2018.

ONROCAD Study Group. Romanian Childhood Diabetes Registry 2019.

ONROCAD Study Group. Romanian Childhood Diabetes Registry 2020.

ONROCAD Study Group. Romanian Childhood Diabetes Registry 2021.

ONROCAD Study Group. Romanian Childhood Diabetes Registry 2022.

Ostovaneh MR, Zamani F, Sharafkhah M, Ansari-Moghaddam A, Akhavan Khaleghi N, Saeedian FS, Rohani Z, Motamed N, Maadi M, Malekzadeh R, Poustchi H. Prevalence of metabolic syndrome in Amol and Zahedan, Iran: a population based study. Arch Iran Med. 2014; 17(7): 477–82.

Owoaje EE, Rotimi CN, Kaufman JS, Tracy J, Cooper RS. Prevalence of adult diabetes in Ibadan, Nigeria. East Afr Med J. 1997; 74(5): 299-302.

Özalkak S, Yildirim R, Tunç S, Ünal E, Tas FF, Demirbilek H, Özbek MN. Revisiting the Annual Incidence of Type 1 Diabetes Mellitus in Children from the Southeastern Anatolian Region of Turkey: A Regional Report. J Clin Res Pediatr Endocrinol. 2022; 14(2): 172-178.

Palomo G I, Icaza N G, Mujica E V, Núñez F L, Leiva M E, Vásquez R M, Alarcón L M, Moyano D E. Prevalencia de factores de riesgo cardiovascular clásicos en población adulta de Talca, Chile, 2005. Rev Med Chil. 2007; 135(7): 904-12.

Pan American Health Organization (PAHO). Central America Diabetes Initiative (CAMDI): Survey of Diabetes, Hypertension and Chronic Disease Risk Factors. Belize, San José, San Salvador, Guatemala City, Managua and Tegucigalpa. Washington, D.C., United States: Pan American Health Organization (PAHO), 2011.

Pan American Health Organization (PAHO). Honduras - Tegucigalpa Diabetes, Hypertension, and Non-Communicable Disease Risk Factors Survey 2003-2004.

Pan W-H, Wu H-J, Yeh C-J, Chuang S-Y, Chang H-Y, Yeh N-H, Hsieh Y-T. Diet and health trends in Taiwan: comparison of two nutrition and health surveys from 1993-1996 and 2005-2008. Asia Pac J Clin Nutr. 2011; 20(2): 238–50.

Pan X, Yang W, Liu J. Prevalence of diabetes and its risk factors in China 1994. National Diabetes Prevention and Control Cooperative Group. Chin J Intern Med. 1997; 36(6): 384-9.

Papantoniou K, Fito M, Covas M-I, Munoz D, Schroder H. trans Fatty acid consumption, lifestyle and type 2 diabetes prevalence in a Spanish population. Eur J Nutr. 2010; 49(6): 357–64.

Papazoglou N, Manes C, Chatzimitrofanous P, Papadeli E, Tzounas K, Scaragas G, Kontogiannis I, Alexiades D. Epidemiology of diabetes mellitus in the elderly in northern Greece: a population study. Diabet Med. 1995; 12(5): 397-400.

Papoz L, Ben Khalifa F, Eschwege E, Ben Ayed H. Diabetes mellitus in Tunisia: description in urban and rural populations. Int J Epidemiol. 1988; 17(2): 419-22.

Park Y, Lee H, Koh CS, Min H, Yoo K, Kim Y, Shin Y. Prevalence of diabetes and IGT in Yonchon County, South Korea. Diabetes Care. 1995; 18(4): 545-8.

Parviainen A, But A, Siljander H, Knip M, Finnish Pediatric Diabetes Register. Decreased Incidence of Type 1 Diabetes in Young Finnish Children. Diabetes Care. 2020; 43(12): 2953-2958.

Passos VM de A, Barreto SM, Diniz LM, Lima-Costa MF. Type 2 diabetes: prevalence and associated factors in a Brazilian community--the Bambuí health and aging study. Sao Paulo Med J. 2005; 123(2): 66-71.

Patterson CC, Smith PG, Webb J, Heasman MA, Mann JI. Geographical variation in the incidence of diabetes mellitus in Scottish children during the period 1977-1983. Diabet Med. 1988; 5(2): 160-5.

Pedro JM, Brito M, Barros H. Prevalence, awareness, treatment and control of hypertension, diabetes and hypercholesterolaemia among adults in Dande municipality, Angola. Cardiovasc J Afr. 2017; 28: 1–10.

Pemminati S, Prabha Adhikari MR, Pathak R, Pai MRSM. Prevalence of metabolic syndrome (METS) using IDF 2005 guidelines in a semi urban south Indian (Boloor Diabetes Study) population of Mangalore. J Assoc Physicians India. 2010; 58: 674–7.

Pérez CM, Guzmán M, Ortiz AP, Estrella M, Valle Y, Pérez N, Haddock L, Suárez E. Prevalence of the metabolic syndrome in San Juan, Puerto Rico. Ethn Dis. 2008; 18(4): 434-41.

Perkkiö Y, Jokelainen J, Auvinen J, Eskola P, Saltevo J, Keinänen-Kiukaanniemi S, Timonen M. Glucose status and depressive symptoms: a cohort study of elderly people in northwest Finland. Scand J Prim Health Care. 2019; 37(2): 242-248.

Pessinaba S, Mbaye A, Yabeta G-A-D, Kane A, Ndao CT, Ndiaye MB, Harouna H, Bodian M, Diao M, Mbaye MN, Diagne MN, Diack B, Kane M, Niang K, Mathieu J-BS, Kane A. Prevalence and determinants of hypertension and associated cardiovascular risk factors: data from a population-based, cross-sectional survey in Saint Louis, Senegal. Cardiovasc J Afr. 2013; 24(5): 180–3.

Petry N, Rohner F, Phall MC, Jallow B, Ceesay AA, Sawo Y, Darboe MK, Barrow S, Sarr A, Ceesay PO, Fofana MN, Prentice AM, Wegmüller R, Wirth JP. Prevalence and co-existence of cardiometabolic risk factors and associations with nutrition-related and socioeconomic indicators in a national sample of Gambian women. Sci Rep. 2021; 11(1): 12057.

Piffaretti C, Mandereau-Bruno L, Guilmin-Crepon S, Choleau C, Coutant R, Fosse-Edorh S. Trends in childhood type 1 diabetes incidence in France, 2010-2015. Diabetes Res Clin Pract. 2019; 149: 200-207.

Pinelli L, Beretta F, Dalla Bernardina P, Gonfiantini E, Groff P. Incidence of insulin dependent diabetes mellitus in children 0-14 years old in the Veneto Region, Italy. J Pediatr Endocrinol Metab. 1998; 11(3): 447-50.

Pishdad GR. Low incidence of type 1 diabetes in Iran. Diabetes Care. 2005; 28(4): 927-8.

Podar T, Solntsev A, Karvonen M, Padaiga Z, Brigis G, Urbonaite B, Viik-Kajander M, Reunanen A, Tuomilehto J. Increasing incidence of childhood-onset type I diabetes in 3 Baltic countries and Finland 1983-1998. Diabetologia. 2001; B17-20.

Posadas-Romero C, Yamamoto-Kimura L, Lerman-Garber I, Zamora-Gonzalez J, Fajardo-Gutierrez A, Velazquez L, Cardoso-Saldaãna G. The Prevalence of NIDDM and Associated Coronary Risk Factors in Mexico City. Diabetes Care. 1994; 17(12): 1441-8.

Poyrazoglu S, Bundak R, Yavas Abali Z, Önal H, Sarikaya S, Akgün A, Bas S, Abali S, Bereket A, Eren E, Tarim Ö, Güven A, Yildiz M, Karaman Aksakal D, Yüksel A, Seymen Karabulut G, Hatun S, Özgen T, Cesur Y, Azizoglu M, Dilek E, Tütüncüler F, Papatya Çakir E, Özcabi B, Evliyaoglu O, Karadeniz S, Dursun F, Bolu S, Arslanoglu I, Yesiltepe Mutlu G, Kirmizibekmez H, Isgüven P, Üstyol A, Adal E, Uçar A, Cebeci N, Bezen D, Binay Ç, Semiz S, Korkmaz HA, Memioglu N, Sagsak E, Peltek HN, Yildiz M, Akçay T, Turan S, Güran T, Atay Z, Akcan N, Çizmecioglu F, Ercan O, Dagdeviren A, Bas F, Issever H, Darendeliler F. Incidence of Type 1 Diabetes in Children Aged Below 18 Years during 2013-2015 in Northwest Turkey. J Clin Res Pediatr Endocrinol. 2018; 10(4): 336-342.

Preis SR, Hwang SJ, Coady S, Pencina MJ, D'Agostino RB Sr, Savage PJ, Levy D, Fox CS. Trends in all-cause and cardiovascular disease mortality among women and men with and without diabetes mellitus in the Framingham Heart Study, 1950 to 2005. Circulation. 2009; 119(13): 1728-35.

Pronina EA, Petraikina EE, Antsiferov MB, Duchareva OV, Petrone A, Buzzetti R, Pozzilli P. A 10-year (1996-2005) prospective study of the incidence of Type 1 diabetes in Moscow in the age group 0-14 years. Diabet Med. 2008; 25(8): 956-9.

Public Health Institute, Ministry of Health (Mongolia), World Health Organization (WHO). Mongolia STEPS Noncommunicable Disease Risk Factors Survey 2013.

Public Health Institute, Ministry of Health (Mongolia), World Health Organization (WHO). Mongolia STEPS Noncommunicable Disease Risk Factors Survey 2019. Geneva, Switzerland: World Health Organization (WHO).

Pubudu De Silva A, Padmal De Silva SH, Liyanage IK, Rajapakse LC, Jayasinghe KSA, Katulanda P, Wijeratne CN, Wijeratne S. Social, cultural and economical determinants of diabetes mellitus in Kalutara district, Sri Lanka: a cross sectional descriptive study. Int J Equity Health. 2012; 11: 76.

Qatar Statistics Authority, Supreme Council of Health (Qatar), World Health Organization (WHO). Qatar STEPS Noncommunicable Disease Risk Factors Survey 2012. Geneva, Switzerland: World Health Organization (WHO).

Qi L, Feng L, Ding X, Mao D, Wang Y, Xiong H. Prevalence of diabetes and impaired fasting glucose among residents in the Three Gorges Reservoir Region, China. BMC Public Health. 2014; 14: 1152.

Qin Y, Wang R, Ma X, Zhao Y, Lu J, Wu C, He J. Prevalence, Awareness, Treatment and Control of Diabetes Mellitus-A Population Based Study in Shanghai, China. Int J Environ Res Public Health. 2016; 13(5).

Quan HL, Blizzard CL, Venn AJ, Thuy AB, Luc PH, Sharman JE. Blood pressure and body mass index: a comparison of the associations in the Caucasian and Asian populations. Hypertens Res. 2012; 35(5): 523-30.

Quoc PS, Charles MA, Cuong NH, Lieu LH, Tuan NA, Thomas M, Balkau B, Simon D. Blood glucose distribution and prevalence of diabetes in Hanoi (Vietnam). Am J Epidemiol. 1994; 139(7): 713-22.

Radosevic B, Bukara-Radujkovic G, Miljkovic V, Pejicic S, Bratina N, Battelino T. The incidence of type 1 diabetes in Republic of Srpska (Bosnia and Herzegovina) and Slovenia in the period 1998-2010. Pediatr Diabetes. 2013; 14(4): 273-9.

Rafferty J, Stephens JW, Atkinson MD, Luzio SD, Akbari A, Gregory JW, Bain S, Owens DR, Thomas RL. A retrospective epidemiological study of type 1 diabetes mellitus in wales, UK between 2008 and 2018. Int J Popul Data Sci. 2021; 6(1): 1387.

Rahim MA, Hussain A, Azad Khan AK, Sayeed MA, Keramat Ali SM, Vaaler S. Rising prevalence of type 2 diabetes in rural Bangladesh: a population based study. Diabetes Res Clin Pract. 2007; 77(2): 300-5.

Rahman MM, Rahim MA, Nahar Q. Prevalence and risk factors of type 2 diabetes in an urbanizing rural community of Bangladesh. Bangladesh Med Res Counc Bull. 2007; 33(2): 48-54.

Rahmani F, Asgari S, Khalili D, Habibi Moeini AS, Tohidi M, Azizi F, Hadaegh F. National trends in cardiovascular health metrics among Iranian adults using results of three cross-sectional STEPwise approaches to surveillance surveys. Sci Rep. 2021; 11(1): 58.

Raiko JRH, Viikari JSA, Ilmanen A, Hutri-Kahonen N, Taittonen L, Jokinen E, Pietikainen M, Jula A, Loo B-M, Marniemi J, Lehtimaki T, Kahonen M, Ronnemaa T, Raitakari OT, Juonala M. Follow-ups of the Cardiovascular Risk in Young Finns Study in 2001 and 2007: levels and 6-year changes in risk factors. J Intern Med. 2010; 267(4): 370–84.

Ramachandran A, Snehalatha C, Krishnaswamy CV. Incidence of IDDM in children in urban population in southern India. Madras IDDM Registry Group Madras, South India. Diabetes Res Clin Pract. 1996; 34(2): 79–82.

Raman Kutty V, Joseph A, Soman CR. High prevalence of type 2 diabetes in an urban settlement in Kerala, India. Ethn Health. 1999; 4(4): 231-9.

Rampal S, Rampal L, Rahmat R, Zain AM, Yap YG, Mohamed M, Taha M. Variation in the prevalence, awareness, and control of diabetes in a multiethnic population: a nationwide population study in Malaysia. Asia Pac J Public Health. 2010; 22(2): 194-202.

Rangasami JJ, Greenwood DC, McSporran B, Smail PJ, Patterson CC, Waugh NR. Rising incidence of type 1 diabetes in Scottish children, 1984-93. The Scottish Study Group for the Care of Young Diabetics. Arch Dis Child. 1997; 77(3): 210-3.

Rathmann W, Haastert B, Icks A, Löwel H, Meisinger C, Holle R, Giani G. High prevalence of undiagnosed diabetes mellitus in Southern Germany: target populations for efficient screening. The KORA survey 2000. Diabetologia. 2003; 46(2): 182-9.

Rawal I, Ghosh S, Hameed SS, Shivashankar R, Ajay VS, Patel SA, Goodman M, Ali MK, Narayan KMV, Tandon N, Prabhakaran D. Association between poor oral health and diabetes among Indian adult population: potential for integration with NCDs. BMC Oral Health. 2019; 19(1): 191.

Republican Scientific and Practical Center of Medical Technologies, Informatization, Management and Economics of Public Health (Belarus), World Health Organization (WHO). Belarus STEPS Noncommunicable Disease Risk Factors Survey 2016-2017.

Rigo Carratala F, Frontera Juan G, Canaves Llobera J, Rodriguez Ruiz T, Borras Bosch T, Fuentespina Vidal E, . Prevalencia de factores de riesgo cardiovascular en IslasBaleares (estudio CORSAIB). Rev Esp Cardiol. 2005; 58(12): 1411-9.

Rigo JC, Vieira JL, Dalacorte RR, Reichert CL. Prevalence of metabolic syndrome in an elderly community: comparison between three diagnostic methods. Arq Bras Cardiol. 2009; 93(2): 85-91.

Roche EF, McKenna AM, Ryder KJ, Brennan AA, O’Regan M, Hoey HM. Is the incidence of type 1 diabetes in children and adolescents stabilising? The first 6 years of a National Register. Eur J Pediatr. 2016; 175(12): 1913–9.

Roche EF, Menon A, Gill D, Hoey HM. Incidence of type 1 diabetes mellitis in children aged under 15 years in the Republic of Ireland. J Pediatr Endocrinol Metab. 2002; 15(8): 1191-4.

Roche MM, Wang PP. Sex differences in all-cause and cardiovascular mortality, hospitalization for individuals with and without diabetes, and patients with diabetes diagnosed early and late. Diabetes Care. 2013; 36(9): 2582-90.

Rodrigues Junior W, Gaban SCN, Pontes ERJC, Souza CC, Gimenes LP, Lacerda PF, Cunha MLMN, Stefanello II JVL, Brum LM, Oliveira LA, Silva CR, Ribeiro ALD. Diabetes mellitus and impaired glucose tolerance in urban adult population. Rev Assoc Med Bras. 2014; 60(2): 118-24.

Rogers MAM, Kim C, Banerjee T, Lee JM. Fluctuations in the incidence of type 1 diabetes in the United States from 2001 to 2015: a longitudinal study. BMC Med. 2017; 15(1): 199.

Roglic G, Pavlic-Renar I, Sestan-Crnek S, Prasek M, Kadrnka-Lovrencic M, Radica A, Metelko Z. Incidence of IDDM during 1988-1992 in Zagreb, Croatia. Diabetologia. 1995; 38(5): 550-4.

Rojnic Putarek N, Ille J, Spehar Uroic A, Skrabic V, Stipancic G, Krnic N, Radica A, Marjanac I, Severinski S, Svigir A, Bogdanic A, Dumic M. Incidence of type 1 diabetes mellitus in 0 to 14-yr-old children in Croatia - 2004 to 2012 study. Pediatr Diabetes. 2015; 16(6): 448–53.

Rojo-Martínez G, Valdés S, Soriguer F, Vendrell J, Urrutia I, Pérez V, Ortega E, Ocón P, Montanya E, Menéndez E, Lago-Sampedro A, González-Frutos T, Gomis R, Goday A, García-Serrano S, García-Escobar E, Galán-García JL, Castell C, Badía-Guillén R, Aguilera-Venegas G, Girbés J, Gaztambide S, Franch-Nadal J, Delgado E, Chaves FJ, Castaño L, Calle-Pascual A. Incidence of diabetes mellitus in Spain as results of the nation-wide cohort dibet.es study. Sci Rep. 2020; 10(1): 2765.

Rønn PF, Jørgensen ME, Smith LS, Bjerregaard P, Dahl-Petersen IK, Larsen CVL, Grarup N, Andersen GS. Associations between birth weight and glucose intolerance in adulthood among Greenlandic Inuit. Diabetes Res Clin Pract. 2019; 150: 129-137.

Roper NA, Bilous RW, Kelly WF, Unwin NC, Connolly VM. Excess mortality in a population with diabetes and the impact of material deprivation: longitudinal, population based study. BMJ. 2001; 322(7299): 1389-93.

Rosado Martín J, Martínez López MÁ, Mantilla Morató T, Dujovne Kohan I, Palau Cuevas FJ, Torres Jiménez R, García Puig J. [Prevalence of diabetes in an adult population in the region of Madrid (Spain). The Madrid Cardiovascular Risk study]. Gac Sanit. 2012; 26(3): 243-50.

Rosenbauer J, Herzig P, von Kries R, Neu A, Giani G. Temporal, seasonal, and geographical incidence patterns of type I diabetes mellitus in children under 5 years of age in Germany. Diabetologia. 1999; 42(9): 1055-9.

Rosero-Bixby, Luis , Xinia Fernández, and William H. Dow. CRELES: Costa Rican Longevity and Healthy Aging Study, 2005 (Costa Rica Estudio de Longevidad y Envejecimiento Saludable) [Computer file]. ICPSR26681-v1. Ann Arbor, MI: Inter-university Consortium for Political and Social Research [distributor], 2010-07-21. doi:10.3886/ICPSR26681

Rosmond R, Björntorp P. Blood pressure in relation to obesity, insulin and the hypothalamic-pituitary-adrenal axis in Swedish men. J Hypertens. 1998; 16(12): 1721-6.

Rosso D, Campagna S, Di Stefano F, Romano G, Maugeri D, Maggi S, Motta M, Catanzaro S, Carnazzo G. Prevalence of diabetes mellitus in a sample of the elderly population of the city of Catania. Arch Gerontol Geriatr. 1998; 27(3): 223-35.

Rouvre M, Vol S, Gusto G, Born C, Lantieri O, Tichet J, Lecomte P. Low high density lipoprotein cholesterol: prevalence and associated risk-factors in a large French population. Ann Epidemiol. 2011; 21(2): 118–27.

Saad FM, Mahmoud LA, Ali AMM, Omer IM, Abdullah MA. Incidence and prevalence of type 1 diabetes mellitus in children and adolescents aged 6 months-19 years in Khartoum State, Sudan. Sudan J Paediatr. 2020; 20(2): 163-169.

Saadi H, Carruthers SG, Nagelkerke N, Al-Maskari F, Afandi B, Reed R, Lukic M, Nicholls MG, Kazam E, Algawi K, Al-Kaabi J, Leduc C, Sabri S, El-Sadig M, Elkhumaidi S, Agarwal M, Benedict S. Prevalence of diabetes mellitus and its complications in a population-based sample in Al Ain, United Arab Emirates. Diabetes Res Clin Pract. 2007; 78(3): 369-77.

Sabir A, Ohwovoriole A, Isezuo S, Fasanmade O, Abubakar S, Iwuala S. Type 2 diabetes mellitus and its risk factors among the rural Fulanis of Northern Nigeria. Ann Afr Med. 2013; 12(4): 217-22.

Sadeghi M, Roohafza H, Shirani S, Poormoghadas M, Kelishadi R, Baghaii A, Sarraf-Zadegan N. Diabetes and associated cardiovascular risk factors in Iran: the Isfahan Healthy Heart Programme. Ann Acad Med Singapore. 2007; 36(3): 175-80.

Sadikot SM, Nigam A, Das S, Bajaj S, Zargar AH, Prasannakumar KM, Sosale A, Munichoodappa C, Seshiah V, Singh SK, Jamal A, Sai K, Sadasivrao Y, Murthy SS, Hazra DK, Jain S, Mukherjee S, Bandyopadhay S, Sinha NK, Mishra R, Dora M, Jena B, Patra P, Goenka K, DiabetesIndia. The burden of diabetes and impaired fasting glucose in India using the ADA 1997 criteria: prevalence of diabetes in India study (PODIS). Diabetes Res Clin Pract. 2004; 66(3): 293-300.

Saeed KMI, Rasooly MH, Nejaby M. Profile of risk factors for noncommunicable diseases in major cities of Afghanistan: WHO STEPwise approach. East Mediterr Health J. 2020; 26(4): 388-399.

Saito T, Kobayashi K, Kobayashi K, Mochizuki M, Yagasaki H, Makino K, Narusawa H, Watanabe D, Mitsui Y, Sato K, Sano T, Ohta M, Yokomichi H, Amemiya S. Incidence of childhood type 1 diabetes mellitus in Yamanashi Prefecture, Japan, 1986-2018. Endocrinol Diabetes Metab. 2021; 4(2): e00214.

Salazar MR, Carbajal HA, Espeche WG, Aizpurua M, Leiva Sisnieguez CE, March CE, Balbin E, Stavile RN, Reaven GM. Identifying cardiovascular disease risk and outcome: use of the plasma triglyceride/high-density lipoprotein cholesterol concentration ratio versus metabolic syndrome criteria. J Intern Med. 2013; 273(6): 595–601.

Salmi H, Heinonen S, Hästbacka J, Lääperi M, Rautiainen P, Miettinen PJ, Vapalahti O, Hepojoki J, Knip M. New-onset type 1 diabetes in Finnish children during the COVID-19 pandemic. Arch Dis Child. 2022; 107(2): 180-185.

Salti IS, Khogali M, Alam S, Haidar NA, Masri A. Epidemiology of diabetes mellitus in relation to other cardiovascular risk factors in Lebanon. East Mediterr Health J. 1997; 3(3): 462-71.

Samardzic M, Marinkovic J, Kocev N, Curovic N, Terzic N. Increasing incidence of childhood type 1 diabetes in Montenegro from 1997 to 2006. Pediatr Diabetes. 2010; 11(6): 412-6.

Sandy JL, Besançon S, Sidibé AT, Minkailou M, Togo A, Ogle GD. Rapid increases in observed incidence and prevalence of Type 1 diabetes in children and youth in Mali, 2007-2016. Pediatr Diabetes. 2021; 22(4): 545-551.

Sanisoglu SY, Oktenli C, Hasimi A, Yokusoglu M, Ugurlu M. Prevalence of metabolic syndrome-related disorders in a large adult population in Turkey. BMC Public Health. 2006; 92.

Santander Center for Public Health (Colombia), Santander Ministry of Health (Colombia), World Health Organization (WHO). Colombia - Santander STEPS Noncommunicable Disease Risk Factors Survey 2010.

Saquib N, Khanam MA, Saquib J, Anand S, Chertow GM, Barry M, Ahmed T, Cullen MR. High prevalence of type 2 diabetes among the urban middle class in Bangladesh. BMC Public Health. 2013; 1032.

Sasaki H, Kawasaki T, Ogaki T, Kobayashi S, Itoh K, Yoshimizu Y, Sharma S, Acharya GP. The prevalence of diabetes mellitus and impaired fasting glucose/glycaemia (IFG) in suburban and rural Nepal-the communities--based cross-sectional study during the democratic movements in 1990. Diabetes Res Clin Pract. 2005; 67(2): 167-74.

Sayeed MA, Hussain MZ, Banu A, Rumi MA, Azad Khan AK. Prevalence of diabetes in a suburban population of Bangladesh. Diabetes Res Clin Pract. 1997; 34(3): 149-55.

Sayeed MA, Mahtab H, Khanam PA, Latif ZA, Banu A, Khan AK. Prevalence of diabetes and impaired fasting glucose in urban population of Bangladesh. Bangladesh Med Res Counc Bull. 2007; 33(1): 1-12.

Schaan BD, Harzheim E, Gus I. [Cardiac risk profile in diabetes mellitus and impaired fasting glucose]. Rev Saude Publica. 2004; 38(4): 529-36.

Scheltens T, Bots ML, Numans ME, Grobbee DE, Hoes AW. Awareness, treatment and control of hypertension: the "rule of halves" in an era of risk-based treatment of hypertension. J Hum Hypertens. 2007; 21(2): 99-106.

Schöttker B, Raum E, Rothenbacher D, Müller H, Brenner H. Prognostic value of haemoglobin A1c and fasting plasma glucose for incident diabetes and implications for screening. Eur J Epidemiol. 2011; 26(10): 779–87.

Schranz AG. Abnormal glucose tolerance in the Maltese. A population-based longitudinal study of the natural history of NIDDM and IGT in Malta. Diabetes Res Clin Pract. 1989; 7(1): 7-16.

ScotCen Social Research and University College London. Department of Epidemiology and Public Health, Scottish Health Survey, 2010 [computer file]. Colchester, Essex: UK Data Archive [distributor], April 2012. SN: 6987, http://dx.doi.org/10.5255/UKDA-SN-6987-1

ScotCen Social Research, University College London. Department of Epidemiology and Public Health and University of Glasgow. MRC/CSO Social and Public Health Sciences Unit, Scottish Health Survey, 2011 [computer file]. 2nd Edition. Colchester, Essex: UK Data Archive [distributor], August 2013. SN: 7247, http://dx.doi.org/10.5255/UKDA-SN-7247-2

Scottish Centre for Social Research and University College London. Department of Epidemiology and Public Health, Scottish Health Survey, 2008 [computer file]. 2nd Edition. Colchester, Essex: UK Data Archive [distributor], April 2013. SN: 6383, http://dx.doi.org/10.5255/UKDA-SN-6383-2

Scottish Centre for Social Research and University College London. Department of Epidemiology and Public Health, Scottish Health Survey, 2009 [computer file]. 4th Edition. Colchester, Essex: UK Data Archive [distributor], November 2011. SN: 6713, http://dx.doi.org/10.5255/UKDA-SN-6713-2

Secretariat of Health and Environment (Libya), World Health Organization (WHO). Libya STEPS Noncommunicable Disease Risk Factors Survey 2009.

Seifu Y, Tsegaw D, Haji Y, Ejeso A. Prevalence and Associated Factors of Diabetes Mellitus Among Adult Population in Hawassa Zuria Woreda, Sidama Region, Ethiopia. Diabetes Metab Syndr Obes. 2020; 13: 4571-4579.

Sekikawa A, Tominaga M, Takahashi K, Eguchi H, Igarashi M, Ohnuma H, Sugiyama K, Manaka H, Sasaki H, Fukuyama H. Prevalence of diabetes and impaired glucose tolerance in Funagata area, Japan. Diabetes Care. 1993; 16(4): 570-4.

Sekita A, Arima H, Ninomiya T, Ohara T, Doi Y, Hirakawa Y, Fukuhara M, Hata J, Yonemoto K, Ga Y, Kitazono T, Kanba S, Kiyohara Y. Elevated depressive symptoms in metabolic syndrome in a general population of Japanese men: a cross-sectional study. BMC Public Health. 2013; 13: 862.

Serban V, Brink S, Timar B, Sima A, Vlad M, Timar R, Vlad A. An increasing incidence of type 1 diabetes mellitus in Romanian children aged 0 to 17 years. J Pediatr Endocrinol Metab. 2015; 28(3-4): 293-8.

Serban V, Timar R, Dabelea D, Green A, McKinney P, Law G. The epidemiology of childhood-onset type 1 diabetes mellitus in Romania. ONROCAD Study Group. National Romanian Organisation for the Care of Diabetic Children and Adolescents. J Pediatr Endocrinol Metab. 2001; 14(5): 535-41.

Seyed Reza SR, Bayani M, Zabihi A, Shakerian M, Habibian T, Bijani A. Undiagnosed hypertension and diabetes among the elderly in Amirkola, North of Iran. Caspian J Intern Med. 2019; 10(3): 303-308.

Shaltout AA, Moussa MAA, Qabazard M, Abdella N, Karvonen M, Al-Khawari M, Al-Arouj M, Al-Nakhi A, Tuomilehto J, El-Gammal A, Kuwait Diabetes Study Group. Further evidence for the rising incidence of childhood Type 1 diabetes in Kuwait. Diabet Med. 2002; 19(6): 522-5.

Shaltout AA, Wake D, Thanaraj TA, Omar DM, Al-AbdulRazzaq D, Channanath A, AlKandari H, Abdulrasoul M, Miller S, Conway N, Tuomilehto J, Davidsson L, on behalf of the Steering Group for the Study of Childhood Diabetes in Kuwait. Incidence of type 1 diabetes has doubled in Kuwaiti children 0-14 years over the last 20 years. Pediatr Diabetes. 2017; 18(8): 761–6.

Shan Y, Zhang Q, Liu Z, Hu X, Liu D. Prevalence and risk factors associated with chronic kidney disease in adults over 40 years: a population study from Central China. Nephrology (Carlton). 2010; 15(3): 354-61.

Sharma SK, Ghimire A, Radhakrishnan J, Thapa L, Shrestha NR, Paudel N, Gurung K, R M, Budathoki A, Baral N, Brodie D. Prevalence of hypertension, obesity, diabetes, and metabolic syndrome in Nepal. Int J Hypertens. 2011; 821971.

Shen SX, Wang HB, Chen ZW, Shen YE, Fu H, Wu CE, Ye TT, Wang JJ, Wang KA, Li TL, Yang Z, LaPorte RE, Dorman JS. The incidence of insulin-dependent diabetes mellitus in urban districts of Shanghai (1989-1993). J Pediatr Endocrinol Metab. 1996; 9(4): 469-73.

Sheng C-S, Liu M, Kang Y-Y, Wei F-F, Zhang L, Li G-L, Dong Q, Huang Q-F, Li Y, Wang J-G. Prevalence, awareness, treatment and control of hypertension in elderly Chinese. Hypertens Res. 2013; 36(9): 824–8.

Shera AS, Rafique G, Khawaja IA, Ara J, Baqai S, King H. Pakistan national diabetes survey: prevalence of glucose intolerance and associated factors in Shikarpur, Sindh Province. Diabet Med. 1995; 12(12): 1116-21.

Shera AS, Rafique G, Khawaja IA, Baqai S, King H. Pakistan National Diabetes Survey: prevalence of glucose intolerance and associated factors in Baluchistan province. Diabetes Res Clin Pract. 1999; 44(1): 49-58.

Shi G, Zhu N, Qiu L, Yan H, Zeng L, Wang D, Dang S, Li Z, Kang Y, Chen T, Li C. Impact of the 2020 China Diabetes Society Guideline on the Prevalence of Diabetes Mellitus and Eligibility for Antidiabetic Treatment in China. Int J Gen Med. 2021; 14: 6639-6645.

Shih WL, Tung YC, Chang LY, Fang CT, Tsai WY. Increased Incidence of Pediatric Type 1 Diabetes With Novel Association With Coxsackievirus A Species in Young Children but Declined Incidence in Adolescents in Taiwan. Diabetes Care. 2021; 44(7): 1579-1585.

Shrestha UK, Singh DL, Bhattarai MD. The prevalence of hypertension and diabetes defined by fasting and 2-h plasma glucose criteria in urban Nepal. Diabet Med. 2006; 23(10): 1130-5.

Silva H, Hernandez-Hernandez R, Vinueza R, Velasco M, Boissonnet CP, Escobedo J, Silva HE, Pramparo P, Wilson E, CARMELA Study Investigators. Cardiovascular risk awareness, treatment, and control in urban Latin America. Am J Ther. 2010; 17(2): 159–66.

Silva-Matos C, Gomes A, Azevedo A, Damasceno A, Prista A, Lunet N. Diabetes in Mozambique: prevalence, management and healthcare challenges. Diabetes Metab. 2011; 37(3): 237-44.

Simmons D, McKenzie A, Eaton S, Shaw J, Zimmet P. Prevalence of diabetes in rural Victoria. Diabetes Res Clin Pract. 2005; 70(3): 287-90.

Simons LA, McCallum J, Friedlander Y, Simons J. Diabetes, mortality and coronary heart disease in the prospective Dubbo study of Australian elderly. Aust N Z J Med. 1996; 26(1): 66-74.

Singh A, Milton PE, Nanaiah A, Samuel P, Thomas N. Awareness and attitude toward diabetes in the rural population of Arunachal Pradesh, Northeast India. Indian J Endocrinol Metab. 2012; 16(Suppl 1): S83-6.

Singh DL, Bhattarai MD. High prevalence of diabetes and impaired fasting glycaemia in urban Nepal. Diabet Med. 2003; 20(2): 170-1.

Sipetic S, Maksimovic J, Vlajinac H, Ratkov I, Sajic S, Zdravkovic D, Sipetic T. Rising incidence of Type 1 diabetes in Belgrade children aged 0-14 years in the period from 1982 to 2005. J Endocrinol Invest. 2013; 36(5): 307-12.

Skrivarhaug T, Bangstad HJ, Stene LC, Sandvik L, Hanssen KF, Joner G. Long-term mortality in a nationwide cohort of childhood-onset type 1 diabetic patients in Norway. Diabetologia. 2006; 49(2): 298-305.

Skrivarhaug T, Stene LC, Drivvoll AK, Str?m H, Joner G. Incidence of type 1 diabetes in Norway among children aged 0-14 years between 1989 and 2012: has the incidence stopped rising? Results from the Norwegian Childhood Diabetes Registry. Diabetologia. 2014; 57(1): 57-62.

Smith KJ, Blizzard L, McNaughton SA, Gall SL, Dwyer T, Venn AJ. Daily eating frequency and cardiometabolic risk factors in young Australian adults: cross-sectional analyses. Br J Nutr. 2012; 108(6): 1086–94.

Sobel-Maruniak A, Grzywa M, Orlowska-Florek R, Staniszewski A. The rising incidence of type 1 diabetes in south-eastern Poland. A study of the 0-29 year-old age group, 1980-1999. Endokrynol Pol. 2006; 57(2): 127-30.

Söderberg S, Zimmet P, Tuomilehto J, de Courten M, Dowse GK, Chitson P, Gareeboo H, Alberti KGMM, Shaw JE. Increasing prevalence of Type 2 diabetes mellitus in all ethnic groups in Mauritius. Diabet Med. 2005; 22(1): 61-8.

Soegondo S, Widyahening IS, Istantho R, Yunir E. Prevalence of diabetes among suburban population of Ternate--a small remote island in the eastern part of Indonesia. Acta Med Indones. 2011; 43(2): 99-104.

Solet J-L, Baroux N, Pochet M, Benoit-Cattin T, De Montera A-M, Sissoko D, Favier F, Fagot-Campagna A. Prevalence of type 2 diabetes and other cardiovascular risk factors in Mayotte in 2008: the MAYDIA study. Diabetes Metab. 2011; 37(3): 201-7.

Solfrizzi V, Panza F, Colacicco AM, D'Introno A, Capurso C, Torres F, Grigoletto F, Maggi S, Del Parigi A, Reiman EM, Caselli RJ, Scafato E, Farchi G, Capurso A, Italian Longitudinal Study on Aging Working Group. Vascular risk factors, incidence of MCI, and rates of progression to dementia. Neurology. 2004; 63(10): 1882-91.

Song K-H, Nam-Goomg IS, Han S-M, Kim M-S, Lee E-J, Lee YS, Lee MS, Yoon S, Lee K-U, Park J-Y. Change in prevalence and 6-year incidence of diabetes and impaired fasting glucose in Korean subjects living in a rural area. Diabetes Res Clin Pract. 2007; 78(3): 378-84.

Songini M, Muntoni S. High incidence of type 1 diabetes in Sardinia. Gruppo Collaborativo per l'Epidemiologia dell'IDDM in Sardegna. Ann Ig. 1992; 4(3): 179-90.

Soria MLB, Sy RG, Vega BS, Ty-Willing T, Abenir-Gallardo A, Velandria F, Punzalan FE. The incidence of type 2 diabetes mellitus in the Philippines: a 9-year cohort study. Diabetes Res Clin Pract. 2009; 86(2): 130-3.

Soriguer F, Goday A, Bosch-Comas A, Bordiú E, Calle-Pascual A, Carmena R, Casamitjana R, Castaño L, Castell C, Catalá M, Delgado E, Franch J, Gaztambide S, Girbés J, Gomis R, Gutiérrez G, López-Alba A, Martínez-Larrad MT, Menéndez E, Mora-Peces I, Ortega E, Pascual-Manich G, Rojo-Martínez G, Serrano-Rios M, Valdés S, Vázquez JA, Vendrell J. Prevalence of diabetes mellitus and impaired glucose regulation in Spain: the Di@bet.es Study. Diabetologia. 2012; 55(1): 88-93.

Soriguer F, Rojo-Martínez G, Almaraz MC, Esteva I, Ruiz de Adana MS, Morcillo S, Valdés S, García-Fuentes E, García-Escobar E, Cardona I, Gomez-Zumaquero JM, Olveira-Fuster G. Incidence of type 2 diabetes in southern Spain (Pizarra Study). Eur J Clin Invest. 2008; 38(2): 126-33.

Sossa C, Delisle H, Agueh V, Sodjinou R, Ntandou G, Makoutode M. Lifestyle and dietary factors associated with the evolution of cardiometabolic risk over four years in West-African adults: the Benin study. J Obes. 2013; 2013: 298024.

Soysal A, Demiral Y, Soysal D, Uçku R, Köseoglu M, Aksakoglu G. The prevalence of metabolic syndrome among young adults in Izmir, Turkey. Anadolu Kardiyol Derg. 2005; 5(3): 196-201.

Spaans EA, Gusdorf LM, Groenier KH, Brand PL, Veeze HJ, Reeser HM, Bilo HJ, Kleefstra N. The incidence of type 1 diabetes is still increasing in the Netherlands, but has stabilised in children under five (Young DUDEs-1). Acta Paediatr. 2015; 104(6): 626-9.

Statistics Canada (StatCan). Canada CANSIM Database - Cardiovascular Health Measures of the Household Population 2012-2013.

Statistics Canada (StatCan). Canada CANSIM Database - Cardiovascular Health Measures of the Household Population 2014-2015.

Statistics Canada (StatCan). Canada CANSIM Database - Cardiovascular Health Measures of the Household Population 2016-2017.

Statistics Canada (StatCan). Canada CANSIM Database - Cardiovascular Health Measures of the Household Population 2018-2019.

Stipancic G, La Grasta Sabolic L, Malenica M, Radica A, Skrabic V, Tiljak MK. Incidence and trends of childhood Type 1 diabetes in Croatia from 1995 to 2003. Diabetes Res Clin Pract. 2008; 80(1): 122-7.

Subramani SK, Yadav D, Mishra M, Pakkirisamy U, Mathiyalagen P, Prasad G. Prevalence of Type 2 Diabetes and Prediabetes in the Gwalior-Chambal Region of Central India. Int J Environ Res Public Health. 2019; 16(23).

Sutton DL, Lyle DM, Pierce JP. Incidence and prevalence of insulin-dependent diabetes mellitus in the zero- to 19-years' age-group in Sydney. Med J Aust. 1989; 151(3): 140-6.

Suvd J, Gerel B, Otgooloi H, Purevsuren D, Zolzaya H, Roglic G, King H. Glucose intolerance and associated factors in Mongolia: results of a national survey. Diabet Med. 2002; 19(6): 502–8.

Swai AB, Mclarty DG, Mtinangi BL, Tatala S, Kitange HM, Mlingi N, Rosling H, Howlett WP, Brubaker GR, Alberti KG. Diabetes is not caused by cassava toxicity. A study in a Tanzanian community. Diabetes Care. 1992; 15(10): 1378-85.

Swedish Association for Diabetology, Vastra Gotaland Registry Center. Sweden National Diabetes Register Tables 2008-2012.

Swedish National Diabetes Register. Sweden National Diabetes Register Annual Report 2022. Swedish National Diabetes Register.

Swedish National Diabetes Register. Sweden National Diabetes Register Annual Report 2023. Swedish National Diabetes Register.

Sy RG, Morales DD, Dans AL, Paz-Pacheco E, Punzalan FER, Abelardo NS, Duante CA. Prevalence of atherosclerosis-related risk factors and diseases in the Philippines. J Epidemiol. 2012; 22(5): 440-7.

Tahapary DL, Harbuwono DS, Yunir E, Soewondo P. Diagnosing metabolic syndrome in a multi-ethnic country: is an ethnic-specific cut-off point of waist circumference needed?. Nutr Diabetes. 2020; 10(1): 19.

Tahirovic H, Toromanovic A. Incidence of type 1 diabetes mellitus in children in Tuzla Canton between 1995 and 2004. Eur J Pediatr. 2007; 166(5): 491-2.

Tan WS, Ng CJ, Khoo E-M, Low W-Y, Tan HM. The triad of erectile dysfunction, testosterone deficiency syndrome and metabolic syndrome: findings from a multi-ethnic Asian men study (The Subang Men’s Health Study). Aging Male. 2011; 14(4): 231–6.

Taplin CE, Craig ME, Lloyd M, Taylor C, Crock P, Silink M, Howard NJ. The rising incidence of childhood type 1 diabetes in New South Wales, 1990-2002. Med J Aust. 2005; 183(5): 243-6.

Taylor KS, Heneghan CJ, Farmer AJ, Fuller AM, Adler AI, Aronson JK, Stevens RJ. All-cause and cardiovascular mortality in middle-aged people with type 2 diabetes compared with people without diabetes in a large U.K. primary care database. Diabetes Care. 2013; 36(8): 2366-71.

Teeaar T, Liivak N, Heilman K, Kool P, Sor R, Paal M, Einberg U, Tillmann V. Increasing incidence of childhood-onset type 1 diabetes mellitus among Estonian children in 1999-2006. Time trend analysis 1983-2006. Pediatr Diabetes. 2010; 11(2): 107-10.

Temmar M, Labat C, Benkhedda S, Charifi M, Thomas F, Bouafia MT, Bean K, Darne B, Safar ME, Benetos A. Prevalence and determinants of hypertension in the Algerian Sahara. J Hypertens. 2007; 25(11): 2218-26.

Tenconi MT, Devoti G, Albani I, Lorini R, Martinetti M, Fratino P, Ferrari E, Ferrero E, Severi F. IDDM in the province of Pavia, Italy, from a population-based registry.A descriptive study. Diabetes Care. 1995; 18(7): 1017-9.

Tesfaye TD, Temesgen WA, Kasa AS, Yismaw YS. Prevalence and associated factors of hypertension in Amhara regional state city and its' surrounding rural districts: a community-based cross-sectional study. Afr Health Sci. 2019; 19(3): 2580-2590.

Thakur JS, Nangia R. Prevalence, Awareness, Treatment, and Control of Hypertension and Diabetes: Results From Two State-Wide STEPS Survey in Punjab and Haryana, India. Front Public Health. 2022; 10: 768471.

Thankappan KR, Shah B, Mathur P, Sarma PS, Srinivas G, Mini GK, Daivadanam M, Soman B, Vasan RS. Risk factor profile for chronic non-communicable diseases: results of a community-based study in Kerala, India. Indian J Med Res. 2010; 131: 53-63.

Thomas W, Birgit R, Edith S. Changing geographical distribution of diabetes mellitus type 1 incidence in Austrian children 1989--2005. Eur J Epidemiol. 2008; 23(3): 213-8.

Thuesen BH, Cerqueira C, Aadahl M, Ebstrup JF, Toft U, Thyssen JP, Fenger RV, Hersoug L-G, Elberling J, Pedersen O, Hansen T, Johansen JD, Jørgensen T, Linneberg A. Cohort Profile: the Health2006 cohort, research centre for prevention and health. Int J Epidemiol. 2014; 43(2): 568–75.

Thunander M, Petersson C, Jonzon K, Fornander J, Ossiansson B, Torn C, Edvardsson S, Landin-Olsson M. Incidence of type 1 and type 2 diabetes in adults and children in Kronoberg, Sweden. Diabetes Res Clin Pract. 2008; 82(2): 247-55.

Tian H, Song G, Xie H, Zhang H, Tuomilehto J, Hu G. Prevalence of diabetes and impaired fasting glucose among 769,792 rural Chinese adults. Diabetes Res Clin Pract. 2009; 84(3): 273-8.

Torquato MT, Montenegro Junior RM, Viana LA, de Souza RA, Lanna CM, Lucas JC, Bidurin C, Foss MC. Prevalence of diabetes mellitus and impaired glucose tolerance in the urban population aged 30-69 years in Ribeirao Preto (Sao Paulo), Brazil. Sao Paulo Med J. 2003; 121(6): 224-30.

Touhami M, Zennaki A, Bouchetara A, Naceur M, Aoui A, Gharnouti M, Latroch C, Bouziane-Nedjadi K, Boudraa G. Epidemiological Evolution of Type 1 Diabetes in Children: Data from the Register of the Department of Oran, Algeria, 1973-2017. Rev Epidemiol Sante Publique. 2019; 67(6): 369-374.

Tran Quang B, Pham Tran P, Nguyen Thanh C, Bui Thi N, Do Dinh T, Tran Quang T, Duong Tuan L, Bui Thi Thuy N, Nguyen Anh N. High incidence of type 2 diabetes in a population with normal range body mass index and individual prediction nomogram in Vietnam. Diabet Med. 2022; 39(2): e14680.

Tsirona S, Katsaros F, Bargiota A, Polyzos SA, Arapoglou G, Koukoulis GN. Prevalence and determinants of type 2 diabetes mellitus in a Greek adult population. Hormones (Athens). 2016; 15(1): 88–98.

Tufton N, Chowdhury T. Prevalence of Diabetes on Santa Cruz Island in Galapagos Archipelago. Prev Chronic Dis. 2015; E94.

Tung JY, Kwan EY, But BW, Wong WH, Fu AC, Pang G, Tsang JW, Yau HC, Belaramani K, Wong LM, Wong SM, Lo P, Ng KL, Yeung WK, Chan KT, Chan AM, Wong SW, Tay MK, Chung J, Lee CY, Lam YY, Cheung PT. Increasing incidence of type 1 diabetes among Hong Kong children and adolescents: The Hong Kong Childhood Diabetes Registry 2008 to 2017. Pediatr Diabetes. 2020; 21(5): 713-719.

Tunstall-Pedoe H, Woodward M, Tavendale R, Brook RA, McCluskey MK. Comparison of the prediction by 27 different factors of coronary heart disease and death in men and women of the Scottish heart health study: cohort study. BMJ. 1997; 315(7110): 722-729.

Tuomilehto J, Karvonen M, Pitkaniemi J, Virtala E, Kohtamaki K, Toivanen L, Tuomilehto-Wolf E. Record-high incidence of Type I (insulin-dependent) diabetes mellitus in Finnish children. The Finnish Childhood Type I Diabetes Registry Group. Diabetologia. 1999; 42(6): 655-60.

Tuomilehto J, Nissinen A, Kivelä SL, Pekkanen J, Kaarsalo E, Wolf E, Aro A, Punsar S, Karvonen MJ. Prevalence of diabetes mellitus in elderly men aged 65 to 84 years in eastern and western Finland. Diabetologia. 1986; 29(9): 611-5.

Tuomilehto J, Podar T, Brigis G, Urbonaite B, Rewers M, Adojaan B, Cepaitis Z, Kalits I, King H, LaPorte R. Comparison of the incidence of insulin-dependent diabetes mellitus in childhood among five Baltic populations during 1983-1988. Int J Epidemiol. 1992; 21(3): 518-27.

Tuomilehto J, Rewers M, Reunanen A, Lounamaa P, Lounamaa R, Tuomilehto-Wolf E, Akerblom HK. Increasing trend in type 1 (insulin-dependent) diabetes mellitus in childhood in Finland. Analysis of age, calendar time and birth cohort effects during 1965 to 1984. Diabetologia. 1991; 34(4): 282-7.

Tuomilehto J, Virtala E, Karvonen M, Lounamaa R, Pitk„niemi J, Reunanen A, Tuomilehto-Wolf E, Toivanen L. Increase in incidence of insulin-dependent diabetes mellitus among children in Finland. Int J Epidemiol. 1995; 24(5): 984-92.

Ufa Eye Research Institute. Russia - Ural Eye and Medical Study 2015-2017.

Ufa Eye Research Institute. Russia Ural Very Old Study 2017-2020.

Ulmer H, Kelleher CC, Fitz-Simon N, Diem G, Concin H. Secular trends in cardiovascular risk factors: an age-period cohort analysis of 698,954 health examinations in 181,350 Austrian men and women. J Intern Med. 2007; 261(6): 566-76.

Umeå University. Sweden - Norrbotten and Västerbotten Northern Sweden MONICA Study 1986.

Umeå University. Sweden - Norrbotten and Västerbotten Northern Sweden MONICA Study 1990.

Umeå University. Sweden - Norrbotten and Västerbotten Northern Sweden MONICA Study 1994.

Umeå University. Sweden - Norrbotten and Västerbotten Northern Sweden MONICA Study 1999.

Umeå University. Sweden - Norrbotten and Västerbotten Northern Sweden MONICA Study 2004.

Umeå University. Sweden - Norrbotten and Västerbotten Northern Sweden MONICA Study 2009.

Umeå University. Sweden - Norrbotten and Västerbotten Northern Sweden MONICA Study 2014.

Unal B, Sozmen K, Ucku R, Ergor G, Soysal A, Baydur H, Meseri R, Simsek H, Gerceklioglu G, Doganay S, Budak R, Kilic B, Gunay T, Ergor A, Demiral Y, Aslan O, Cimrin D, Akvardar Y, Tuncel P. High prevalence of cardiovascular risk factors in a Western urban Turkish population: a community-based study. Anadolu Kardiyol Derg. 2013; 13(1): 9–17.

University of Malta. Malta Health and Wellbeing Study 2014-2016.

University of the West Indies. Jamaica Health and Lifestyle Survey 2007-2008.

Upmeier E, Vire J, Korhonen MJ, Isoaho H, Lehtonen A, Arve S, Wuorela M, Viitanen M. Cardiovascular risk profile and use of statins at the age of 70 years: a comparison of two Finnish birth cohorts born 20 years apart. Age Ageing. 2016; 45(1): 84-90.

Valent F, Candido R, Faleschini E, Tonutti L, Tortul C, Zanatta M, Zanette G, Zanier L. The incidence rate and prevalence of pediatric type 1 diabetes mellitus (age 0-18) in the Italian region Friuli Venezia Giulia: population-based estimates through the analysis of health administrative databases. Acta Diabetol. 2016; 53(4): 629–35.

Velmurugan G, Mohanraj S, Dhivakar M, Veerasekar G, Brag-Gresham J, He K, Alexander T, Cherian M, Saran R, Pradeep T, Swaminathan K. Differential risk factor profile of diabetes and atherosclerosis in rural, sub-urban and urban regions of South India: The KMCH-Non-communicable disease studies. Diabet Med. 2021; 38(6): e14466.

Verrillo A, de Teresa A, La Rocca S, Giarrusso PC. Prevalence of diabetes mellitus and impaired glucose tolerance in a rural area of Italy. Diabetes Res. 1985; 2(6): 301-6.

Vijayakumar G, Manghat S, Vijayakumar R, Simon L, Scaria LM, Vijayakumar A, Sreehari GK, Kutty VR, Rachana A, Jaleel A. Incidence of type 2 diabetes mellitus and prediabetes in Kerala, India: results from a 10-year prospective cohort. BMC Public Health. 2019; 19(1): 140.

Vilbergsson S, Sigurdsson G, Sigvaldason H, Sigfusson N. Coronary heart disease mortality amongst non-insulin-dependent diabetic subjects in Iceland: the independent effect of diabetes. The Reykjavik Study 17-year follow up. J Intern Med. 1998; 244(4): 309-16.

Wadsworth E, Shield J, Hunt L, Baum D. Insulin dependent diabetes in children under 5: incidence and ascertainment validation for 1992. BMJ. 1995; 310(6981): 700-3.

Waernbaum I, Blohmé G, Ostman J, Sundkvist G, Eriksson JW, Arnqvist HJ, Bolinder J, Nyström L. Excess mortality in incident cases of diabetes mellitus aged 15 to 34 years at diagnosis: a population-based study (DISS) in Sweden. Diabetologia. 2006; 49(4): 653-9.

Walters DP, Gatling W, Houston AC, Mullee MA, Julious SA, Hill RD. Mortality in diabetic subjects: an eleven-year follow-up of a community-based population. Diabet Med. 1994; 11(10): 968-73.

Wandell PE, Carlsson AC, de Faire U, Hellenius M-L. Prevalence of blood lipid disturbances in Swedish and foreign-born 60-year-old men and women in Stockholm, Sweden. Nutr Metab Cardiovasc Dis. 2011; 21(3): 173–81.

Wang B, Cheng J, Wan H, Wang Y, Zhang W, Chen Y, Chen C, Xia F, Jensen MD, Wang N, Lu Y. Early-life exposure to the Chinese famine, genetic susceptibility and the risk of type 2 diabetes in adulthood. Diabetologia. 2021; 64(8): 1766-1774.

Wang C, Hou X, Bao Y, Pan J, Zuo Y, Zhong W, Jia W, Xiang K. The metabolic syndrome increased risk of cardiovascular events in Chinese--a community based study. Int J Cardiol. 2010; 139(2): 159–65.

Wang F, Ye P, Luo L, Xiao W, Qi L, Bian S, Wu H, Sheng L, Xiao T, Xu R. Association of serum lipids with arterial stiffness in a population-based study in Beijing. Eur J Clin Invest. 2011; 41(9): 929–36.

Wang H, Qiu Q, Tan LL, Liu T, Deng XQ, Chen YM, Chen W, Yu XQ, Hu BJ, Chen WQ. Prevalence and determinants of diabetes and impaired fasting glucose among urban community-dwelling adults in Guangzhou, China. Diabetes Metab. 2009; 35(5): 378-84.

Wang M, Jin Y, Dai T, Yu C, Zheng S, Nie Y, Bai Y. Association between ambient particulate matter (PM10) and incidence of diabetes in northwest of China: A prospective cohort study. Ecotoxicol Environ Saf. 2020; 202: 110880.

Wang R, Zhang P, Li Z, Lv X, Cai H, Gao C, Song Y, Yu Y, Li B, Cui Y. The prevalence of pre-diabetes and diabetes and their associated factors in Northeast China: a cross-sectional study. Sci Rep. 2019; 9(1): 2513.

Wang R, Zhang P, Lv X, Jiang L, Gao C, Song Y, Yu Y, Li B. Situation of Diabetes and Related Disease Surveillance in Rural Areas of Jilin Province, Northeast China. Int J Environ Res Public Health. 2016; 13(6).

Wang S, Ma W, Yuan Z, Wang S-M, Yi X, Jia H, Xue F. Association between obesity indices and type 2 diabetes mellitus among middle-aged and elderly people in Jinan, China: a cross-sectional study. BMJ Open. 2016; 6(11): e012742.

Wang T, Bi Y, Xu M, Huang Y, Xu Y, Li X, Wang W, Ning G. Serum uric acid associates with the incidence of type 2 diabetes in a prospective cohort of middle-aged and elderly Chinese. Endocrine. 2011; 40(1): 109–16.

Wang X, Luan D, Xin S, Liu Y, Gao Q. Association Between Individual Components of Metabolic Syndrome and Cognitive Function in Northeast Rural China. Am J Alzheimers Dis Other Demen. 2019; 34(7-8): 507-512.

Warsy AS, el-Hazmi MA. Diabetes mellitus, hypertension and obesity--common multifactorial disorders in Saudis. East Mediterr Health J. 1999; 5(6): 1236-42.

Washington RE, Orchard TJ, Arena VC, Laporte RE, Tull ES. Incidence of type 1 and type 2 diabetes in youth in the U.S. Virgin Islands, 2001-2010. Pediatr Diabetes. 2013; 14(4): 280-7.

Waspadji S, Ranakusuma AB, Suyono S, Supartondo S, Sukaton U. Diabetes mellitus in an urban population in Jakarta, Indonesia. Tohoku J Exp Med. 1983; 141(Suppl): 219-28.

Weets I, De Leeuw IH, Du Caju MV, Rooman R, Keymeulen B, Mathieu C, Rottiers R, Daubresse JC, Rocour-Brumioul D, Pipeleers DG, Gorus FK. The incidence of type 1 diabetes in the age group 0-39 years has not increased in Antwerp (Belgium) between 1989 and 2000: evidence for earlier disease manifestation. Diabetes Care. 2002; 25(5): 840-6.

Wei Q, Sun J, Huang J, Zhou HY, Ding YM, Tao YC, He SM, Liu YL, Niu JQ. Prevalence of hypertension and associated risk factors in Dehui City of Jilin Province in China. J Hum Hypertens. 2015; 29(1): 64–8.

Weinstein, Maxine, Noreen Goldman, Ming-Cheng Chang, Hui-Sheng Lin, Yi-Li Chuang, Christine E. Peterson, Dana A. Glei, Baai-Shyun Hurng, Yu-Hsuan Lin, Shu-Hui Lin, I-Wen Liu, Hsia-Yuan Liu, Shio-Jean Lin, Chun-Ming Wu, Mei-Ling Hsiao, and Shiow-lng Wu. Social Environment and Biomarkers of Aging Study (SEBAS) in Taiwan, 2000 and 2006 [Computer file]. ICPSR 3792.vS. Ann Arbor, MI: Inter-university Consortium/or Political and Social Research /distributor/, 2011-06-17. doi: 10.3886/ICPSR03792

Weinstein, Maxine, Noreen Goldman, Ming-Cheng Chang, Hui-Sheng Lin, Yi-Li Chuang, Christine E. Peterson, Dana A. Glei, Baai-Shyun Hurng, Yu-Hsuan Lin, Shu-Hui Lin, I-Wen Liu, Hsia-Yuan Liu, Shio-Jean Lin, Chun-Ming Wu, Mei-Ling Hsiao, and Shiow-lng Wu. Social Environment and Biomarkers of Aging Study (SEBAS) in Taiwan, 2000 and 2006 [Computer file]. ICPSR 3792.vS. Ann Arbor, MI: Inter-university Consortium/or Political and Social Research /distributor/, 2011-06-17. doi: 10.3886/ICPSR03792

Weng J, Zhou Z, Guo L, Zhu D, Ji L, Luo X, Mu Y, Jia W. Incidence of type 1 diabetes in China, 2010-13: population based study. BMJ. 2018; 360: j5295.

Wibell L, Nyström L, Ostman J, Arnqvist H, Blohmé G, Lithner F, Littorin B, Sundkvist G. Increased mortality in diabetes during the first 10 years of the disease. A population-based study (DISS) in Swedish adults 15-34 years old at diagnosis. J Intern Med. 2001; 249(3): 263-70.

Wijewardene K, Mohideen MR, Mendis S, Fernando DS, Kulathilaka T, Weerasekara D, Uluwitta P. Prevalence of hypertension, diabetes and obesity: baseline findings of a population based survey in four provinces in Sri Lanka. Ceylon Med J. 2005; 50(2): 62-70.

Williams ED, Tapp RJ, Magliano DJ, Shaw JE, Zimmet PZ, Oldenburg BF. Health behaviours, socioeconomic status and diabetes incidence: the Australian Diabetes Obesity and Lifestyle Study (AusDiab). Diabetologia. 2010; 53(12): 2538-45.

Willis J, Cunningham-Tisdall C, Griffin C, Scott R, Darlow BA, Owens N, Ferguson J, Mackenzie K, Williman J, de Bock M. Type 1 diabetes diagnosed before age 15years in Canterbury, New Zealand: A 50 year record of increasing incidence. Pediatr Diabetes. 2022; 23(3): 301-309.

Wittek A, Sokalski B, Grzeszczak W, Strojek K. Prevalence of diabetes and cardiovascular risk factors of industrial area in southern Poland. Exp Clin Endocrinol Diabetes. 2009; 117(7): 350-3.

Wong JS, Pearson DW, Murchison LE, Williams MJ, Narayan V. Mortality in diabetes mellitus: experience of a geographically defined population. Diabet Med. 1991; 8(2): 135-9.

World Health Organization (WHO), Ministry of Health (Mongolia), National Medical Research Institute (Mongolia), Health Sciences University (Mongolia), National Oncology Center of Mongolia (Mongolia). Mongolia STEPS Noncommunicable Disease Risk Factors Survey 2005.

World Health Organization (WHO), Ministry of Health (Mongolia), National Medical Research Institute (Mongolia), Health Sciences University (Mongolia), National Oncology Center of Mongolia (Mongolia). Mongolia STEPS Noncommunicable Disease Risk Factors Survey 2009.

World Health Organization (WHO), Ministry of Health (Seychelles), Institute of Social and Preventive Medicine, University of Lausanne (Switzerland), University Hospital Center (Switzerland). Seychelles STEPS Noncommunicable Disease Risk Factors Survey 2004.

World Health Organization (WHO), Ministry of Health and Medical Education (Iran), Center for Non-Communicable Diseases Control (Iran). Iran STEPS Noncommunicable Disease Risk Factors Survey 2005.

World Health Organization (WHO). Armenia STEPS Noncommunicable Disease Risk Factors Survey 2016.

World Health Organization (WHO). Brunei STEPS Noncommunicable Disease Risk Factors Survey 2015-2016.

World Health Organization (WHO). Cape Verde STEPS Noncommunicable Disease Risk Factors Survey 2007.

World Health Organization (WHO). Central African Republic - Bangui and Ombella M'Poko STEPS Noncommunicable Disease Risk Factors Survey 2017. WHO NCD Microdata Repository, 2020.

World Health Organization (WHO). Central African Republic - Bangui STEPS Noncommunicable Disease Risk Factors Survey 2010.

World Health Organization (WHO). Comoros STEPS Noncommunicable Disease Risk Factors Survey 2011.

World Health Organization (WHO). Eritrea STEPS Noncommunicable Disease Risk Factors Survey 2010.

World Health Organization (WHO). Fiji STEPS Noncommunicable Disease Risk Factors Survey 2011.

World Health Organization (WHO). Georgia STEPS Noncommunicable Disease Risk Factors Survey 2010.

World Health Organization (WHO). Guatemala STEPS Noncommunicable Disease Risk Factors Survey 2015.

World Health Organization (WHO). Guinea STEPS Noncommunicable Disease Risk Factors Survey 2009.

World Health Organization (WHO). Kazakhstan - Aktobe Oblast STEPS Noncommunicable Disease Risk Factors Survey 2016.

World Health Organization (WHO). Kazakhstan - Almaty STEPS Noncommunicable Disease Risk Factors Survey 2015.

World Health Organization (WHO). Kazakhstan - Shymkent STEPS Noncommunicable Disease Risk Factors Survey 2015.

World Health Organization (WHO). Laos STEPS Noncommunicable Disease Risk Factors Survey 2013. WHO NCD Microdata Repository, 2019.

World Health Organization (WHO). Liberia STEPS Noncommunicable Disease Risk Factors Survey 2011.

World Health Organization (WHO). Malawi STEPS Noncommunicable Disease Risk Factors Survey 2017.

World Health Organization (WHO). Mali STEPS Noncommunicable Disease Risk Factors Survey 2007.

World Health Organization (WHO). Micronesia - Kosrae STEPS Noncommunicable Disease Risk Factors Survey 2009.

World Health Organization (WHO). Micronesia - Pohnpei STEPS Noncommunicable Disease Risk Factors Survey 2008 .

World Health Organization (WHO). Micronesia - Yap STEPS Noncommunicable Disease Risk Factors Survey 2009.

World Health Organization (WHO). Niue STEPS Noncommunicable Disease Risk Factors Survey 2011-2012.

World Health Organization (WHO). Papua New Guinea STEPS Noncommunicable Disease Risk Factors Survey 2007-2008.

World Health Organization (WHO). Sao Tome and Principe STEPS Noncommunicable Disease Risk Factors Survey 2008.

World Health Organization (WHO). Vietnam - C?n Tho STEPS Noncommunicable Disease Risk Factors Survey 2005.

World Health Organization (WHO). Zambia STEPS Noncommunicable Disease Risk Factors Survey 2017. Geneva, Switzerland: World Health Organization (WHO).

Wu X, Liu X, Liao W, Kang N, Dong X, Abdulai T, Zhai Z, Wang C, Wang X, Li Y. Prevalence and characteristics of alcohol consumption and risk of type 2 diabetes mellitus in rural China. BMC Public Health. 2021; 21(1): 1644.

Wyka J, Biernat J, Kiedik D. Nutritional determination of the health status in Polish elderly people from an urban environment. J Nutr Health Aging. 2010; 14(1): 67-71.

Wysocki MJ, Chanska M, Bak M, Czyzyk AS. Incidence of insulin-dependent diabetes mellitus in Warsaw, Poland, in children and young adults, 1983-1988. World Health Stat Q. 1992; 45(4): 315-20.

Xie D, Wang J. Comparison of self-reports and biomedical measurements on hypertension and diabetes among older adults in China. BMC Public Health. 2020; 20(1): 1664.

Xie J, Guan F, Wang J-H, Hu D-Y. Reasons for the upsetting cholesterol level during the community investigation from residents, physicians, and social aspects: the China Cholesterol Education Program (CCEP). Chin Med J (Engl). 2011; 124(19): 3030–4.

Xie L, Zhao X, Zhang B, Zhu H. Epidemiology and risk factors for diabetes in the suburbs of Beijing: a retrospective cohort study. BMJ Open. 2021; 11(3): e041526.

Xin Z, Yuan M-X, Li H-X, Hua L, Feng J-P, Shi J, Zhu X-R, Cao X, Yang J-K. Evaluation for fasting and 2-hour glucose and HbA1c for diagnosing diabetes based on prevalence of retinopathy in a Chinese population. PLoS One. 2012; 7(7): e40610.

Xing L, Tian Y, Jing L, Lin M, Du Z, Sun Q, Dai D, Shi L, Yang Z, Liu S. Status and disparities of diabetes among urban and rural residents aged 40 years and older: insight from a population-based study in northeast China, 2017-2019. J Epidemiol Community Health. 2021; 75(8): 800-808.

Xu J, Xu L, Du KF, Shao L, Chen CX, Zhou JQ, Wang YX, You QS, Jonas JB, Wei WB. Subfoveal choroidal thickness in diabetes and diabetic retinopathy [Unpublished data]. Ophthalmology. 2013 Oct;120(10):2023-8.

Xu J, Xu L, Du KF, Shao L, Chen CX, Zhou JQ, Wang YX, You QS, Jonas JB, Wei WB. Subfoveal choroidal thickness in diabetes and diabetic retinopathy [Unpublished data]. Ophthalmology. 2013 Oct;120(10):2023-8.

Xu S, Ming J, Xing Y, Gao B, Yang C, Ji Q, Chen G. Regional differences in diabetes prevalence and awareness between coastal and interior provinces in China: a population-based cross-sectional study. BMC Public Health. 2013; 299.

Xu WL, Qiu CX, Wahlin A, Winblad B, Fratiglioni L. Diabetes mellitus and risk of dementia in the Kungsholmen project: a 6-year follow-up study. Neurology. 2004; 63(7): 1181–6.

Xu Y, Wang L, He J, Bi Y, Li M, Wang T, Wang L, Jiang Y, Dai M, Lu J, Xu M, Li Y, Hu N, Li J, Mi S, Chen CS, Li G, Mu Y, Zhao J, Kong L, Chen J, Lai S, Wang W, Zhao W, Ning G. Prevalence and control of diabetes in Chinese adults. JAMA. 2013; 310(9): 948-59.

Yang WS, Chen PC, Lin HJ, Su TC, Hsu HC, Chen MF, Lee YT, Chien KL. Association between type 2 diabetes and cancer incidence in Taiwan: data from a prospective community-based cohort study. Acta Diabetol. 2017; 54(5): 455-461.

Yang Z, Wang K, Li T, Sun W, Li Y, Chang YF, Dorman JS, LaPorte RE. Childhood diabetes in China. Enormous variation by place and ethnic group. Diabetes Care. 1998; 21(4): 525-9.

Yin T, Zhang JX, Wang FX, Zhao JH, Zhao Y, Liu L, Liu XY, Zhang YH, Zhao Y. The Association Between Sarcopenic Obesity and Hypertension, Diabetes, and Abnormal Lipid Metabolism in Chinese Adults. Diabetes Metab Syndr Obes. 2021; 14: 1963-1973.

Ylihärsilä H, Lindström J, Eriksson JG, Jousilahti P, Valle TT, Sundvall J, Tuomilehto J. Prevalence of diabetes and impaired glucose regulation in 45- to 64-year-old individuals in three areas of Finland. Diabet Med. 2005; 22(1): 88-91.

Yokoyama M, Saito I, Ueno M, Kato H, Yoshida A, Kawamura R, Maruyama K, Takata Y, Osawa H, Tanigawa T, Sugiyama T. Low birthweight is associated with type2 diabetes mellitus in Japanese adults: The Toon Health Study. J Diabetes Investig. 2020; 11(6): 1643-1650.

Yu L, Cai Y, Qin R, Zhao B, Li X. Association between triglyceride glucose index and abnormal liver function in both urban and rural Chinese adult populations: Findings from two independent surveys. Medicine (Baltimore). 2019; 98(50): e18265.

Yu S, Guo X, Yang H, Zheng L, Sun Y. An update on the prevalence of metabolic syndrome and its associated factors in rural northeast China. BMC Public Health. 2014; 14: 877.

Zabeen B, Maniam J, Balsa AMM, Tayyeb S, Huda K, Azad K, Ogle GD. Incidence of diabetes in children and adolescents in Dhaka, Bangladesh. J Pediatr Endocrinol Metab. 2021; 34(4): 509-515.

Zafar J, Bhatti F, Akhtar N, Rasheed U, Bashir R, Humayun S, Waheed A, Younus F, Nazar M, Umaimato. Prevalence and risk factors for diabetes mellitus in a selected urban population of a city in Punjab. J Pak Med Assoc. 2011; 61(1): 40-7.

Zatonska K, Basiak-Rasala A, Rózanska D, Karczewski M, Wolyniec M, Szuba A, Poltyn-Zaradna K. Changes in diabetes prevalence and corresponding risk factors - findings from 3- and 6-year follow-up of PURE Poland cohort study. BMC Public Health. 2020; 20(1): 843.

Zatonska K, Ilow R, Regulska-Ilow B, Rózanska D, Szuba A, Wolyniec M, Einhorn J, Vatten L, Asvold BO, Manczuk M, Zatonski WA. Prevalence of diabetes mellitus and IFG in the prospective cohort 'PONS' study - baseline assessment. Ann Agric Environ Med. 2011; 18(2): 265-9.

Zhan Y, Zhuang J, Dong Y, Xu H, Hu D, Yu J. Predicting the prevalence of peripheral arterial diseases: modelling and validation in different cohorts. VASA. 2016; 45(1): 31-9.

Zhang H, Qi D, Gu H, Wang T, Wu Y, Li J, Ni J, Liu J, Tu J, Ning X, Wang J. Trends in the prevalence, awareness, treatment and control of diabetes in rural areas of northern China from 1992 to 2011. J Diabetes Investig. 2020; 11(1): 241-249.

Zhang H, Xu W, Dahl AK, Xu Z, Wang H-X, Qi X. Relation of socio-economic status to impaired fasting glucose and Type 2 diabetes: findings based on a large population-based cross-sectional study in Tianjin, China. Diabet Med. 2013; 30(5): e157–62.

Zhang J, Cheng N, Ma Y, Li H, Cheng Z, Yang Y, He C, Li J, Pu H, Shen X, Ren X, Shi D, Pu R, Gan T, Ding J, Zheng T, Bai Y. Liver Enzymes, Fatty Liver and Type 2 Diabetes Mellitus in a Jinchang Cohort: A Prospective Study in Adults. Can J Diabetes. 2018; 42(6): 652-658.

Zhang J, Lim K, Shin S. Dairy product consumption and type 2 diabetes among Korean adults: a prospective cohort study based on the Health Examinees (HEXA) study. Epidemiol Health. 2022; 44: e2022019.

Zhang L, Qin L-Q, Liu A-P, Wang P-Y. Prevalence of risk factors for cardiovascular disease and their associations with diet and physical activity in suburban Beijing, China. J Epidemiol. 2010; 20(3): 237–43.

Zhang L, Zhang W-H, Zhang L, Wang P-Y. Prevalence of overweight/obesity and its associations with hypertension, diabetes, dyslipidemia, and metabolic syndrome: a survey in the suburban area of Beijing, 2007. Obes Facts. 2011; 4(4): 284-9.

Zhang S, Mwiberi S, Pickford R, Breitner S, Huth C, Koenig W, Rathmann W, Herder C, Roden M, Cyrys J, Peters A, Wolf K, Schneider A. Longitudinal associations between ambient air pollution and insulin sensitivity: results from the KORA cohort study. Lancet Planet Health. 2021; 5(1): e39-e49.

Zhang S, Tong W, Xu T, Wu B, Zhang Y. Diabetes and impaired fasting glucose in Mongolian population, Inner Mongolia, China. Diabetes Res Clin Pract. 2009; 86(2): 124-9.

Zhang YH, Ma WJ, Thomas GN, Xu YJ, Lao XQ, Xu XJ, Song XL, Xu HF, Cai QM, Xia L, Nie SP, Deng HH, Yu IT. Diabetes and pre-diabetes as determined by glycated haemoglobin A1c and glucose levels in a developing southern Chinese population. PLoS One. 2012; 7(5): e37260.

Zhao Y, Yang K, Wang F, Liang Y, Peng Y, Shen R, Wong T, Wang N. Associations between metabolic syndrome and syndrome components and retinal microvascular signs in a rural Chinese population: the Handan Eye Study. Graefes Arch Clin Exp Ophthalmol. 2012; 250(12): 1755–63.

Zhao Z, Sun C, Wang C, Li P, Wang W, Ye J, Gu X, Wang X, Shen S, Zhi D, Lu Z, Ye R, Cheng R, Xi L, Li X, Zheng Z, Zhang M, Luo F. Rapidly rising incidence of childhood type 1 diabetes in Chinese population: epidemiology in Shanghai during 1997-2011. Acta Diabetol. 2014; 51(6): 947-53.

Zhou X, Guan H, Zheng L, Li Z, Guo X, Yang H, Yu S, Sun G, Li W, Hu W, Guo L, Pan G, Xing L, Zhang Y, Sun Y. Prevalence and awareness of diabetes mellitus among a rural population in China: results from Liaoning Province. Diabet Med. 2015; 32(3): 332-42.

Zhou X, Ji L, Luo Y, Han X, Zhang X, Sun X, Ren Q, Qiao Q. Risk factors associated with the presence of diabetes in Chinese communities in Beijing. Diabetes Res Clin Pract. 2009; 86(3): 233–8.

Zhuang J, Zhan Y, Zhang F, Tang Z, Wang J, Sun Y, Ding R, Hu D, Yu J. Self-reported insomnia and coronary heart disease in the elderly. Clin Exp Hypertens. 2016; 38(1): 51–5.

Zung A, Blumenfeld O, Shehadeh N, Dally Gottfried O, Tenenbaum Rakover Y, Hershkovitz E, Gillis D, Zangen D, Pinhas-Hamiel O, Hanukoglu A, Rachmiel M, Shalitin S. Increase in the incidence of type 1 diabetes in Israeli children following the Second Lebanon War. Pediatr Diabetes. 2012; 13(4): 326-33.

## Table S21. Global Burden of Disease 2023 location hierarchy

| **Location** | **Level** |
| --- | --- |
| **Global** | **0** |
| **Central Europe, Eastern Europe, and Central Asia** | **1** |
| **Central Asia** | **2** |
| Armenia | 3 |
| Azerbaijan | 3 |
| Georgia | 3 |
| Kazakhstan | 3 |
| Kyrgyzstan | 3 |
| Mongolia | 3 |
| Tajikistan | 3 |
| Turkmenistan | 3 |
| Uzbekistan | 3 |
| **Central Europe** | **2** |
| Albania | 3 |
| Bosnia and Herzegovina | 3 |
| Bulgaria | 3 |
| Croatia | 3 |
| Czechia | 3 |
| Hungary | 3 |
| Montenegro | 3 |
| North Macedonia | 3 |
| Poland | 3 |
| Romania | 3 |
| Serbia | 3 |
| Slovakia | 3 |
| Slovenia | 3 |
| **Eastern Europe** | **2** |
| Belarus | 3 |
| Estonia | 3 |
| Latvia | 3 |
| Lithuania | 3 |
| Moldova | 3 |
| Russia | 3 |
| Ukraine | 3 |
| **High income** | **1** |
| **Australasia** | **2** |
| Australia | 3 |
| New Zealand | 3 |
| **High-income Asia Pacific** | **2** |
| Brunei | 3 |
| Japan | 3 |
| Singapore | 3 |
| South Korea | 3 |
| **High-income North America** | **2** |
| Canada | 3 |
| Greenland | 3 |
| USA | 3 |
| **Southern Latin America** | **2** |
| Argentina | 3 |
| Chile | 3 |
| Uruguay | 3 |
| **Western Europe** | **2** |
| Andorra | 3 |
| Austria | 3 |
| Belgium | 3 |
| Cyprus | 3 |
| Denmark | 3 |
| Finland | 3 |
| France | 3 |
| Germany | 3 |
| Greece | 3 |
| Iceland | 3 |
| Ireland | 3 |
| Israel | 3 |
| Italy | 3 |
| Luxembourg | 3 |
| Malta | 3 |
| Monaco | 3 |
| Netherlands | 3 |
| Norway | 3 |
| Portugal | 3 |
| San Marino | 3 |
| Spain | 3 |
| Sweden | 3 |
| Switzerland | 3 |
| UK | 3 |
| **Latin America and Caribbean** | **1** |
| **Andean Latin America** | **2** |
| Bolivia | 3 |
| Ecuador | 3 |
| Peru | 3 |
| **Caribbean** | **2** |
| Antigua and Barbuda | 3 |
| Barbados | 3 |
| Belize | 3 |
| Bermuda | 3 |
| The Bahamas | 3 |
| Cuba | 3 |
| Dominica | 3 |
| Dominican Republic | 3 |
| Grenada | 3 |
| Guyana | 3 |
| Haiti | 3 |
| Jamaica | 3 |
| Puerto Rico | 3 |
| Saint Kitts and Nevis | 3 |
| Saint Lucia | 3 |
| Saint Vincent and the Grenadines | 3 |
| Suriname | 3 |
| Trinidad and Tobago | 3 |
| Virgin Islands | 3 |
| **Central Latin America** | **2** |
| Venezuela | 3 |
| Colombia | 3 |
| Costa Rica | 3 |
| El Salvador | 3 |
| Guatemala | 3 |
| Honduras | 3 |
| Mexico | 3 |
| Nicaragua | 3 |
| Panama | 3 |
| **Tropical Latin America** | **2** |
| Brazil | 3 |
| Paraguay | 3 |
| **North Africa and Middle East** | **1** |
| **North Africa and Middle East** | **2** |
| Afghanistan | 3 |
| Algeria | 3 |
| Bahrain | 3 |
| Egypt | 3 |
| Iraq | 3 |
| Iran | 3 |
| Jordan | 3 |
| Kuwait | 3 |
| Lebanon | 3 |
| Libya | 3 |
| Morocco | 3 |
| Palestine | 3 |
| Oman | 3 |
| Qatar | 3 |
| Saudi Arabia | 3 |
| Sudan | 3 |
| Syria | 3 |
| Tunisia | 3 |
| Türkiye | 3 |
| United Arab Emirates | 3 |
| Yemen | 3 |
| **South Asia** | **1** |
| **South Asia** | **2** |
| Bangladesh | 3 |
| Bhutan | 3 |
| India | 3 |
| Nepal | 3 |
| Pakistan | 3 |
| **Southeast Asia, East Asia, and Oceania** | **1** |
| **East Asia** | **2** |
| China | 3 |
| North Korea | 3 |
| Taiwan (Province of China) | 3 |
| **Oceania** | **2** |
| American Samoa | 3 |
| Cook Islands | 3 |
| Federated States of Micronesia | 3 |
| Fiji | 3 |
| Guam | 3 |
| Kiribati | 3 |
| Marshall Islands | 3 |
| Nauru | 3 |
| Niue | 3 |
| Northern Mariana Islands | 3 |
| Palau | 3 |
| Papua New Guinea | 3 |
| Samoa | 3 |
| Solomon Islands | 3 |
| Tokelau | 3 |
| Tonga | 3 |
| Tuvalu | 3 |
| Vanuatu | 3 |
| **Southeast Asia** | **2** |
| Cambodia | 3 |
| Indonesia | 3 |
| Laos | 3 |
| Malaysia | 3 |
| Maldives | 3 |
| Mauritius | 3 |
| Myanmar | 3 |
| Philippines | 3 |
| Sri Lanka | 3 |
| Seychelles | 3 |
| Thailand | 3 |
| Timor-Leste | 3 |
| Vietnam | 3 |
| **Sub-Saharan Africa** | **1** |
| **Central sub-Saharan Africa** | **2** |
| Angola | 3 |
| Central African Republic | 3 |
| Congo (Brazzaville) | 3 |
| DR Congo | 3 |
| Equatorial Guinea | 3 |
| Gabon | 3 |
| **Eastern sub-Saharan Africa** | **2** |
| Burundi | 3 |
| Comoros | 3 |
| Djibouti | 3 |
| Eritrea | 3 |
| Ethiopia | 3 |
| Kenya | 3 |
| Madagascar | 3 |
| Malawi | 3 |
| Mozambique | 3 |
| Rwanda | 3 |
| Somalia | 3 |
| South Sudan | 3 |
| Tanzania | 3 |
| Uganda | 3 |
| Zambia | 3 |
| **Southern sub-Saharan Africa** | **2** |
| Botswana | 3 |
| Eswatini | 3 |
| Lesotho | 3 |
| Namibia | 3 |
| South Africa | 3 |
| Zimbabwe | 3 |
| **Western sub-Saharan Africa** | **2** |
| Benin | 3 |
| Burkina Faso | 3 |
| Cameroon | 3 |
| Cape Verde | 3 |
| Chad | 3 |
| Côte d'Ivoire | 3 |
| The Gambia | 3 |
| Ghana | 3 |
| Guinea | 3 |
| Guinea-Bissau | 3 |
| Liberia | 3 |
| Mali | 3 |
| Mauritania | 3 |
| Niger | 3 |
| Nigeria | 3 |
| São Tomé and Príncipe | 3 |
| Senegal | 3 |
| Sierra Leone | 3 |
| Togo | 3 |
